# Supplementary material for: Decarboxylation of β-boryl NHPI esters enables radical 1,2-boron shift for the assembly of versatile organoborons
Source: Nat Commun. 2023 Sep 14;14:5693. doi: 10.1038/s41467-023-41254-1 (PMC10502150; doi:10.1038/s41467-023-41254-1)
Supplement: Supplementary file 1 — Supplementary Information [file 41467_2023_41254_MOESM1_ESM.pdf]

Supplementary Information

for

Decarboxylation of *β*-Boryl NHPI Esters Enables Radical 1,2-Boron Shift for the Assembly of Versatile Organoborons

Yu Guo[1], Xiaosha Wang[1], Chengbo Li[1], Jianke Su[1], Jian Xu*[1] & Qiuling Song*[1,2,3]

[1] Institute of Next Generation Matter Transformation, College of Material Sciences Engineering, Huaqiao University, Xiamen, Fujian, 361021, P. R. China.

[2] Key Laboratory of Molecule Synthesis and Function Discovery, Fujian Province University, College of Chemistry at Fuzhou University, Fuzhou, 350108, P. R. China.

[3] School of Chemistry and Chemical Engineering, Henan Normal University, Xinxiang, Henan, 453007, P. R. China.

**email: qsong@hqu.edu.cn；jianx@hqu.edu.cn*

Table of Contents

1. Supplementary Methods 3
1.1 General information 3
1.2 General process 4
2.1 Optimization studies 15
2.2 Control experiments 27
2.3 Crystal data 31
2.4 Characterization data for Substrates 33
2.5 Characterization data for Products 44
2.6 NMR spectroscopic data 90
2.7 References 327

1. Supplementary Methods

1.1 General information

All chemicals were purchased from Leyan.com (B_2pin_2, $Ir(ppy)_3$, $[Ir(dtbppy)_3]PF_6$, $Ru(bpy)_3(PF6)_2$, 4CzIPN), Energy chemical company ($Ru(bpy)_3Cl_2$, $Ru(bpy)_3Cl_2 \cdot 6H_2O$, CuCl, Ethyl acrylate, Phenyl Acrylate). Unless otherwise stated, all experiments were conducted in a sealed tube under N_2 atmosphere. Reactions were monitored by TLC or GC-MS analysis. Flash column chromatography was performed over silica gel (200-300 mesh).

^1H-NMR and ^{13}C-NMR spectra were recorded in $CDCl_3$ and DMSO-d_6 on a Bruker Avance 500 spectrometer (500 MHz ^1H, 125 MHz ^{13}C (CPD), 470 MHz ^{19}F) at room temperature. Chemical shifts were reported in ppm on the scale relative to $CDCl_3$ (δ = 7.26 for ^1H NMR, δ = 77.00 for ^{13}C-NMR) as an internal reference. Coupling constants (*J*) were reported in Hertz (Hz).

1.2 General process

General process 1：Preparation of propargyl β-boryl NHPI esters 1.

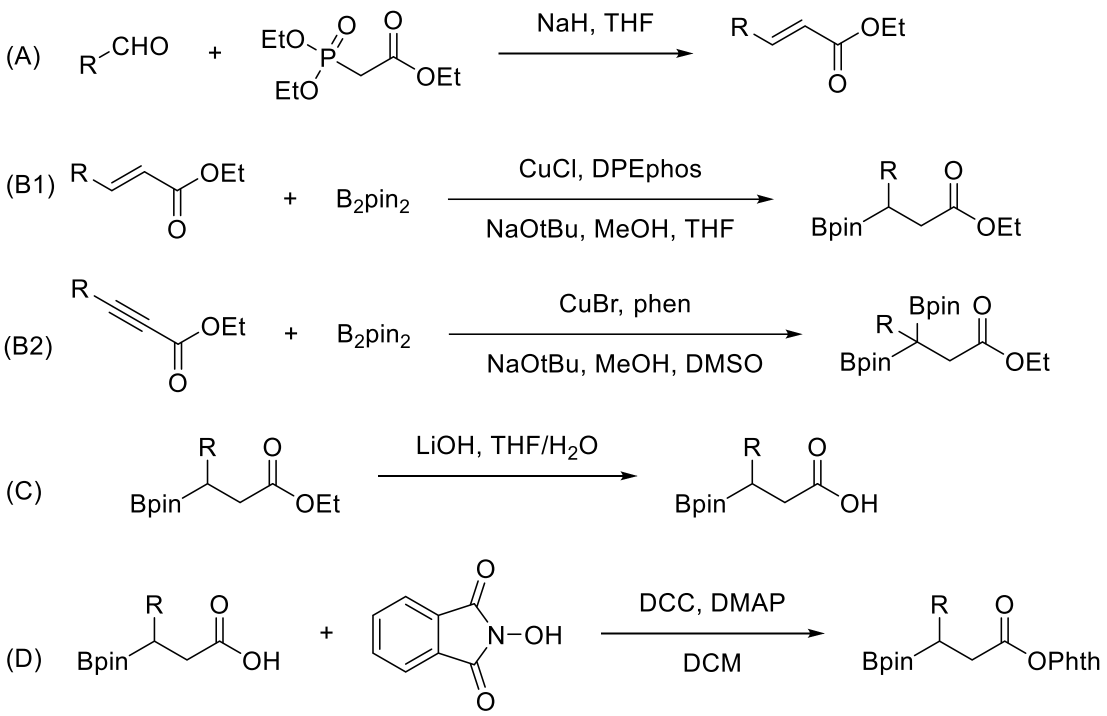

General process 1A[1]: Add triethyl phosphonoacetate (1.5 equiv) dropwise to a stirred suspension of NaH (60% on mineral oil; 1.5 equiv) in dry THF (120 mL) under nitrogen over a period of 5 minutes. Stir the resulting mixture for another 0.5 hours at 0 °C. Add a solution of aldehyde (1 equiv) in dry THF (30 mL) slowly to the resulting mixture. The reaction mixture was stirred at room temperature for 6 h. Then quench the reaction with a saturated aqueous solution of NH_4Cl. Extract the reaction mixture with Et_2O (3 × 100 mL). Wash the combined organic layer with brine. Dry the combined organic layer over $NaSO_4$. After concentration under vacuum, purify the residue by flash chromatography on silica gel (eluent: petroleum ether: EtOAc =40:1, v/v).

General process 1B1[2a]: In an oven-dried Schlenk tube were placed CuCl (0.03 equiv), NaO^{t}Bu (0.09 equiv) and DPEphos ligand (0.03 equiv). THF (1 mL) was added under argon. The reaction mixture was stirred for 30 min at room temperature, and then bis(pinacolato)diboron (1.1 equiv) in THF (1 mL) was added. The reaction mixture was stirred for 30 min and then a,ß-unsaturated ester (1 equiv) in THF (1 mL) was added, followed by MeOH (2 equiv). The reaction tube was washed with THF (5 mL), sealed, and stirred until no starting material was detected by TLC. The reaction

mixture was filtered through a pad of celite and concentrated. The product was purified by silica gel chromatography (eluent: petroleum ether: EtOAc =30:1, v/v).

General process 1B2[2b]: To a mixture of CuBr (0.86 mg, 2.0 mol%, 6.0 μmol), phen (2.0 mol%,), K_2CO_3 (4.1 mg, 10 mol%, 30 μmol) and B_2pin_2 (152 mg, 0.60 mmol) were added DMSO (0.6 mL) and then ynoates (0.30 mmol) and methanol (36 μL, 0.90 mmol) in a pressure vial. After stirring at 30 °C for 20 h, the mixture was filtered through a short plug of silica gel using ethyl acetate as an eluent.

General process 1C[3]: β-boryl esters was dissolved in 2 ml THF and hydrolyzed with 4 equiv of LiOH dissolved in 2 ml H_2O at room temperature for 1 h. The reaction mixture was diluted with water (100 mL). The aqueous phase was washed with EA (3 x 50 mL) and was acidified with conc. aq. HCl until pH 1. The aqueous phase was extracted with EA (3 x 50 mL).The combined organic phases mixture was washed with water, brine, dried with Na_2SO_4, filtered and evaporated under reduced pressure, β-boryl acid was obtained without further purification.

General process 1D[4]: The corresponding alkyl β-boryl acid (1.2 equiv.), *N*-hydroxyphthalimide (1.0 equiv.), and 4-dimethylaminopyridine (5 mol %) were mixed in a flask with a magnetic stirring bar. dry THF or CH_2Cl_2 (40 mL) (according to the solubility of the carboxylic acids) was added. Then a solution of *N, N*-dicyclohexylcarbodiimide (1.2 equiv.) in THF or CH_2Cl_2 (15 mL) was added slowly at room temperature. The reaction mixture was stirred at room temperature for 6 h. After *N*-hydroxyphthalimide was completely converted, the white precipitate was filtered off and the solution was concentrated under vacuum. Corresponding redox active esters were purified by column chromatography on silica gel (CH_2Cl_2 or petroleum ether/ethyl acetate as eluent).

General process 2: Preparation of propargyl alkynyl sulfones 2[5].

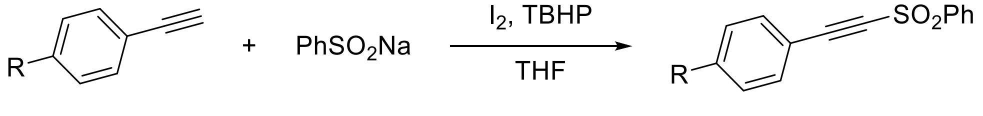

Add arylacetylene (1 equiv, 5 mmol) and tert-butyl hydroperoxide (TBHP) (3 equiv, 15 mmol) to a solution of sodium benzenesulfinate (2 equiv, 10 mmol) and iodine (I_2) (0.5 equiv, 2.5 mmol) in THF (20 mL). Stir the solution at room temperature for 8-12 hours. Monitor the reaction by TLC. Add H_2O (10 mL) to the solution to quench the reaction. Add ($Na_2S_2O_3$) solution dropwise to the

mixture until the solution changes from brown to clear and transparent. Extract the separated aqueous phase with ethyl acetate (3 × 40 mL). Dry the combined organic layers over $NaSO_4$. Concentrate the combined organic layers in vacuo. Purify the residue by column chromatography using ethyl acetate/petroleum ether as eluent (eluent: petroleum ether: EtOAc =10:1, v/v).

General process 3: Preparation of propargyl alkenyl sulfones 2[6].

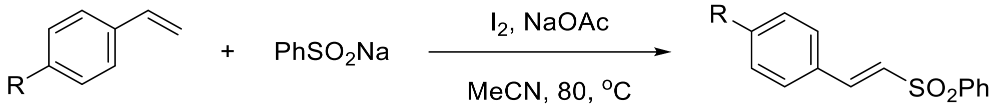

I_2 (762 mg, 3.0 mmol) was added to a suspension mixture of styrene derivative (2.0 mmol), sulfonic acid sodium (6.0 mmol) and NaOAc (246 mg, 3.0 mmol) in MeCN (10 mL), and the reaction mixture was vigorously stirred at refluxing temperature for 2 hours. Upon completion of the reaction, the reaction mixture was quenched by the addition of saturated aqueous sodium thiosulfate ($Na_2S_2O_3$) (5 mL), and basified with saturated aqueous sodium hydrogen carbonate ($NaHCO_3$) (5 mL). Further stirring was followed by extraction with ethyl acetate (3×15 mL). The combined organic extracts were washed with water (20 mL), brine (20 mL), dried over anhydrous Na_2SO_4, filtered, and concentrated in vacuo. The residue was purified by column chromatography (silica gel) to furnish the product (silica gel, petroleum ether: EtOAc =10:1, v/v).

General process 4: Preparation of propargyl alkenyl sulfones 4[7].

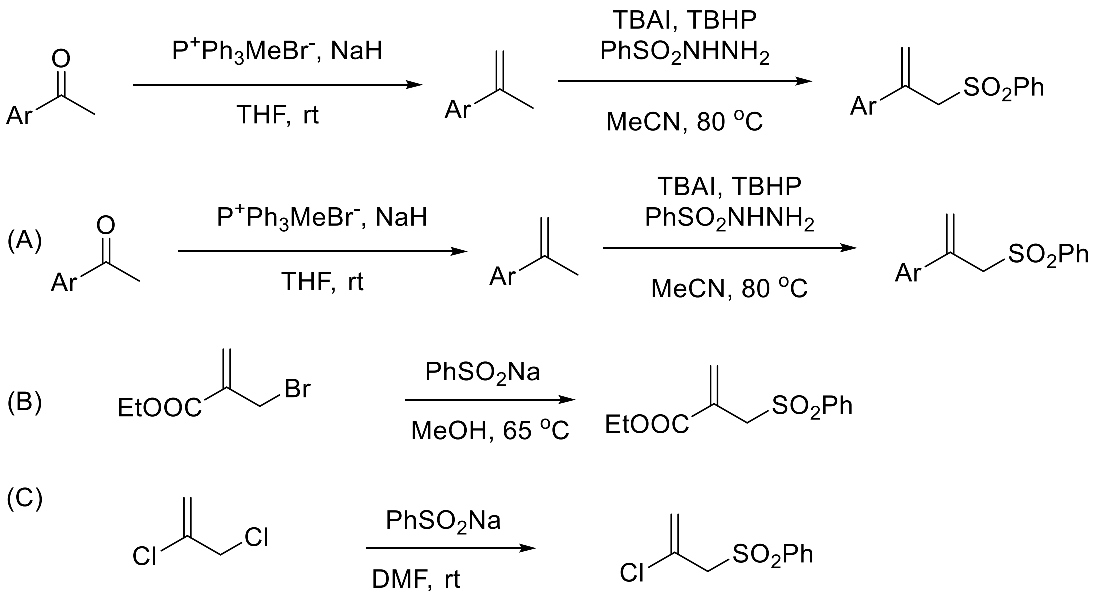

General process 4A: To a solution of $P^+Ph_3MeBr^-$(11 mmol, 1.1 equiv) in THF (20 mL) was added

NaH (60%, 1.2 equiv.), the reaction mixture was reacted for 2 h at room temperature. Then the corresponding ketones (10 mmol) in THF (10 mL) were added dropwise at 0 ℃. The mixture was reacted overnight at room temperature. When the starting material was consumed (monitored by TLC), the reaction mixture was diluted by petroleum ether and filtered through a pad of silica gel. The filtrate was concentrated to give a crude product which was distilled or purified through flash column chromatography to obtain the desired product. The known products were identical to the literature.

Alkenes (5 mmol) and TBHP (10 mmol) was added to a solution of sulfonylhydrazides (6 mmol) and tetrabutylammonium iodide (1 mmol) in 20 mL of MeCN. After the reaction mixture was stirred at 80 ^oC in an oil bath for 20 h, the solvent was removed under reduced pressure, then add saturated aqueous $Na_2S_2O_3$ (20 mL). Further stirring was followed by extraction with EtOAc (2 × 30 mL). The combined organic extracts were washed with H_2O (20 mL) and brine (20 mL), dried with Na_2SO_4, filtered and the filtrate was evaporated and purified by flash chromatography afforded the corresponding product.

General process 4B: To a solution of alkyl bromide (10 mmol) in dry methanol was added sodium phenylsulfinate (15 mmol). After 2.5 h of reflux in an oil bath, the mixture was concentrated under reduced pressure, the obtained residue was dissolved in EtOAc and the mixture was washed with water, brine, dried with Na_2SO_4, filtered and the filtrate was evaporated and purified by flash chromatography afforded product.

General process 4C: To a stirred solution containing 1.64 g (10 mmol) of benzenesulfinic acid sodium salt in 8 mL of DMF was slowly added 0.98 mL (10 mmol) of 3-chloro-2- chlorolpropene at room temperature. After being stirred for 5 h at the same temperature, the reaction mixture was diluted with 25 mL of saturated aqueous NH_4Cl and extracted with EtOAc. The mixture was washed with water, brine, dried with Na_2SO_4, filtered and evaporated under reduced pressure. ((2-chloroallyl)sulfonyl)benzene (1.92 g, 98%) was obtained without further purification.

General process 5: For synthesis of 3

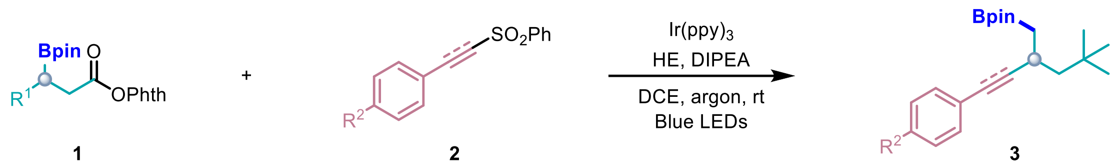

A mixture of **1** (0.2 mmol), **2** (0.3 mmol), Ir(ppy)$_3$ (2% mmol) and HE (0.3 mmol) were charged into a Schleck tube, then the air was removed, argon was filled of Schleck tube and DIPEA (0.2 mmol), DCE (2 mL) is added the mixture. The mixture was stirred under irradiation from 40W Blue LEDs. After the solvent was removed under reduced pressure, the residue was purified by silica gel chromatography using PE/EA (50:1) to afford the corresponding product.

General process 6: For synthesis of 5

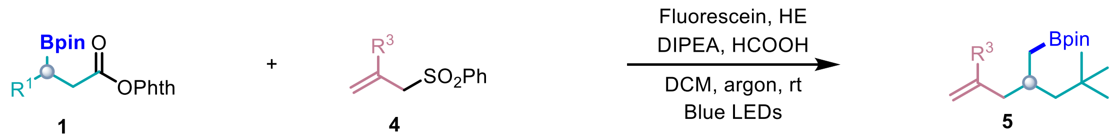

A mixture of **1** (0.2 mmol), Fluorescien (2% mmol) and HE (0.4 mmol) were charged into a Schleck tube, then the air was removed, argon was filled of Schleck tube and **4** (0.3 mmol), DIPEA (0. 4 mmol), HCOOH (0.2 mmol), DCM (2 mL) is added the mixture. The mixture was stirred under irradiation from 40W Blue LEDs. After the solvent was removed under reduced pressure, the residue was purified by silica gel chromatography using PE/EA (50:1) to afford the corresponding product.

General process 7: For synthesis of 7

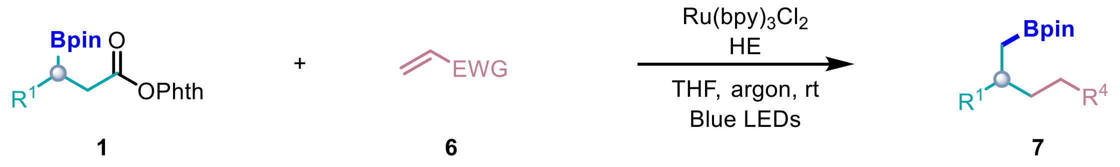

A mixture of **1** (0.2 mmol), Ru(bpy)$_3$Cl$_2$ (2% mmol) and HE (0.3 mmol) were charged into a Schleck tube, then the air was removed, argon was filled of Schleck tube and **6** (0.3 mmol), THF (2 mL) is added the mixture. The mixture was stirred under irradiation from 40W Blue LEDs. After the solvent was removed under reduced pressure, the residue was purified by silica gel chromatography using PE/EA (30:1) to afford the corresponding product.

General process 8: For synthesis of 8

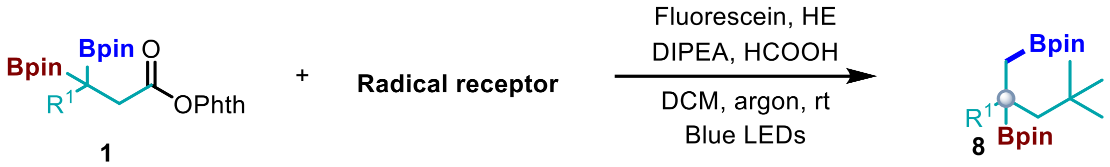

1) A mixture of **1** (0.2 mmol), Fluorescien (2% mmol) and HE (0.4 mmol) were charged into a Schleck tube, then the air was removed, argon was filled of Schleck tube and **4** (0.3 mmol), DIPEA (0. 4 mmol), HCOOH (0.2 mmol), DCM (2 mL) is added the mixturee. The mixture was stirred under irradiation from 40W Blue LEDs. After the solvent was removed under reduced pressure, the residue was purified by silica gel chromatography using PE/EA (20:1) to afford the corresponding product.

2) A mixture of **1** (0.2 mmol), **2** (0.3 mmol), Ir(ppy)$_3$ (2% mmol) and HE (0.3 mmol) were charged into a Schleck tube, then the air was removed, argon was filled of Schleck tube and DIPEA (0.2 mmol), DCE (2 mL) is added the mixture. The mixture was stirred under irradiation from 40W Blue LEDs. The crude material was dissolved in THF (2 mL, 0.1 M) and cooled to 0 °C. An aqueous solution of sodium hydroxide (3 M, 5 mL) and aqueous hydrogen peroxide (30%, 2.5 mL) were subsequently added dropwise. After 10 min, the vigorously stirred biphasic reaction mixture was allowed to reach ambient temperature and stirred for another 2 h, at which point TLC analysis showed the disappearance of the boronic ester. The mixture was diluted with water (5 mL) and ethyl acetate (10 mL) and the phases were separated. The aqueous phase was washed twice with ethyl acetate (10 mL each), and the organic phases were combined and subsequently washed with brine (15 mL). The washed organic solution was then dried over anhydrous sodium sulfate, the dried solution was filtered and the filtrate was concentrated under reduced pressure. The resulting crude material was purified by flash column chromatography on silica gel to afford analytically pure productsthe residue was purified by silica gel chromatography using PE/EA (5:1) to afford the corresponding product.

General process 9: Gram-scale synthesis of 3a

A mixture of **1a** (3 mmol), **2a** (4.5 mmol), Ir(ppy)$_3$ (5% mmol) and HE (4.5 mmol) were charged

into a Schleck tube, then the air was removed, argon was filled of Schleck tube and DIPEA (6 mmol), DCE (30 mL) is added the mixture. The mixture was stirred under irradiation from 40W Blue LEDs. After the solvent was removed under reduced pressure, the residue was purified by silica gel chromatography using PE/EA (50:1) to afford the corresponding product **3a** (67%).

General process 10: Gram-scale synthesis of 5a

A mixture of **1a** (2 mmol), Fluorescien (5% mmol) and HE (4 mmol) were charged into a Schleck tube, then the air was removed, argon was filled of Schleck tube and **4a** (3 mmol), DIPEA (4 mmol), HCOOH (2 mmol), DCM (20 mL) is added the mixture. The mixture was stirred under irradiation from 40W Blue LEDs. After the solvent was removed under reduced pressure, the residue was purified by silica gel chromatography using PE/EA (50:1) to afford the corresponding product **5a** (71%).

General process 11: Gram-scale synthesis of 7a

A mixture of **1a** (2 mmol), $Ru(bpy)_3Cl_2$ (5% mmol) and HE (3 mmol) were charged into a Schleck tube, then the air was removed, argon was filled of Schleck tube and **6a** (3 mmol), THF (20 mL) is added the mixture. The mixture was stirred under irradiation from 40W Blue LEDs. After the solvent was removed under reduced pressure, the residue was purified by silica gel chromatography using PE/EA (30:1) to afford the corresponding product **7a** (57%).

General process 12: For synthesis of 9[8].

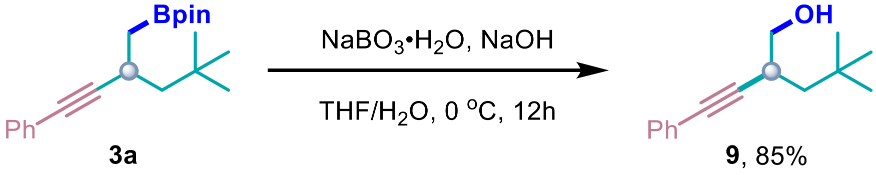

To a 25 mL Schlenk tube, was added **3a** (1 equiv, 0.2 mmol) followed by 0.3 mmol of sodium perborate monohydrate (1.5 equiv) under air. Then THF (0.8 mL) and H_2O (0.8 mL) was added in ice-bath, with that the NaOH (aq.) was added by drop wise. The reaction mixture was stirred at 0-rt until full conversion by TLC. Then, the reaction mixture was extracted with EA/H_2O.

Subsequently, the organic layers were dried with Na_2SO_4, and concentrated to dryness. The crude product was purified by silica gel chromatography (silica gel, PE: EA =100:1, v/v) to afford the product with the yield of 85%.

General process 13: For synthesis of 10[9].

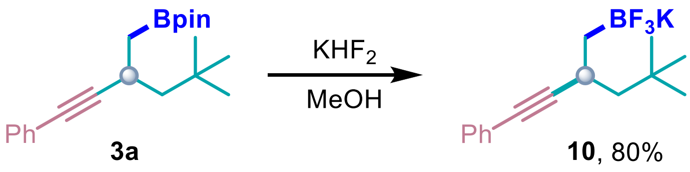

In air, to a 25 mL schlenk tube equipped with a magnetic stir bar were added the **3a** (0.2 mmol, 1 equiv) and 5 mL MeOH. To this solution sat. KHF_2 (2.4 mmol, 12 equiv, in water) was added dropwise under room temperature, and the mixture was allowed to stir at ambient temperature for 3 h. The resultant suspension was concentrated under reduced pressure. Then hot acetone (2 mL × 3) was added and filtered. The filtrate was concentrated to near dryness and Et_2O (5 mL) was added to yield a white precipitate. The precipitate was isolated by filtration, washed by CH_2Cl_2 (0.5 mL), to afford the product (80% yield) as a white solid.

General process 14: For synthesis of 11[10].

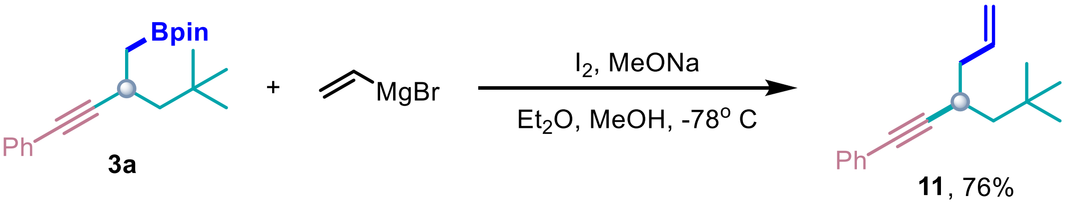

An oven-dried 10 mL Schlenk tube equipped with a Teflon stir bar was added with a solution of **3a** (0.10 mmol) and hydrous THF (1.0 mL) under argon atmosphere via syringe. Then a solution of vinyl magnesium bromide (0.4 mmol, 0.8 mL, 0.5 M in THF) was added dropwise, and the mixture was stirred for 30 min at room temperature. Then the tube was cooled to -78 °C and a solution of I_2 (0.4 mmol in 1.0 mL MeOH) was added dropwise. The solution was stirred for another 30 min at this temperature, followed by addition of a solution of NaOMe (0.8 mmol in 1.0 mL MeOH). The reaction mixture was allowed to warm to room temperature and stirred for another 2 hours. Once

finished, the reaction was quenched with $Na_2S_2O_3$(saturated aqueous solution, 3.0 mL). The aqueous solution was extracted with Et_2O (3 × 5.0 mL). The combined organic layers were dried over anhydrous Na_2SO_4, filtered and concentrated, the crude product was purified carefully by column chromatography on silica gel affording **10** (76% yield, colorless oil).

General process 15: For synthesis of 12 and 13[11].

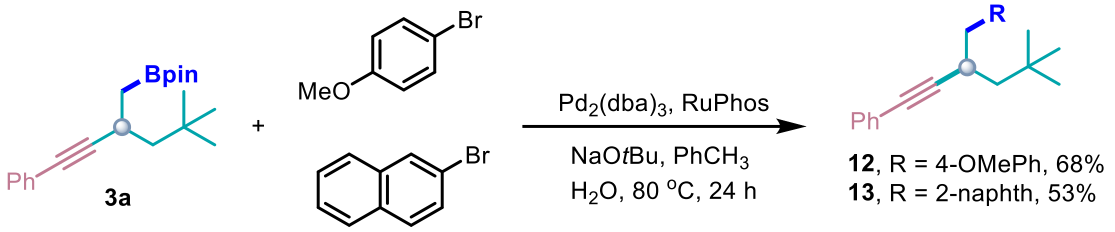

In air, $Pd_2(dba)_3$ (4.6 mg, 0.005 mmol, 5.0 mol%), NaO*t*Bu (28.8 mg, 0.3 mmol, 3.0 equiv), Ruphos (4.7 mg, 0.01 mmol, 10.0 mol%) and substrate **3a** (0.1 mmol, 1.0 equiv) were added to a Schlenk tube equipped with a stir bar. The vessel was evacuated and filled with argon (three cycles). Then $PhCH_3$ (1.0 mL), 4-OMePhBr (0.1 mmol, 1.0 equiv) or 2-bromonaphthalene (0.1 mmol) and H_2O (0.1 mL) was added under argon atmosphere. The mixture was stirred at 80 °C for 24 hours. Then, the mixture was cooled to room temperature and diluted with DCM (20 mL). The organic layer was separated and the aqueous phase was extracted with DCM (20 mL x 2). The combined organic layer was washed with H_2O (30 mL) and brine (30 mL). The combined organic layers were dried over Na_2SO_4 and concentrated in vacuo. The crude product was purified by silica gel chromatography (silica gel, PE: EA =50:1, v/v) to afford the product with the yield of 68% and 53%.

General process 16: For synthesis of 14[12].

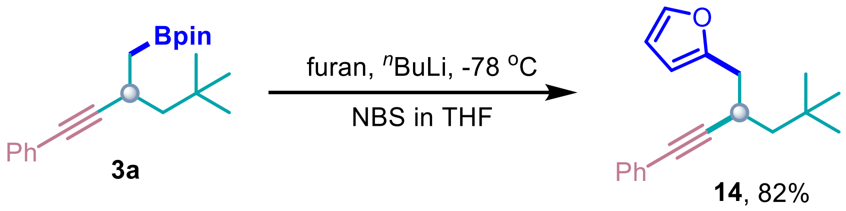

A solution of furan (0.5 mmol, 2.5 equiv) in THF (2.0 mL) was cooled to -78 °C and treated with ^{n}BuLi (1.6 M in hexanes, 0.6 mmol, 3 equiv). The cooling bath was removed and the mixture was stirred at room temperature for 1 h. The mixture was cooled to -78 °C and a solution of **3a** (0.2

mmol, 1.0 equiv) in THF (1 mL) was added. The mixture was stirred at -78 °C for 1 h, and then a solution of NBS (0.5 mmol, 2.5 equiv) in THF (2.0 mL) was added. After 10 h at room temperature, sat. $Na_2S_2O_3$ (aq.) (4 mL) was added. The reaction mixture was diluted with water and extracted with ethyl acetate. The combined organic layers were dried over Na_2SO_4 and concentrated in vacuo. The crude product was purified by silica gel chromatography (silica gel, PE: EA =50:1, v/v) to afford the product with the yield of 82%.

General process 17: For synthesis of 15[12].

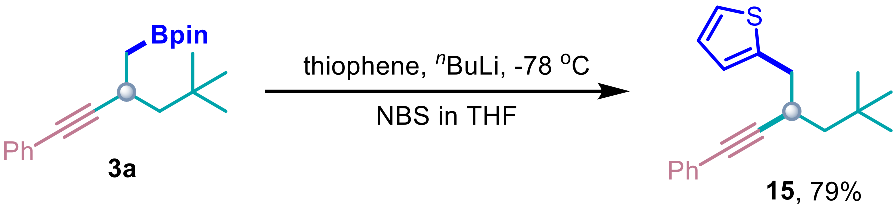

A solution of thiophene (1 mmol, 5.0 equiv) in THF (2.0 mL) was cooled to -78 °C and treated with ^{n}BuLi (1.6 M in hexanes, 1.2 mmol, 6.0 equiv). The cooling bath was removed and the mixture was stirred at room temperature for 1 h. The mixture was cooled to -78 °C and a solution of substrate **3a** (0.2 mmol, 1.0 equiv) in THF (1 mL) was added. The mixture was stirred at -78 °C for 1 h, and then a solution of NBS (1 mmol, 5.0 equiv) in THF (2.0 mL) was added. After 10 h at room temperature, sat. $Na_2S_2O_3$ (aq.) (4 mL) was added. The reaction mixture was diluted with water and extracted with ethyl acetate. The combined organic layers were dried over Na_2SO_4 and concentrated in vacuo. The crude product was purified by silica gel chromatography (silica gel, PE: EA =50:1, v/v) to afford the product with the yield of 79%.

General process 18: For synthesis of 16[13].

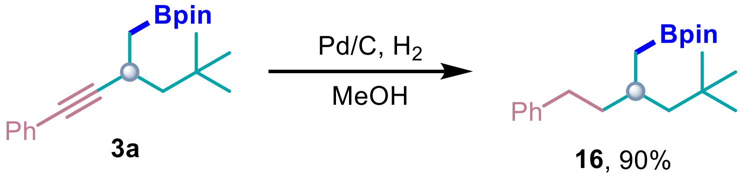

To a Schlenk tube containing of the **3a** (0.2 mmol) and 2 mL of MeOH, Pd/C (10% w) was amed. The tube was evacuated and refilled with H_2 with three times. The reaction was refilled with

hydrogen through a balloon. The resulting reaction mixture was stirred under the hydrogen atmosphere at room temperature for 12 h. After the reaction was completed, which was determined by TLC analysis. The resulting suspension was filtered through a plug of celite and the filter cake washed with DCM. The mixture was concentrated in vacuum and the residue was purified by silica gel chromatography using petroleum as the eluent to afford the **16** as a colorless oil (90%).

2. Supplementary Discussion

2.1 Optimization studies

2.1.1 The condition screening for the 3a

Table S1: Screening of photocatalysts.[a]

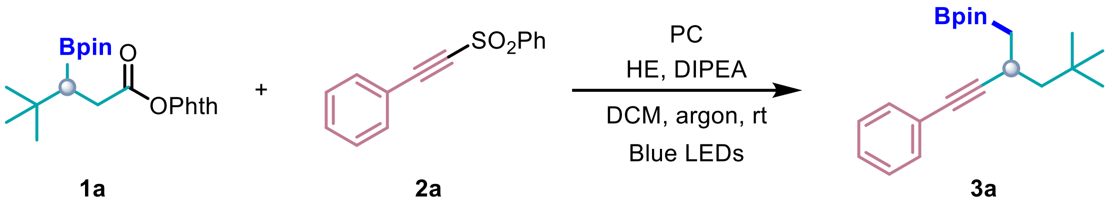

entry	PC	yield(%)[b]
1	$Ru(bpy)_2Cl_2•6H_2O$	26%
2	$Ru(bpy)_3(PF_6)_2$	24%
3	Eosin Y	36%
4	$Ir(ppy)_3$	52%
5	$Ir(dtbpy)(bpy)_2PF_6$	48%
6	$Ir(dCF_3bpy)(bpy)_2PF_6$	40%
7	Acid red 94	38%
8	4CzIPN	41%
9	4',5'-dibromofluorescein	40%
10	Rhodamine B	32%
11	Benzophenone	22%
12	Eosin B	34%
13	Fluorescein	33%
14	$Ru(bpy)_3Cl_2$	40%

[a] Reaction conditions: **1a** (0.2 mmol), **2a** (1.5 equiv, 0.3 mmol), photocatalyst (1 mol%, 0.002 mmol), HE (1.5 equiv, 0.3 mmol), DIPEA (2 equiv, 0.4 mmol), DCM (2 mL) at room temperature, 40 W blue LEDs, 12 h in argon. [b] Isolated yield.

Table S2: Screening of different solvents.[a]

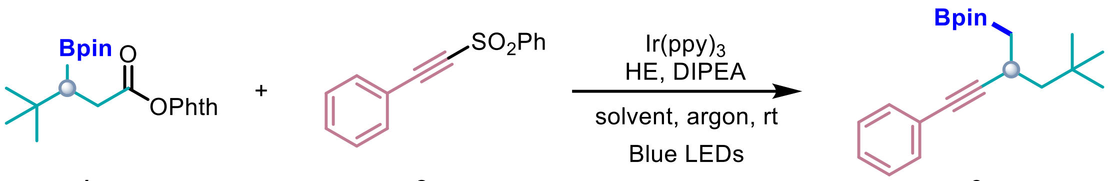

1a **2a** **3a**

entry	solvent	yield (%)[b]
1	THF	45%
2	EA	43%
3	toluene	29%
4	CH_3CN	39%
5	DCE	74%
6	DMSO	nr
7	DMA	38%
8	DMF	48%
9	dioxane	47%
10	$CHCl_3$	45%
11	MeOH	nr
12	acetone	52%

[a] Reaction conditions: **1a** (0.2 mmol), **2a** (1.5 equiv, 0.3 mmol), Ir(ppy)$_3$ (1 mol%, 0.002 mmol), HE (1.5 equiv, 0.3 mmol), DIPEA (2 equiv, 0.4 mmol), solvent (2 mL) at room temperature, 40 W blue LEDs, 12 h in argon. [b] Isolated yield.

Table S3: The effects of equivalent of 2a, HE and DIPEA.

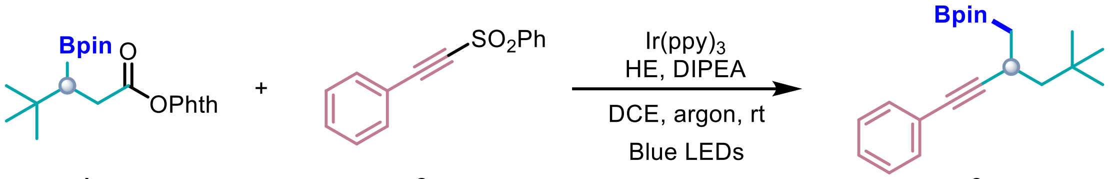

1a **2a** **3a**

entry	**2a** (x equiv)	HE (y equiv)	DIPEA (z equiv)	yield(%)[b]
1	1.5	1	2	61%
2	1.5	2	2	56%
3	1.5	2.5	2	37%
4	1.5	3	2	45%
5	1.5	1.5	1	48%
6	1.5	1.5	3	52%
7	2	1.5	2	39%

[a] Reaction conditions: **1a** (0.2 mmol), **2a** (x equiv), $Ir(ppy)_3$ (1 mol%, 0.002 mmol), HE (y equivl), DIPEA (z equiv), DCE (2 mL) at room temperature, 40 W blue LEDs, 12 h in argon. [b] Isolated yield.

Table S4: Control Experiments.[a]

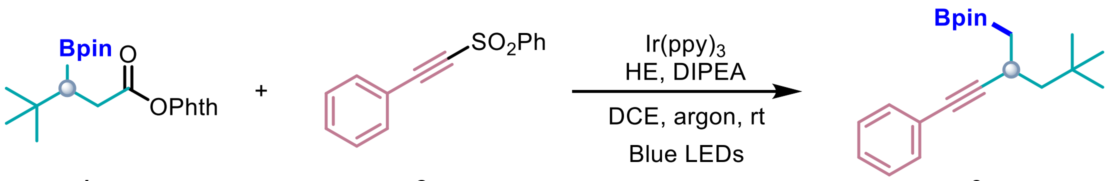

1a 2a 3a

entry	PC	HE	DIPEA	yield(%)[b]
1	-	1.5	2	trace
2	Ir(ppy)$_3$	-	2	trace
3	Ir(ppy)$_3$	1.5	-	38%
4[c]	Ir(ppy)$_3$	1.5	2	NR

[a] Reaction conditions: **1a** (0.2 mmol), **2a** (1.5 equiv, 0.3 mmol), Ir(ppy)$_3$ (1 mol%, 0.002 mmol), HE (1.5 equiv, 0.3 mmol), DIPEA (2 equiv, 0.4 mmol), DCE (2 mL) at room temperature, 40 W blue LEDs, 12 h in argon. [b] Isolated yield. [c] Without light.

2.1.2 The condition screening for the 5a

Table S5: Screening of photocatalysts.[a]

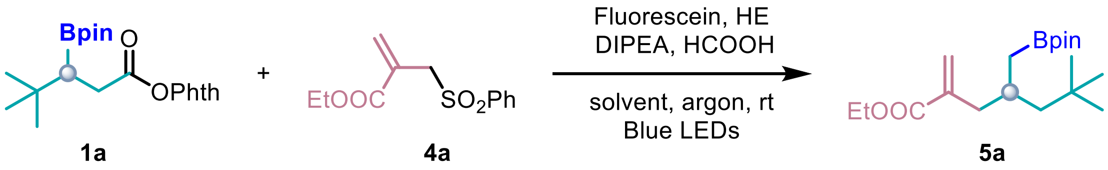

entry	**PC**	yield(%)[b]
1	$Ru(bpy)_2Cl_2•6H_2O$	45%
2	$Ru(bpy)_3(PF_6)_2$	45%
3	Eosin Y	63%
4	$Ir(ppy)_3$	65%
5	$Ir(dtbpy)(bpy)_2PF_6$	39%
6	$Ir(dCF_3bpy)(bpy)_2PF_6$	34%
7	Acid red 94	38%
8	4CzIPN	21%
9	4',5'-dibromofluorescein	31%
10	Rhodamine B	38%
11	Benzophenone	62%
12	Eosin B	70%
13	Fluorescein	83%
14	$Ru(bpy)_3Cl_2$	54%

[a] Reaction conditions: **1a** (0.2 mmol), **4a** (1.5 equiv, 0.3 mmol), PC (1 mol%, 0.002 mmol), HE (2 equiv, 0.4 mmol), DIPEA (1 equiv, 0.2 mmol), HCOOH (1 equiv, 0.2 mmol), DCM (2 mL) at room temperature, 40 W blue LEDs, 12 h in argon. [b] Isolated yield.

Table S6: Screening of different solvents.[a]

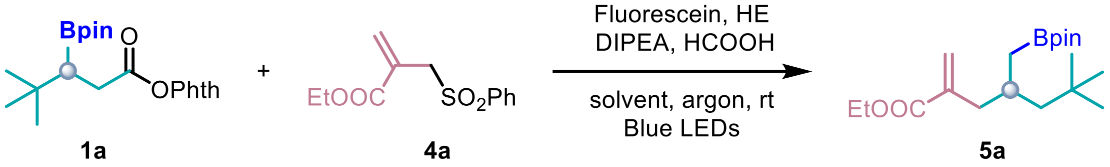

entry	solvent	yield(%)[b]
1	THF	58%
2	toluene	31%
3	EA	33%
4	acetone	49%
5	$CHCl_3$	44%
6	DCE	65%
7	DMF	38%
8	DMA	48%
9	DMSO	30%
10	dioxane	60%
11	DME	30%
12	CH_3CN	70%

[a] Reaction conditions: **1a** (0.2 mmol), **4a** (1.5 equiv, 0.3 mmol), Fluoresein (1 mol%, 0.002 mmol), HE (2 equiv, 0.4 mmol), DIPEA (1 equiv, 0.2 mmol), HCOOH (1 equiv, 0.2 mmol), solvent (2 mL) at room temperature, 40 W blue LEDs, 12 h in argon. [b] Isolated yield.

Table S7: The effects of equivalent of 4a, HE and DIPEA.

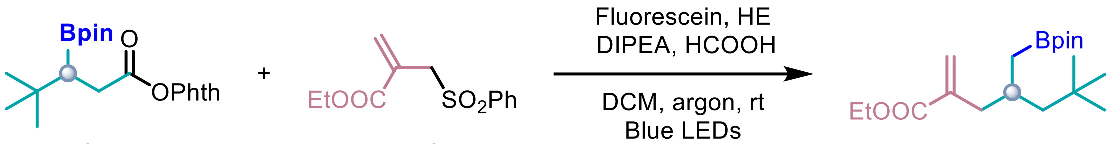

entry	**4a** (x equiv)	HE (y equiv)	DIPEA (z equiv)	yield(%)[b]
1	1.5	1	1	60%
2	1.5	1.5	1	68%
3	1.5	3	1	56%
4	2	2	1	70%
5	1.5	2	2	59%
6	1.5	2	3	54%

[a] Reaction conditions: **1a** (0.2 mmol), **4a** (x equiv), Fluoresein (1 mol%, 0.002 mmol), HE (y equiv), DIPEA (z equiv), HCOOH (1 equiv, 0.2 mmol), DCM (2 mL) at room temperature, 40 W blue LEDs, 12 h in argon. [b] Isolated yield.

Table S8: Control Experiments.[a]

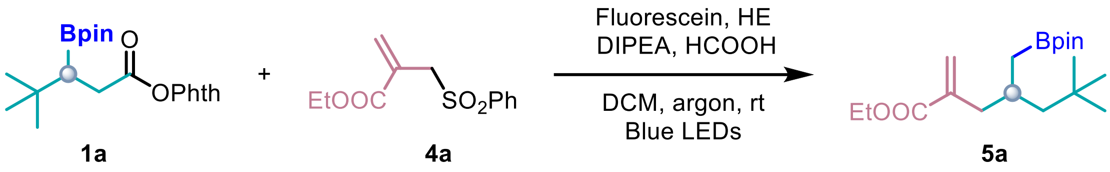

entry	PC	HE	DIPEA	HCOOH	yield(%)[b]
1	-	2	1	1	trace
2	Fluorescein	-	1	1	trace
3	Fluorescein	2	-	1	38%
4	Fluorescein	2	1	-	52%
5[c]	Fluorescein	2	1	1	NR

[a] Reaction conditions: **1a** (0.2 mmol), **4a** (1.5 equiv, 0.3 mmol), Fluoresein (1 mol%, 0.002 mmol), HE (2 equiv, 0.4 mmol), DIPEA (1 equiv, 0.2 mmol), HCOOH (1 equiv, 0.2 mmol), DCM (2 mL) at room temperature, 40 W blue LEDs, 12 h in argon. [b] Isolated yield. [c] Without light.

2.1.3 The condition screening for the 7a

Table S9: Screening of photocatalysts.[a]

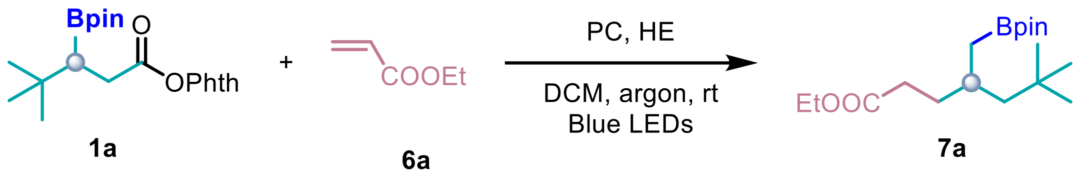

entry	**PC**	yield(%)[b]
1	$Ru(bpy)_2Cl_2$•$6H_2O$	42%
2	$Ru(bpy)_3(PF_6)_2$	45%
3	Eosin Y	43%
4	$Ir(ppy)_3$	45%
5	$Ir(dtbpy)(bpy)_2PF_6$	39%
6	$Ir(dCF_3bpy)(bpy)_2PF_6$	34%
7	Acid red 94	38%
8	4CzIPN	21%
9	Rhodamine B	31%
10	Eosin B	38%
11	Fluorescein	32%
12	$Ru(bpy)_3Cl_2$	51%

[a] Reaction conditions: **1a** (0.2 mmol), **6a** (1.5 equiv, 0.3 mmol), PC (1 mol%, 0.002 mmol), HE (1.5 equiv, 0.3 mmol), DCM (2 mL) at room temperature, 40 W blue LEDs, 12 h in argon. [b] Isolated yield.

Table S10: Screening of different solvents.[a]

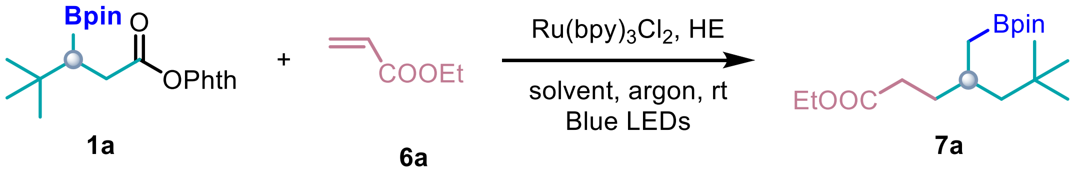

entry	solvent	yield(%)[b]
1	CH_3CN	14%
2	dioxane	trace
3	THF	71%
4	EA	51%
5	DMF	trace
6	DCE	48%
7	DMSO	NR
8	2-MeTHF	31%
9	EtOH	trace
10	MeOH	43%
11	Et_2O	25%
12	toluene	trace
13	CH_3Cl	35%
14	DME	50%

[a] Reaction conditions: **1a** (0.2 mmol), **6a** (1.5 equiv, 0.3 mmol), $Ru(bpy)_3Cl_2$ (1 mol%, 0.002 mmol), HE (1.5 equiv, 0.3 mmol), solvent (2 mL) at room temperature, 40 W blue LEDs, 12 h in argon. [b] Isolated yield.

Table S11: The effects of equivalent of 6a and HE.

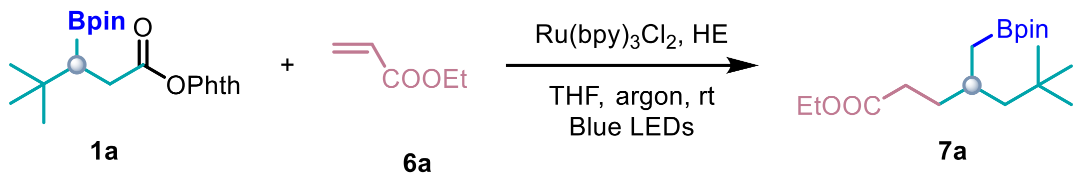

entry	**6a** (x equiv)	HE (y equiv)	yield(%)[b]
1	1.5	1	52%
2	1.5	2	57%
3	1.5	3	57%
4	2	1.5	51%
5	3	1.5	42%

[a] Reaction conditions: **1a** (0.2 mmol), **6a** (x equiv), $Ru(bpy)_3Cl_2$ (1 mol%, 0.002 mmol), HE (y equiv), THF (2 mL) at room temperature, 40 W blue LEDs, 12 h in argon. [b] Isolated yield.

Table S12: Control Experiments.[a]

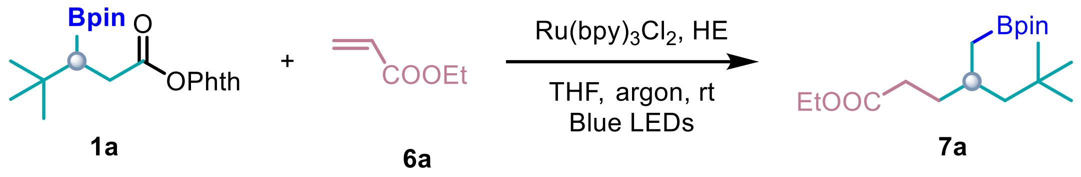

entry	**PC**	HE	yield(%)[b]
1	-	1.5	trace
2	$Ru(bpy)_3Cl_2$	-	trace
3[c]	$Ru(bpy)_3Cl_2$	1.5	NR

[a] Reaction conditions: **1a** (0.2 mmol), **6a** (1.5 equiv, 0.3 mmol), $Ru(bpy)_3Cl_2$ (1 mol%, 0.002 mmol), HE (1.5 equiv, 0.3 mmol), THF (2 mL) at room temperature, 40 W blue LEDs, 12 h in argon. [b] Isolated yield. [c] Without light.

2.2 Control experiments

Radical capture experiment with HRMS analysis

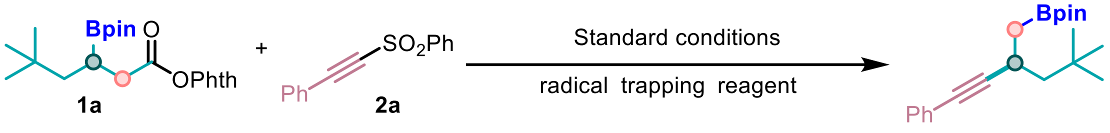

3a not detected

TMPEO (3 equiv)	BHT (3 equiv)	1,1-diphenylethylene (3 equiv)
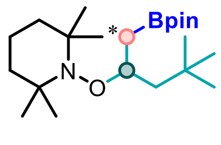	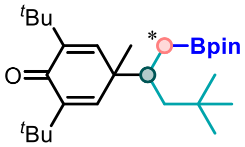	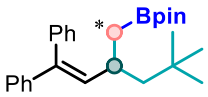
**17,** HRMS: $[M+H]^+$ Calcd: 382.3487 Found: 382.3481	**18,** HRMS: $[M+H]^+$ Calcd: 445.3848 Found: 445.3840	**19,** HRMS: $[M+H]^+$ Calcd: 405.2959 Found: 405.2950

A mixture of **1** (0.2 mmol), **2** (0.3 mmol), $Ir(ppy)_3$ (2% mmol), HE (0.3 mmol) and radical trapping reagent (3 equiv, if solid) were charged into a Schleck tube, then the air was removed, argon was filled of Schleck tube and DIPEA (0.2 mmol), radical trapping reagent (3 equiv, if liquid), DCE (2 mL) is added the mixture. The mixture was stirred under irradiation from 40W Blue LEDs. After the solvent was removed under reduced pressure, the residue was purified by silica gel chromatography using PE/EA (50:1) to afford the corresponding product.

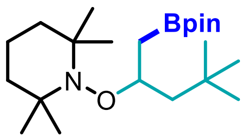

Bpin
N
O

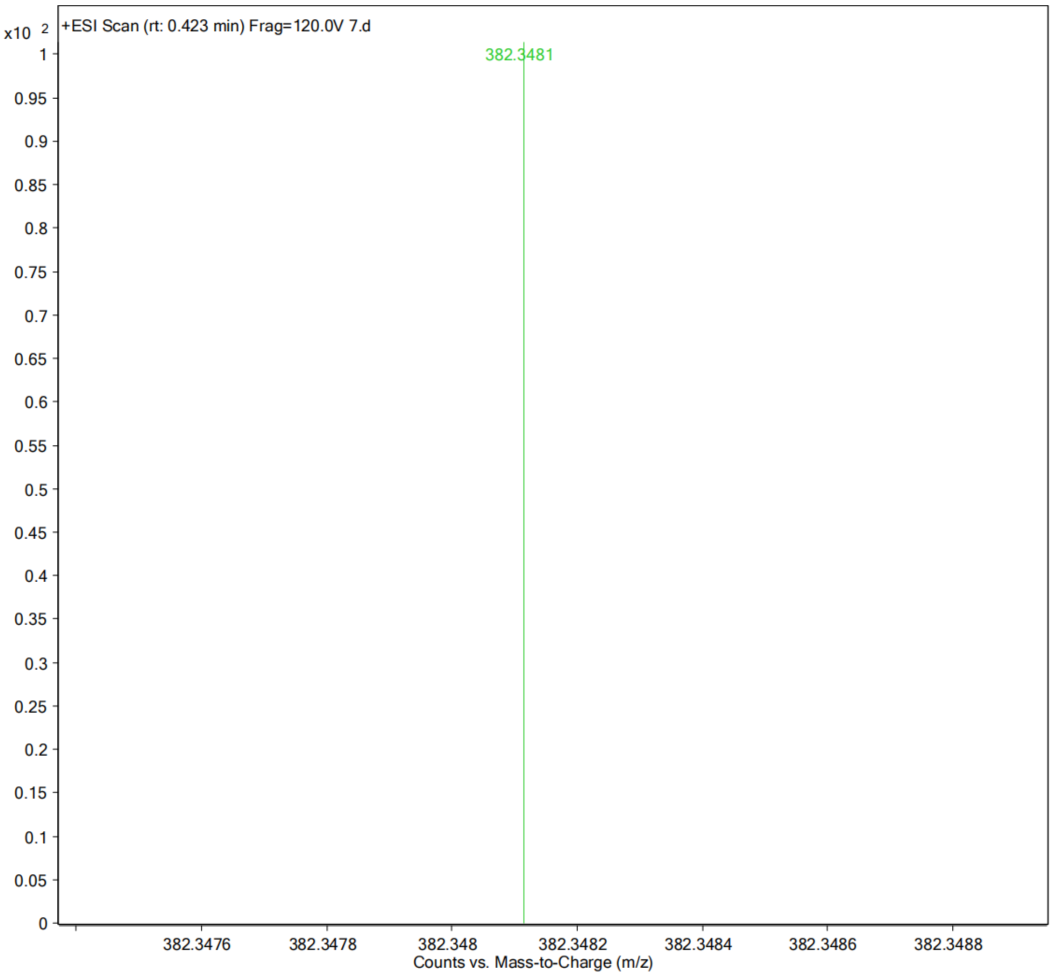

x10 2
+ESI Scan (rt: 0.423 min) Frag=120.0V 7.d
382.3481
1
0.95
0.9
0.85
0.8
0.75
0.7
0.65
0.6
0.55
0.5
0.45
0.4
0.35
0.3
0.25
0.2
0.15
0.1
0.05
0
382.3476
382.3478
382.348
382.3482
382.3484
382.3486
382.3488
Counts vs. Mass-to-Charge (m/z)

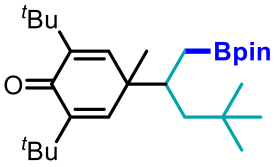

tBu
Bpin
O
tBu

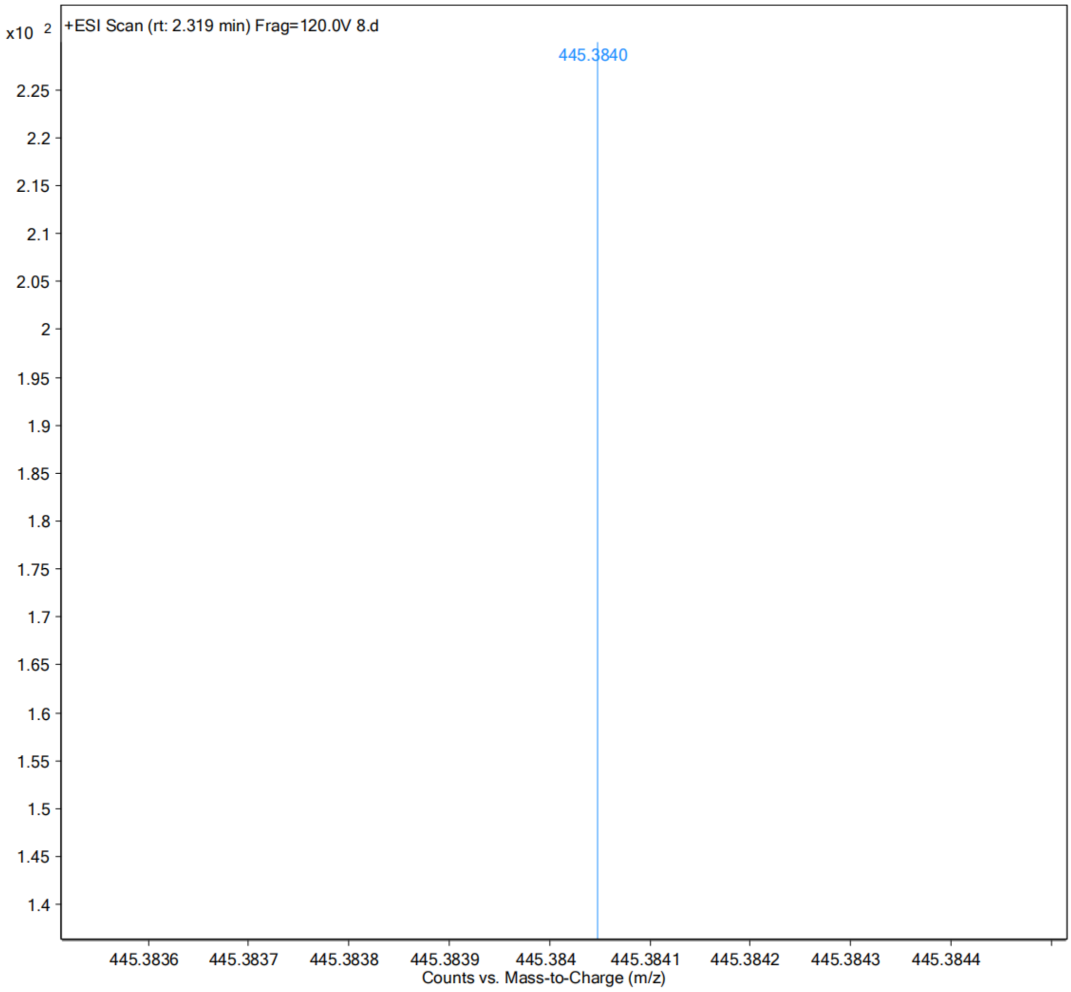

x10 2
+ESI Scan (rt: 2.319 min) Frag=120.0V 8.d
445.3840
2.25
2.2
2.15
2.1
2.05
2
1.95
1.9
1.85
1.8
1.75
1.7
1.65
1.6
1.55
1.5
1.45
1.4
445.3836
445.3837
445.3838
445.3839
445.384
445.3841
445.3842
445.3843
445.3844
Counts vs. Mass-to-Charge (m/z)

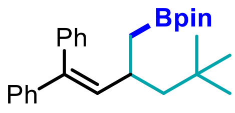
Bpin
Ph
Ph

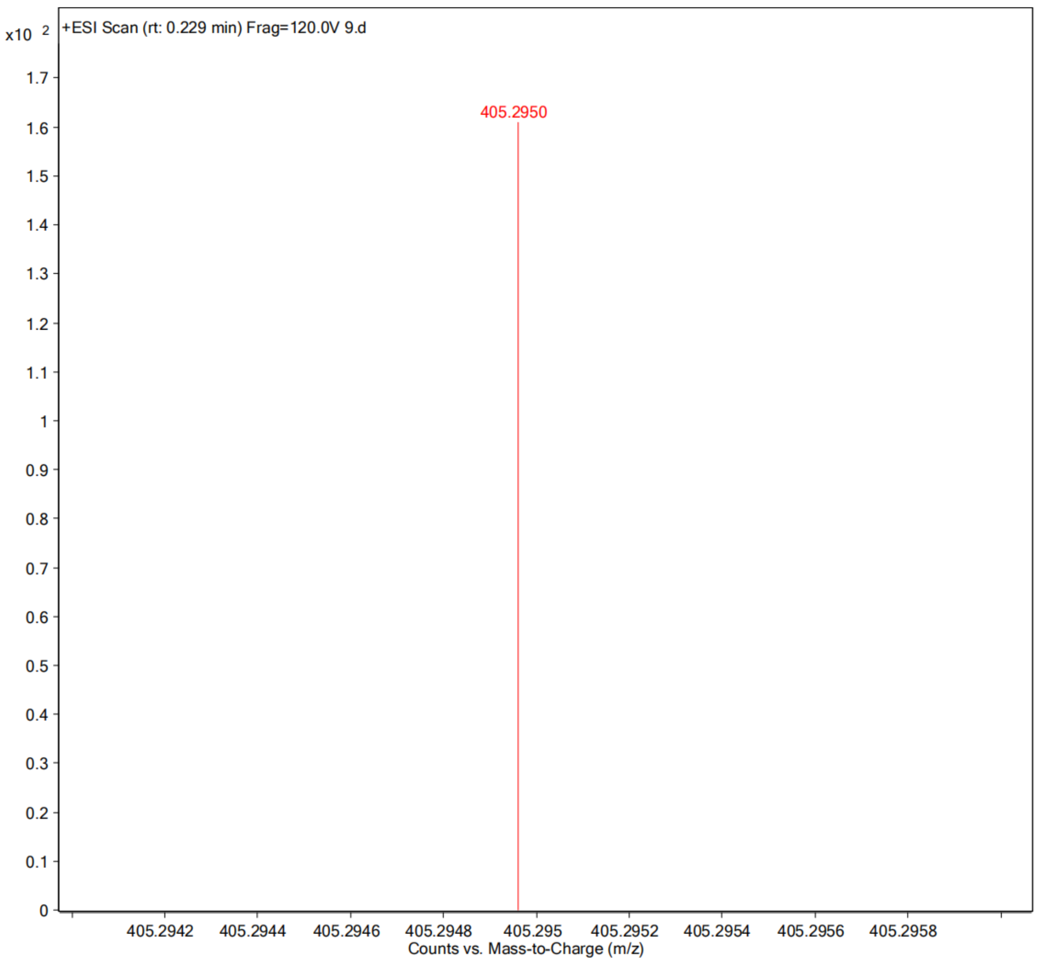
x10 2
+ESI Scan (rt: 0.229 min) Frag=120.0V 9.d
405.2950
1.7
1.6
1.5
1.4
1.3
1.2
1.1
1
0.9
0.8
0.7
0.6
0.5
0.4
0.3
0.2
0.1
0
405.2942
405.2944
405.2946
405.2948
405.295
405.2952
405.2954
405.2956
405.2958
Counts vs. Mass-to-Charge (m/z)

2.3 Crystal data

Crystallographic data for compound **1a** (CCDC 2247689) has been deposited with the Cambridge Crystallographic Data Centre, Copies of the data can be obtained, free of charge, on application to CCDC (Email: deposit@ccdc.cam.ac.uk).

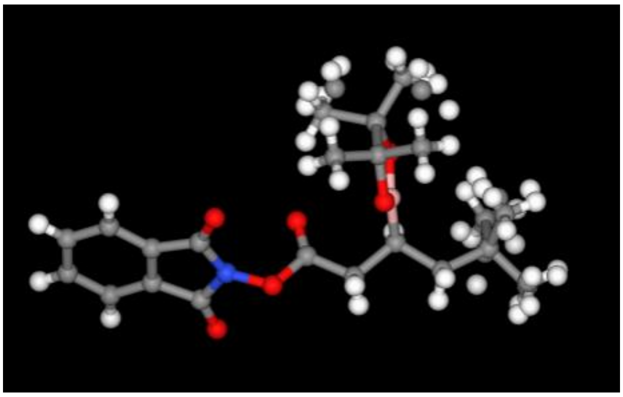

Bond precision:	C-C = 0.0034 A		Wavelength=0.71073
Cell:	a=9.751(1)	b=10.9070(11)	c=12.1691(15)
	alpha=93.921(9)	beta=110.358(10)	gamma=104.886(9)
Temperature:	293 K		

	Calculated	Reported
Volume	1154.8(2)	1154.8(2)
Space group	P -1	P -1
Hall group	-P 1	-P 1
Moiety formula	C22 H30 B N O6	C22 H30 B N O6
Sum formula	C22 H30 B N O6	C22 H30 B N O6
Mr	415.28	415.28
Dx,g cm-3	1.194	1.194
Z	2	2
Mu (mm-1)	0.085	0.085
F000	444.0	444.0
F000’	444.23	
h,k,lmax	12,13,15	12,13,15
Nref	4777	4754
Tmin,Tmax		0.563,1.000
Tmin’		

Correction method= # Reported T Limits: Tmin=0.563 Tmax=1.000
AbsCorr = MULTI-SCAN

Data completeness= 0.995 Theta(max)= 26.499

R(reflections)= 0.0663(2670) wR2(reflections)= 0.1867(4754)

S = 1.032 Npar= 318

Crystallographic data for compound **3e** (CCDC 2242317) has been deposited with the Cambridge Crystallographic Data Centre, Copies of the data can be obtained, free of charge, on application to CCDC (Email: deposit@ccdc.cam.ac.uk).

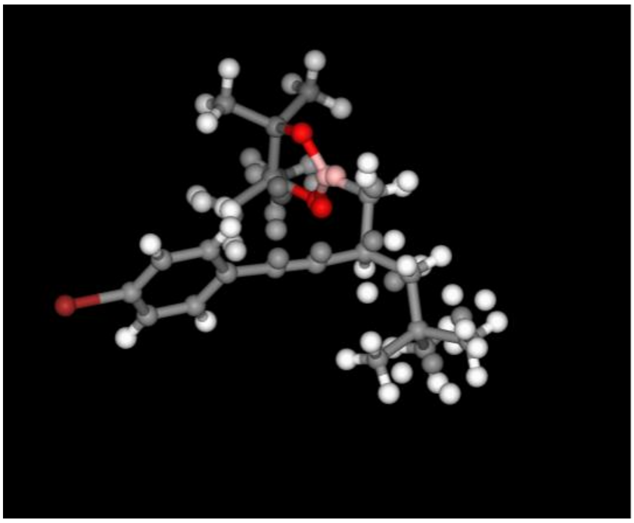

```
Bond precision:    C-C = 0.0031 A           Wavelength=1.54184

Cell:          a=10.1974(6)       b=10.2772(6)      c=11.2980(6)
               alpha=100.4422(19) beta=104.9074(18) gamma=107.0325(18)
Temperature:   100 K

                Calculated                  Reported
Volume          1050.97(11)                 1050.97(10)
Space group     P -1                        P -1
Hall group      -P 1                        -P 1
Moiety formula  C21 H30 B Br O2             C21 H30 B Br O2
Sum formula     C21 H30 B Br O2             C21 H30 B Br O2
Mr              405.16                      405.17
Dx,g cm-3       1.280                       1.280
Z               2                           2
Mu (mm-1)       2.732                       2.732
F000            424.0                       424.0
F000'           423.58
h,k,lmax        12,12,13                    12,12,13
Nref            3862                        3832
Tmin,Tmax       0.442,0.465                 0.513,0.753
Tmin'           0.334

Correction method= # Reported T Limits: Tmin=0.513 Tmax=0.753
AbsCorr = MULTI-SCAN

Data completeness= 0.992           Theta(max)= 68.327

                                                   wR2(reflections)=
R(reflections)= 0.0304( 3805)                      0.0762( 3832)

S = 1.192                  Npar= 274
```

2.4 Characterization data for Substrates

1,3-dioxoisoindolin-2-yl 5,5-dimethyl-3-(4,4,5,5-tetramethyl-1,3,2-dioxaborolan-2-yl)hexanoate (1a)

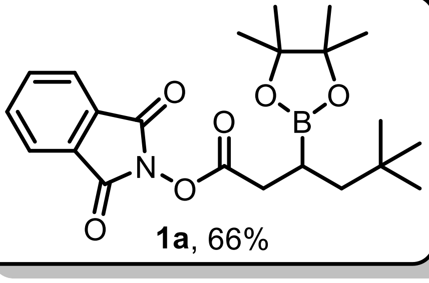

1a, 66%

Following the **general procedure 1** on 50 mmol scale, **1a** was obtained in 66% yield (13.7 g) as a white solid (m.p. : 86-88 °C), $R_f = 0.4$ (silica gel, PE: EA = 5:1, v/v), column chromatography (silica gel, PE: EA = 5:1, v/v).

^{1}H NMR (500 MHz, Chloroform-*d*) δ 7.87 (dd, *J* = 5.4, 3.1 Hz, 2H), 7.77 (dd, *J* = 5.4, 3.1 Hz, 2H), 2.78 (dd, *J* = 16.8, 7.3 Hz, 1H), 2.69 (dd, *J* = 16.9, 7.5 Hz, 1H), 1.56 – 1.48 (m, 1H), 1.23 (d, *J* = 1.9 Hz, 13H), 0.92 (s, 9H).

^{13}C NMR (126 MHz, Chloroform-*d*) δ 169.8, 161.9, 134.6, 129.0, 123.9, 83.5, 44.5, 34.3, 31.1, 29.6, 24.7.

^{11}B NMR (160 MHz, Chloroform-*d*) δ 33.58.

HRMS (ESI) m/z: $[M+H]^+$ Calcd. for: $C_{22}H_{30}BNO_6$ 416.2239; Found: 416.2236.

1,3-dioxoisoindolin-2-yl 3-cyclohexyl-3-(4,4,5,5-tetramethyl-1,3,2-dioxaborolan-2-yl)propanoate (1b)

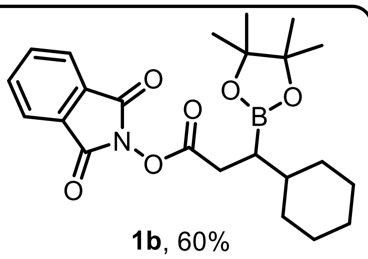

1b, 60%

Following the **general procedure 1** on 20 mmol scale, **1b** was obtained in 60% yield (5.1 g) as a white solid (m.p. : 108-110 °C), $R_f = 0.4$ (silica gel, PE: EA = 5:1, v/v), column chromatography (silica gel, PE: EA = 5:1, v/v).

^{1}H NMR (500 MHz, Chloroform-*d*) δ 7.87 (dd, *J* = 5.5, 3.1 Hz, 2H), 7.77 (dd, *J* = 5.5, 3.1 Hz, 2H), 2.85 (dd, *J* = 17.4, 10.1 Hz, 1H), 2.72 (dd, *J* = 17.4, 5.5 Hz, 1H), 1.77 – 1.69 (m, 4H), 1.68 – 1.64 (m, 1H), 1.53 – 1.40 (m, 2H), 1.24 (d, *J* = 9.0 Hz, 14H), 1.16 – 1.02 (m, 3H).

^{13}C NMR (126 MHz, Chloroform-*d*) δ 170.6, 134.6, 129.0, 123.9, 83.5, 39.1, 32.4, 31.9, 30.1, 26.7, 26.6, 26.4, 24.9, 24.6.

^{11}B NMR (160 MHz, Chloroform-*d*) δ 33.97.

HRMS (ESI) m/z: $[M+H]^+$ Calcd. for: $C_{23}H_{31}BNO_6$ 429.2239; Found: 429.2236.

1,3-dioxoisoindolin-2-yl 3-cyclopentyl-3-(4,4,5,5-tetramethyl-1,3,2-dioxaborolan-2-yl)propanoate (1c)

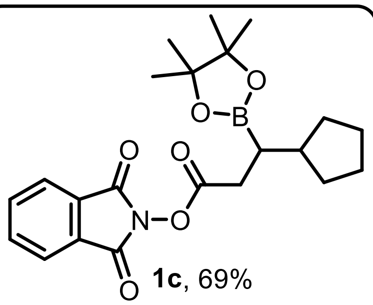

1c, 69%

Following the **general procedure 1** on 20 mmol scale, **1c** was obtained in 69% yield (5.7 g) as a white solid (m.p. : 107-109 ºC), R_f = 0.4 (silica gel, PE: EA = 5:1, v/v), column chromatography (silica gel, PE: EA = 5:1, v/v).

^{1}H NMR (500 MHz, Chloroform-*d*) δ 7.79 (dd, *J* = 5.5, 3.1 Hz, 2H), 7.70 (dd, *J* = 5.5, 3.1 Hz, 2H), 2.80 – 2.66 (m, 2H), 1.83 – 1.70 (m, 3H), 1.55 (m, 2H), 1.50 – 1.42 (m, 2H), 1.36 (m, 1H), 1.16 (d, *J* = 6.9 Hz, 14H).

^{13}C NMR (126 MHz, Chloroform-*d*) δ 170.3, 161.9, 134.6, 129.0, 123.9, 83.5, 41.1, 3.18, 31.9, 31.6, 25.3, 25.1, 24.9, 24.6.

^{11}B NMR (160 MHz, Chloroform-*d*) δ 33.89.

HRMS (ESI) m/z: $[M+H]^+$ Calcd. for: $C_{22}H_{29}BNO_6$ 414.2082; Found: 414.2079.

1,3-dioxoisoindolin-2-yl 5-phenyl-3-(4,4,5,5-tetramethyl-1,3,2-dioxaborolan-2-yl)pentanoate (1d)

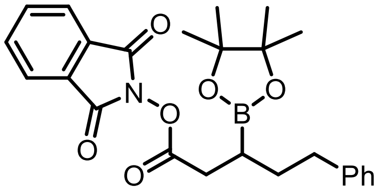

1d, 60%

Following the **general procedure 1** on 20 mmol scale, **1d** was obtained in 60% yield (5.4 g) as a white solid (m.p. : 123-125ºC), R_f = 0.4 (silica gel, PE: EA = 5:1, v/v), column chromatography (silica gel, PE: EA = 5:1, v/v).

^{1}H NMR (500 MHz, Chloroform-*d*) δ 7.87 (dd, *J* = 5.5, 3.1 Hz, 2H), 7.77 (dd, *J* = 5.5, 3.1 Hz, 2H), 7.28 (m, 2H), 7.23 – 7.15 (m, 3H), 2.88 (dd, *J* = 17.1, 8.4 Hz, 1H), 2.79 (dd, *J* = 17.2, 6.4 Hz, 1H), 2.69 (m, 2H), 1.94 – 1.73 (m, 3H), 1.61 – 1.52 (m, 1H), 1.26 (d, *J* = 4.2 Hz, 12H).

^{13}C NMR (126 MHz, Chloroform-*d*) δ 170.0, 161.9, 142.2, 134.7, 129.0, 128.5, 128.4, 125.8, 123.9, 83.7, 34.9, 32.2, 32.2, 24.8, 24.6.

^{11}B NMR (160 MHz, Chloroform-*d*) δ 34.27.

HRMS (ESI) m/z: $[M+H]^+$ Calcd. for: $C_{25}H_{29}BNO_6$ 450.2082; Found: 450.2086.

1,3-dioxoisoindolin-2-yl 3-(4,4,5,5-tetramethyl-1,3,2-dioxaborolan-2-yl)butanoate (1e)

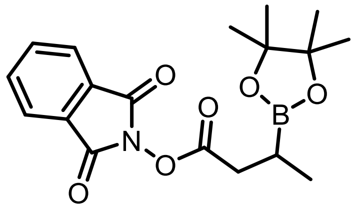

1e, 64%

Following the **general procedure 1** on 20 mmol scale, **1e** was obtained in 64% yield (4.6 g) as a white solid (m.p. : 47-49°C), $R_f = 0.4$ (silica gel, PE: EA = 5:1, v/v), column chromatography (silica gel, PE: EA = 5:1, v/v).

^{1}H NMR (500 MHz, Chloroform-*d*) δ 7.87 (dd, *J* = 5.5, 3.1 Hz, 2H), 7.77 (dd, *J* = 5.5, 3.1 Hz, 2H), 2.83 (dd, *J* = 16.9, 7.0 Hz, 1H), 2.67 (dd, *J* = 16.9, 7.4 Hz, 1H), 1.55 (m, 1H), 1.23 (d, *J* = 2.6 Hz, 11H), 1.12 (d, *J* = 7.5 Hz, 3H).

^{13}C NMR (126 MHz, Chloroform-*d*) δ 170.0, 161.9, 134.6, 129.0, 123.9, 83.6, 34.1, 24.7, 24.6, 14.8.

^{11}B NMR (160 MHz, Chloroform-*d*) δ 34.20.

HRMS (ESI) m/z: $[M+H]^+$ Calcd. for: $C_{18}H_{23}BNO_6$ 360.1613; Found: 360.1609.

1,3-dioxoisoindolin-2-yl 4-methyl-3-(4,4,5,5-tetramethyl-1,3,2-dioxaborolan-2-yl)heptanoate (1f)

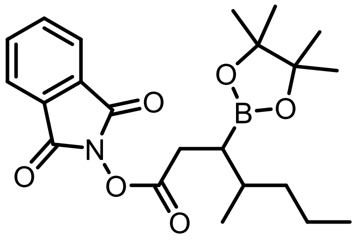

1f, 65%

Following the **general procedure 1** on 20 mmol scale, **1f** was obtained in 65% yield (5.4 g) as a white solid (m.p. : 70-72 °C), $R_f = 0.4$ (silica gel, PE: EA = 5:1, v/v), column chromatography (silica gel, PE: EA = 5:1, v/v).

^{1}H NMR (500 MHz, Chloroform-*d*) δ 7.86 (dd, *J* = 5.5, 3.1 Hz, 2H), 7.76 (dd, *J* = 5.5, 3.0 Hz, 2H), 2.85 (m, 1H), 2.66 (m, 1H), 1.78 – 1.65 (m, 1H), 1.60 – 1.51 (m, 1H), 1.37 (m, 2H), 1.30 – 1.17 (m, 14H), 0.94 (dd, *J* = 6.8, 3.4 Hz, 3H), 0.92 – 0.86 (m, 3H).

^{13}C NMR (126 MHz, Chloroform-*d*) δ 170.7, 170.5, 161.9, 134.6, 129.0, 123.9, 83.6, 83.5, 38.5, 37.6, 33.9, 33.4, 30.5, 29.1, 25.0, 24.9, 24.6, 24.5, 20.6, 20.5, 18.6, 18.2, 14.3, 14.2.

^{11}B NMR (160 MHz, Chloroform-*d*) δ 33.98.

HRMS (ESI) m/z: $[M+H]^+$ Calcd. for: $C_{22}H_{30}BNO_6$ 416.2239; Found: 416.2233.

1,3-dioxoisoindolin-2-yl 5-methyl-3-(4,4,5,5-tetramethyl-1,3,2-dioxaborolan-2-yl)hexanoate (1g)

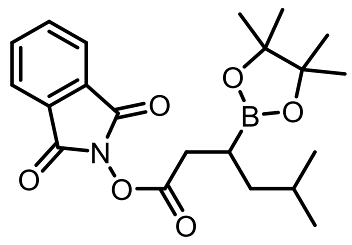

1g, 67%

Following the **general procedure 1** on 20 mmol scale, **1g** was obtained in 67% yield (5.4 g) as a white solid (m.p. : 68-70 °C), R_f = 0.4 (silica gel, PE: EA = 5:1, v/v), column chromatography (silica gel, PE: EA = 5:1, v/v).

^{1}H NMR (500 MHz, Chloroform-*d*) δ 7.87 (dd, *J* = 5.4, 3.1 Hz, 2H), 7.77 (dd, *J* = 5.5, 3.1 Hz, 2H), 2.83 – 2.67 (m, 2H), 1.59 – 1.53 (m, 1H), 1.46 – 1.41 (m, 1H), 1.34 – 1.28 (m, 2H), 1.23 (d, *J* = 5.2 Hz, 14H), 0.91 (dd, *J* = 11.9, 6.6 Hz, 6H).

^{13}C NMR (126 MHz, Chloroform-*d*) δ 170.1, 161.9, 134.6, 129.0, 123.9, 83.5, 39.3, 32.3, 26.6, 24.8, 24.6, 22.8, 22.7, 22.6.

^{11}B NMR (160 MHz, Chloroform-*d*) δ 34.38.

HRMS (ESI) m/z: $[M+H]^+$ Calcd. for: $C_{21}H_{29}BNO_6$ 402.2082; Found: 402.2080.

1,3-dioxoisoindolin-2-yl 5,10-dimethyl-3-(4,4,5,5-tetramethyl-1,3,2-dioxaborolan-2-yl)undec-9-enoate (1h)

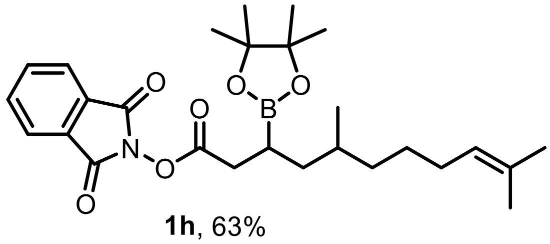

1h, 63%

Following the **general procedure 1** on 20 mmol scale, **1h** was obtained in 63%% yield (6.1 g) as a white solid (m.p. : 70-71 °C), R_f = 0.4 (silica gel, PE: EA = 5:1, v/v), column chromatography (silica gel, PE: EA = 5:1, v/v).

^{1}H NMR (500 MHz, Chloroform-*d*) δ 7.86 (dd, *J* = 5.5, 3.1 Hz, 2H), 7.76 (dd, *J* = 5.5, 3.1 Hz, 2H), 5.14 – 5.03 (m, 1H), 2.84 – 2.62 (m, 2H), 1.97 (m, 2H), 1.66 (s, 3H), 1.59 (s, 4H), 1.55 – 1.45 (m, 2H), 1.42 – 1.29 (m, 2H), 1.22 (d, *J* = 5.2 Hz, 12H), 1.17 – 1.10 (m, 1H), 0.89 (dd, *J* = 13.4, 6.5 Hz, 3H).

^{13}C NMR (126 MHz, Chloroform-*d*) δ 170.0, 161.9, 134.7, 131.1, 129.0, 124.9, 123.9, 83.5, 83.5, 38.0, 37.4, 32.9, 31.3, 25.7, 25.5, 24.8, 24.6, 24.6, 19.5, 17.7.

^{11}B NMR (160 MHz, Chloroform-*d*) δ 34.28.

HRMS (ESI) m/z: $[M+H]^+$ Calcd. for: $C_{27}H_{39}BNO_6$ 484.2865; Found: 484.2858.

1,3-dioxoisoindolin-2-yl 5-(4-isopropylphenyl)-4-methyl-3-(4,4,5,5-tetramethyl-1,3,2-dioxaborolan-2-yl)pentanoate (1i)

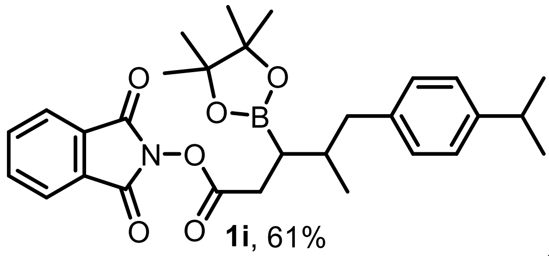

1i, 61%

Following the **general procedure 1** on 20 mmol scale, **1i** was obtained in 61% yield (6.2 g) as a white solid (mp: 81-83 °C), R_f = 0.4 (silica gel, PE: EA = 5:1, v/v), column chromatography (silica gel, PE: EA = 5:1, v/v).

^{1}H NMR (500 MHz, Chloroform-*d*) δ 7.88 (m, 2H), 7.77 (m, 2H), 7.16 – 7.06 (m, 4H), 3.00 – 2.84 (m, 2H), 2.83 – 2.67 (m, 2H), 2.37 (m, 1H), 2.09 – 1.94 (m, 1H), 1.64 (m, 1H), 1.32 – 1.20 (m, 18H), 0.91 (dd, *J* = 8.6, 6.8 Hz, 3H).

^{13}C NMR (126 MHz, Chloroform-*d*) δ 170.5, 170.4, 161.9, 146.3, 146.2, 138.4, 138.3, 134.7, 129.1, 129.1, 129.0, 126.3, 126.2, 123.9, 83.7, 83.7, 42.3, 41.6, 36.7, 35.9, 33.7, 30.8, 29.4, 25.1, 24.9, 24.6, 24.1, 18.1, 17.8.

^{11}B NMR (160 MHz, Chloroform-*d*) δ 34.21.

HRMS (ESI) m/z: $[M+H]^+$ Calcd. for: $C_{29}H_{37}BNO_6$ 506.2708; Found: 506.2706.

1,3-dioxoisoindolin-2-yl 3-(4,4,5,5-tetramethyl-1,3,2-dioxaborolan-2-yl)pentadecanoate (1j)

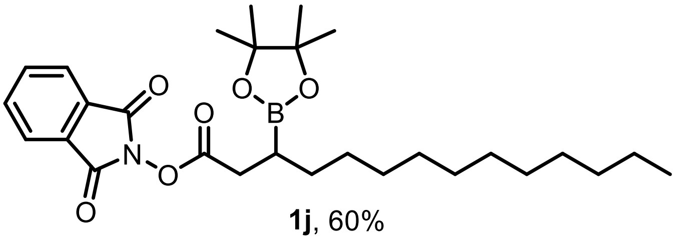

1j, 60%

Following the **general procedure 1** on 20 mmol scale, **1j** was obtained in 60% yield (6.0 g) as a white solid (mp: 65-67 °C), R_f = 0.4 (silica gel, PE: EA = 5:1, v/v), column chromatography (silica gel, PE: EA = 5:1, v/v).

^{1}H NMR (500 MHz, Chloroform-*d*) δ 7.87 (dd, *J* = 5.5, 3.1 Hz, 2H), 7.77 (dd, *J* = 5.5, 3.1 Hz, 2H), 2.81 (dd, *J* = 17.0, 8.0 Hz, 1H), 2.71 (dd, *J* = 17.1, 5.9 Hz, 1H), 1.53 – 1.47 (m, 2H), 1.33 – 1.21 (m, 31H), 0.87 (t, *J* = 6.9 Hz, 3H).

^{13}C NMR (126 MHz, Chloroform-*d*) δ 170.1, 161.9, 134.6, 129.0, 123.9, 83.5, 32.2, 31.9, 30.2, 29.7, 29.7, 29.6, 29.5, 29.4, 28.6, 24.8, 24.6, 22.7, 14.1.

^{11}B NMR (160 MHz, Chloroform-*d*) δ 34.30.

HRMS (ESI) m/z: $[M+H]^+$ Calcd. for: $C_{28}H_{42}BNO_6$ 500.3178; Found: 500.3173.

1,3-dioxoisoindolin-2-yl 5-(4-(tert-butyl)phenyl)-4-methyl-3-(4,4,5,5-tetramethyl-1,3,2-dioxaborolan-2-yl)pentanoate (1k)

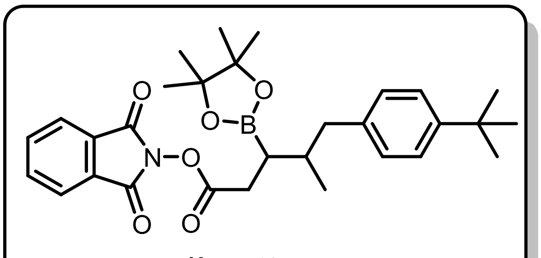

1k, 63%

Following the **general procedure 1** on 20 mmol scale, **1k** was obtained in 63% yield (6.5 g) as a white solid (mp: 87-89 °C), $R_f = 0.4$ (silica gel, PE: EA = 5:1, v/v), column chromatography (silica gel, PE: EA = 5:1, v/v).

^{1}H NMR (500 MHz, Chloroform-*d*) δ 7.88 (m, 2H), 7.78 (m, 2H), 7.32 – 7.28 (m, 2H), 7.17 – 7.09 (m, 2H), 2.94 (m, 1H), 2.85 – 2.67 (m, 2H), 2.02 (m, 1H), 1.71 – 1.59 (m, 1H), 1.31 (d, *J* = 1.8 Hz, 9H), 1.26 (dd, *J* = 10.5, 5.1 Hz, 12H), 0.91 (dd, *J* = 9.6, 6.8 Hz, 3H).

^{13}C NMR (126 MHz, Chloroform-*d*) δ 170.5, 170.4, 161.9, 148.6, 148.5, 138.0, 138.0, 134.7, 129.0, 128.9, 128.8, 125.2, 125.1, 123.9, 83.7, 83.7, 42.1, 41.5, 36.6, 35.9, 34.4, 31.5, 30.8, 29.4, 25.1, 24.9, 24.6, 18.1, 17.9.

^{11}B NMR (160 MHz, Chloroform-*d*) δ 34.09.

HRMS (ESI) m/z: $[M+H]^+$ Calcd. for: $C_{30}H_{38}BNO_6$ 520.2865; Found: 520.2863.

1,3-dioxoisoindolin-2-yl 3-(2,4-dimethylcyclohex-3-en-1-yl)-3-(4,4,5,5-tetramethyl-1,3,2-dioxaborolan-2-yl)propanoate (1l)

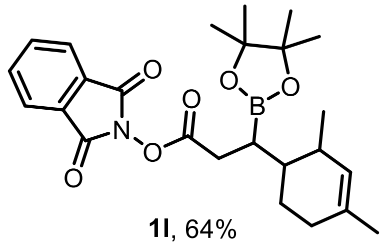

1l, 64%

Following the **general procedure 1** on 20 mmol scale, **1l** was obtained in 64%% yield (5.8 g) as a white solid (mp: 82-85 °C), $R_f = 0.4$ (silica gel, PE: EA = 5:1, v/v), column chromatography (silica gel, PE: EA = 5:1, v/v).

^{1}H NMR (500 MHz, Chloroform-*d*) δ 7.89 – 7.85 (m, 2H), 7.77 (dd, *J* = 5.5, 3.1 Hz, 2H), 5.35 (m, 1H), 2.90 – 2.77 (m, 2H), 2.23 (m, 1H), 2.01 – 1.90 (m, 2H), 1.80 – 1.72 (m, 1H), 1.66 – 1.59 (m, 4H), 1.49 – 1.38 (m, 2H), 1.22 (d, *J* = 7.0 Hz, 12H), 0.90 (d, *J* = 7.0 Hz, 3H).

^{13}C NMR (126 MHz, Chloroform-*d*) δ 170.5, 161.9, 134.7, 132.6, 129.0, 127.9, 123.9, 83.5, 38.7, 32.8, 31.2, 31.1, 24.9, 24.9, 24.6, 23.4, 22.4, 15.5.

^{11}B NMR (160 MHz, Chloroform-*d*) δ 34.25.

HRMS (ESI) m/z: $[M+H]^+$ Calcd. for: $C_{25}H_{32}BNO_6$ 454.2395; Found: 454.2394.

1,3-dioxoisoindolin-2-yl 4-ethyl-3-(4,4,5,5-tetramethyl-1,3,2-dioxaborolan-2-yl)hexanoate (1m)

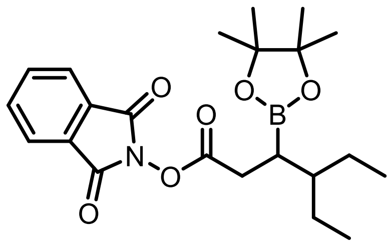

1m, 67%

Following the **general procedure 1** on 20 mmol scale, **1m** was obtained in 67% yield (5.6 g) as a white solid (mp: 75-77 °C), R_f = 0.4 (silica gel, PE: EA = 5:1, v/v), column chromatography (silica gel, PE: EA = 5:1, v/v).

^{1}H NMR (500 MHz, Chloroform-*d*) δ 7.41 – 7.37 (m, 2H), 7.27 (m, 3H), 2.97 (m, 1H), 1.59 (m, 1H), 1.55 – 1.46 (m, 2H), 1.44 – 1.38 (m, 1H), 1.28 (s, 13H), 1.20 (dd, *J* = 15.1, 10.2 Hz, 1H), 1.06 (dd, *J* = 15.2, 5.9 Hz, 1H), 0.96 (m, 6H).

^{13}C NMR (126 MHz, Chloroform-*d*) δ 131.5, 128.1, 127.2, 124.5, 93.9, 83.2, 81.5, 46.8, 30.4, 25.0, 24.8, 24.7, 23.9, 22.8, 12.0, 11.8.

^{11}B NMR (160 MHz, Chloroform-*d*) δ 33.89.

HRMS (ESI) m/z: $[M+H]^+$ Calcd. for: $C_{22}H_{30}BNO_6$ 416.2239; Found: 416.2235.

1,3-dioxoisoindolin-2-yl 3-(cyclohex-2-en-1-yl)-3-(4,4,5,5-tetramethyl-1,3,2-dioxaborolan-2-yl)propanoate (1n)

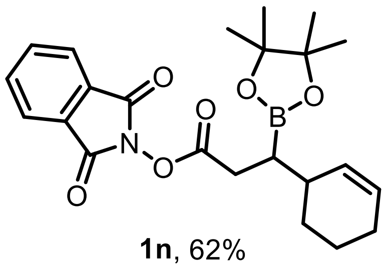

1n, 62%

Following the **general procedure 1** on 20 mmol scale, **1n** was obtained in 62% yield (5.3 g) as a white solid (mp: 95-97 °C), R_f = 0.4 (silica gel, PE: EA = 5:1, v/v), column chromatography (silica gel, PE: EA = 5:1, v/v).

^{1}H NMR (500 MHz, Chloroform-*d*) δ 7.37 (m, 2H), 7.26 – 7.24 (m, 2H), 5.73 – 5.64 (m, 2H), 2.83 – 2.70 (m, 1H), 2.26 – 1.91 (m, 5H), 1.72 – 1.61 (m, 1H), 1.46 – 1.37 (m, 1H), 1.26 (s, 12H), 1.21 – 1.05 (m, 2H).

^{13}C NMR (126 MHz, Chloroform-*d*) δ 131.6, 128.1, 127.3, 127.1, 126.8, 126.7, 126.5, 124.3, 93.4, 93.1, 83.3, 82.1, 81.9, 39.6, 39.4, 33.7, 33.4, 30.1, 27.9, 27.5, 25.7, 25.3, 25.0, 25.0, 24.7.

^{11}B NMR (160 MHz, Chloroform-*d*) δ 33.96.

HRMS (ESI) m/z: $[M+H]^+$ Calcd. for: $C_{23}H_{28}BNO_6$ 426.2082; Found: 426.2080.

1,3-dioxoisoindolin-2-yl 3-(4,4,5,5-tetramethyl-1,3,2-dioxaborolan-2-yl)nonanoate (1o)

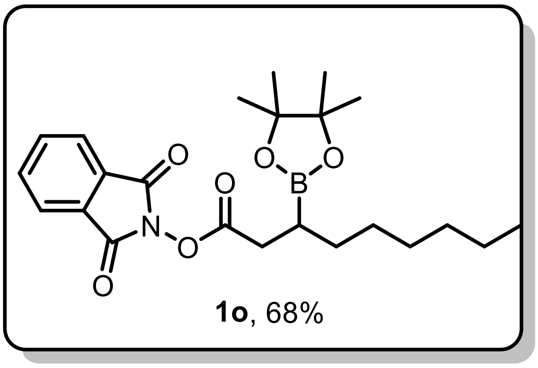

1o, 68%

Following the **general procedure 1** on 20 mmol scale, **1o** was obtained in 68% yield (5.8 g) as a white solid (mp: 91-93 °C), R_f = 0.4 (silica gel, PE: EA = 5:1, v/v), column chromatography (silica gel, PE: EA = 5:1, v/v).

^{1}H NMR (500 MHz, Chloroform-*d*) δ 7.87 (dd, *J* = 5.5, 3.1 Hz, 2H), 7.79 – 7.74 (m, 2H), 2.81 (dd, *J* = 17.0, 8.0 Hz, 1H), 2.71 (dd, *J* = 17.1, 6.0 Hz, 1H), 1.57 – 1.44 (m, 3H), 1.40 – 1.27 (m, 7H), 1.23 (d, *J* = 5.7 Hz, 13H), 0.88 (t, *J* = 6.9 Hz, 3H).

^{13}C NMR (126 MHz, Chloroform-*d*) δ 170.1, 161.9, 134.6, 129.0, 123.9, 83.5, 32.1, 31.9, 30.1, 28.2, 24.8, 24.5, 22.5, 14.0.

^{11}B NMR (160 MHz, Chloroform-*d*) δ 34.30.

HRMS (ESI) m/z: $[M+H]^+$ Calcd. for: $C_{23}H_{32}BNO_6$ 430.2395; Found: 430.2393.

1,3-dioxoisoindolin-2-yl 3-(4,4,5,5-tetramethyl-1,3,2-dioxaborolan-2-yl)hexanoate (1p)

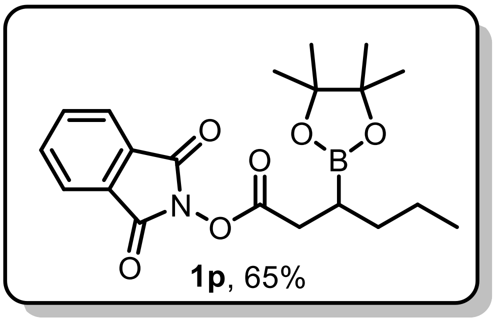

1p, 65%

Following the **general procedure 1** on 20 mmol scale, **1p** was obtained in 65% yield (5.0 g) as a white solid (mp: 73-75 °C), R_f = 0.4 (silica gel, PE: EA = 5:1, v/v), column chromatography (silica gel, PE: EA = 5:1, v/v).

^{1}H NMR (500 MHz, Chloroform-*d*) δ 7.87 (dd, *J* = 5.5, 3.1 Hz, 2H), 7.77 (dd, *J* = 5.5, 3.1 Hz, 2H), 2.81 (dd, *J* = 17.0, 7.9 Hz, 1H), 2.71 (dd, *J* = 17.0, 5.9 Hz, 1H), 1.56 – 1.44 (m, 3H), 1.37 – 1.27 (m, 6H), 1.23 (d, *J* = 5.6 Hz, 12H), 0.88 (t, *J* = 6.8 Hz, 3H).

^{13}C NMR (126 MHz, Chloroform-*d*) δ 170.1, 161.9, 134.7, 129.0, 123.9, 83.5, 32.2, 31.9, 30.2, 28.2, 24.8, 24.6, 22.5, 14.0.

^{11}B NMR (160 MHz, Chloroform-*d*) δ 34.34.

HRMS (ESI) m/z: $[M+H]^+$ Calcd. for: $C_{20}H_{27}BNO_6$ 388.1926; Found: 388.1923.

1,3-dioxoisoindolin-2-yl 2-(1-(4,4,5,5-tetramethyl-1,3,2-dioxaborolan-2-yl)cyclobutyl)acetate (1q)

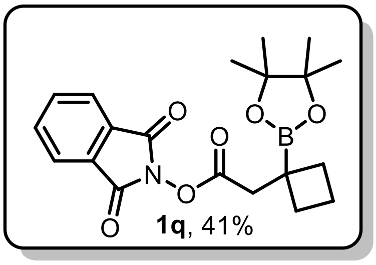

Following the **general procedure 1** on 20 mmol scale, **1q** was obtained in 41% yield (3.16 g) as a white solid (mp: 66-68 °C), R_f = 0.4 (silica gel, PE: EA = 5:1, v/v), column chromatography (silica gel, PE: EA = 5:1, v/v).

^{1}H NMR (500 MHz, Chloroform-*d*) δ 7.80 (dd, *J* = 5.5, 3.1 Hz, 2H), 7.70 (dd, *J* = 5.5, 3.1 Hz, 2H), 2.88 (s, 2H), 2.22 – 2.15 (m, 2H), 2.03 – 1.88 (m, 2H), 1.85 – 1.77 (m, 2H), 1.18 (s, 12H).

^{13}C NMR (126 MHz, Chloroform-*d*) δ 169.2, 161.9, 134.6, 129.0, 123.9, 83.6, 39.9, 29.4, 24.5, 18.1.

^{11}B NMR (160 MHz, Chloroform-d) δ 34.23.

HRMS (ESI) m/z: $[M+H]^+$ Calcd. for: $C_{20}H_{24}BNO_6$ 386.1769; Found: 386.1765.

1,3-dioxoisoindolin-2-yl 3-(5,5-dimethyl-1,3,2-dioxaborinan-2-yl)-5,5-dimethylhexanoate (1r)

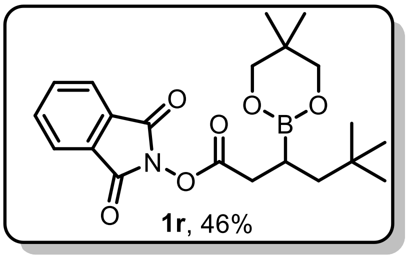

Following the **general procedure 1** on 20 mmol scale, **1r** was obtained in 46% yield (3.69 g) as a white solid (mp: 113-115 °C), R_f = 0.4 (silica gel, PE: EA = 5:1, v/v), column chromatography (silica gel, PE: EA = 5:1, v/v).

^{1}H NMR (500 MHz, Chloroform-*d*) δ 7.89 – 7.84 (m, 2H), 7.79 – 7.76 (m, 2H), 3.60 (d, *J* = 0.8 Hz, 4H), 2.75 (dd, *J* = 16.6, 8.2 Hz, 1H), 2.66 (dd, *J* = 16.6, 6.9 Hz, 1H), 1.61 (t, *J* = 4.2 Hz, 1H), 1.45 – 1.39 (m, 1H), 1.23 (dd, *J* = 13.7, 4.4 Hz, 1H), 0.93 (dd, *J* = 8.7, 0.9 Hz, 15H).

^{13}C NMR (126 MHz, Chloroform-*d*) δ 170.3, 162.0, 134.6, 129.1, 123.9, 72.0, 44.8, 34.4, 31.7, 31.2, 29.6, 22.0.

^{11}B NMR (160 MHz, Chloroform-*d*) δ 33.72.

HRMS (ESI) m/z: $[M+H]^+$ Calcd. for: $C_{21}H_{28}BNO_6$ 402.2082; Found: 402.2080.

1,3-dioxoisoindolin-2-yl 3,3-bis(4,4,5,5-tetramethyl-1,3,2-dioxaborolan-2-yl)butanoate (1s)

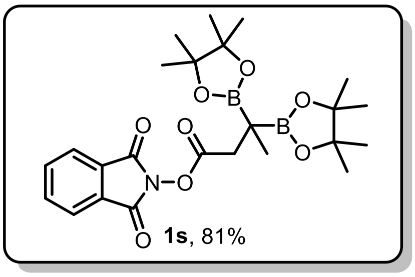

Following the **general procedure 1** on 10 mmol scale, **1s** was obtained in 81% yield (3.9 g) as a white solid (mp: 103-105 °C), R_f = 0.4 (silica gel, PE: EA = 5:1, v/v), column chromatography (silica gel, PE: EA = 5:1, v/v).

^{1}H NMR (500 MHz, Chloroform-*d*) δ 7.86 (dd, *J* = 5.5, 3.1 Hz, 2H), 7.76 (dd, *J* = 5.5, 3.1 Hz, 2H), 2.94 (s, 2H), 1.21 (d, *J* = 4.5 Hz, 27H).

^{13}C NMR (126 MHz, Chloroform-*d*) δ 170.3, 161.9, 134.6, 129.1, 124.0, 123.8, 83.6, 35.5, 24.6, 24.6, 16.2.

^{11}B NMR (160 MHz, Chloroform-*d*) δ34.37, 32.90.

HRMS (ESI) m/z: $[M+H]^+$ Calcd. for: $C_{24}H_{33}B_2NO_8$ 486.2465; Found: 486.2463.

1,3-dioxoisoindolin-2-yl 3,3-bis(4,4,5,5-tetramethyl-1,3,2-dioxaborolan-2-yl)pentanoate (1t)

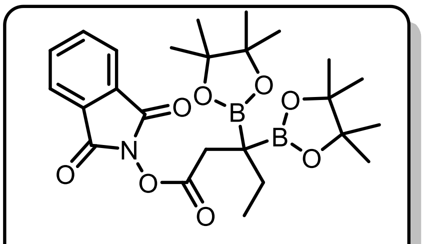

1t, 76%

Following the **general procedure 1** on 10 mmol scale, **1t** was obtained in 76% yield (3.8 g) as a white solid (mp: 113-115 °C), R_f = 0.4 (silica gel, PE: EA = 5:1, v/v), column chromatography (silica gel, PE: EA = 5:1, v/v).

^{1}H NMR (500 MHz, Chloroform-*d*) δ 7.84 (dd, *J* = 5.5, 3.1 Hz, 2H), 7.75 (dd, *J* = 5.5, 3.1 Hz, 2H), 3.01 (s, 2H), 1.79 (d, *J* = 7.5 Hz, 2H), 1.20 (d, *J* = 1.9 Hz, 24H), 0.92 (t, *J* = 7.5 Hz, 3H).

^{13}C NMR (126 MHz, Chloroform-*d*) δ 170.3, 161.9, 134.6, 129.1, 123.8, 83.6, 31.1, 24.7, 22.4, 11.5.

^{11}B NMR (160 MHz, Chloroform-*d*) δ 35.04, 34.43.

HRMS (ESI) m/z: $[M+H]^+$ Calcd. for: $C_{25}H_{35}B_2NO_8$ 500.2622; Found: 500.2620.

1,3-dioxoisoindolin-2-yl 3,3-bis(4,4,5,5-tetramethyl-1,3,2-dioxaborolan-2-yl)heptanoate (1u)

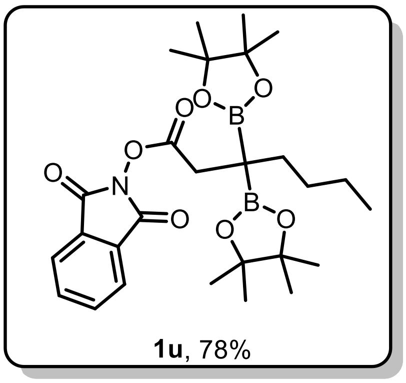

1u, 78%

Following the **general procedure 1** on 10 mmol scale, **1u** was obtained in 78% yield (4.0 g) as a white solid (mp: 142-144 °C), R_f = 0.4 (silica gel, PE: EA = 5:1, v/v), column chromatography (silica gel, PE: EA = 5:1, v/v).

^{1}H NMR (500 MHz, Chloroform-*d*) δ 7.86 (dd, *J* = 5.5, 3.1 Hz, 2H), 7.75 (dd, *J* = 5.5, 3.1 Hz, 2H), 3.01 (s, 2H), 1.76 – 1.71 (m, 2H), 1.29 – 1.27 (m, 4H), 1.21 (s, 24H), 0.90 – 0.84 (m, 3H).

^{13}C NMR (126 MHz, Chloroform-*d*) δ 170.4, 161.9, 134.5, 129.1, 123.8, 83.6, 31.8, 31.5, 29.8, 29.5, 27.4, 24.7, 24.6, 22.6, 14.1.

^{11}B NMR (160 MHz, Chloroform-*d*) δ 34.42, 32.77.

HRMS (ESI) m/z: $[M+H]^+$ Calcd. for: $C_{27}H_{40}B_2NO_8$ 528.2935; Found: 528.2938.

2.5 Characterization data for Products

2-(4,4-dimethyl-2-(phenylethynyl)pentyl)-4,4,5,5-tetramethyl-1,3,2-dioxaborolane (3a)

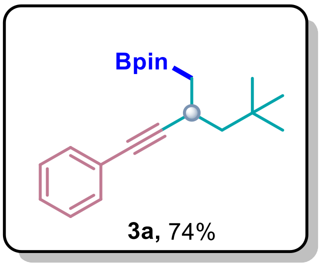

Following the **general procedure 5** on 0.2 mmol scale, colorless oil, yield: 74% (48.3 mg), $R_f = 0.4$ (silica gel, PE: EA = 30:1, v/v), column chromatography (silica gel, PE: EA = 30:1, v/v).

^{1}H NMR (500 MHz, Chloroform-*d*) δ 7.37 – 7.33 (m, 2H), 7.27 – 7.22 (m, 3H), 2.83 (m, 1H), 1.37 (d, *J* = 2.5 Hz, 1H), 1.34 (dd, *J* = 6.0, 2.4 Hz, 1H), 1.26 (d, *J* = 1.6 Hz, 12H), 1.19 (dd, *J* = 15.3, 8.4 Hz, 1H), 1.08 (dd, *J* = 15.3, 6.9 Hz, 1H), 1.02 (s, 9H).

^{13}C NMR (126 MHz, Chloroform-*d*) δ 131.23, 128.04, 127.15, 124.52, 96.58, 83.17, 51.79, 31.13, 29.95, 24.87, 24.79, 24.07.

^{11}B NMR (160 MHz, Chloroform-*d*) δ 33.41.

HRMS (ESI) m/z: $[M+H]^+$ Calcd. for $C_{21}H_{31}BO_2$ 327.2490; Found: 327.2487.

2-(4,4-dimethyl-2-(p-tolylethynyl)pentyl)-4,4,5,5-tetramethyl-1,3,2-dioxaborolane (3b)

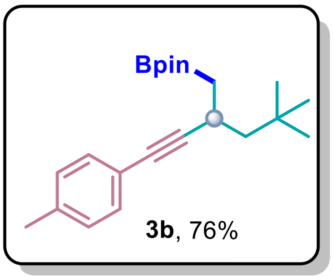

Following the **general procedure 5** on 0.2 mmol scale, colorless oil, yield: 76% (51.7 mg), $R_f = 0.4$ (silica gel, PE: EA = 30:1, v/v), column chromatography (silica gel, PE: EA = 30:1, v/v).

^{1}H NMR (500 MHz, Chloroform-*d*) δ 7.24 (d, *J* = 7.8 Hz, 2H), 7.06 (d, *J* = 7.8 Hz, 2H), 2.83 (m, 1H), 2.31 (s, 3H), 1.68 – 1.61 (m, 1H), 1.37 (d, *J* = 2.8 Hz, 1H), 1.26 (s, 12H), 1.20 – 1.15 (m, 1H), 1.10 – 1.05 (m, 1H), 1.02 (s, 9H).

^{13}C NMR (126 MHz, Chloroform-*d*) δ 137.0, 131.1, 128.8, 121.5, 95.7, 83.1, 51.8, 31.1, 30.0, 24.9, 24.8, 24.1, 21.3.

^{11}B NMR (160 MHz, Chloroform-*d*) δ 34.03.

HRMS (ESI) m/z: $[M+Na]^+$ Calcd. for $C_{22}H_{33}BO_2$ 363.2466; Found: 363.2461.

2-(2-((4-fluorophenyl)ethynyl)-4,4-dimethylpentyl)-4,4,5,5-tetramethyl-1,3,2-dioxaborolane (3c)

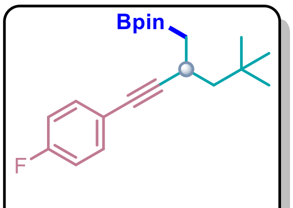

3c, 68%

Following the **general procedure 5** on 0.2 mmol scale, colorless oil, yield: 68% (44.1 mg), $R_f = 0.4$ (silica gel, PE: EA = 30:1, v/v), column chromatography (silica gel, PE: EA = 30:1, v/v).

^{1}H NMR (500 MHz, Chloroform-*d*) δ 7.34 – 7.28 (m, 2H), 6.97 – 6.91 (m, 2H), 2.86 – 2.76 (m, 1H), 1.62 (dd, *J* = 13.6, 10.4 Hz, 1H), 1.38 – 1.32 (m, 1H), 1.26 – 1.23 (m, 12H), 1.17 (dd, *J* = 15.3, 8.3 Hz, 1H), 1.07 (dd, *J* = 15.3, 6.9 Hz, 1H), 1.00 (s, 9H).

^{13}C NMR (126 MHz, Chloroform-*d*) δ 162.9, 160.9, 133.0, 132.9, 120.6, 120.6, 115.4, 115.2, 96.2, 83.2, 51.8, 31.2, 30.0, 24.9, 24.8, 24.1.

^{11}B NMR (160 MHz, Chloroform-*d*) δ 33.66.

^{19}F NMR (471 MHz, Chloroform-*d*) δ -112.88.

HRMS (ESI) m/z: $[M+H]^+$ Calcd. for $C_{21}H_{30}BFO_2$ 352.2545; Found: 325.2545.

2-(2-((4-chlorophenyl)ethynyl)-4,4-dimethylpentyl)-4,4,5,5-tetramethyl-1,3,2-dioxaborolane (3d)

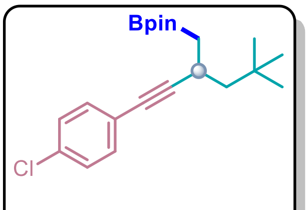

3d, 66%

Following the **general procedure 5** on 0.2 mmol scale, colorless oil, yield: 66% (47.5 mg), $R_f = 0.4$ (silica gel, PE: EA = 30:1, v/v), column chromatography (silica gel, PE: EA = 30:1, v/v).

^{1}H NMR (500 MHz, Chloroform-*d*) δ 7.86 (dd, *J* = 5.5, 3.1 Hz, 2H), 7.76 (dd, *J* = 5.5, 3.1 Hz, 2H), 2.73 (m, 2H), 1.65 (m, 1H), 1.56 (m, 1H), 1.43 (m, 1H), 1.33 – 1.27 (m, 1H), 1.22 (d, *J* = 5.2 Hz, 12H), 0.90 (dd, *J* = 11.9, 6.6 Hz, 6H).

^{13}C NMR (126 MHz, Chloroform-*d*) δ 133.1, 132.5, 128.4, 123.0, 97.7, 83.2, 51.7, 31.2, 30.0, 24.9, 24.8, 24.1.

^{11}B NMR (160 MHz, Chloroform-*d*) δ 33.77.

HRMS (ESI) m/z: $[M+H]^+$ Calcd. for $C_{21}H_{30}BClO_2$ 361.2100; Found: 361.2098.

2-(2-((4-bromophenyl)ethynyl)-4,4-dimethylpentyl)-4,4,5,5-tetramethyl-1,3,2-dioxaborolane (3e)

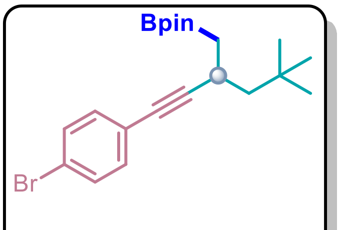

3e, 69%

Following the **general procedure 5** on 0.2 mmol scale, colorless oil, yield: 69% (55.8 mg), R_f = 0.4 (silica gel, PE: EA = 30:1, v/v), column chromatography (silica gel, PE: EA = 30:1, v/v).

^{1}H NMR (500 MHz, Chloroform-*d*) δ 7.41 – 7.34 (m, 2H), 7.23 – 7.17 (m, 2H), 2.84 – 2.75 (m, 1H), 1.66 – 1.61 (m, 1H), 1.38 – 1.32 (m, 1H), 1.24 (s, 12H), 1.18 – 1.13 (m, 1H), 1.07 (dd, *J* = 15.4, 6.8 Hz, 1H), 1.00 (s, 9H).

^{13}C NMR (126 MHz, Chloroform-*d*) δ 132.8, 131.3, 123.5, 121.3, 97.9, 83.2, 51.7, 31.2, 30.0, 24.9, 24.82, 2.14, 1.0.

^{11}B NMR (160 MHz, Chloroform-*d*) δ 33.64.

HRMS (ESI) m/z: $[M+H]^+$ Calcd. for $C_{21}H_{30}BBrO_2$ 405.1595; Found: 405.1595.

2-(2-((4-methoxyphenyl)ethynyl)-4,4-dimethylpentyl)-4,4,5,5-tetramethyl-1,3,2-dioxaborolane (3f)

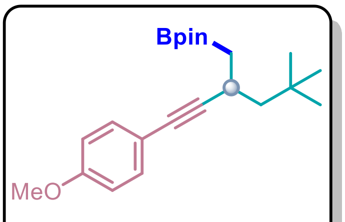

3f, 70%

Following the **general procedure 5** on 0.2 mmol scale, colorless oil, yield: 70% (49.8 mg), R_f = 0.4 (silica gel, PE: EA = 30:1, v/v), column chromatography (silica gel, PE: EA = 30:1, v/v).

^{1}H NMR (500 MHz, Chloroform-*d*) δ 7.26 (dd, *J* = 6.1, 2.6 Hz, 2H), 6.85 – 6.79 (m, 2H), 6.30 (d, *J* = 15.8 Hz, 1H), 5.88 (dd, *J* = 15.8, 8.9 Hz, 1H), 3.79 (s, 3H), 2.50 (m, 1H), 1.58 (m, 1H), 1.34 – 1.27 (m, 2H), 1.20 (d, *J* = 3.3 Hz, 13H), 0.95 (dd, *J* = 15.1, 6.1 Hz, 1H), 0.86 (dd, *J* = 6.6, 4.9 Hz, 6H).

^{13}C NMR (126 MHz, Chloroform-*d*) δ 158.5, 134.7, 130.8, 127.7, 127.1, 113.8, 83.0, 55.3, 47.6, 37.1, 25.7, 25.0, 24.8, 23.6, 22.0.

^{11}B NMR (160 MHz, Chloroform-*d*) δ 33.91.

HRMS (ESI) m/z: $[M+H]^+$ Calcd. for $C_{22}H_{33}BO_3$ 357.2596; Found: 357.2596.

2-(2-([1,1'-biphenyl]-4-ylethynyl)-4,4-dimethylpentyl)-4,4,5,5-tetramethyl-1,3,2-dioxaborolane (3g)

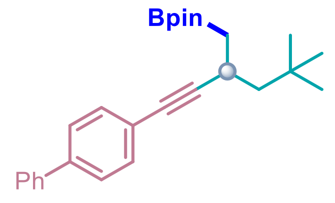

3g, 61%

Following the **general procedure 5** on 0.2 mmol scale, colorless oil, yield: 61% (49.0 mg), R_f = 0.4 (silica gel, PE: EA = 30:1, v/v), column chromatography (silica gel, PE: EA = 30:1, v/v).

^{1}H NMR (500 MHz, Chloroform-*d*) δ 7.61 – 7.56 (m, 2H), 7.54 – 7.49 (m, 2H), 7.44 (m, 4H), 7.35 (m, 1H), 2.88 (m, 1H), 1.72 – 1.64 (m, 1H), 1.40 (dd, *J* = 13.6, 2.9 Hz, 1H), 1.29 (d, *J* = 1.7 Hz, 12H), 1.24 – 1.20 (m, 1H), 1.05 (s, 10H).
^{13}C NMR (126 MHz, Chloroform-*d*) δ 140.6, 140.0, 131.7, 128.8, 127.4, 127.0, 126.8, 123.6, 97.4, 83.3, 51.9, 31.2, 30.0, 30.0, 25.0, 24.9, 24.2.
^{11}B NMR (160 MHz, Chloroform-*d*) δ 33.58.
HRMS (ESI) m/z: $[M+K]^+$ Calcd. for $C_{27}H_{35}BO_2$ 441.2367; Found: 441.2365.

2-(2-cyclopentyl-4-phenylbut-3-yn-1-yl)-4,4,5,5-tetramethyl-1,3,2-dioxaborolane (3h)

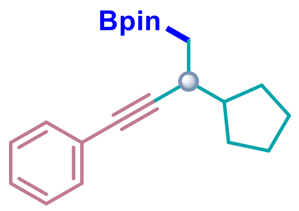

3h, 71%

Following the **general procedure 5** on 0.2 mmol scale, colorless oil, yield: 71% (46.0 mg), R_f = 0.4 (silica gel, PE: EA = 30:1, v/v), column chromatography (silica gel, PE: EA = 30:1, v/v).
^{1}H NMR (500 MHz, Chloroform-*d*) δ 7.39 – 7.35 (m, 2H), 7.27 – 7.23 (m, 3H), 2.77 (dt, *J* = 9.6, 6.4 Hz, 1H), 2.00 – 1.91 (m, 1H), 1.88 – 1.74 (m, 2H), 1.66 (p, *J* = 5.2, 4.7 Hz, 2H), 1.58 – 1.47 (m, 3H), 1.40 – 1.33 (m, 1H), 1.26 (s, 12H), 1.15 – 1.07 (m, 2H).
^{13}C NMR (126 MHz, Chloroform-*d*) δ 131.54, 128.09, 127.95, 127.25, 124.43, 94.05, 83.23, 46.22, 32.96, 31.07, 29.65, 25.72, 25.61, 25.01, 24.78, 24.68.
^{11}B NMR (160 MHz, Chloroform-*d*) δ 33.85.
HRMS (ESI) m/z: $[M+H]^+$ Calcd. for $C_{21}H_{29}BO_2$ 325.2333; Found: 325.2337.

2-(2-cyclohexyl-4-phenylbut-3-yn-1-yl)-4,4,5,5-tetramethyl-1,3,2-dioxaborolane (3i)

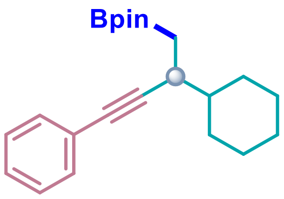

3i, 70%

Following the **general procedure 5** on 0.2 mmol scale, colorless oil, yield: 70% (47.3 mg), R_f = 0.4 (silica gel, PE: EA = 30:1, v/v), column chromatography (silica gel, PE: EA = 30:1, v/v).
^{1}H NMR (500 MHz, Chloroform-*d*) δ 7.42 – 7.38 (m, 2H), 7.27 (m, 3H), 2.69 (m, 1H), 1.98 – 1.91 (m, 1H), 1.84 – 1.74 (m, 3H), 1.68 – 1.64 (m, 1H), 1.40 – 1.36 (m, 1H), 1.27 (s, 12H), 1.24 – 1.13 (m, 6H), 1.08 (dd, *J* = 15.2, 5.7 Hz, 1H).
^{13}C NMR (126 MHz, Chloroform-*d*) δ 131.5, 128.1, 127.2, 124.5, 93.8, 83.2, 81.9, 43.4, 34.1, 31.4, 29.2, 26.5, 26.4, 25.0, 24.7.
^{11}B NMR (160 MHz, Chloroform-*d*) δ 33.65.

HRMS (ESI) m/z: $[M+H]^+$ Calcd. for $C_{22}H_{31}BO_2$ 369.2596; Found: 369.2591.

2-(3-ethyl-2-(phenylethynyl)pentyl)-4,4,5,5-tetramethyl-1,3,2-dioxaborolane (3j)

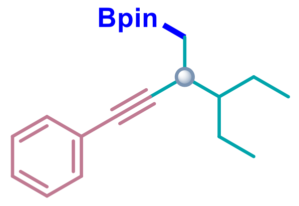

3j, 75%

Following the **general procedure 5** on 0.2 mmol scale, colorless oil, yield: 75% (48.9 mg), R_f = 0.4 (silica gel, PE: EA = 30:1, v/v), column chromatography (silica gel, PE: EA = 30:1, v/v).
^{1}H NMR (500 MHz, Chloroform-*d*) δ 7.41 – 7.37 (m, 2H), 7.27 (m, 3H), 2.97 (m, 1H), 1.59 (m, 1H), 1.55 – 1.46 (m, 2H), 1.44 – 1.38 (m, 1H), 1.28 (s, 13H), 1.20 (dd, *J* = 15.1, 10.2 Hz, 1H), 1.06 (dd, *J* = 15.2, 5.9 Hz, 1H), 0.96 (m, 6H).
^{13}C NMR (126 MHz, Chloroform-*d*) δ 131.5, 128.0, 127.2, 124.5, 93.9, 83.2, 81.5, 46.8, 30.4, 25.0, 24.8, 24.7, 23.9, 22.8, 12.0, 11.8.
^{11}B NMR (160 MHz, Chloroform-*d*) δ 33.89.
HRMS (ESI) m/z: $[M+H]^+$ Calcd. for $C_{21}H_{31}BO_2$ 327.2490; Found: 327.2495.

4,4,5,5-tetramethyl-2-(3-methyl-2-(phenylethynyl)hexyl)-1,3,2-dioxaborolane (3k)

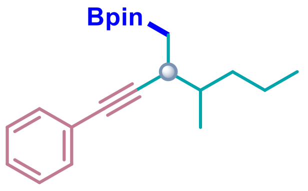

3k, 73%

Following the **general procedure 5** on 0.2 mmol scale, colorless oil, yield: 73% (47.6 mg), R_f = 0.4 (silica gel, PE: EA = 30:1, v/v), column chromatography (silica gel, PE: EA = 30:1, v/v).
^{1}H NMR (500 MHz, Chloroform-*d*) δ 7.40 – 7.35 (m, 2H), 7.29 – 7.21 (m, 3H), 2.91 – 2.72 (m, 1H), 1.60 (m, 1H), 1.55 – 1.34 (m, 3H), 1.26 (s, 14H), 1.19 – 1.09 (m, 1H), 1.07 – 0.96 (m, 4H), 0.95 – 0.89 (m, 3H).
^{13}C NMR (126 MHz, Chloroform-*d*) δ 131.5, 131.5, 128.0, 127.2, 124.5, 124.5, 94.0, 93.1, 83.2, 82.0, 81.7, 38.1, 38.0, 37.8, 35.6, 33.8, 33.1, 25.0, 25.0, 24.7, 24.7, 20.5, 20.4, 17.3, 15.4, 14.4, 14.3.
^{11}B NMR (160 MHz, Chloroform-*d*) δ 33.86.
HRMS (ESI) m/z: $[M+H]^+$ Calcd. for $C_{21}H_{31}BO_2$ 327.2490; Found: 327.2487.

4,4,5,5-tetramethyl-2-(2-phenethyl-4-phenylbut-3-yn-1-yl)-1,3,2-dioxaborolane (3l)

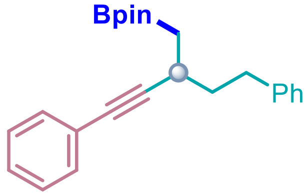

3l, 66%

Following the **general procedure 5** on 0.2 mmol scale, colorless oil, yield: 66% (47.5 mg), R_f = 0.4 (silica gel, PE: EA = 30:1, v/v), column chromatography (silica gel, PE: EA = 30:1, v/v).
^{1}H NMR (500 MHz, Chloroform-*d*) δ 7.46 – 7.39 (m, 2H), 7.34 – 7.26 (m, 5H), 7.26 – 7.22 (m, 2H), 7.21 – 7.17 (m, 1H), 2.92 (m, 1H), 2.87 – 2.74 (m, 2H), 1.94 – 1.84 (m, 2H), 1.26 (s, 12H), 1.24 – 1.17 (m, 1H), 1.13 (m, 1H).
^{13}C NMR (126 MHz, Chloroform-*d*) δ 142.3, 131.6, 128.6, 128.4, 128.2, 127.4, 125.8, 124.2, 94.3, 83.3, 39.3, 33.9, 27.7, 24.9, 24.8.
^{11}B NMR (160 MHz, Chloroform-*d*) δ 33.84.
HRMS (ESI) m/z: $[M+H]^+$ Calcd. For $C_{24}H_{29}BO_2$ 361.2333; Found: 361.2337.

4,4,5,5-tetramethyl-2-(2-(phenylethynyl)pentyl)-1,3,2-dioxaborolane (3m)

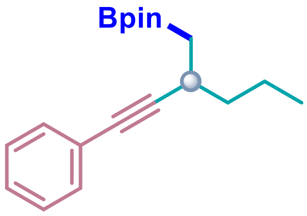

3m, 63%

Following the **general procedure 5** on 0.2 mmol scale, colorless oil, yield: 63% (37.5 mg), R_f = 0.4 (silica gel, PE: EA = 30:1, v/v), column chromatography (silica gel, PE: EA = 30:1, v/v).
^{1}H NMR (500 MHz, Chloroform-*d*) δ 7.42 – 7.38 (m, 2H), 7.29 – 7.25 (m, 3H), 2.84 – 2.77 (m, 1H), 1.56 (dd, *J* = 7.0, 4.4 Hz, 2H), 1.34 (dd, *J* = 4.9, 2.8 Hz, 2H), 1.28 (d, *J* = 2.2 Hz, 12H), 1.19 – 1.09 (m, 2H), 0.92 (t, *J* = 6.8 Hz, 3H).
^{13}C NMR (126 MHz, Chloroform-*d*) δ 131.5, 128.1, 128.1, 128.0, 127.3, 124.4, 95.0, 83.2, 37.5, 31.7, 28.0, 27.2, 24.9, 24.8, 24.8, 22.6, 14.1.
^{11}B NMR (160 MHz, Chloroform-*d*) δ 32.96.
HRMS (ESI) m/z: $[M+H]^+$ Calcd. for $C_{19}H_{27}BO_2$ 299.2177; Found: 299.2173.

4,4,5,5-tetramethyl-2-(2-(phenylethynyl)octyl)-1,3,2-dioxaborolane (3n)

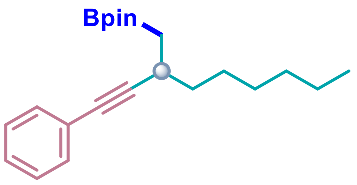

3n, 65%

Following the **general procedure 5** on 0.2 mmol scale, colorless oil, yield: 65% (44.2 mg), R_f = 0.4 (silica gel, PE: EA = 30:1, v/v), column chromatography (silica gel, PE: EA = 30:1, v/v).

^{1}H NMR (500 MHz, Chloroform-*d*) δ 7.39 – 7.35 (m, 2H), 7.28 – 7.23 (m, 3H), 2.82 – 2.73 (m, 1H), 1.55 – 1.51 (m, 2H), 1.48 – 1.40 (m, 1H), 1.34 – 1.28 (m, 6H), 1.25 (d, *J* = 2.2 Hz, 12H), 1.21 (t, *J* = 2.9 Hz, 1H), 1.17 – 1.05 (m, 2H), 0.91 – 0.86 (m, 3H).

^{13}C NMR (126 MHz, Chloroform-*d*) δ 131.5, 128.1, 128.1, 128.0, 127.3, 124.4, 95.0, 83.2, 37.6, 31.8, 29.1, 28.0, 27.5, 24.9, 24.8, 22.7, 14.1.

^{11}B NMR (160 MHz, Chloroform-*d*) δ 33.73.

HRMS (ESI) m/z: $[M+H]^+$ Calcd. for $C_{22}H_{33}BO_2$ 371.2752; Found: 371.2752.

4,4,5,5-tetramethyl-2-(4-methyl-2-(phenylethynyl)pentyl)-1,3,2-dioxaborolane (3o)

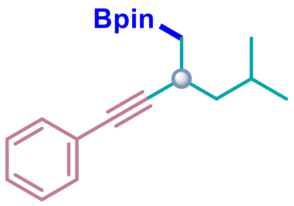

3o, 68%

Following the **general procedure 5** on 0.2 mmol scale, colorless oil, yield: 68% (42.4 mg), R_f = 0.4 (silica gel, PE: EA = 30:1, v/v), column chromatography (silica gel, PE: EA = 30:1, v/v).

^{1}H NMR (500 MHz, Chloroform-*d*) δ 7.39 – 7.35 (m, 2H), 7.28 – 7.21 (m, 3H), 2.84 (m, 1H), 1.89 (m, 1H), 1.54 (m, 1H), 1.26 (s, 13H), 1.16 – 1.04 (m, 2H), 0.94 (dd, *J* = 6.7, 1.9 Hz, 6H).

^{13}C NMR (126 MHz, Chloroform-*d*) δ 131.5, 128.1, 128.1, 127.3, 124.4, 94.0, 83.2, 5.10, 26.3, 26.1, 24.9, 24.8, 24.8, 23.4, 21.9.

^{11}B NMR (160 MHz, Chloroform-*d*) δ 35.05.

HRMS (ESI) m/z: $[M+H]^+$ Calcd. for $C_{20}H_{29}BO_2$ 313.2333; Found: 313.2334.

4,4,5,5-tetramethyl-2-((1-(phenylethynyl)cyclobutyl)methyl)-1,3,2-dioxaborolane (3p)

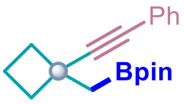

3p, 33%

Following the **general procedure 5** on 0.2 mmol scale, colorless oil, yield: 33% (20.5 mg), R_f = 0.4 (silica gel, PE: EA = 30:1, v/v), column chromatography (silica gel, PE: EA = 30:1, v/v).

^{1}H NMR (500 MHz, Chloroform-*d*) δ 7.39 – 7.37 (m, 2H), 7.28 – 7.23 (m, 3H), 2.45 – 2.39 (m, 2H), 2.20 – 2.09 (m, 2H), 2.04 – 1.94 (m, 2H), 1.36 (d, *J* = 7.6 Hz, 2H), 1.26 (d, *J* = 13.3 Hz, 12H).
^{13}C NMR (126 MHz, Chloroform-*d*) δ 131.6, 131.5, 128.0, 127.2, 98.1,83.0, 80.8, 36.7, 3
4.3, 31.5, 30.1, 28.9, 24.8, 24.7, 16.2.
^{11}B NMR (160 MHz, Chloroform-*d*) δ 33.15.
HRMS (ESI) m/z: $[M+H]^+$ Calcd. for $C_{20}H_{27}BO_2$ 297.2020; Found: 297.2025.

4,4,5,5-tetramethyl-2-(2-(phenylethynyl)tridecyl)-1,3,2-dioxaborolane (3q)

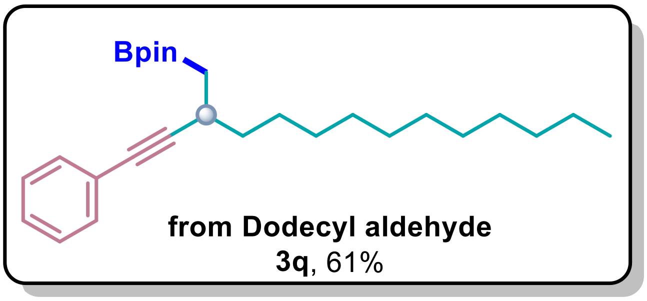

Following the **general procedure 5** on 0.2 mmol scale, colorless oil, yield: 61% (50.1 mg), R_f = 0.4 (silica gel, PE: EA = 30:1, v/v), column chromatography (silica gel, PE: EA = 30:1, v/v).
^{1}H NMR (500 MHz, Chloroform-*d*) δ 7.40 – 7.36 (m, 2H), 7.30 – 7.21 (m, 3H), 2.83 – 2.74 (m, 1H), 1.54 (m, 3H), 1.48 – 1.42 (m, 1H), 1.26 (s, 30H), 1.12 (m, 2H), 0.88 (t, *J* = 6.9 Hz, 3H).
^{13}C NMR (126 MHz, Chloroform-*d*) δ 131.5, 128.1, 128.1, 127.3, 124.4, 95.0, 83.2, 37.6, 32.0, 29.7, 29.7, 29.7, 29.6, 29.5, 29.4, 28.0, 27.5, 24.9, 24.8, 24.8, 22.7, 14.2.
^{11}B NMR (160 MHz, Chloroform-*d*) δ 34.20.
HRMS (ESI) m/z: $[M+H]^+$ Calcd. for $C_{27}H_{43}BO_2$ 411.3429; Found: 411.3427.

2-(2-(1-(4-isopropylphenyl)propan-2-yl)-4-phenylbut-3-yn-1-yl)-4,4,5,5-tetramethyl-1,3,2-dioxaborolane (3r)

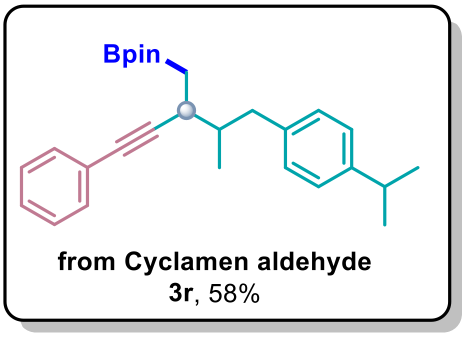

Following the **general procedure 5** on 0.2 mmol scale, colorless oil, yield: 58% (48.3 mg), R_f = 0.4 (silica gel, PE: EA = 30:1, v/v), column chromatography (silica gel, PE: EA = 30:1, v/v).
^{1}H NMR (500 MHz, Chloroform-*d*) δ 7.42 (m, 2H), 7.32 – 7.26 (m, 3H), 7.17 – 7.10 (m, 4H), 3.02 (dd, *J* = 13.4, 3.9 Hz, 0.5H), 2.92 – 2.80 (m, 2.5H), 2.52 (dd, *J* = 13.5, 8.3 Hz, 0.5H), 2.36 (dd, *J* = 13.4, 10.2 Hz, 0.5H), 1.88 (m, 1H), 1.28 (s, 6H), 1.25 (m, 13H), 1.19 – 1.13 (m, 1H), 1.04 (dd, *J* = 15.2, 6.3 Hz, 1H), 0.97 (dd, *J* = 17.3, 6.7 Hz, 3H).

^{13}C NMR (126 MHz, Chloroform-*d*) δ 146.2, 146.2, 138.8, 138.6, 131.6, 131.6, 129.2, 129.1, 128.1, 127.4, 126.2, 126.2, 124.4, 124.3, 93.6, 92.6, 83.3, 83.3, 82.5, 82.2, 41.7, 40.9, 40.5, 39.2, 33.9, 33.7, 33.7, 32.7, 25.0, 24.9, 24.7, 24.7, 24.1, 24.1, 17.2, 15.0.
^{11}B NMR (160 MHz, Chloroform-*d*) δ 33.91.
HRMS (ESI) m/z: $[M+H]^+$ Calcd. for $C_{28}H_{37}BO_2$ 417.2959; Found: 417.2959.

2-(2-(1-(4-isopropylphenyl)propan-2-yl)-4-phenylbut-3-yn-1-yl)-4,4,5,5-tetramethyl-1,3,2-dioxaborolane (3s)

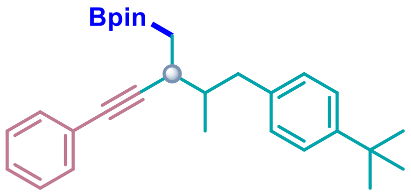

from Lily aldehyde
3s, 60%

Following the **general procedure 5** on 0.2 mmol scale, colorless oil, yield: 60% (50.0 mg), R_f = 0.4 (silica gel, PE: EA = 30:1, v/v), column chromatography (silica gel, PE: EA = 30:1, v/v).
^{1}H NMR (500 MHz, Chloroform-*d*) δ 7.43 (m, 2H), 7.30 (m, 5H), 7.14 (t, *J* = 7.9 Hz, 2H), 3.02 (dd, *J* = 13.4, 3.9 Hz, 0.5H), 2.95 – 2.82 (m, 1.5H), .53 (dd, *J* = 13.5, 8.2 Hz, 0.5H),)2.37 (dd, *J* = 13.4, 10.1 Hz, 0.5H), 1.91 (m, 1H), 1.33 – 1.25 (m, 21H), 1.17 (dd, *J* = 15.2, 5.5 Hz, 1H), 1.05 (dd, *J* = 15.3, 6.4 Hz, 1H), 0.98 (dd, *J* = 17.5, 6.6 Hz, 3H).
^{13}C NMR (126 MHz, Chloroform-*d*) δ 148.5, 148.4, 138.4, 138.2, 131.6, 131.6, 128.9, 128.9, 128.2, 127.4, 125.1, 125.1, 124.9, 124.4, 124.3, 93.6, 92.6, 83.3, 83.3, 82.5, 82.2, 41.6, 40.9, 40.5, 39.3, 34.4, 33.9, 32.7, 31.5, 25.1, 25.0, 24.9, 24.8, 24.7, 17.3, 15.0.
^{11}B NMR (160 MHz, Chloroform-*d*) δ 34.36.
HRMS (ESI) m/z: $[M+H]^+$ Calcd. for $C_{28}H_{37}BO_2$ 431.3116; Found: 431.3115.

2-(4,4-dimethyl-2-(phenylethynyl)pentyl)-5,5-dimethyl-1,3,2-dioxaborinane (3t)

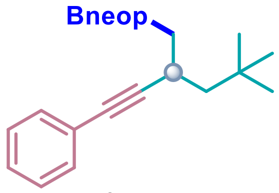

3t, 66%

Following the **general procedure 5** on 0.2 mmol scale, colorless oil, yield: 66% (41.2 mg), R_f = 0.4 (silica gel, PE: EA = 30:1, v/v), column chromatography (silica gel, PE: EA = 30:1, v/v).
^{1}H NMR (500 MHz, Chloroform-*d*) δ 7.38 – 7.34 (m, 2H), 7.28 – 7.22 (m, 3H), 3.63 (s, 4H), 2.87 – 2.78 (m, 1H), 1.65 (dd, *J* = 13.6, 10.2 Hz, 1H), 1.37 (dd, *J* = 13.7, 2.8 Hz, 1H), 1.13 (dd, *J* = 15.2, 8.3 Hz, 1H), 1.03 (s, 9H), 0.98 (s, 6H), 0.96 – 0.87 (m, 1H).

^{13}C NMR (126 MHz, Chloroform-*d*) δ 131.3, 128.1, 127.1, 124.7, 97.3, 80.6, 72.1, 51.9, 31.7, 31.2, 30.0, 24.0, 21.9.
^{11}B NMR (160 MHz, Chloroform-*d*) δ 33.51.
HRMS (ESI) m/z: $[M+H]^+$ Calcd. for $C_{28}H_{37}BO_2$ 313.2333; Found: 313.2329.

(E)-2-(2-(4-methoxystyryl)-4,4-dimethylpentyl)-4,4,5,5-tetramethyl-1,3,2-dioxaborolane (3u)

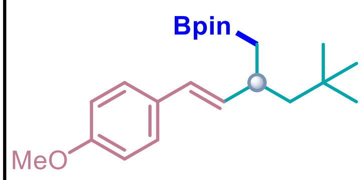

3u, 51%

Following the **general procedure 5** on 0.2 mmol scale, colorless oil, yield: 51% (36.5 mg), R_f = 0.4 (silica gel, PE: EA = 30:1, v/v), column chromatography (silica gel, PE: EA = 30:1, v/v).
^{1}H NMR (500 MHz, Chloroform-*d*) δ 7.33 (d, *J* = 8.6 Hz, 2H), 6.89 – 6.83 (m, 2H), 6.14 (d, *J* = 11.7 Hz, 1H), 5.45 (dd, *J* = 11.8, 10.6 Hz, 1H), 3.81 (s, 3H), 3.11 (dt, *J* = 11.1, 6.3 Hz, 1H), 1.31 – 1.29 (m, 2H), 1.22 (d, *J* = 4.3 Hz, 12H), 1.01 – 0.96 (m, 1H), 0.92 (s, 1H), 0.76 (s, 9H).
^{13}C NMR (126 MHz, Chloroform-*d*) δ 157.9, 140.3, 130.8, 129.9, 125.2, 113.5, 83.1, 55.2, 52.8, 31.2, 30.4, 30.8, 25.1, 24.7.
^{11}B NMR (160 MHz, Chloroform-*d*) δ 33.89.
HRMS (ESI) m/z: $[M+H]^+$ Calcd. for $C_{22}H_{35}BO_3$ 359.2752; Found: 359.2750.

(E)-2-(2-(4-methoxystyryl)-4-methylpentyl)-4,4,5,5-tetramethyl-1,3,2-dioxaborolane (3v)

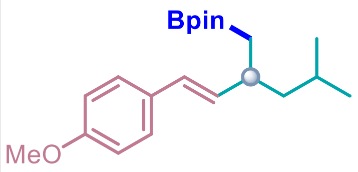

3v, 53%

Following the **general procedure 5** on 0.2 mmol scale, colorless oil, yield: 53% (36.5 mg), R_f = 0.4 (silica gel, PE: EA = 30:1, v/v), column chromatography (silica gel, PE: EA = 30:1, v/v).
^{1}H NMR (500 MHz, Chloroform-*d*) δ 7.26 (dd, *J* = 6.1, 2.6 Hz, 2H), 6.85 – 6.79 (m, 2H), 6.30 (d, *J* = 15.8 Hz, 1H), 5.88 (dd, *J* = 15.8, 8.9 Hz, 1H), 3.79 (s, 3H), 2.50 (m, 1H), 1.58 (m, 1H), 1.34 – 1.27 (m, 2H), 1.20 (d, *J* = 3.3 Hz, 13H), 0.95 (dd, *J* = 15.1, 6.1 Hz, 1H), 0.86 (dd, *J* = 6.6, 4.9 Hz, 6H).
^{13}C NMR (126 MHz, Chloroform-*d*) δ 158.5, 134.70 130.8, 127.7, 127.1, 113.8, 83.0, 55.3, 47.6, 37.1, 25.7, 25.0, 24.8, 23.6, 22.0.
^{11}B NMR (160 MHz, Chloroform-*d*) δ 33.91.
HRMS (ESI) m/z: $[M+H]^+$ Calcd. for $C_{21}H_{33}BO_3$ 345.2596; Found: 345.2590.

(E)-2-(2-cyclopentyl-4-(4-methoxyphenyl)but-3-en-1-yl)-4,4,5,5-tetramethyl-1,3,2-dioxaborolane(3w)

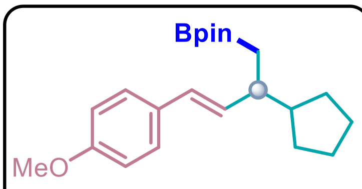

3w, 50%

Following the **general procedure 5** on 0.2 mmol scale, colorless oil, yield: 50% (35.6 mg), $R_f = 0.4$ (silica gel, PE: EA = 30:1, v/v), column chromatography (silica gel, PE: EA = 30:1, v/v).

^{1}H NMR (500 MHz, Chloroform-*d*) δ 7.25 (d, *J* = 8.3 Hz, 2H), 6.85 – 6.78 (m, 2H), 6.29 (d, *J* = 15.8 Hz, 1H), 5.96 (dd, *J* = 15.8, 8.9 Hz, 1H), 3.79 (s, 3H), 2.31 – 2.21 (m, 1H), 1.75 (m 2H), 1.68 – 1.62 (m, 1H), 1.59 – 1.46 (m, 4H), 1.17 (s, 14H), 1.08 (dd, *J* = 15.1, 4.6 Hz, 1H), 0.88 (dd, *J* = 15.1, 10.3 Hz, 1H).

^{13}C NMR (126 MHz, Chloroform-*d*) δ 158.5 133.6 130.9, 128.1 127.1 113.8, 830 55.3 47.1, 44.5 30.8 30.49, 25.5 25.4, 25.1 24.8, 24.7.

^{11}B NMR (160 MHz, Chloroform-*d*) δ 34.16.

HRMS (ESI) m/z: $[M+H]^+$ Calcd. for $C_{22}H_{33}BO_3$ 357.2596; Found: 357.2595.

(E)-2-(3-ethyl-2-(4-methoxystyryl)pentyl)-4,4,5,5-tetramethyl-1,3,2-dioxaborolane (3x)

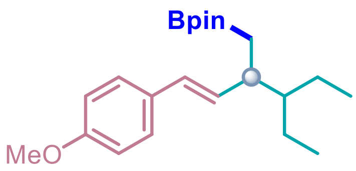

3x, 56%

Following the **general procedure 5** on 0.2 mmol scale, colorless oil, yield: 56% (40.1 mg), $R_f = 0.4$ (silica gel, PE: EA = 30:1, v/v), column chromatography (silica gel, PE: EA = 30:1, v/v).

^{1}H NMR (500 MHz, Chloroform-*d*) δ 7.26 – 7.24 (m, 2H), 6.86 – 6.78 (m, 2H), 6.28 (d, *J* = 15.8 Hz, 1H), 5.95 (dd, *J* = 15.8, 8.8 Hz, 1H), 3.79 (s, 3H), 2.53 (tt, *J* = 9.7, 5.0 Hz, 1H), 1.45 (dddd, *J* = 11.4, 9.5, 7.5, 4.1 Hz, 2H), 1.17 (s, 12H), 0.90 – 0.80 (m, 10H).

^{13}C NMR (126 MHz, Chloroform-*d*) δ 158.48, 132.79, 130.95, 128.51, 127.05, 113.81, 82.93, 55.28, 47.35, 40.75, 25.04, 24.90, 24.83, 24.80, 24.77, 23.00, 22.55, 12.14, 12.06, 11.75, 10.94.

^{11}B NMR (160 MHz, Chloroform-*d*) δ 33.96.

HRMS (ESI) m/z: $[M+H]^+$ Calcd. for $C_{22}H_{35}O_3$ 359.2752; Found: 359.2756.

(E)-2-(4-(4-methoxyphenyl)-2-phenethylbut-3-en-1-yl)-4,4,5,5-tetramethyl-1,3,2-dioxaborolane (3y)

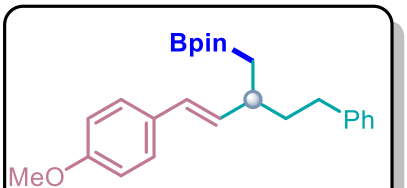

3y, 48%

Following the **general procedure 5** on 0.2 mmol scale, colorless oil, yield: 48% (37.6 mg), R_f = 0.4 (silica gel, PE: EA = 30:1, v/v), column chromatography (silica gel, PE: EA = 30:1, v/v).

^{1}H NMR (500 MHz, Chloroform-*d*) δ 7.30 – 7.25 (m, 4H), 7.20 – 7.16 (m, 3H), 6.89 – 6.81 (m, 2H), 6.35 (d, *J* = 15.7 Hz, 1H), 5.97 (dd, *J* = 15.8, 8.7 Hz, 1H), 3.81 (s, 3H), 2.70 – 2.62 (m, 1H), 2.62 – 2.54 (m, 1H), 2.49 (m, 1H), 1.85 – 1.75 (m, 1H), 1.69 (m, 1H), 1.20 (d, *J* = 1.9 Hz, 12H), 1.04 (dd, *J* = 15.2, 6.1 Hz, 1H), 0.96 (dd, *J* = 15.2, 8.6 Hz, 1H).

^{13}C NMR (126 MHz, Chloroform-*d*) δ 158.6, 142.9, 134.0, 130.7, 128.5, 128.4, 128.4, 128.3, 128.2, 127.1, 125.6, 113.9, 83.1, 55.3, 39.7, 39.0, 33.9, 25.0, 24.9, 24.8.

^{11}B NMR (160 MHz, Chloroform-*d*) δ 33.41.

HRMS (ESI) m/z: $[M+H]^+$ Calcd. for $C_{25}H_{33}BO_3$ 393.2596; Found: 393.2597.

(E)-2-(2-cyclohexyl-4-(4-methoxyphenyl)but-3-en-1-yl)-4,4,5,5-tetramethyl-1,3,2-dioxaborolane (3z)

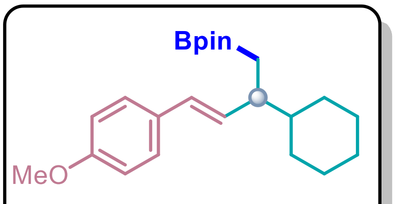

3z, 52%

Following the **general procedure 5** on 0.2 mmol scale, colorless oil, yield: 52% (38.5 mg), R_f = 0.4 (silica gel, PE: EA = 30:1, v/v), column chromatography (silica gel, PE: EA = 30:1, v/v).

^{1}H NMR (500 MHz, Chloroform-*d*) δ 7.26 (dd, *J* = 6.3, 2.5 Hz, 3H), 6.83 – 6.79 (m, 2H), 6.26 (d, *J* = 15.7 Hz, 1H), 5.94 (dd, *J* = 15.8, 9.0 Hz, 1H), 3.79 (s, 3H), 2.24 (m, 1H), 1.79 – 1.66 (m, 5H), 1.17 (s, 13H), 1.13 – 1.00 (m, 3H), 1.00 – 0.84 (m, 3H).

^{13}C NMR (126 MHz, Chloroform-*d*) δ 158.5, 133.0, 130.9, 128.6, 127.1, 113.8, 83.0, 55.3, 44.9, 44.1, 33.0, 30.9, 30.1, 26.7, 26.7, 25.0, 24.8, 24.7.

^{11}B NMR (160 MHz, Chloroform-*d*) δ 34.41.

HRMS (ESI) m/z: $[M+H]^+$ Calcd. for $C_{23}H_{35}BO_3$ 371.2752; Found: 371,2750.

ethyl 6,6-dimethyl-2-methylene-4-((4,4,5,5-tetramethyl-1,3,2-dioxaborolan-2-yl)methyl)heptanoate (5a)

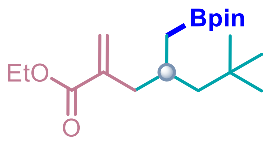

5a, 83%

Following the **general procedure 5** on 0.2 mmol scale, colorless oil, yield: 83% (56.1mg), R_f = 0.4 (silica gel, PE: EA = 30:1, v/v), column chromatography (silica gel, PE: EA = 30:1, v/v).

^{1}H NMR (500 MHz, Chloroform-*d*) δ 6.15 (d, *J* = 1.9 Hz, 1H), 5.49 (m, 1H), 4.18 (m, 2H), 2.47 (m, 1H), 2.07 (m, 1H), 1.90 (m, 1H), 1.28 (t, *J* = 7.2 Hz, 3H), 1.21 (d, *J* = 2.3 Hz, 12H), 1.18 (dd, *J* = 9.0, 5.1 Hz, 2H), 0.89 (s, 9H), 0.84 – 0.69 (m, 2H).

^{13}C NMR (126 MHz, Chloroform-*d*) δ 167.4, 140.1, 126.4, 82.7, 60.4, 50.7, 41.4, 31.1, 30.1, 29.3, 24.9, 24.7, 14.2.

^{11}B NMR (160 MHz, Chloroform-*d*) δ 33.78.

HRMS (ESI) m/z: $[M+H]^+$ Calcd. for $C_{19}H_{35}BO_4$ 339.2701; Found: 339.2701.

4,4,5,5-tetramethyl-2-(2-neopentyl-4-phenylpent-4-en-1-yl)-1,3,2-dioxaborolane (5b)

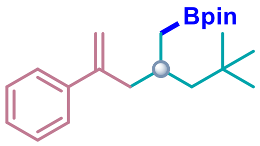

5b, 77%

Following the **general procedure 6** on 0.2 mmol scale, colorless oil, yield: 77% (52.7mg), R_f = 0.4 (silica gel, PE: EA = 30:1, v/v), column chromatography (silica gel, PE: EA = 30:1, v/v).

^{1}H NMR (500 MHz, Chloroform-*d*) δ 7.48 – 7.44 (m, 2H), 7.31 (dd, *J* = 8.3, 6.7 Hz, 2H), 7.25 (d, *J* = 6.8 Hz, 1H), 5.30 (d, *J* = 1.9 Hz, 1H), 5.05 (d, *J* = 1.7 Hz, 1H), 2.61 (m, 1H), 2.41 (dd, *J* = 13.9, 7.7 Hz, 1H), 1.73 (m, 1H), 1.36 – 1.33 (m, 1H), 1.26 (s, 12H), 1.11 (dd, *J* = 14.0, 6.4 Hz, 1H), 0.92 (dd, *J* = 15.9, 4.9 Hz, 1H), 0.81 (d, *J* = 7.6 Hz, 1H), 0.78 (s, 9H).

^{13}C NMR (126 MHz, Chloroform-*d*) δ 148.0, 141.2, 128.0, 127.1, 126.5, 114.1, 82.7, 49.9, 44.5, 30.9, 30.1, 28.8, 24.9, 24.9.

^{11}B NMR (160 MHz, Chloroform-*d*) δ 34.01.

HRMS (ESI) m/z: $[M+H]^+$ Calcd. for $C_{22}H_{35}BO_2$ 343.2803; Found: 343.2810.

4,4,5,5-tetramethyl-2-(2-neopentyl-4-(p-tolyl)pent-4-en-1-yl)-1,3,2-dioxaborolane (5c)

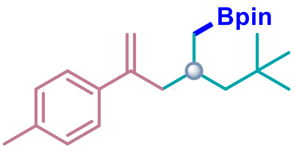

5c, 71%

Following the **general procedure 6** on 0.2 mmol scale, colorless oil, yield: 71% (50.7mg), R_f = 0.4 (silica gel, PE: EA = 30:1, v/v), column chromatography (silica gel, PE: EA = 30:1, v/v).

^{1}H NMR (500 MHz, Chloroform-*d*) δ 7.38 – 7.33 (m, 2H), 7.11 (d, *J* = 7.9 Hz, 2H), 5.27 (d, *J* = 2.0 Hz, 1H), 4.99 (d, *J* = 1.7 Hz, 1H), 2.58 (m, 1H), 2.41 – 2.33 (m, 4H), 1.73 (m, 1H), 1.34 (dd, *J* = 13.9, 3.7 Hz, 1H), 1.26 (s, 13H), 1.10 (dd, *J* = 14.0, 6.4 Hz, 1H), 0.92 (dd, *J* = 15.9, 4.9 Hz, 1H), 0.79 (s, 9H).

^{13}C NMR (126 MHz, Chloroform-*d*) δ 147.8, 138.3, 136.7, 128.7, 126.4, 113.3, 82.7, 49.9, 44.5, 31.0, 30.2, 28.8, 24.9, 24.9, 21.1.

^{11}B NMR (160 MHz, Chloroform-*d*) δ 34.01.

HRMS (ESI) m/z: $[M+H]^+$ Calcd. for $C_{23}H_{37}BO_2$ 357.2959; Found: 357.2960.

2-(4-(4-fluorophenyl)-2-neopentylpent-4-en-1-yl)-4,4,5,5-tetramethyl-1,3,2-dioxaborolane (5d)

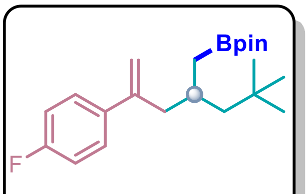

5d, 69%

Following the **general procedure 6** on 0.2 mmol scale, colorless oil, yield: 69% (49.7 mg), R_f = 0.4 (silica gel, PE: EA = 30:1, v/v), column chromatography (silica gel, PE: EA = 30:1, v/v).

^{1}H NMR (500 MHz, Chloroform-*d*) δ 7.42 (m, 2H), 6.98 (t, *J* = 8.7 Hz, 2H), 5.23 (d, *J* = 1.7 Hz, 1H), 5.01 (d, *J* = 1.6 Hz, 1H), 2.58 (m, 1H), 2.33 (dd, *J* = 13.9, 7.9 Hz, 1H), 1.71 – 1.64 (m, 1H), 1.32 (dd, *J* = 14.2, 3.7 Hz, 2H), 1.25 (s, 12H), 1.08 (dd, *J* = 14.0, 6.5 Hz, 1H), 0.91 (dd, *J* = 16.1, 4.8 Hz, 1H), 0.76 (s, 9H).

^{13}C NMR (126 MHz, Chloroform-*d*) δ 163.1, 161.2, 147.0, 137.2, 137.2, 128.1, 128.1, 114.9, 114.7, 114.0, 82.8, 82.8, 49.8, 47.4, 44.6, 30.9, 30.1, 29.4, 28.6, 24.9, 24.9, 24.8, 19.0.

^{11}B NMR (160 MHz, Chloroform-*d*) δ 34.04.

^{19}F NMR (471 MHz, Chloroform-*d*) δ -115.84.

HRMS (ESI) m/z: $[M+H]^+$ Calcd. for $C_{22}H_{34}BFO_2$ 361.2709; Found: 361.2707.

2-(4-(4-chlorophenyl)-2-neopentylpent-4-en-1-yl)-4,4,5,5-tetramethyl-1,3,2-dioxaborolane (5e)

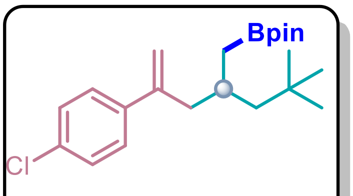

5e, 68%

Following the **general procedure 6** on 0.2 mmol scale, colorless oil, yield: 68% (51.1 mg), R_f = 0.4 (silica gel, PE: EA = 30:1, v/v), column chromatography (silica gel, PE: EA = 30:1, v/v).

^{1}H NMR (500 MHz, Chloroform-*d*) δ 7.40 (d, *J* = 8.6 Hz, 2H), 7.28 – 7.25 (m, 2H), 5.28 (d, *J* = 1.7 Hz, 1H), 5.04 (d, *J* = 1.5 Hz, 1H), 2.58 (m, 1H), 2.32 (dd, *J* = 13.9, 7.9 Hz, 1H), 1.71 – 1.64 (m, 1H), 1.31 (dd, *J* = 14.0, 3.5 Hz, 2H), 1.25 (s, 12H), 1.08 (dd, *J* = 14.0, 6.5 Hz, 1H), 0.91 (dd, *J* = 16.1, 4.8 Hz, 2H), 0.76 (s, 9H).

^{13}C NMR (126 MHz, Chloroform-*d*) δ 146.8, 139.6, 132.8, 128.2, 127.9, 114.6, 82.8, 49.8, 44.4, 30.9, 30.1, 29.4, 28.7, 24.9, 24.8.

^{11}B NMR (160 MHz, Chloroform-*d*) δ 34.00.

HRMS (ESI) m/z: $[M+H]^+$ Calcd. for $C_{22}H_{34}BClO_2$ 377.2413; Found: 377.2413.

2-(4-(4-bromophenyl)-2-neopentylpent-4-en-1-yl)-4,4,5,5-tetramethyl-1,3,2-dioxaborolane (5f)

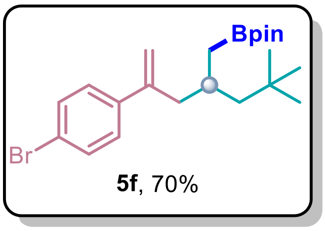

Following the **general procedure 6** on 0.2 mmol scale, colorless oil, yield: 70% (58.8 mg), R_f = 0.4 (silica gel, PE: EA = 30:1, v/v), column chromatography (silica gel, PE: EA = 30:1, v/v).

^{1}H NMR (500 MHz, Chloroform-*d*) δ 7.45 – 7.40 (m, 2H), 7.36 – 7.31 (m, 2H), 5.28 (d, *J* = 1.7 Hz, 1H), 5.05 (d, *J* = 1.6 Hz, 1H), 2.58 (m, 1H), 2.38 – 2.29 (m, 1H), 1.70 – 1.65 (m, 1H), 1.31 (dd, *J* = 14.0, 3.5 Hz, 2H), 1.25 (s, 12H), 1.08 (dd, *J* = 14.0, 6.5 Hz, 1H), 0.91 (dd, *J* = 16.0, 4.7 Hz, 1H), 0.76 (s, 9H).

^{13}C NMR (126 MHz, Chloroform-*d*) δ 146.9, 140.0, 131.1, 128.2, 121.0, 114.7, 82.8, 49.8, 44.3, 30.9, 30.1, 28.7, 24.9, 24.8.

^{11}B NMR (160 MHz, Chloroform-*d*) δ 34.12.

HRMS (ESI) m/z: $[M+H]^+$ Calcd. for $C_{22}H_{34}BBrO_2$ 421.1908; Found: 421.1911.

2-(4-(4-methoxyphenyl)-2-neopentylpent-4-en-1-yl)-4,4,5,5-tetramethyl-1,3,2-dioxaborolane (5g)

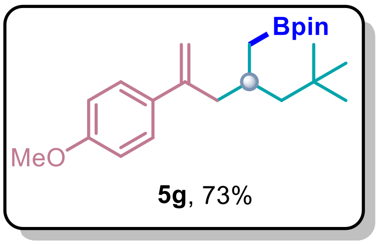

Following the **general procedure 6** on 0.2 mmol scale, colorless oil, yield: 73% (54.3 mg), R_f = 0.4 (silica gel, PE: EA = 30:1, v/v), column chromatography (silica gel, PE: EA = 30:1, v/v).

^{1}H NMR (500 MHz, Chloroform-*d*) δ 7.42 – 7.37 (m, 2H), 6.87 – 6.79 (m, 2H), 5.22 (d, *J* = 1.9 Hz, 1H), 4.95 (d, *J* = 1.9 Hz, 1H), 3.81 (s, 3H), 2.57 (dd, *J* = 13.8, 6.6 Hz, 1H), 2.35 (dd, *J* = 13.8, 7.8 Hz, 1H), 1.71 (m, 1H), 1.37 – 1.32 (m, 1H), 1.25 (d, *J* = 8.3 Hz, 12H), 1.09 (dd, *J* = 14.0, 6.4 Hz, 1H), 0.92 (dd, *J* = 15.9, 4.8 Hz, 1H), 0.77 (s, 9H), 0.66 (t, *J* = 7.4 Hz, 1H).

^{13}C NMR (126 MHz, Chloroform-*d*) δ 158.8, 147.2, 133.6, 127.6, 127.6, 113.4, 112.6, 82.7, 55.2, 49.8, 44.6, 30.9, 30.1, 28.7, 24.9, 24.9, 24.9, 24.8.

^{11}B NMR (160 MHz, Chloroform-*d*) δ 34.70.

HRMS (ESI) m/z: $[M+Na]^+$ Calcd. for $C_{23}H_{37}BO_3$ 395.2728; Found: 395.2726.

2-(4-(3,4-dimethoxyphenyl)-2-neopentylpent-4-en-1-yl)-4,4,5,5-tetramethyl-1,3,2-dioxaborolane (5h)

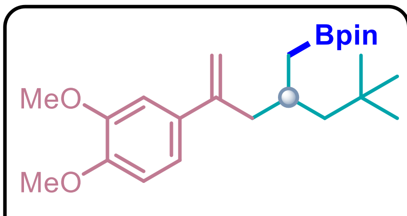

5h, 69%

Following the **general procedure 6** on 0.2 mmol scale, colorless oil, yield: 69% (55.5 mg), $R_f = 0.4$ (silica gel, PE: EA = 30:1, v/v), column chromatography (silica gel, PE: EA = 30:1, v/v).

^{1}H NMR (500 MHz, Chloroform-*d*) δ 7.01 (dd, *J* = 8.3, 2.1 Hz, 1H), 6.96 (d, *J* = 2.0 Hz, 1H), 6.81 (d, *J* = 8.3 Hz, 1H), 5.21 (d, *J* = 1.9 Hz, 1H), 4.97 (d, *J* = 1.8 Hz, 1H), 3.89 (d, *J* = 3.5 Hz, 6H), 2.53 (dd, *J* = 13.9, 7.3 Hz, 1H), 2.42 (td, *J* = 15.2, 13.9, 7.5 Hz, 1H), 1.78 – 1.69 (m, 1H), 1.35 – 1.28 (m, 2H), 1.24 (s, 12H), 1.10 (dd, *J* = 14.1, 6.1 Hz, 1H), 0.90 (dd, *J* = 15.9, 5.3 Hz, 1H), 0.78 (s, 9H).

^{13}C NMR (126 MHz, Chloroform-*d*) δ 148.5, 148.4, 147.6, 134.2, 118.9, 112.9, 110.7, 110.0, 82.7, 55.9, 55.8, 49.9, 44.6, 31.0, 30.2, 28.6, 24.9, 24.9.

^{11}B NMR (160 MHz, Chloroform-*d*) δ 33.82.

HRMS (ESI) m/z: $[M+H]^+$ Calcd. for $C_{24}H_{39}BO_4$ 403.3014; Found: 403.3010.

4,4,5,5-tetramethyl-2-(4-(naphthalen-2-yl)-2-neopentylpent-4-en-1-yl)-1,3,2-dioxaborolane (5i)

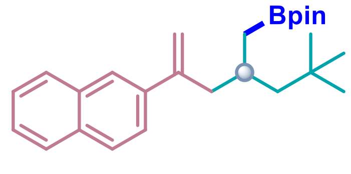

5i, 73%

Following the **general procedure 6** on 0.2 mmol scale, colorless oil, yield: 73% (57.2 mg), $R_f = 0.4$ (silica gel, PE: EA = 30:1, v/v), column chromatography (silica gel, PE: EA = 30:1, v/v).

^{1}H NMR (500 MHz, Chloroform-*d*) δ 7.93 (d, *J* = 1.8 Hz, 1H), 7.87 – 7.77 (m, 3H), 7.64 (d, *J* = 1.8 Hz, 1H), 7.46 (pd, *J* = 6.9, 1.6 Hz, 2H), 5.46 (d, *J* = 1.8 Hz, 1H), 5.17 (d, *J* = 1.7 Hz, 1H), 2.79 – 2.71 (m, 1H), 2.54 (dd, *J* = 13.9, 7.6 Hz, 1H), 1.82 (qt, *J* = 7.0, 3.0 Hz, 1H), 1.41 (dd, *J* = 14.0, 3.7 Hz, 1H), 1.29 (d, *J* = 1.7 Hz, 12H), 1.15 (dd, *J* = 14.0, 6.3 Hz, 1H), 0.98 (dd, *J* = 16.0, 5.0 Hz, 1H), 0.84 (dd, *J* = 16.0, 7.5 Hz, 2H), 0.79 (s, 9H).

^{13}C NMR (126 MHz, Chloroform-*d*) δ 147.81, 138.49, 133.41, 132.73, 128.13, 127.51, 127.47, 125.84, 125.55, 125.15, 125.12, 114.79, 82.76, 49.92, 44.53, 30.96, 30.15, 28.79, 24.96, 24.90, 24.80.

^{11}B NMR (160 MHz, Chloroform-*d*) δ 34.24.

HRMS (ESI) m/z: $[M+H]^+$ Calcd. for $C_{26}H_{37}BO_2$ 393.2959; Found: 393.2955.

4,4,5,5-tetramethyl-2-(2-neopentyl-4-(thiophen-2-yl)pent-4-en-1-yl)-1,3,2-dioxaborolane (5j)

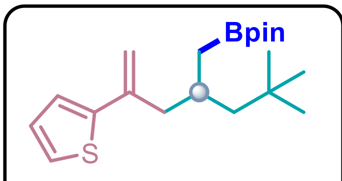

5j, 71%

Following the **general procedure 6** on 0.2 mmol scale, colorless oil, yield: 71% (49.4 mg), $R_f = 0.4$ (silica gel, PE: EA = 30:1, v/v), column chromatography (silica gel, PE: EA = 30:1, v/v).
^{1}H NMR (500 MHz, Chloroform-*d*) δ 7.19 (dd, *J* = 3.7, 1.1 Hz, 1H), 7.13 (dd, *J* = 5.1, 1.1 Hz, 1H), 6.96 (dd, *J* = 5.1, 3.6 Hz, 1H), 5.41 (d, *J* = 1.2 Hz, 1H), 4.93 (d, *J* = 1.3 Hz, 1H), 2.52 (m, 1H), 2.37 – 2.30 (m, 1H), 1.95 (m, 1H), 1.37 (dd, *J* = 13.9, 3.7 Hz, 1H), 1.26 (d, *J* = 1.8 Hz, 12H), 1.13 (dd, *J* = 14.0, 6.3 Hz, 1H), 0.98 – 0.93 (m, 1H), 0.83 (s, 10H).
^{13}C NMR (126 MHz, Chloroform-*d*) δ 145.6, 141.1, 127.2, 123.9, 123.9, 112.8, 82.9, 50.2, 45.0, 31.0, 30.1, 29.2, 25.0.
^{11}B NMR (160 MHz, Chloroform-*d*) δ 34.22.
HRMS (ESI) m/z: $[M+H]^+$ Calcd. for $C_{20}H_{33}BO_2S$ 349.2367; Found: 349.2368.

2-(4-chloro-2-neopentylpent-4-en-1-yl)-4,4,5,5-tetramethyl-1,3,2-dioxaborolane (5k)

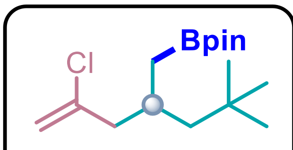

5k, 76%

Following the **general procedure 6** on 0.2 mmol scale, colorless oil, yield: 76% (45.6 mg), $R_f = 0.4$ (silica gel, PE: EA = 30:1, v/v), column chromatography (silica gel, PE: EA = 30:1, v/v).
^{1}H NMR (500 MHz, Chloroform-*d*) δ 5.14 (d, *J* = 1.0 Hz, 1H), 5.11 – 5.09 (m, 1H), 2.34 (m, 1H), 2.27 (dd, *J* = 14.0, 8.0 Hz, 1H), 2.04 (m, 1H), 1.23 (d, *J* = 1.4 Hz, 12H), 1.21 – 1.14 (m, 2H), 0.91 (d, *J* = 1.5 Hz, 9H), 0.88 – 0.85 (m, 2H).
^{13}C NMR (126 MHz, Chloroform-*d*) δ 142.6, 113.3, 82.8, 49.7, 48.0, 31.1, 30.1, 29.4, 28.4, 24.9, 24.8, 24.8.
^{11}B NMR (160 MHz, Chloroform-*d*) δ 33.81.
HRMS (ESI) m/z: $[M+H]^+$ Calcd. for $C_{16}H_{30}BClO_2$ 301.2100; Found: 301.2107.

adamantan-1-yl 6,6-dimethyl-2-methylene-4-((4,4,5,5-tetramethyl-1,3,2-dioxaborolan-2-yl)methyl)heptanoate (5l)

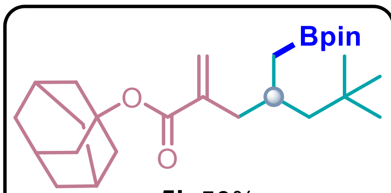

5l, 58%

Following the **general procedure 6** on 0.2 mmol scale, colorless oil, yield: 58% (51.5 mg), R_f = 0.4 (silica gel, PE: EA = 30:1, v/v), column chromatography (silica gel, PE: EA = 30:1, v/v).

^{1}H NMR (500 MHz, Chloroform-*d*) δ 6.20 (t, *J* = 1.8 Hz, 1H), 5.51 (t, *J* = 1.7 Hz, 1H), 4.98 (d, *J* = 3.2 Hz, 1H), 2.47 (m, 1H), 2.16 (dd, *J* = 13.5, 8.4 Hz, 1H), 2.04 (d, *J* = 10.6 Hz, 4H), 1.95 (m, 1H), 1.84 (m, 4H), 1.78 (d, *J* = 11.5 Hz, 2H), 1.73 (s, 2H), 1.59 – 1.53 (m, 2H), 1.22 (s, 12H), 0.89 (s, 9H), 0.86 – 0.79 (m, 2H).

^{13}C NMR (126 MHz, Chloroform-*d*) δ 166.7, 140.7, 126.0, 82.7, 50.4, 41.3, 37.4, 36.3, 36.3, 31.9, 31.9, 31.8, 31.1, 30.2, 29.5, 27.3, 27.0, 24.9, 24.8.

^{11}B NMR (160 MHz, Chloroform-*d*) δ 33.91.

HRMS (ESI) m/z: $[M+H]^+$ Calcd. for $C_{27}H_{45}BO_4$ 445.3484; Found: 445.3479.

cycloheptyl 6,6-dimethyl-2-methylene-4-((4,4,5,5-tetramethyl-1,3,2-dioxaborolan-2-yl)methyl)heptanoate (5m)

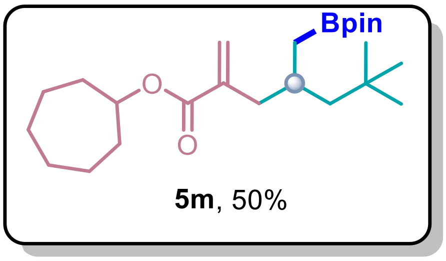

Following the **general procedure 6** on 0.2 mmol scale, colorless oil, yield: 50% (40.6 mg), R_f = 0.4 (silica gel, PE: EA = 30:1, v/v), column chromatography (silica gel, PE: EA = 30:1, v/v).

^{1}H NMR (500 MHz, Chloroform-*d*) δ 6.13 (d, *J* = 2.0 Hz, 1H), 5.46 (d, *J* = 1.9 Hz, 1H), 4.99 (m, 1H), 2.46 (m, 1H), 2.09 (dd, *J* = 13.4, 8.8 Hz, 1H), 1.98 – 1.86 (m, 3H), 1.73 – 1.63 (m, 4H), 1.59 – 1.53 (m, 4H), 1.50 – 1.40 (m, 2H), 1.22 (d, *J* = 2.1 Hz, 12H), 1.20 – 1.14 (m, 2H), 0.90 (s, 9H), 0.78 (m, 2H).

^{13}C NMR (126 MHz, Chloroform-*d*) δ 166.8, 140.7, 126.2, 82.8, 50.7, 41.5, 33.8, 33.8, 31.2, 30.2, 29.4, 28.3, 28.3, 25.0, 24.8, 23.0, 23.0.

^{11}B NMR (160 MHz, Chloroform-*d*) δ 34.01.

HRMS (ESI) m/z: $[M+H]^+$ Calcd. for $C_{24}H_{43}BO_4$ 407.3327; Found: 407.3319.

(E)-3,7-dimethylocta-2,6-dien-1-yl 6,6-dimethyl-2-methylene-4-((4,4,5,5-tetramethyl-1,3,2-dioxaborolan-2-yl)methyl)heptanoate (5n)

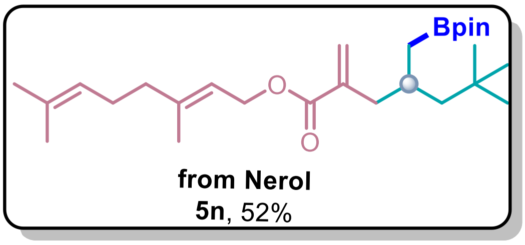

Following the **general procedure 6** on 0.2 mmol scale, colorless oil, yield: 52% (46.5 mg), R_f = 0.4 (silica gel, PE: EA = 30:1, v/v), column chromatography (silica gel, PE: EA = 30:1, v/v).

^{1}H NMR (500 MHz, Chloroform-*d*) δ 6.14 (d, *J* = 1.9 Hz, 1H), 5.50 (d, *J* = 1.8 Hz, 1H), 5.07 (m, 1H), 4.22 – 4.08 (m, 2H), 2.48 (m, 1H), 2.08 (dd, *J* = 13.5, 8.9 Hz, 1H), 2.02 – 1.85 (m, 3H), 1.75

– 1.66 (m, 4H), 1.61 – 1.53 (m, 4H), 1.51 – 1.45 (m, 1H), 1.38 – 1.32 (m, 1H), 1.24 – 1.16 (m, 15H), 0.91 (d, *J* = 10.6 Hz, 12H), 0.84 – 0.79 (m, 1H), 0.74 (m, 1H).

^{13}C NMR (126 MHz, Chloroform-*d*) δ 167.5, 140.2, 131.3, 126.4, 124.6, 82.8, 63.1, 50.7, 50.7, 41.4, 37.0, 35.5, 31.2, 30.2, 29.5, 29.4, 25.7, 25.4, 25.0, 24.8, 19.4, 19.4, 17.7.

^{11}B NMR (160 MHz, Chloroform-*d*) δ 34.10.

HRMS (ESI) m/z: $[M+H]^+$ Calcd. for $C_{27}H_{47}BO_4$ 447.3640; Found: 447.3637.

(1R,2S,5R)-2-isopropyl-5-methylcyclohexyl 6,6-dimethyl-2-methylene-4-((4,4,5,5-tetramethyl-1,3,2-dioxaborolan-2-yl)methyl)heptanoate (5o)

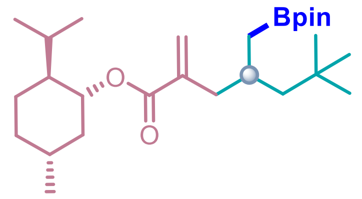

from DL-Menthol
5o, 65%

Following the **general procedure 6** on 0.2 mmol scale, colorless oil, yield: 65% (58.4 mg), R_f = 0.4 (silica gel, PE: EA = 30:1, v/v), column chromatography (silica gel, PE: EA = 30:1, v/v).

^{1}H NMR (500 MHz, Chloroform-*d*) δ 6.13 (dd, *J* = 9.8, 2.1 Hz, 1H), 5.47 (dd, *J* = 6.9, 1.9 Hz, 1H), 4.74 (m, 1H), 2.47 (m, 1H), 2.13 – 1.97 (m, 2H), 1.90 (m, 2H), 1.67 (m, 2H), 1.55 – 1.38 (m, 2H), 1.21 (d, *J* = 2.1 Hz, 15H), 1.10 – 0.97 (m, 2H), 0.91 – 0.86 (m, 15H), 0.84 – 0.78 (m, 1H), 0.77 – 0.70 (m, 4H).

^{13}C NMR (126 MHz, Chloroform-*d*) δ 167.0, 140.6, 140.5, 126.4, 126.14 82.7, 74.3, 50.6, 50.8, 47.1, 47.1, 41.5, 41.4, 40.9, 40.8, 34.4, 34.3, 31.42 31.4, 31.2, 31.1, 30.9, 30.2, 29.5, 26.3, 26.1, 25.0, 24.9, 24.9, 24.8, 23.5, 23.3, 22.1, 20.9, 20.8, 16.4, 16.2.

^{11}B NMR (160 MHz, Chloroform-*d*) δ 34.12.

HRMS (ESI) m/z: $[M+H]^+$ Calcd. for $C_{27}H_{49}BO_4$ 449.3797; Found: 449.3792.

3,7-dimethyloct-6-en-1-yl 6,6-dimethyl-2-methylene-4-((4,4,5,5-tetramethyl-1,3,2-dioxaborolan-2-yl)methyl)heptanoate (5p)

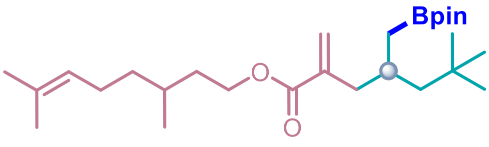

from Citronellol
5p, 57%

Following the **general procedure 6** on 0.2 mmol scale, colorless oil, yield: 75% (48.9mg), R_f = 0.4 (silica gel, PE: EA = 30:1, v/v), column chromatography (silica gel, PE: EA = 30:1, v/v).

^{1}H NMR (500 MHz, Chloroform-*d*) δ 6.15 (d, *J* = 2.0 Hz, 1H), 5.49 (d, *J* = 1.8 Hz, 1H), 5.38 (td, *J* = 7.2, 1.6 Hz, 1H), 5.12 – 5.05 (m, 1H), 4.62 (qdd, *J* = 12.6, 7.3, 1.1 Hz, 2H), 2.49 (ddd, *J* = 13.6, 5.3, 1.2 Hz, 1H), 2.14 – 2.03 (m, 5H), 1.94 – 1.87 (m, 1H), 1.75 (d, *J* = 1.4 Hz, 3H), 1.67 (d,

J = 1.5 Hz, 3H), 1.59 (d, J = 1.3 Hz, 3H), 1.22 (d, J = 2.3 Hz, 12H), 1.20 – 1.14 (m, 2H), 0.89 (s, 9H), 0.81 (dd, J = 15.7, 6.2 Hz, 1H), 0.73 (dd, J = 15.7, 6.3 Hz, 1H).

^{13}C NMR (126 MHz, Chloroform-*d*) δ 167.43, 142.08, 140.14, 132.09, 126.49, 123.65, 119.45, 82.77, 61.19, 50.71, 41.50, 32.19, 31.18, 30.11, 29.33, 26.66, 25.71, 24.97, 24.79, 23.49, 17.68.

^{11}B NMR (160 MHz, Chloroform-*d*) δ 33.90.

HRMS (ESI) m/z: $[M+H]^+$ Calcd. for $C_{27}H_{49}BO_4$ 449.3797; Found: 449.3795.

ethyl 4-cyclopentyl-2-methylene-5-(4,4,5,5-tetramethyl-1,3,2-dioxaborolan-2-yl)pentanoate (5q)

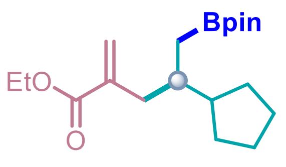

5q, 81%

Following the **general procedure 6** on 0.2 mmol scale, colorless oil, yield: 81% (54.5 mg), R_f = 0.4 (silica gel, PE: EA = 30:1, v/v), column chromatography (silica gel, PE: EA = 30:1, v/v).

^{1}H NMR (500 MHz, Chloroform-*d*) δ 6.12 (d, J = 1.9 Hz, 1H), 5.51 (d, J = 1.7 Hz, 1H), 4.21 – 4.11 (m, 2H), 2.54 (ddd, J = 13.7, 4.0, 1.3 Hz, 1H), 2.03 (dd, J = 13.6, 9.0 Hz, 1H), 1.79 – 1.64 (m, 4H), 1.59 – 1.51 (m, 2H), 1.46 (dtd, J = 15.3, 7.7, 7.2, 4.0 Hz, 2H), 1.27 (t, J = 7.1 Hz, 3H), 1.19 (s, 14H), 0.75 – 0.68 (m, 2H).

^{13}C NMR (126 MHz, Chloroform-*d*) δ 167.43, 140.08, 126.03, 82.75, 60.42, 46.09, 38.13, 38.12, 30.44, 30.18, 25.54, 25.49, 24.90, 24.84, 24.71, 14.19.

^{11}B NMR (160 MHz, Chloroform-*d*) δ 34.08.

HRMS (ESI) m/z: $[M+H]^+$ Calcd. for $C_{19}H_{33}BO_4$ 337.2545; Found: 337.2543.

ethyl 4-cyclohexyl-2-methylene-5-(4,4,5,5-tetramethyl-1,3,2-dioxaborolan-2-yl)pentanoate (5r)

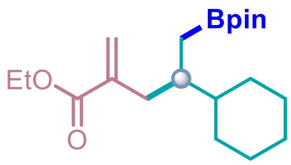

5r, 80%

Following the **general procedure 6** on 0.2 mmol scale, colorless oil, yield: 80% (56.0 mg), R_f = 0.4 (silica gel, PE: EA = 30:1, v/v), column chromatography (silica gel, PE: EA = 30:1, v/v).

^{1}H NMR (500 MHz, Chloroform-*d*) δ 6.14 (d, J = 1.8 Hz, 1H), 5.49 (q, J = 1.3 Hz, 1H), 4.23 – 4.12 (m, 2H), 2.50 (ddd, J = 13.6, 5.2, 1.3 Hz, 1H), 1.99 (dd, J = 13.6, 9.0 Hz, 1H), 1.75 – 1.69 (m, 3H), 1.62 (tt, J = 7.9, 4.2 Hz, 3H), 1.28 (t, J = 7.1 Hz, 3H), 1.21 (s, 15H), 1.12 – 0.98 (m, 3H), 0.70 (dd, J = 7.0, 3.5 Hz, 2H).

^{13}C NMR (126 MHz, Chloroform-*d*) δ 167.41, 140.31, 125.92, 82.77, 60.42, 42.54, 38.39, 36.20, 29.73, 29.38, 26.84, 26.79, 24.85, 24.68, 14.17.
^{11}B NMR (160 MHz, Chloroform-*d*) δ 34.48.
HRMS (ESI) m/z: $[M+H]^+$ Calcd. for $C_{20}H_{35}BO_4$ 351.2701; Found: 351.2702.

ethyl 5-ethyl-2-methylene-4-((4,4,5,5-tetramethyl-1,3,2-dioxaborolan-2-yl)methyl)heptanoate (5s)

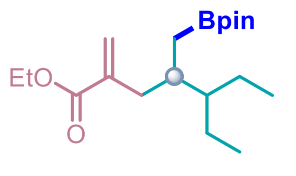

5s, 79%

Following the **general procedure 6** on 0.2 mmol scale, colorless oil, yield: 79% (53.4 mg), R_f = 0.4 (silica gel, PE: EA = 30:1, v/v), column chromatography (silica gel, PE: EA = 30:1, v/v).
^{1}H NMR (500 MHz, Chloroform-*d*) δ 6.12 (d, *J* = 1.8 Hz, 1H), 5.46 (d, *J* = 1.8 Hz, 1H), 4.16 (m, 2H), 2.46 – 2.38 (m, 1H), 2.03 – 1.93 (m, 2H), 1.28 (m, 5H), 1.22 – 1.11 (m, 15H), 1.03 (m, 1H), 0.85 (m, 6H), 0.65 (d, *J* = 6.6 Hz, 2H).
^{13}C NMR (126 MHz, Chloroform-*d*) δ 167.4, 140.3, 140.3, 126.0, 82.8, 60.4, 46.1, 35.8, 34.1, 34.1, 25.0, 24.7, 22.8, 22.5, 14.2, 12.6, 12.5.
^{11}B NMR (160 MHz, Chloroform-*d*) δ 34.05.
HRMS (ESI) m/z: $[M+H]^+$ Calcd. for $C_{19}H_{35}BO_4$ 339.2701; Found: 339.2768.

ethyl 5-methyl-2-methylene-4-((4,4,5,5-tetramethyl-1,3,2-dioxaborolan-2-yl)methyl)octanoate (5t)

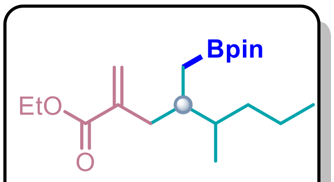

5t, 78%

Following the **general procedure 6** on 0.2 mmol scale, colorless oil, yield: 78% (52.7 mg), R_f = 0.4 (silica gel, PE: EA = 30:1, v/v), column chromatography (silica gel, PE: EA = 30:1, v/v).
^{1}H NMR (500 MHz, Chloroform-*d*) δ 6.14 (dd, *J* = 4.3, 1.8 Hz, 1H), 5.49 (dd, *J* = 3.6, 2.1 Hz, 1H), 4.17 (m, 2H), 2.38 (m, 1H), 2.08 (m, 1H), 1.91 – 1.80 (m, 1H), 1.46 (m, 1H), 1.38 – 1.32 (m, 1H), 1.28 (t, *J* = 7.1 Hz, 4H), 1.20 (d, *J* = 5.5 Hz, 13H), 1.15 – 1.01 (m, 1H), 0.86 (m, 3H), 0.79 (t, *J* = 6.5 Hz, 3H), 0.73 – 0.66 (m, 1H), 0.59 (dd, *J* = 15.6, 8.1 Hz, 1H).
^{13}C NMR (126 MHz, Chloroform-*d*) δ 167.4, 167.4, 140.2, 126.1, 125.9, 82.8, 82.8, 60.5, 60.4, 37.4, 37.4, 36.9, 36.8, 36.3, 36.2, 36.0, 34.9, 25.0, 24.9, 24.8, 24.7, 20.8, 20.7, 15.3, 15.0, 14.4, 14.2, 14.2.
^{11}B NMR (160 MHz, Chloroform-*d*) δ 34.16.

HRMS (ESI) m/z: $[M+H]^+$ Calcd. for $C_{19}H_{35}BO_4$ 339.2701; Found: 339.2700.

ethyl 4-(cyclohex-2-en-1-yl)-2-methylene-5-(4,4,5,5-tetramethyl-1,3,2-dioxaborolan-2-yl)pentanoate (5u)

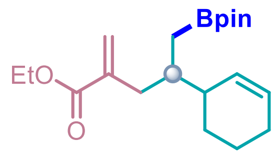

5u, 76%

Following the **general procedure 6** on 0.2 mmol scale, colorless oil, yield: 76% (52.9 mg), R_f = 0.4 (silica gel, PE: EA = 30:1, v/v), column chromatography (silica gel, PE: EA = 30:1, v/v).
^{1}H NMR (500 MHz, Chloroform-*d*) δ 6.15 (d, *J* = 1.8 Hz, 1H), 5.68 – 5.59 (m, 2H), 5.51 (m, 1H), 4.22 – 4.11 (m, 2H), 2.57 – 2.47 (m, 1H), 2.07 – 1.96 (m, 3H), 1.87 – 1.77 (m, 2H), 1.70 (m, 1H), 1.61 – 1.51 (m, 1H), 1.28 (t, *J* = 7.1 Hz, 4H), 1.20 (s, 13H), 0.73 (d, *J* = 1.8 Hz, 2H).
^{13}C NMR (126 MHz, Chloroform-*d*) δ 167.3, 140.2, 140.1, 127.1, 127.1, 127.0, 126.9, 126.2, 126.1, 82.9, 60.5, 38.4, 38.2, 37.9, 37.6, 36.4, 36.0, 28.8, 28.0, 26.1, 25.9, 25.5, 24.9, 24.7, 14.21.
^{11}B NMR (160 MHz, Chloroform-*d*) δ 34.44.
HRMS (ESI) m/z: $[M+H]^+$ Calcd. for $C_{20}H_{33}BO_4$ 349.2545; Found: 349.2543.

ethyl 4-(3,6-dimethylcyclohex-3-en-1-yl)-2-methylene-5-(4,4,5,5-tetramethyl-1,3,2-dioxaborolan-2-yl)pentanoate (5v)

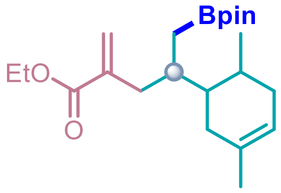

5v, 70%

Following the **general procedure 6** on 0.2 mmol scale, colorless oil, yield: 70% (52.6 mg), R_f = 0.4 (silica gel, PE: EA = 30:1, v/v), column chromatography (silica gel, PE: EA = 30:1, v/v).
^{1}H NMR (500 MHz, Chloroform-*d*) δ 6.15 (dd, *J* = 8.1, 1.9 Hz, 1H), 5.54 (s, 1H), 5.38 – 5.29 (m, 1H), 4.16 (m, 2H), 2.72 (m, 1H), 2.27 (m, 1H), 2.03 – 1.85 (m, 3H), 1.82 – 1.69 (m, 1H), 1.59 (d, *J* = 3.5 Hz, 4H), 1.35 (m, 1H), 1.31 – 1.25 (m, 4H), 1.18 (d, *J* = 4.7 Hz, 13H), 0.90 (d, *J* = 6.9 Hz, 2H), 0.82 (d, *J* = 6.9 Hz, 1H), 0.75 (t, *J* = 5.1 Hz, 1H).
^{13}C NMR (126 MHz, Chloroform-*d*) δ 167.4, 167.4, 140.3, 140.2, 132.5, 132.4, 128.3, 128.3, 126.6, 126.3, 82.7, 82.7, 60.5, 60.4, 42.7, 42.3, 36.7, 36.5, 34.5, 33.9, 31.7, 31.6, 31.2, 25.0, 24.9, 24.7, 24.6, 23.4, 21.4, 20.8, 15.1, 14.9, 14.2, 14.2.
^{11}B NMR (160 MHz, Chloroform-*d*) δ 34.32.
HRMS (ESI) m/z: $[M+H]^+$ Calcd. for $C_{22}H_{37}BO_4$ 377.2858; Found: 377.2853.

ethyl 2-methylene-6-phenyl-4-((4,4,5,5-tetramethyl-1,3,2-dioxaborolan-2-yl)methyl)hexanoate (5w)

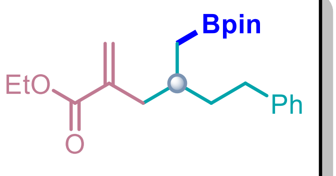

5w, 77%

Following the **general procedure 6** on 0.2 mmol scale, colorless oil, yield: 77% (57.2 mg), R_f = 0.4 (silica gel, PE: EA = 30:1, v/v), column chromatography (silica gel, PE: EA = 30:1, v/v).

^{1}H NMR (500 MHz, Chloroform-*d*) δ 7.26 (t, *J* = 7.5 Hz, 2H), 7.20 – 7.14 (m, 3H), 6.18 (d, *J* = 1.8 Hz, 1H), 5.52 (d, *J* = 1.7 Hz, 1H), 4.19 (m, 2H), 2.63 (m, 2H), 2.48 (dd, *J* = 13.6, 6.1 Hz, 1H), 2.20 (dd, *J* = 13.7, 7.9 Hz, 1H), 1.93 (m, 1H), 1.60 (m, 2H), 1.28 (d, *J* = 4.4 Hz, 3H), 1.24 (d, *J* = 1.9 Hz, 12H), 0.84 (m, 2H).

^{13}C NMR (126 MHz, Chloroform-*d*) δ 167.4, 143.1, 139.7, 128.4, 128.4, 128.2, 126.2, 125.5, 83.0, 60.6, 39.2, 38.4, 33.3, 33.2, 25.0, 24.8, 14.2.

^{11}B NMR (160 MHz, Chloroform-*d*) δ 34.41.

HRMS (ESI) m/z: $[M+H]^+$ Calcd. for $C_{22}H_{33}BO_4$ 373.2545; Found: 373.2546.

ethyl 6-methyl-2-methylene-4-((4,4,5,5-tetramethyl-1,3,2-dioxaborolan-2-yl)methyl)heptanoate (5x)

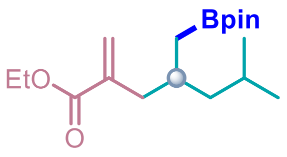

5x, 80%

Following the **general procedure 6** on 0.2 mmol scale, colorless oil, yield: 80% (51.8 mg), R_f = 0.4 (silica gel, PE: EA = 30:1, v/v), column chromatography (silica gel, PE: EA = 30:1, v/v).

^{1}H NMR (500 MHz, Chloroform-*d*) δ 6.14 (d, *J* = 1.9 Hz, 1H), 5.48 (s, 1H), 4.17 (m, 2H), 2.42 (dd, *J* = 13.5, 5.2 Hz, 1H), 2.02 (dd, *J* = 13.5, 8.6 Hz, 1H), 1.93 – 1.83 (m, 1H), 1.66 (m, 1H), 1.27 (t, *J* = 7.1 Hz, 3H), 1.20 (d, *J* = 2.9 Hz, 12H), 1.09 (m, 2H), 0.84 (dd, *J* = 20.5, 6.6 Hz, 6H), 0.75 (dd, *J* = 15.5, 5.9 Hz, 1H), 0.63 (dd, *J* = 15.5, 6.9 Hz, 1H).

^{13}C NMR (126 MHz, Chloroform-*d*) δ 167.5, 139.9, 126.1, 82.8, 60.4, 46.5, 39.5, 30.9, 25.3, 25.0, 24.8, 24.7, 22.9, 22.8, 14.2.

^{11}B NMR (160 MHz, Chloroform-*d*) δ 34.04.

HRMS (ESI) m/z: $[M+H]^+$ Calcd. for $C_{18}H_{33}BO_4$ 325.2545; Found: 325.2545.

ethyl 2-methylene-4-((4,4,5,5-tetramethyl-1,3,2-dioxaborolan-2-yl)methyl)decanoate (5y)

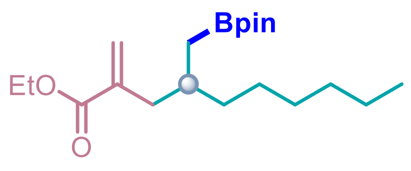

5y, 76%

Following the **general procedure 6** on 0.2 mmol scale, colorless oil, yield: 76% (53.5 mg), R_f = 0.4 (silica gel, PE: EA = 30:1, v/v), column chromatography (silica gel, PE: EA = 30:1, v/v).
^{1}H NMR (500 MHz, Chloroform-*d*) δ 6.15 (d, *J* = 1.8 Hz, 1H), 5.49 (d, *J* = 1.8 Hz, 1H), 4.18 (m, 2H), 2.40 (m, 1H), 2.09 (dd, *J* = 13.6, 8.0 Hz, 1H), 1.84 – 1.75 (m, 1H), 1.29 – 1.24 (m, 13H), 1.22 (d, *J* = 1.6 Hz, 12H), 0.86 (t, *J* = 6.8 Hz, 3H), 0.77 (dd, *J* = 15.5, 6.1 Hz, 1H), 0.69 (dd, *J* = 15.6, 7.3 Hz, 1H).
^{13}C NMR (126 MHz, Chloroform-*d*) δ 167.5, 140.0, 126.0, 82.8, 60.5, 39.3, 36.5 33.3, 31.9, 29.6, 26.8, 24.9, 24.8, 24.8, 24.7, 22.7, 14.2, 14.1.
^{11}B NMR (160 MHz, Chloroform-*d*) δ 34.33.
HRMS (ESI) m/z: $[M+H]^+$ Calcd. for $C_{20}H_{37}BO_4^+$ 353.2858; Found: 353.2855.

ethyl 2-methylene-4-((4,4,5,5-tetramethyl-1,3,2-dioxaborolan-2-yl)methyl)heptanoate (5z)

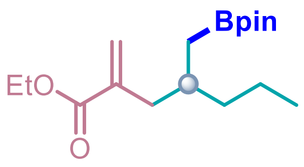

5z, 78%

Following the **general procedure 6** on 0.2 mmol scale, colorless oil, yield: 78% (48.4 mg), R_f = 0.4 (silica gel, PE: EA = 30:1, v/v), column chromatography (silica gel, PE: EA = 30:1, v/v).
^{1}H NMR (500 MHz, Chloroform-*d*) δ 6.14 (d, *J* = 1.8 Hz, 1H), 5.48 (d, *J* = 1.9 Hz, 1H), 4.17 (m, 2H), 2.40 (m, 1H), 2.09 (dd, *J* = 13.6, 8.1 Hz, 1H), 1.80 (m, 1H), 1.28 (t, *J* = 7.1 Hz, 7H), 1.21 (d, *J* = 1.6 Hz, 12H), 0.85 (t, *J* = 7.1 Hz, 3H), 0.76 (dd, *J* = 15.6, 6.2 Hz, 1H), 0.68 (dd, *J* = 15.6, 7.3 Hz, 1H).
^{13}C NMR (126 MHz, Chloroform-*d*) δ 167.4, 140.0, 125.9, 82.8, 60.4, 39.2, 36.4, 33.2, 32.1, 32.1, 26.4, 24.9, 24.8, 24.8, 24.7, 22.6, 14.2, 14.1.
^{11}B NMR (160 MHz, Chloroform-*d*) δ 33.95.
HRMS (ESI) m/z: $[M+H]^+$ Calcd. for $C_{17}H_{31}BO_4$ 311.2388; Found: 311.2387.

ethyl 6,10-dimethyl-2-methylene-4-((4,4,5,5-tetramethyl-1,3,2-dioxaborolan-2-yl)methyl)undec-9-enoate (5aa)

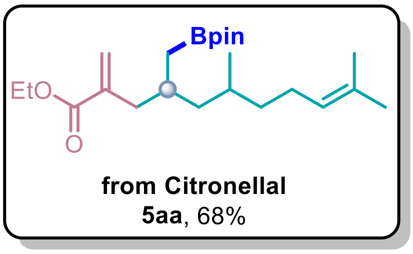

Following the **general procedure 6** on 0.2 mmol scale, colorless oil, yield: 68% (53.3 mg), R_f = 0.4 (silica gel, PE: EA = 30:1, v/v), column chromatography (silica gel, PE: EA = 30:1, v/v).

^{1}H NMR (500 MHz, Chloroform-*d*) δ 6.12 (t, *J* = 2.1 Hz, 1H), 5.46 (d, *J* = 2.2 Hz, 1H), 5.05 (m, 1H), 4.24 – 4.03 (m, 2H), 2.33 (dd, *J* = 13.7, 5.9 Hz, 1H), 1.99 – 1.83 (m, 4H), 1.63 (s, 3H), 1.55 (s, 3H), 1.49 (m, 1H), 1.27 – 1.23 (m, 3H), 1.18 (d, *J* = 3.4 Hz, 15H), 1.11 – 0.99 (m, 2H), 0.86 – 0.78 (m, 3H), 0.74 – 0.65 (m, 1H).

^{13}C NMR (126 MHz, Chloroform-*d*) δ 167.4, 167.3, 139.9, 139.9, 130.8, 130.8, 126.2, 123.0, 125.1, 125.0, 82.7, 60.4, 60.4, 44.7, 44.4, 39.8, 39.1, 37.5, 37.5, 30.7, 30.7, 29.8, 29.8, 25.7, 25.4, 25.4, 25.0, 24.9, 24.9, 24.8, 24.8, 24.8, 24.7, 24.7, 19.7, 19.6, 17.6, 14.2, 14.2.

^{11}B NMR (160 MHz, Chloroform-*d*) δ 34.21.

HRMS (ESI) m/z: $[M+H]^+$ Calcd. for $C_{23}H_{41}BO_4$ 393.3171; Found: 393.3169.

ethyl 2-methylene-4-((4,4,5,5-tetramethyl-1,3,2-dioxaborolan-2-yl)methyl)pentadecanoate (5ab)

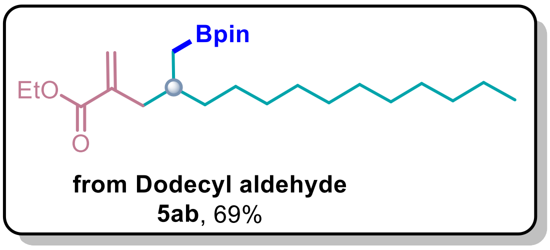

Following the **general procedure 6** on 0.2 mmol scale, colorless oil, yield: 69% (58.4 mg), R_f = 0.4 (silica gel, PE: EA = 30:1, v/v), column chromatography (silica gel, PE: EA = 30:1, v/v).

^{1}H NMR (500 MHz, Chloroform-*d*) δ 6.14 (d, *J* = 1.8 Hz, 1H), 5.48 (d, *J* = 1.8 Hz, 1H), 4.17 (m, 2H), 2.39 (dd, *J* = 13.6, 5.9 Hz, 1H), 2.08 (dd, *J* = 13.6, 8.1 Hz, 1H), 1.29 – 1.20 (m, 36H), 0.85 (t, *J* = 6.9 Hz, 3H), 0.79 – 0.63 (m, 2H).

^{13}C NMR (126 MHz, Chloroform-*d*) δ 140.0, 125.9, 82.9, 82.8, 39.3, 36.5, 33.3, 31.9, 29.9, 29.7, 29.7, 29.6, 29.4, 26.8, 24.9, 24.7, 22.7, 14.2, 14.1.

^{11}B NMR (160 MHz, Chloroform-*d*) δ 34.28.

HRMS (ESI) m/z: $[M+H]^+$ Calcd. for $C_{25}H_{47}BO_4$ 423.3640; Found: 423.3643.

5,5-dimethyl-2-(2-neopentyl-4-phenylpent-4-en-1-yl)-1,3,2-dioxaborinane (5ac)

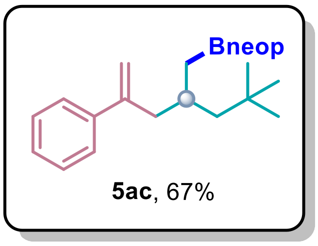

Following the **general procedure 6** on 0.2 mmol scale, colorless oil, yield: 67% (44.0 mg), R_f = 0.4 (silica gel, PE: EA = 30:1, v/v), column chromatography (silica gel, PE: EA = 30:1, v/v).

^{1}H NMR (500 MHz, Chloroform-*d*) δ 7.47 – 7.44 (m, 2H), 7.34 – 7.31 (m, 2H), 7.29 – 7.23 (m, 1H), 5.31 (t, *J* = 1.4 Hz, 1H), 5.06 (t, *J* = 1.5 Hz, 1H), 3.59 (d, *J* = 1.1 Hz, 4H), 2.60 – 2.55 (m, 1H), 2.42 (dd, *J* = 13.9, 7.5 Hz, 1H), 1.80 – 1.71 (m, 1H), 1.35 (dd, *J* = 13.9, 3.7 Hz, 1H), 1.13 (dd, *J* = 14.0, 6.1 Hz, 1H), 0.98 (d, *J* = 1.1 Hz, 6H), 0.89 – 0.85 (m, 1H), 0.81 (d, *J* = 1.3 Hz, 9H), 0.75 (dd, *J* = 15.8, 7.8 Hz, 1H).

^{13}C NMR (126 MHz, Chloroform-*d*) δ 148.3, 141.5, 128.0, 127.1, 126.6, 113.9, 71.8, 50.2, 45.0, 31.6, 31.1, 30.2 28.8, 22.0.

^{11}B NMR (160 MHz, Chloroform-*d*) δ 33.98.

HRMS (ESI) m/z: $[M+H]^+$ Calcd. for $C_{25}H_{47}BO_4$ 329.2646; Found: 329.2640.

ethyl 6,6-dimethyl-4-((4,4,5,5-tetramethyl-1,3,2-dioxaborolan-2-yl)methyl)heptanoate (7a)

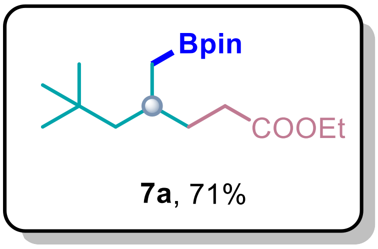

Following the **general procedure 7** on 0.2 mmol scale, colorless oil, yield: 71% (46.2 mg), R_f = 0.4 (silica gel, PE: EA = 30:1, v/v), column chromatography (silica gel, PE: EA = 30:1, v/v).

^{1}H NMR (500 MHz, Chloroform-*d*) δ 4.10 (m, 2H), 2.28 (m, 2H), 1.68 (m, 2H), 1.61 – 1.50 (m, 1H), 1.23 (d, *J* = 4.2 Hz, 15H), 1.20 – 1.09 (m, 2H), 0.88 (s, 9H), 0.84 – 0.76 (m, 2H).

^{13}C NMR (126 MHz, Chloroform-*d*) δ 174.2, 82.9, 60.1, 50.5, 33.3, 32.1, 31.1, 30.1, 30.1, 24.8, 24.8, 14.3.

^{11}B NMR (160 MHz, Chloroform-*d*) δ 33.86.

HRMS (ESI) m/z: $[M+H]^+$ Calcd. for $C_{18}H_{35}BO_4$ 327.2701; Found: 327.2707.

tert-butyl 6,6-dimethyl-4-((4,4,5,5-tetramethyl-1,3,2-dioxaborolan-2-yl)methyl)heptanoate (7b)

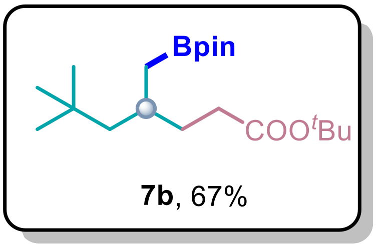

Following the **general procedure 7** on 0.2 mmol scale, colorless oil, yield: 67% (47.4 mg), $R_f = 0.4$ (silica gel, PE: EA = 30:1, v/v), column chromatography (silica gel, PE: EA = 30:1, v/v).

^{1}H NMR (500 MHz, Chloroform-*d*) δ 2.23 – 2.16 (m, 2H), 1.70 – 1.65 (m, 2H), 1.53 – 1.48 (m, 1H), 1.43 (s, 9H), 1.23 (s, 12H), 1.20 – 1.09 (m, 2H), 0.89 (s, 9H), 0.81 (dd, *J* = 7.5, 6.3 Hz, 2H).

^{13}C NMR (126 MHz, Chloroform-*d*) δ 173.6, 82.8, 50.5, 33.6, 33.4, 31.1, 30.1, 30.07, 28.1, 24.9, 24.8.

^{11}B NMR (160 MHz, Chloroform-*d*) δ 33.99.

HRMS (ESI) m/z: $[M+H]^+$ Calcd. for $C^{20}H^{39}BO^{4}$ 355.3014; Found: 355.3012.

benzyl 6,6-dimethyl-4-((4,4,5,5-tetramethyl-1,3,2-dioxaborolan-2-yl)methyl)heptanoate (4c)

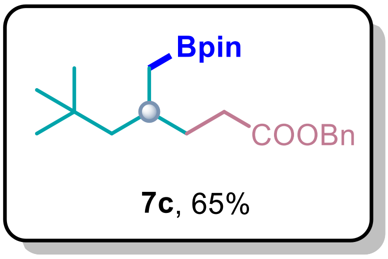

Following the **general procedure 7** on 0.2 mmol scale, colorless oil, yield: 65% (50.4 mg), $R_f = 0.4$ (silica gel, PE: EA = 30:1, v/v), column chromatography (silica gel, PE: EA = 30:1, v/v).

^{1}H NMR (500 MHz, Chloroform-*d*) δ 7.35 (d, *J* = 4.0 Hz, 4H), 7.33 – 7.29 (m, 1H), 5.10 (s, 2H), 2.36 (m, 2H), 1.72 – 1.66 (m, 1H), 1.60 (m, 1H), 1.22 (d, *J* = 1.5 Hz, 12H), 1.20 – 1.12 (m, 2H), 0.88 (d, *J* = 1.5 Hz, 9H), 0.82 (dd, *J* = 10.2, 6.2 Hz, 2H).

^{13}C NMR (126 MHz, Chloroform-*d*) δ 173.9, 136.1, 128.5, 128.1, 128.1, 82.9, 66.0, 50.4, 33.3, 32.0, 31.1, 30.1, 24.8, 24.8.

^{11}B NMR (160 MHz, Chloroform-*d*) δ 34.13.

HRMS (ESI) m/z: $[M+H]^+$ Calcd. for $C_{23}H_{37}BO_4$ 389.2858; Found: 389.2858.

phenyl 6,6-dimethyl-4-((4,4,5,5-tetramethyl-1,3,2-dioxaborolan-2-yl)methyl)heptanoate (7d)

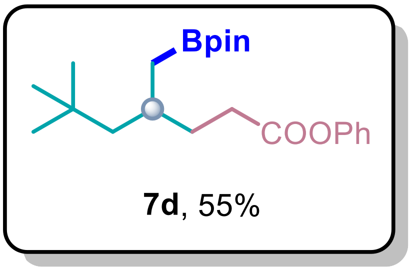

Following the **general procedure 7** on 0.2 mmol scale, colorless oil, yield: 55% (41.4 mg), $R_f = 0.4$ (silica gel, PE: EA = 30:1, v/v), column chromatography (silica gel, PE: EA = 30:1, v/v).

^{1}H NMR (500 MHz, Chloroform-*d*) δ 7.39 – 7.34 (m, 2H), 7.24 – 7.18 (m, 1H), 7.10 – 7.05 (m, 2H), 2.56 (m, 2H), 1.88 – 1.75 (m, 2H), 1.70 (m, 1H), 1.24 (s, 13H), 1.20 (dd, *J* = 14.1, 5.0 Hz, 1H), 0.93 (s, 9H), 0.91 – 0.85 (m, 2H).

^{13}C NMR (126 MHz, Chloroform-*d*) δ 172.6, 150.8, 129.3, 125.6, 121.6, 82.9, 50.5, 33.3, 32.1, 31.1, 30.1, 30.0, 24.9, 24.8.

^{11}B NMR (160 MHz, Chloroform-*d*) δ 33.93.

HRMS (ESI) m/z: $[M+H]^+$ Calcd. for $C_{22}H_{35}BO_4$ 375.2701; Found: 375.2698.

6,6-dimethyl-4-((4,4,5,5-tetramethyl-1,3,2-dioxaborolan-2-yl)methyl)heptanenitrile (7e)

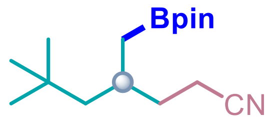

7e, 60%

Following the **general procedure 7** on 0.2 mmol scale, colorless oil, yield: 60% (33.5 mg), R_f = 0.4 (silica gel, PE: EA = 30:1, v/v), column chromatography (silica gel, PE: EA = 30:1, v/v).
^{1}H NMR (500 MHz, Chloroform-*d*) δ 2.32 (m, 2H), 1.78 – 1.69 (m, 2H), 1.66 – 1.58 (m, 1H), 1.23 (s, 12H), 1.17 (dd, *J* = 4.9, 2.9 Hz, 2H), 0.90 (s, 9H), 0.83 (dd, *J* = 8.3, 6.1 Hz, 2H).
^{13}C NMR (126 MHz, Chloroform-*d*) δ 120.2, 83.1, 50.2, 33.7, 31.1, 30.0, 29.9, 24.9, 24.8, 14.9.
^{11}B NMR (160 MHz, Chloroform-*d*) δ 33.79.
HRMS (ESI) m/z: $[M+H]^+$ Calcd. for $C_{16}H_{30}BNO_2$ 280.2442; Found: 280.2445.

ethyl (5,5-dimethyl-3-((4,4,5,5-tetramethyl-1,3,2-dioxaborolan-2-yl)methyl)hexyl)(oxo)-l4-phosphanecarboxylate (7f)

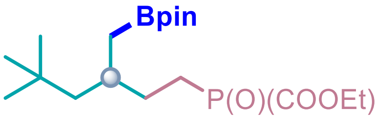

7f, 73%

Following the **general procedure 7** on 0.2 mmol scale, colorless oil, yield: 78% (48.4 mg), R_f = 0.4 (silica gel, PE: EA = 30:1, v/v), column chromatography (silica gel, PE: EA = 30:1, v/v).
^{1}H NMR (500 MHz, Chloroform-*d*) δ 4.09 – 3.99 (m, 4H), 1.74 – 1.58 (m, 4H), 1.53 – 1.45 (m, 1H), 1.27 (t, *J* = 7.1 Hz, 6H), 1.20 (s, 12H), 1.17 – 1.08 (m, 2H), 0.86 (d, *J* = 3.9 Hz, 9H), 0.82 – 0.72 (m, 2H).
^{13}C NMR (126 MHz, Chloroform-*d*) δ 82.9, 61.38, 61.3, 50.2, 31.3, 31.1, 31.1, 30.5, 30.4, 30.1, 30.0, 24.9, 24.8, 23.7, 22.6, 16.5, 16.4.
^{11}B NMR (160 MHz, Chloroform-*d*) δ 33.72.
^{31}P NMR (202 MHz, Chloroform-*d*) δ 33.44.
HRMS (ESI) m/z: $[M+H]^+$ Calcd. for $C_{18}H_{35}BO_5P$ 374.2388; Found: 374.2387.

N-(tert-butyl)-6,6-dimethyl-4-((4,4,5,5-tetramethyl-1,3,2-dioxaborolan-2-yl)methyl)heptanamide (7g)

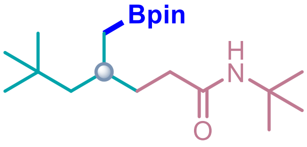

7g, 69%

Following the **general procedure 7** on 0.2 mmol scale, colorless oil, yield: 69% (48.7 mg), $R_f = 0.4$ (silica gel, PE: EA = 30:1, v/v), column chromatography (silica gel, PE: EA = 30:1, v/v).

^{1}H NMR (500 MHz, Chloroform-*d*) δ 5.36 (s, 1H), 2.17 – 2.00 (m, 2H), 1.70 (m, 2H), 1.56 – 1.40 (m, 2H), 1.35 (d, *J* = 1.6 Hz, 9H), 1.25 (s, 12H), 1.23 – 1.12 (m, 2H), 0.94 – 0.82 (m, 10H).

^{13}C NMR (126 MHz, Chloroform-*d*) δ 172.9, 82.9, 51.0, 50.7, 35.5, 34.6, 31.2, 30.1, 30.1, 28.9, 24.9, 24.9.

^{11}B NMR (160 MHz, Chloroform-*d*) δ 34.12.

HRMS (ESI) m/z: $[M+H]^+$ Calcd. for $C_{20}H_{40}BNO_3$ 354.3174; Found: 354.3179.

6,6-dimethyl-4-((4,4,5,5-tetramethyl-1,3,2-dioxaborolan-2-yl)methyl)heptanamide (7h)

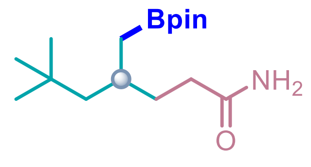

7h, 62%

Following the **general procedure 7** on 0.2 mmol scale, colorless oil, yield: 62% (36.8 mg), $R_f = 0.4$ (silica gel, PE: EA = 30:1, v/v), column chromatography (silica gel, PE: EA = 30:1, v/v).

^{1}H NMR (500 MHz, Chloroform-*d*) δ 5.92 (s, 1H), 5.70 (s, 1H), 2.22 (m, 2H), 1.72 – 1.51 (m, 3H), 1.23 (s, 14H), 1.12 (dd, *J* = 14.1, 5.3 Hz, 1H), 0.87 (s, 9H), 0.77 (dd, *J* = 15.8, 7.3 Hz, 1H).

^{13}C NMR (126 MHz, Chloroform-*d*) δ 176.2, 83.0, 50.7, 34.1, 33.6, 31.1, 30.0, 29.9, 24.8, 24.8.

^{11}B NMR (160 MHz, Chloroform-*d*) δ 34.04.

HRMS (ESI) m/z: $[M+H]^+$ Calcd. for $C_{16}H_{32}BNO_3$ 298.2548; Found: 298.2457.

2-(4,4-dimethyl-2-(2-(methylsulfonyl)ethyl)pentyl)-4,4,5,5-tetramethyl-1,3,2-dioxaborolane (7i)

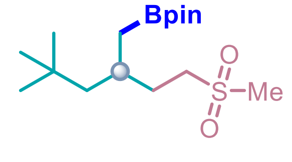

7i, 67%

Following the **general procedure 7** on 0.2 mmol scale, colorless oil, yield: 67% (44.5 mg), $R_f = 0.4$ (silica gel, PE: EA = 30:1, v/v), column chromatography (silica gel, PE: EA = 30:1, v/v).

^{1}H NMR (500 MHz, Chloroform-*d*) δ 3.08 – 2.91 (m, 2H), 2.86 (s, 3H), 1.91 – 1.83 (m, 1H), 1.83 – 1.72 (m, 2H), 1.21 (d, *J* = 3.2 Hz, 15H), 1.18 (d, *J* = 4.7 Hz, 1H), 0.88 (s, 9H), 0.78 (dd, *J* = 15.7, 7.0 Hz, 1H).

^{13}C NMR (126 MHz, Chloroform-*d*) δ 83.2, 52.9, 50.5, 40.2, 40.2, 31.1, 30.5, 30.0, 29.7, 29.4, 24.9, 24.9, 24.8.

^{11}B NMR (160 MHz, Chloroform-*d*) δ 33.76.

HRMS (ESI) m/z: $[M+H]^+$ Calcd. for $C_{16}H_{33}BO_4S$ 333.2265; Found: 333.2261.

2-(4,4-dimethyl-2-(2-(phenylsulfonyl)ethyl)pentyl)-4,4,5,5-tetramethyl-1,3,2-dioxaborolane (7j)

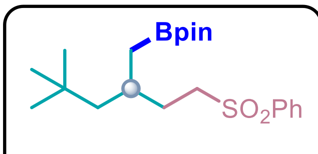

7j, 51%

Following the **general procedure 7** on 0.2 mmol scale, colorless oil, yield: 78% (48.4 mg), $R_f = 0.4$ (silica gel, PE: EA = 30:1, v/v), column chromatography (silica gel, PE: EA = 30:1, v/v).

^{1}H NMR (500 MHz, Chloroform-*d*) δ 7.92 – 7.86 (m, 2H), 7.65 – 7.60 (m, 1H), 7.57 – 7.51 (m, 2H), 3.16 – 3.01 (m, 2H), 1.79 – 1.66 (m, 3H), 1.65 – 1.55 (m, 1H), 1.15 (d, *J* = 5.6 Hz, 12H), 1.10 (d, *J* = 4.9 Hz, 2H), 0.82 (d, *J* = 1.5 Hz, 9H), 0.79 – 0.65 (m, 2H).

^{13}C NMR (126 MHz, Chloroform-*d*) δ 139.2, 133.4, 129.1, 128.0, 83.0, 54.3, 50.3, 31.0, 30.6, 29.9, 29.6, 24.8, 24.7.

^{11}B NMR (160 MHz, Chloroform-*d*) δ 33.79.

HRMS (ESI) m/z: $[M+H]^+$ Calcd. for $C_{21}H3_5BO_4S$ 395.2422; Found: 395.2428.

2-methoxyethyl 6,6-dimethyl-4-((4,4,5,5-tetramethyl-1,3,2-dioxaborolan-2-yl)methyl)heptanoate (7k)

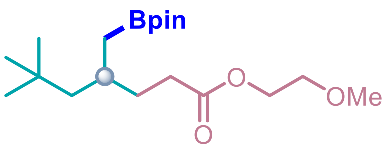

7k, 53%

Following the **general procedure 7** on 0.2 mmol scale, colorless oil, yield: 53% (37.3 mg), $R_f = 0.4$ (silica gel, PE: EA = 30:1, v/v), column chromatography (silica gel, PE: EA = 30:1, v/v).

^{1}H NMR (500 MHz, Chloroform-*d*) δ 4.24 – 4.17 (m, 2H), 3.61 – 3.55 (m, 2H), 3.38 (d, *J* = 1.2 Hz, 3H), 2.33 (m, 2H), 1.68 – 1.63 (m, 1H), 1.56 (m, 1H), 1.22 (s, 14H), 1.20 – 1.09 (m, 2H), 0.91 – 0.78 (m, 10H).

^{13}C NMR (126 MHz, Chloroform-*d*) δ 174.2, 82.9, 82.9, 70.5, 63.2, 59.0, 50.4, 33.3, 31.9, 31.1, 30.1, 30.0, 24.9, 24.8, 12.5.

^{11}B NMR (160 MHz, Chloroform-*d*) δ 33.93.

HRMS (ESI) m/z: $[M+Na]^+$ Calcd. for $C_{19}H_{37}BO_5$ 379.2626; Found: 379.2624.

(tetrahydrofuran-2-yl)methyl 6,6-dimethyl-4-((4,4,5,5-tetramethyl-1,3,2-dioxaborolan-2-yl)methyl)heptanoate (7l)

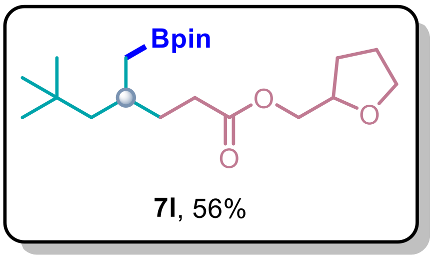

Following the **general procedure 7** on 0.2 mmol scale, colorless oil, yield: 56% (42.8 mg), $R_f = 0.4$ (silica gel, PE: EA = 30:1, v/v), column chromatography (silica gel, PE: EA = 30:1, v/v).

^{1}H NMR (500 MHz, Chloroform-*d*) δ 4.14 – 4.06 (m, 2H), 3.97 (m, 1H), 3.87 (dt, *J* = 8.4, 6.7 Hz, 1H), 3.77 (m, 1H), 2.41 – 2.25 (m, 2H), 2.03 – 1.93 (m, 1H), 1.93 – 1.83 (m, 2H), 1.74 – 1.63 (m, 2H), 1.57 (m, 2H), 1.22 (s, 13H), 1.19 – 1.10 (m, 2H), 0.90 – 0.78 (m, 10H).

^{13}C NMR (126 MHz, Chloroform-*d*) δ 174.1, 82.8, 68.4, 66.23, 66.2, 50.4, 45.7, 33.3, 32.7, 31.9, 31.1, 30.1, 28.2, 28.0, 25.6, 24.8, 24.8, 22.7, 22.6, 12.5.

^{11}B NMR (160 MHz, Chloroform-*d*) δ 33.98.

HRMS (ESI) m/z: $[M+H]^+$ Calcd. for $C_{21}H_{39}BO_5$ 383.2963; Found: 383.2967.

cyclopropylmethyl 6,6-dimethyl-4-((4,4,5,5-tetramethyl-1,3,2-dioxaborolan-2-yl)methyl)heptanoate (7m)

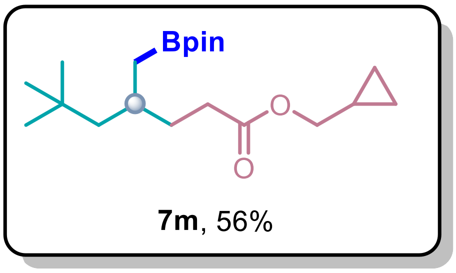

Following the **general procedure 7** on 0.2 mmol scale, colorless oil, yield: 56% (39.4 mg), $R_f = 0.4$ (silica gel, PE: EA = 30:1, v/v), column chromatography (silica gel, PE: EA = 30:1, v/v).

^{1}H NMR (500 MHz, Chloroform-*d*) δ 4.44 – 4.29 (m, 1H), 4.01 – 3.86 (m, 1H), 3.20 (m, 1H), 2.87 – 2.79 (m, 1H), 2.68 – 2.61 (m, 1H), 2.54 (m, 1H), 1.77 – 1.69 (m, 1H), 1.62 (m, 1H), 1.22 (d, *J* = 4.2 Hz, 12H), 1.20 – 1.17 (m, 1H), 1.15 (m, 4H), 0.89 (d, *J* = 2.2 Hz, 9H), 0.87 – 0.79 (m, 2H).

^{13}C NMR (126 MHz, Chloroform-*d*) δ 177.0, 176.9, 82.9, 82.8, 64.8, 65.0, 64.6, 50.9, 50.8, 49.4, 49.4, 44.7, 44.7, 44.7, 43.0, 42.9, 42.8, 42.7, 37.4, 37.4, 37.3, 37.3, 31.2, 31.1, 30.2, 30.2, 28.6, 28.2, 28.2, 25.0, 24.9, 24.9, 17.8, 17.8, 17.0, 17.0.

^{11}B NMR (160 MHz, Chloroform-*d*) δ 34.10.

HRMS (ESI) m/z: $[M+H]^+$ Calcd. for $C_{20}H_{37}BO_4$ 353.2858; Found: 353.2854.

ethyl 4-methyl-5-(4,4,5,5-tetramethyl-1,3,2-dioxaborolan-2-yl)pentanoate (7n)

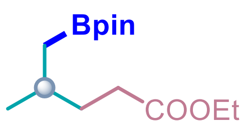

7n, 73%

Following the **general procedure 7** on 0.2 mmol scale, colorless oil, yield: 73% (39.4 mg), $R_f = 0.4$ (silica gel, PE: EA = 30:1, v/v), column chromatography (silica gel, PE: EA = 30:1, v/v).

^{1}H NMR (500 MHz, Chloroform-*d*) δ 4.10 (m, 2H), 2.28 (m, 2H), 1.61 (m, 1H), 1.51 (m, 1H), 1.26 – 1.21 (m, 15H), 0.91 (d, *J* = 6.6 Hz, 3H), 0.83 (dd, *J* = 15.4, 5.5 Hz, 1H), 0.66 (dd, *J* = 15.4, 8.5 Hz, 1H).

^{13}C NMR (126 MHz, Chloroform-*d*) δ 174.1, 82.9, 60.1, 34.4, 32.4, 29.2, 24.9, 24.7, 21.9, 14.2.

^{11}B NMR (160 MHz, Chloroform-*d*) δ 33.99.

HRMS (ESI) m/z: $[M+H]^+$ Calcd. for $C_{14}H_{27}BO_4$ 271.2075; Found: 271.2078.

ethyl 4-((4,4,5,5-tetramethyl-1,3,2-dioxaborolan-2-yl)methyl)heptanoate (7o)

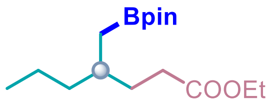

7o, 53%

Following the **general procedure 7** on 0.2 mmol scale, colorless oil, yield: 53% (31.6 mg), $R_f = 0.4$ (silica gel, PE: EA = 30:1, v/v), column chromatography (silica gel, PE: EA = 30:1, v/v).

^{1}H NMR (500 MHz, Chloroform-*d*) δ 4.12 (m, 2H), 2.33 – 2.24 (m, 2H), 1.71 – 1.51 (m, 3H), 1.29 – 1.24 (m, 19H), 0.88 (t, *J* = 7.1 Hz, 3H), 0.77 (d, *J* = 6.6 Hz, 2H).

^{13}C NMR (126 MHz, Chloroform-*d*) δ 174.2, 82.9, 60.1, 36.1, 33.8, 32.2, 32.0, 31.5, 26.4, 24.8, 24.8, 22.6, 14.2, 14.1.

^{11}B NMR (160 MHz, Chloroform-*d*) δ 33.95.

HRMS (ESI) m/z: $[M+H]^+$ Calcd. for $C_{16}H_{31}BO_4$ 299.2388; Found: 299.2390.

ethyl 6-methyl-4-((4,4,5,5-tetramethyl-1,3,2-dioxaborolan-2-yl)methyl)heptanoate (7p)

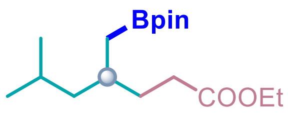

7p, 53%

Following the **general procedure 7** on 0.2 mmol scale, colorless oil, yield: 53% (33.1 mg), $R_f = 0.4$ (silica gel, PE: EA = 30:1, v/v), column chromatography (silica gel, PE: EA = 30:1, v/v).

^{1}H NMR (500 MHz, Chloroform-*d*) δ 4.07 (m, 2H), 2.25 (m, 2H), 1.69 – 1.56 (m, 3H), 1.48 (m, 1H), 1.23 – 1.18 (m, 15H), 1.09 (m, 1H), 1.02 (m, 1H), 0.81 (dd, *J* = 8.8, 6.5 Hz, 6H), 0.70 (dd, *J* = 6.4, 3.6 Hz, 2H).

^{13}C NMR (126 MHz, Chloroform-*d*) δ 174.2, 82.9, 60.1, 46.1, 31.8, 31.5, 31.4, 25.2, 24.8, 24.8, 22.9, 22.8, 14.2.
^{11}B NMR (160 MHz, Chloroform-*d*) δ 33.87.
HRMS (ESI) m/z: $[M+H]^+$ Calcd. for $C_{17}H_{33}BO_4$ 313.2545; Found: 313.2548.

ethyl 6-phenyl-4-((4,4,5,5-tetramethyl-1,3,2-dioxaborolan-2-yl)methyl)hexanoate (7q)

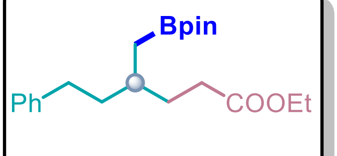

7q, 62%

Following the **general procedure 7** on 0.2 mmol scale, colorless oil, yield: 62% (44.6 mg), R_f = 0.4 (silica gel, PE: EA = 30:1, v/v), column chromatography (silica gel, PE: EA = 30:1, v/v).
^{1}H NMR (500 MHz, Chloroform-*d*) δ 7.26 (t, *J* = 7.5 Hz, 2H), 7.19 – 7.14 (m, 3H), 4.11 (q, *J* = 7.1 Hz, 2H), 2.61 (m, 2H), 2.31 (m, 2H), 1.73 (m, 2H), 1.60 (m, 3H), 1.24 (s, 15H), 0.85 (d, *J* = 6.4 Hz, 2H).
^{13}C NMR (126 MHz, Chloroform-*d*) δ 174.1, 142.9, 128.4, 128.4, 128.3, 125.6, 83.0, 60.2, 38.2, 33.7, 33.2, 21.0, 31.3, 24.9, 24.9, 14.3.
^{11}B NMR (160 MHz, Chloroform-*d*) δ 34.29.
HRMS (ESI) m/z: $[M+H]^+$ Calcd. for $C_{21}H_{33}BO_4$ 361.2545; Found: 361.2547.

ethyl 5-ethyl-4-((4,4,5,5-tetramethyl-1,3,2-dioxaborolan-2-yl)methyl)heptanoate (7r)

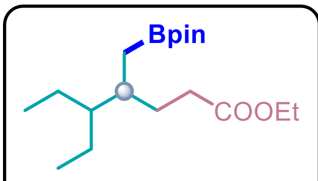

7r, 65%

Following the **general procedure 7** on 0.2 mmol scale, colorless oil, yield: 65% (42.4 mg), R_f = 0.4 (silica gel, PE: EA = 30:1, v/v), column chromatography (silica gel, PE: EA = 30:1, v/v).
^{1}H NMR (500 MHz, Chloroform-*d*) δ 4.08 (m, 2H), 2.25 (m, 2H), 1.75 – 1.68 (m, 1H), 1.67 – 1.59 (m, 1H), 1.48 – 1.39 (m, 1H), 1.29 – 1.13 (m, 19H), 1.02 (m, 1H), 0.85 (dd, *J* = 7.4, 5.1 Hz, 6H), 0.73 (dd, *J* = 15.5, 6.9 Hz, 1H), 0.64 (dd, *J* = 15.5, 7.4 Hz, 1H).
^{13}C NMR (126 MHz, Chloroform-*d*) δ 174.2, 82.9, 60.1, 45.8, 35.1, 32.9, 28.3, 24.8, 22.7, 22.6, 14.2, 12.5, 12.5.
^{11}B NMR (160 MHz, Chloroform-*d*) δ 34.20.
HRMS (ESI) m/z: $[M+H]^+$ Calcd. for $C_{18}H_{35}BO_4$ 327.2701; Found: 327.2775.

ethyl 4-cyclopentyl-5-(4,4,5,5-tetramethyl-1,3,2-dioxaborolan-2-yl)pentanoate (7s)

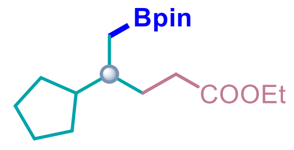

7s, 65%

Following the **general procedure 7** on 0.2 mmol scale, colorless oil, yield: 65% (42.1 mg), $R_f = 0.4$ (silica gel, PE: EA = 30:1, v/v), column chromatography (silica gel, PE: EA = 30:1, v/v).

^{1}H NMR (500 MHz, Chloroform-*d*) δ 4.10 (m, 2H), 2.37 – 2.21 (m, 2H), 1.76 – 1.66 (m, 4H), 1.60 – 1.46 (m, 6H), 1.23 (d, *J* = 2.6 Hz, 15H), 1.11 (m, 2H), 0.88 – 0.67 (m, 2H).

^{13}C NMR (126 MHz, Chloroform-*d*) δ 174.4, 82.9, 60.1, 45.8, 38.9, 31.7, 30.6, 30.4, 30.3, 25.4, 25.3, 24.9, 24.8, 14.3.

^{11}B NMR (160 MHz, Chloroform-*d*) δ 34.31.

HRMS (ESI) m/z: $[M+H]^+$ Calcd. for $C_{18}H_{33}BO_4$ 325.2545; Found: 325.2540.

ethyl 4-cyclohexyl-5-(4,4,5,5-tetramethyl-1,3,2-dioxaborolan-2-yl)pentanoate (7t)

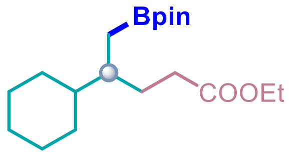

7t, 69%

Following the **general procedure 7** on 0.2 mmol scale, colorless oil, yield: 69% (46.6 mg), $R_f = 0.4$ (silica gel, PE: EA = 30:1, v/v), column chromatography (silica gel, PE: EA = 30:1, v/v).

^{1}H NMR (500 MHz, Chloroform-*d*) δ 4.10 (m, 2H), 2.35 – 2.19 (m, 2H), 1.76 – 1.68 (m, 3H), 1.64 – 1.56 (m, 3H), 1.49 (m, 2H), 1.23 (d, *J* = 2.8 Hz, 18H), 1.12 – 1.06 (m, 1H), 1.04 – 0.93 (m, 2H), 0.82 – 0.75 (m, 1H), 0.66 (dd, *J* = 15.6, 6.9 Hz, 1H).

^{13}C NMR (126 MHz, Chloroform-*d*) δ 174.2, 82.9, 60.1, 42.2, 39.2, 32.6, 30.0, 29.2, 28.7, 26.8, 26.8, 26.7, 24.8, 24.8, 24.8, 14.2.

^{11}B NMR (160 MHz, Chloroform-*d*) δ 34.11.

HRMS (ESI) m/z: $[M+H]^+$ Calcd. for $C_{19}H_{35}BO_4$ 339.2701; Found: 339.2705.

ethyl 5-methyl-4-((4,4,5,5-tetramethyl-1,3,2-dioxaborolan-2-yl)methyl)octanoate (7u)

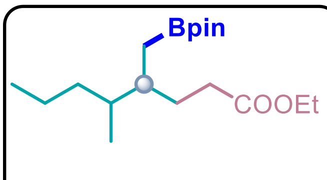

7u, 70%

Following the **general procedure 7** on 0.2 mmol scale, colorless oil, yield: 70% (45.6mg), $R_f = 0.4$ (silica gel, PE: EA = 30:1, v/v), column chromatography (silica gel, PE: EA = 30:1, v/v).

^{1}H NMR (500 MHz, Chloroform-*d*) δ 4.09 (m, 2H), 2.36 – 2.18 (m, 2H), 1.74 – 1.47 (m, 3H), 1.46 – 1.31 (m, 3H), 1.32 – 1.12 (m, 19H), 1.06 (m, 1H), 0.85 (m, 3H), 0.77 (dd, *J* = 19.0, 6.8 Hz, 3H), 0.72 (dd, *J* = 7.0, 3.8 Hz, 1H).

^{13}C NMR (126 MHz, Chloroform-*d*) δ 174.2, 174.2, 82.9, 60.1, 60.1, 38.5, 38.3, 36.8, 36.5, 35.9, 35.7, 32.9, 32.8, 29.3, 27.6, 24.9, 24.8, 24.8, 24.8, 20.8, 20.8, 15.6, 14.7, 14.4, 14.3, 14.25.
^{11}B NMR (160 MHz, Chloroform-*d*) δ 34.29.
HRMS (ESI) m/z: $[M+H]^+$ Calcd. for $C_{18}H_{35}BO_4$ 327.2701; Found: 327.2768.

ethyl 3-(1-((4,4,5,5-tetramethyl-1,3,2-dioxaborolan-2-yl)methyl)cyclobutyl)propanoate (7v)

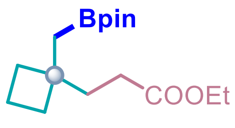

7v, 52%

Following the **general procedure 7** on 0.2 mmol scale, colorless oil, yield: 52% (30.8 mg), R_f = 0.4 (silica gel, PE: EA = 30:1, v/v), column chromatography (silica gel, PE: EA = 30:1, v/v).
^{1}H NMR (500 MHz, Chloroform-*d*) δ 4.11 (q, J = 7.1 Hz, 2H), 2.26 – 2.19 (m, 2H), 1.86 – 1.77 (m, 8H), 1.26 – 1.25 (m, 3H), 1.23 (s, 12H).
^{13}C NMR (126 MHz, Chloroform-*d*) δ 174.5, 82.9, 60.2, 39.5, 36.5, 33.4, 30.1, 24.9, 24.7, 14.9, 14.2.
^{11}B NMR (160 MHz, Chloroform-*d*) δ 33.58.
HRMS (ESI) m/z: $[M+H]^+$ Calcd. for $C_{16}H_{29}BO_4$ 297.2232; Found: 297.2235.

ethyl 6-(4-(tert-butyl)phenyl)-5-m ethyl-4-((4,4,5,5-tetramethyl-1,3,2-dioxaborolan-2-yl)methyl)hexanoate (7w)

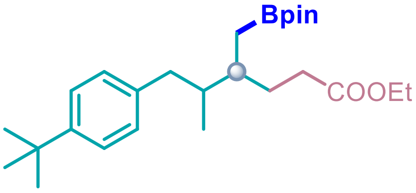

7w, 53%

Following the **general procedure 7** on 0.2 mmol scale, colorless oil, yield: 53% (45.6 mg), R_f = 0.4 (silica gel, PE: EA = 30:1, v/v), column chromatography (silica gel, PE: EA = 30:1, v/v).
^{1}H NMR (500 MHz, Chloroform-*d*) δ 7.29 – 7.25 (m, 2H), 7.07 (m, 2H), 4.11 (m, 2H), 2.68 (m, 1H), 2.38 (m, 1H), 2.32 – 2.24 (m, 2H), 1.86 – 1.75 (m, 2H), 1.72 – 1.56 (m, 2H), 1.49 (m, 1H), 1.30 (d, J = 1.3 Hz, 9H), 1.26 – 1.22 (m, 14H), 0.91 – 0.69 (m, 5H).
^{13}C NMR (126 MHz, Chloroform-*d*) δ 174.2, 148.3, 138.8, 128.8, 128.8, 125.0, 83.0, 83.0, 60.2, 60.2, 40.1, 39.6, 39.5, 38.7, 38.5, 38.4, 34.3, 32.9, 32.8, 31.5, 29.3, 27.8, 24.9, 24.9, 24.9, 24.8, 15.1, 14.4, 14.3.
^{11}B NMR (160 MHz, Chloroform-*d*) δ 34.68.
HRMS (ESI) m/z: $[M+H]^+$ Calcd. for $C_{26}H_{43}BO_4$ 431.3327; Found: 431.3324.

ethyl 4-((4,4,5,5-tetramethyl-1,3,2-dioxaborolan-2-yl)methyl)pentadecanoate (7x)

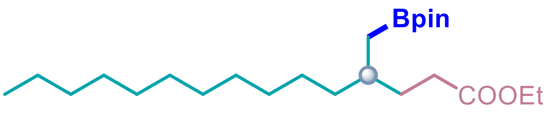

7x, 80%

Following the **general procedure 7** on 0.2 mmol scale, colorless oil, yield: 80% (65.6 mg), $R_f = 0.4$ (silica gel, PE: EA = 30:1, v/v), column chromatography (silica gel, PE: EA = 30:1, v/v).
^{1}H NMR (500 MHz, Chloroform-*d*) δ 4.09 (m, 2H), 2.26 (m, 2H), 1.69 – 1.50 (m, 3H), 1.28 – 1.19 (m, 35H), 0.86 (t, *J* = 6.9 Hz, 3H), 0.74 (d, *J* = 6.6 Hz, 2H).
^{13}C NMR (126 MHz, Chloroform-*d*) δ 174.2, 82.9, 60.1, 36.1, 33.8, 32.0, 31.9, 31.5, 30.0, 29.7, 29.7, 29.6, 29.6, 29.4, 26.7, 24.8, 24.8, 22.7, 14.2, 14.1.
^{11}B NMR (160 MHz, Chloroform-*d*) δ 34.24.
HRMS (ESI) m/z: $[M+H]^+$ Calcd. for $C_{24}H_{47}BO_4$ 411.3640; Found: 411.3637.

2,2'-(2-methyl-4-phenylpent-4-ene-1,2-diyl)bis(4,4,5,5-tetramethyl-1,3,2-dioxaborolane) (8a)

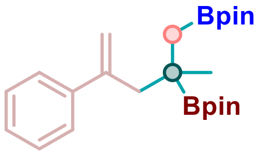

8a, 73%

Following the **general procedure 8.1** on 0.2 mmol scale, colorless oil, yield: 73% (60.2 mg), $R_f = 0.4$ (silica gel, PE: EA = 20:1, v/v), column chromatography (silica gel, PE: EA = 20:1, v/v).
^{1}H NMR (500 MHz, Chloroform-*d*) δ 7.41 – 7.37 (m, 2H), 7.26 (m, 2H), 7.22 – 7.17 (m, 1H), 5.20 (d, *J* = 2.0 Hz, 1H), 5.07 (d, *J* = 2.1 Hz, 1H), 2.73 (d, *J* = 13.6 Hz, 1H), 2.57 (d, *J* = 13.6 Hz, 1H), 1.21 (d, *J* = 4.0 Hz, 12H), 1.14 (s, 12H), 0.95 (d, *J* = 15.7 Hz, 1H), 0.87 (s, 3H), 0.72 (d, *J* = 15.7 Hz, 1H).
^{13}C NMR (126 MHz, Chloroform-*d*) δ 147.6, 143.7, 128.0, 126.9, 126.7, 116.0, 83.0, 82.8, 45.6, 25.0, 24.9, 24.8, 24.8, 24.4.
^{11}B NMR (160 MHz, Chloroform-*d*) δ 34.60, 34.10.
HRMS (ESI) m/z: $[M+H]^+$ Calcd. for $C_{24}H_{38}B_2O_4$ 413.3029; Found: 413.3027.

2,2'-(2-methyl-4-(p-tolyl)pent-4-ene-1,2-diyl)bis(4,4,5,5-tetramethyl-1,3,2-dioxaborolane) (8b)

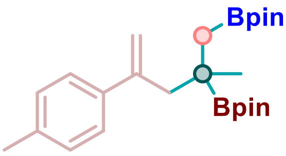

8b, 70%

Following the **general procedure 8.1** on 0.2 mmol scale, colorless oil, yield: 70% (60.2 mg), R_f = 0.4 (silica gel, PE: EA = 20:1, v/v), column chromatography (silica gel, PE: EA = 20:1, v/v).

^{1}H NMR (500 MHz, Chloroform-*d*) δ 7.30 – 7.27 (m, 2H), 7.07 (d, *J* = 7.9 Hz, 2H), 5.18 (d, *J* = 2.0 Hz, 1H), 5.03 (d, *J* = 2.0 Hz, 1H), 2.70 (d, *J* = 13.6 Hz, 1H), 2.56 (d, *J* = 13.6 Hz, 1H), 2.31 (s, 3H), 1.21 (d, *J* = 4.0 Hz, 12H), 1.15 (s, 12H), 0.95 (d, *J* = 15.7 Hz, 1H), 0.87 (s, 3H), 0.71 (d, *J* = 15.7 Hz, 1H).

^{13}C NMR (126 MHz, Chloroform-*d*) δ 147.4, 140.8, 136.49, 128.7, 126.59, 115.2, 83.0, 82.7, 45.5, 25.0, 24.9, 24.8, 24.8, 24.3, 21.1.

^{11}B NMR (160 MHz, Chloroform-*d*) δ 34.38, 34.10.

HRMS (ESI) m/z: $[M+H]^+$ Calcd. for $C_{25}H_{41}B_2O_4$ 427.3185; Found: 427.3183.

2,2'-(4-(4-bromophenyl)-2-methylpent-4-ene-1,2-diyl)bis(4,4,5,5-tetramethyl-1,3,2-dioxaborolane) (8c)

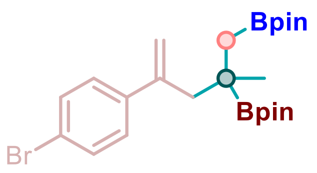

8c, 78%

Following the **general procedure 8.1** on 0.2 mmol scale, colorless oil, yield: 78% (76.4 mg), R_f = 0.4 (silica gel, PE: EA = 20:1, v/v), column chromatography (silica gel, PE: EA = 20:1, v/v).

^{1}H NMR (500 MHz, Chloroform-*d*) δ 7.38 – 7.35 (m, 2H), 7.27 – 7.23 (m, 2H), 5.18 (d, *J* = 1.8 Hz, 1H), 5.07 (d, *J* = 1.7 Hz, 1H), 2.66 (d, *J* = 13.7 Hz, 1H), 2.53 (d, *J* = 13.6 Hz, 1H), 1.20 (d, *J* = 3.6 Hz, 12H), 1.14 (s, 12H), 0.90 (d, *J* = 15.6 Hz, 1H), 0.84 (s, 3H), 0.68 (d, *J* = 15.6 Hz, 1H).

^{13}C NMR (126 MHz, Chloroform-*d*) δ 146.6, 142.7, 131.1, 128.5, 120.8, 116.6, 83.1, 82.8, 45.3, 25.0, 24.9, 24.7, 24.8, 24.4.

^{11}B NMR (160 MHz, Chloroform-*d*) δ 34.91, 34.42.

HRMS (ESI) m/z: $[M+H]^+$ Calcd. for $C_{24}H_{37}B_2BrO_4$ 491.2134; Found: 491.2140.

2,2'-(4-chloro-2-methylpent-4-ene-1,2-diyl)bis(4,4,5,5-tetramethyl-1,3,2-dioxaborolane) (8d)

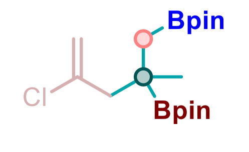

8d, 65%

Following the **general procedure 8.1** on 0.2 mmol scale, colorless oil, yield: 65% (48.1 mg), R_f = 0.4 (silica gel, PE: EA = 20:1, v/v), column chromatography (silica gel, PE: EA = 20:1, v/v).

^{1}H NMR (500 MHz, Chloroform-*d*) δ 5.49 (s, 1H), 5.43 (s, 1H), 2.86 (d, *J* = 14.3 Hz, 1H), 2.77 (d, *J* = 14.3 Hz, 1H), 1.56 (d, *J* = 8.0 Hz, 24H), 1.35 (d, *J* = 20.9 Hz, 4H), 1.12 (d, *J* = 15.7 Hz, 1H).

^{13}C NMR (126 MHz, Chloroform-*d*) δ 141.37, 114.24, 83.23, 82.84, 49.25, 24.94, 24.90, 24.86, 24.80, 23.63.
^{11}B NMR (160 MHz, Chloroform-*d*) δ 33.90, 33.87.
HRMS (ESI) m/z: $[M+H]^+$ Calcd. for $C_{18}H_{33}B_2ClO_4$ 371.2326; Found: 371.2322.

2,2'-(2-ethyl-4-phenylpent-4-ene-1,2-diyl)bis(4,4,5,5-tetramethyl-1,3,2-dioxaborolane) (8e)

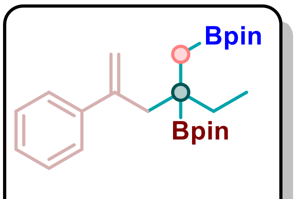

8e, 74%

Following the **general procedure 8.1** on 0.2 mmol scale, colorless oil, yield: 74% (63.2 mg), R_f = 0.4 (silica gel, PE: EA = 20:1, v/v), column chromatography (silica gel, PE: EA = 20:1, v/v).
^{1}H NMR (500 MHz, Chloroform-*d*) δ 7.40 – 7.36 (m, 2H), 7.28 – 7.23 (m, 2H), 7.21 – 7.17 (m, 1H), 5.16 (d, *J* = 2.1 Hz, 1H), 5.10 (d, *J* = 2.1 Hz, 1H), 2.77 (d, *J* = 13.9 Hz, 1H), 2.69 (d, *J* = 13.9 Hz, 1H), 1.39 (dd, *J* = 13.8, 7.5 Hz, 1H), 1.32 – 1.27 (m, 1H), 1.21 (d, *J* = 1.9 Hz, 12H), 1.15 (d, *J* = 3.6 Hz, 12H), 0.84 (d, *J* = 3.7 Hz, 2H), 0.72 (s, 3H).
^{13}C NMR (126 MHz, Chloroform-*d*) δ 148.0, 144.3, 127.9, 126.8, 126.8, 116.3, 83.0, 82.6, 41.5, 30.1, 25.1, 24.98, 25.0, 24.9, 9.7.
^{11}B NMR (160 MHz, Chloroform-*d*) δ 35.76, 34.19.
HRMS (ESI) m/z: $[M+H]^+$ Calcd. for $C_{25}H_{40}B_2O_4$ 427.3185; Found: 427.3174.

2,2'-(2-ethyl-4-(4-fluorophenyl)pent-4-ene-1,2-diyl)bis(4,4,5,5-tetramethyl-1,3,2-dioxaborolane) (8f)

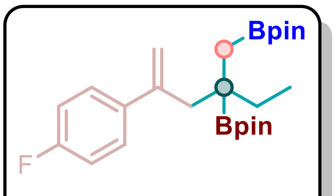

8f, 77%

Following the **general procedure 8.1** on 0.2 mmol scale, colorless oil, yield: 77% (68.4 mg), R_f = 0.4 (silica gel, PE: EA = 20:1, v/v), column chromatography (silica gel, PE: EA = 20:1, v/v).
^{1}H NMR (500 MHz, Chloroform-*d*) δ 7.36 – 7.31 (m, 2H), 6.96 – 6.90 (m, 2H), 5.11 (d, *J* = 2.0 Hz, 1H), 5.07 (d, *J* = 2.0 Hz, 1H), 2.72 (d, *J* = 13.9 Hz, 1H), 2.64 (d, *J* = 13.8 Hz, 1H), 1.37 (dd, *J* = 13.8, 7.5 Hz, 1H), 1.31 – 1.26 (m, 1H), 1.20 (d, *J* = 1.4 Hz, 12H), 1.14 (d, *J* = 3.2 Hz, 12H), 0.86 – 0.77 (m, 2H), 0.72 (t, *J* = 7.5 Hz, 3H).
^{13}C NMR (126 MHz, Chloroform-*d*) δ 163.0, 161.1, 147.0, 140.3, 140.3, 128.3, 128.3, 116.3, 114.7, 114.6, 83.0, 82.6, 41.7, 30.2, 25.1, 25.0, 24.9, 24.8, 9.7.
^{19}F NMR (471 MHz, Chloroform-*d*) δ -116.48.
^{11}B NMR (160 MHz, Chloroform-*d*) δ 34.63, 34.22.
HRMS (ESI) m/z: $[M+H]^+$ Calcd. for $C_{25}H_{39}B_2FO_4$ 445.3091; Found: 445.3088.

2,2'-(4-(4-chlorophenyl)-2-ethylpent-4-ene-1,2-diyl)bis(4,4,5,5-tetramethyl-1,3,2-dioxaborolane) (8g)

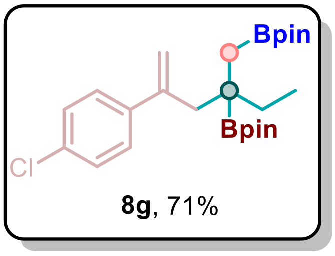

Following the **general procedure 8.1** on 0.2 mmol scale, colorless oil, yield: 71% (65.3 mg), R_f = 0.4 (silica gel, PE: EA = 20:1, v/v), column chromatography (silica gel, PE: EA = 20:1, v/v).

^{1}H NMR (500 MHz, Chloroform-*d*) δ 7.32 – 7.29 (m, 2H), 7.23 – 7.19 (m, 2H), 5.14 (d, *J* = 1.9 Hz, 1H), 5.10 (d, *J* = 1.9 Hz, 1H), 2.71 (d, *J* = 13.9 Hz, 1H), 2.64 (d, *J* = 13.9 Hz, 1H), 1.36 (dd, *J* = 13.8, 7.4 Hz, 1H), 1.28 – 1.24 (m, 1H), 1.20 (d, *J* = 1.6 Hz, 12H), 1.14 (d, *J* = 2.9 Hz, 12H), 0.86 – 0.76 (m, 2H), 0.72 (t, *J* = 7.5 Hz, 3H).

^{13}C NMR (126 MHz, Chloroform-*d*) δ 146.9, 142.7, 132.5, 128.2, 128.0, 116.8, 83.0, 82.6, 41.4, 30.3, 25.1, 25.0, 24.9, 24.8, 9.8.

^{11}B NMR (160 MHz, Chloroform-*d*) δ 34.72, 34.65.

HRMS (ESI) m/z: $[M+H]^+$ Calcd. for $C_{25}H_{39}B_2ClO_4$ 461.2796; Found: 461.2798.

2,2'-(2-ethyl-4-(4-methoxyphenyl)pent-4-ene-1,2-diyl)bis(4,4,5,5-tetramethyl-1,3,2-dioxaborolane) (8h)

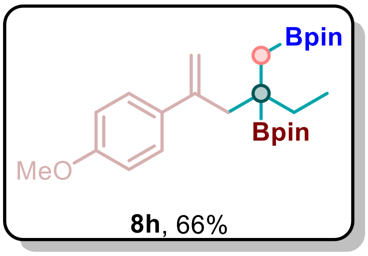

Following the **general procedure 8.1** on 0.2 mmol scale, colorless oil, yield: 66% (60.2 mg), R_f = 0.4 (silica gel, PE: EA = 20:1, v/v), column chromatography (silica gel, PE: EA = 20:1, v/v).

^{1}H NMR (500 MHz, Chloroform-*d*) δ 7.34 – 7.29 (m, 2H), 6.82 – 6.76 (m, 2H), 5.10 (d, *J* = 2.1 Hz, 1H), 5.02 (d, *J* = 2.1 Hz, 1H), 3.77 (s, 3H), 2.72 (d, *J* = 13.9 Hz, 1H), 2.64 (d, *J* = 13.7 Hz, 1H), 1.42 – 1.35 (m, 1H), 1.32 – 1.27 (m, 1H), 1.21 (d, *J* = 1.8 Hz, 12H), 1.15 (d, *J* = 3.7 Hz, 12H), 0.83 (d, *J* = 1.8 Hz, 2H), 0.73 (t, *J* = 7.5 Hz, 3H).

^{13}C NMR (126 MHz, Chloroform-*d*) δ 158.7, 147.3, 136.8, 127.8, 115.0, 113.3, 83.0, 82.6, 55.2, 41.6, 30.1, 25.1, 25.0, 24.9, 9.8.

^{11}B NMR (160 MHz, Chloroform-*d*) δ 35.04, 34.72.

HRMS (ESI) m/z: $[M+H]^+$ Calcd. for $C_{26}H_{42}B_2O_5$ 457.3291; Found: 457.3288.

2,2'-(2-ethyl-4-(naphthalen-2-yl)pent-4-ene-1,2-diyl)bis(4,4,5,5-tetramethyl-1,3,2-dioxaborolane) (8i)

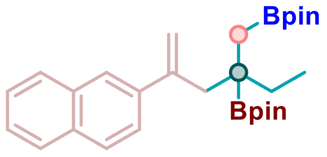

8i, 70%

Following the **general procedure 8.1** on 0.2 mmol scale, colorless oil, yield: 70% (66.6 mg), R_f = 0.4 (silica gel, PE: EA = 20:1, v/v), column chromatography (silica gel, PE: EA = 20:1, v/v).
^{1}H NMR (500 MHz, Chloroform-*d*) δ 7.84 (d, *J* = 1.7 Hz, 1H), 7.79 (m, 2H), 7.74 (d, *J* = 8.5 Hz, 1H), 7.56 (dd, *J* = 8.5, 1.8 Hz, 1H), 7.42 (m, 2H), 5.31 (d, *J* = 2.0 Hz, 1H), 5.22 (d, *J* = 1.9 Hz, 1H), 2.91 (d, *J* = 14.1 Hz, 1H), 2.85 – 2.77 (m, 1H), 1.44 (dd, *J* = 13.8, 7.5 Hz, 1H), 1.36 – 1.31 (m, 1H), 1.22 (d, *J* = 4.4 Hz, 12H), 1.11 (d, *J* = 3.5 Hz, 12H), 0.92 – 0.89 (m, 2H), 0.75 (t, *J* = 7.4 Hz, 3H).
^{13}C NMR (126 MHz, Chloroform-*d*) δ 147.9, 141.6, 133.4, 132.7, 128.1, 127.5, 127.4, 125.8, 125.8, 125.4, 125.2, 117.0, 83.0, 82.6, 41.7, 30.3, 25.0, 25.0, 24.9, 24.9, 9.1.
^{11}B NMR (160 MHz, Chloroform-*d*) δ 35.13, 34.10.
HRMS (ESI) m/z: $[M+H]^+$ Calcd. for $C_{29}H_{42}B_2O_4$ 477.3342; Found: 477.3345.

2,2'-(4-chloro-2-ethylpent-4-ene-1,2-diyl)bis(4,4,5,5-tetramethyl-1,3,2-dioxaborolane) (8j)

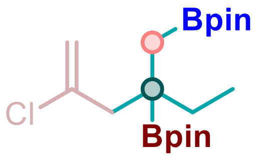

8j, 67%

Following the **general procedure 8.1** on 0.2 mmol scale, colorless oil, yield: 67% (51.5 mg), R_f = 0.4 (silica gel, PE: EA = 20:1, v/v), column chromatography (silica gel, PE: EA = 20:1, v/v).
^{1}H NMR (500 MHz, Chloroform-*d*) δ 5.16 (d, *J* = 0.8 Hz, 1H), 5.11 (d, *J* = 0.8 Hz, 1H), 2.57 (t, *J* = 1.0 Hz, 2H), 1.55 (dd, *J* = 13.8, 7.5 Hz, 1H), 1.51 – 1.43 (m, 1H), 1.23 (s, 12H), 1.20 (s, 12H), 1.00 – 0.88 (m, 2H), 0.84 (t, *J* = 7.5 Hz, 3H).
^{13}C NMR (126 MHz, Chloroform-*d*) δ 141.3, 114.5, 83.2, 82.7, 45.1, 29.8, 25.1, 25.0, 24.9, 24.8, 9.8.
^{11}B NMR (160 MHz, Chloroform-*d*) δ 33.92, 32.96.
HRMS (ESI) m/z: $[M+H]^+$ Calcd. for $C_{19}H_{35}B_2ClO_4$ 385.2483; Found: 385.2480.

2,2'-(2-(2-phenylallyl)hexane-1,2-diyl)bis(4,4,5,5-tetramethyl-1,3,2-dioxaborolane) (8k)

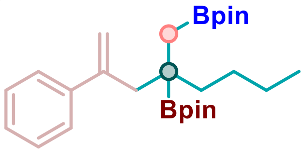

8k, 69%

Following the **general procedure 8.1** on 0.2 mmol scale, colorless oil, yield: 69% (65.7 mg), R_f = 0.4 (silica gel, PE: EA = 20:1, v/v), column chromatography (silica gel, PE: EA = 20:1, v/v).
^{1}H NMR (500 MHz, Chloroform-*d*) δ 7.39 – 7.35 (m, 2H), 7.27 – 7.22 (m, 2H), 7.21 – 7.16 (m, 1H), 5.15 (d, *J* = 2.1 Hz, 1H), 5.10 (d, *J* = 2.0 Hz, 1H), 2.77 (d, *J* = 13.9 Hz, 1H), 2.67 (d, *J* = 13.8 Hz, 1H), 1.29 – 1.24 (m, 2H), 1.21 (s, 12H), 1.15 (d, *J* = 4.9 Hz, 12H), 1.10 (dd, *J* = 7.0, 2.6 Hz, 2H), 0.98 (p, *J* = 7.0 Hz, 2H), 0.86 (d, *J* = 4.5 Hz, 2H), 0.82 (t, *J* = 7.2 Hz, 3H).
^{13}C NMR (126 MHz, Chloroform-*d*) δ 148.1, 144.3, 127.9, 126.8, 126.8, 116.4, 83.0, 82.6, 41.6, 37.7, 31.8, 30.0, 25.1, 25.0, 24.8, 22.6, 14.1.
^{11}B NMR (160 MHz, Chloroform-*d*) δ 34.50, 33.86.
HRMS (ESI) m/z: $[M+H]^+$ Calcd. for $C_{27}H_{44}B_2O_4$ 455.3498; Found: 455.3499.

2,2'-(2-(2-chloroallyl)hexane-1,2-diyl)bis(4,4,5,5-tetramethyl-1,3,2-dioxaborolane) (8l)

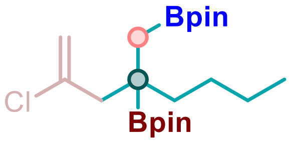

8l, 64%

Following the **general procedure 8.1** on 0.2 mmol scale, colorless oil, yield: 64% (52.7 mg), R_f = 0.4 (silica gel, PE: EA = 20:1, v/v), column chromatography (silica gel, PE: EA = 20:1, v/v).
^{1}H NMR (500 MHz, Chloroform-*d*) δ 5.16 (d, *J* = 0.8 Hz, 1H), 5.10 (d, *J* = 0.8 Hz, 1H), 2.57 (s, 2H), 1.51 – 1.44 (m, 1H), 1.42 – 1.33 (m, 1H), 1.23 (s, 16H), 1.20 (s, 12H), 1.02 – 0.90 (m, 2H), 0.84 (t, *J* = 6.7 Hz, 3H).
^{13}C NMR (126 MHz, Chloroform-*d*) δ 141.4, 114.5, 83.2, 82.7, 45.5, 37.5, 31.8, 30.1, 25.2, 25.1, 25.0, 25.0, 24.8, 22.6, 14.1.
^{11}B NMR (160 MHz, Chloroform-*d*) δ 34.40, 33.78.
HRMS (ESI) m/z: $[M+H]^+$ Calcd. for $C_{21}H_{39}B_2ClO_4$ 413.2796; Found: 413.2798.

2-methyl-4-phenylbut-3-yne-1,2-diol (8m)

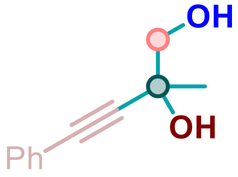

8m, 65%^{b}

Following the **general procedure 8.2** on 0.2 mmol scale, colorless oil, yield: 65% (22.9 mg), R_f = 0.4 (silica gel, PE: EA = 2:1, v/v), column chromatography (silica gel, PE: EA = 5:1, v/v).
^{1}H NMR (500 MHz, Chloroform-*d*) δ 7.46 – 7.40 (m, 2H), 7.31 (m, 3H), 3.76 (d, *J* = 11.1 Hz, 1H), 3.58 (d, *J* = 11.1 Hz, 1H), 1.55 (s, 3H).
^{13}C NMR (126 MHz, Chloroform-*d*) δ 131.78, 128.62, 128.33, 122.17, 90.36, 84.51, 70.77, 69.05, 25.38.
HRMS (ESI) m/z: $[M+H]^+$ Calcd. for $C_{11}H_{12}O_2$ 177.0910; Found: 177.0916.

2-ethyl-4-phenylbut-3-yne-1,2-diol (8n)

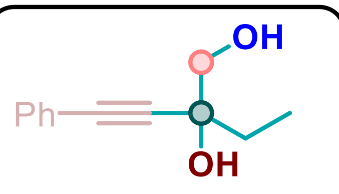

8n, 63%^{b}

Following the **general procedure 8.2** on 0.2 mmol scale, colorless oil, yield: 63% (24.0 mg), R_f = 0.4 (silica gel, PE: EA = 2:1, v/v), column chromatography (silica gel, PE: EA = 5:1, v/v).
^{1}H NMR (500 MHz, Chloroform-*d*) δ 7.47 – 7.40 (m, 2H), 7.36 – 7.28 (m, 3H), 3.78 (m, 1H), 3.61 (m, 1H), 2.80 (s, 1H), 2.28 (m, 1H), 1.78 (m, 2H), 1.13 (t, *J* = 7.5 Hz, 3H).
^{13}C NMR (126 MHz, Chloroform-*d*) δ 131.8, 128.6, 128. 3, 122.3, 89.3, 85.7, 72.8, 69.4, 30.9, 8.6.
HRMS (ESI) m/z: $[M+H]^+$ Calcd. for $C_{12}H_{14}O_2$ 191.1067; Found: 191.1063.

2-(phenylethynyl)hexane-1,2-diol (8o)

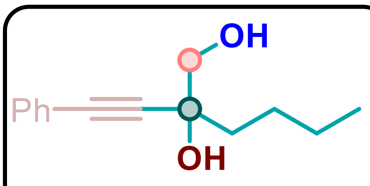

8o, 60%^{b}

Following the **general procedure 8.2** on 0.2 mmol scale, colorless oil, yield: 60% (26.2 mg), R_f = 0.4 (silica gel, PE: EA = 2:1, v/v), column chromatography (silica gel, PE: EA = 5:1, v/v).
^{1}H NMR (500 MHz, Chloroform-*d*) δ 7.48 – 7.40 (m, 2H), 7.31 (d, *J* = 6.5 Hz, 3H), 3.77 (dd, *J* = 11.1, 4.2 Hz, 1H), 3.60 (dd, *J* = 11.0, 8.2 Hz, 1H), 2.86 (s, 1H), 2.34 (dd, *J* = 8.6, 5.0 Hz, 1H), 1.74 (ddd, *J* = 15.4, 7.0, 4.3 Hz, 2H), 1.65 – 1.48 (m, 2H), 1.42 – 1.23 (m, 6H), 0.94 – 0.83 (m, 3H).
^{13}C NMR (126 MHz, Chloroform-*d*) δ 131.80, 128.58, 128.32, 122.29, 89.62, 85.58, 72.37, 69.77, 37.96, 31.76, 29.49, 24.18, 22.62, 14.11.
HRMS (ESI) m/z: $[M+H]^+$ Calcd. for $C_{14}H_{18}O_2$ 219.1380; Found: 219.1382.

4,4-dimethyl-2-(phenylethynyl)pentan-1-ol (9)

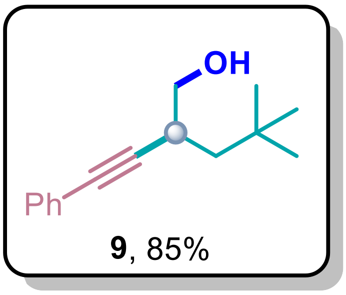

Following the **general procedure 12** on 0.2 mmol scale, colorless oil, yield: 85% (45.9 mg), R_f = 0.4 (silica gel, PE: EA = 10:1, v/v), column chromatography (silica gel, PE: EA = 10:1, v/v).
^{1}H NMR (500 MHz, Chloroform-*d*) δ 7.43 – 7.37 (m, 2H), 7.29 (m, 3H), 3.61 (m, 2H), 2.82 (m, 1H), 2.01 (s, 1H), 1.58 (dd, *J* = 13.7, 10.0 Hz, 1H), 1.38 (dd, *J* = 13.6, 2.8 Hz, 1H), 1.03 (s, 9H).
^{13}C NMR (126 MHz, Chloroform-*d*) δ 131.5, 128.3, 127.9, 123.5, 91.8, 83.4, 66.9, 45.1, 32.2, 30.8, 29.9.
HRMS (ESI) m/z: $[M+H]^+$ Calcd. for $C_{15}H_{20}O$ 217.1587; Found: 217.1586.

(4,4-dimethyl-2-(phenylethynyl)pentyl)trifluoro-l4-borane, potassium salt (10)

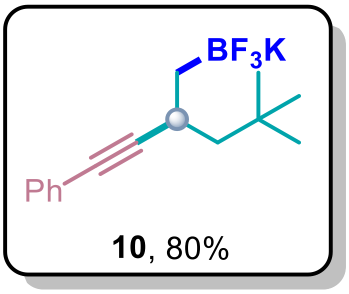

Following the **general procedure 13** on 0.2 mmol scale, white soild, yield: 80% (49.0 mg).
^{1}H NMR (500 MHz, Acetone-*d*$_6$) δ 7.32 – 7.29 (m, 2H), 7.28 – 7.24 (m, 2H), 7.24 – 7.21 (m, 1H), 2.65 (m, 1H), 1.76 (dd, *J* = 13.7, 2.5 Hz, 1H), 1.36 (dd, *J* = 13.6, 10.6 Hz, 1H), 0.99 (s, 12H), 0.67 (m, 1H), 0.52 – 0.40 (m, 1H).
^{13}C NMR (126 MHz, Acetone-*d*$_6$) δ 130.9, 128.2, 126.7, 125.7, 101.3, 78.9, 50.4, 30.7, 29.7, 24.3, 24.3, 24.3.
^{11}B NMR (160 MHz, Acetone-*d*$_6$) δ 5.08.
^{19}F NMR (471 MHz, Acetone-*d*$_6$) δ -138.83 (t, *J* = 54.9 Hz).
HRMS (ESI) m/z: $[M+H]^+$ Calcd. for $C_{15}H_{19}BF_3K$: 307.1242; Found: 307.1238.

(3-neopentylhex-5-en-1-yn-1-yl)benzene (11)

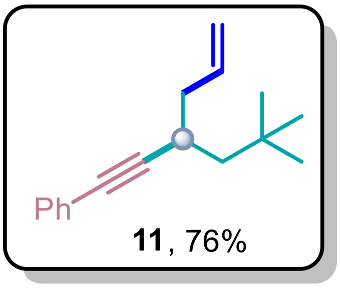

Following the **general procedure 14** on 0.2 mmol scale, colorless oil, yield: 76% (34.4 mg), R_f = 0.4 (silica gel, PE: EA = 100:1, v/v), column chromatography (silica gel, PE: EA = 100:1, v/v).
^{1}H NMR (500 MHz, Chloroform-*d*) δ 7.42 – 7.37 (m, 2H), 7.31 – 7.24 (m, 3H), 5.97 (m, 1H), 5.19 – 5.06 (m, 2H), 2.67 (m, 1H), 2.32 (m, 2H), 1.61 – 1.55 (m, 1H), 1.41 (dd, *J* = 13.7, 2.8 Hz, 1H), 1.04 (s, 9H).
^{13}C NMR (126 MHz, Chloroform-*d*) δ 136.2, 131.4, 128.2, 127.5, 124.3, 116.7, 94.7, 82.1, 48.5, 41.6, 31.0, 30.0, 28.3.
HRMS (ESI) m/z: $[M+H]^+$ Calcd. for $C_{17}H_{22}$ 227.1794; Found: 227.1790.

1-(4,4-dimethyl-2-(phenylethynyl)pentyl)-4-methoxybenzene (12)

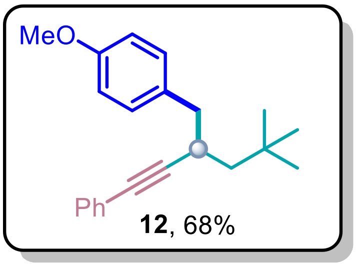

Following the **general procedure 15** on 0.2 mmol scale, colorless oil, yield: 68% (41.6mg), R_f = 0.4 (silica gel, PE: EA = 30:1, v/v), column chromatography (silica gel, PE: EA = 30:1, v/v).
^{1}H NMR (500 MHz, Chloroform-*d*) δ 7.36 – 7.32 (m, 2H), 7.29 – 7.25 (m, 3H), 7.23 – 7.20 (m, 2H), 6.90 – 6.80 (m, 2H), 3.81 (s, 3H), 2.73 (s, 3H), 1.62 – 1.57 (m, 1H), 1.46 – 1.41 (m, 1H), 1.00 (s, 9H).
^{13}C NMR (126 MHz, Chloroform-*d*) δ 158.1, 131.8, 131.3, 130.4, 128.2, 127.4, 124.3, 113.5, 94.9, 82.6, 55.3, 48.4, 42.6, 31.0, 30.8, 29.9.
HRMS (ESI) m/z: $[M+H]^+$ Calcd. for $C_{22}H_{26}O$ 307.2056; Found: 307.2050.

2-(4,4-dimethyl-2-(phenylethynyl)pentyl)naphthalene (13)

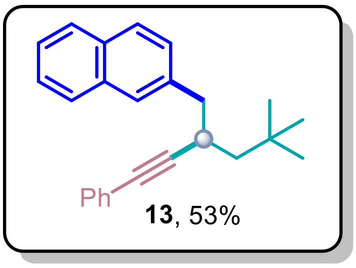

Following the **general procedure 15** on 0.2 mmol scale, colorless oil, yield: 53% (34.6 mg), R_f = 0.4 (silica gel, PE: EA = 30:1, v/v), column chromatography (silica gel, PE: EA = 30:1, v/v).
^{1}H NMR (500 MHz, Chloroform-*d*) δ 7.87 – 7.80 (m, 3H), 7.78 – 7.73 (m, 1H), 7.52 – 7.44 (m, 3H), 7.35 – 7.30 (m, 2H), 7.28 (m, 3H), 3.08 (td, *J* = 9.8, 3.5 Hz, 1H), 3.02 – 2.95 (m, 2H), 1.68 (dd, *J* = 13.6, 9.6 Hz, 1H), 1.53 (dd, *J* = 13.7, 2.3 Hz, 1H), 1.03 (s, 9H).
^{13}C NMR (126 MHz, Chloroform-*d*) δ 137.3, 133.5, 132.3, 131.3, 128.2, 128.1, 127.9, 127.7, 127.6, 127.5, 125.9, 125.2, 124.2, 94.7, 82.7, 48.5, 43.7, 31.0, 30.5, 29.9.
HRMS (ESI) m/z: $[M+H]^+$ Calcd. for : $C_{25}H_{26}$ 327.2107; Found: 327.2101.

2-(4,4-dimethyl-2-(phenylethynyl)pentyl)furan (14)

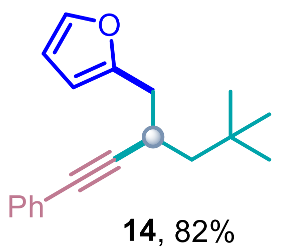

14, 82%

Following the **general procedure 16** on 0.2 mmol scale, colorless oil, yield: 82% (43.6 mg), R_f = 0.4 (silica gel, PE: EA = 100:1, v/v), column chromatography (silica gel, PE: EA = 100:1, v/v).

^{1}H NMR (500 MHz, Chloroform-*d*) δ 7.34 (ddd, *J* = 4.3, 3.3, 2.2 Hz, 3H), 7.26 (h, *J* = 3.0 Hz, 3H), 6.31 (dd, *J* = 3.2, 1.9 Hz, 1H), 6.16 (dd, *J* = 3.1, 0.9 Hz, 1H), 2.97 – 2.90 (m, 2H), 2.86 – 2.77 (m, 1H), 1.64 – 1.58 (m, 1H), 1.43 (dd, *J* = 13.6, 2.4 Hz, 1H), 1.00 (s, 9H).

^{13}C NMR (126 MHz, Chloroform-*d*) δ 153.69, 141.14, 131.35, 128.16, 127.50, 124.11, 110.19, 106.76, 94.26, 82.09, 48.28, 35.80, 30.99, 29.83, 28.01.

HRMS (ESI) m/z: $[M+H]^+$ Calcd. for $C_{19}H_{22}O$ 267.1743; Found: 267.1740.

2-(4,4-dimethyl-2-(phenylethynyl)pentyl)thiophene (15)

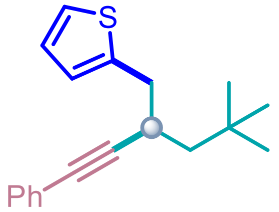

15, 79%

Following the **general procedure 17** on 0.2 mmol scale, colorless oil, yield: 79% (44.6 mg), R_f = 0.4 (silica gel, PE: EA = 100:1, v/v), column chromatography (silica gel, PE: EA = 100:1, v/v).

^{1}H NMR (500 MHz, Chloroform-*d*) δ 7.44 – 7.39 (m, 2H), 7.33 – 7.28 (m, 3H), 7.19 (dd, *J* = 5.1, 1.2 Hz, 1H), 6.98 (dd, *J* = 5.1, 3.4 Hz, 1H), 6.95 (dd, *J* = 3.4, 1.1 Hz, 1H), 3.09 (m, 2H), 2.94 – 2.85 (m, 1H), 1.64 (dd, *J* = 13.7, 10.2 Hz, 1H), 1.53 – 1.46 (m, 1H), 1.04 (s, 10H).

^{13}C NMR (126 MHz, Chloroform-*d*) δ 142.0, 131.4, 128.2, 127.6, 126.5, 125.9, 124.1, 123.9, 94.4, 82.9, 48.3, 37.7, 31.0, 30.9, 30.0.

HRMS (ESI) m/z: $[M+H]^+$ Calcd. for $C_{19}H_{22}S$ 283.1515; Found: 283.1519.

2-(4,4-dimethyl-2-phenethylpentyl)-4,4,5,5-tetramethyl-1,3,2-dioxaborolane (16)

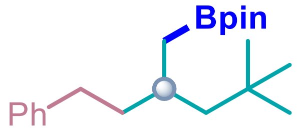

16, 90%

Following the **general procedure 18** on 0.2 mmol scale, colorless oil, yield: 90% (59.4 mg), R_f = 0.4 (silica gel, PE: EA = 30:1, v/v), column chromatography (silica gel, PE: EA = 30:1, v/v).

^{1}H NMR (500 MHz, Chloroform-*d*) δ 7.29 – 7.25 (m, 2H), 7.21 – 7.14 (m, 3H), 2.61 (m, 2H), 1.79 – 1.72 (m, 1H), 1.67 (m, 1H), 1.62 – 1.52 (m, 1H), 1.32 (dd, *J* = 14.1, 5.0 Hz, 1H), 1.26 (d, *J* = 1.9 Hz, 14H), 1.20 (dd, *J* = 14.1, 5.1 Hz, 1H), 0.92 (s, 9H).

^{13}C NMR (126 MHz, Chloroform-*d*) δ 143.4, 128.4, 128.2, 125.5, 82.9, 82.8, 50.9, 40.9, 33.5, 31.3, 30.7, 30.2, 29.5, 25.0, 24.9, 24.9.

^{11}B NMR (160 MHz, Chloroform-*d*) δ 34.03.

HRMS (ESI) m/z: $[M+H]^+$ Calcd. for $C_{21}H_{35}BO_2$ 331.2803; Found: 331.2795.

2.6 NMR spectroscopic data

1,3-dioxoisoindolin-2-yl 5,5-dimethyl-3-(4,4,5,5-tetramethyl-1,3,2-dioxaborolan-2-yl)hexanoate (1a)

^{1}H NMR (500 MHz, Chloroform-*d*)

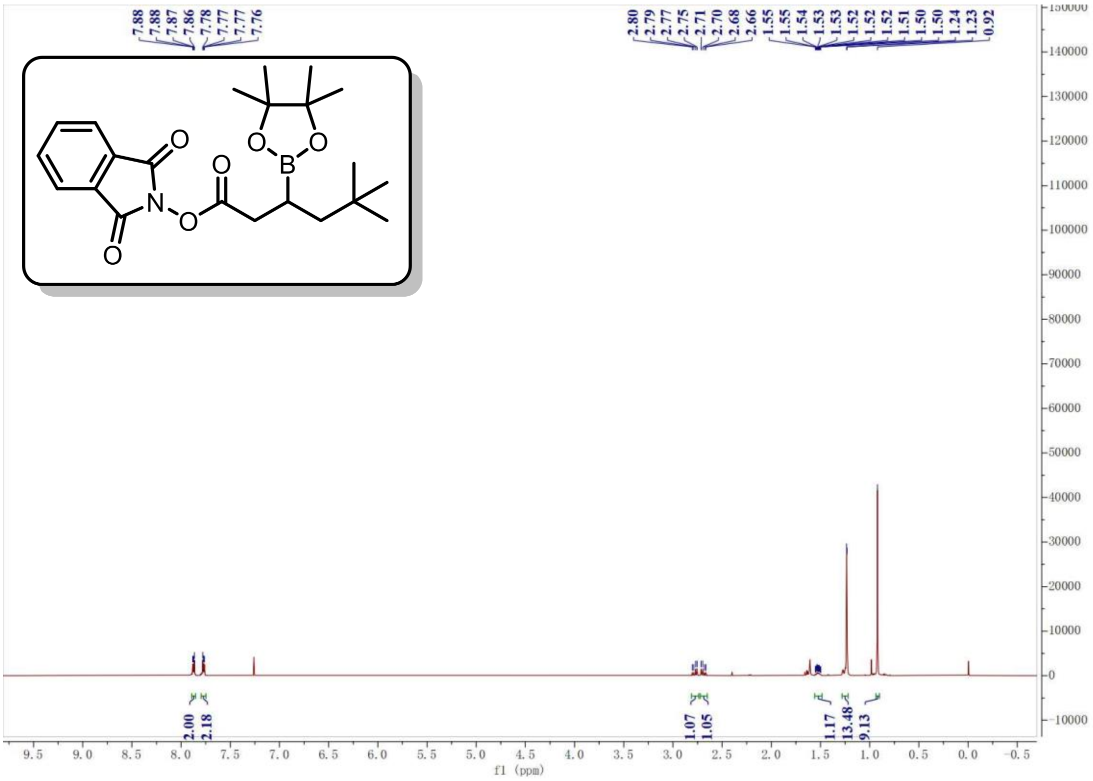

^{13}C NMR (126 MHz, Chloroform-*d*)

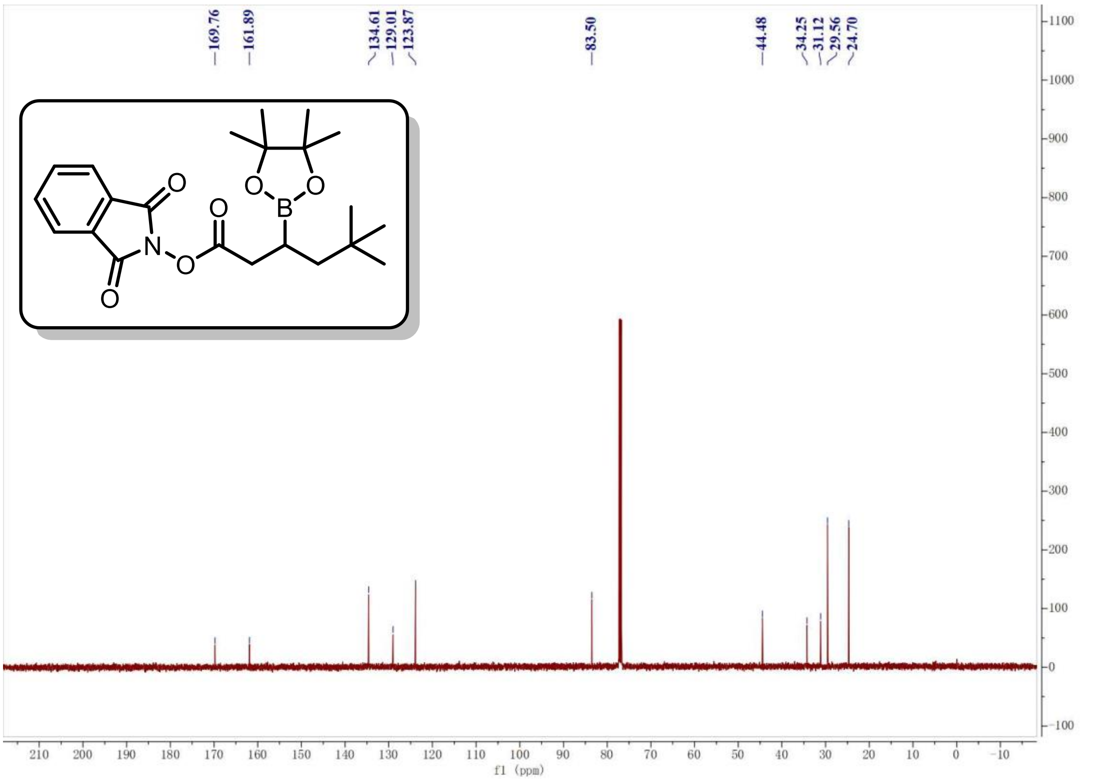

^{11}B NMR (160 MHz, Chloroform-*d*)

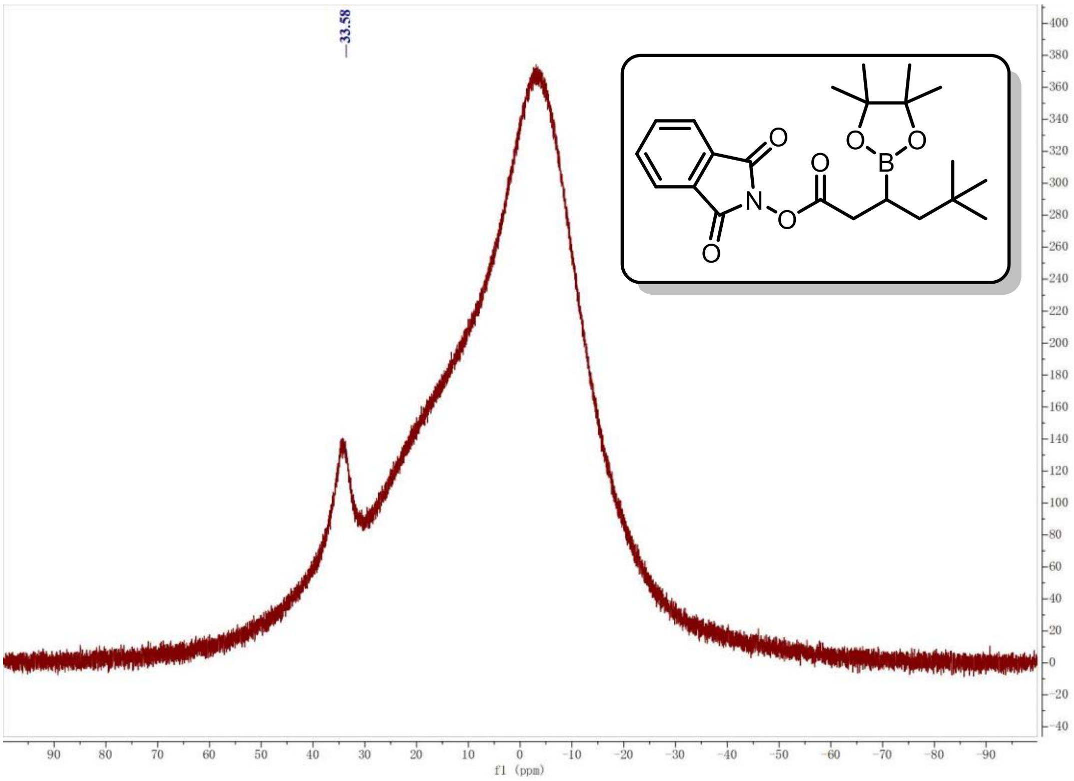

1,3-dioxoisoindolin-2-yl 3-cyclohexyl-3-(4,4,5,5-tetramethyl-1,3,2-dioxaborolan-2-yl)propanoate (1b)

^{1}H NMR (500 MHz, Chloroform-*d*)

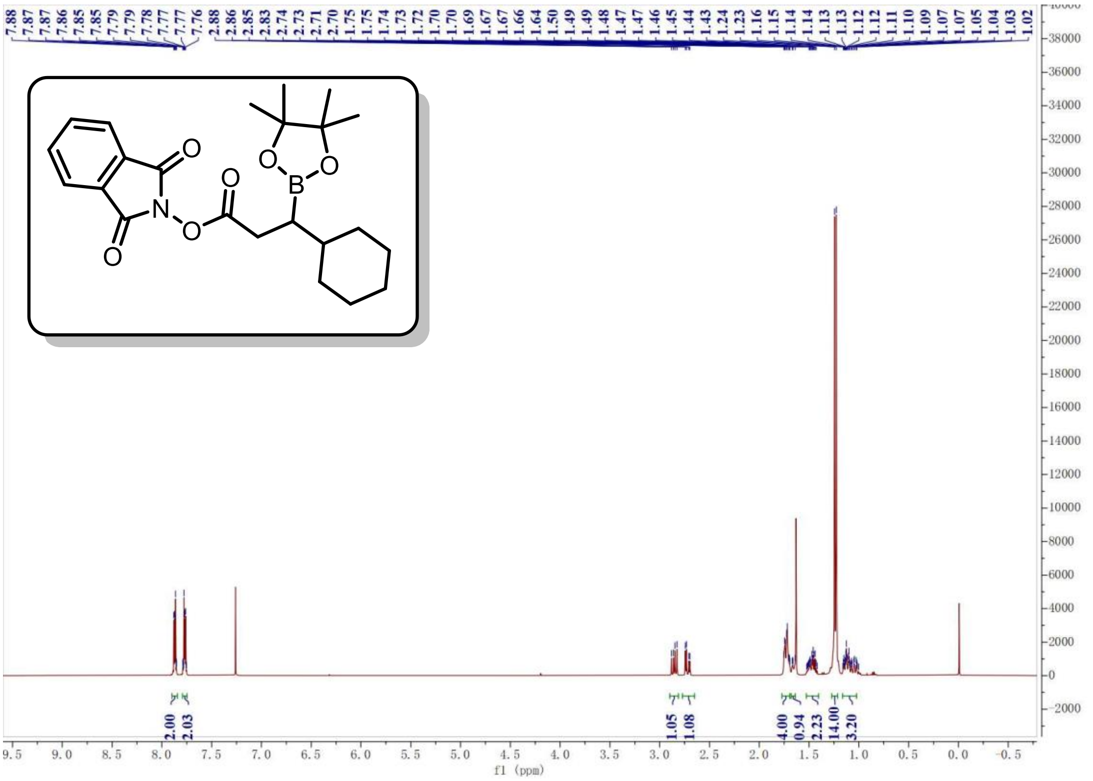

^{13}C NMR (126 MHz, Chloroform-*d*)

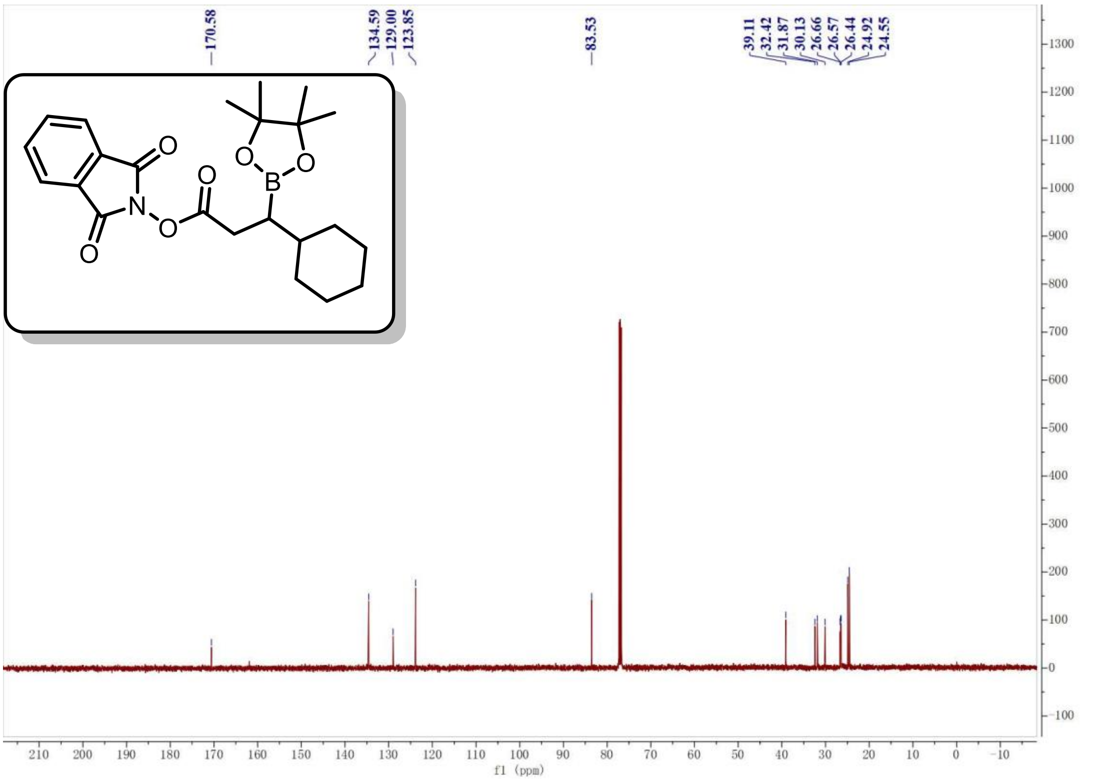

^{11}B NMR (160 MHz, Chloroform-*d*)

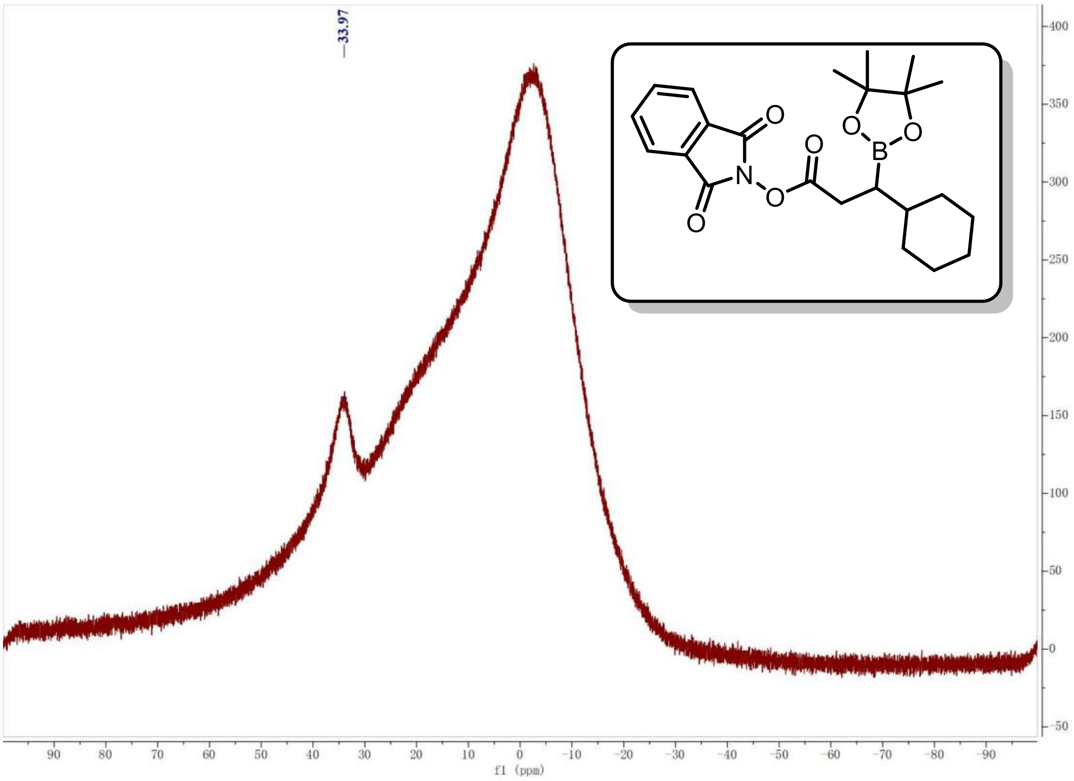

1,3-dioxoisoindolin-2-yl 3-cyclopentyl-3-(4,4,5,5-tetramethyl-1,3,2-dioxaborolan-2-yl)propanoate (1c)

^{1}H NMR (500 MHz, Chloroform-*d*)

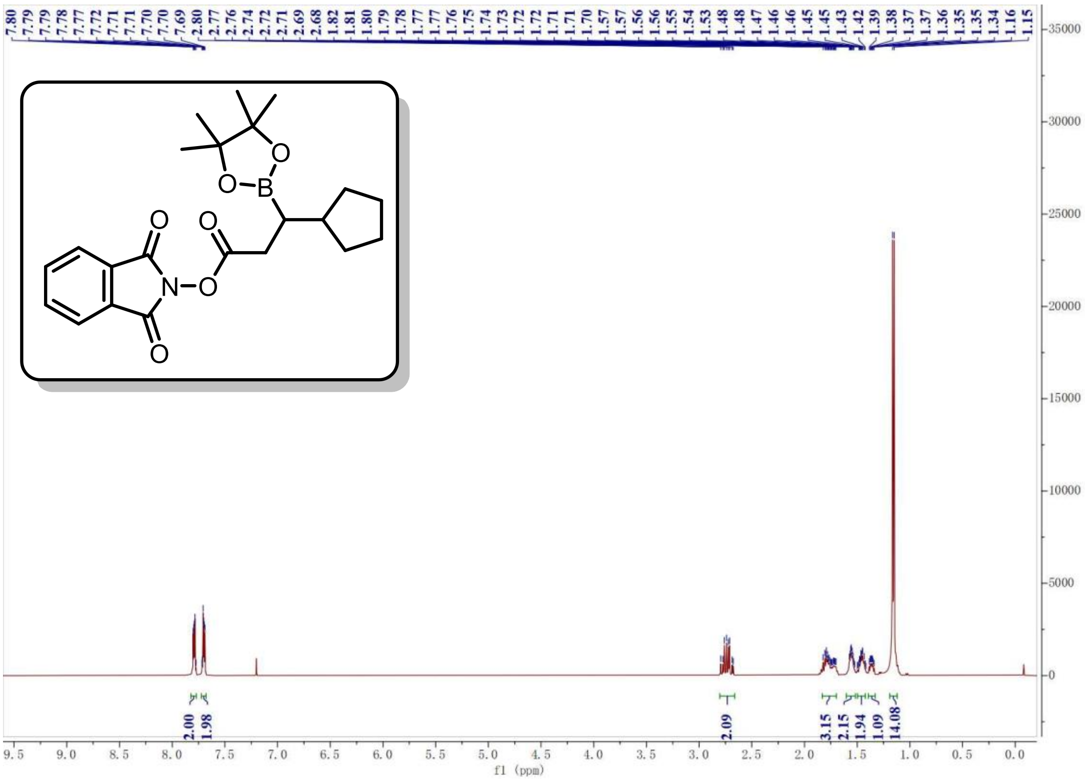

^{13}C NMR (126 MHz, Chloroform-*d*)

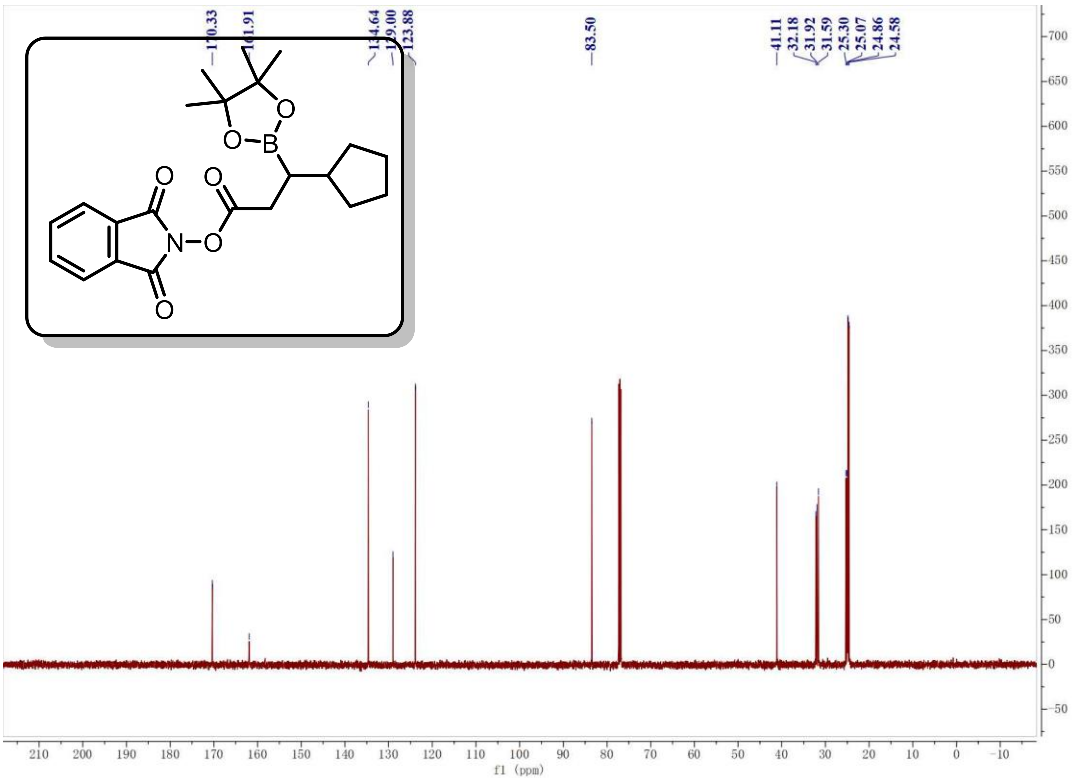

^{11}B NMR (160 MHz, Chloroform-*d*)

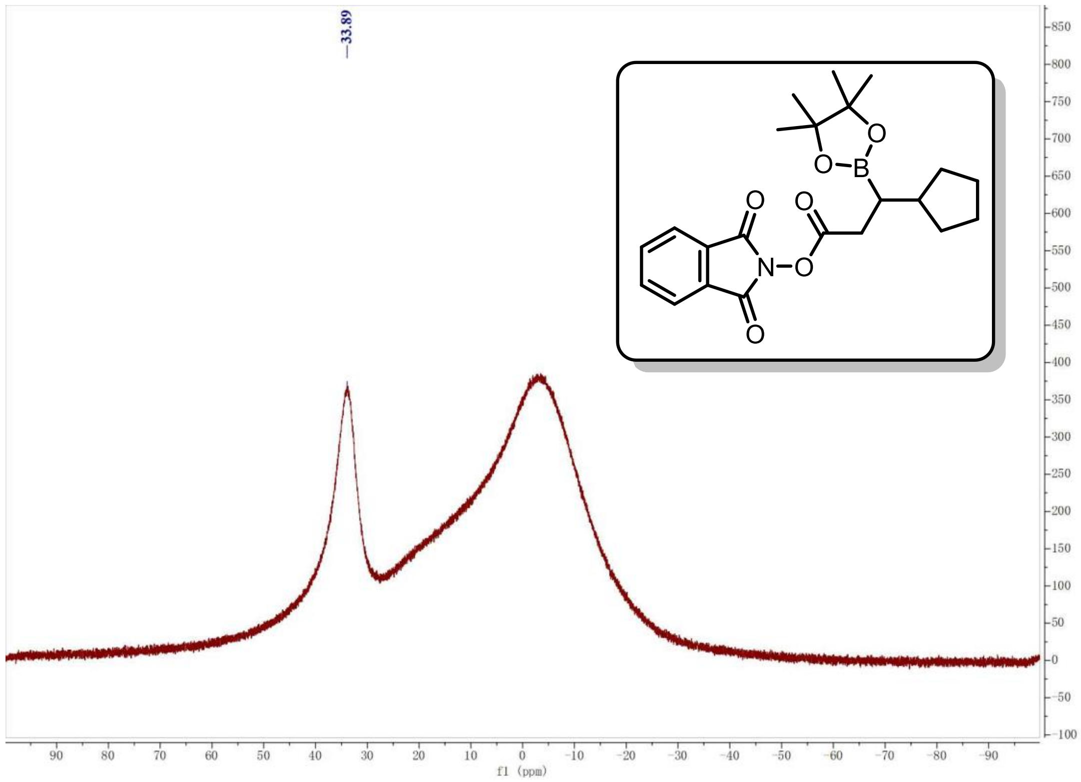

1,3-dioxoisoindolin-2-yl 5-phenyl-3-(4,4,5,5-tetramethyl-1,3,2-dioxaborolan-2-yl)pentanoate (1d)

^{1}H NMR (500 MHz, Chloroform-*d*)

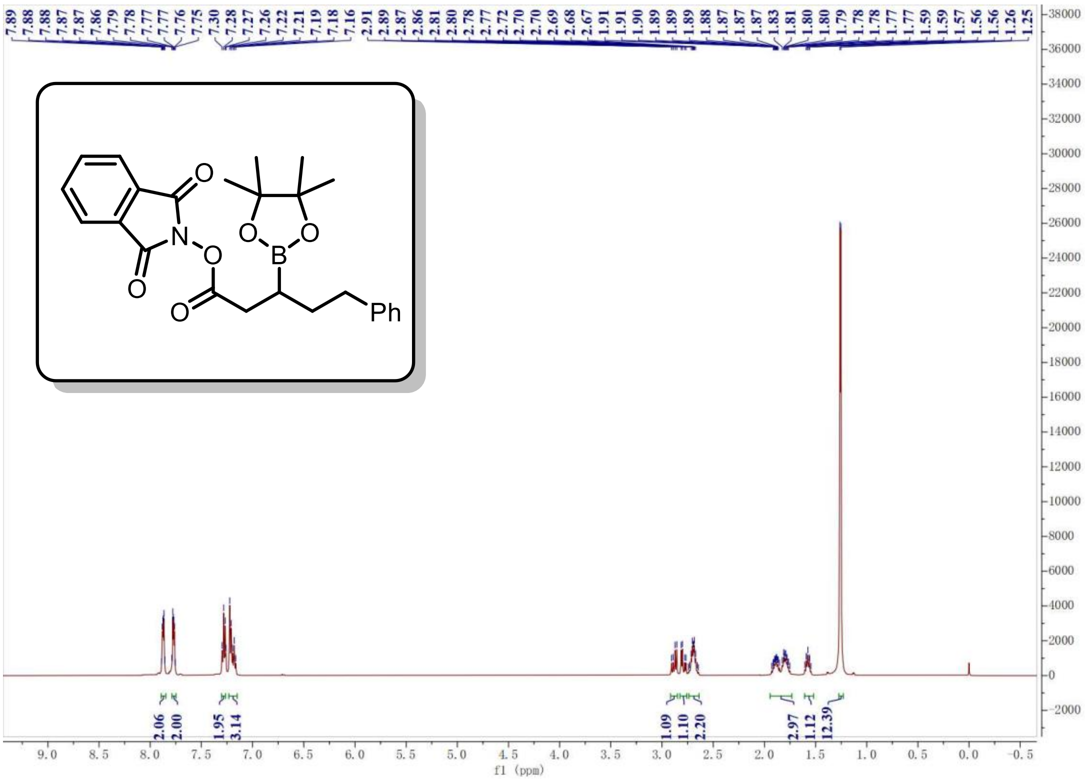

^{13}C NMR (126 MHz, Chloroform-*d*)

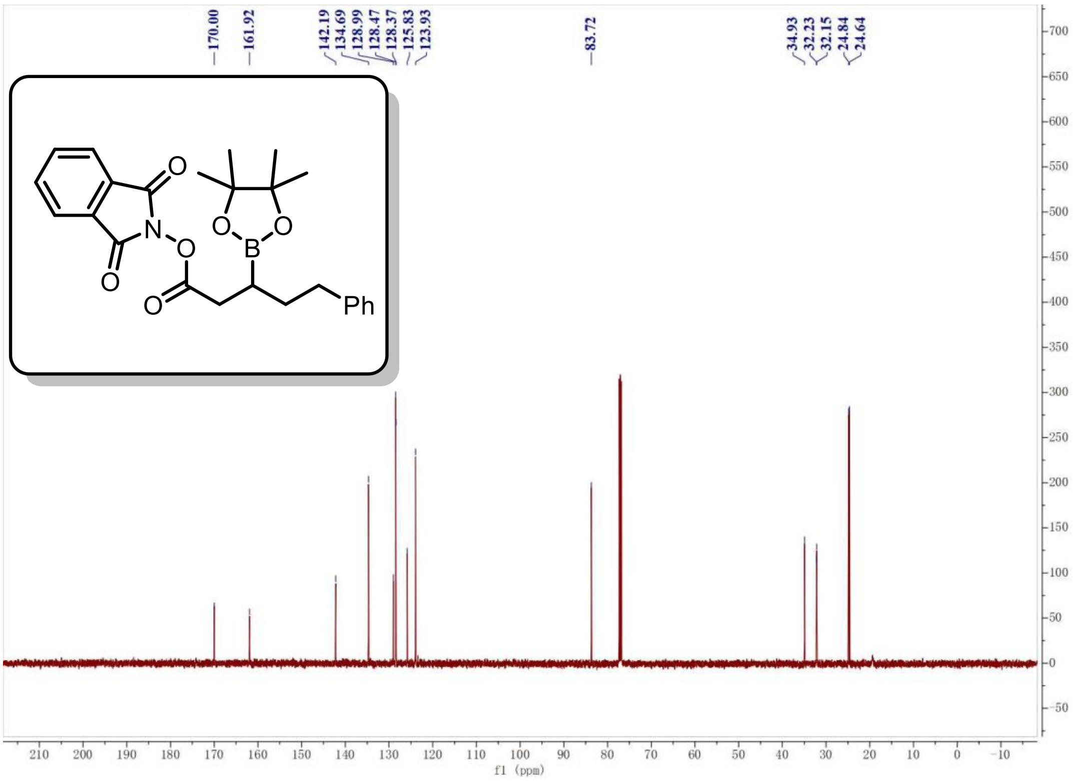

^{11}B NMR (160 MHz, Chloroform-*d*)

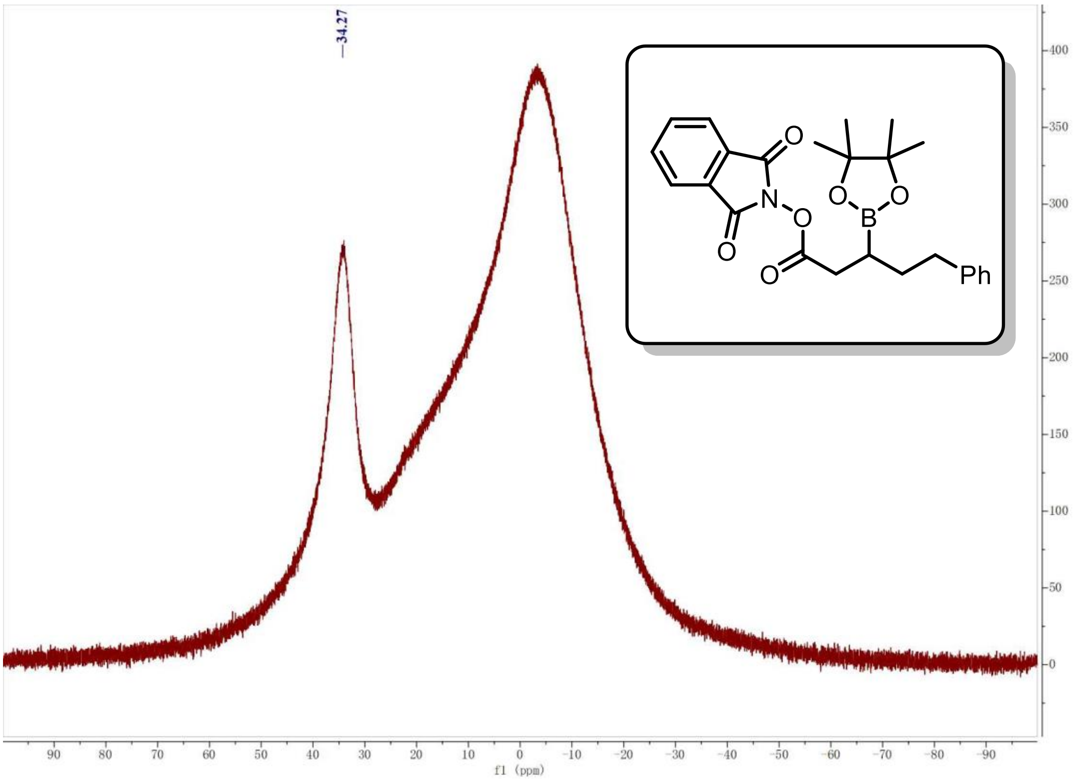

1,3-dioxoisoindolin-2-yl 3-(4,4,5,5-tetramethyl-1,3,2-dioxaborolan-2-yl)butanoate (1e)

^{1}H NMR (500 MHz, Chloroform-*d*)

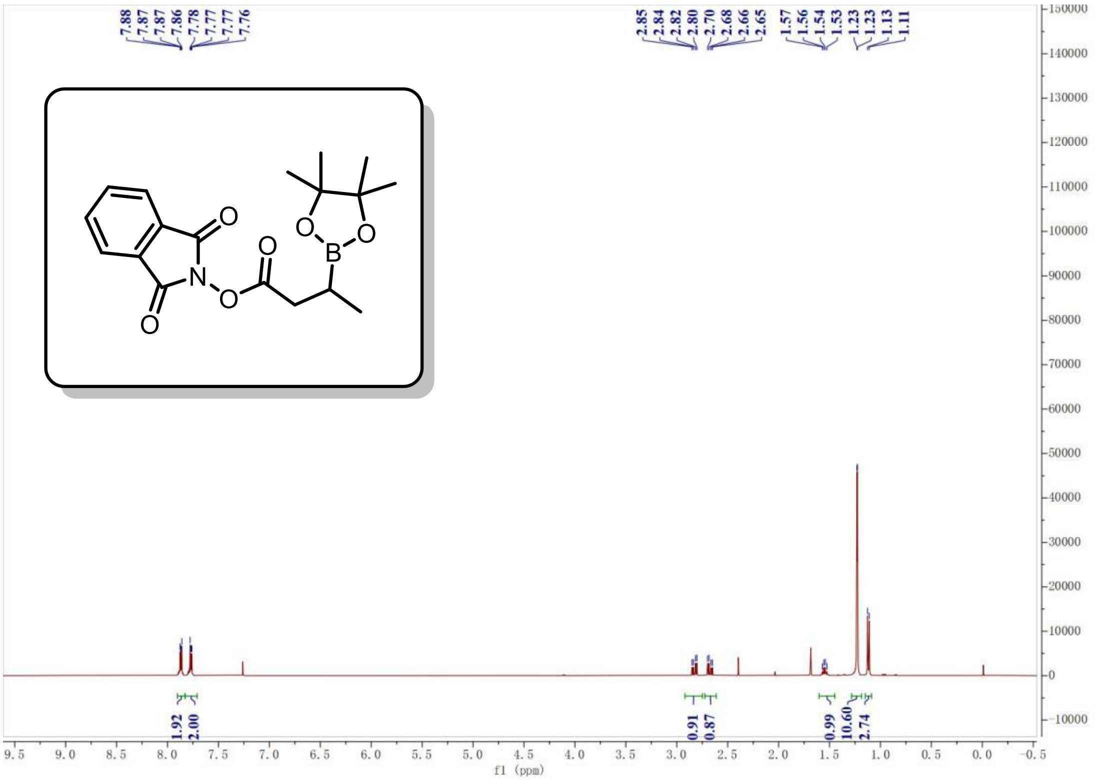

^{13}C NMR (126 MHz, Chloroform-*d*)

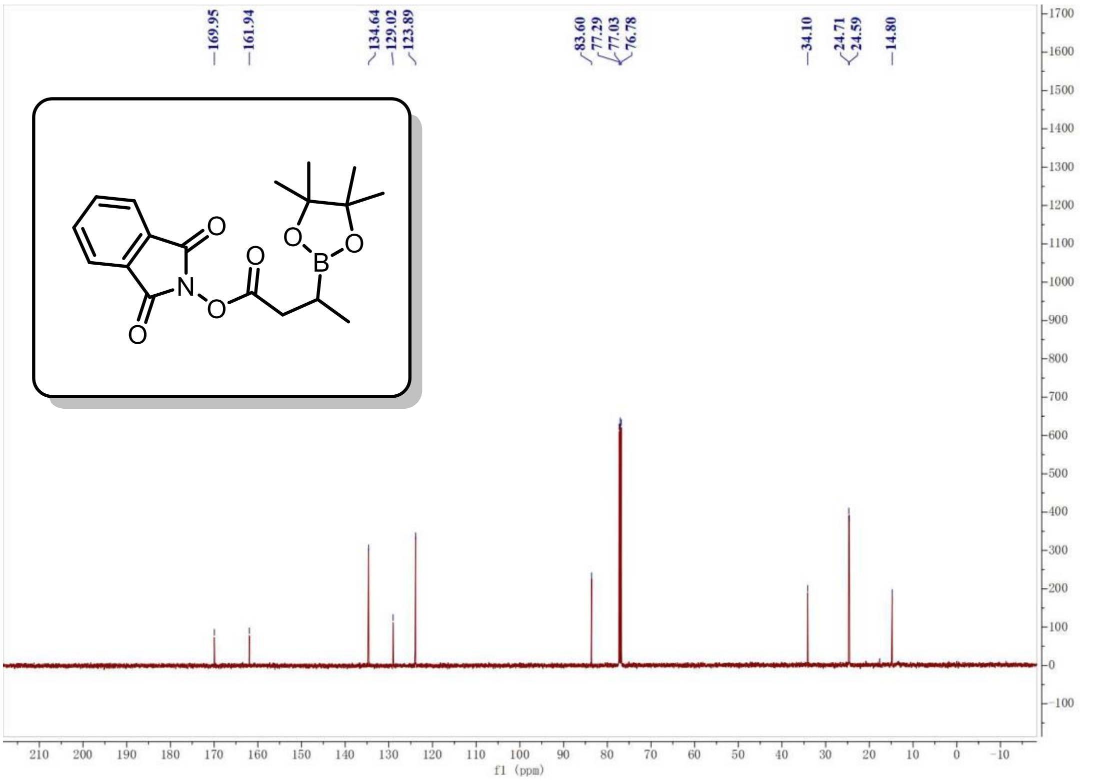

^{11}B NMR (160 MHz, Chloroform-*d*)

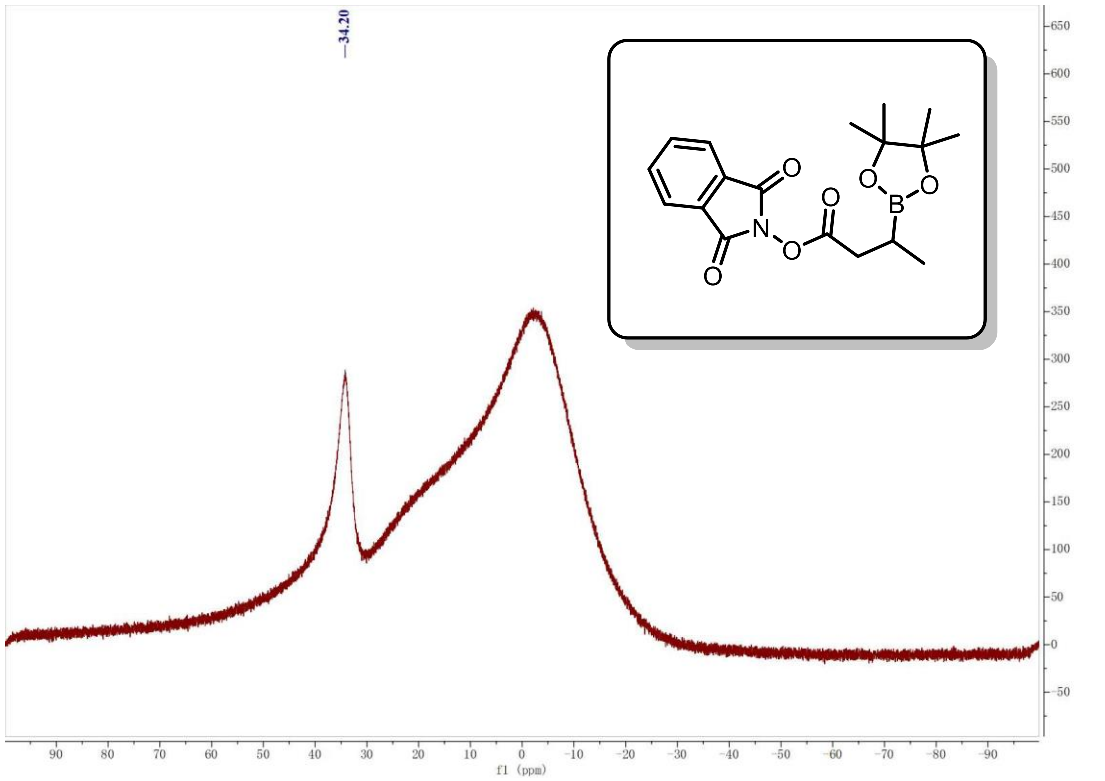

1,3-dioxoisoindolin-2-yl 4-methyl-3-(4,4,5,5-tetramethyl-1,3,2-dioxaborolan-2-yl)heptanoate (1f)

^{1}H NMR (500 MHz, Chloroform-*d*)

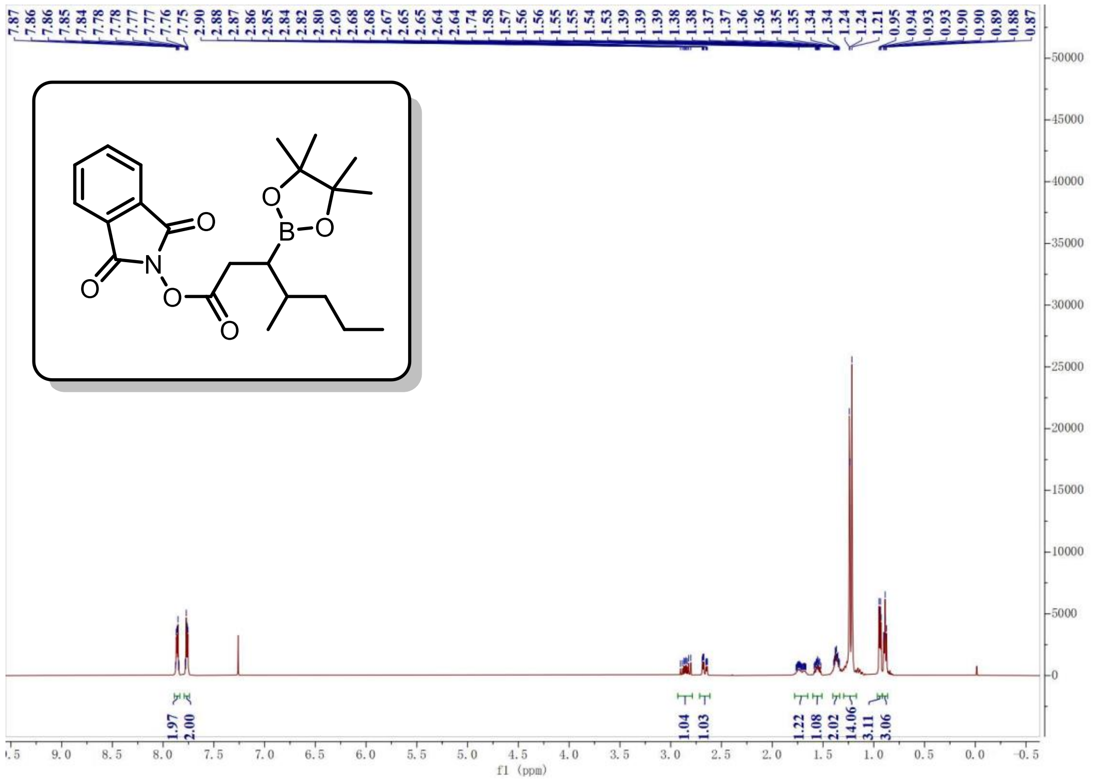

^{13}C NMR (126 MHz, Chloroform-*d*)

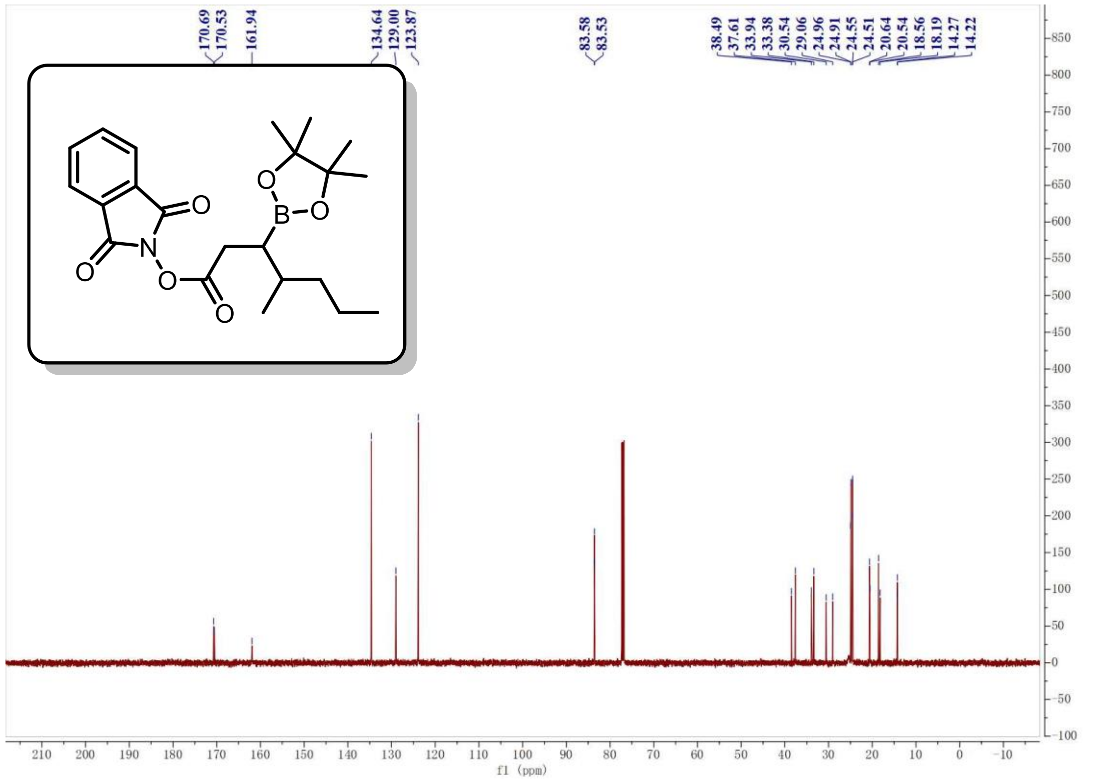

^{11}B NMR (160 MHz, Chloroform-*d*)

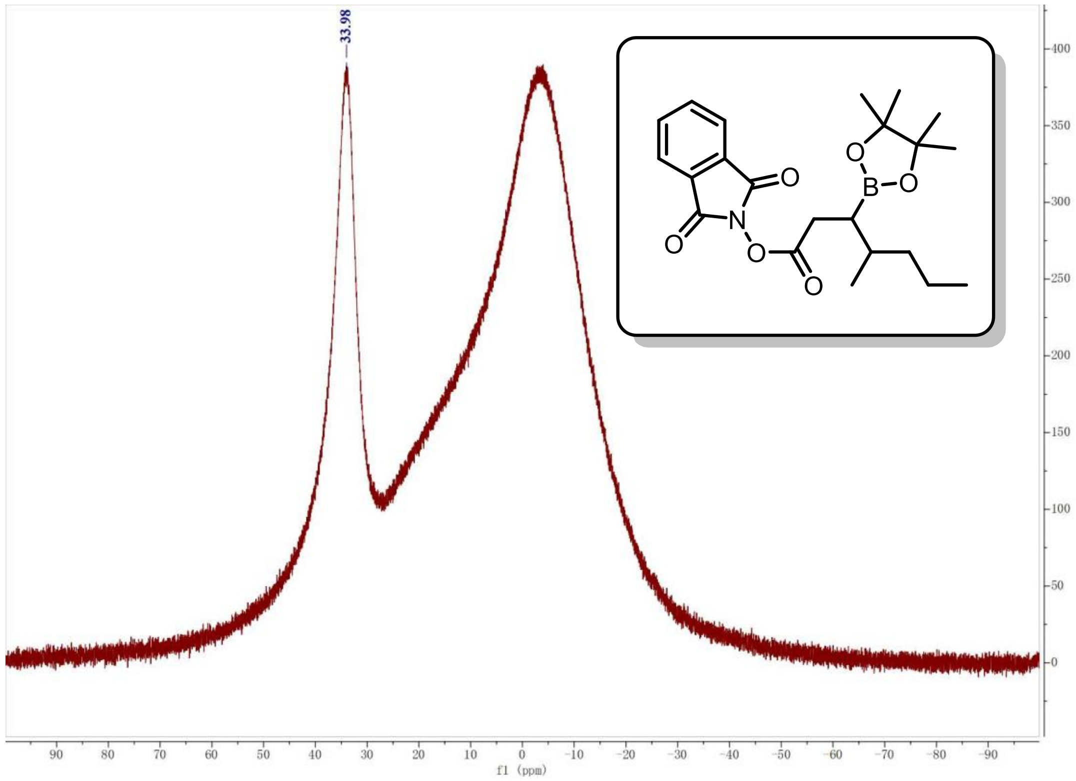

1,3-dioxoisoindolin-2-yl 5-methyl-3-(4,4,5,5-tetramethyl-1,3,2-dioxaborolan-2-yl)hexanoate (1g)

^{1}H NMR (500 MHz, Chloroform-*d*)

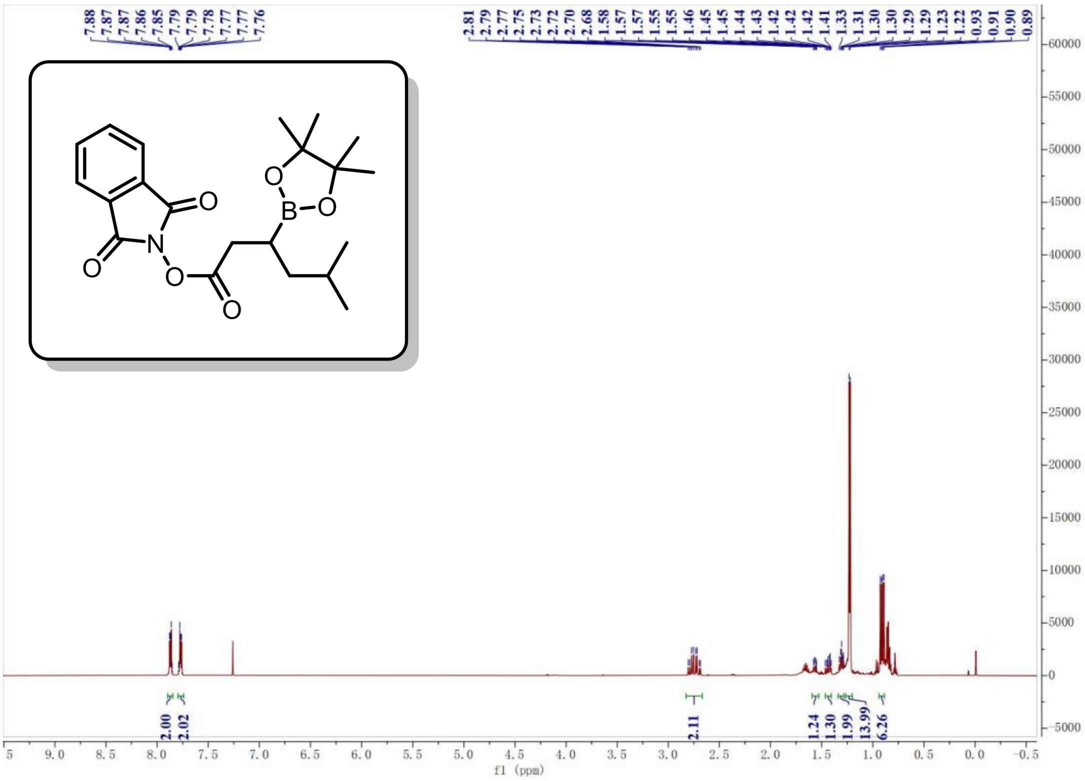

^{13}C NMR (126 MHz, Chloroform-*d*)

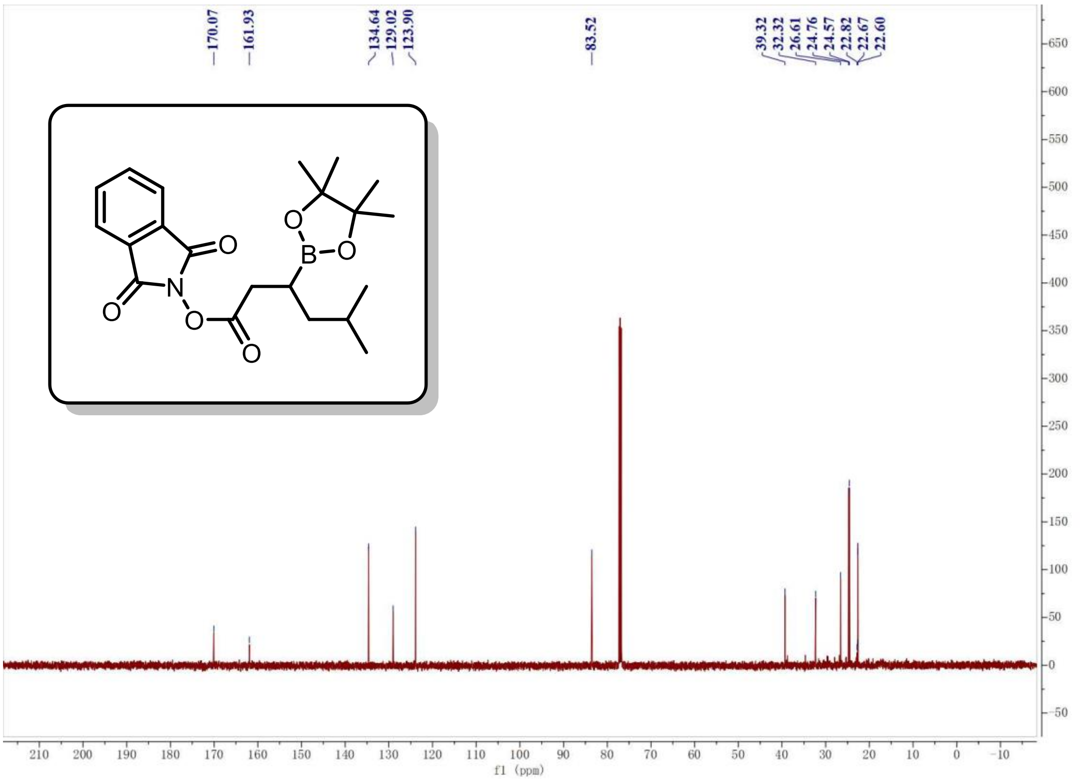

^{11}B NMR (160 MHz, Chloroform-*d*)

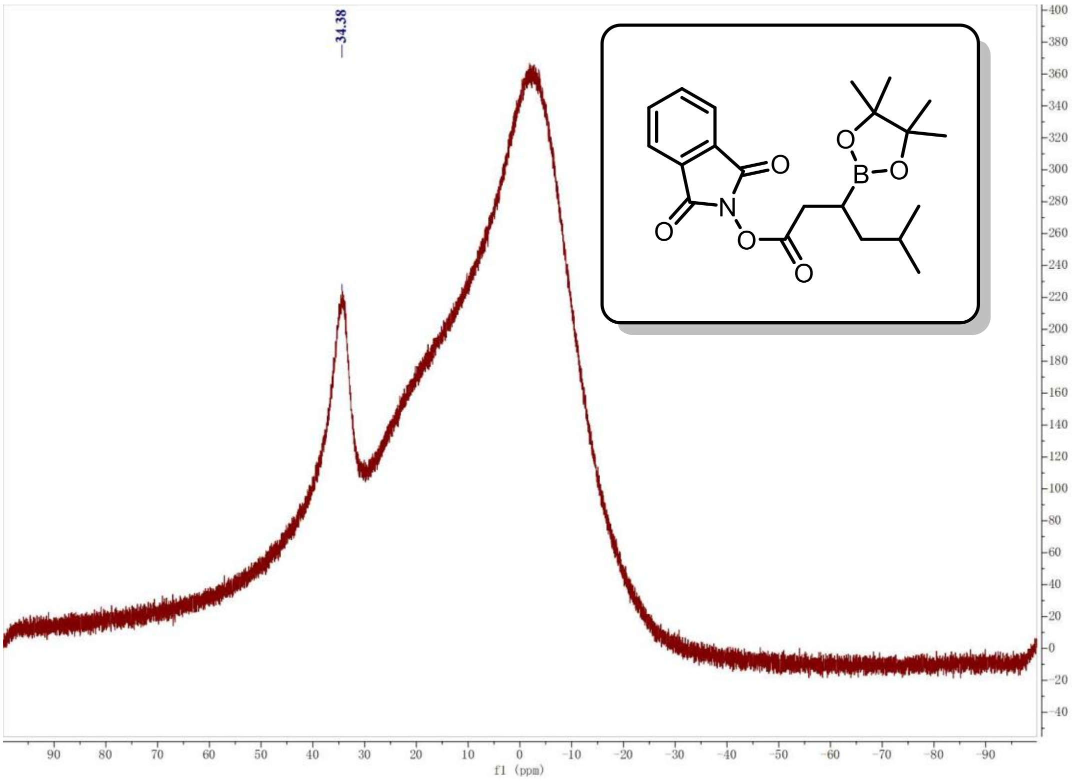

1,3-dioxoisoindolin-2-yl 5,10-dimethyl-3-(4,4,5,5-tetramethyl-1,3,2-dioxaborolan-2-yl)undec-9-enoate (1h)

^{1}H NMR (500 MHz, Chloroform-*d*)

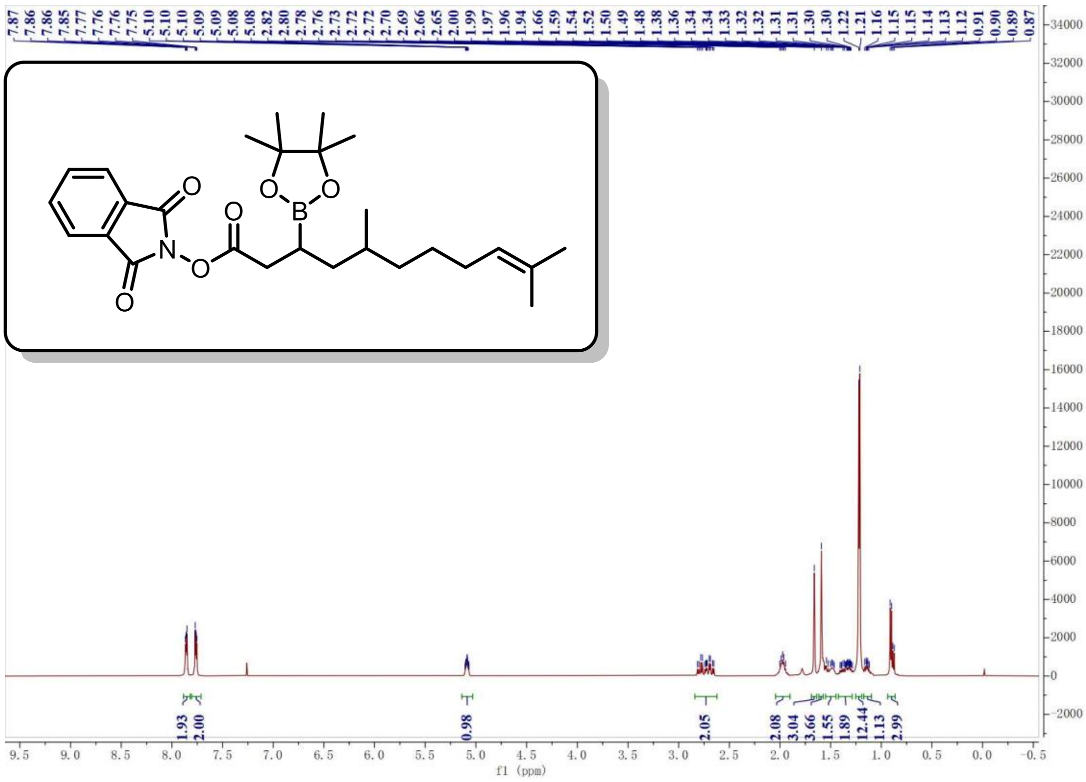

^{13}C NMR (126 MHz, Chloroform-*d*)

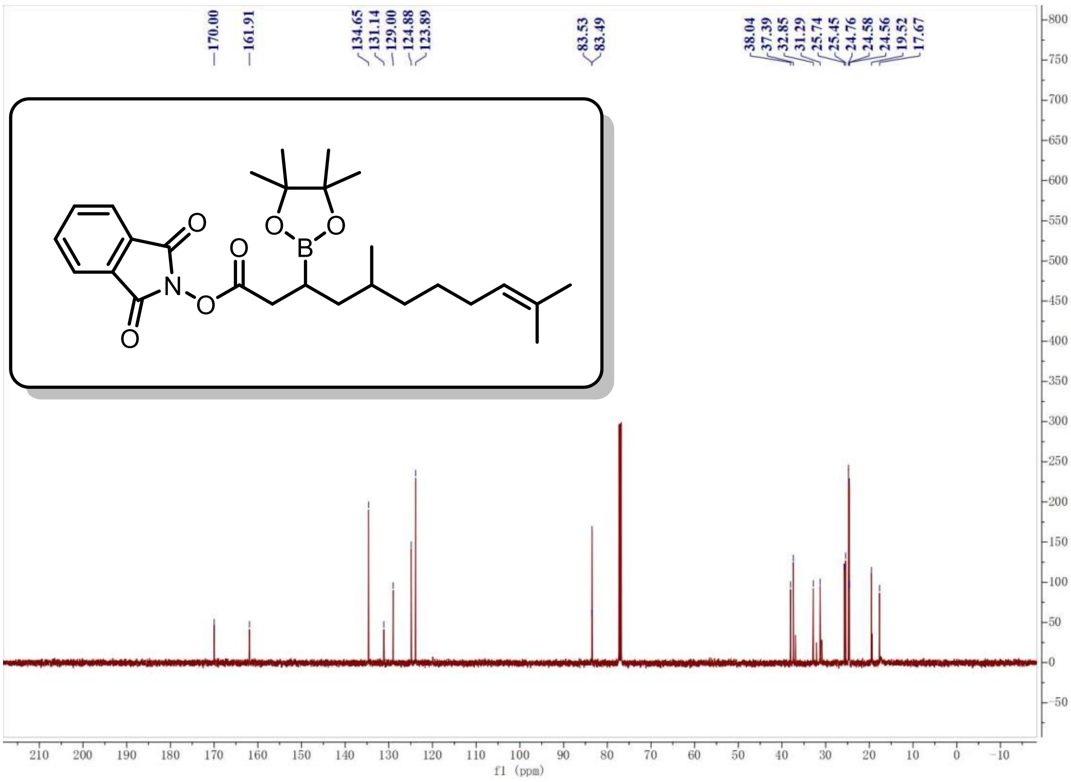

^{11}B NMR (160 MHz, Chloroform-*d*)

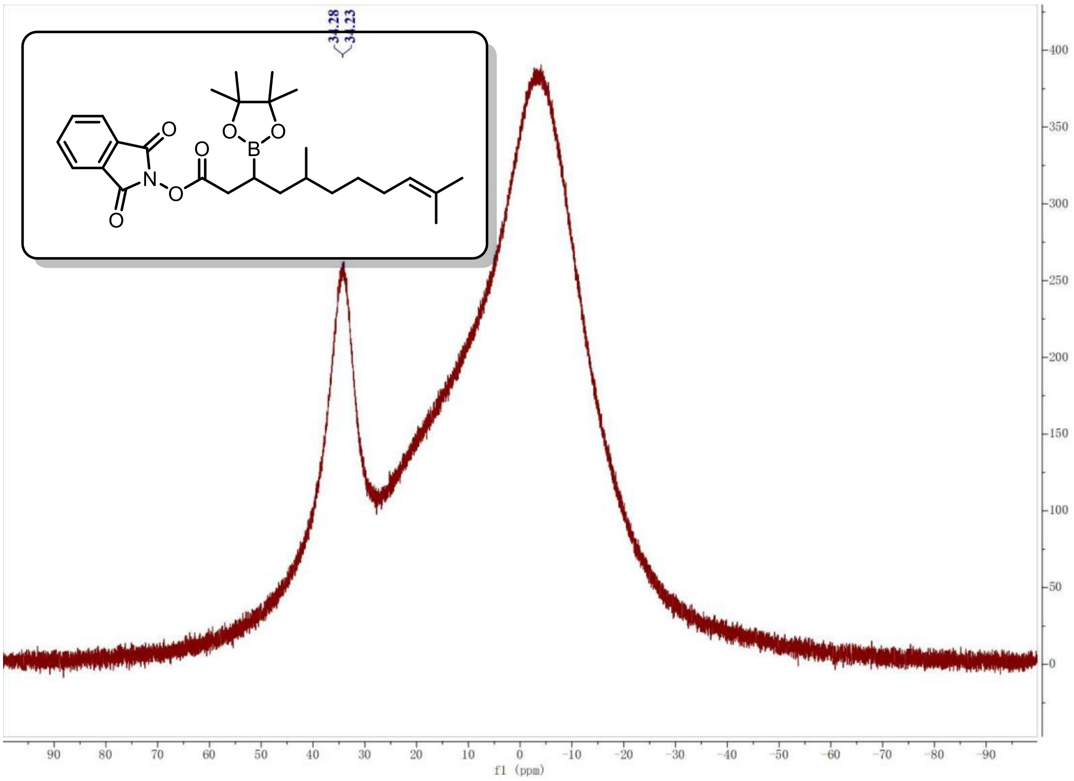

1,3-dioxoisoindolin-2-yl 5-(4-isopropylphenyl)-4-methyl-3-(4,4,5,5-tetramethyl-1,3,2-dioxaborolan-2-yl)pentanoate (1i)

^{1}H NMR (500 MHz, Chloroform-*d*)

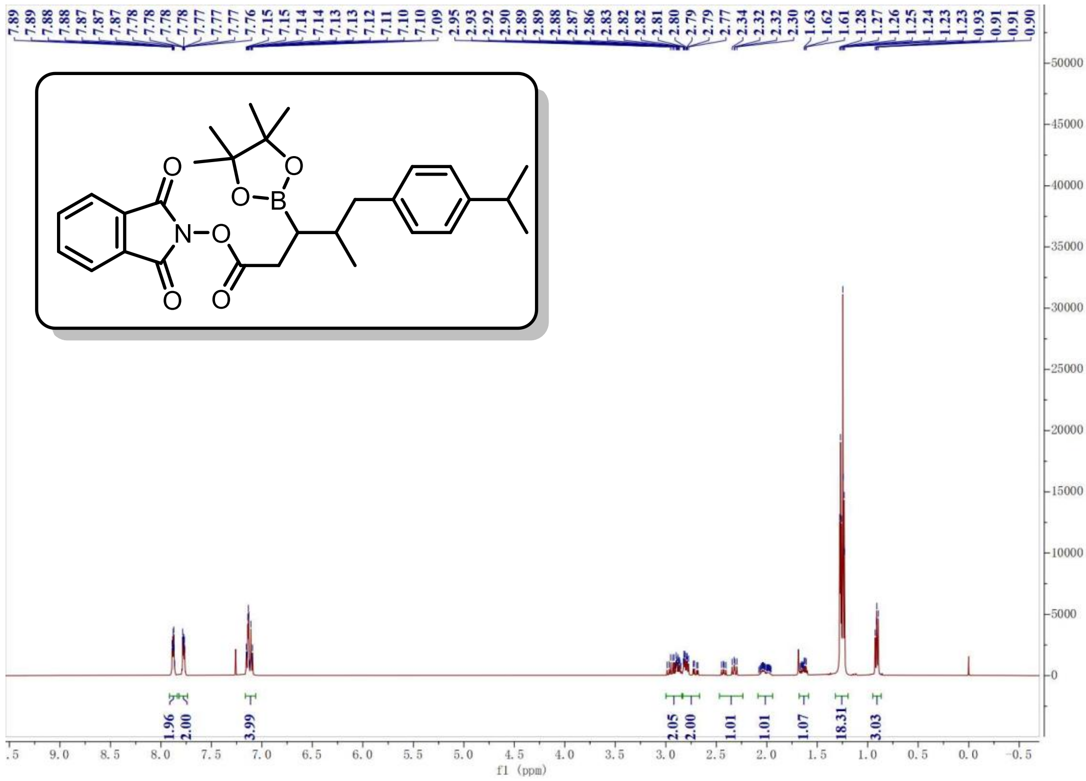

^{13}C NMR (126 MHz, Chloroform-*d*)

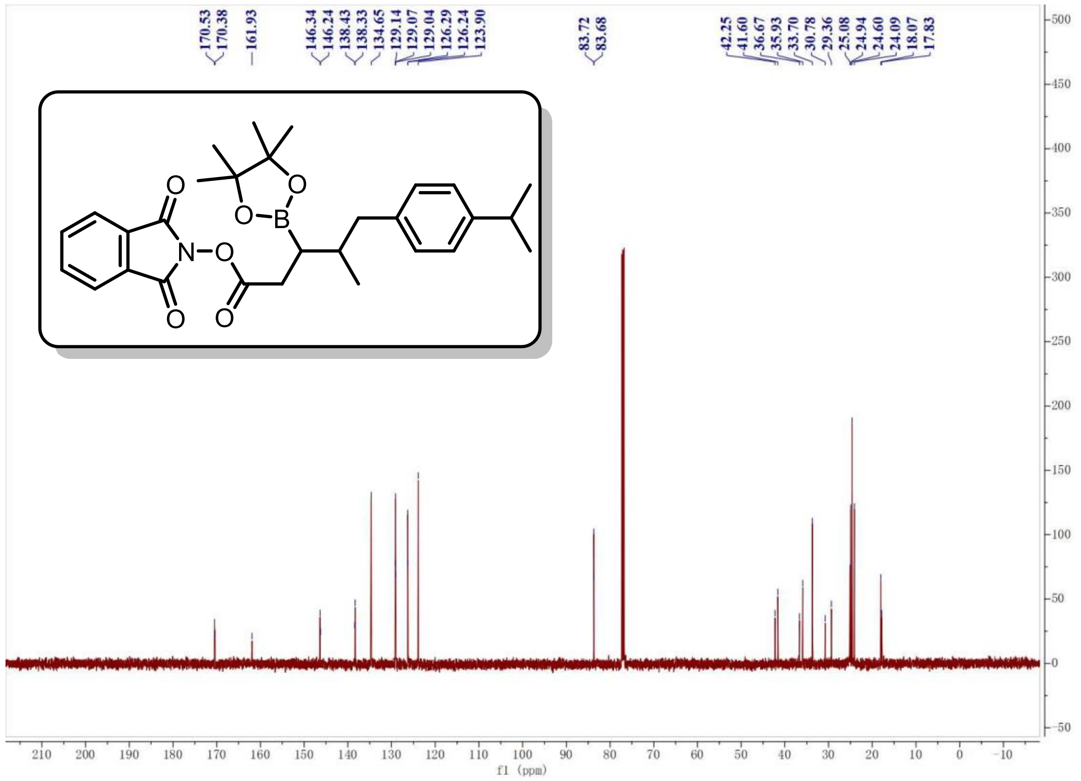

^{11}B NMR (160 MHz, Chloroform-*d*)

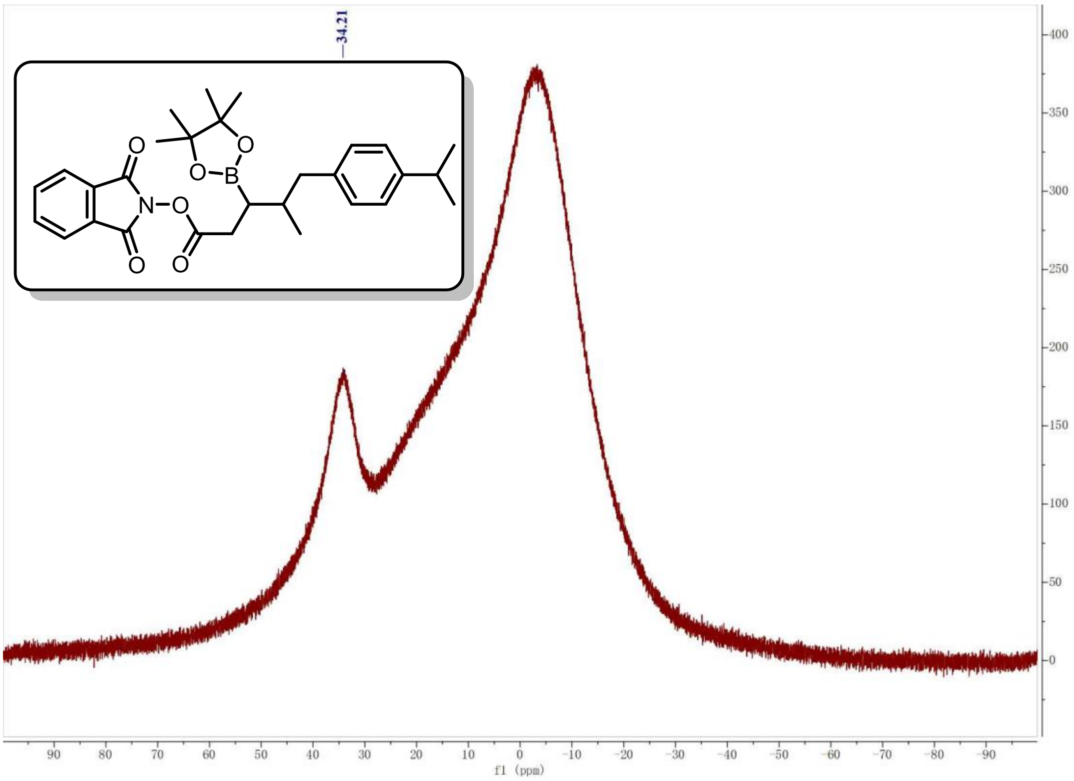

1,3-dioxoisoindolin-2-yl 3-(4,4,5,5-tetramethyl-1,3,2-dioxaborolan-2-yl)pentadecanoate (1j)

^{1}H NMR (500 MHz, Chloroform-*d*)

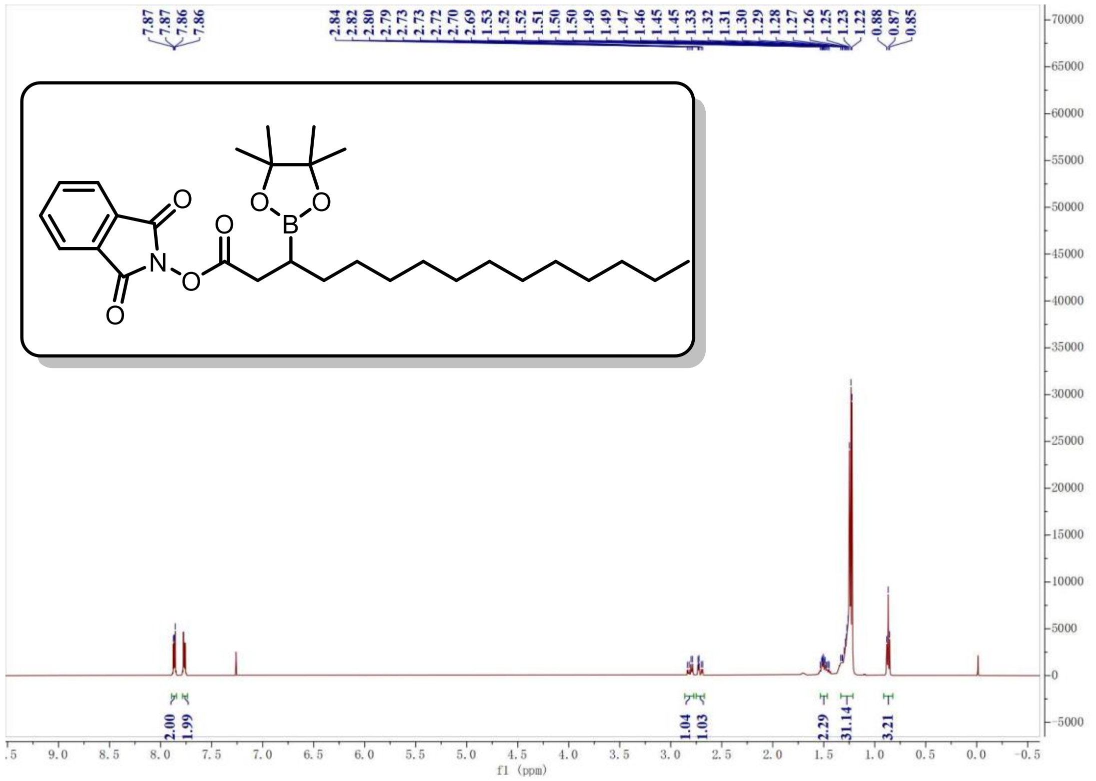

^{13}C NMR (126 MHz, Chloroform-*d*)

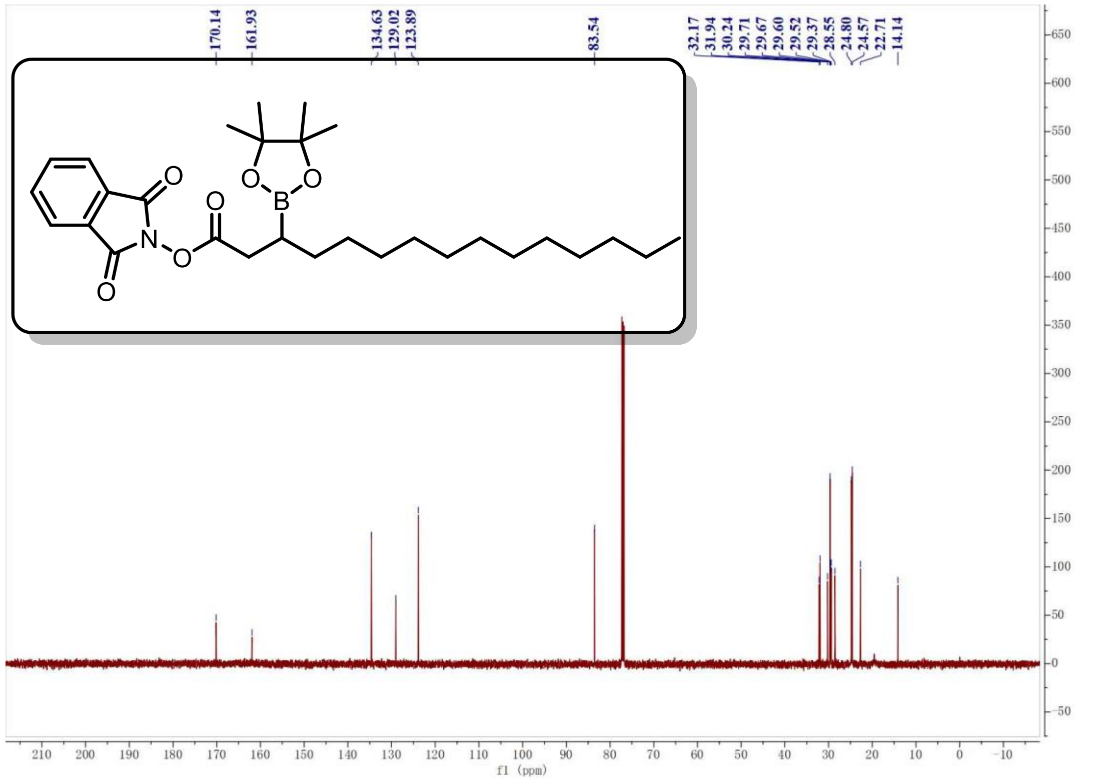

^{11}B NMR (160 MHz, Chloroform-*d*)

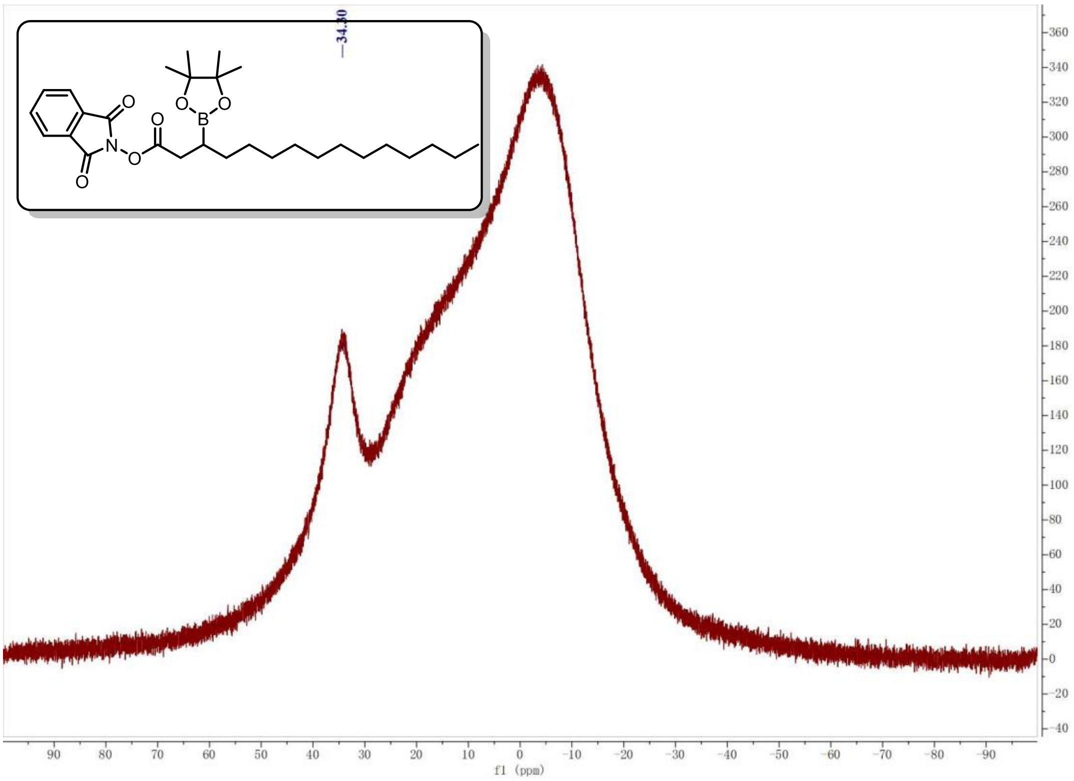

1,3-dioxoisoindolin-2-yl 5-(4-(tert-butyl)phenyl)-4-methyl-3-(4,4,5,5-tetramethyl-1,3,2-dioxaborolan-2-yl)pentanoate (1k)

^{1}H NMR (500 MHz, Chloroform-*d*)

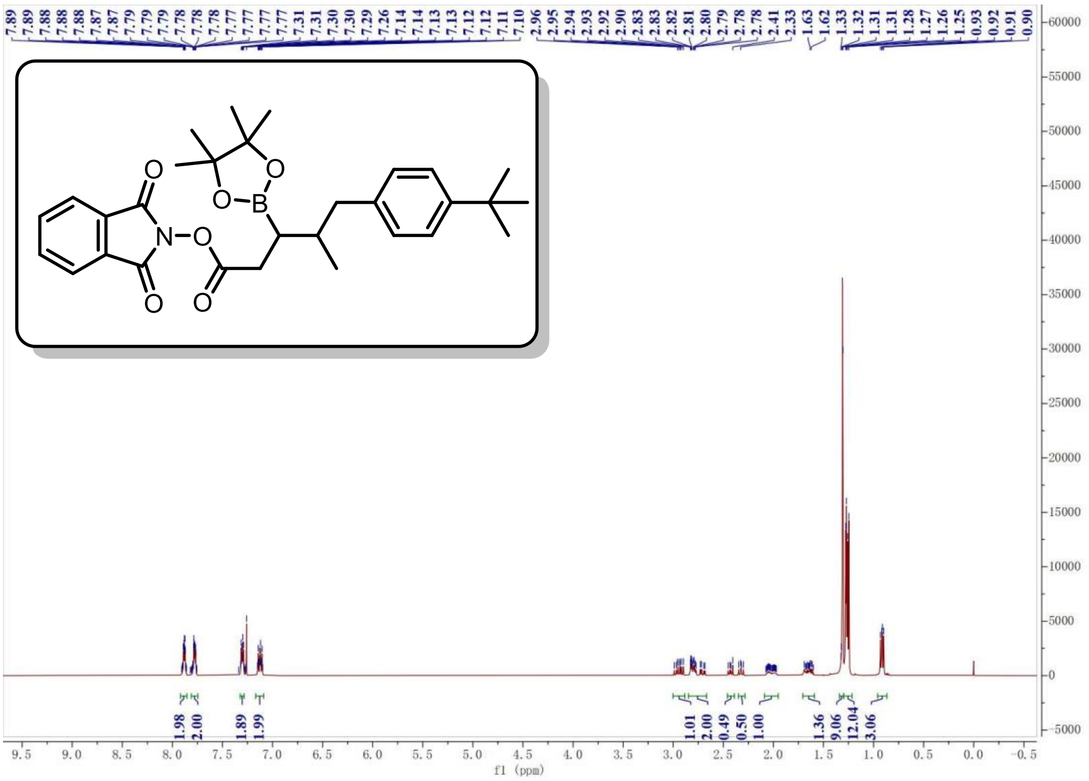

^{13}C NMR (126 MHz, Chloroform-*d*)

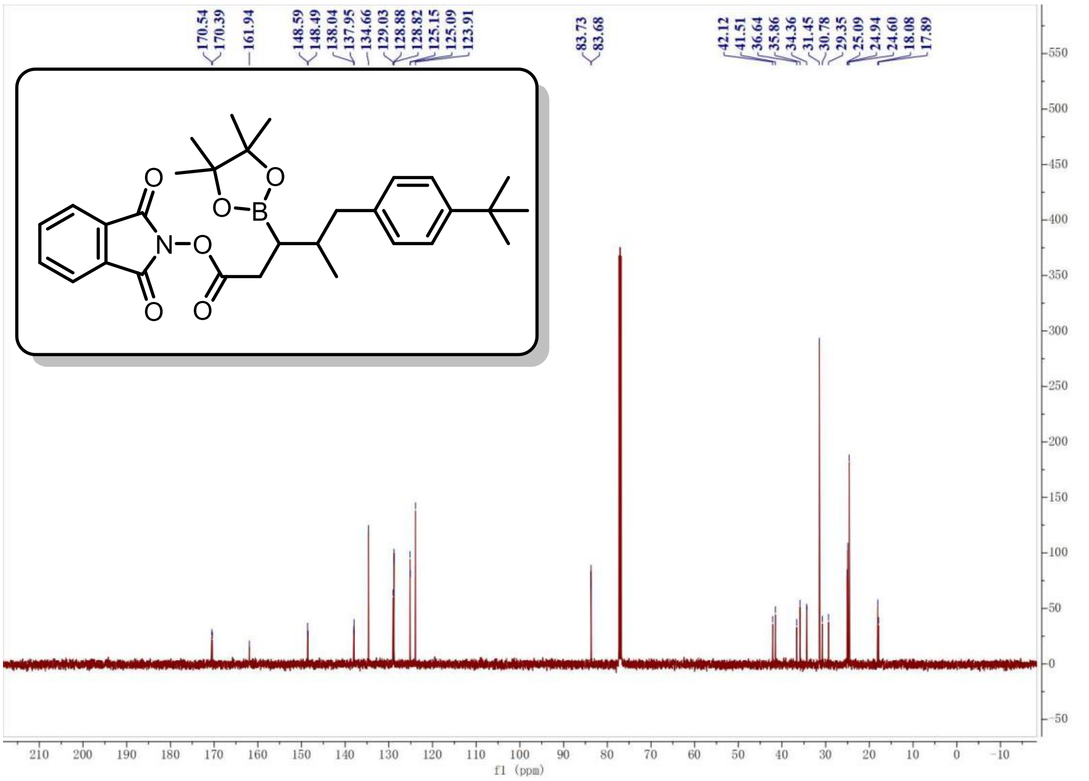

^{11}B NMR (160 MHz, Chloroform-*d*)

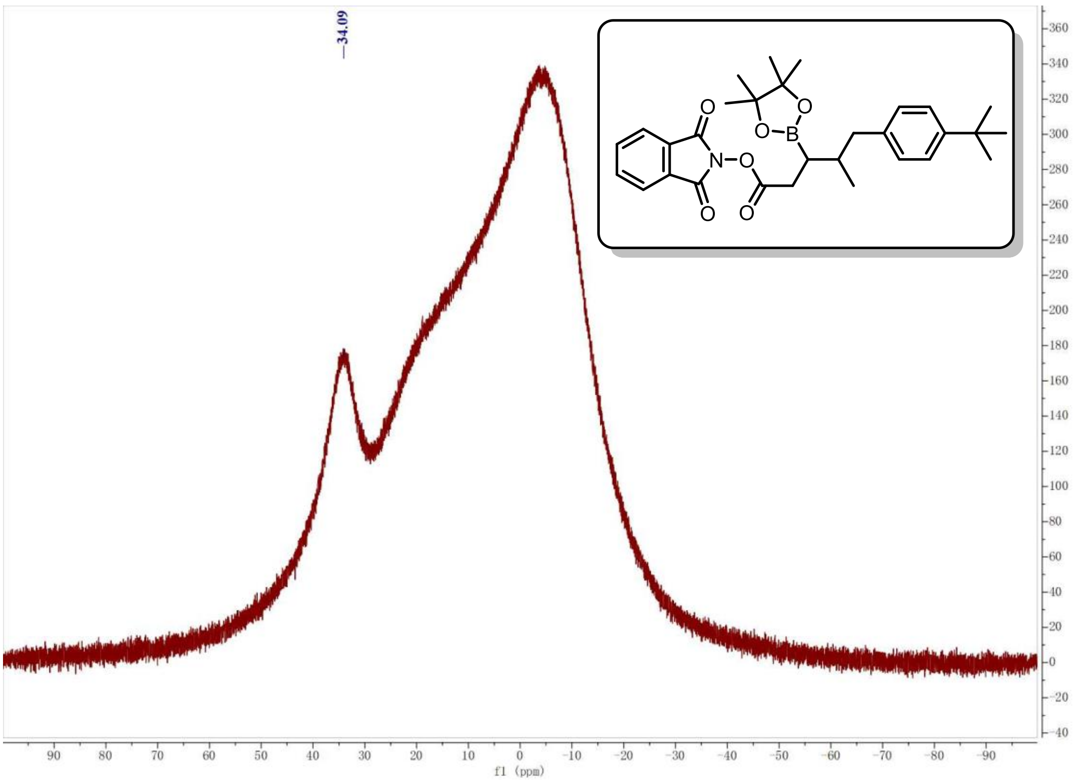

1,3-dioxoisoindolin-2-yl 3-(2,4-dimethylcyclohex-3-en-1-yl)-3-(4,4,5,5-tetramethyl-1,3,2-dioxaborolan-2-yl)propanoate (1l)

^{1}H NMR (500 MHz, Chloroform-*d*)

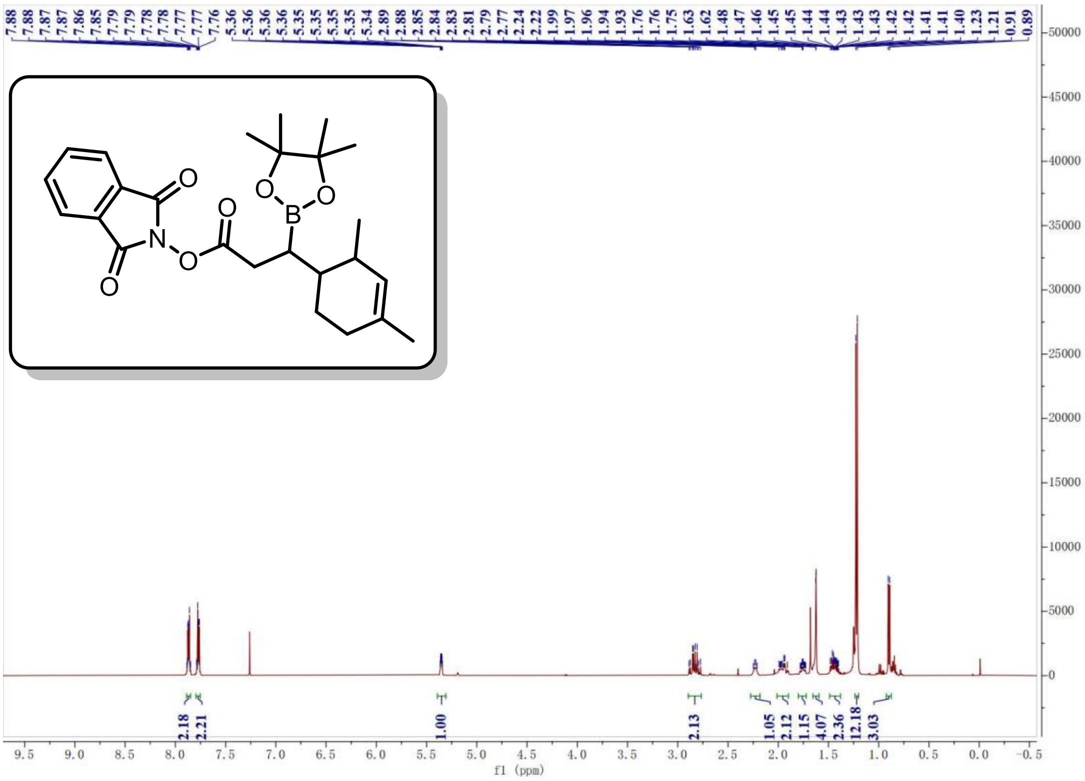

^{13}C NMR (126 MHz, Chloroform-*d*)

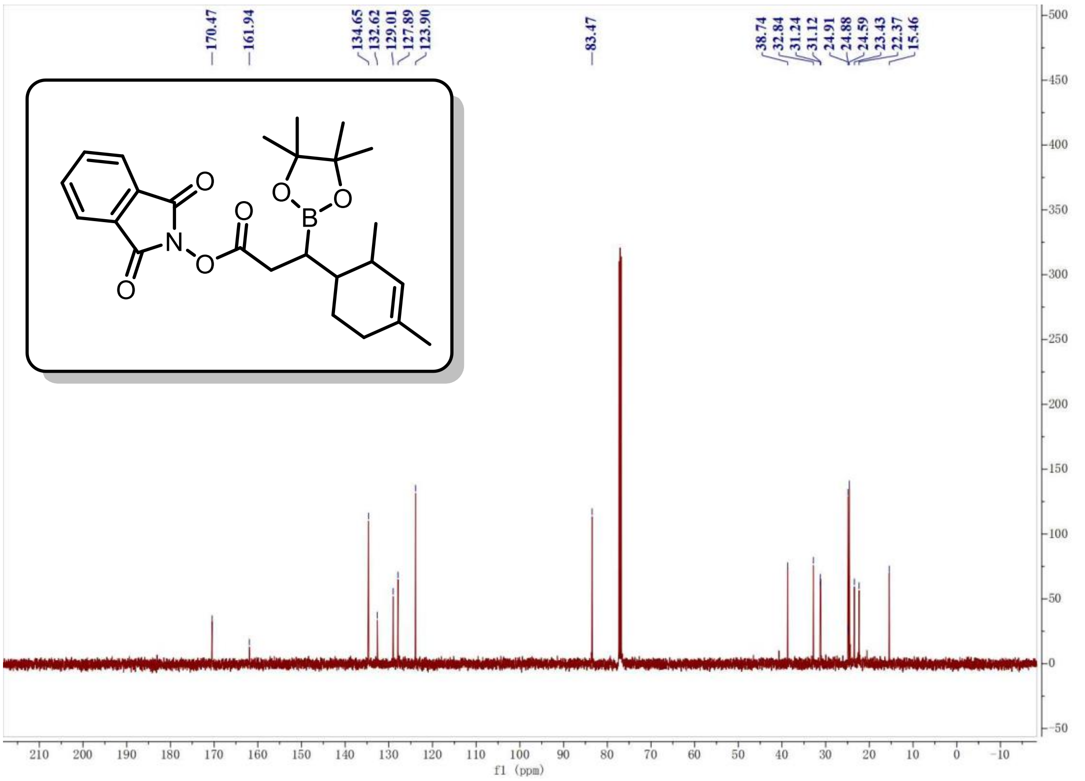

^{11}B NMR (160 MHz, Chloroform-*d*)

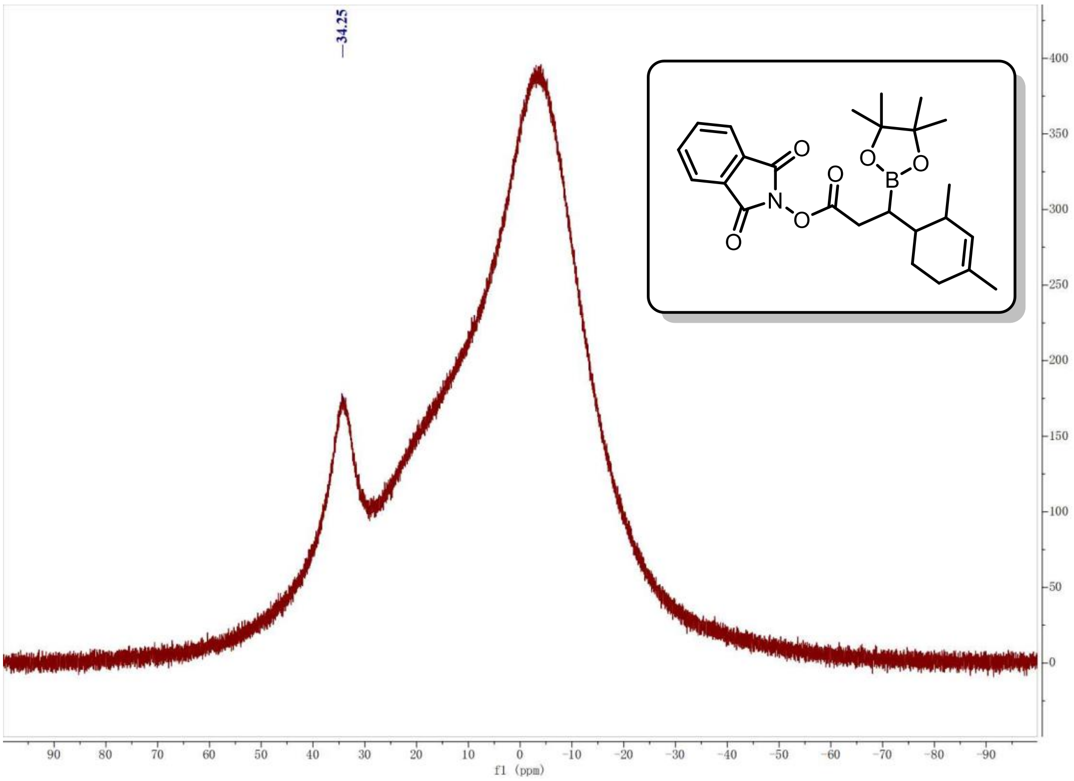

1,3-dioxoisoindolin-2-yl 4-ethyl-3-(4,4,5,5-tetramethyl-1,3,2-dioxaborolan-2-yl)hexanoate (1m)

^{1}H NMR (500 MHz, Chloroform-*d*)

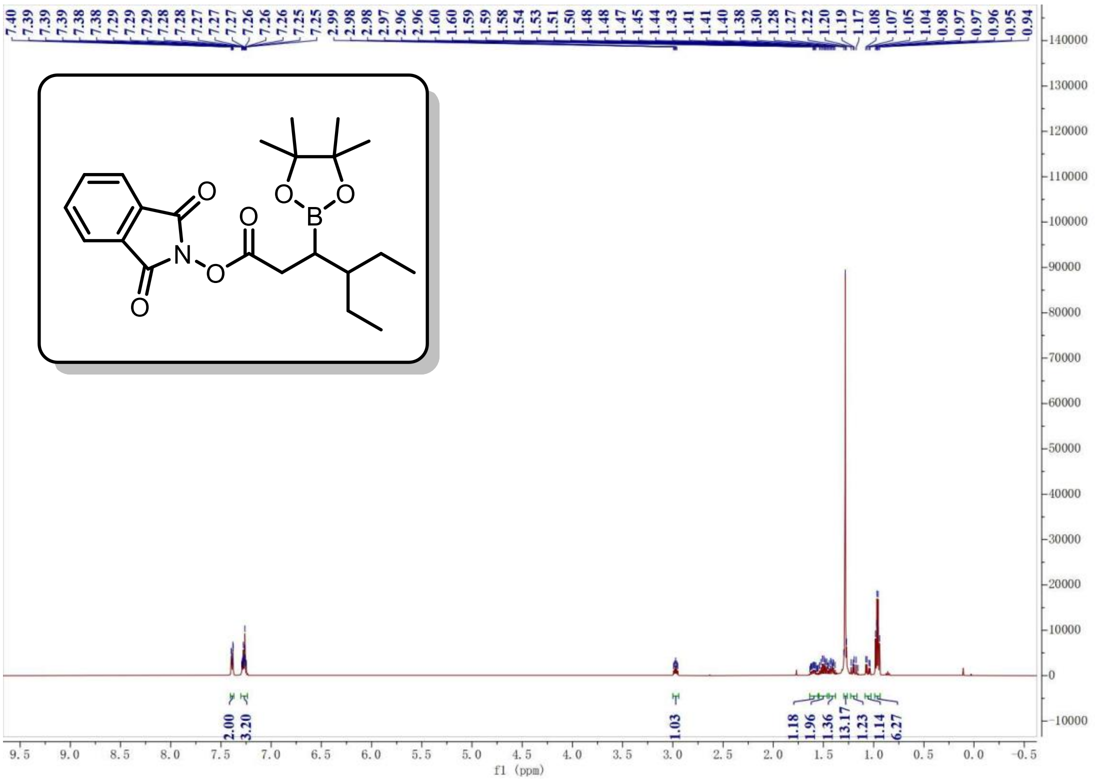

^{13}C NMR (126 MHz, Chloroform-*d*)

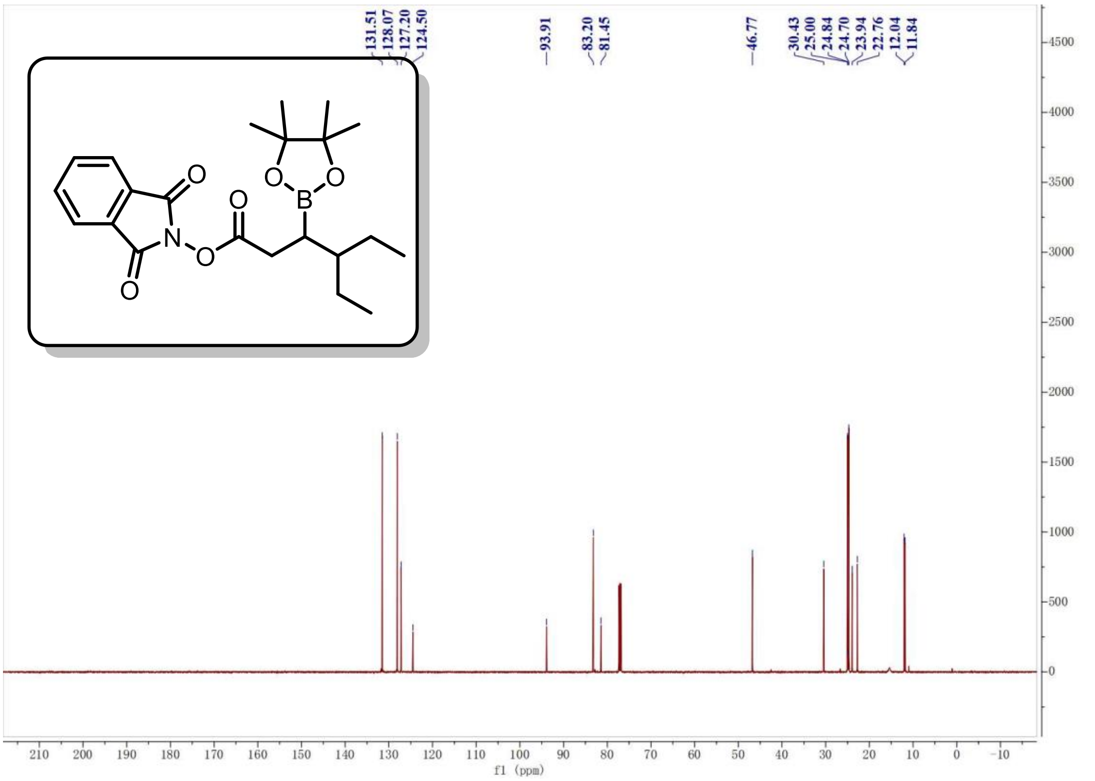

^{11}B NMR (160 MHz, Chloroform-*d*)

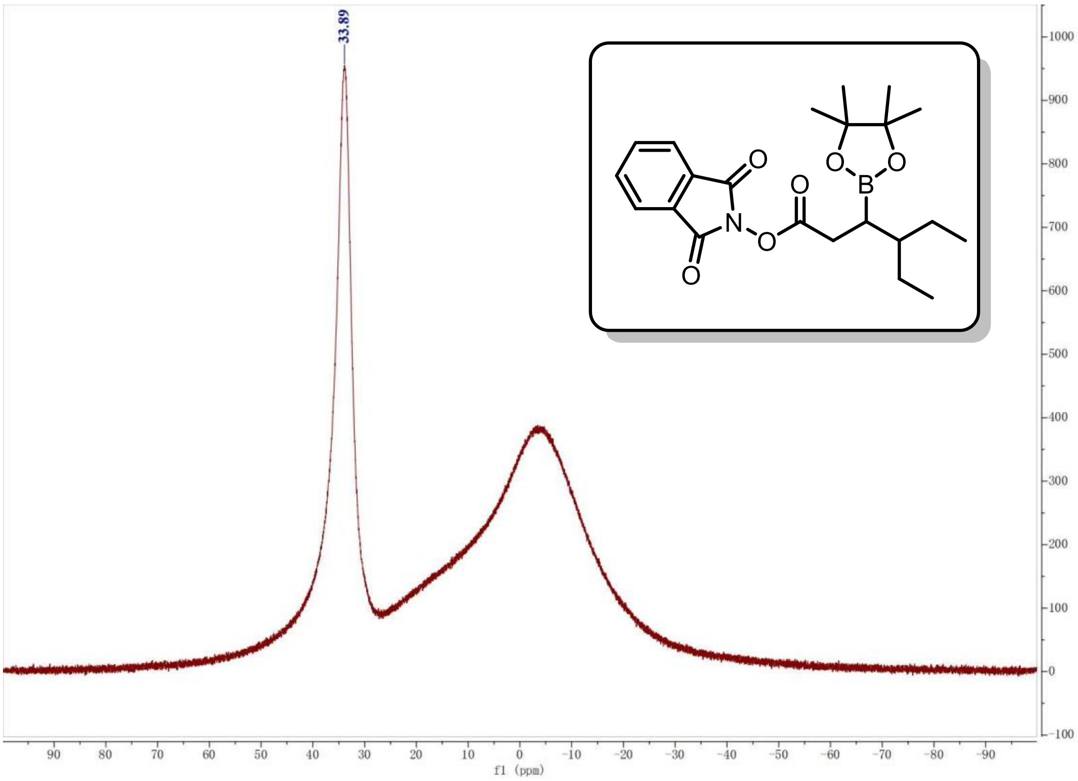

1,3-dioxoisoindolin-2-yl 3-(cyclohex-2-en-1-yl)-3-(4,4,5,5-tetramethyl-1,3,2-dioxaborolan-2-yl)propanoate (1n)

^{1}H NMR (500 MHz, Chloroform-*d*)

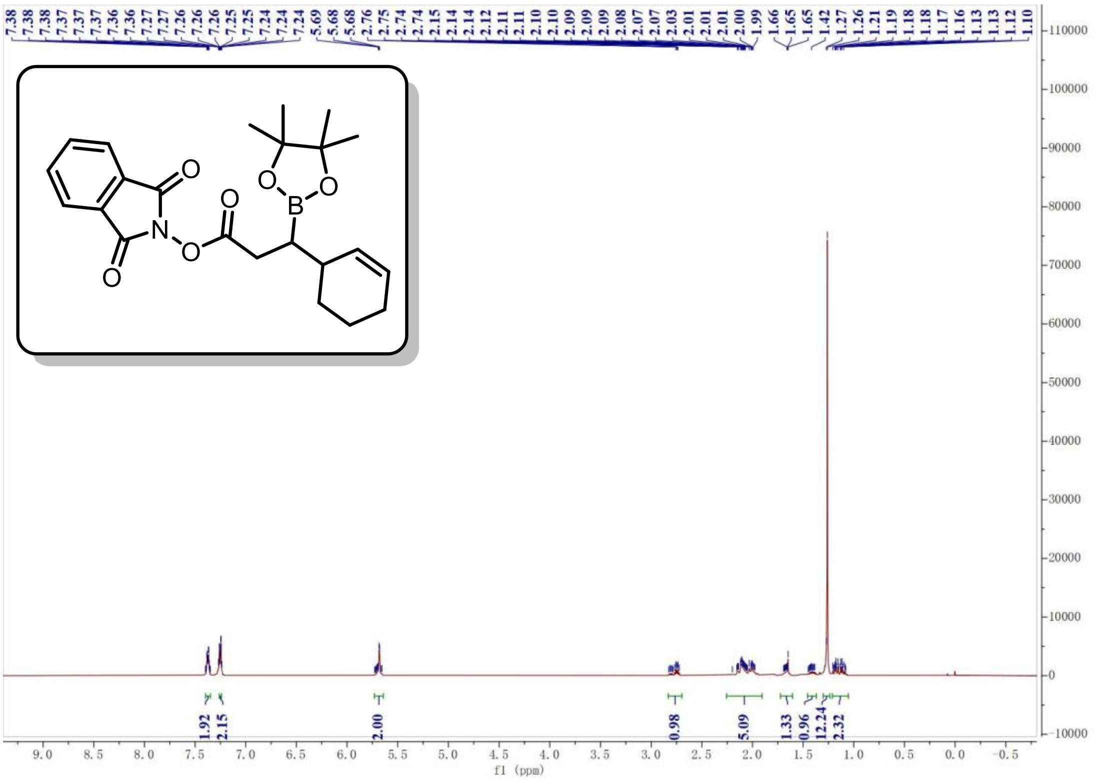

^{13}C NMR (126 MHz, Chloroform-*d*)

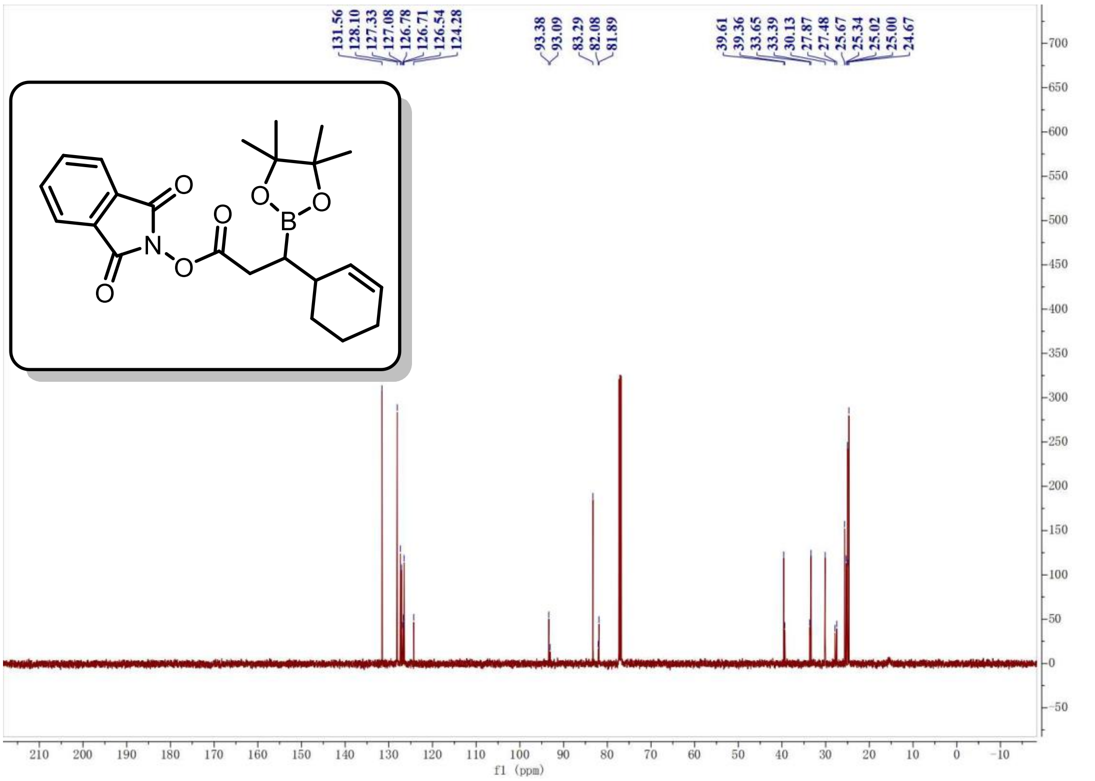

^{11}B NMR (160 MHz, Chloroform-*d*)

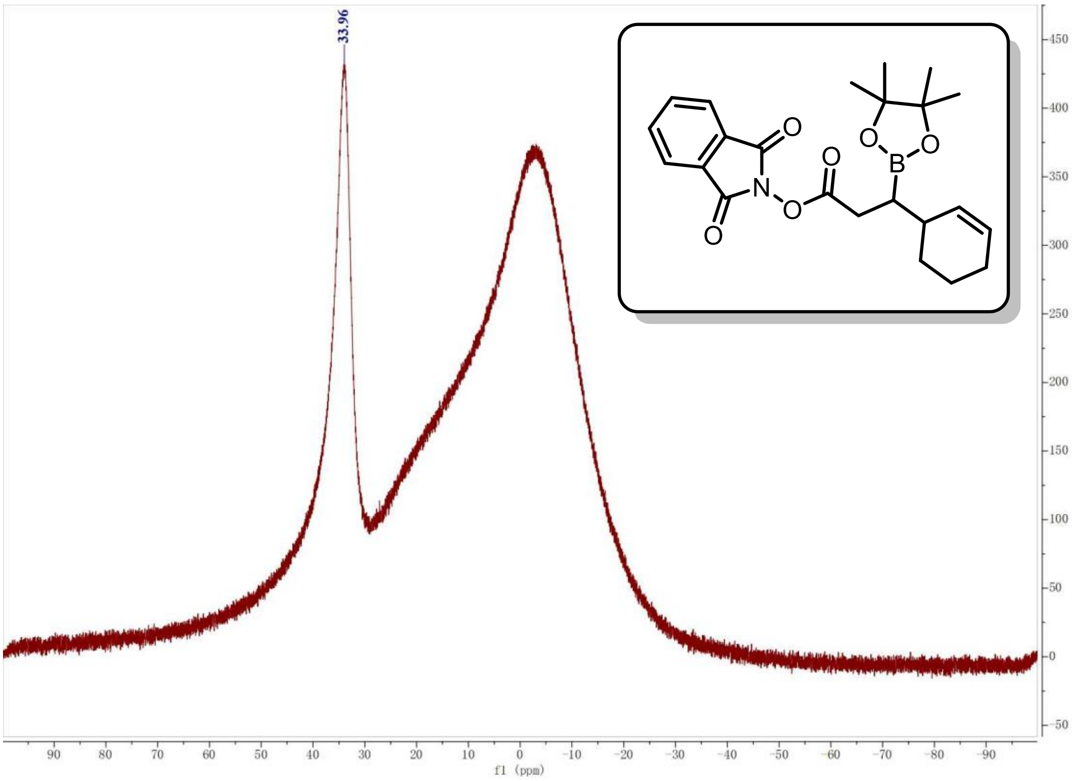

1,3-dioxoisoindolin-2-yl 3-(4,4,5,5-tetramethyl-1,3,2-dioxaborolan-2-yl)nonanoate (1o)

^{1}H NMR (500 MHz, Chloroform-*d*)

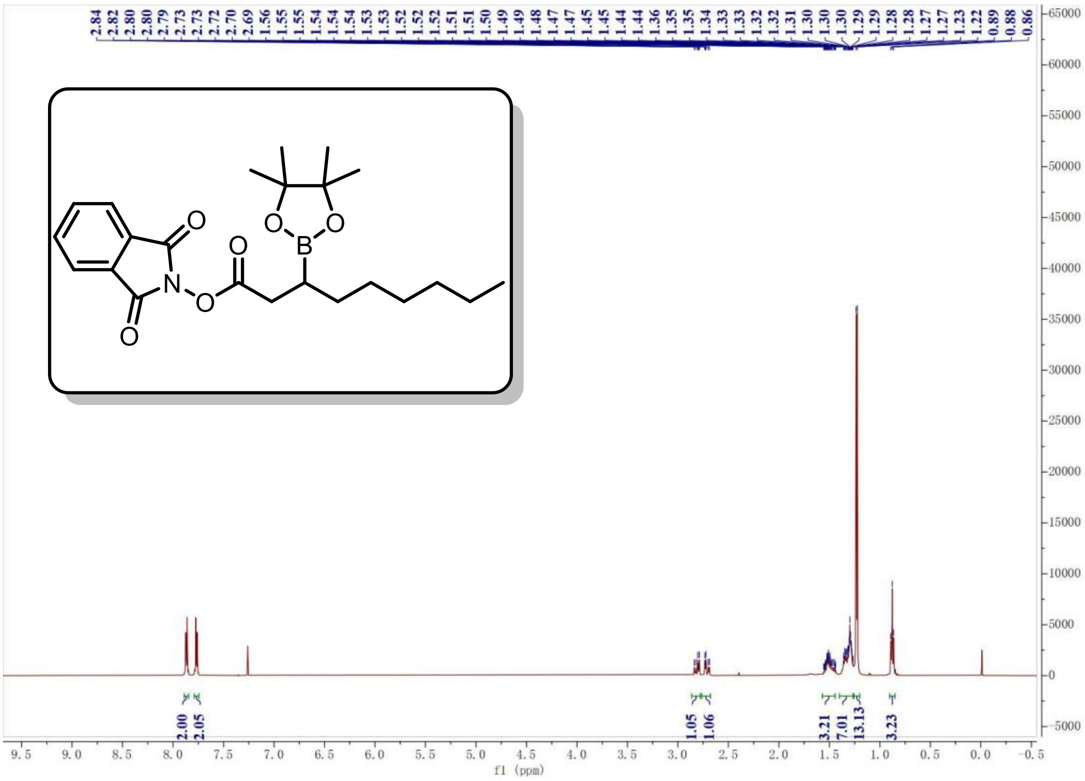

^{13}C NMR (126 MHz, Chloroform-*d*)

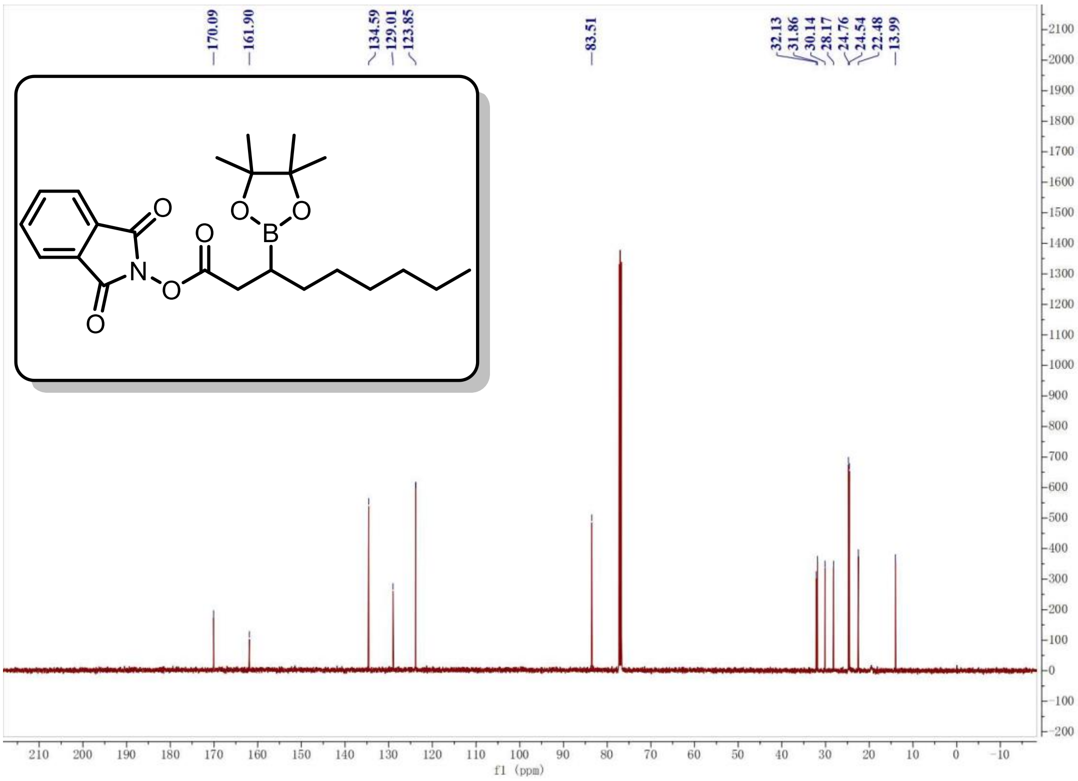

^{11}B NMR (160 MHz, Chloroform-*d*)

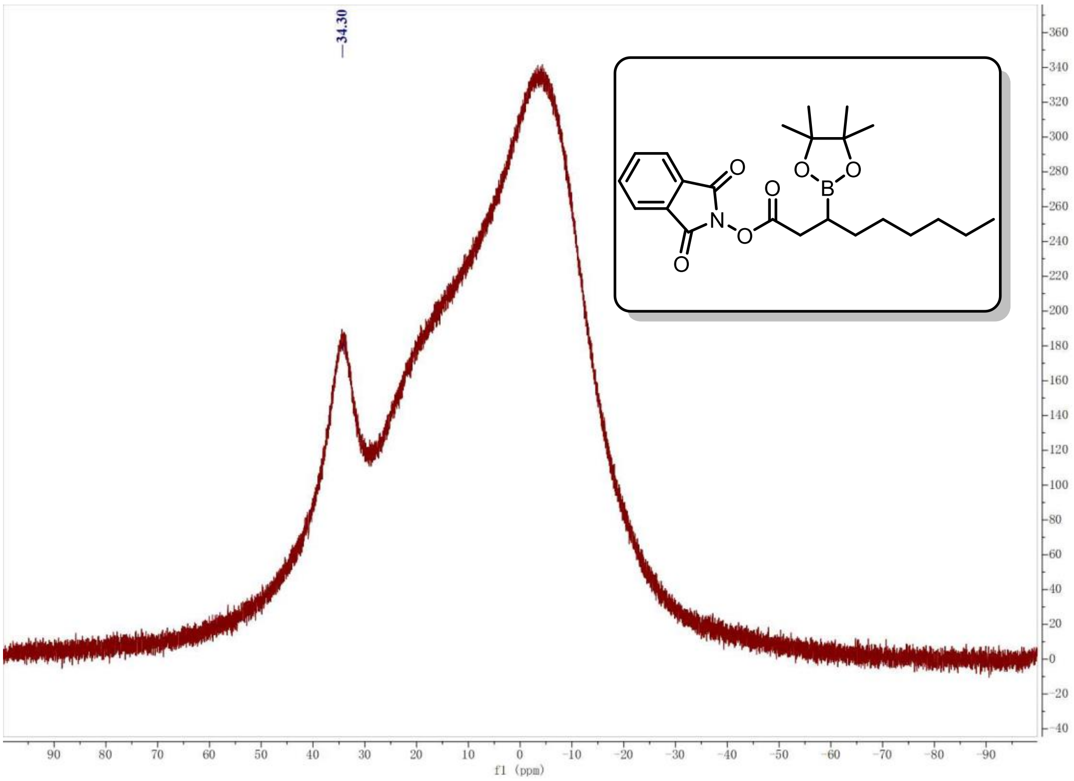

1,3-dioxoisoindolin-2-yl 3-(4,4,5,5-tetramethyl-1,3,2-dioxaborolan-2-yl)hexanoate (1p)

^{1}H NMR (500 MHz, Chloroform-*d*)

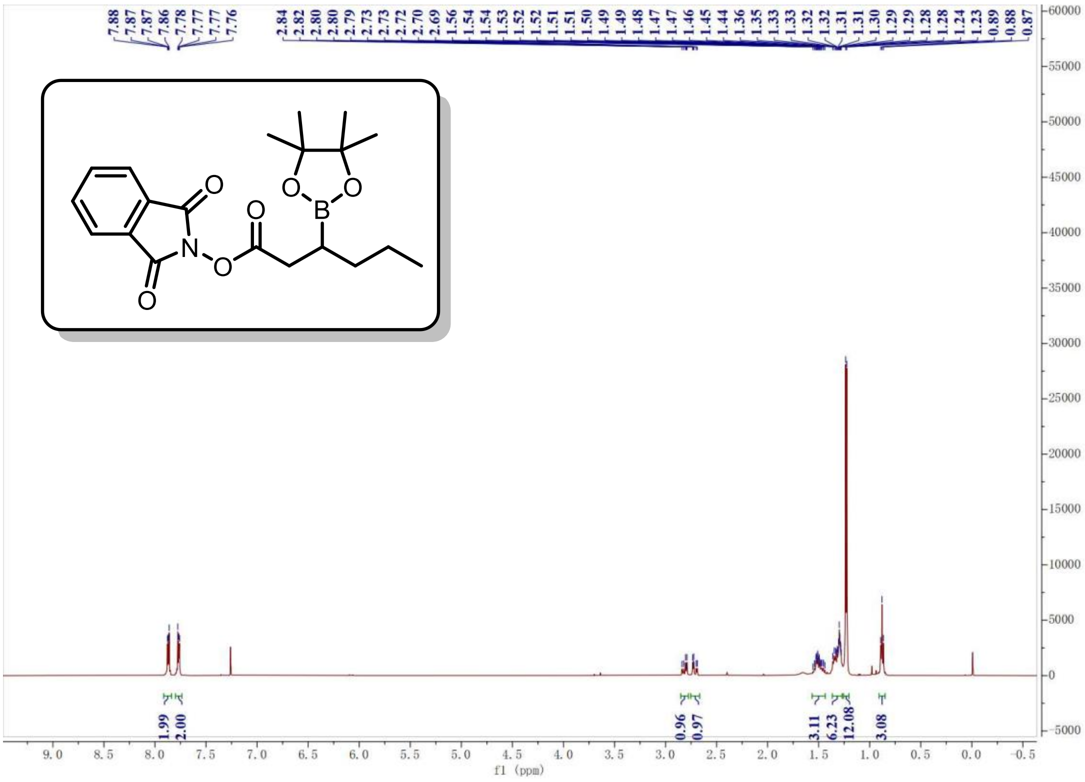

^{13}C NMR (126 MHz, Chloroform-*d*)

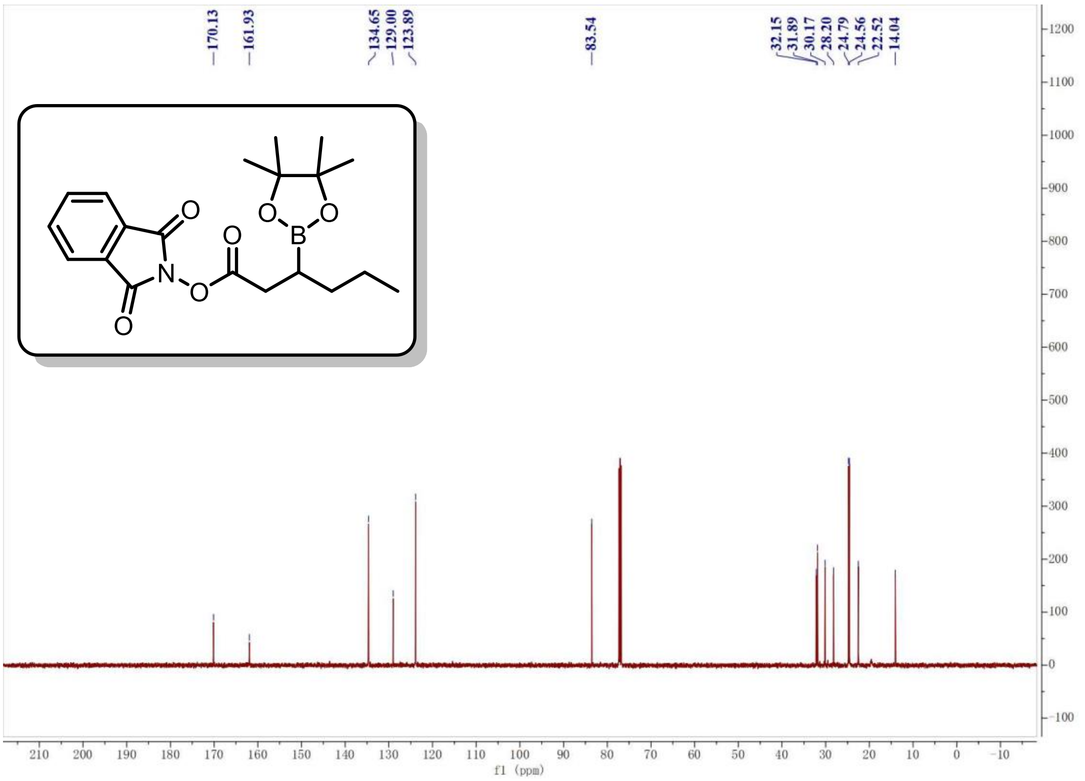

^{11}B NMR (160 MHz, Chloroform-*d*)

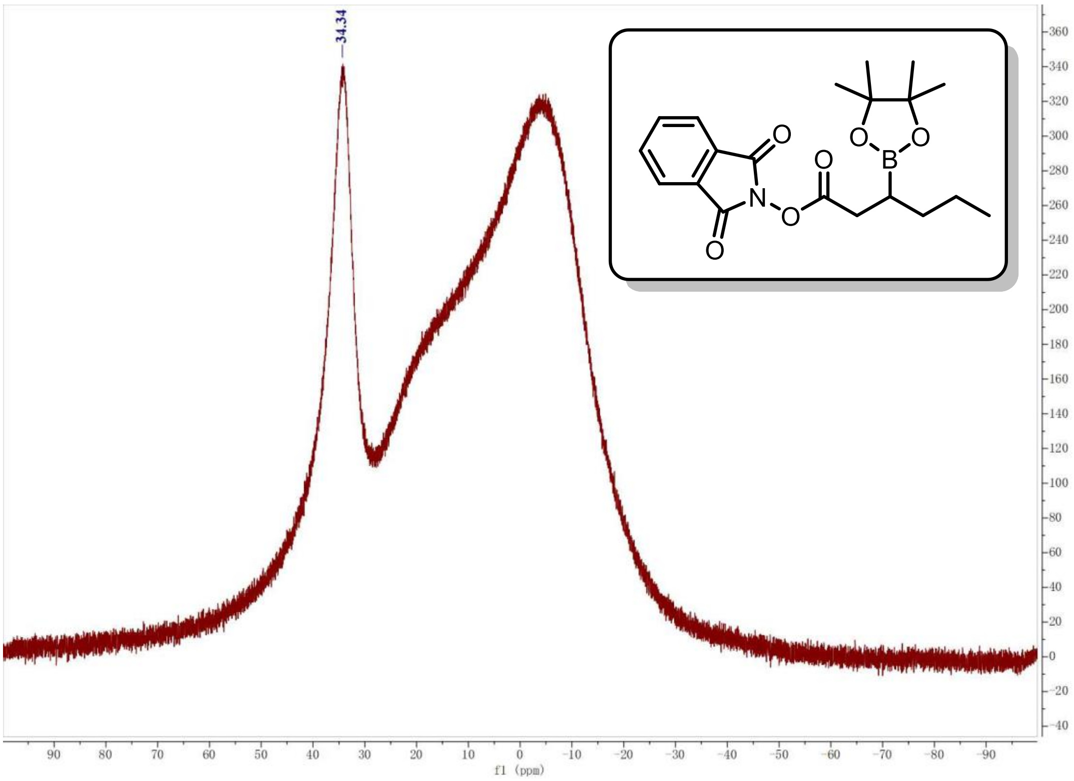

1,3-dioxoisoindolin-2-yl 2-(1-(4,4,5,5-tetramethyl-1,3,2-dioxaborolan-2-yl)cyclobutyl)acetate (1q)

^{1}H NMR (500 MHz, Chloroform-*d*)

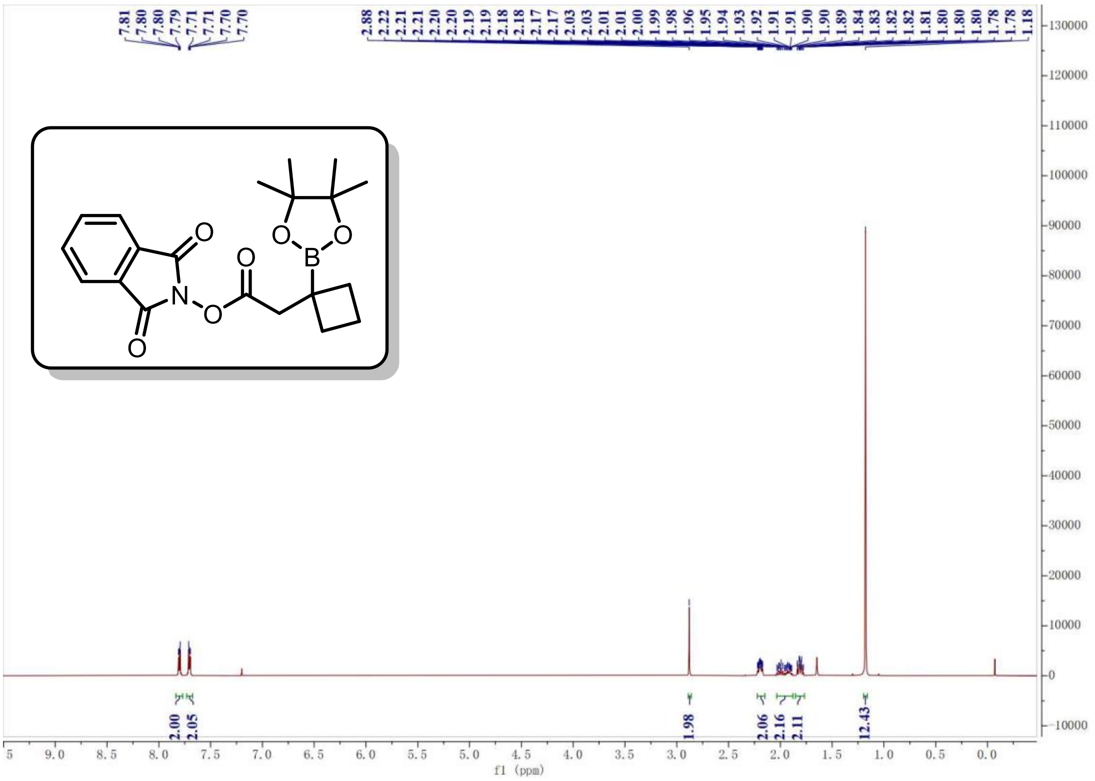

^{13}C NMR (126 MHz, Chloroform-*d*)

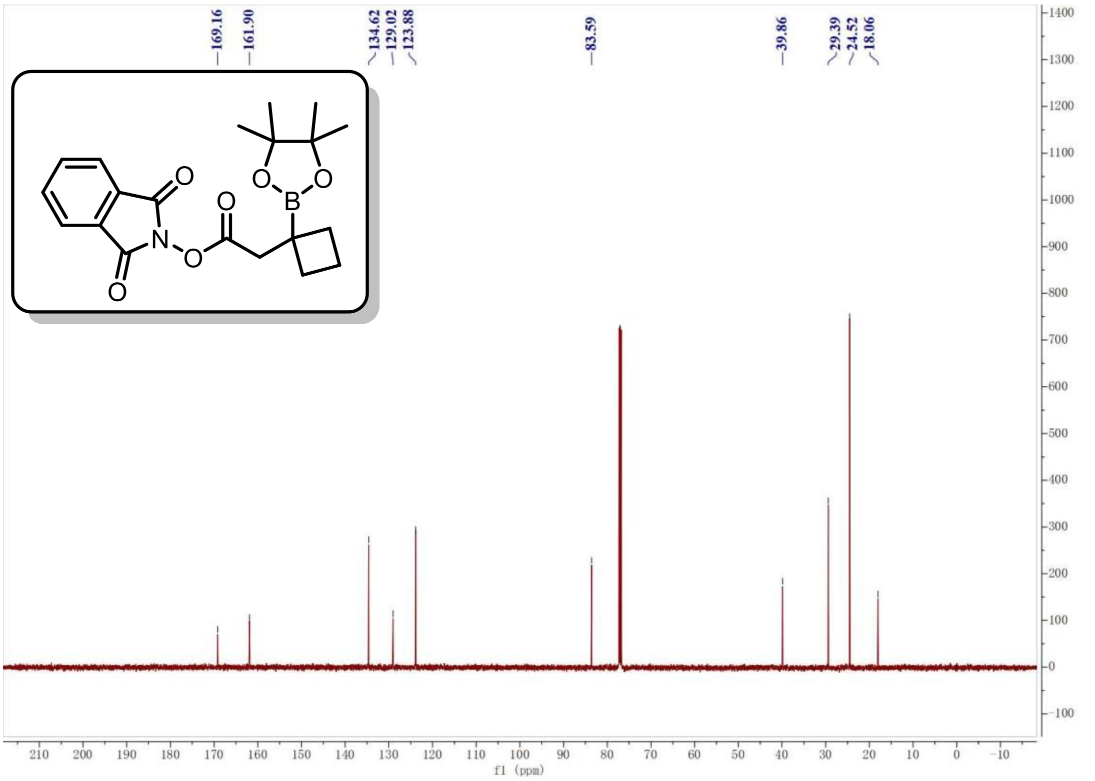

^{11}B NMR (160 MHz, Chloroform-*d*)

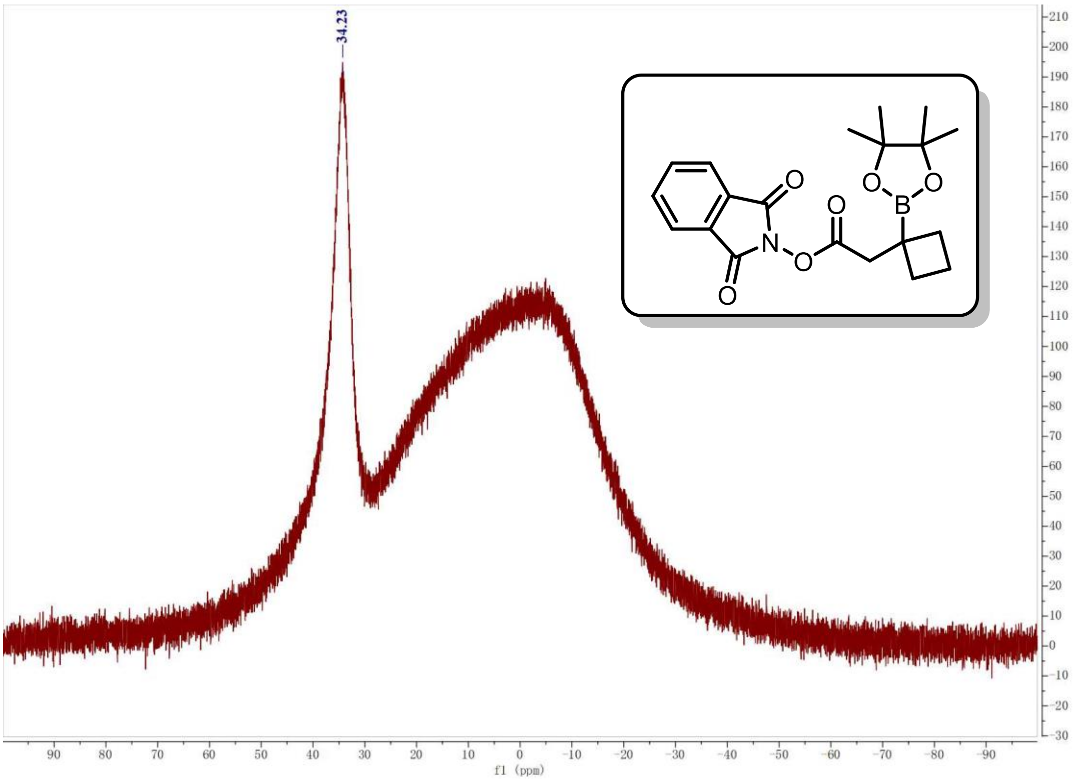

1,3-dioxoisoindolin-2-yl 3-(5,5-dimethyl-1,3,2-dioxaborinan-2-yl)-5,5-dimethylhexanoate (1r)

^{1}H NMR (500 MHz, Chloroform-*d*)

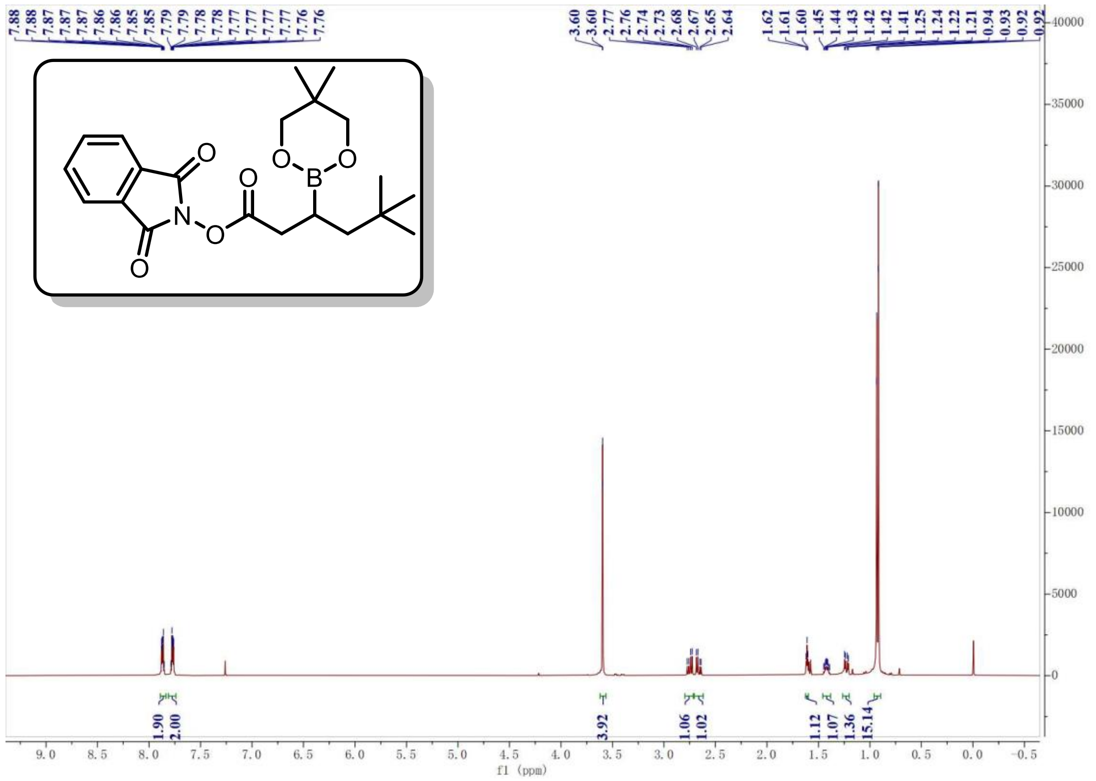

^{13}C NMR (126 MHz, Chloroform-*d*)

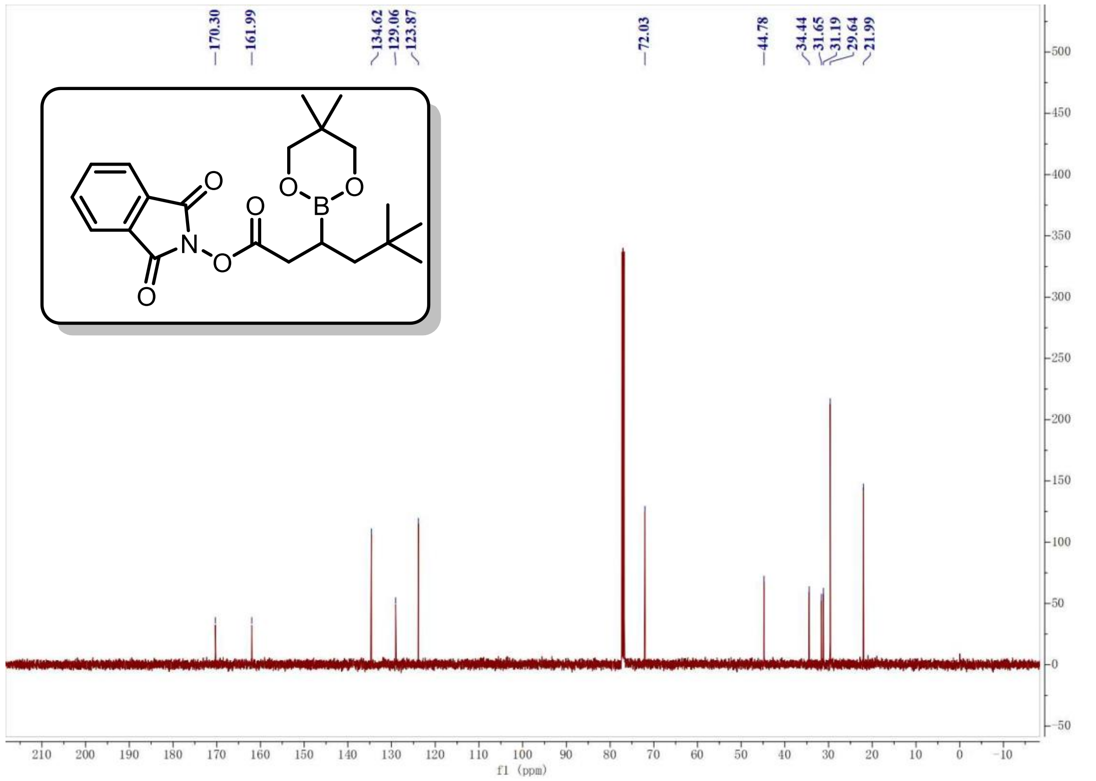

^{11}B NMR (160 MHz, Chloroform-*d*)

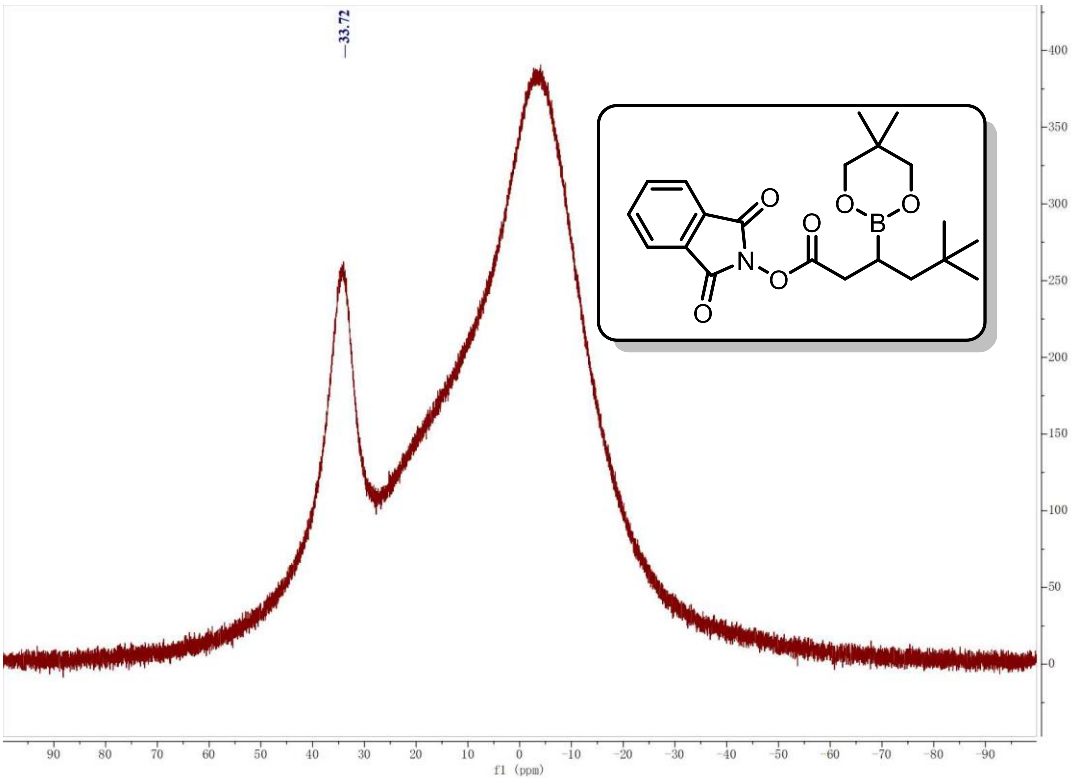

1,3-dioxoisoindolin-2-yl 3,3-bis(4,4,5,5-tetramethyl-1,3,2-dioxaborolan-2-yl)butanoate (1s)

^{1}H NMR (500 MHz, Chloroform-*d*)

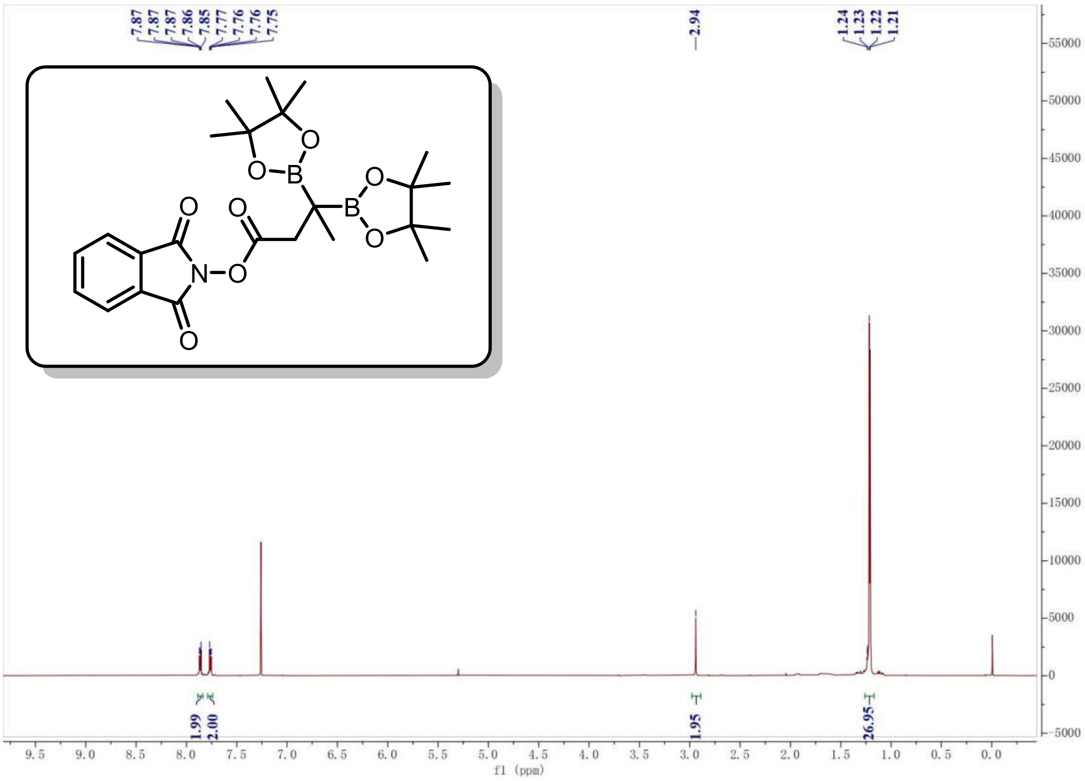

^{13}C NMR (126 MHz, Chloroform-*d*)

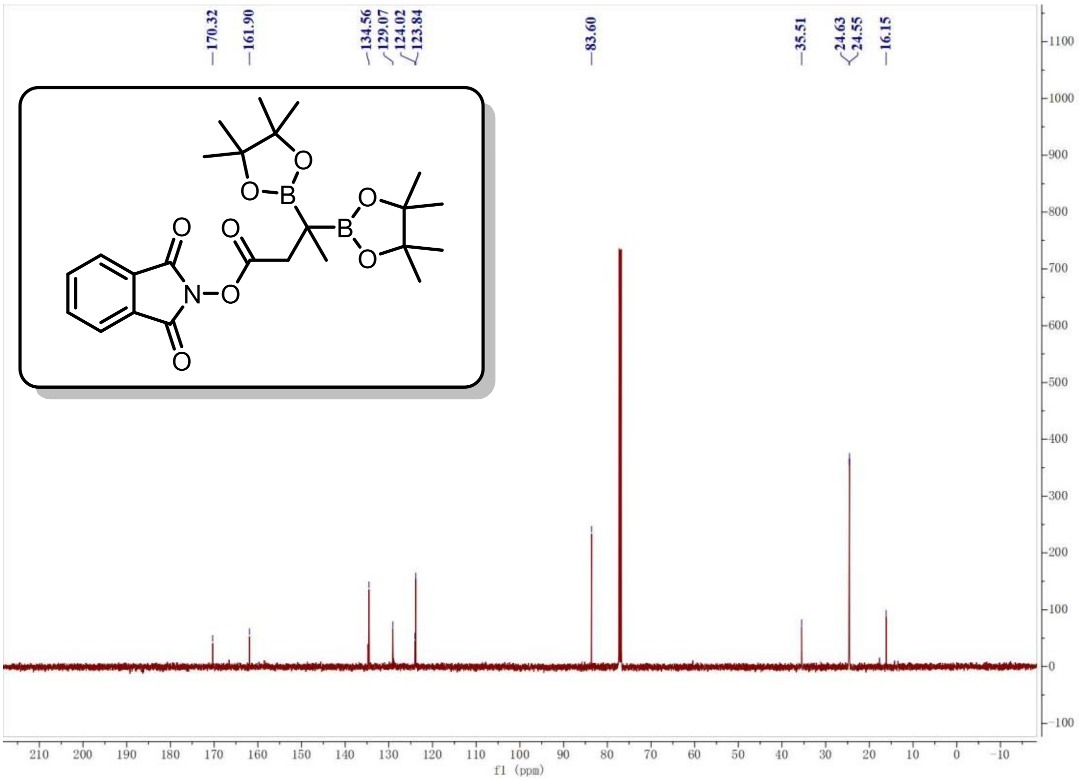

^{11}B NMR (160 MHz, Chloroform-*d*)

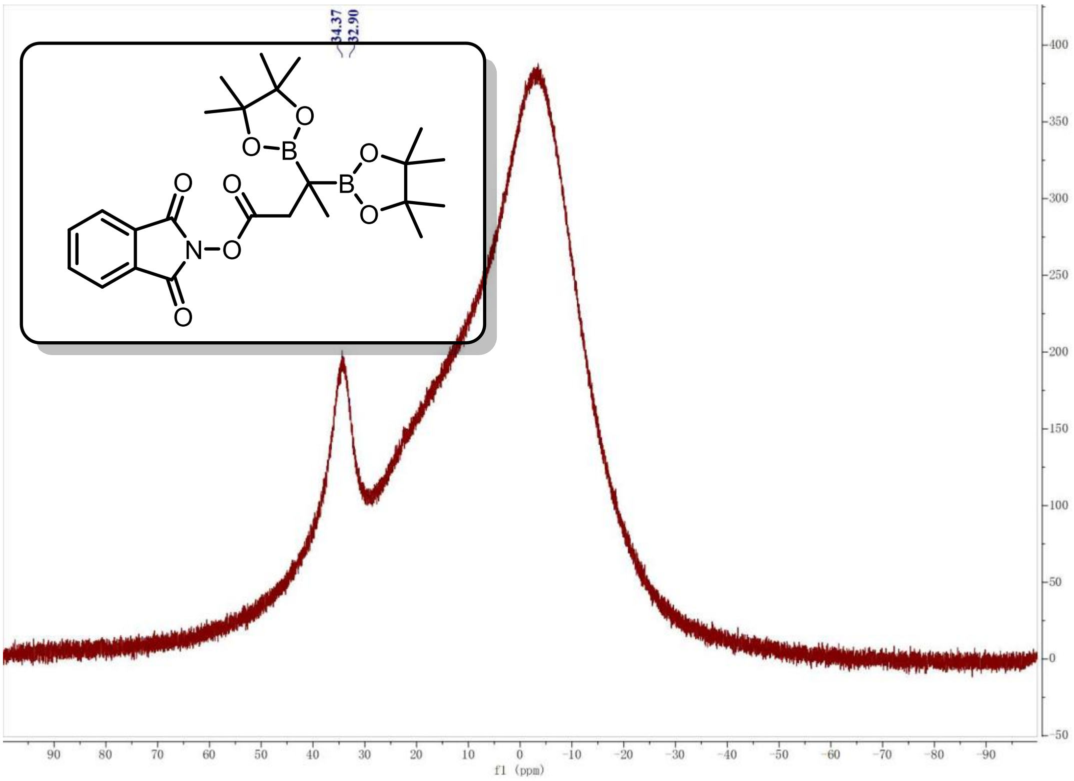

1,3-dioxoisoindolin-2-yl 3,3-bis(4,4,5,5-tetramethyl-1,3,2-dioxaborolan-2-yl)pentanoate (1t)

^{1}H NMR (500 MHz, Chloroform-*d*)

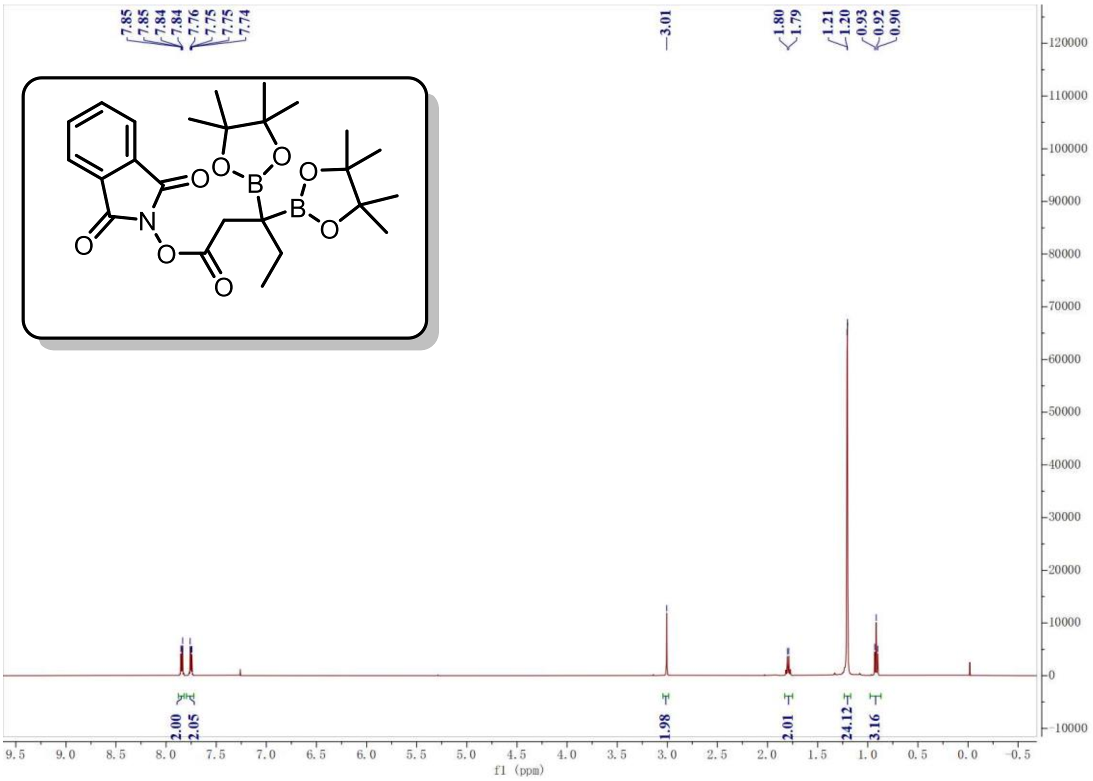

^{13}C NMR (126 MHz, Chloroform-*d*)

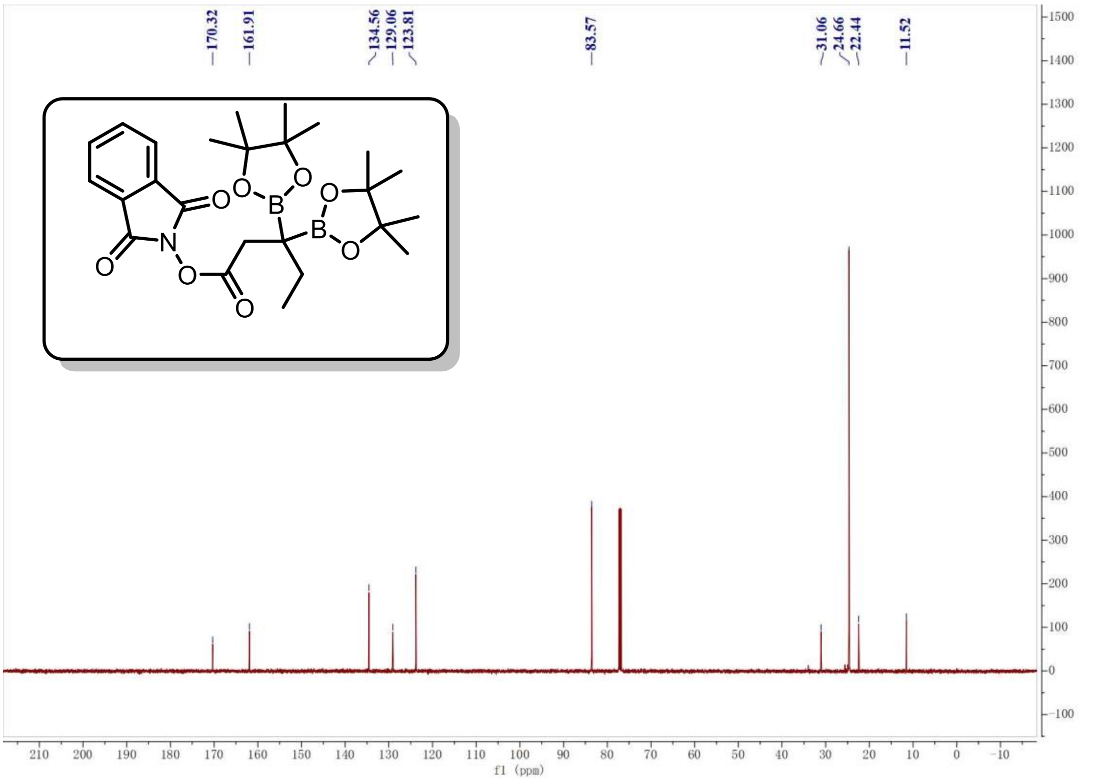

^{11}B NMR (160 MHz, Chloroform-*d*)

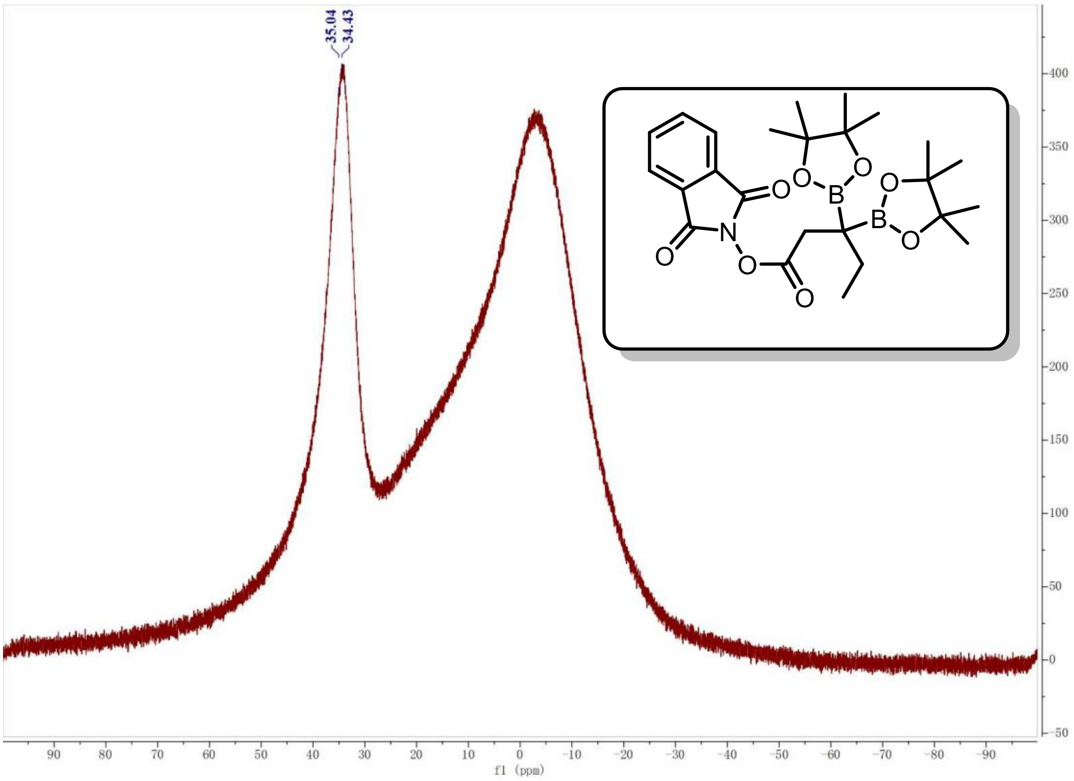

1,3-dioxoisoindolin-2-yl 3,3-bis(4,4,5,5-tetramethyl-1,3,2-dioxaborolan-2-yl)heptanoate (1u)

^{1}H NMR (500 MHz, Chloroform-*d*)

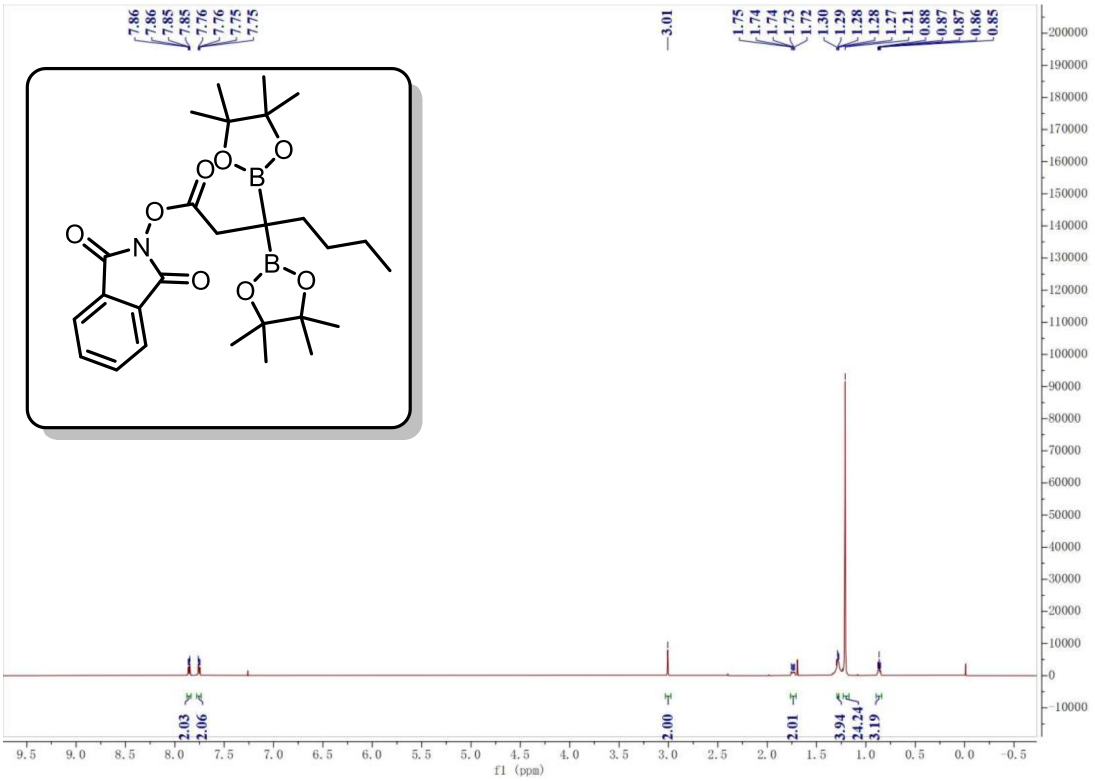

^{13}C NMR (126 MHz, Chloroform-*d*)

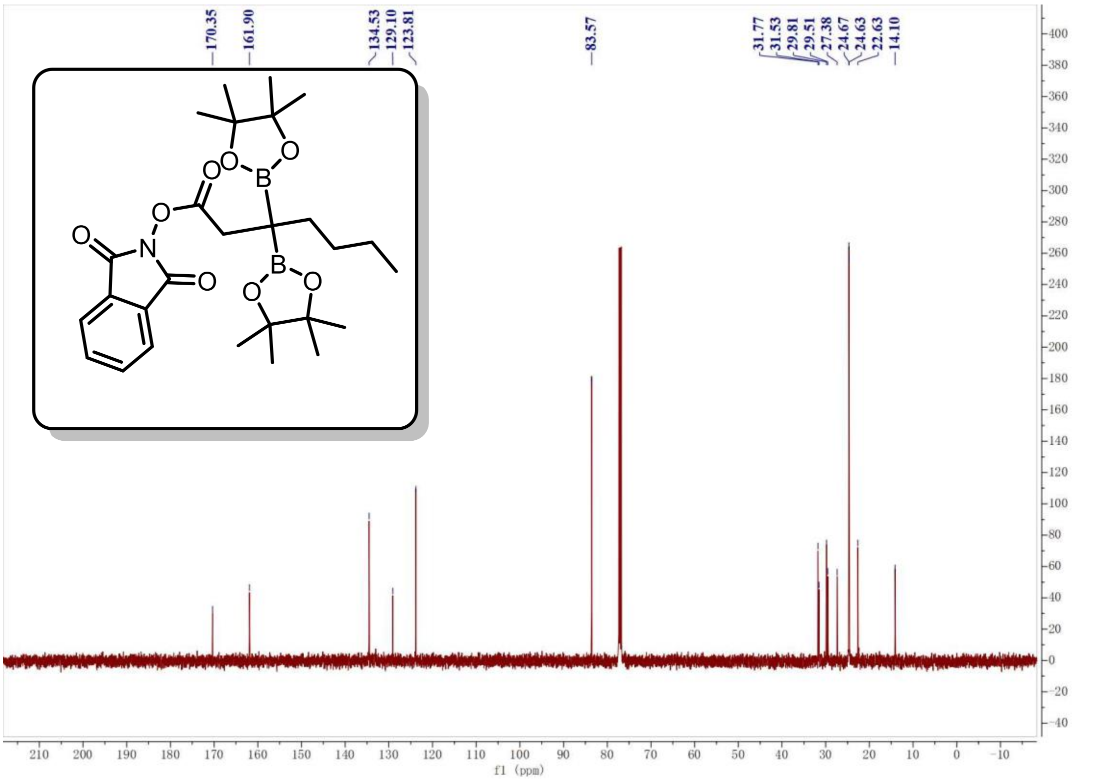

^{11}B NMR (160 MHz, Chloroform-*d*)

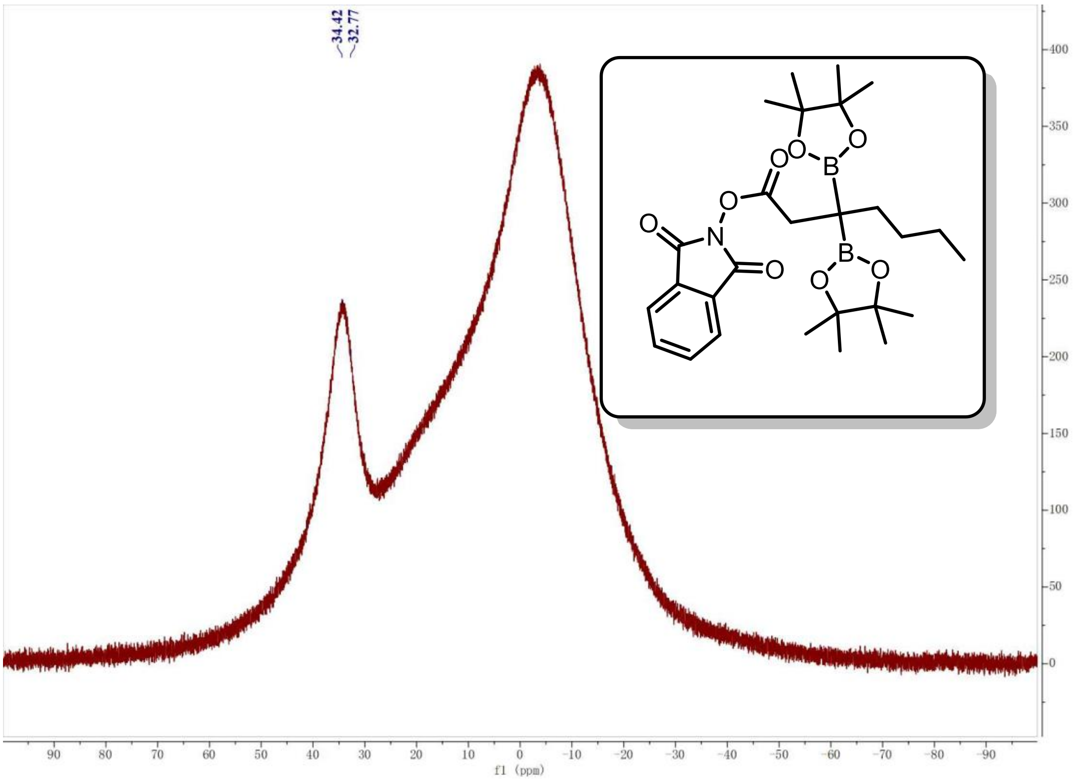

2-(4,4-dimethyl-2-(phenylethynyl)pentyl)-4,4,5,5-tetramethyl-1,3,2-dioxaborolane (3a)

^{1}H NMR (500 MHz, Chloroform-*d*)

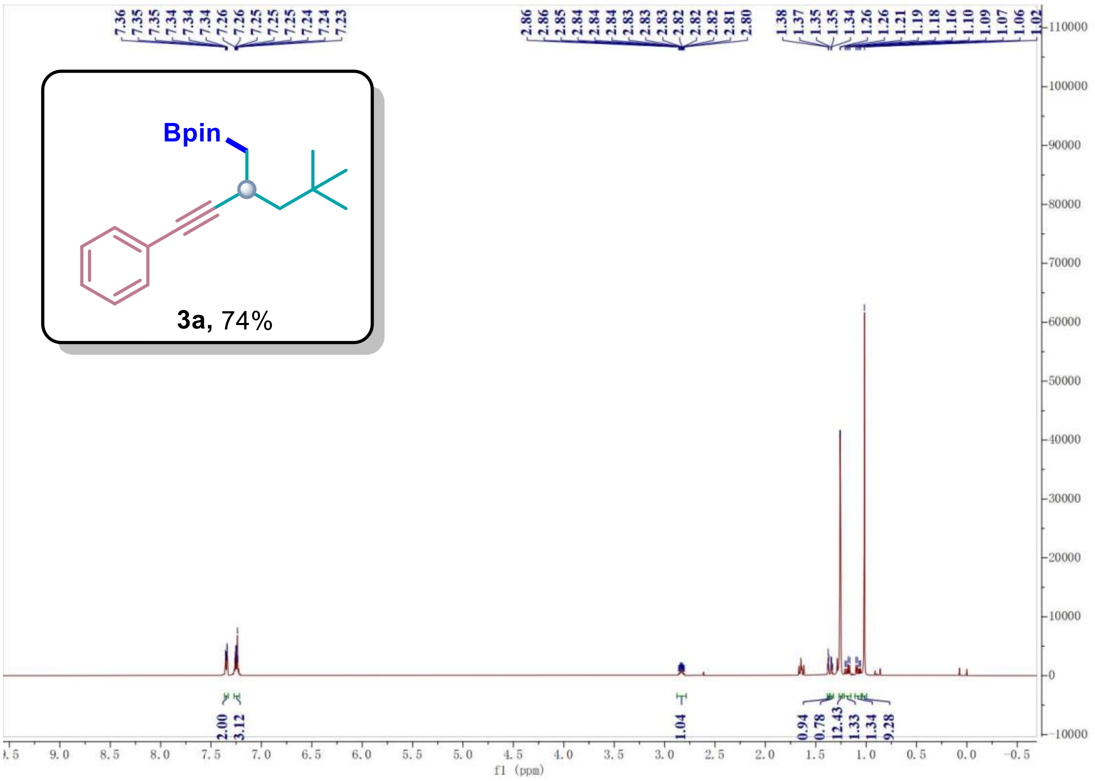

^{13}C NMR (126 MHz, Chloroform-*d*)

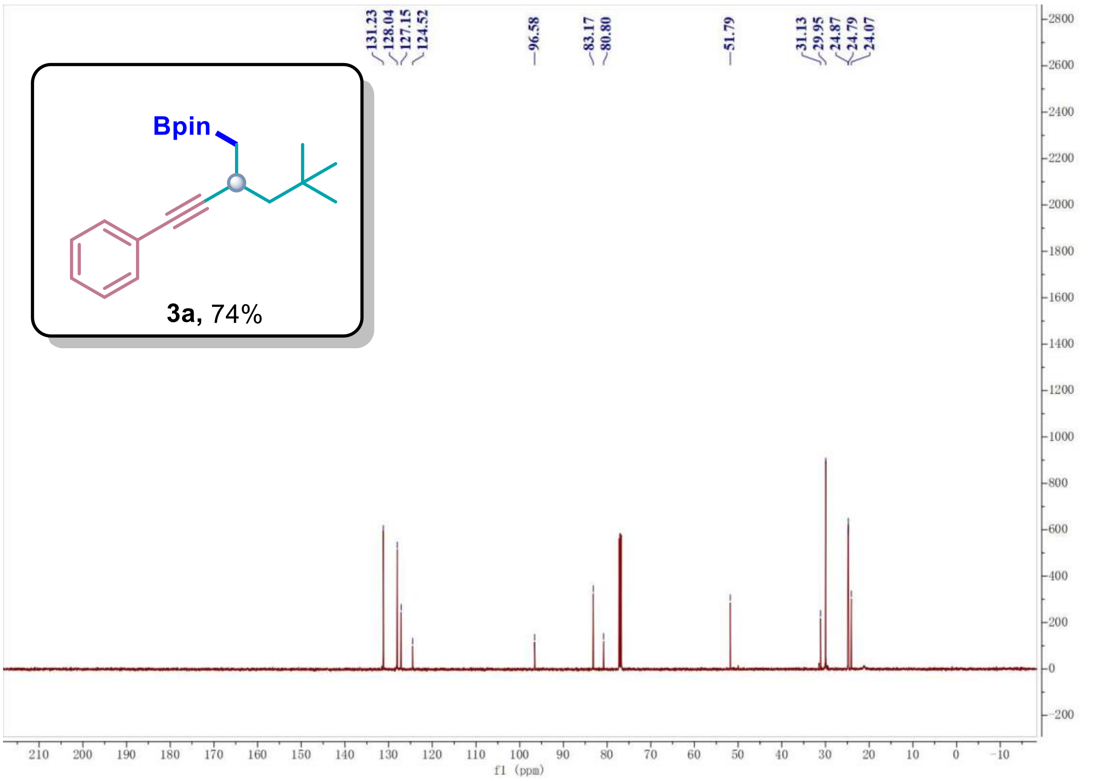

^{11}B NMR (160 MHz, Chloroform-*d*)

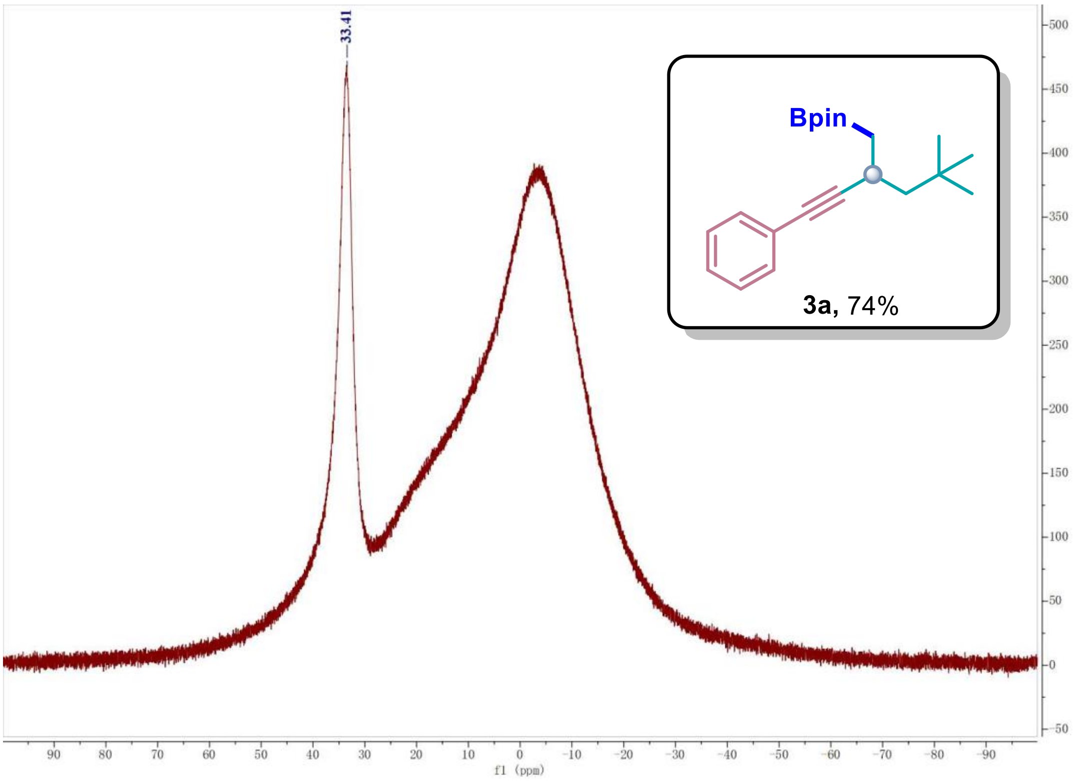

2-(4,4-dimethyl-2-(p-tolylethynyl)pentyl)-4,4,5,5-tetramethyl-1,3,2-dioxaborolane (3b)

^{1}H NMR (500 MHz, Chloroform-*d*)

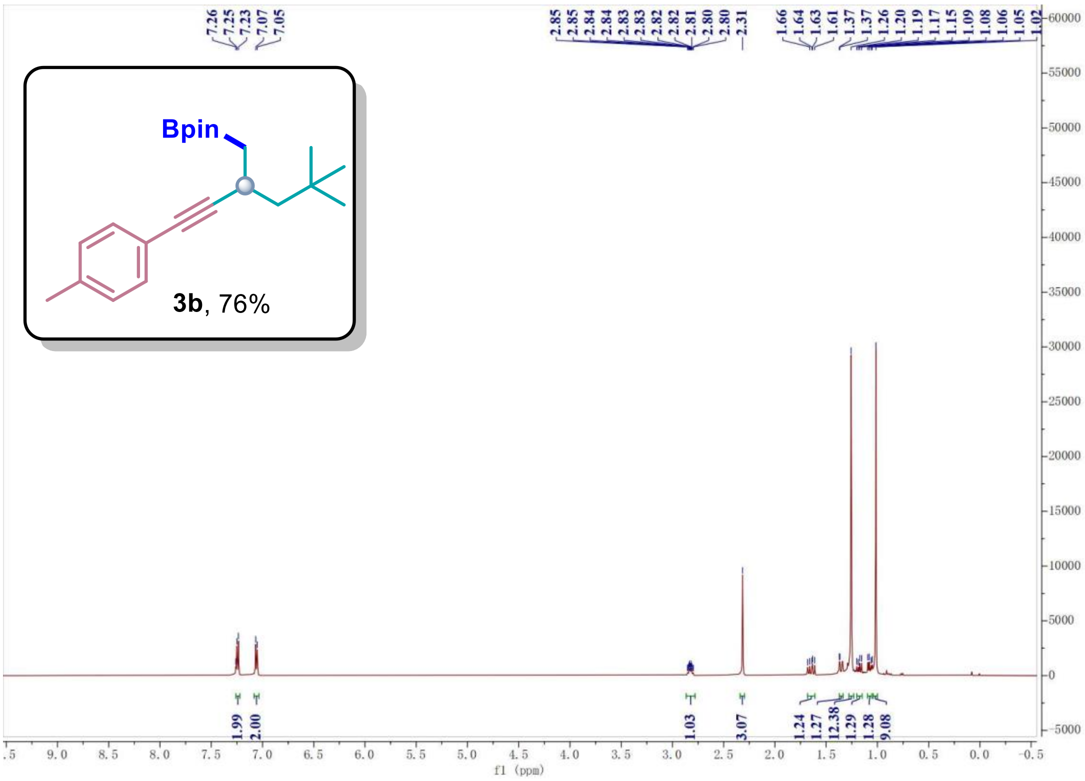

^{13}C NMR (126 MHz, Chloroform-*d*)

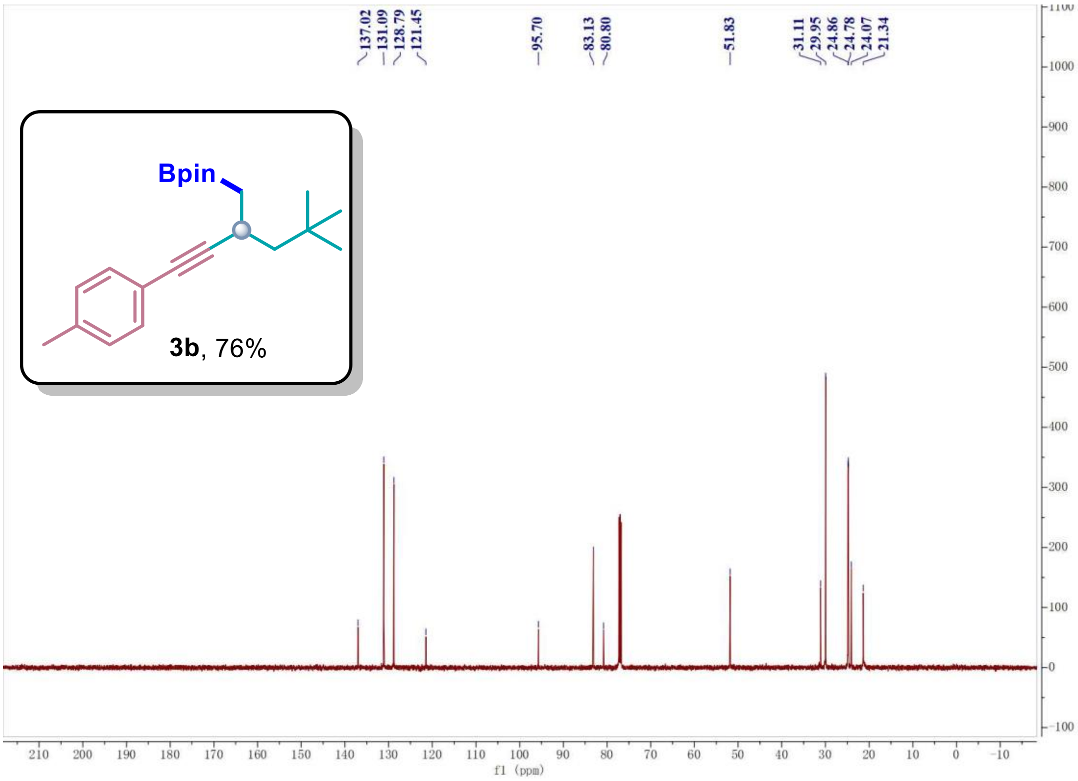

^{11}B NMR (160 MHz, Chloroform-*d*)

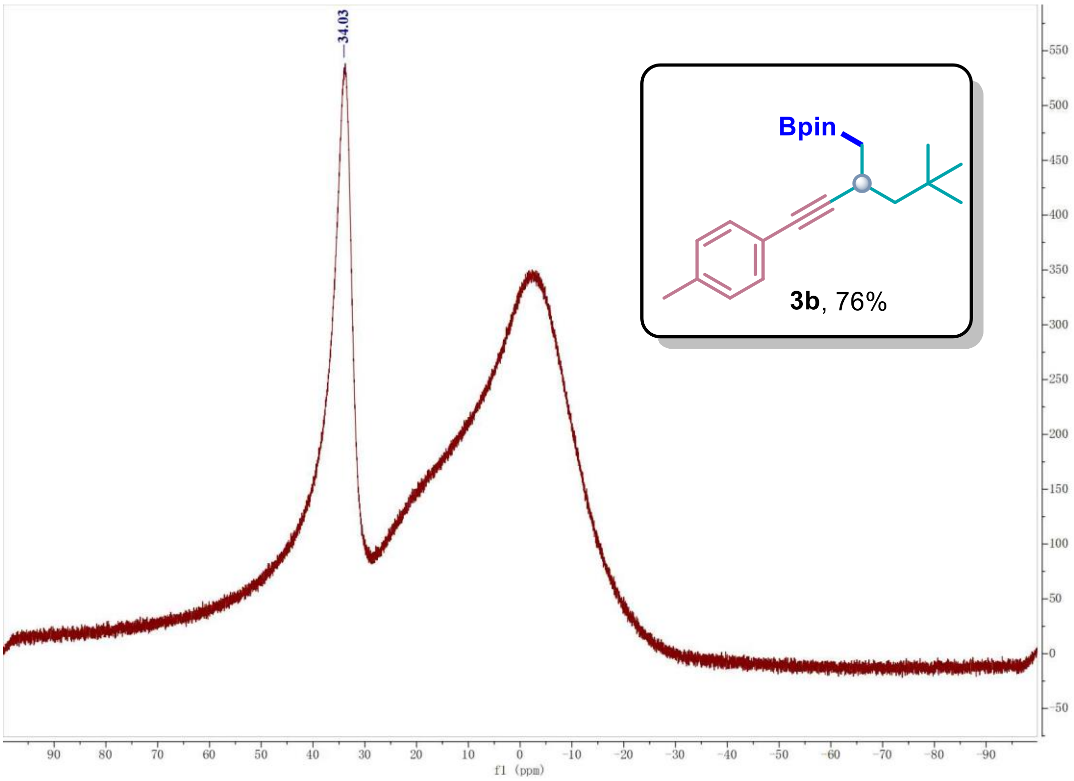

2-(2-((4-fluorophenyl)ethynyl)-4,4-dimethylpentyl)-4,4,5,5-tetramethyl-1,3,2-dioxaborolane (3c)

^{1}H NMR (500 MHz, Chloroform-*d*)

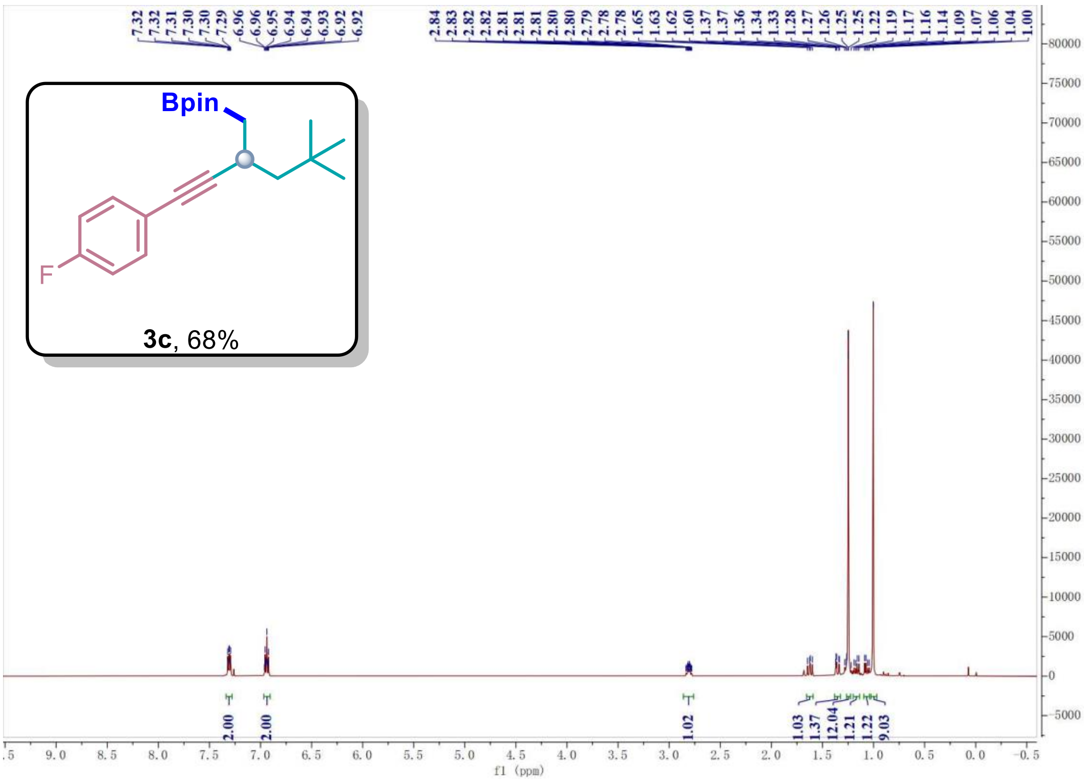

^{13}C NMR (126 MHz, Chloroform-*d*)

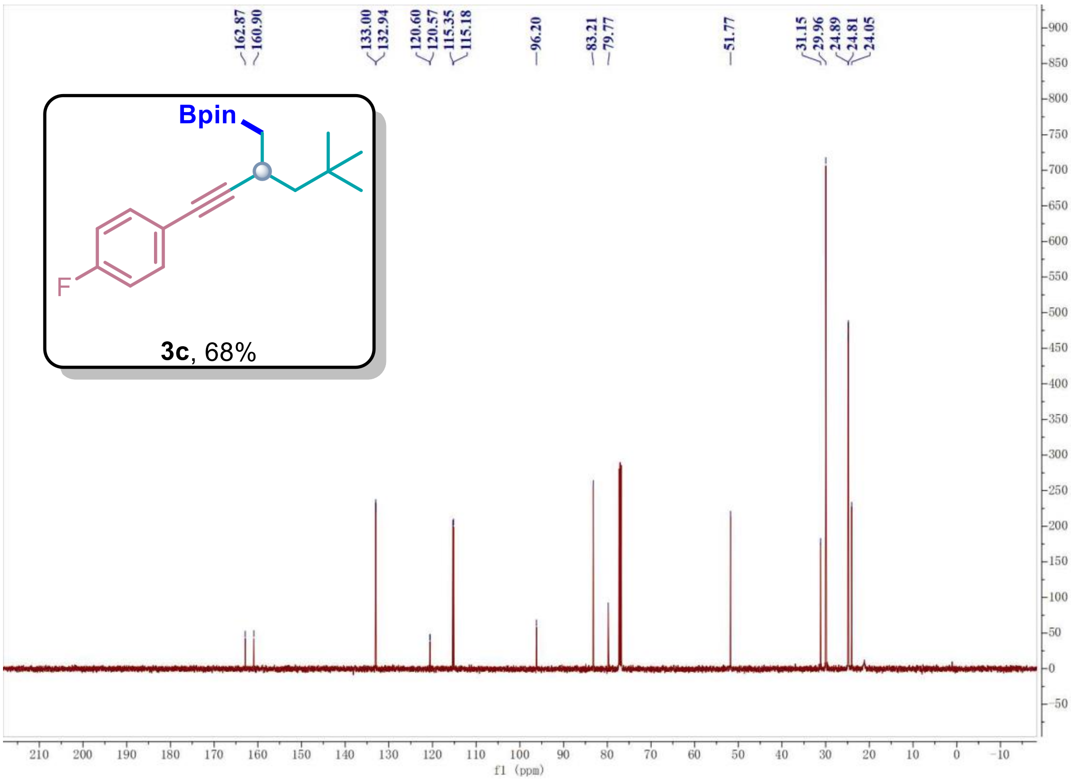

^{11}B NMR (160 MHz, Chloroform-*d*)

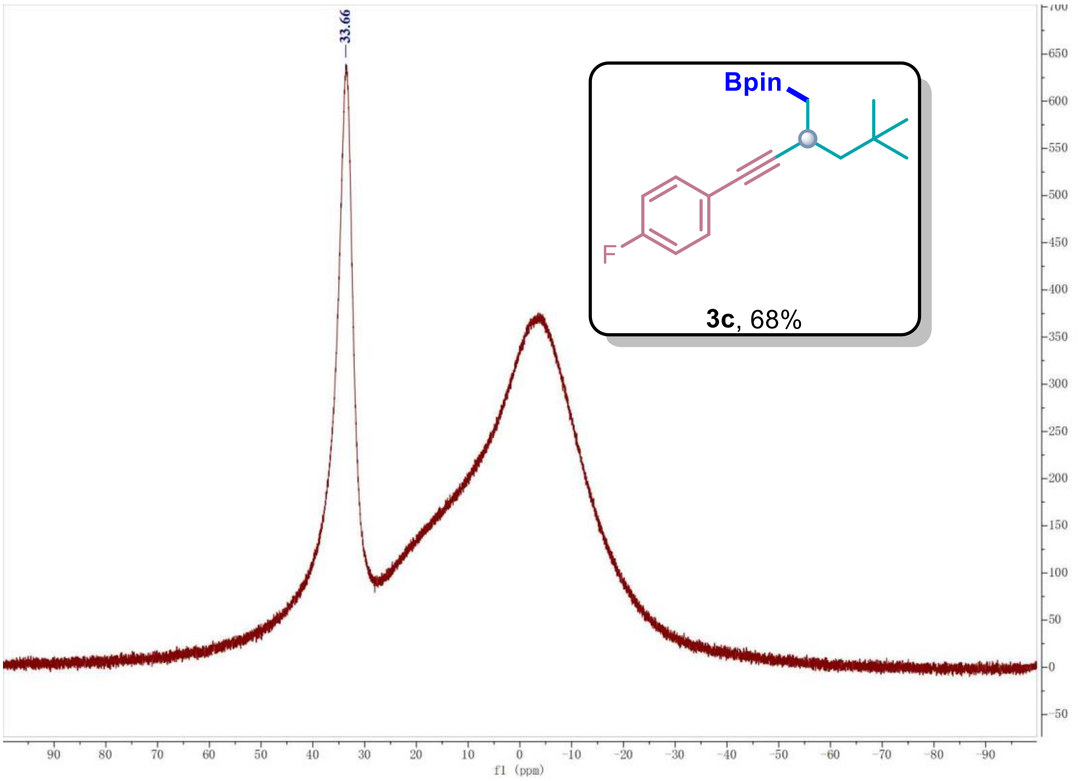

^{19}F NMR (471 MHz, Chloroform-*d*)

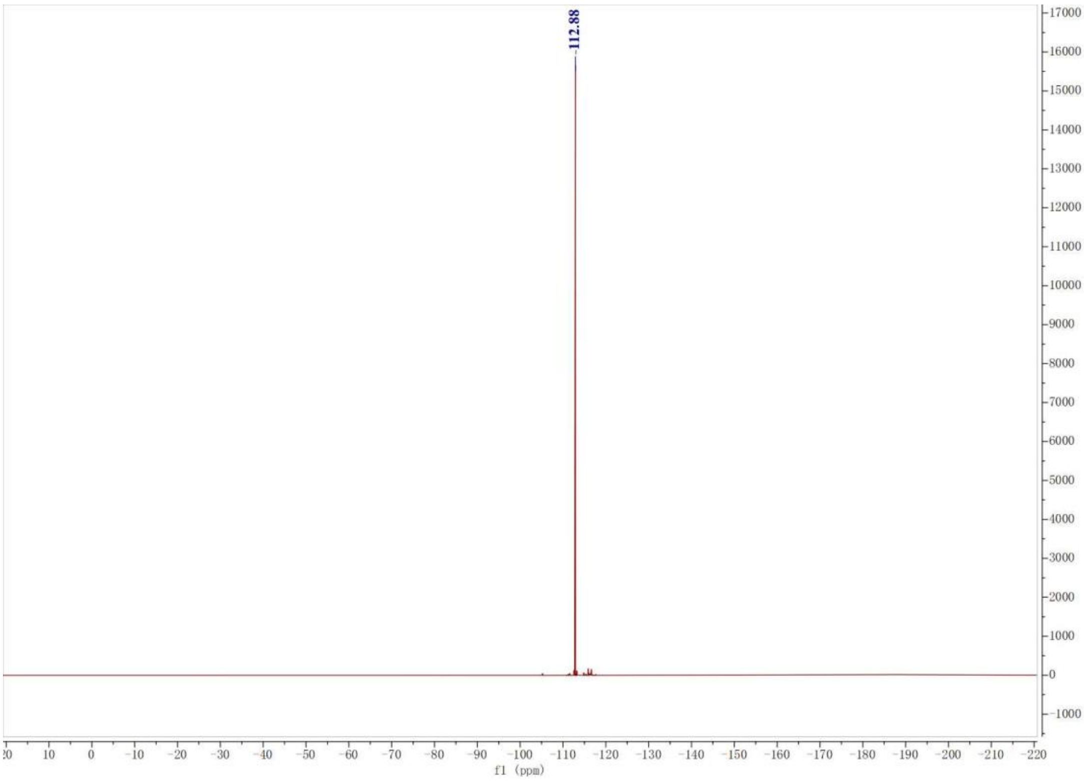

2-(2-((4-chlorophenyl)ethynyl)-4,4-dimethylpentyl)-4,4,5,5-tetramethyl-1,3,2-dioxaborolane (3d)

^{1}H NMR (500 MHz, Chloroform-*d*)

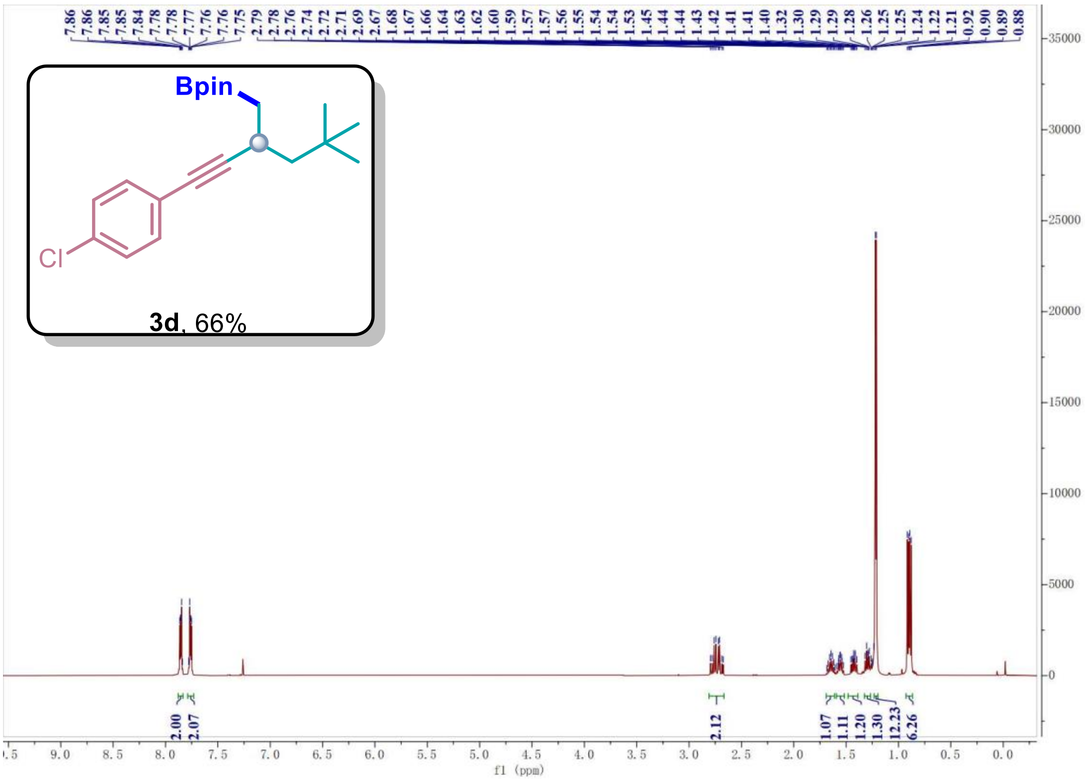

^{13}C NMR (126 MHz, Chloroform-*d*)

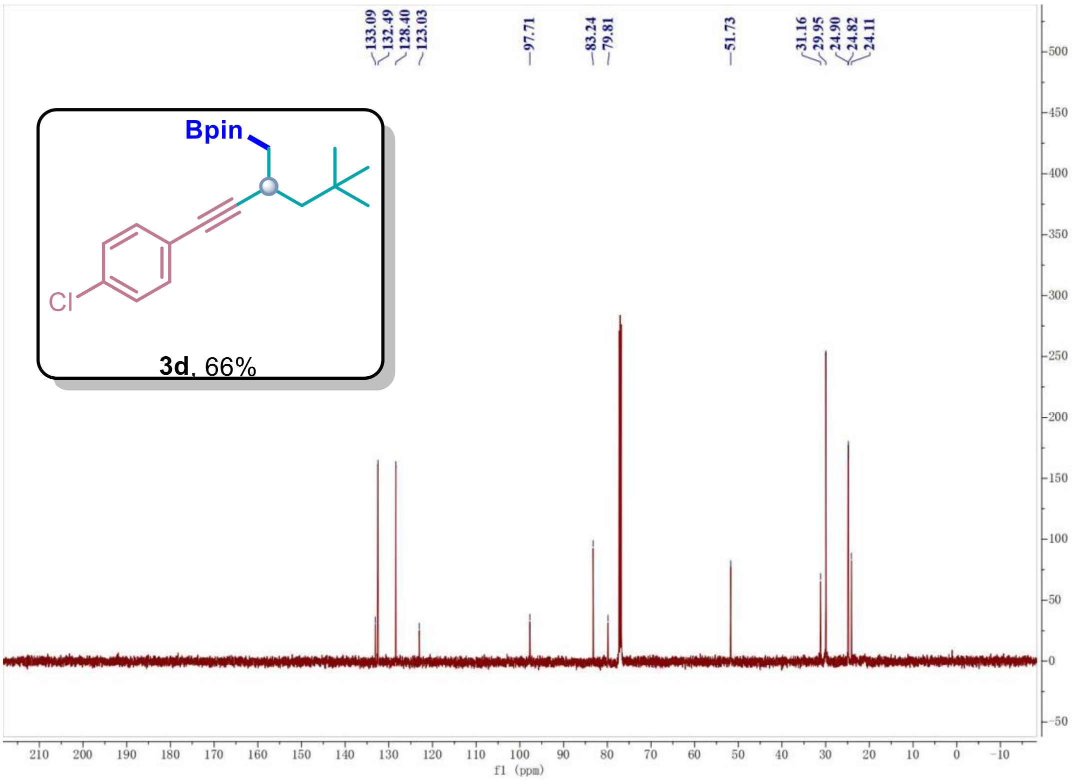

^{11}B NMR (160 MHz, Chloroform-*d*)

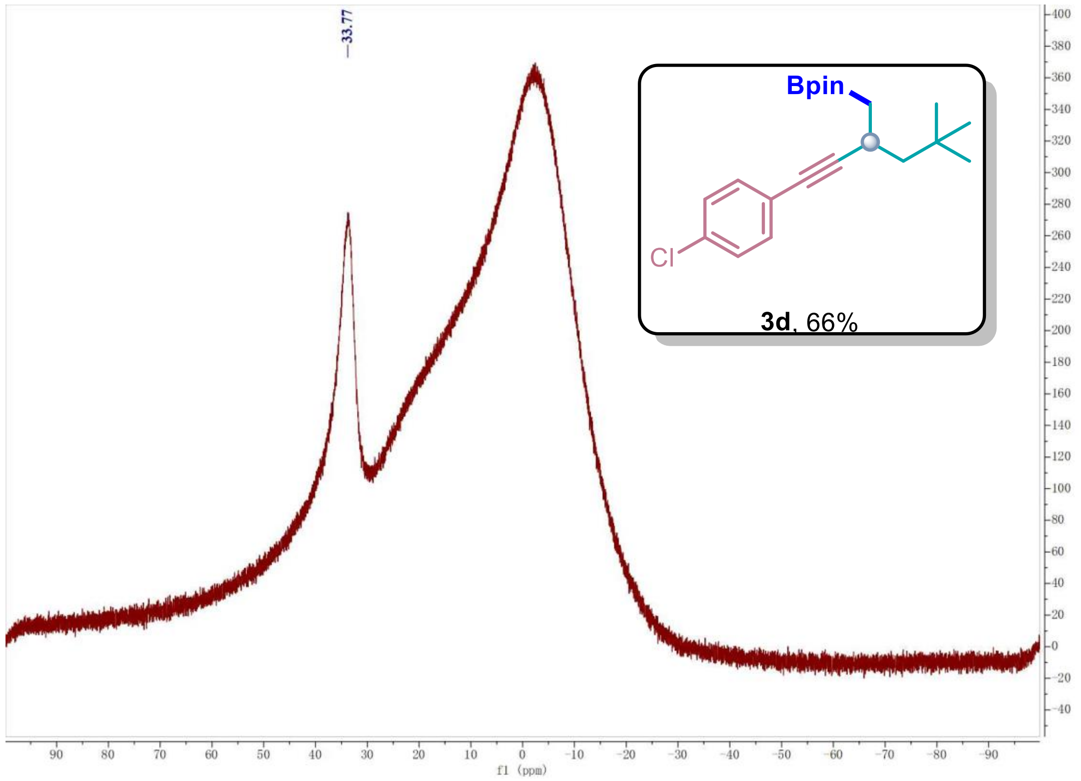

2-(2-((4-bromophenyl)ethynyl)-4,4-dimethylpentyl)-4,4,5,5-tetramethyl-1,3,2-dioxaborolane (3e)

^{1}H NMR (500 MHz, Chloroform-*d*)

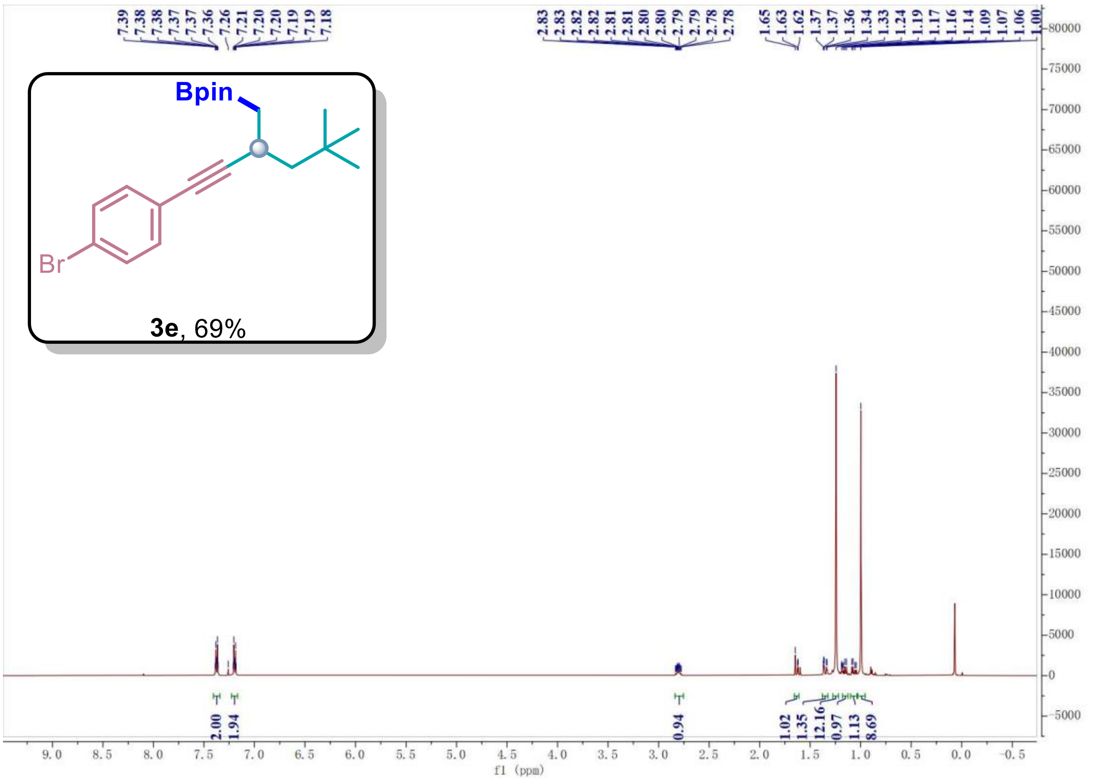

^{13}C NMR (126 MHz, Chloroform-*d*)

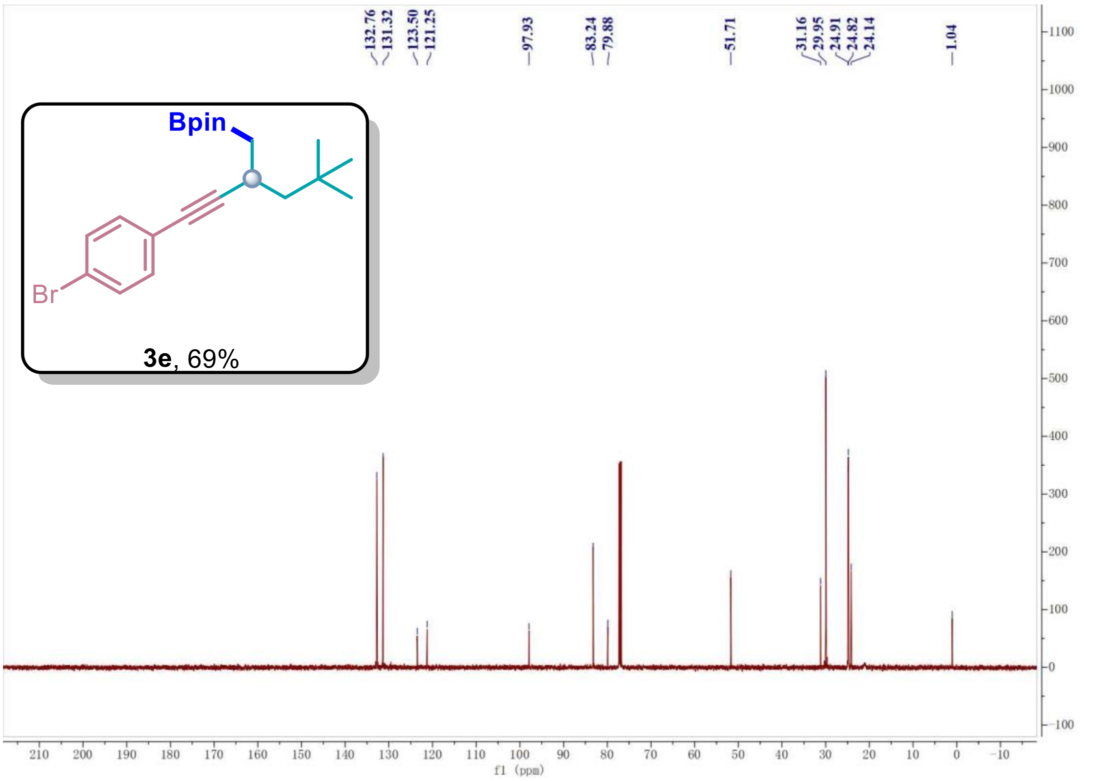

^{11}B NMR (160 MHz, Chloroform-*d*)

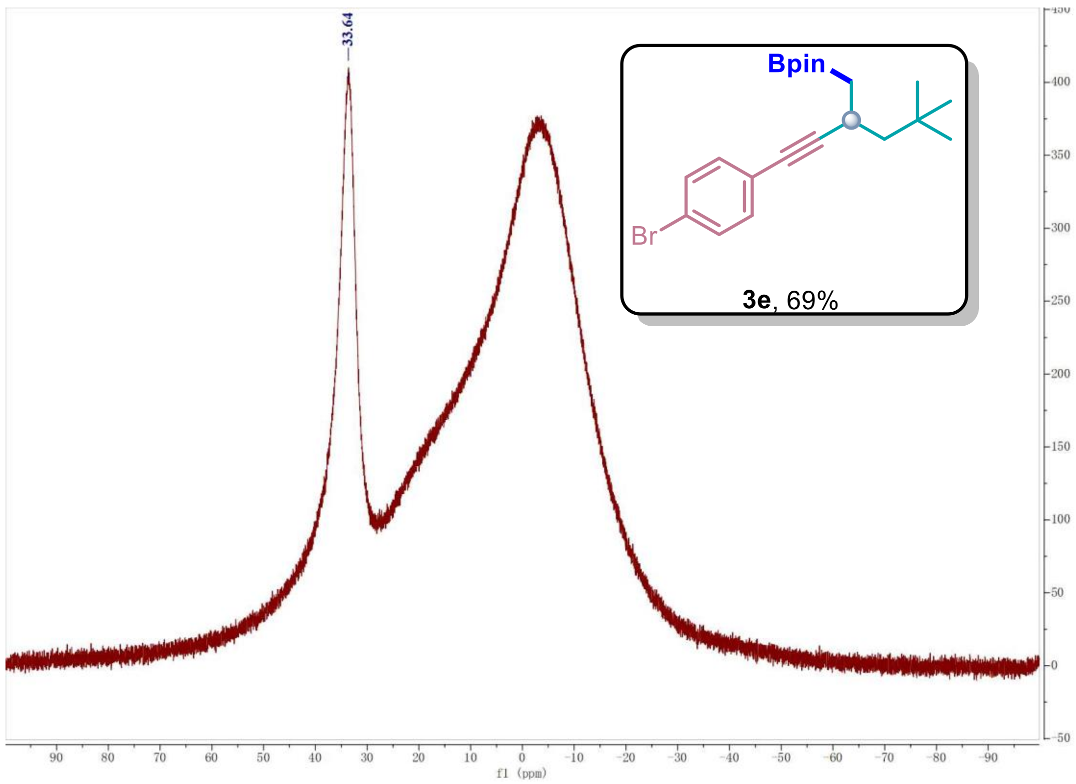

2-(2-((4-methoxyphenyl)ethynyl)-4,4-dimethylpentyl)-4,4,5,5-tetramethyl-1,3,2-dioxaborolane (3f)

^{1}H NMR (500 MHz, Chloroform-*d*)

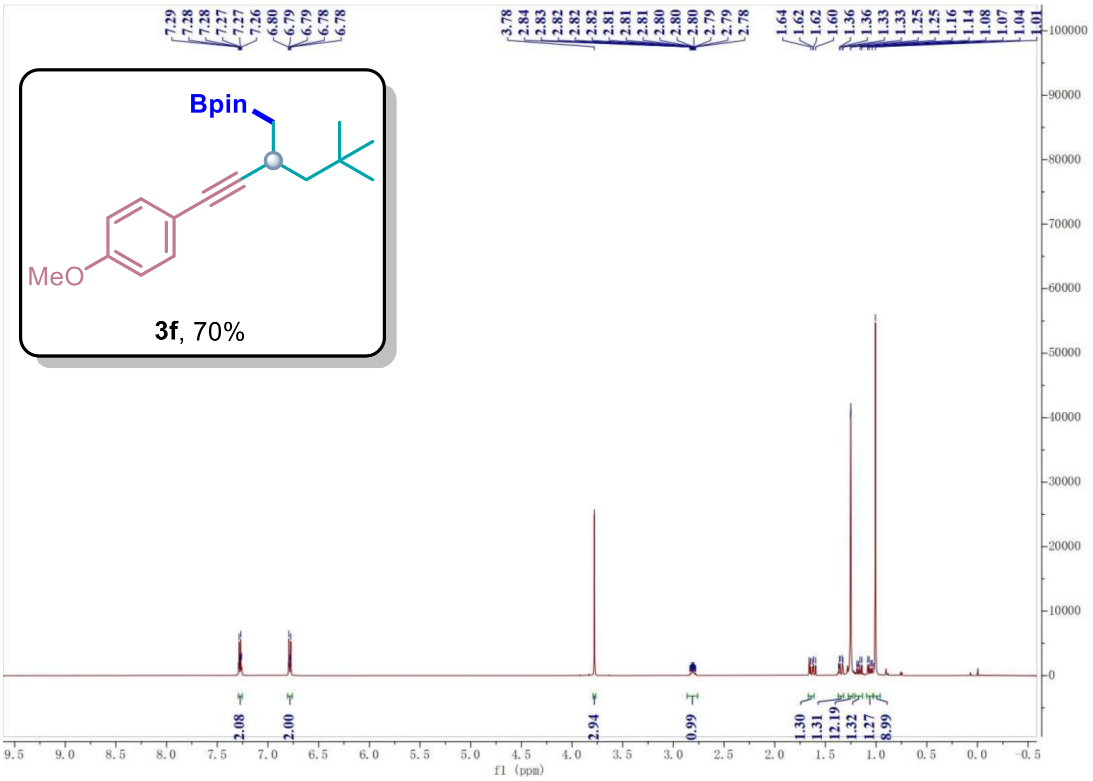

^{13}C NMR (126 MHz, Chloroform-*d*)

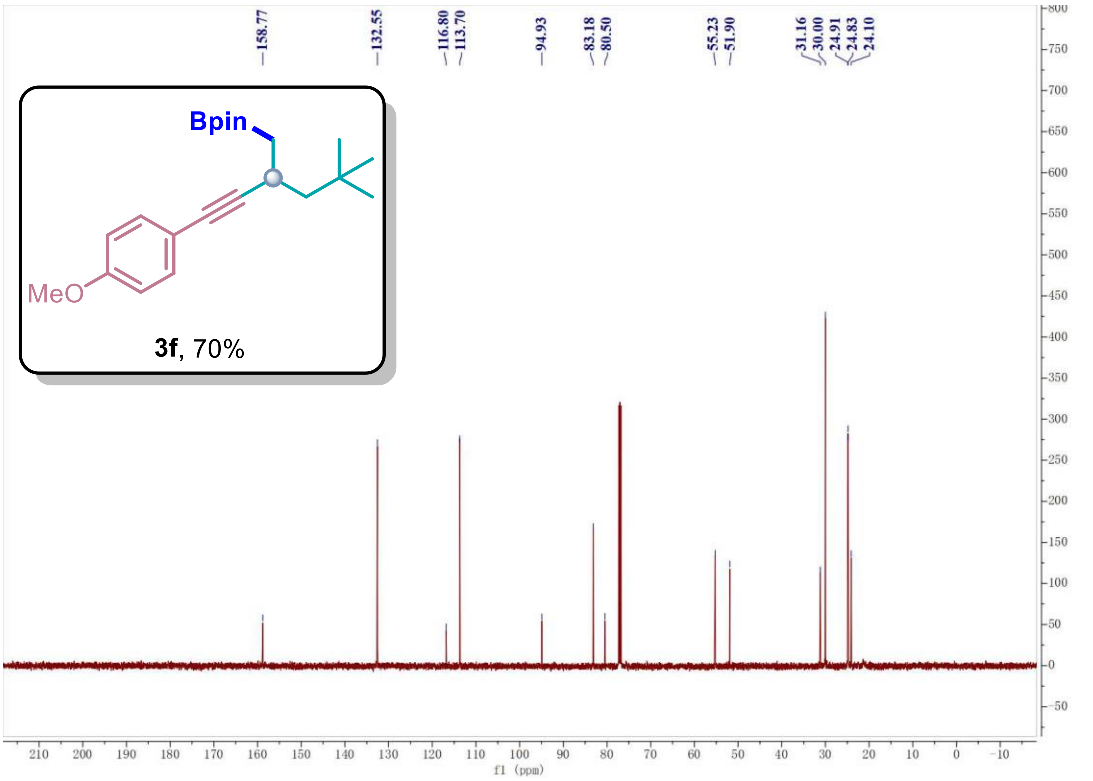

^{11}B NMR (160 MHz, Chloroform-d)

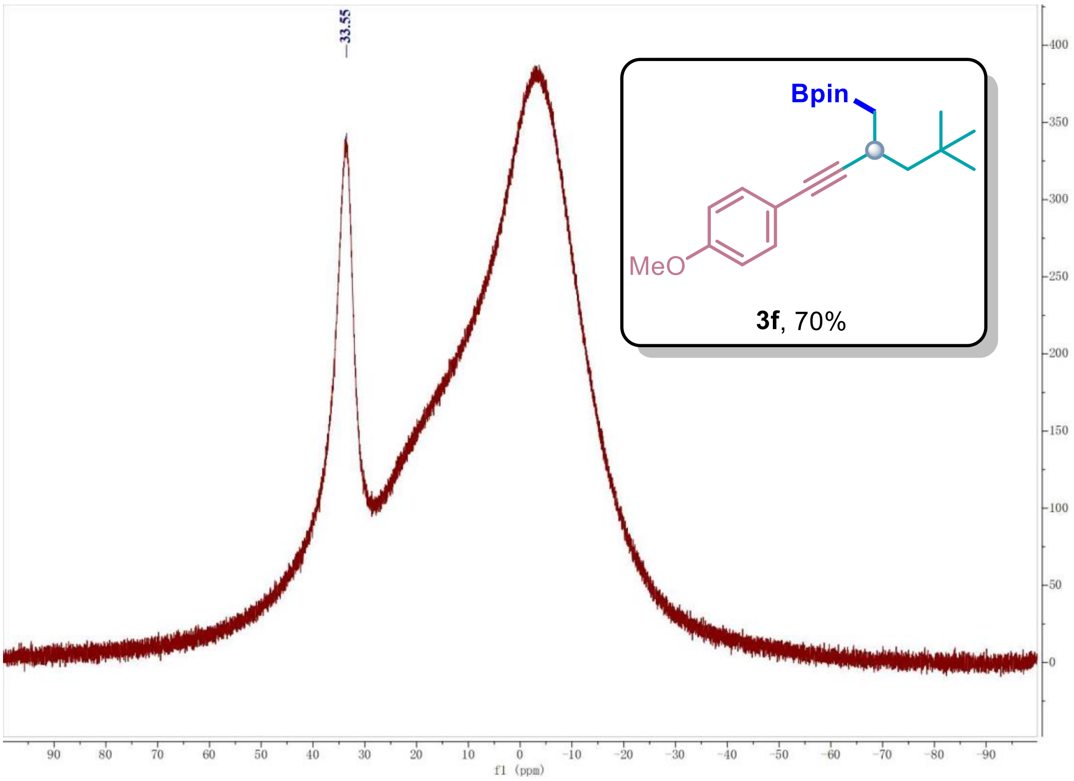

2-(2-([1,1'-biphenyl]-4-ylethynyl)-4,4-dimethylpentyl)-4,4,5,5-tetramethyl-1,3,2-dioxaborolane (3g)

^{1}H NMR (500 MHz, Chloroform-*d*)

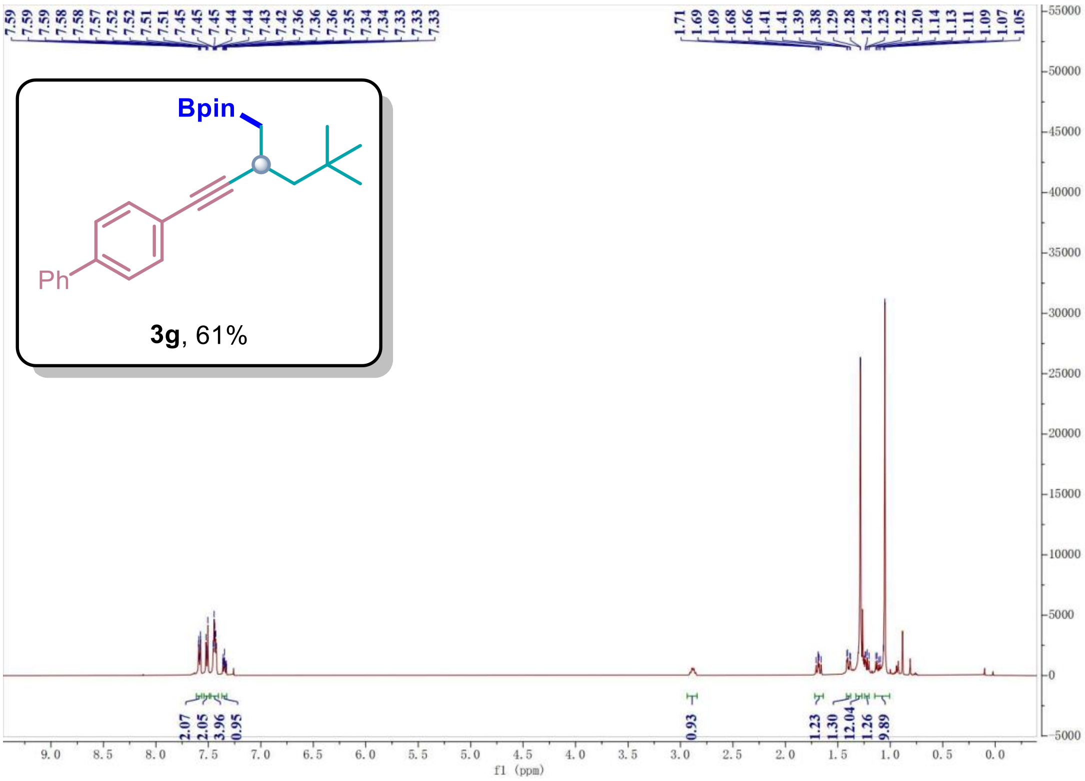

^{13}C NMR (126 MHz, Chloroform-*d*)

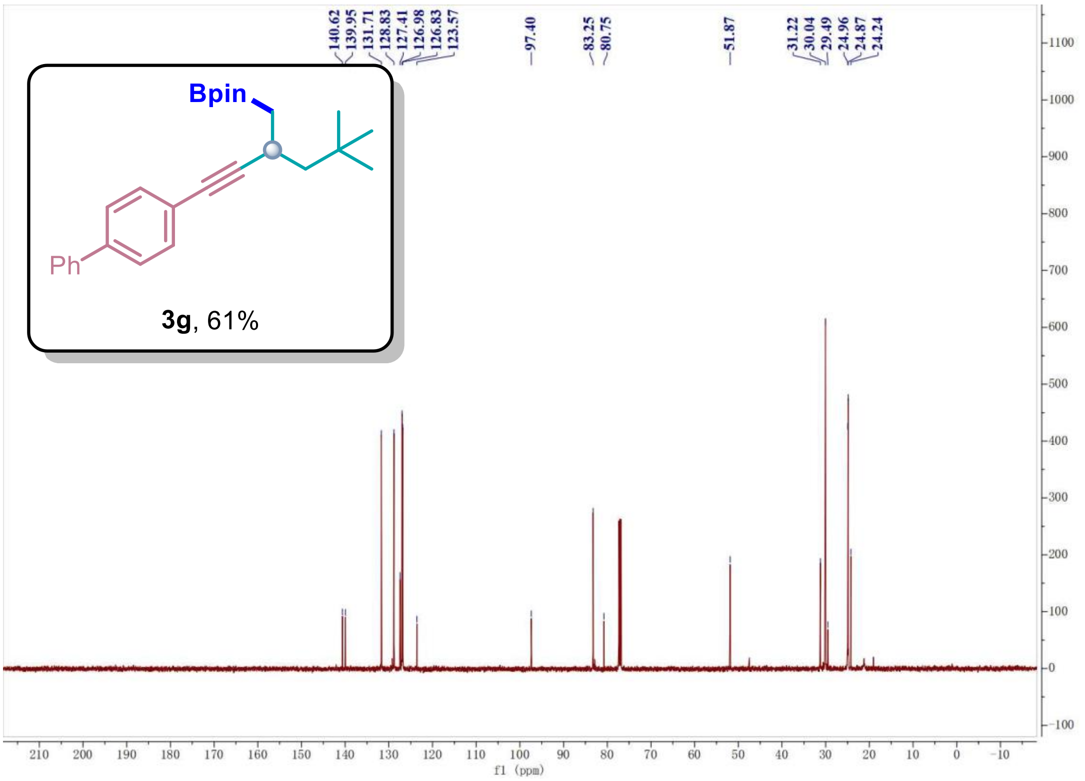

^{11}B NMR (160 MHz, Chloroform-*d*)

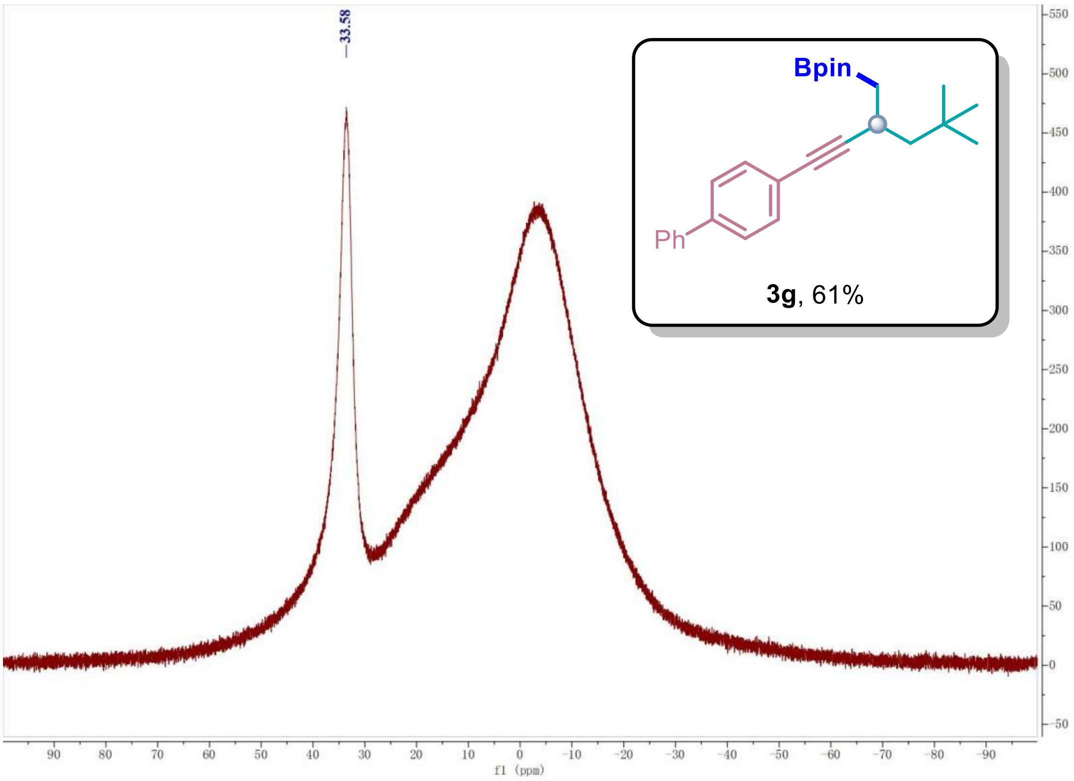

2-(2-cyclopentyl-4-phenylbut-3-yn-1-yl)-4,4,5,5-tetramethyl-1,3,2-dioxaborolane (3h)

^{1}H NMR (500 MHz, Chloroform-*d*)

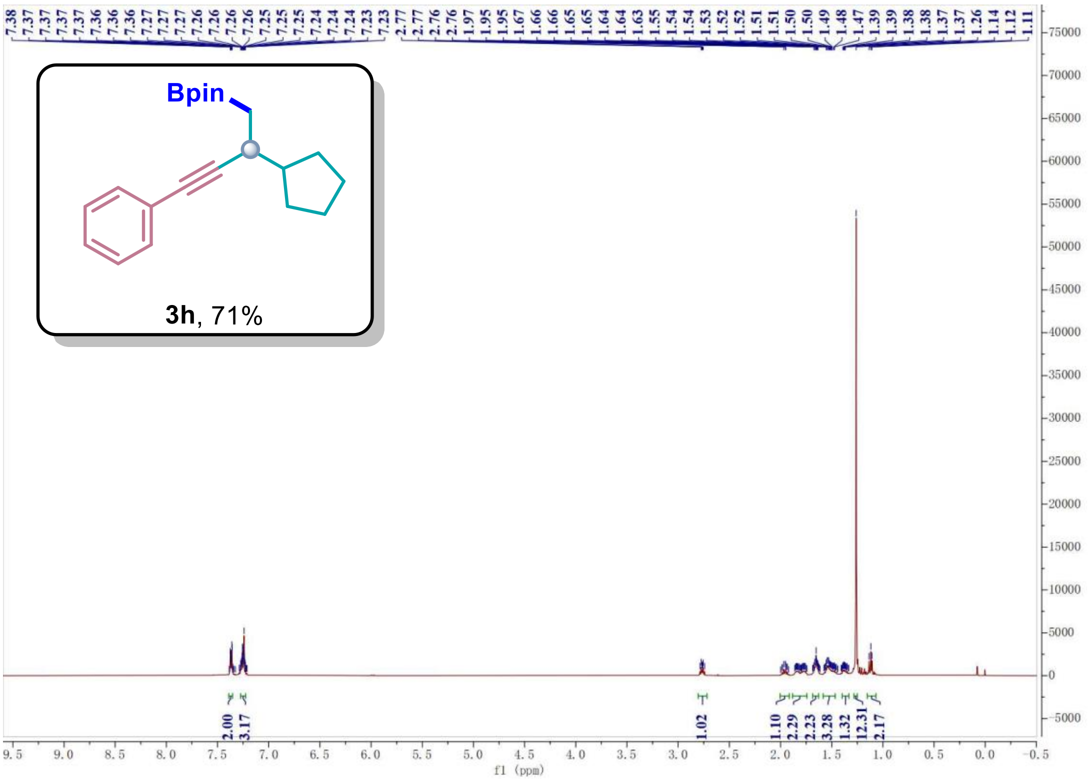

^{13}C NMR (126 MHz, Chloroform-*d*)

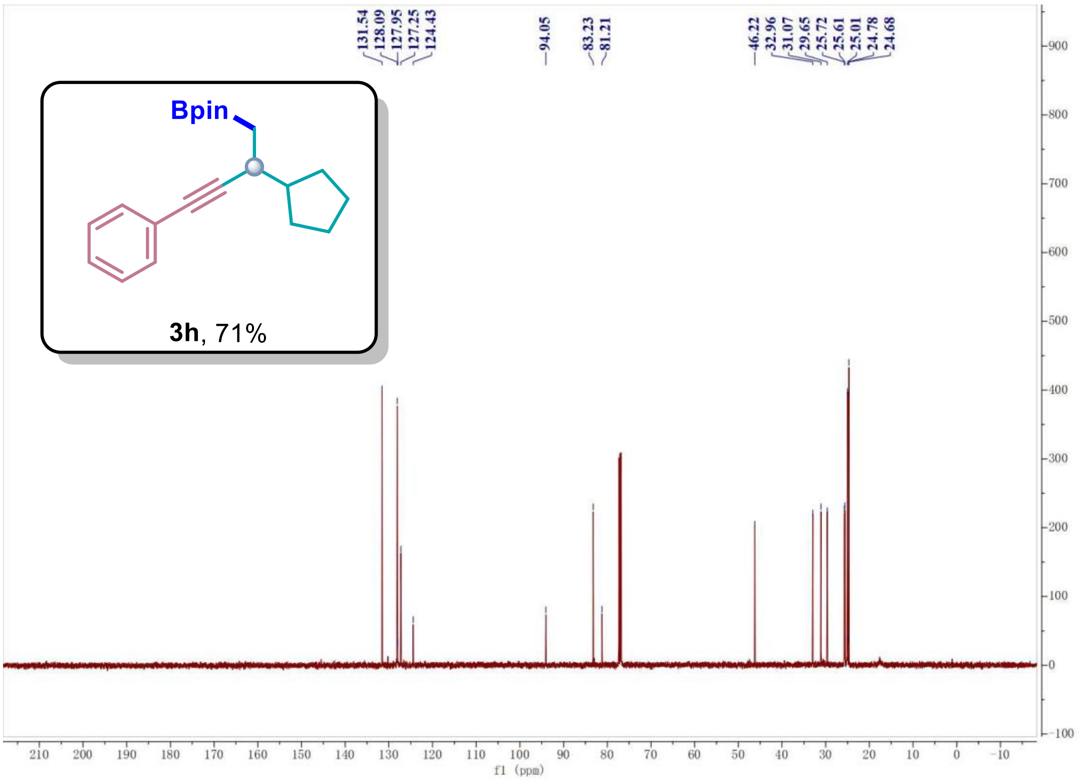

^{11}B NMR (160 MHz, Chloroform-*d*)

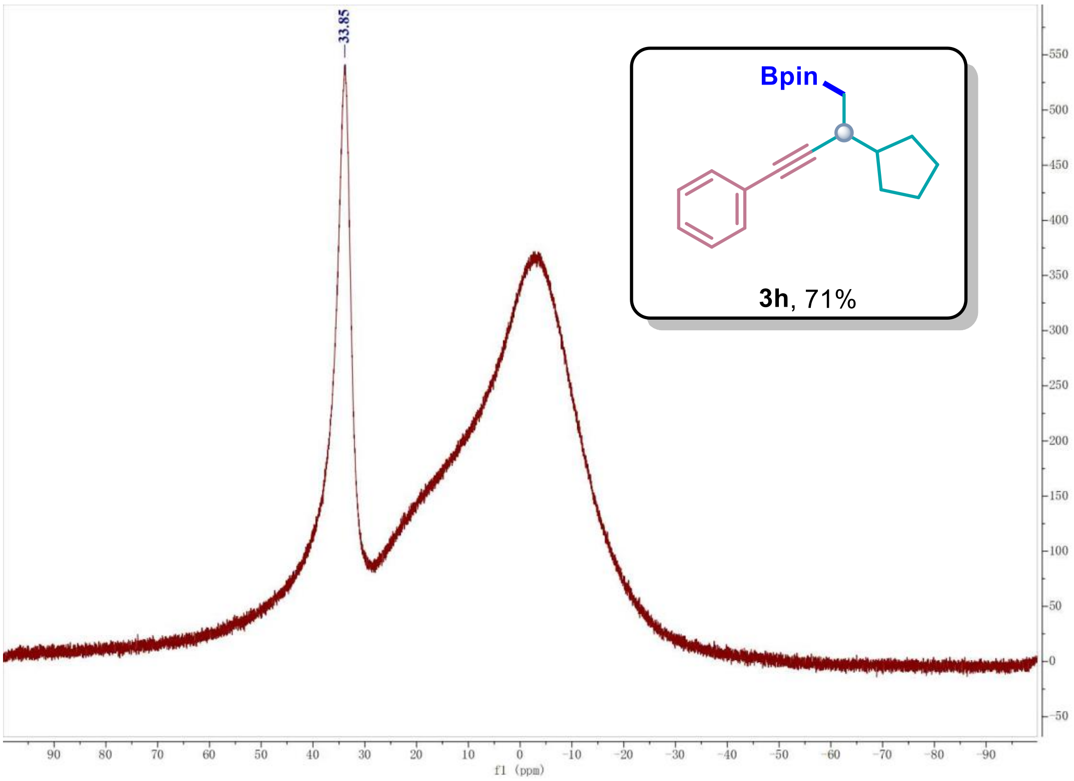

2-(2-cyclohexyl-4-phenylbut-3-yn-1-yl)-4,4,5,5-tetramethyl-1,3,2-dioxaborolane (3i)

^{1}H NMR (500 MHz, Chloroform-*d*)

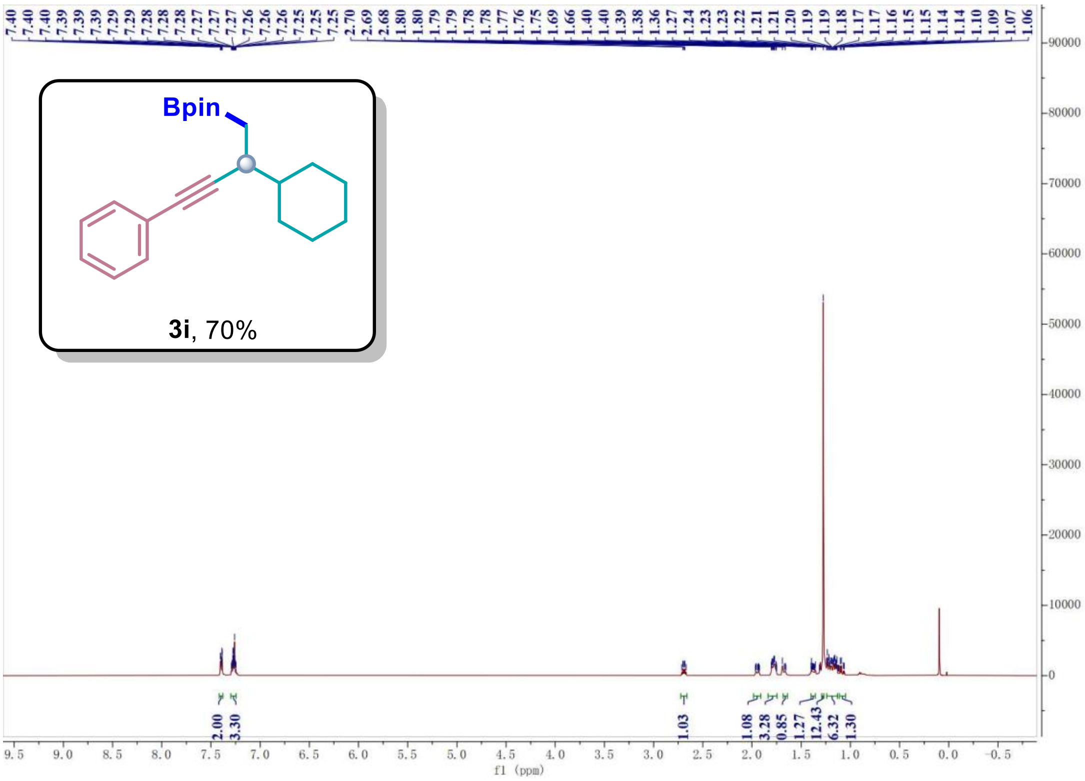

^{13}C NMR (126 MHz, Chloroform-*d*)

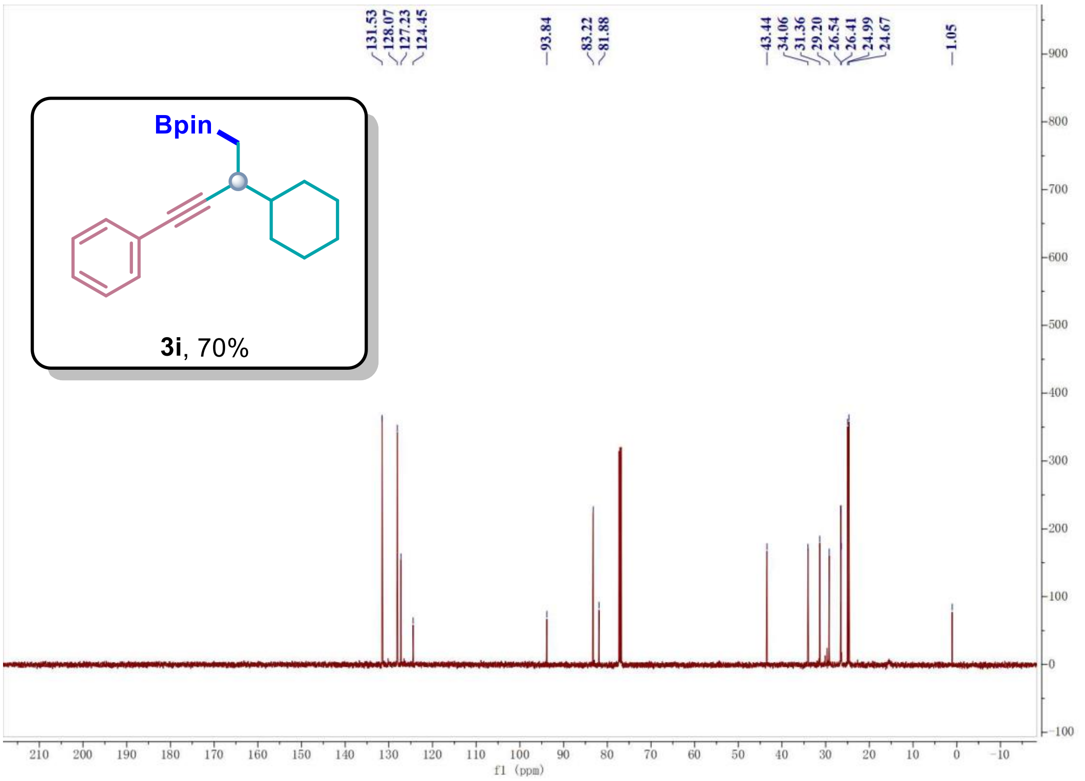

^{11}B NMR (160 MHz, Chloroform-*d*)

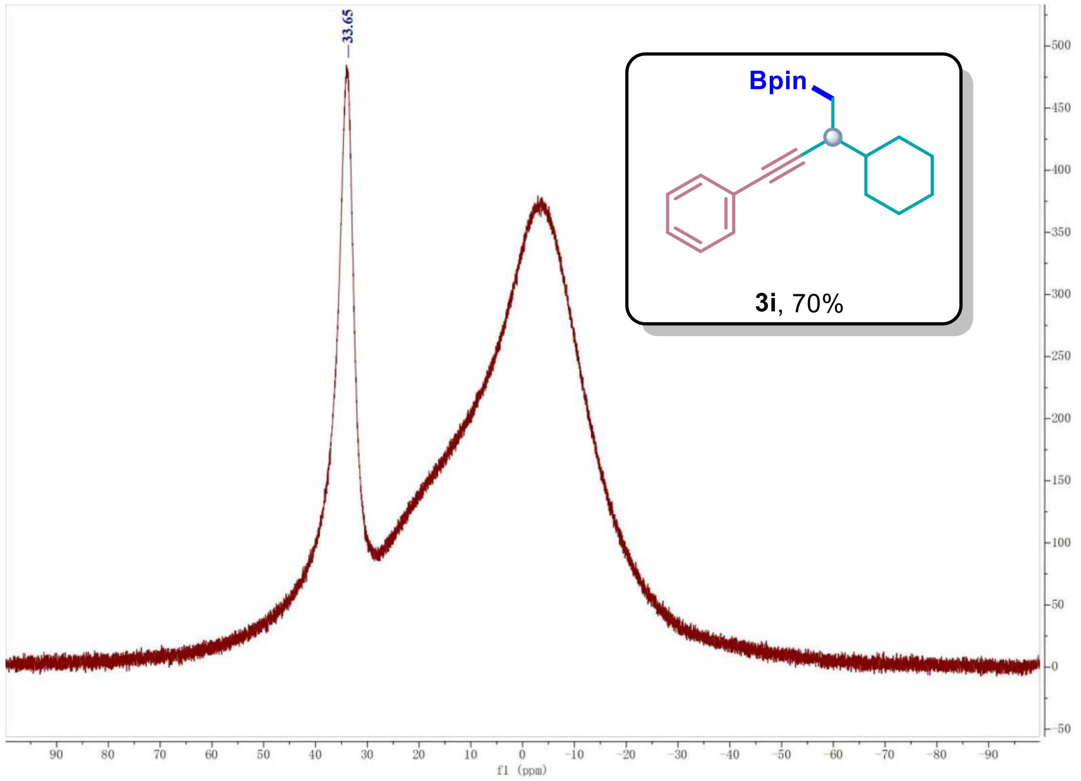

2-(3-ethyl-2-(phenylethynyl)pentyl)-4,4,5,5-tetramethyl-1,3,2-dioxaborolane (3j)

^{1}H NMR (500 MHz, Chloroform-*d*)

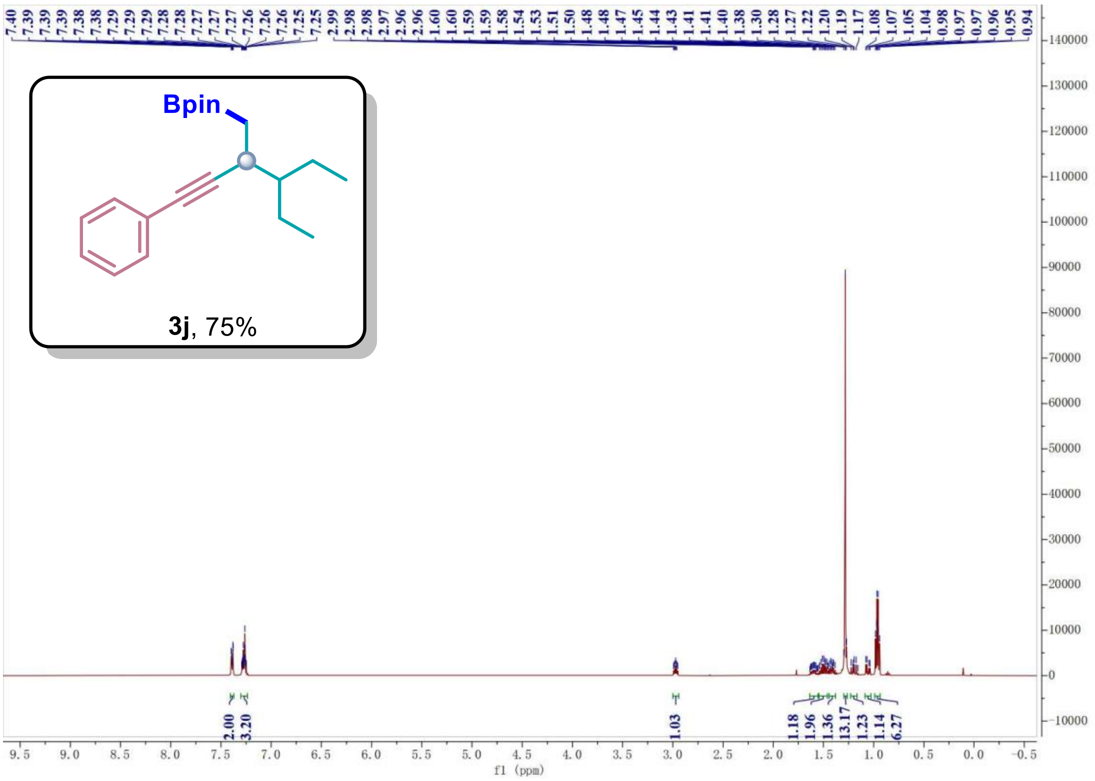

^{13}C NMR (126 MHz, Chloroform-*d*)

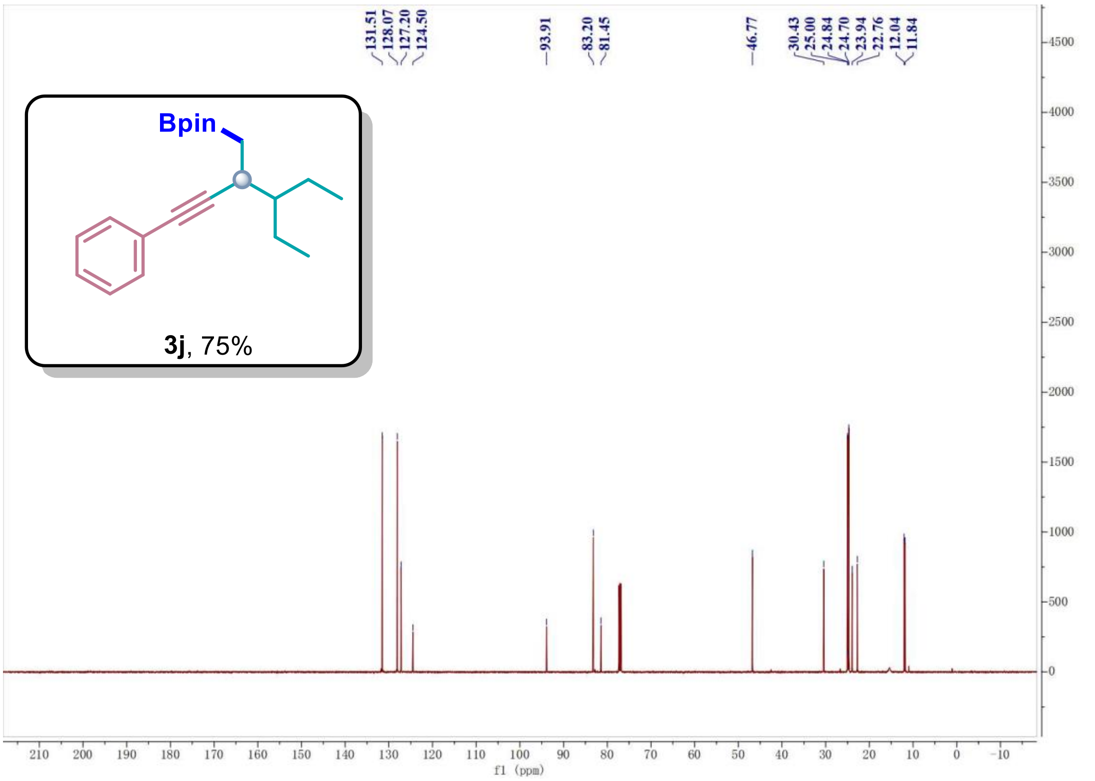

^{11}B NMR (160 MHz, Chloroform-*d*)

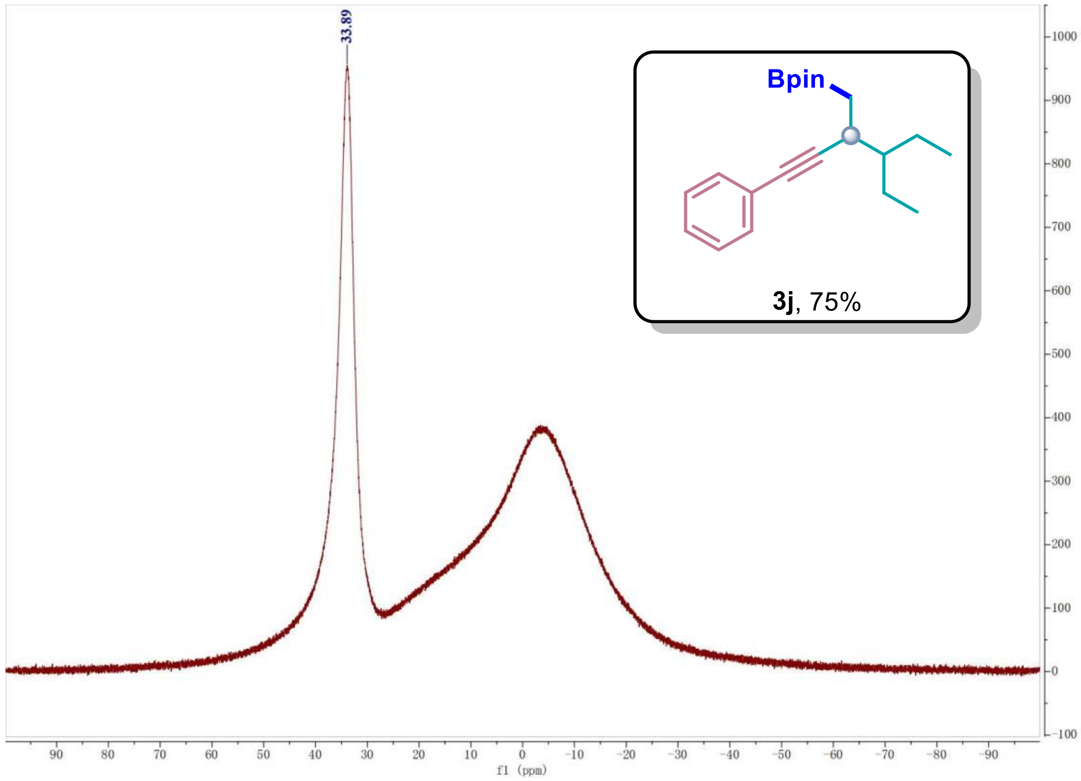

4,4,5,5-tetramethyl-2-(3-methyl-2-(phenylethynyl)hexyl)-1,3,2-dioxaborolane (3k)

^{1}H NMR (500 MHz, Chloroform-*d*)

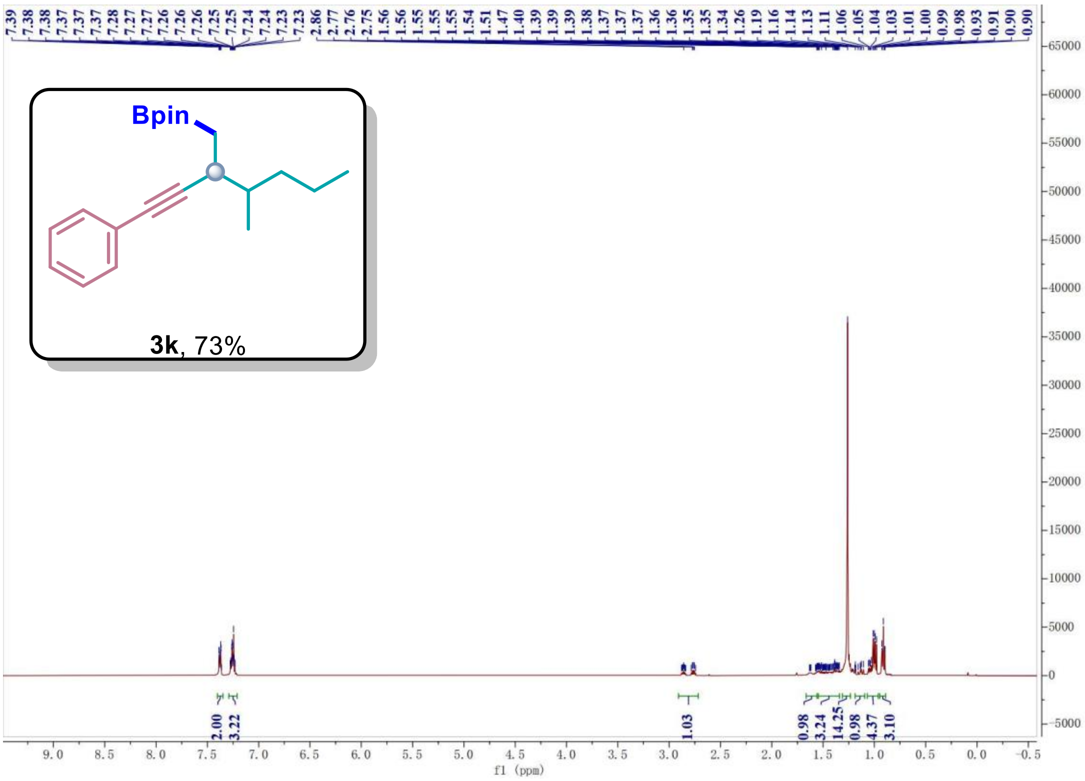

^{13}C NMR (126 MHz, Chloroform-*d*)

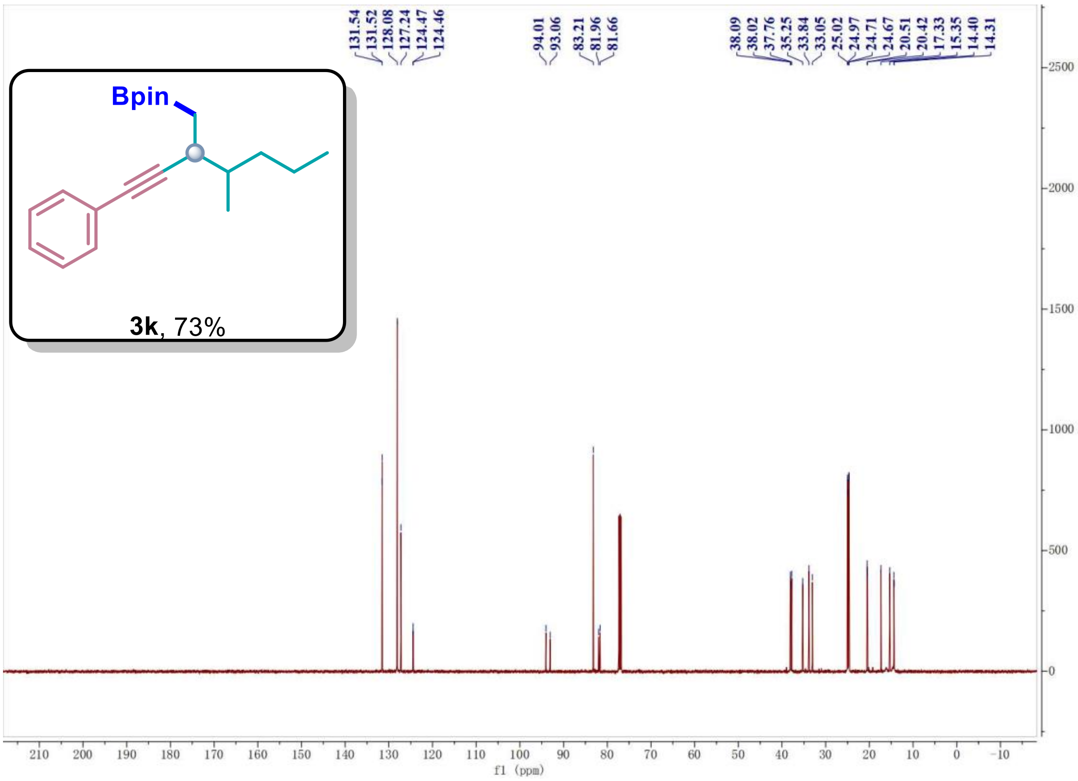

^{11}B NMR (160 MHz, Chloroform-*d*)

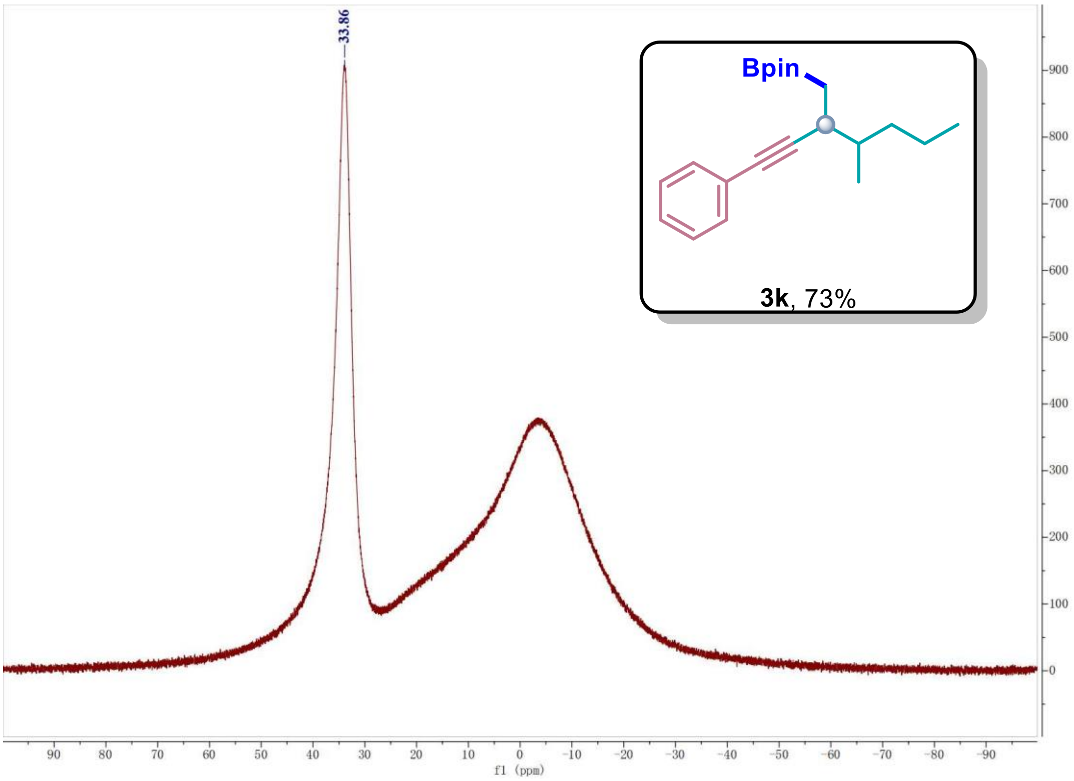

4,4,5,5-tetramethyl-2-(2-phenethyl-4-phenylbut-3-yn-1-yl)-1,3,2-dioxaborolane (3l)

^{1}H NMR (500 MHz, Chloroform-*d*)

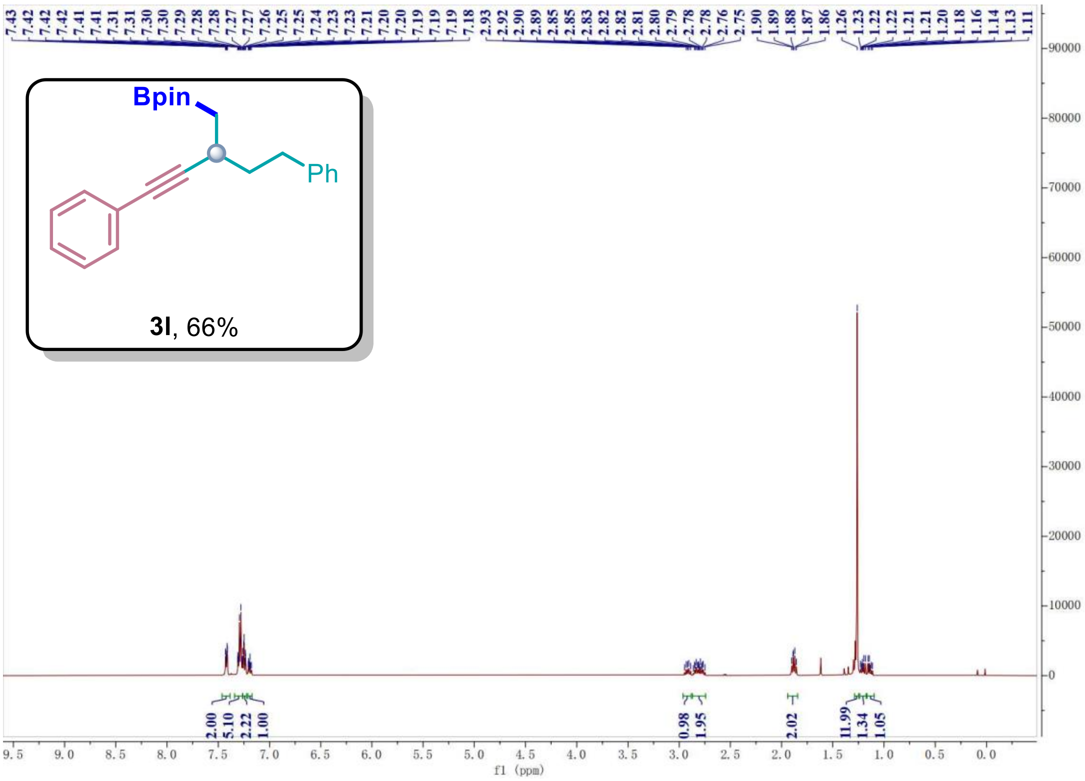

^{13}C NMR (126 MHz, Chloroform-*d*)

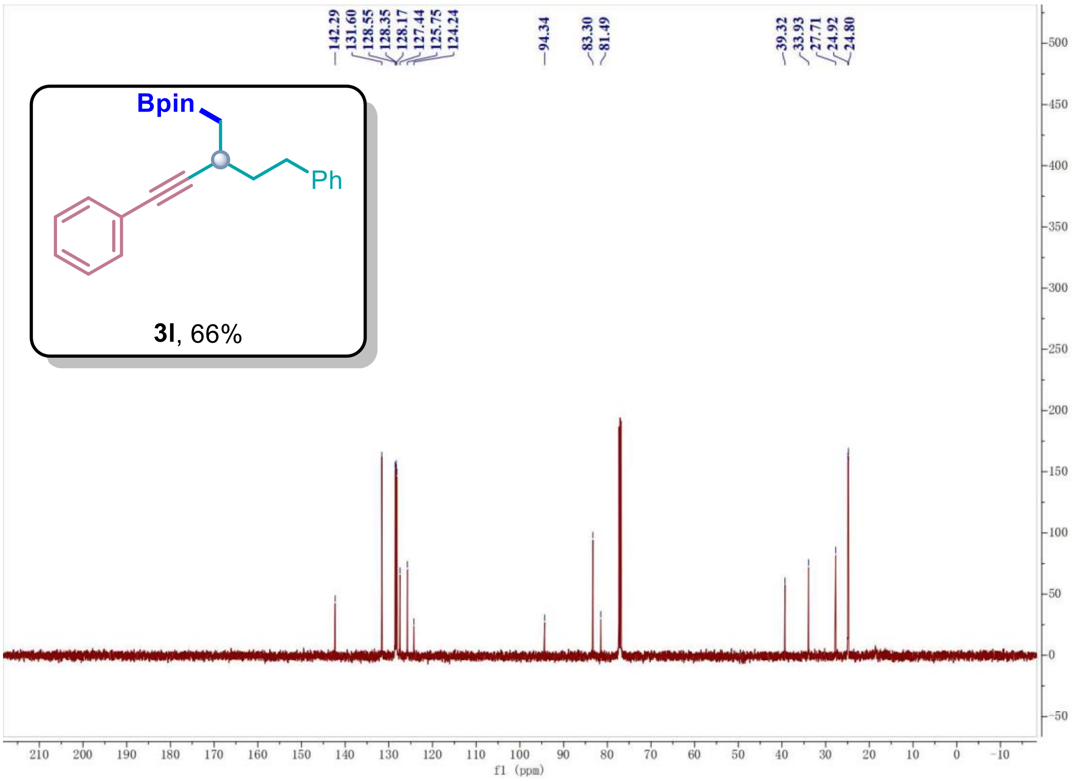

^{11}B NMR (160 MHz, Chloroform-*d*)

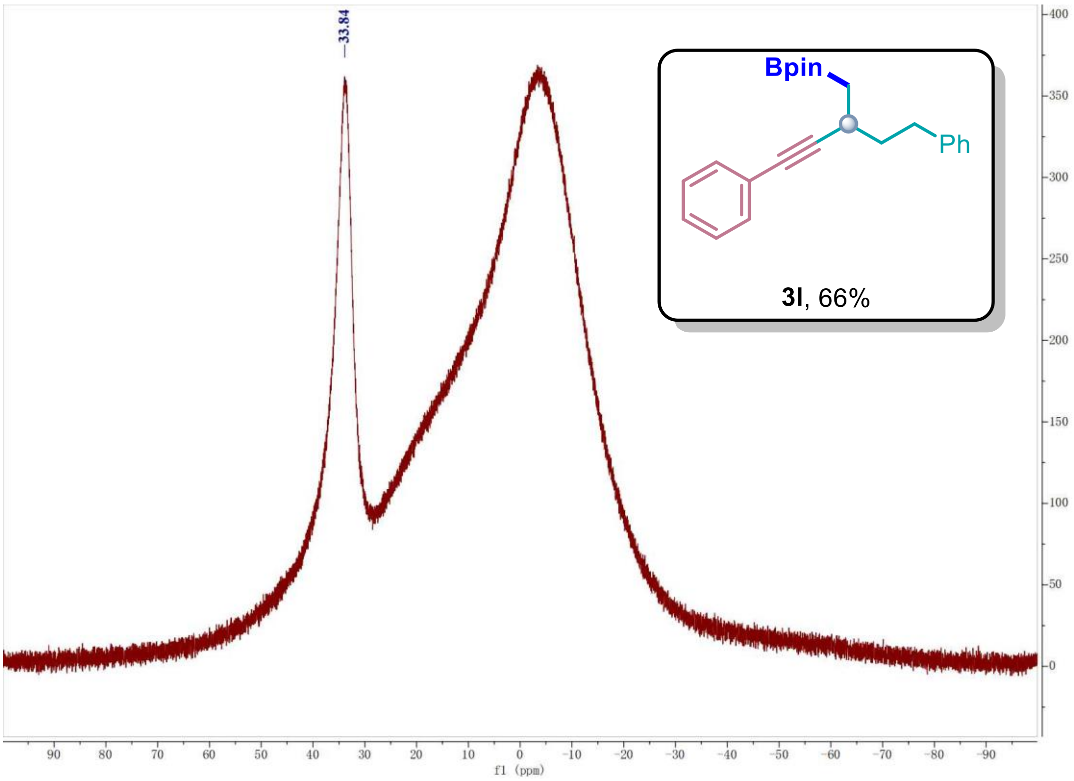

4,4,5,5-tetramethyl-2-(2-(phenylethynyl)pentyl)-1,3,2-dioxaborolane (3m)

^{1}H NMR (500 MHz, Chloroform-*d*)

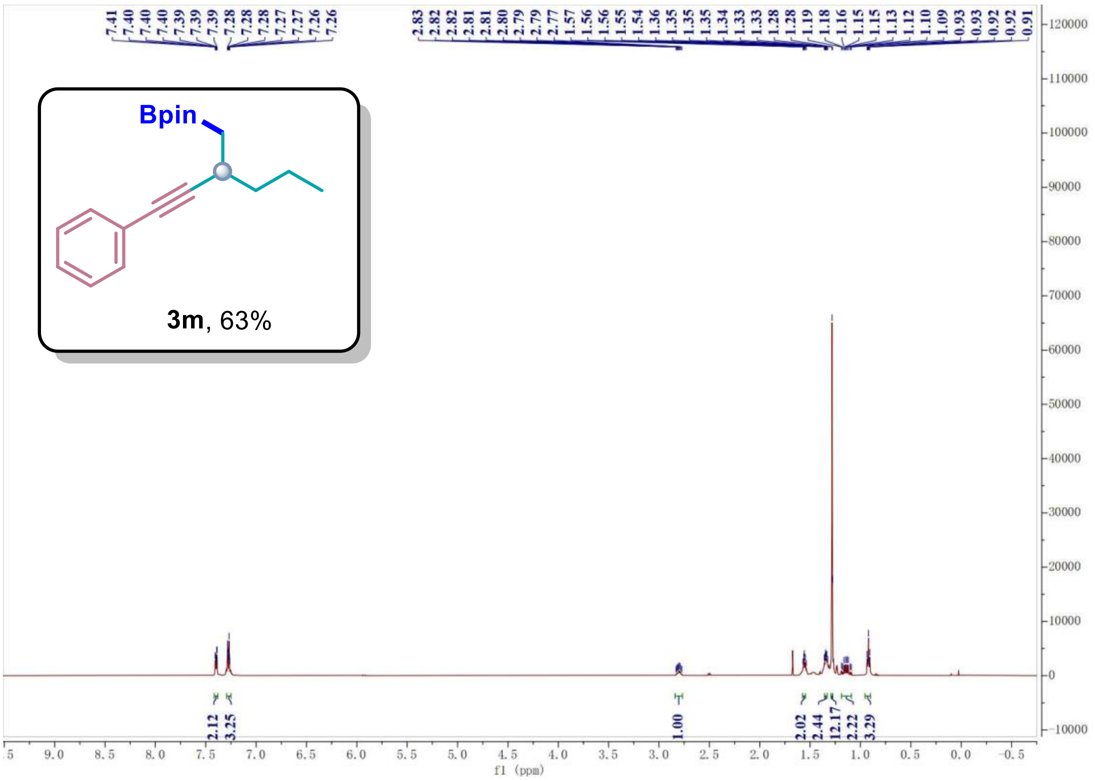

^{13}C NMR (126 MHz, Chloroform-*d*)

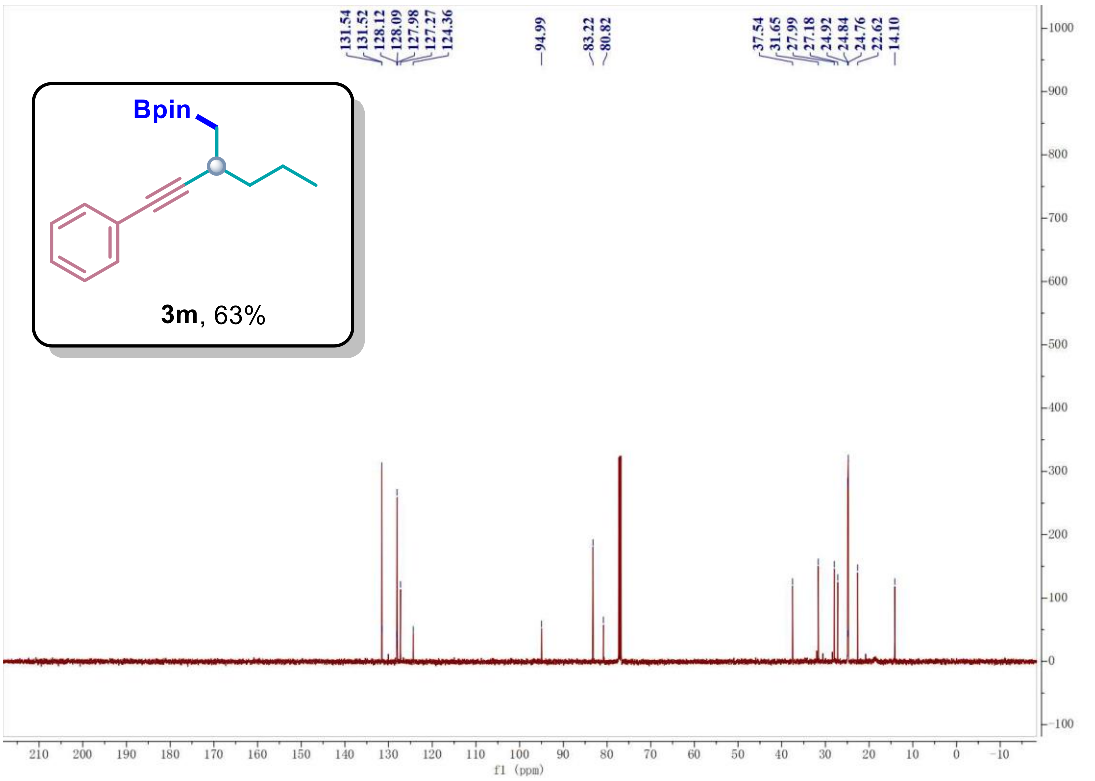

^{11}B NMR (160 MHz, Chloroform-*d*)

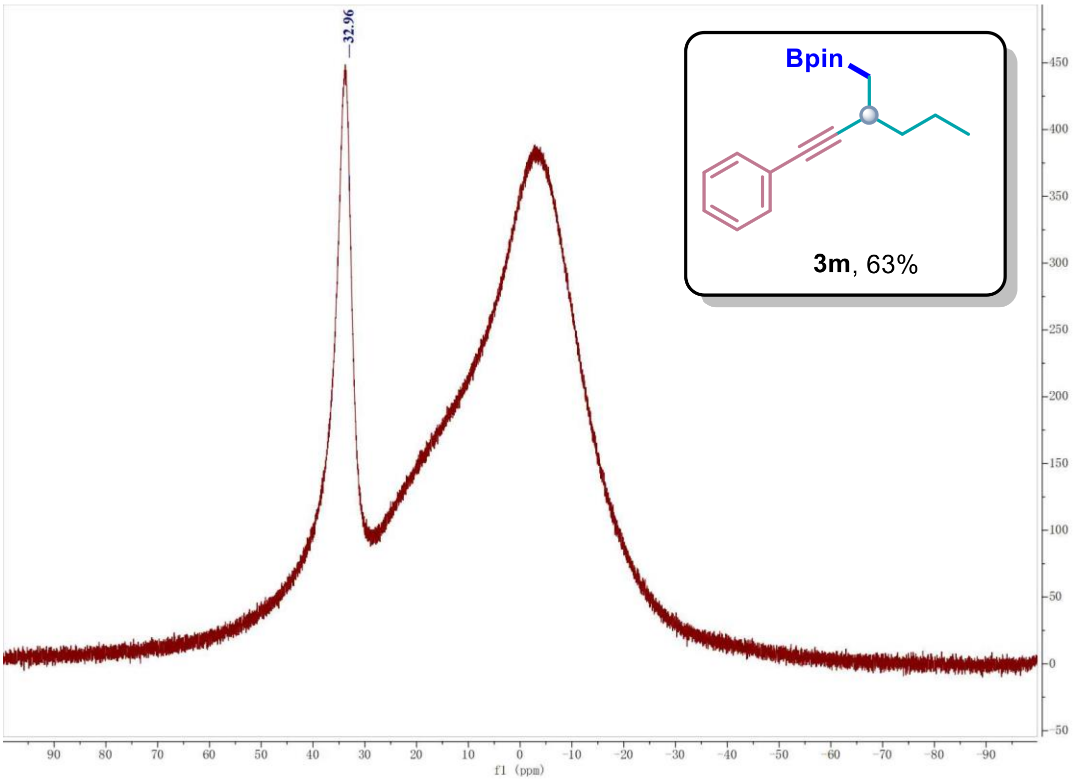

4,4,5,5-tetramethyl-2-(2-(phenylethynyl)octyl)-1,3,2-dioxaborolane (3n)

^{1}H NMR (500 MHz, Chloroform-*d*)

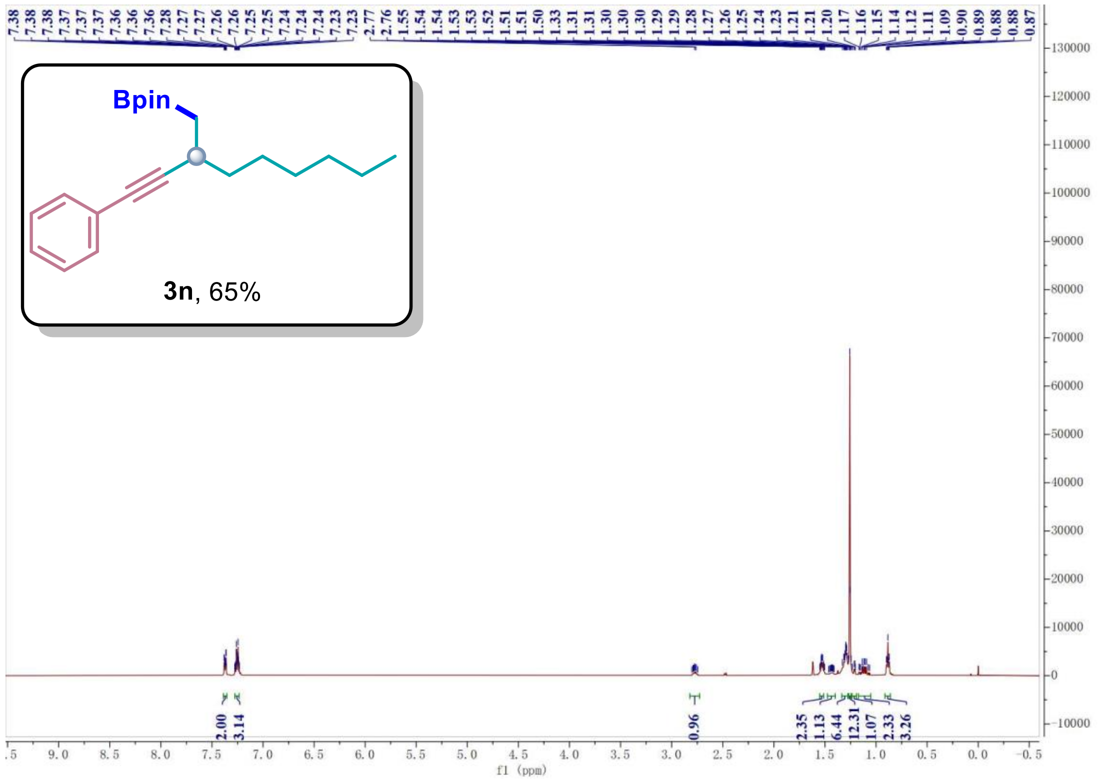

^{13}C NMR (126 MHz, Chloroform-*d*)

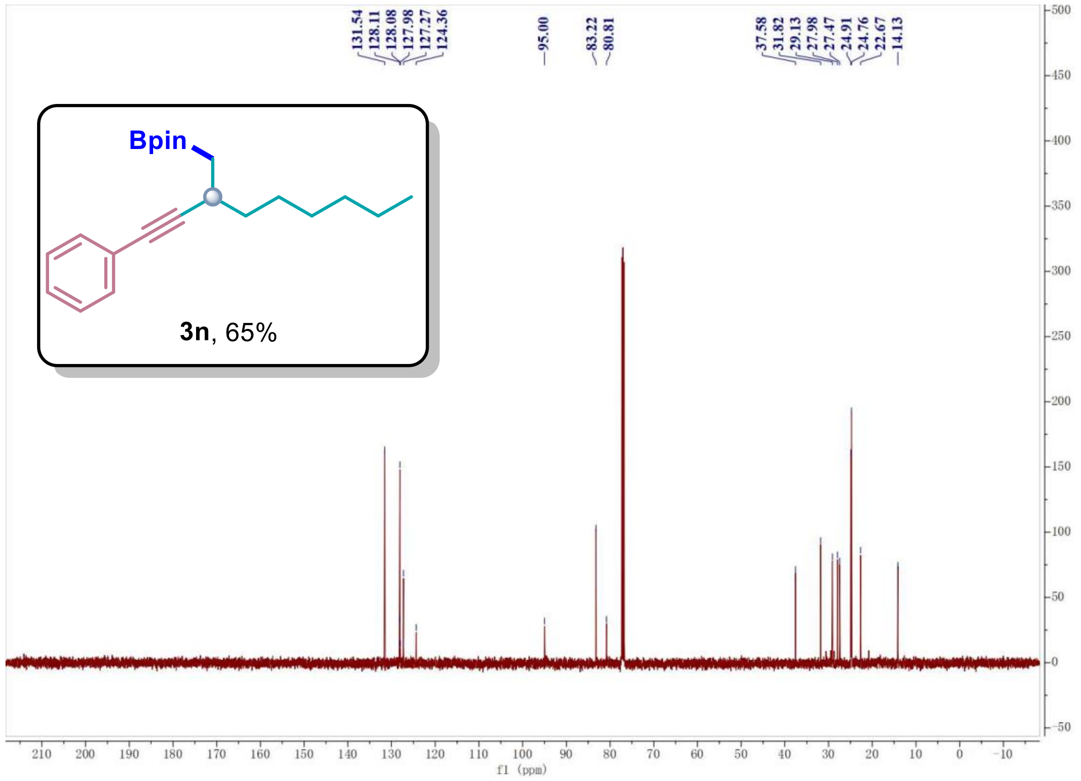

^{11}B NMR (160 MHz, Chloroform-d)

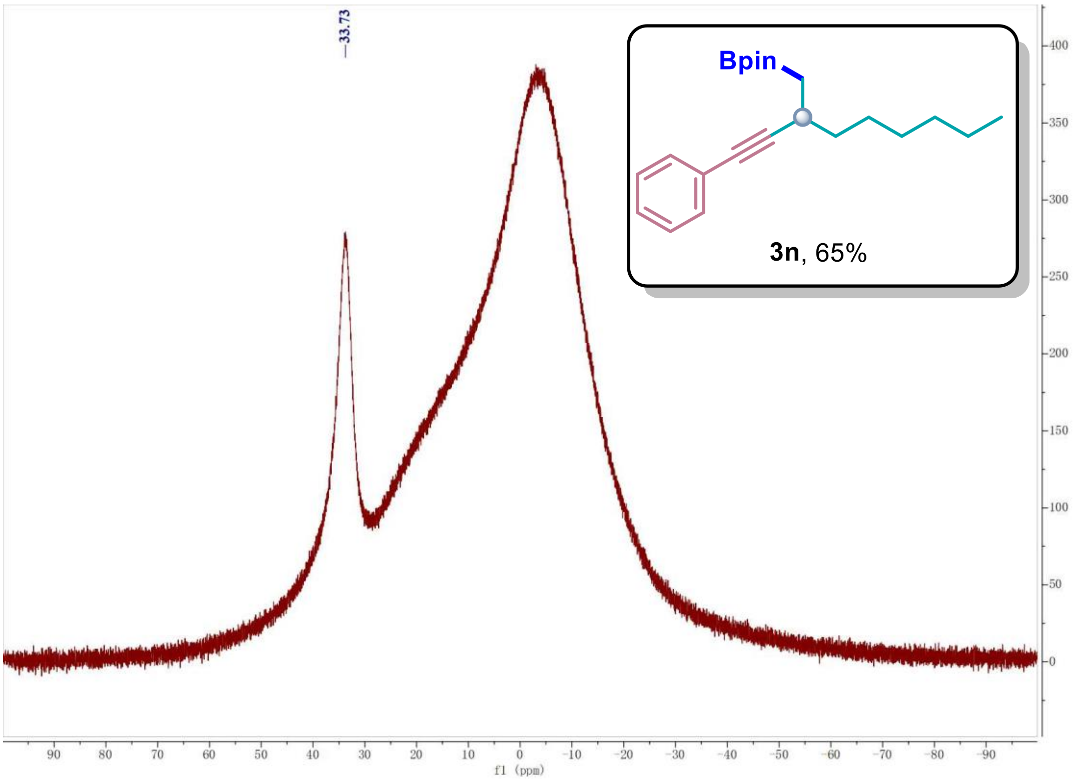

4,4,5,5-tetramethyl-2-(4-methyl-2-(phenylethynyl)pentyl)-1,3,2-dioxaborolane (3o)

^{1}H NMR (500 MHz, Chloroform-*d*)

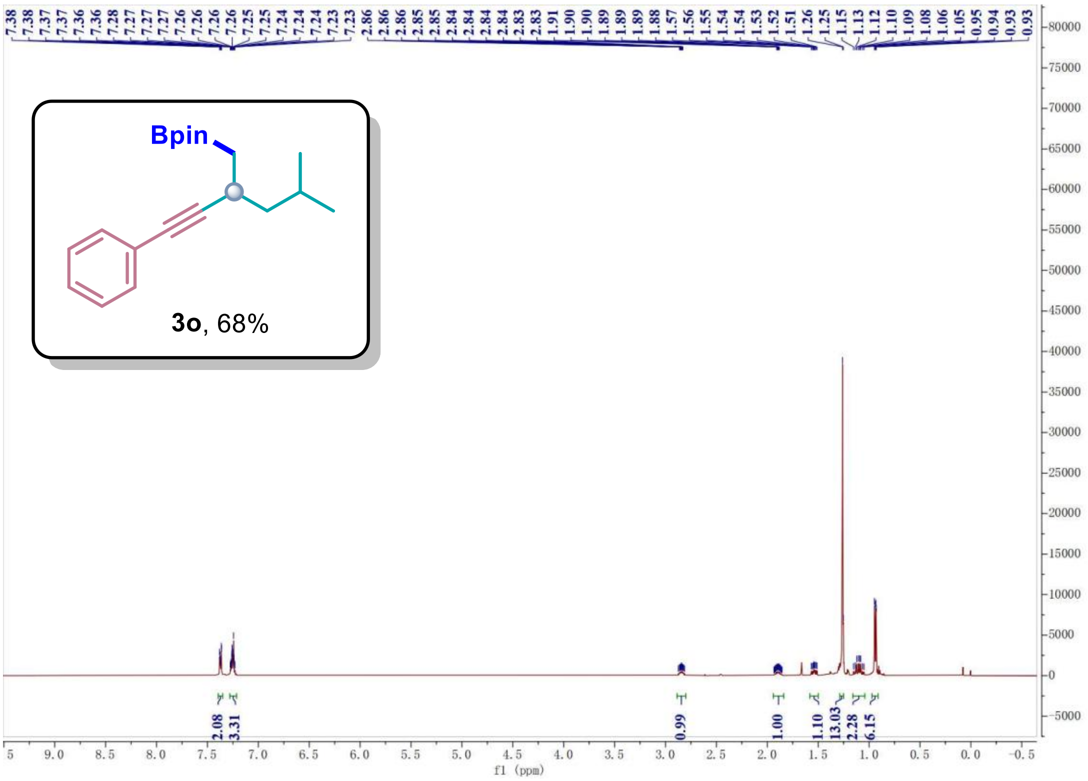

^{13}C NMR (126 MHz, Chloroform-*d*)

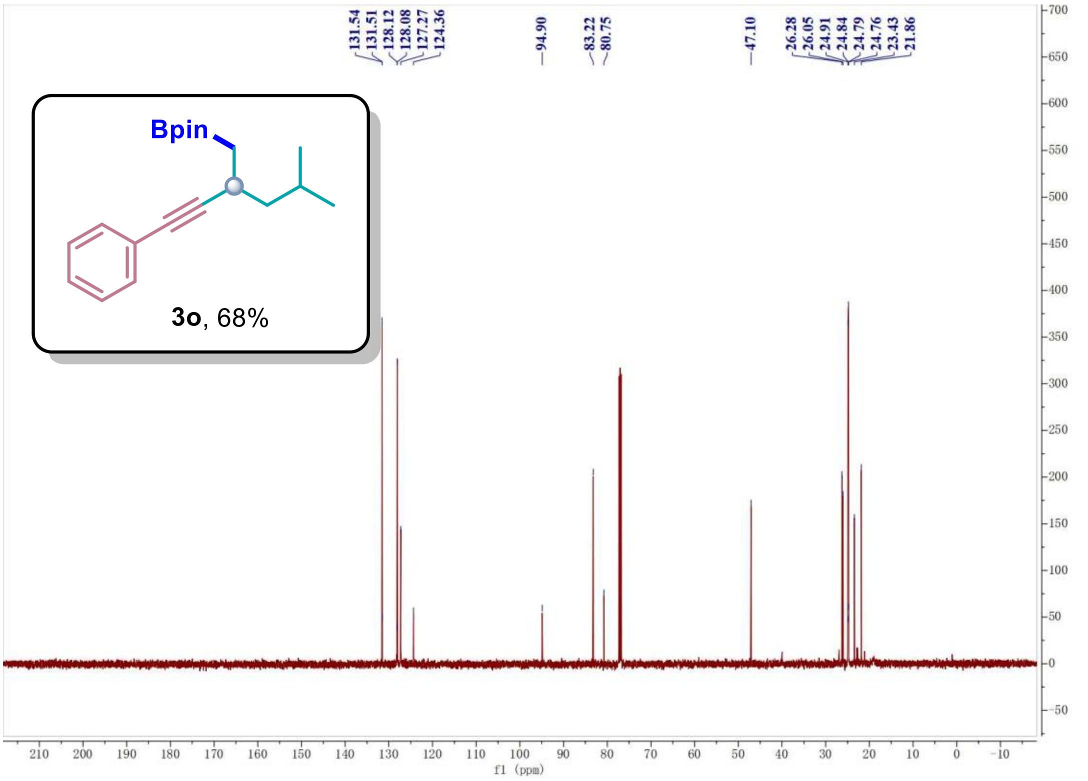

^{11}B NMR (160 MHz, Chloroform-*d*)

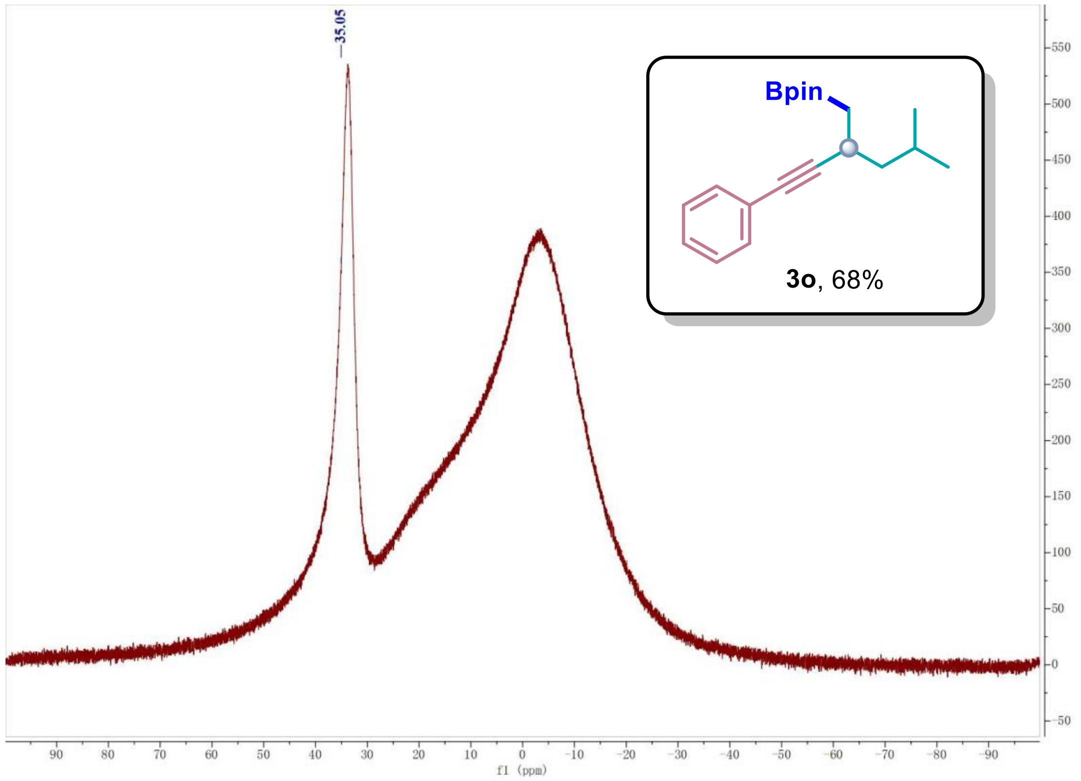

4,4,5,5-tetramethyl-2-(2-(1-(phenylethynyl)cyclobutyl)ethyl)-1,3,2-dioxaborolane (3p)

^{1}H NMR (500 MHz, Chloroform-*d*)

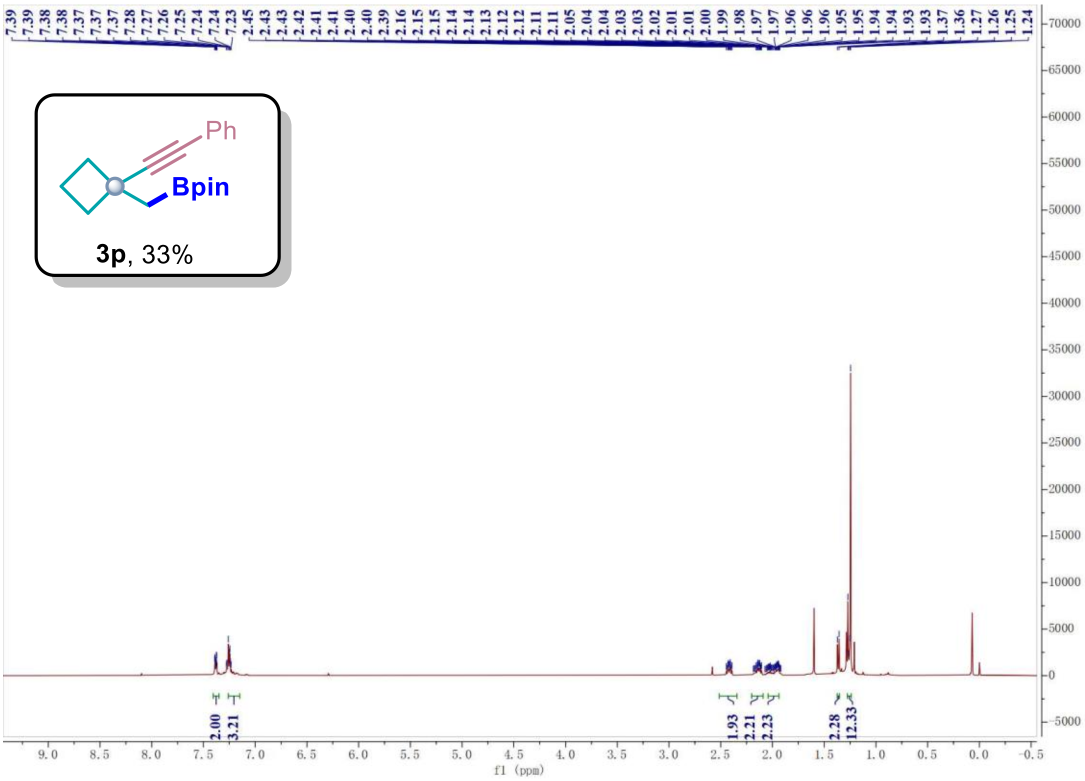

^{13}C NMR (126 MHz, Chloroform-*d*)

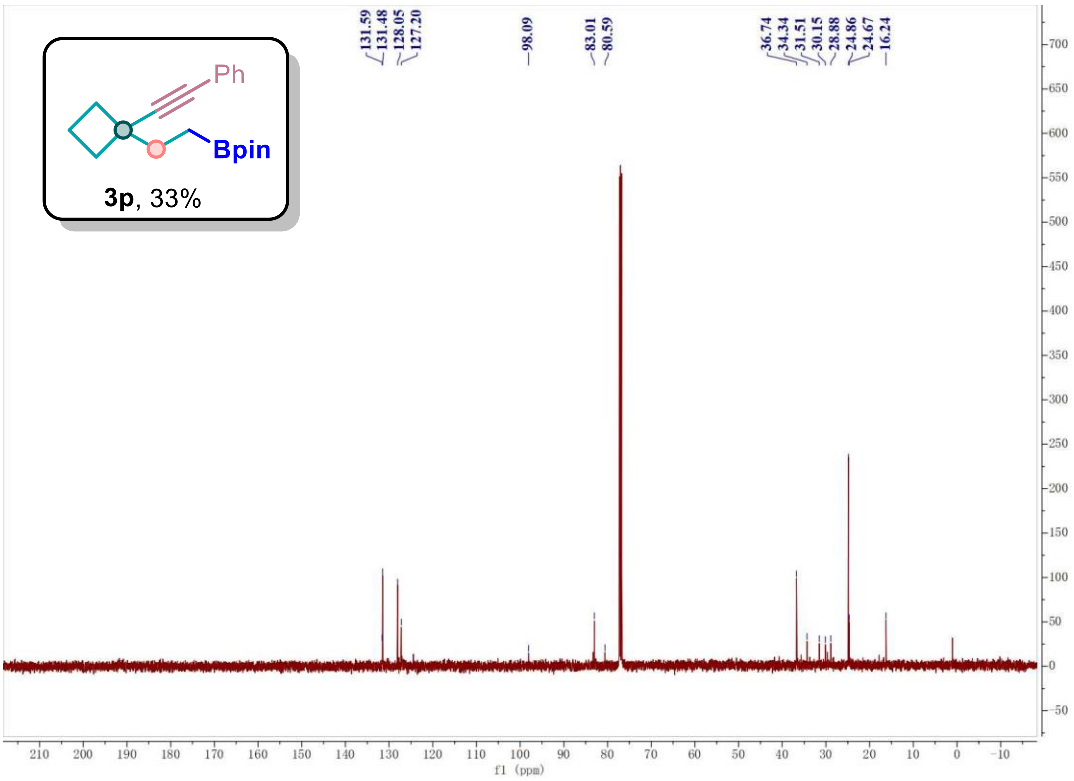

^{11}B NMR (160 MHz, Chloroform-*d*)

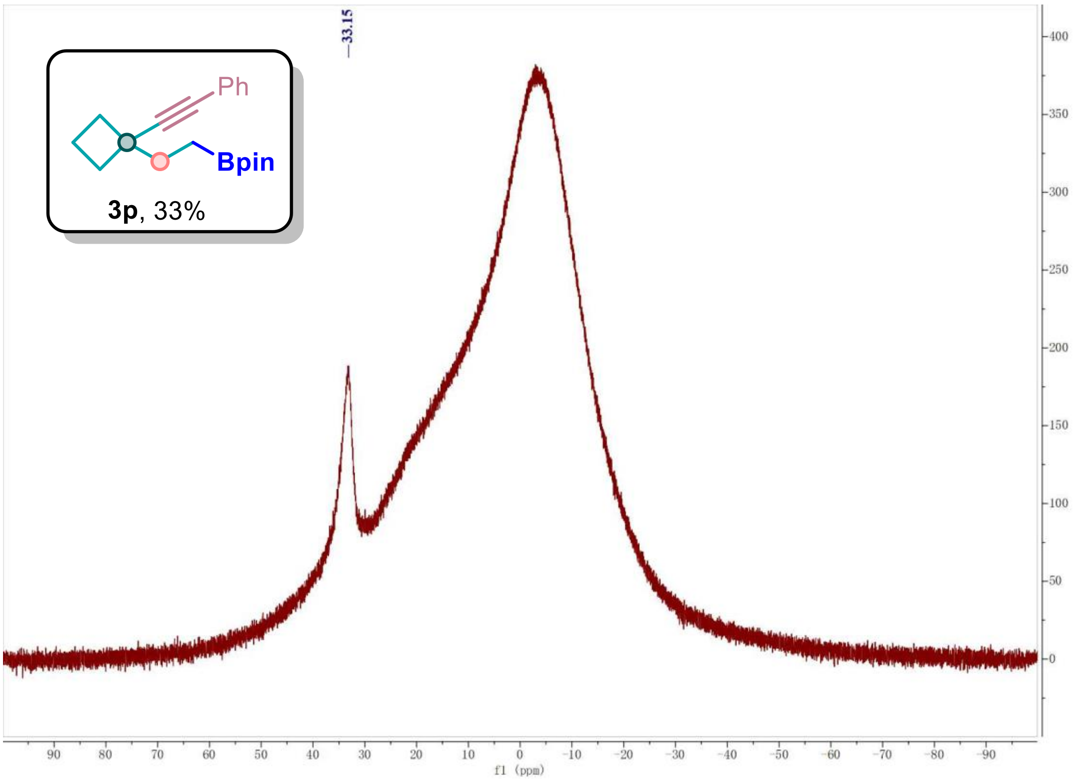

4,4,5,5-tetramethyl-2-(2-(phenylethynyl)tridecyl)-1,3,2-dioxaborolane (3q)

^{1}H NMR (500 MHz, Chloroform-*d*)

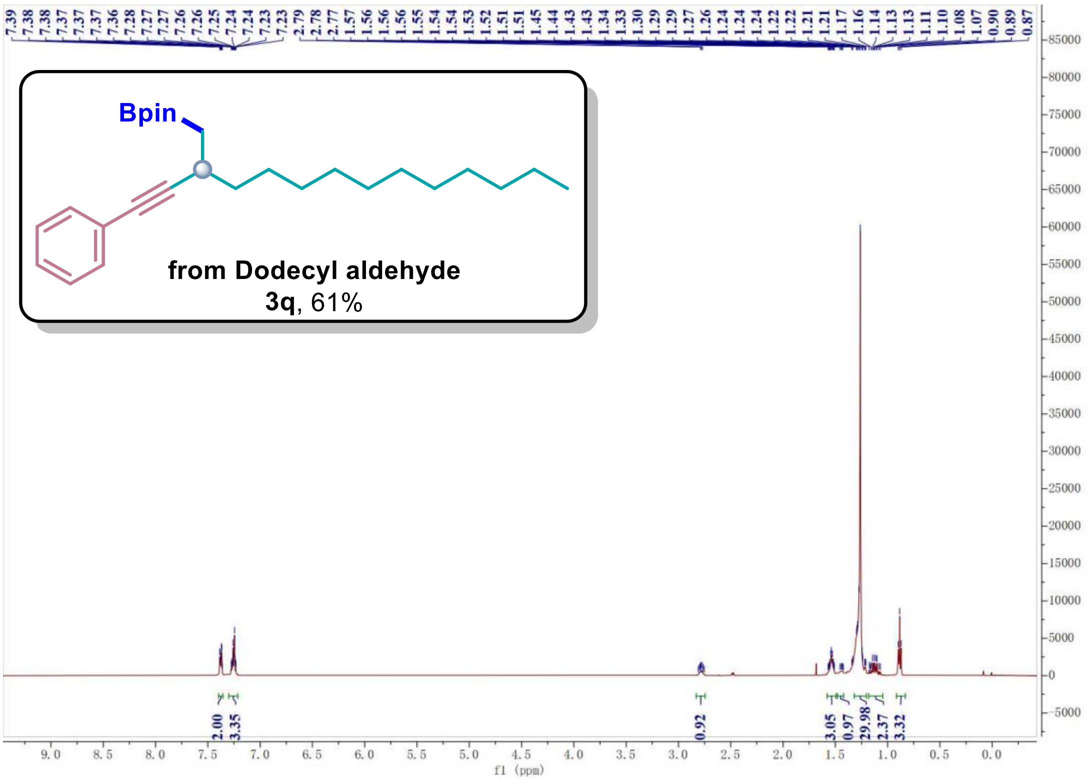

^{13}C NMR (126 MHz, Chloroform-*d*)

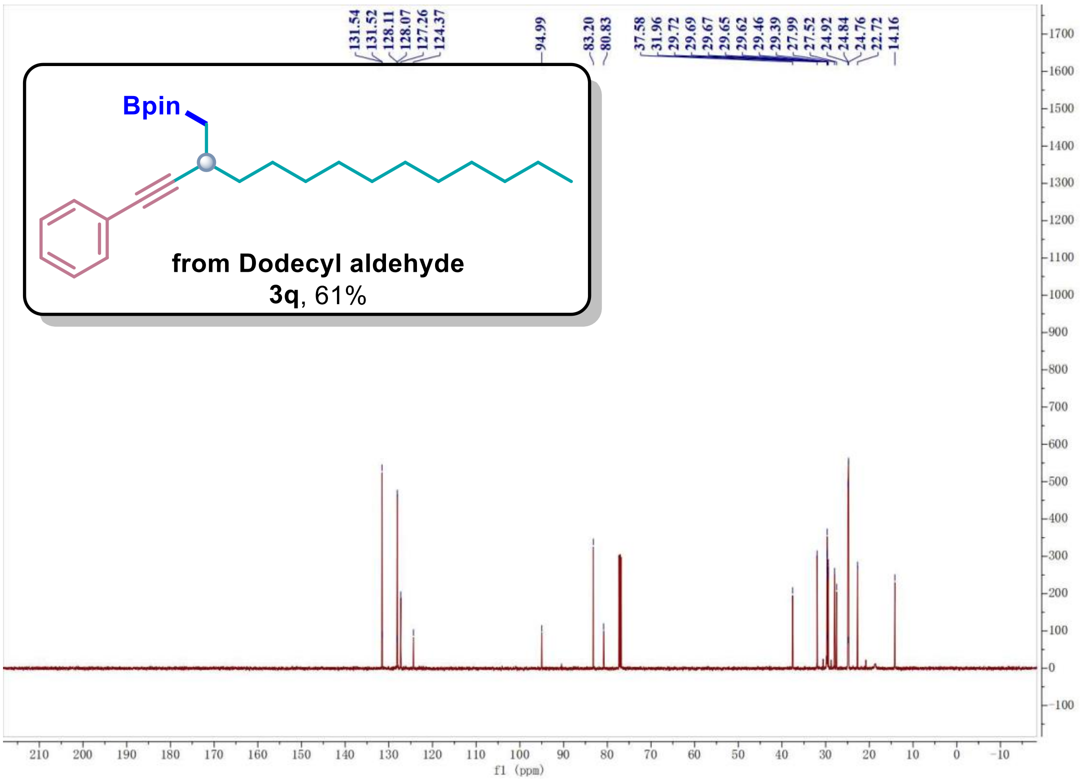

^{11}B NMR (160 MHz, Chloroform-*d*)

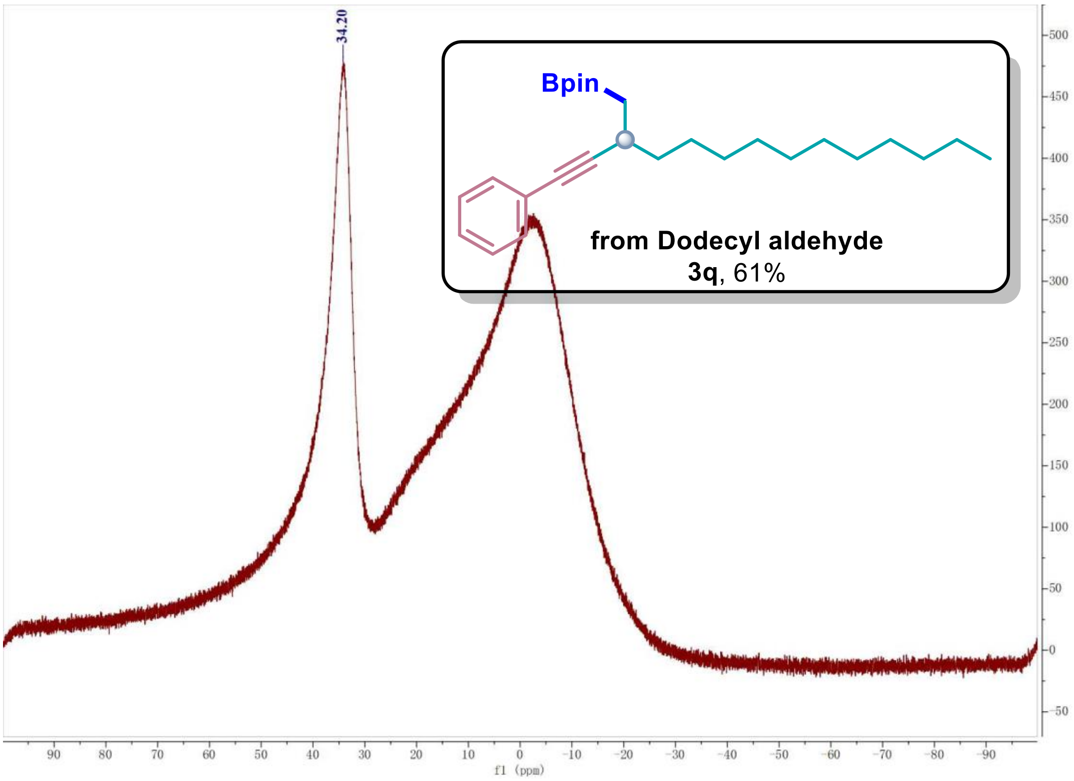

2-(2-(1-(4-isopropylphenyl)propan-2-yl)-4-phenylbut-3-yn-1-yl)-4,4,5,5-tetramethyl-1,3,2-dioxaborolane (3q)

^{1}H NMR (500 MHz, Chloroform-*d*)

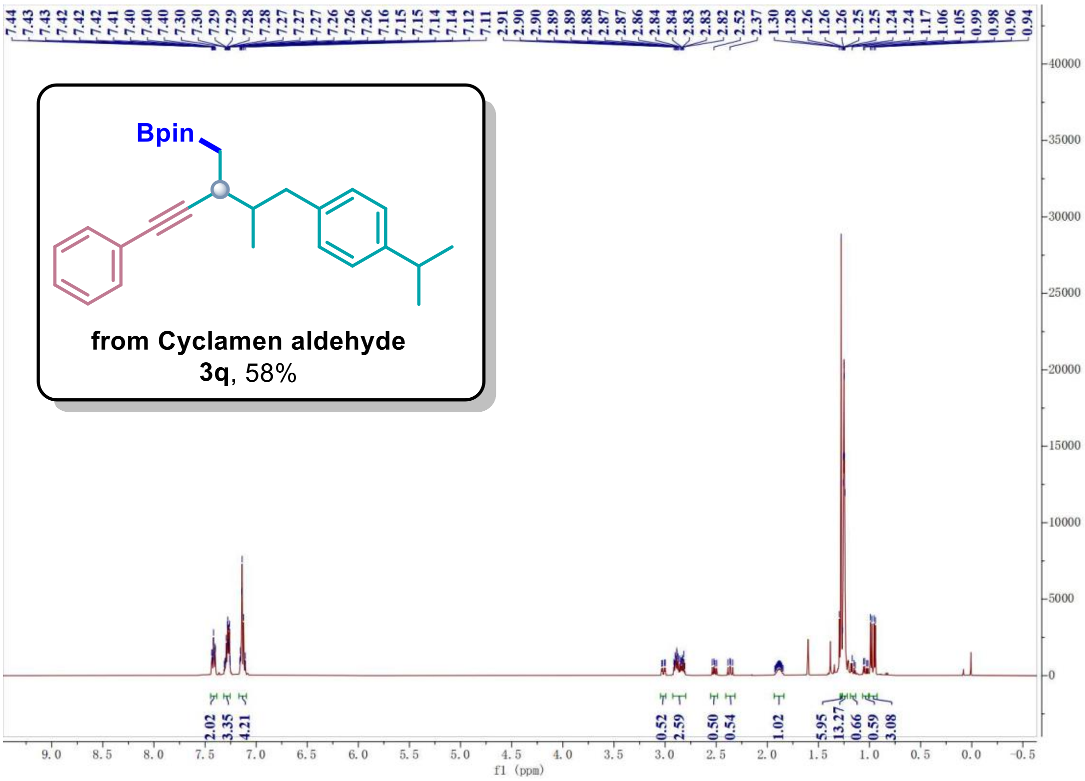

^{13}C NMR (126 MHz, Chloroform-*d*)

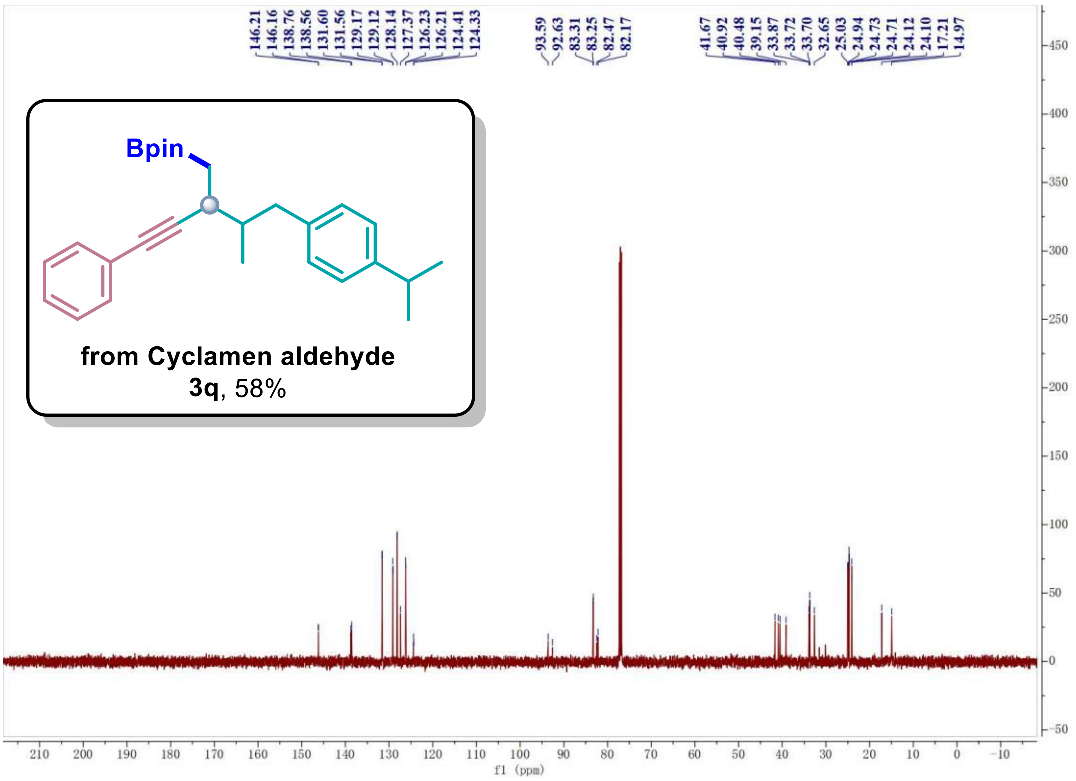

^{11}B NMR (160 MHz, Chloroform-*d*)

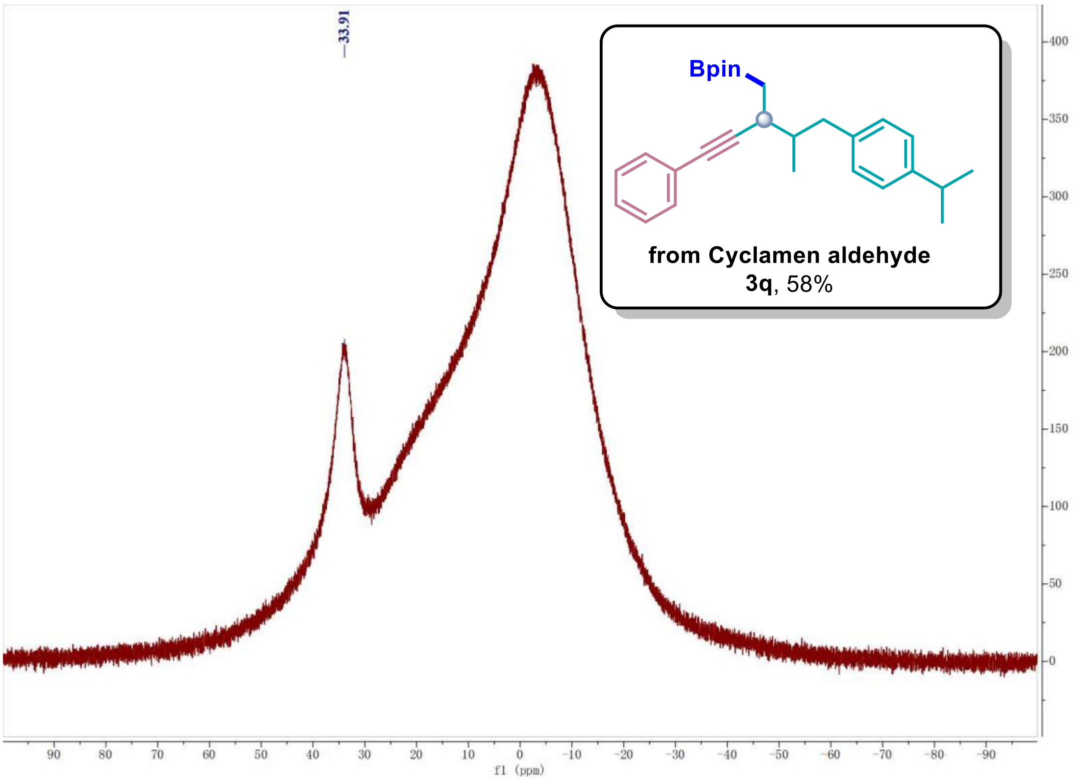

2-(2-(1-(4-isopropylphenyl)propan-2-yl)-4-phenylbut-3-yn-1-yl)-4,4,5,5-tetramethyl-1,3,2-dioxaborolane (3r)

^{1}H NMR (500 MHz, Chloroform-*d*)

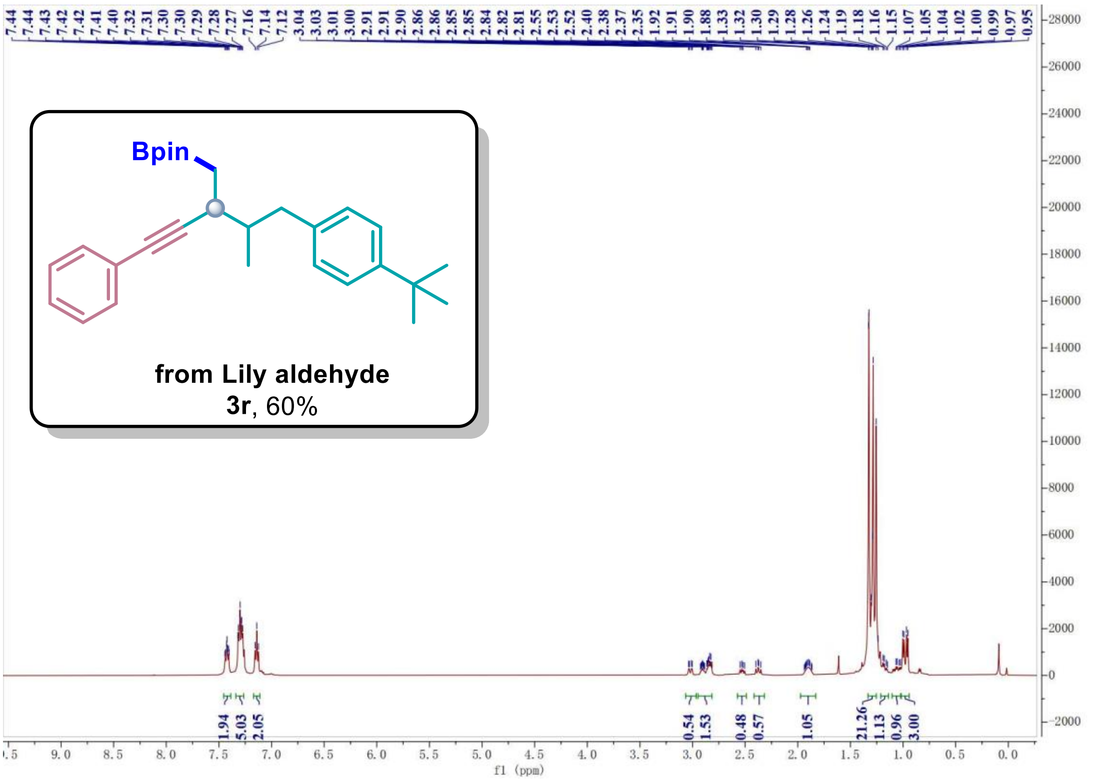

^{13}C NMR (126 MHz, Chloroform-*d*)

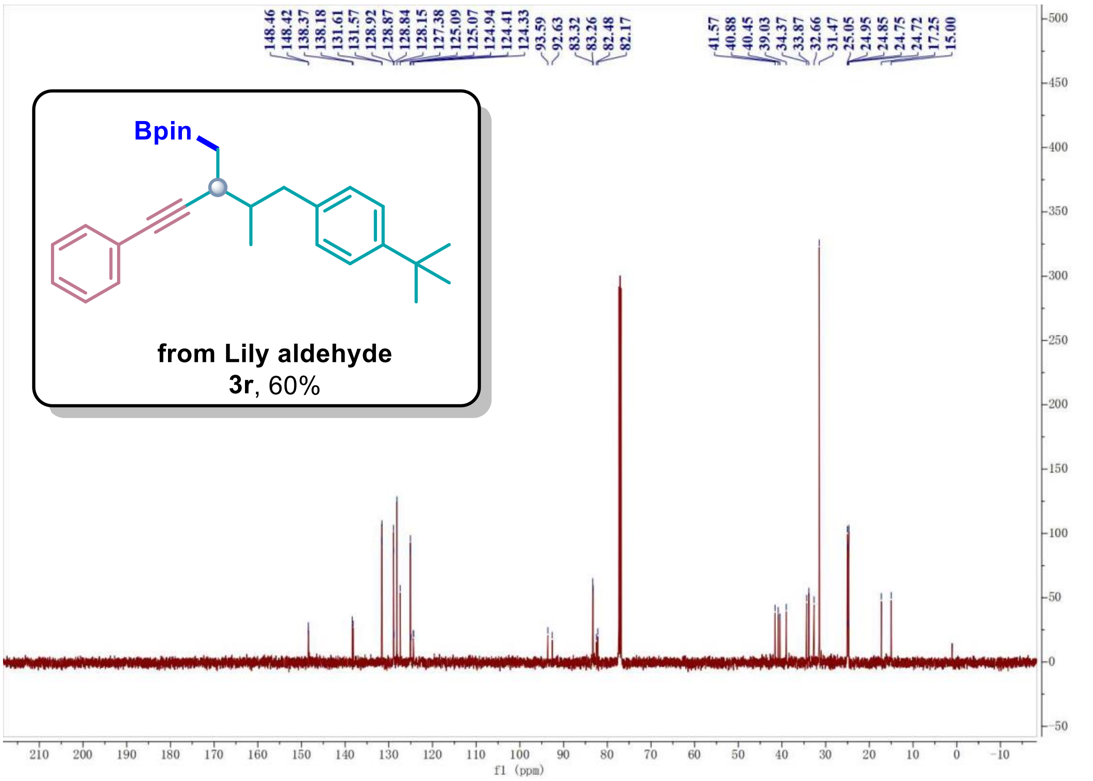

^{11}B NMR (160 MHz, Chloroform-*d*)

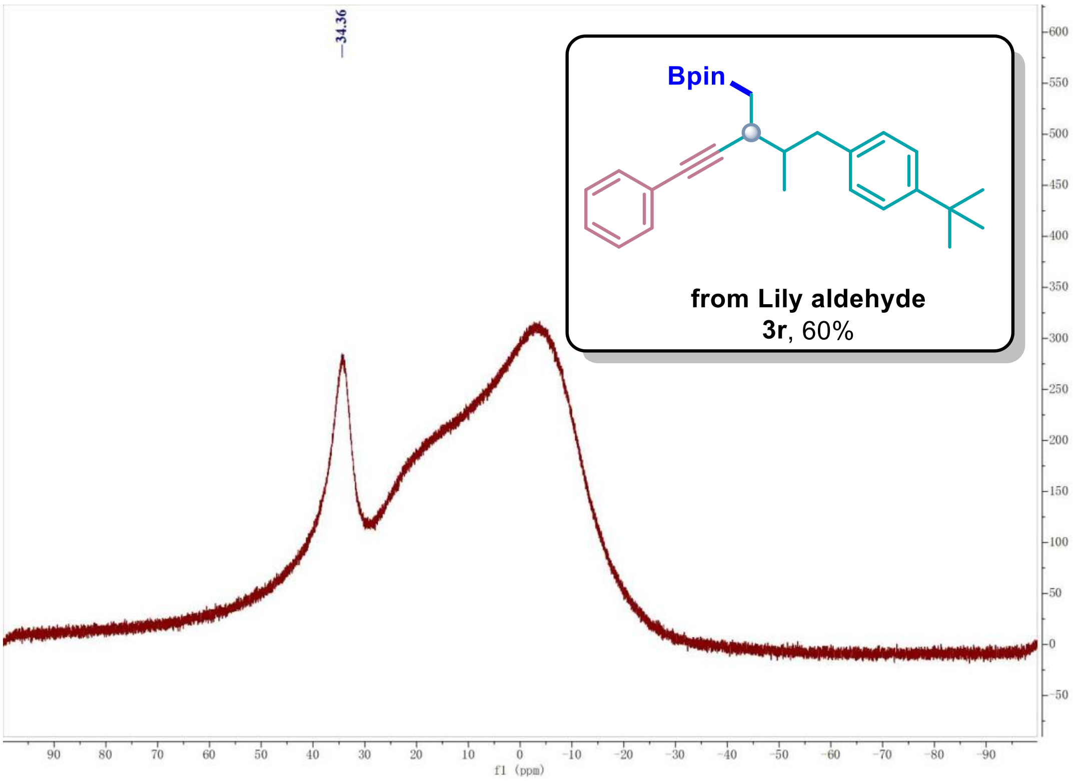

2-(4,4-dimethyl-2-(phenylethynyl)pentyl)-5,5-dimethyl-1,3,2-dioxaborinane (3t)

^{1}H NMR (500 MHz, Chloroform-*d*)

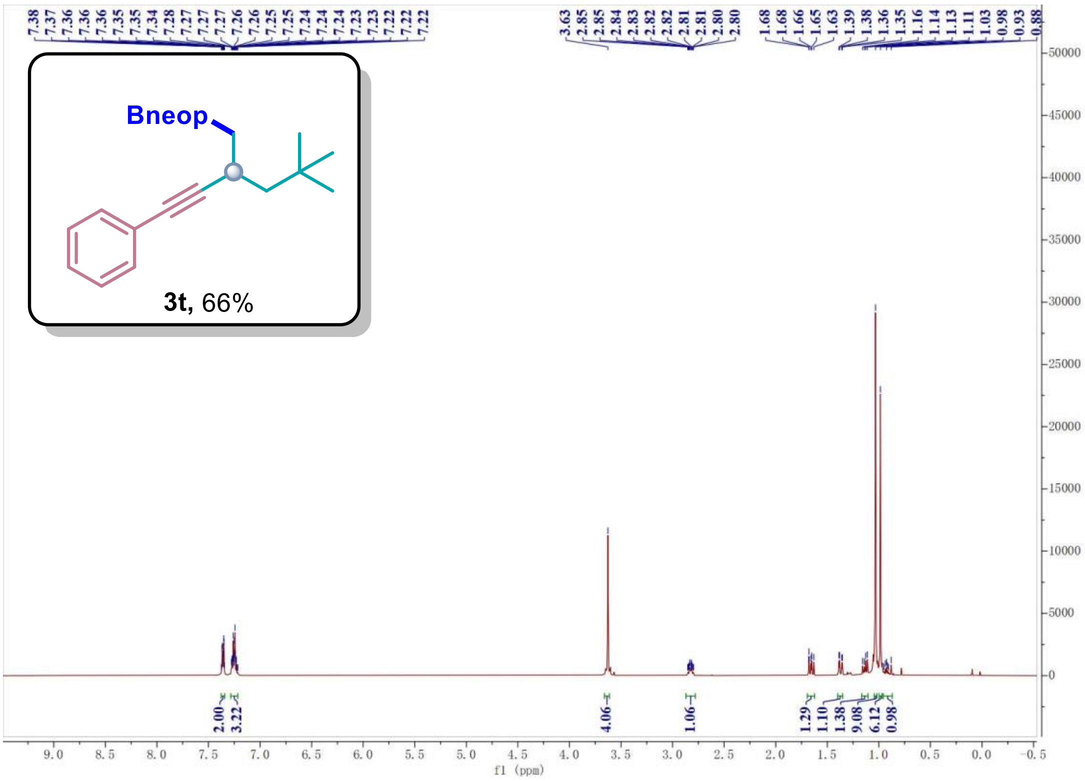

^{13}C NMR (126 MHz, Chloroform-*d*)

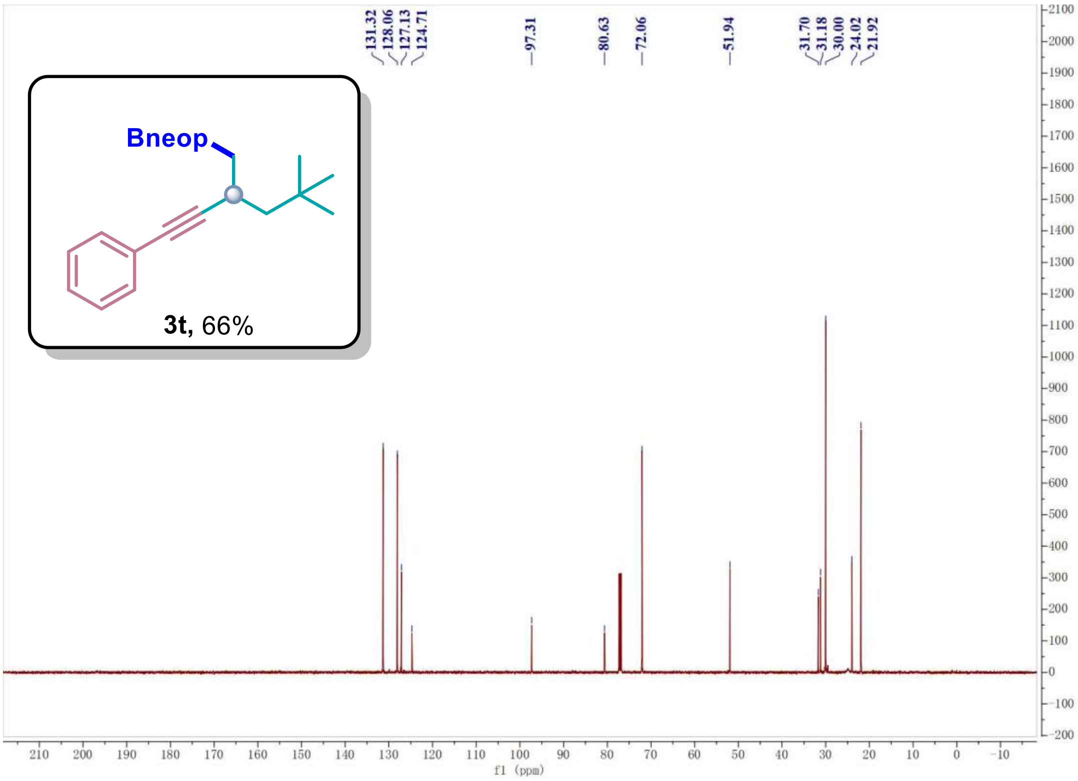

^{11}B NMR (160 MHz, Chloroform-*d*)

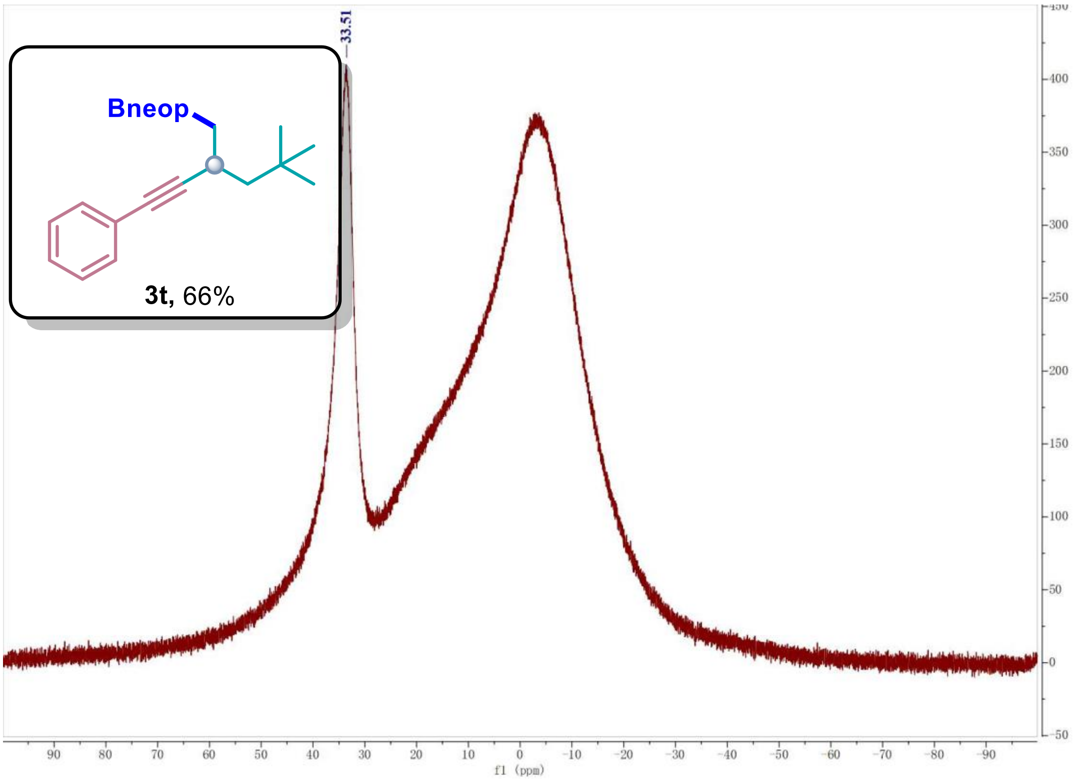

(E)-2-(2-(4-methoxystyryl)-4,4-dimethylpentyl)-4,4,5,5-tetramethyl-1,3,2-dioxaborolane (3u)

^{1}H NMR (500 MHz, Chloroform-*d*)

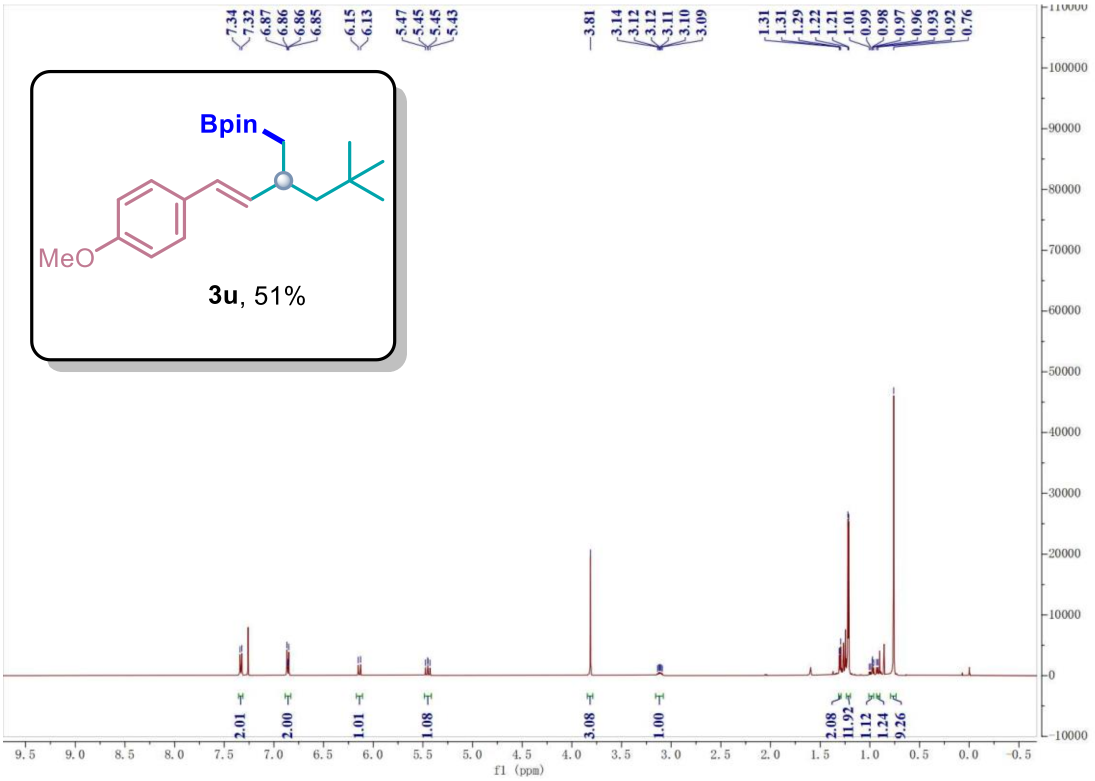

^{13}C NMR (126 MHz, Chloroform-*d*)

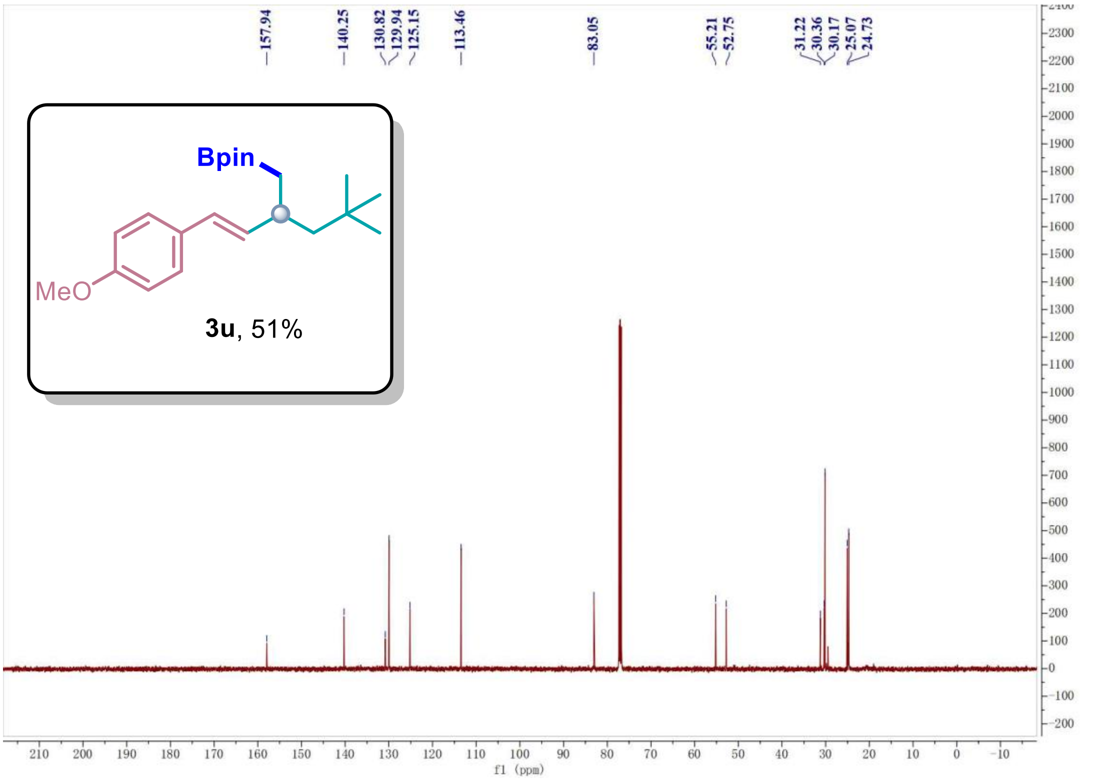

^{11}B NMR (160 MHz, Chloroform-*d*)

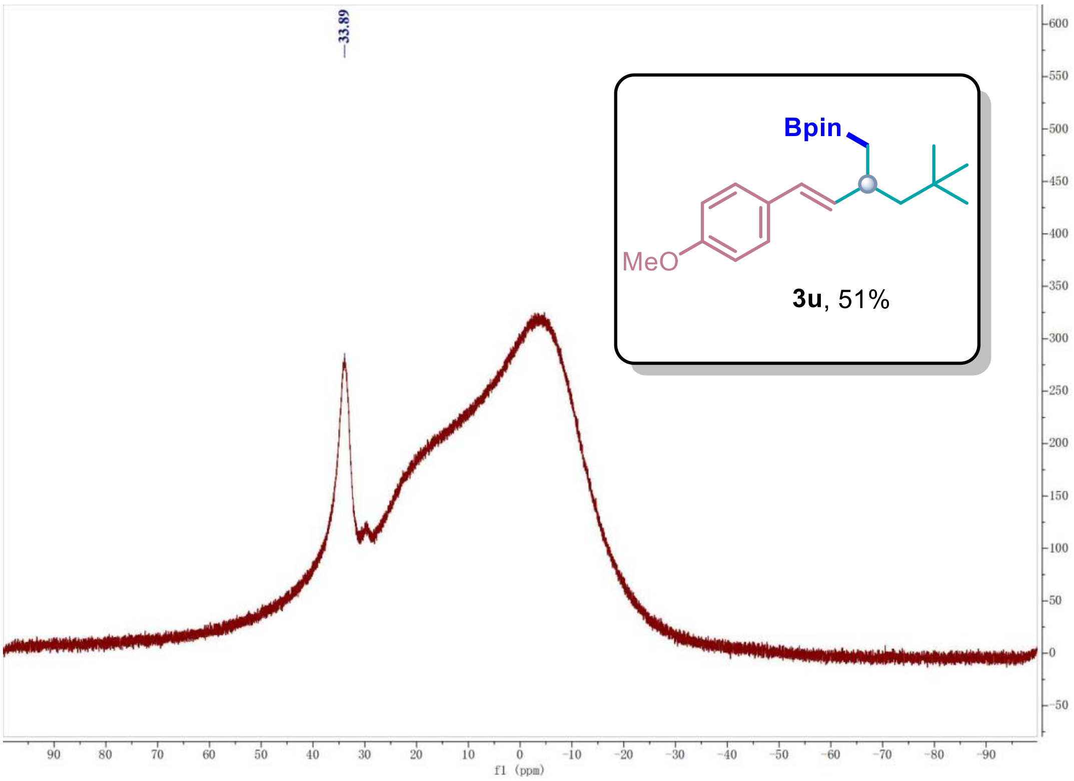

(E)-2-(2-(4-methoxystyryl)-4-methylpentyl)-4,4,5,5-tetramethyl-1,3,2-dioxaborolane (3v)

^{1}H NMR (500 MHz, Chloroform-*d*)

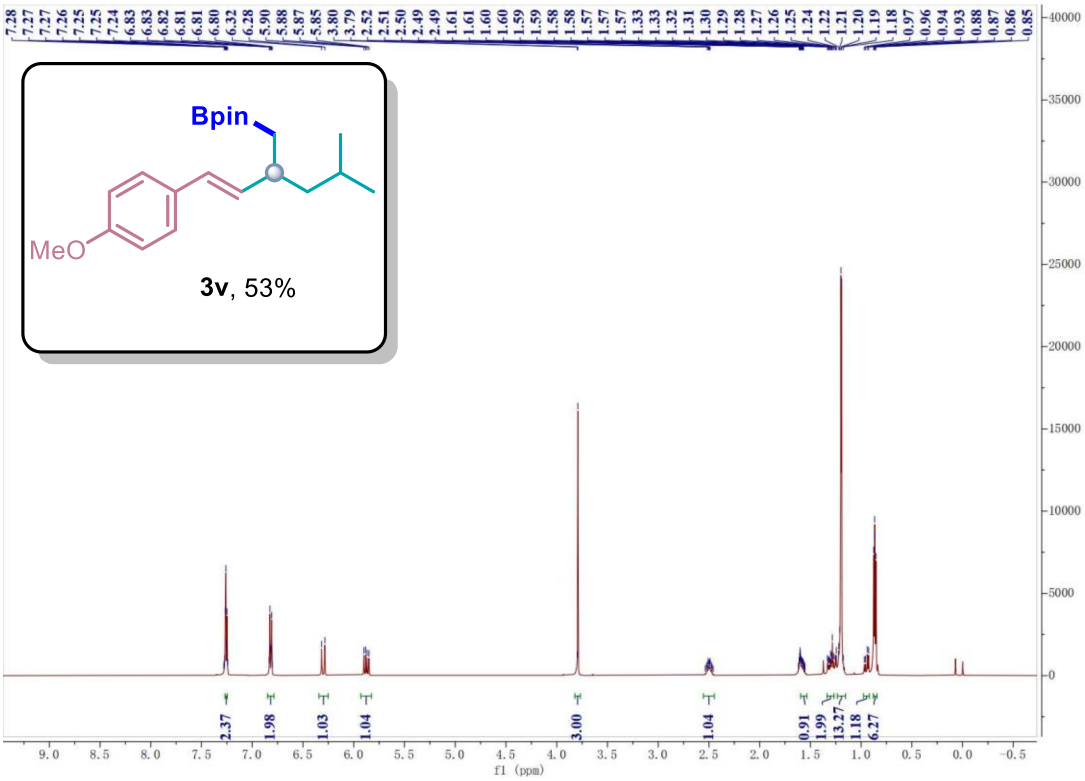

^{13}C NMR (126 MHz, Chloroform-*d*)

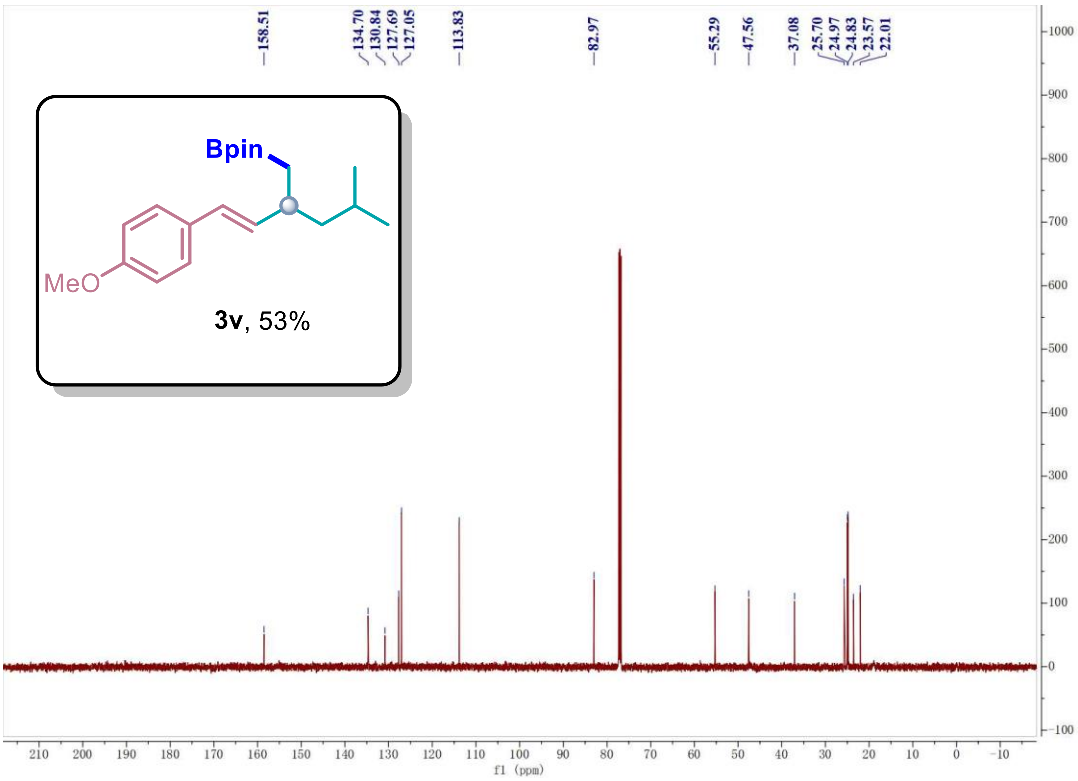

^{11}B NMR (160 MHz, Chloroform-*d*)

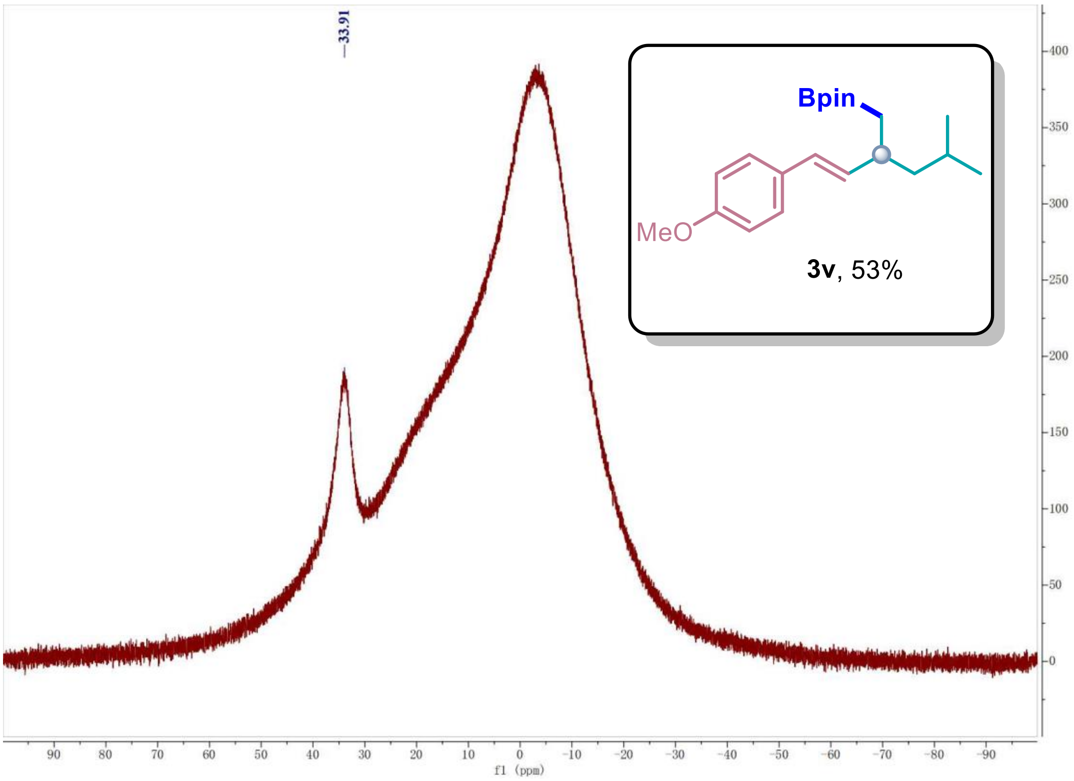

(E)-2-(2-cyclopentyl-4-(4-methoxyphenyl)but-3-en-1-yl)-4,4,5,5-tetramethyl-1,3,2-dioxaborolane (3w)

^{1}H NMR (500 MHz, Chloroform-*d*)

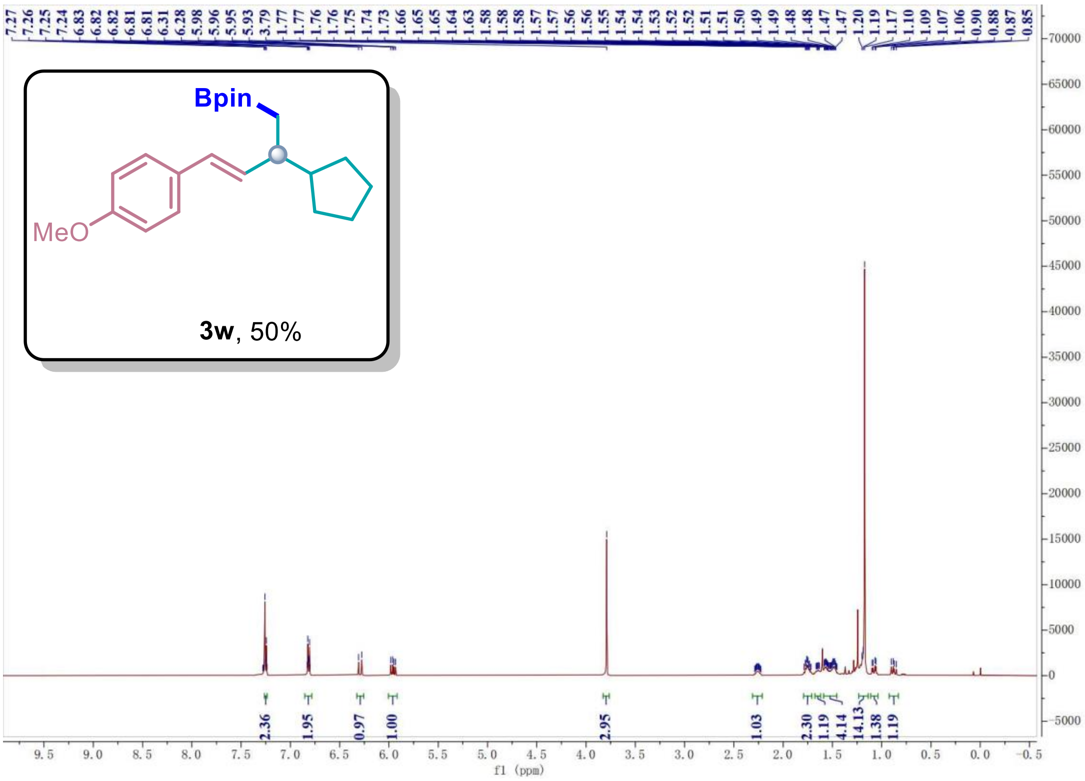

^{13}C NMR (126 MHz, Chloroform-*d*)

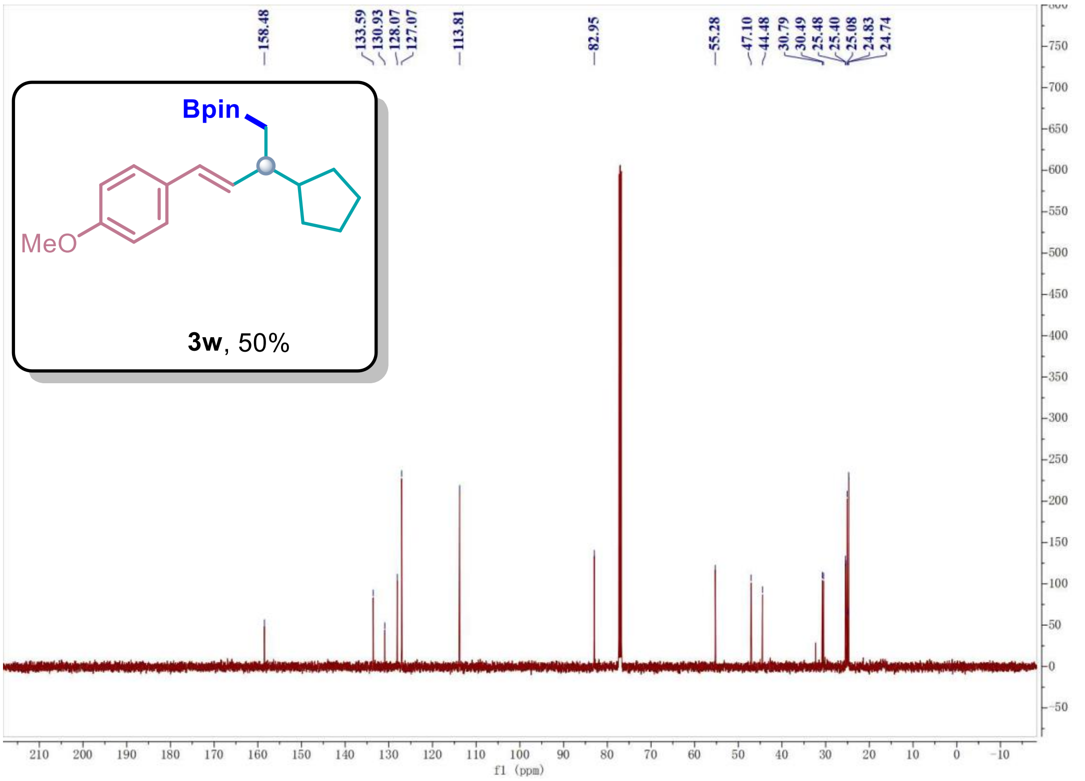

^{11}B NMR (160 MHz, Chloroform-*d*)

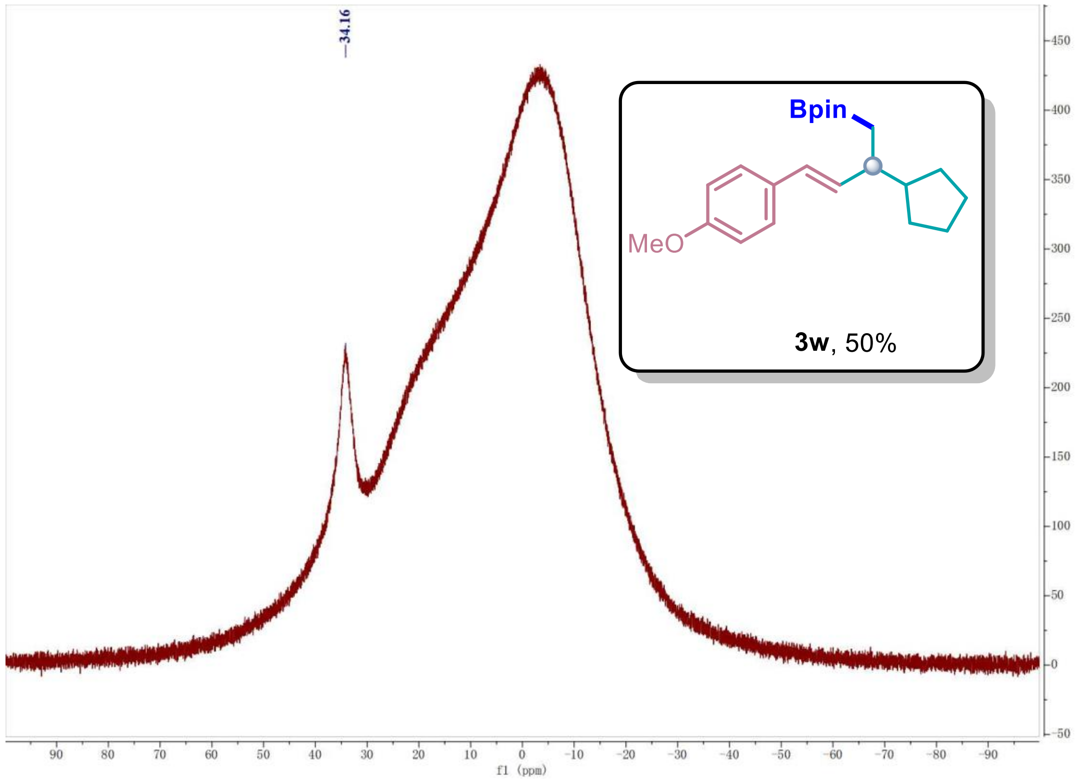

(E)-2-(3-ethyl-2-(4-methoxystyryl)pentyl)-4,4,5,5-tetramethyl-1,3,2-dioxaborolane (3x)

^{1}H NMR (500 MHz, Chloroform-*d*)

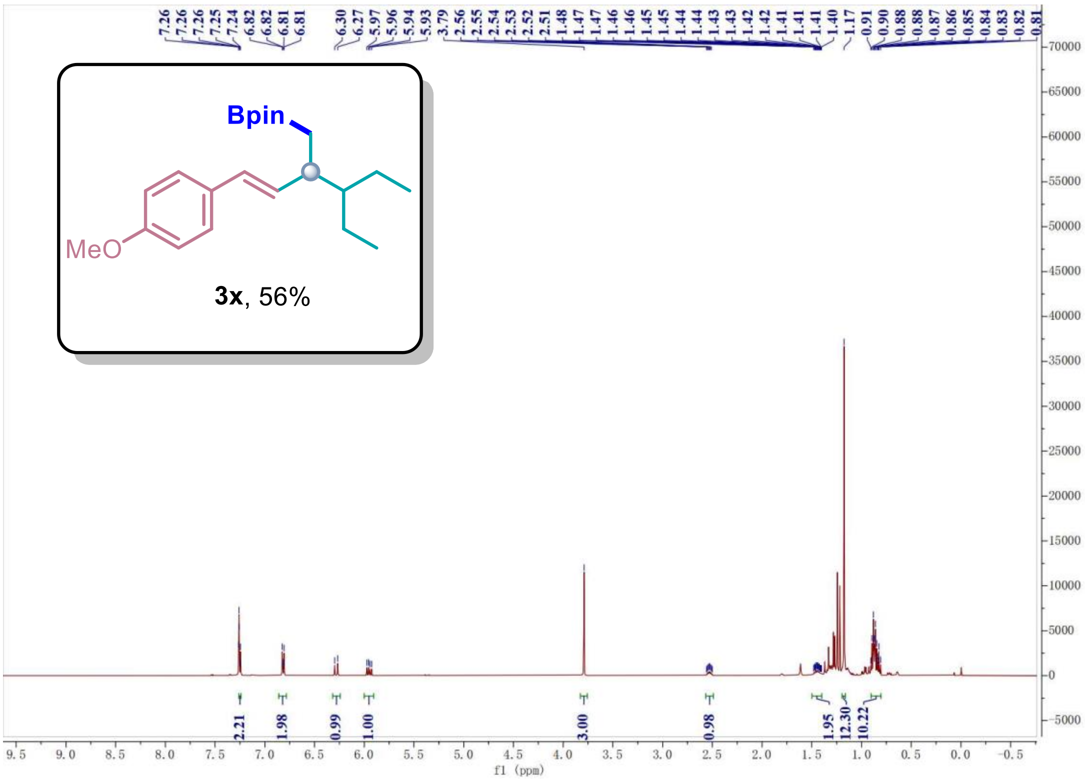

^{13}C NMR (126 MHz, Chloroform-*d*)

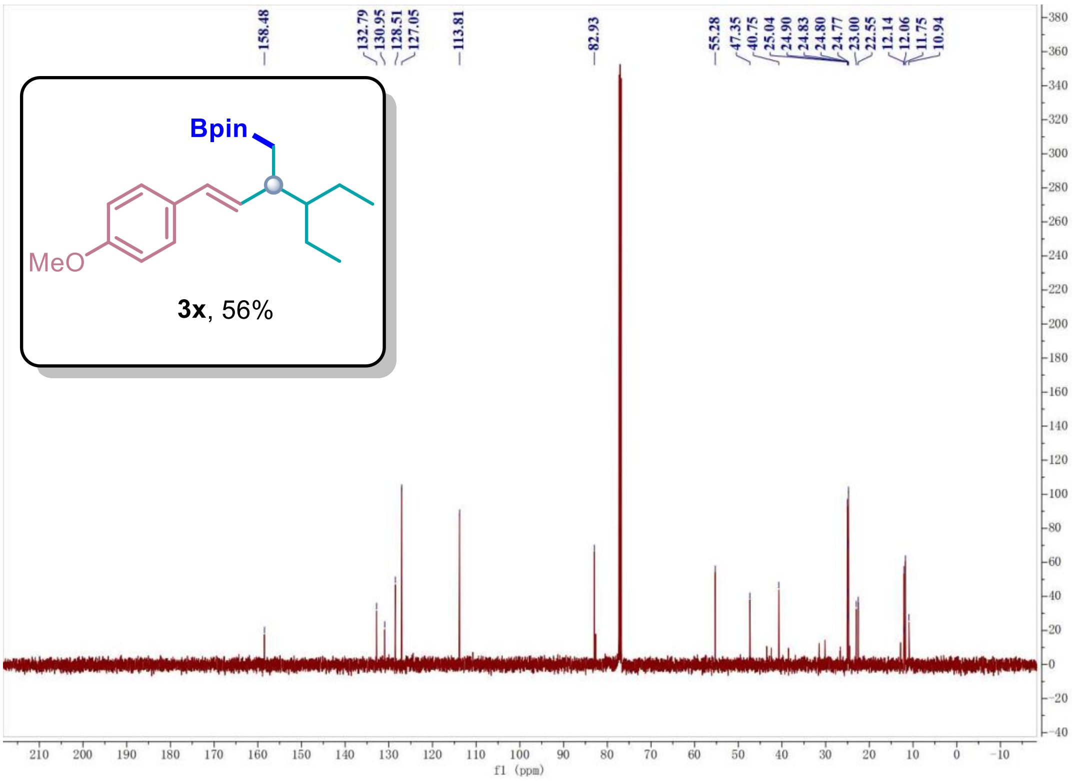

^{11}B NMR (160 MHz, Chloroform-*d*)

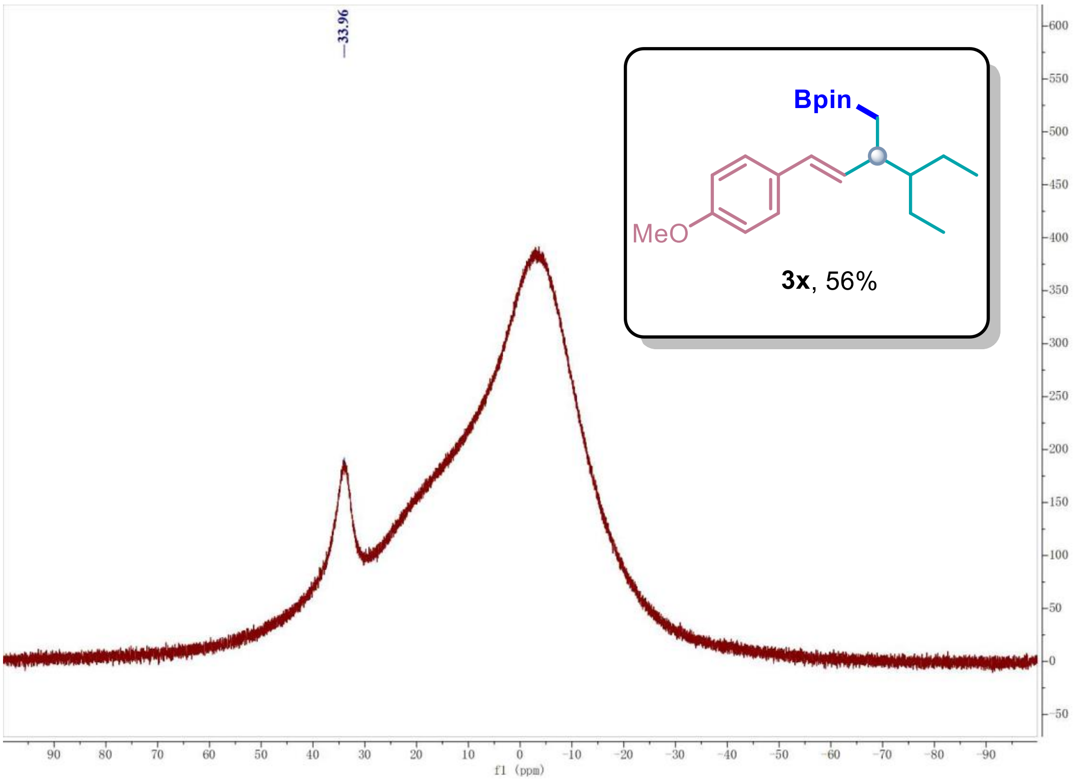

(E)-2-(4-(4-methoxyphenyl)-2-phenethylbut-3-en-1-yl)-4,4,5,5-tetramethyl-1,3,2-dioxaborolane (3y)

^{1}H NMR (500 MHz, Chloroform-*d*)

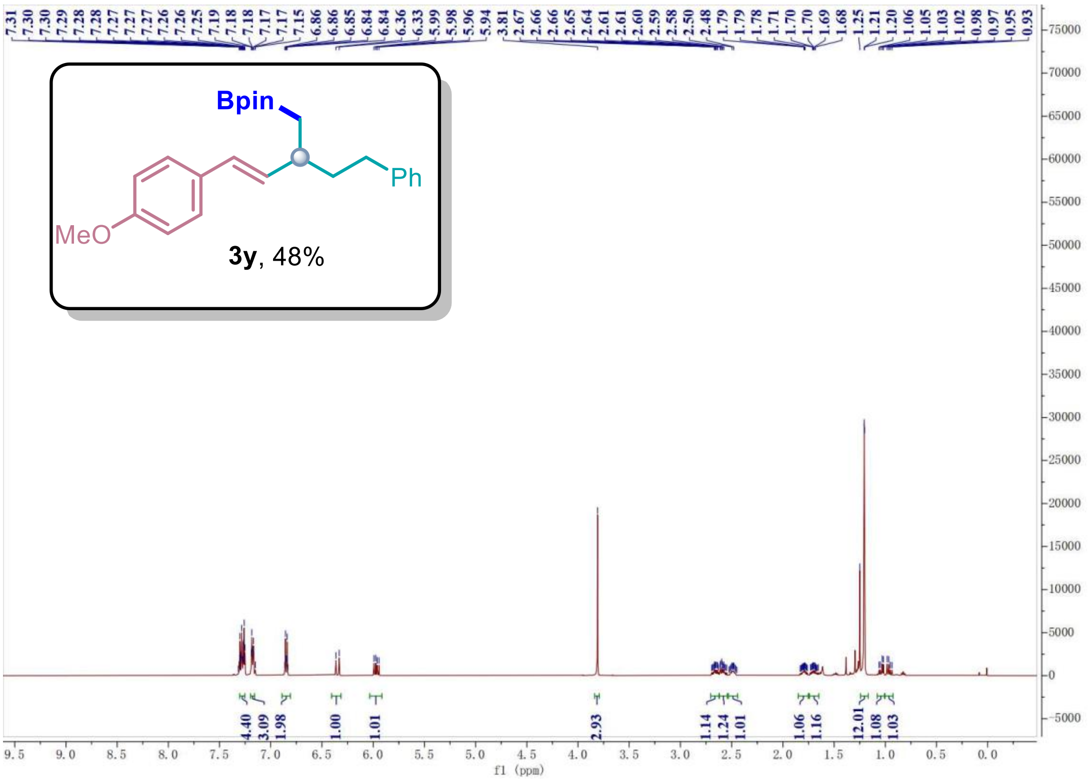

^{13}C NMR (126 MHz, Chloroform-*d*)

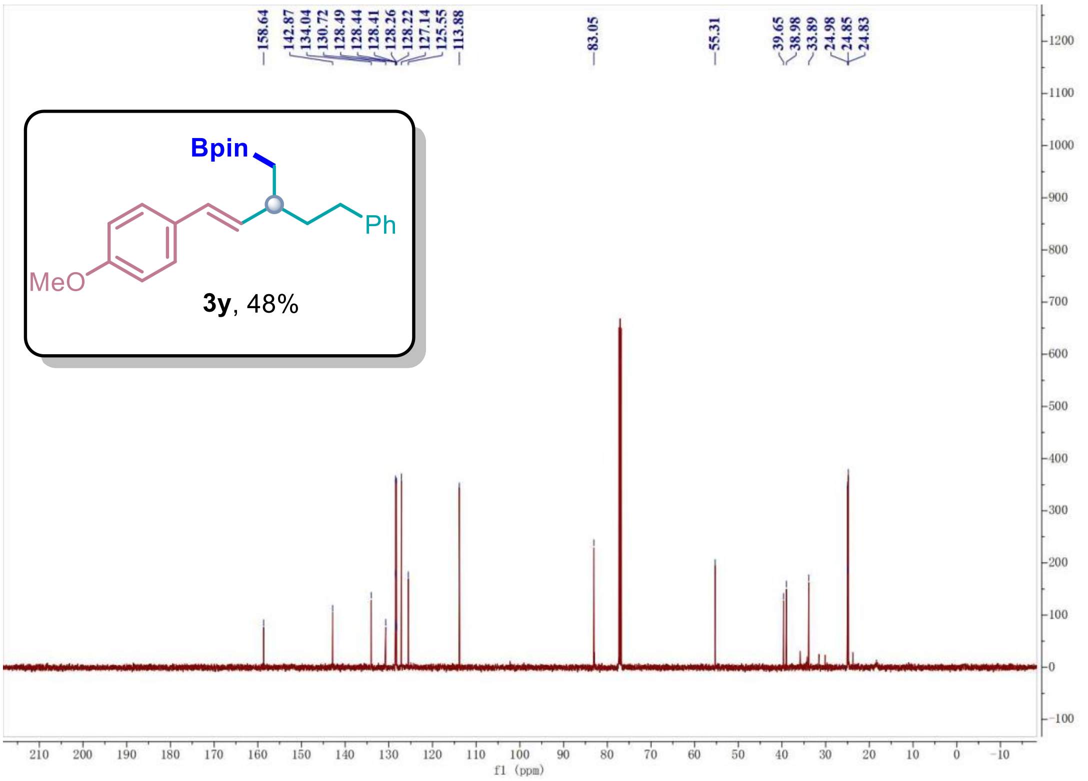

^{11}B NMR (160 MHz, Chloroform-*d*)

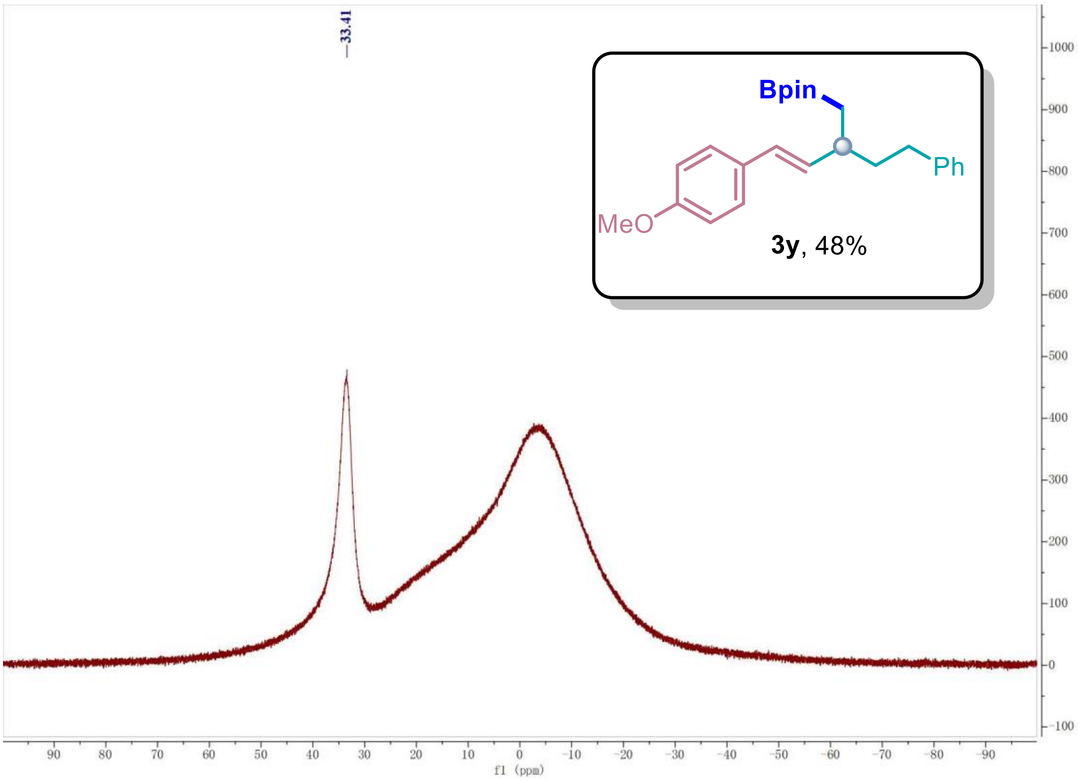

(E)-2-(2-cyclohexyl-4-(4-methoxyphenyl)but-3-en-1-yl)-4,4,5,5-tetramethyl-1,3,2-dioxaborolane (3z)

^{1}H NMR (500 MHz, Chloroform-*d*)

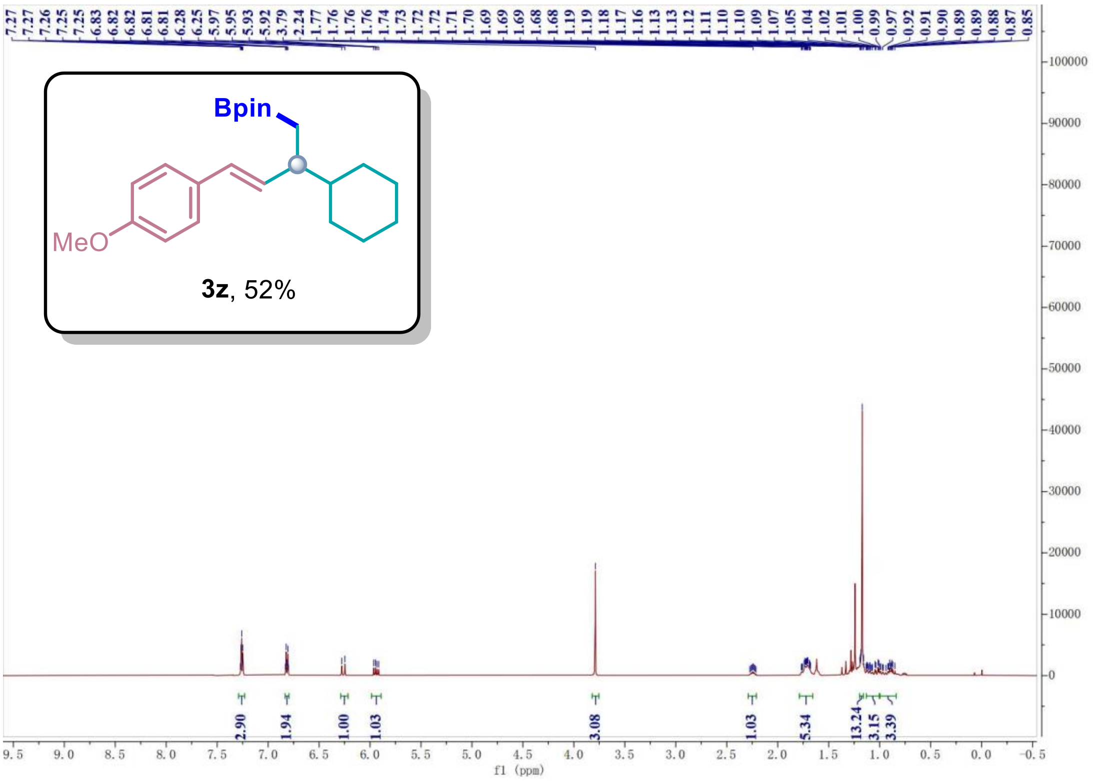

^{13}C NMR (126 MHz, Chloroform-*d*)

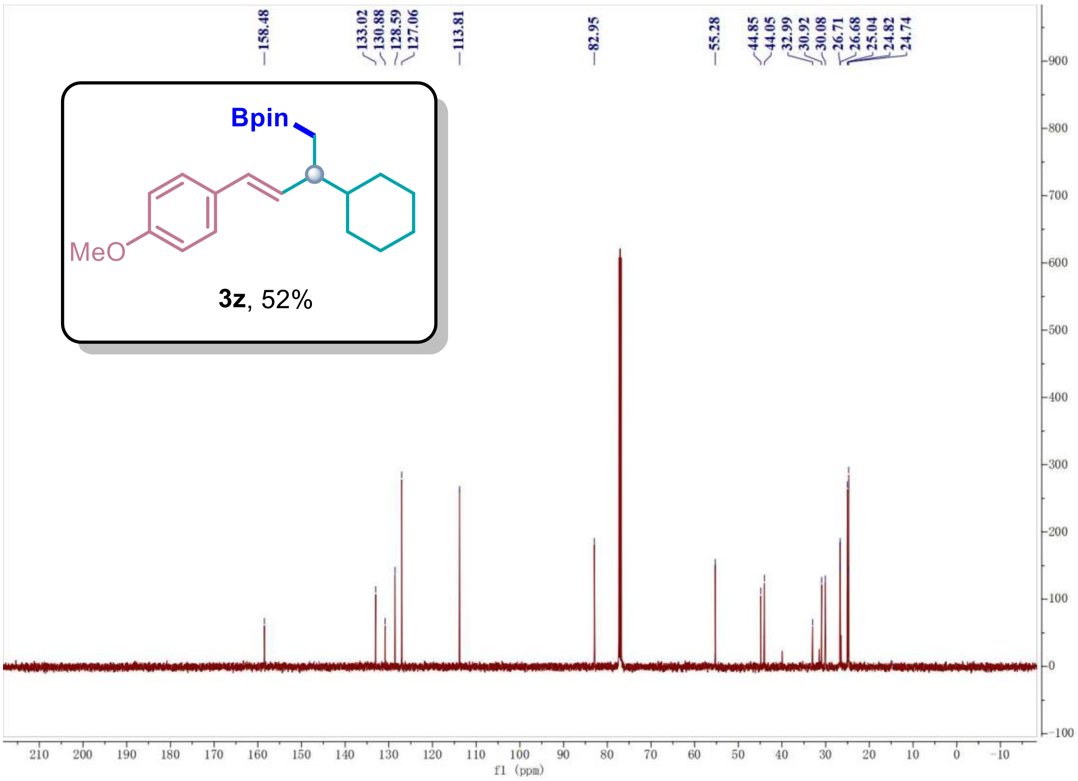

^{11}B NMR (160 MHz, Chloroform-*d*)

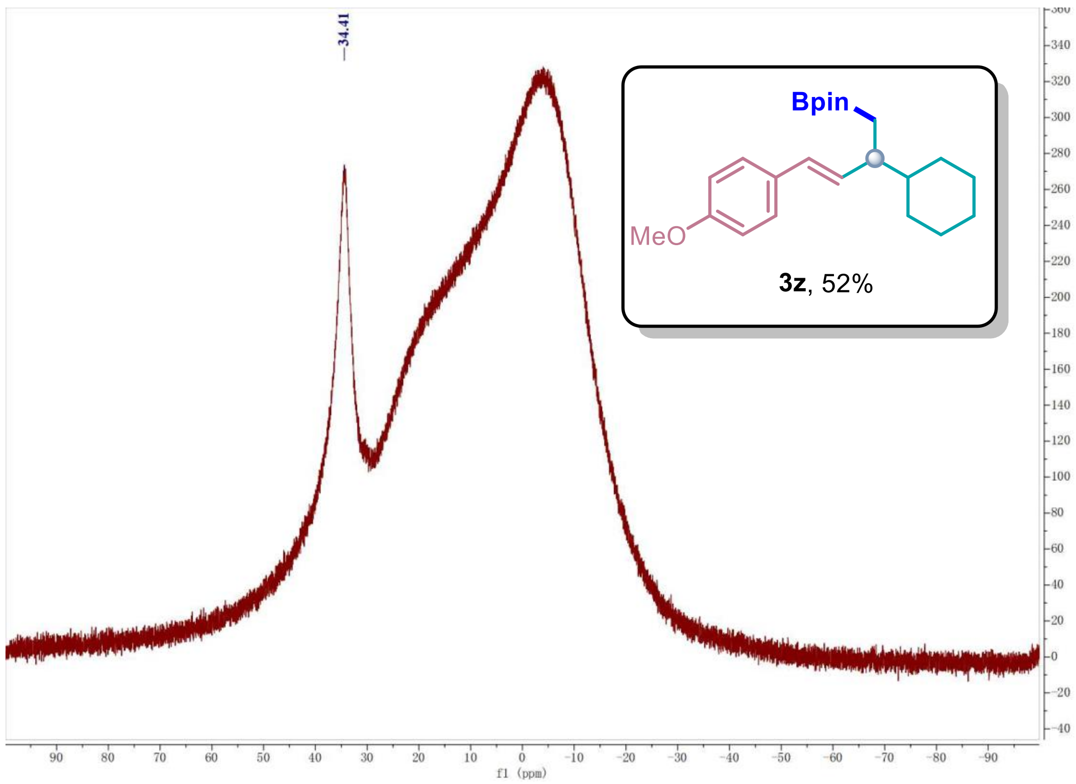

ethyl 6,6-dimethyl-2-methylene-4-((4,4,5,5-tetramethyl-1,3,2-dioxaborolan-2-yl)methyl)heptanoate (5a)

^{1}H NMR (500 MHz, Chloroform-*d*)

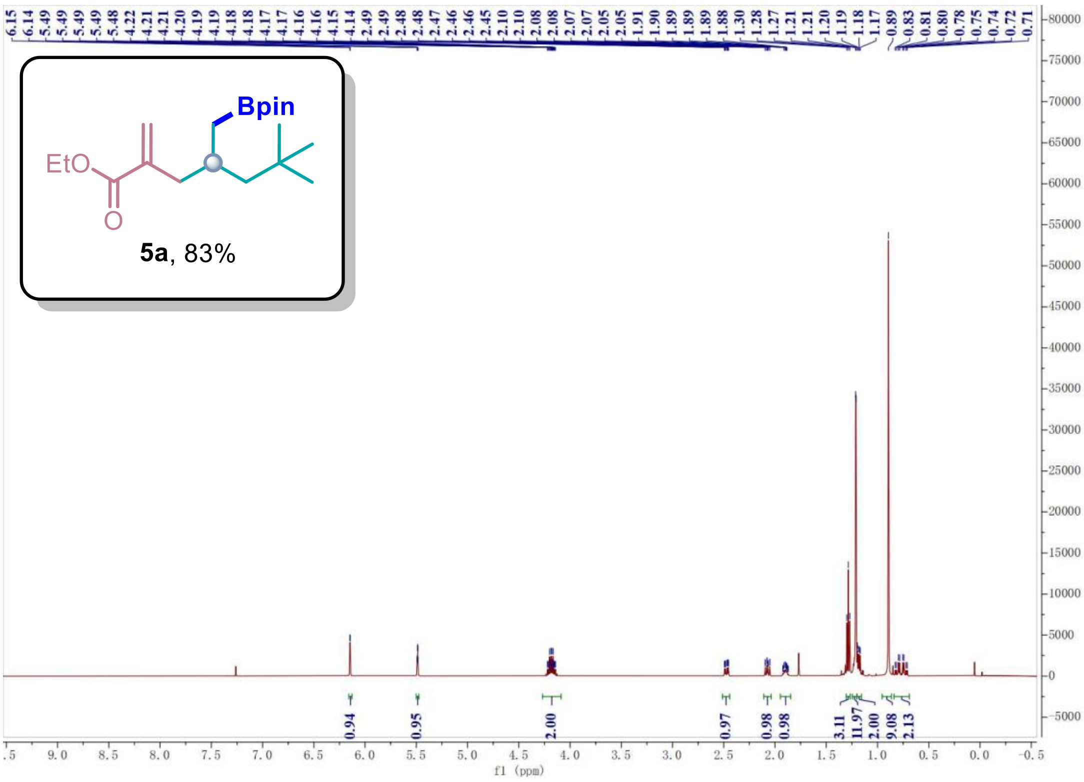

^{13}C NMR (126 MHz, Chloroform-*d*)

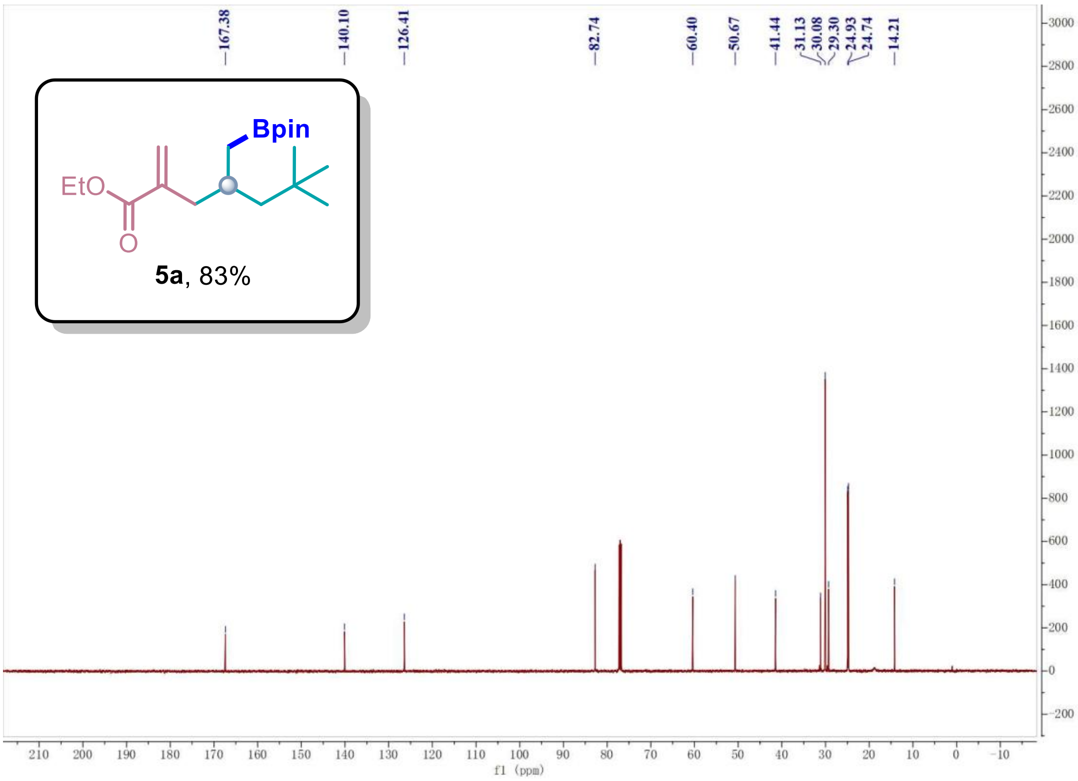

^{11}B NMR (160 MHz, Chloroform-*d*)

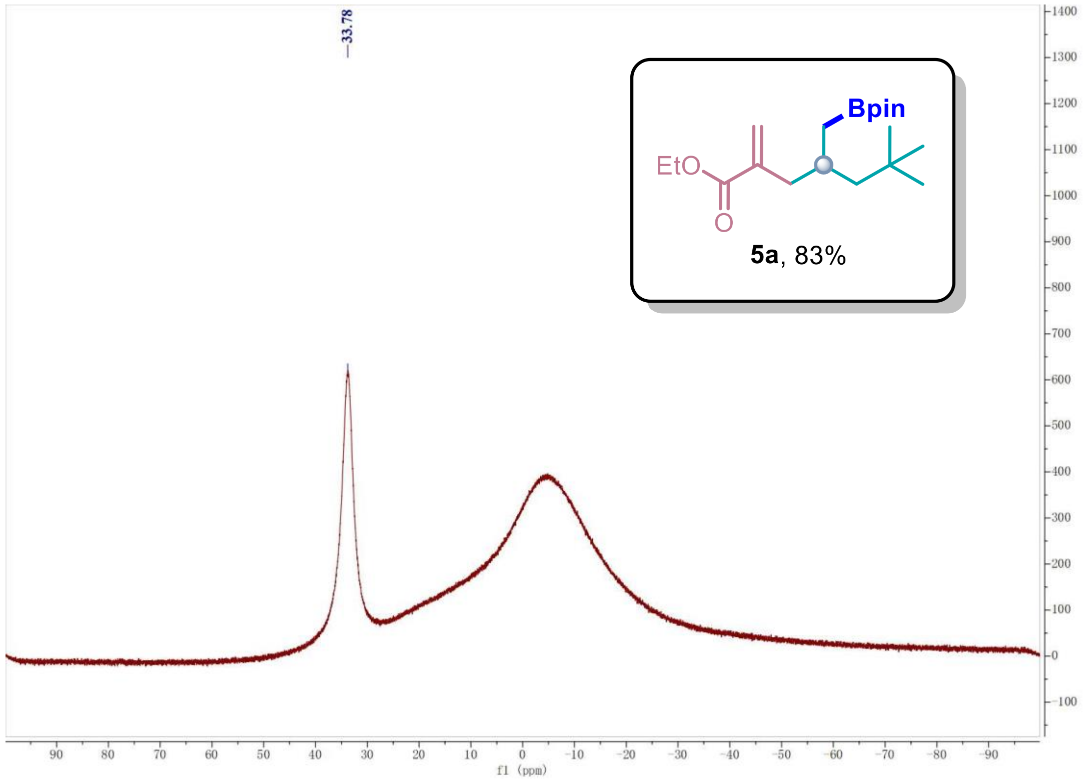

4,4,5,5-tetramethyl-2-(2-neopentyl-4-phenylpent-4-en-1-yl)-1,3,2-dioxaborolane (5b)

^{1}H NMR (500 MHz, Chloroform-*d*)

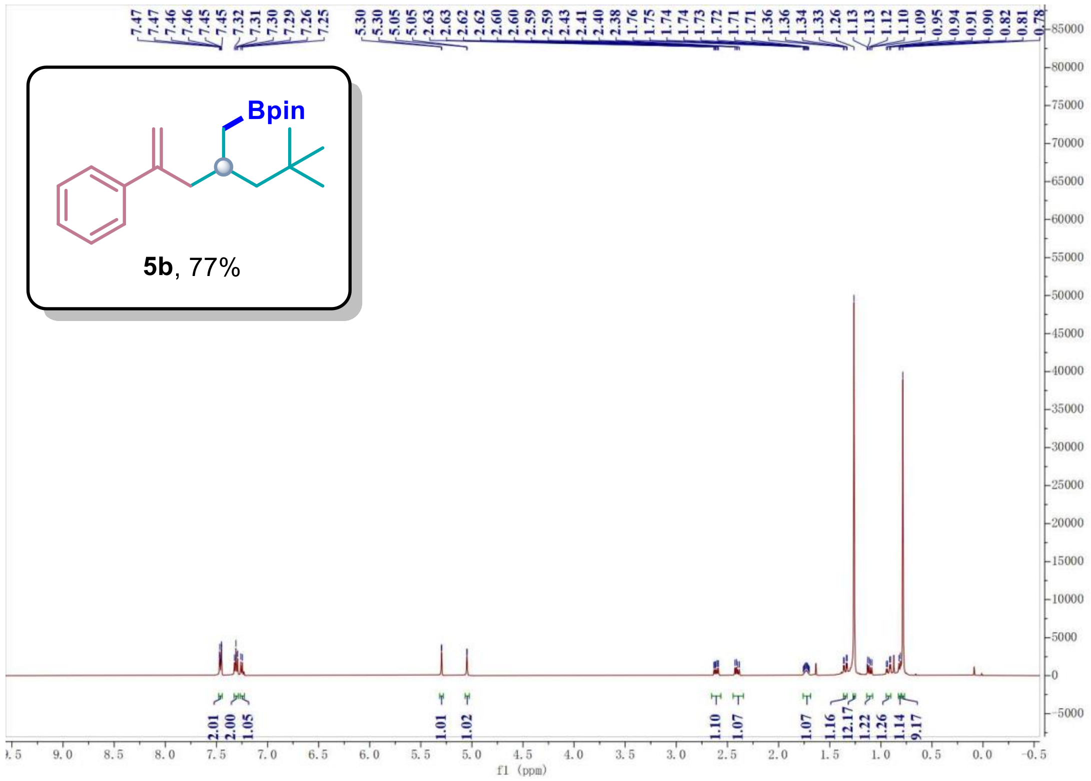

^{13}C NMR (126 MHz, Chloroform-*d*)

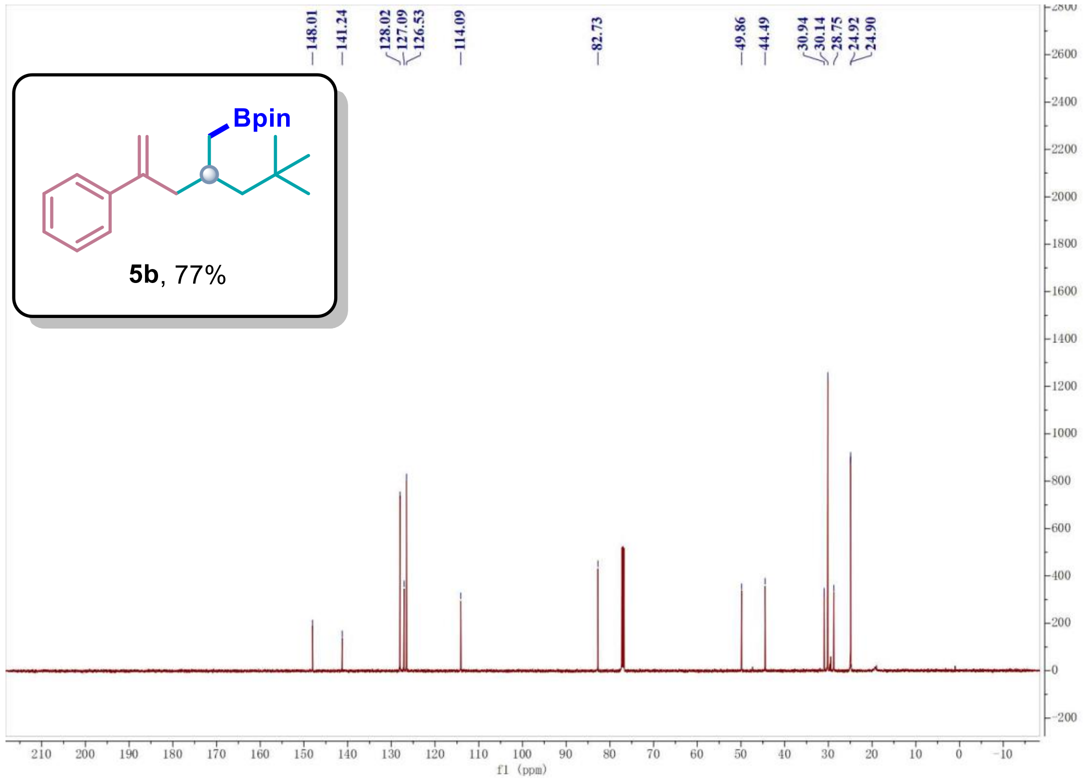

^{11}B NMR (160 MHz, Chloroform-d)

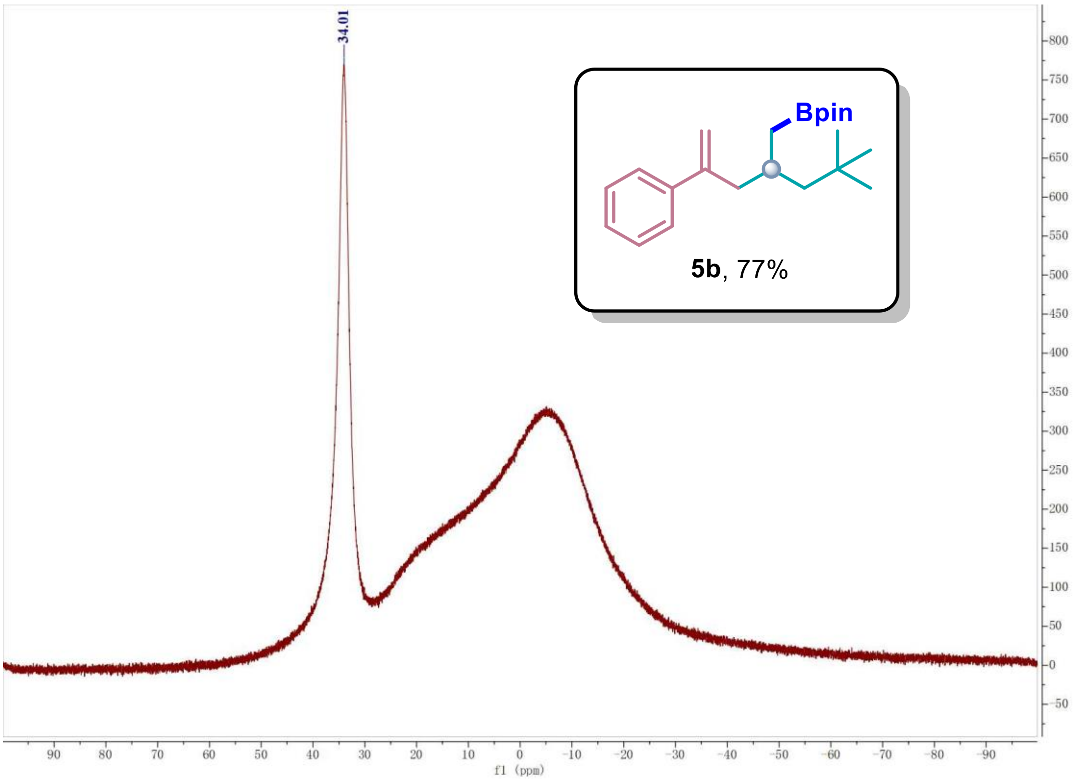

4,4,5,5-tetramethyl-2-(2-neopentyl-4-(p-tolyl)pent-4-en-1-yl)-1,3,2-dioxaborolane (5c)

^{1}H NMR (500 MHz, Chloroform-*d*)

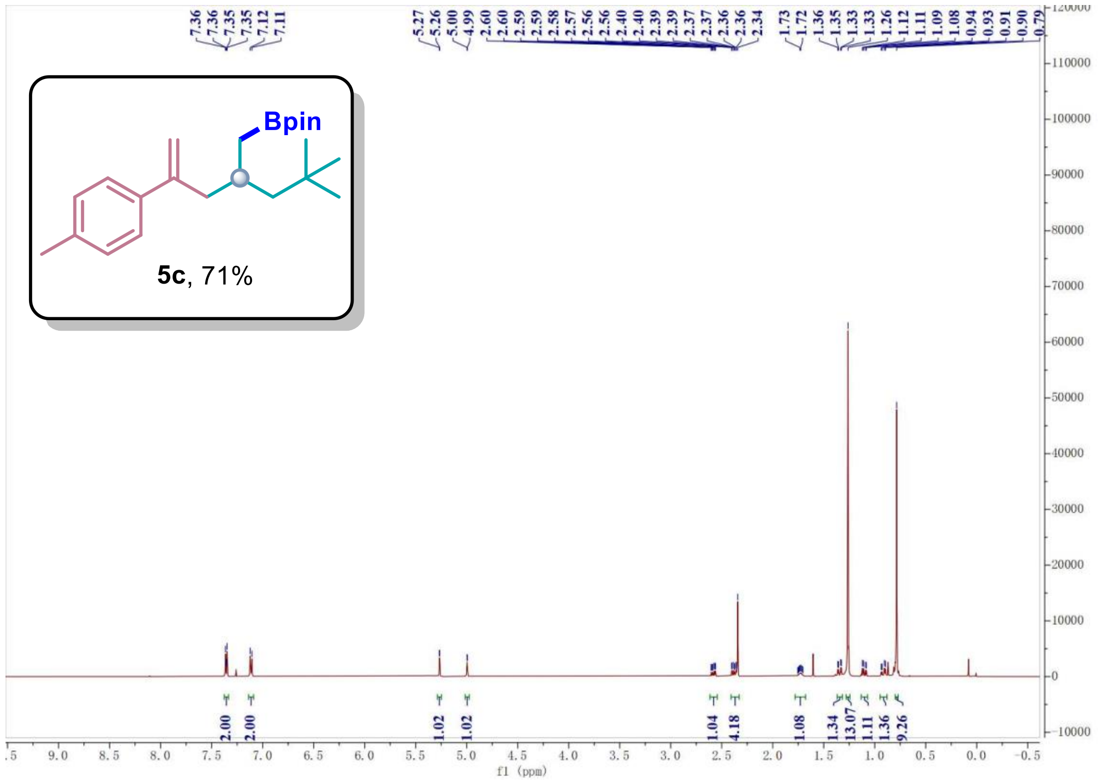

^{13}C NMR (126 MHz, Chloroform-*d*)

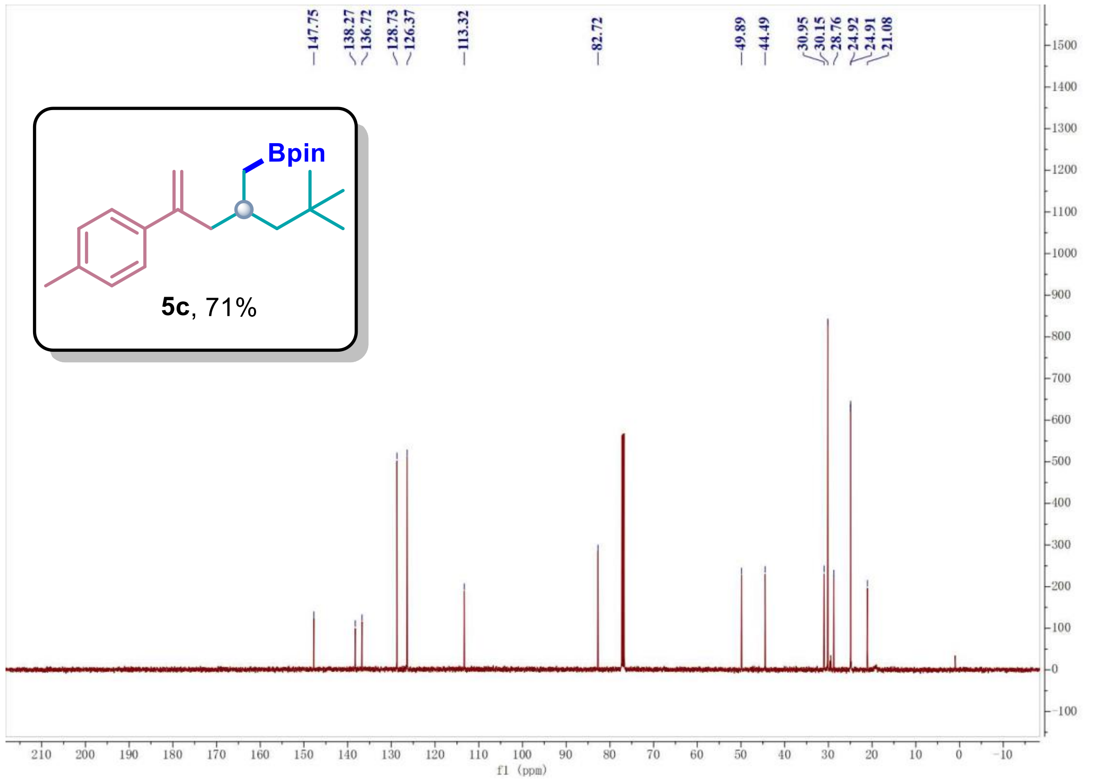

^{11}B NMR (160 MHz, Chloroform-*d*)

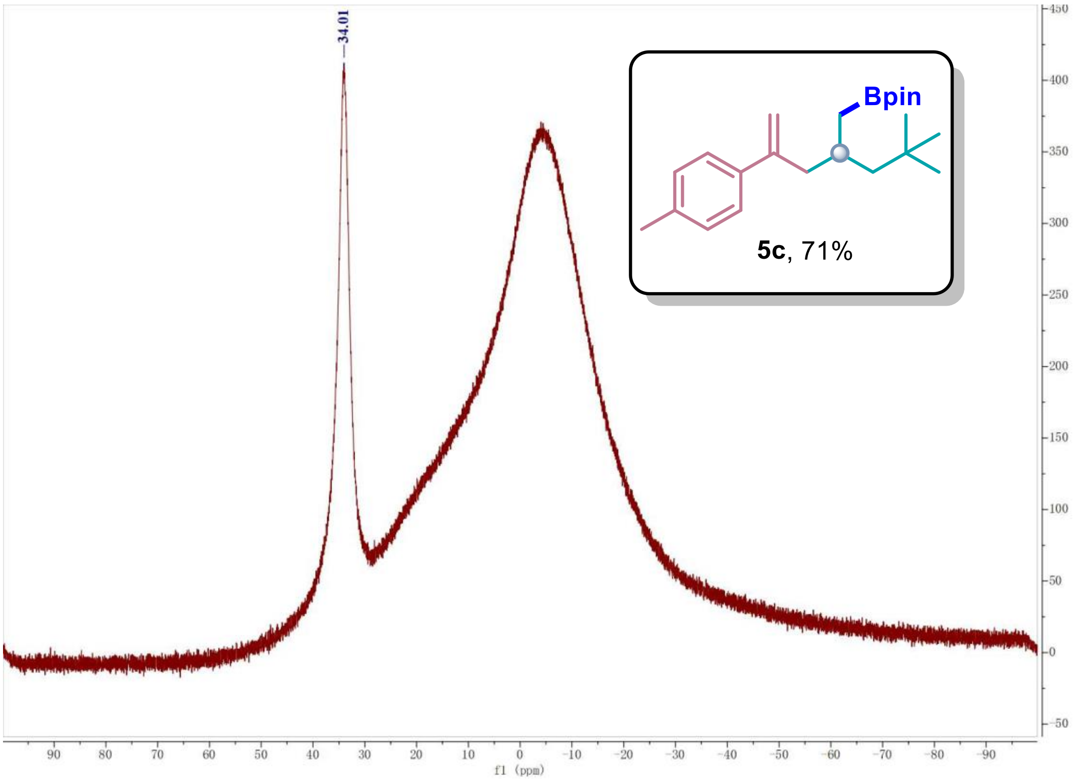

2-(4-(4-fluorophenyl)-2-neopentylpent-4-en-1-yl)-4,4,5,5-tetramethyl-1,3,2-dioxaborolane (5d)

^{1}H NMR (500 MHz, Chloroform-*d*)

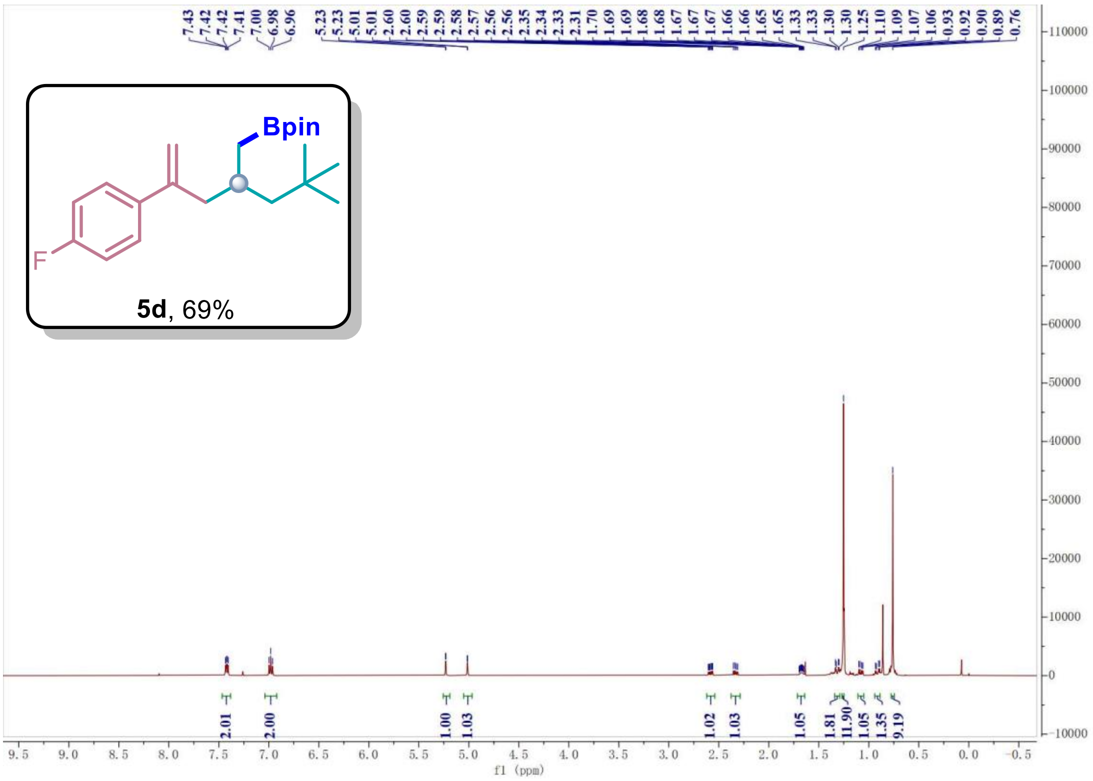

^{13}C NMR (126 MHz, Chloroform-*d*)

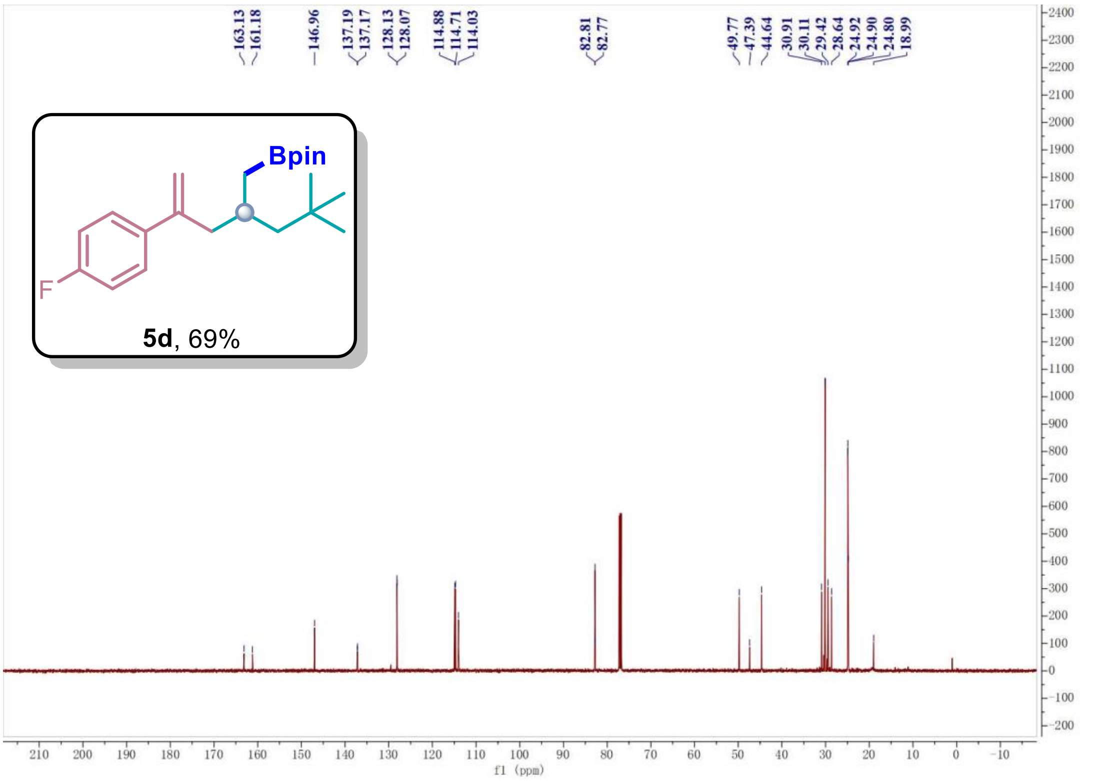

^{11}B NMR (160 MHz, Chloroform-*d*)

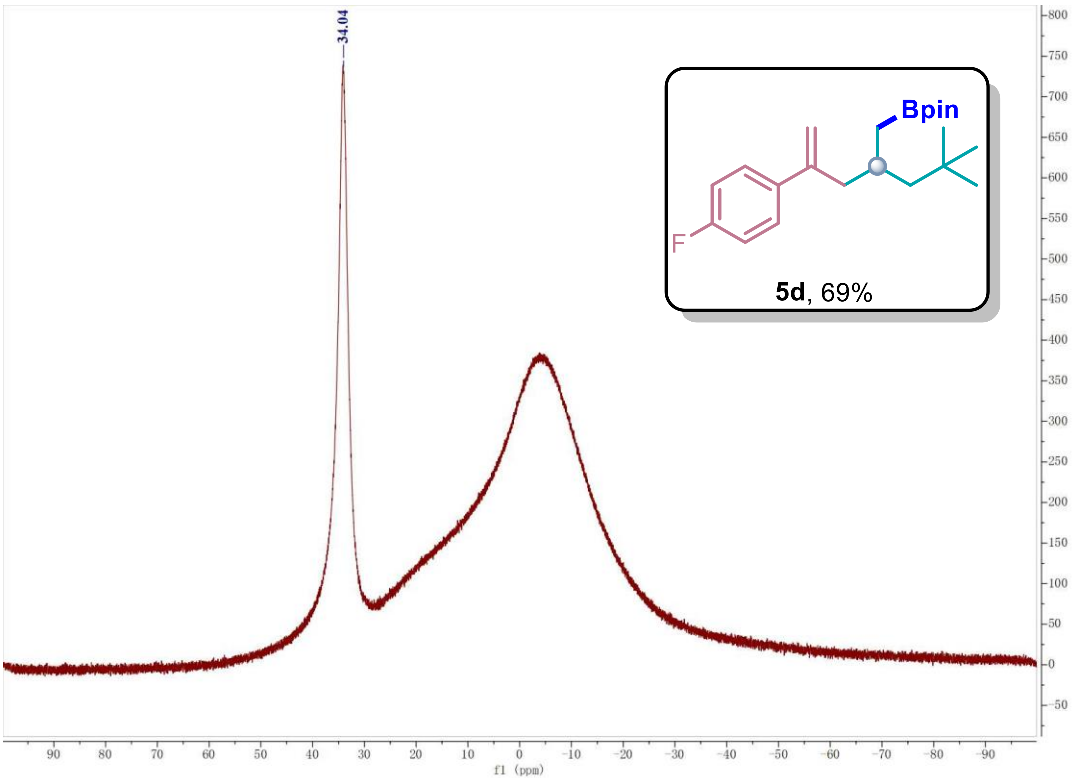

^{19}F NMR (471 MHz, Chloroform-*d*)

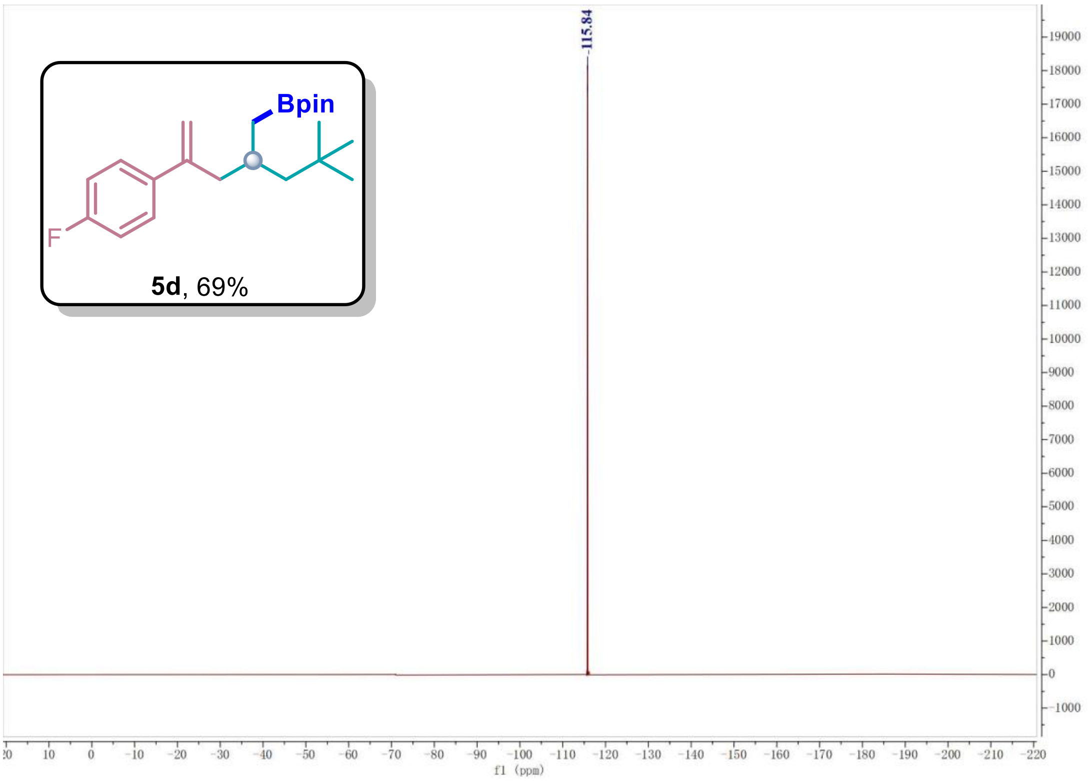

2-(4-(4-chlorophenyl)-2-neopentylpent-4-en-1-yl)-4,4,5,5-tetramethyl-1,3,2-dioxaborolane (5e)

^{1}H NMR (500 MHz, Chloroform-*d*)

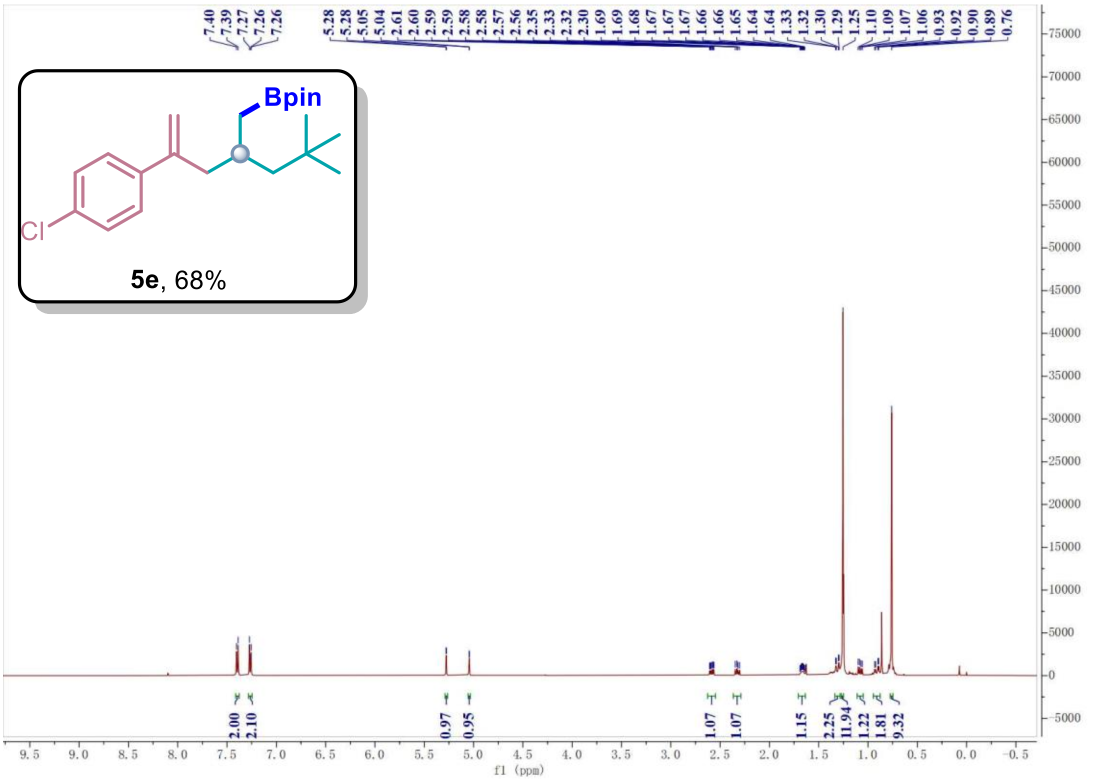

^{13}C NMR (126 MHz, Chloroform-*d*)

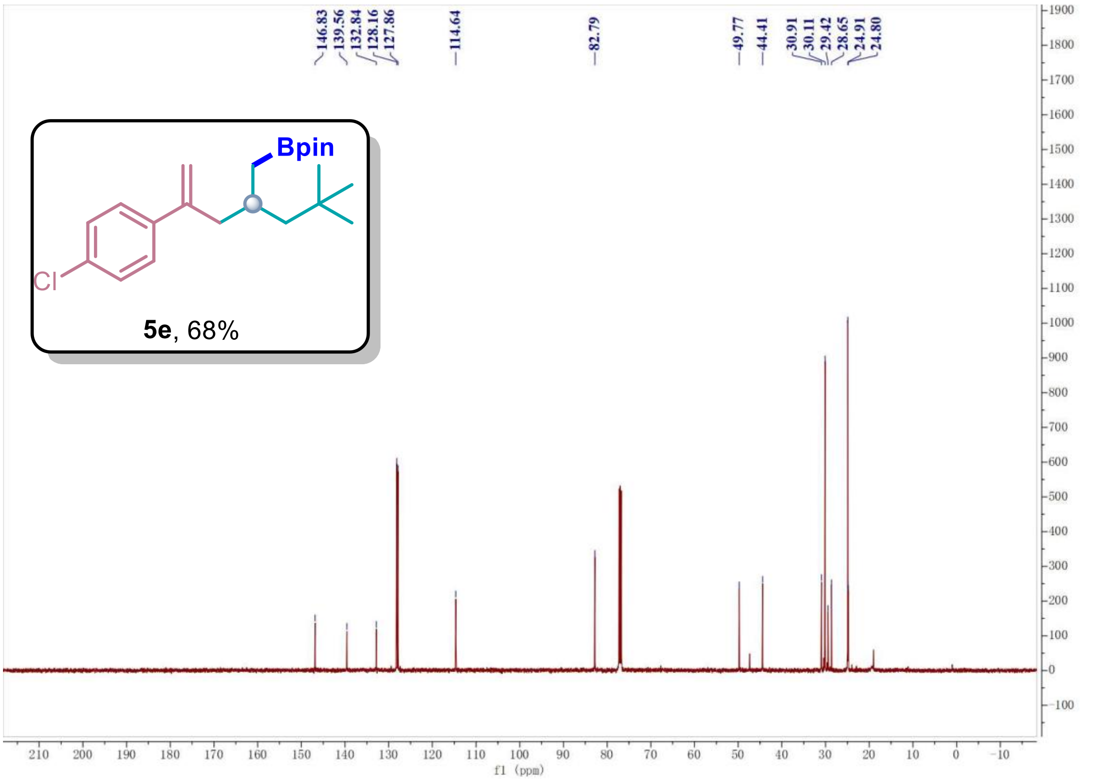

^{11}B NMR (160 MHz, Chloroform-*d*)

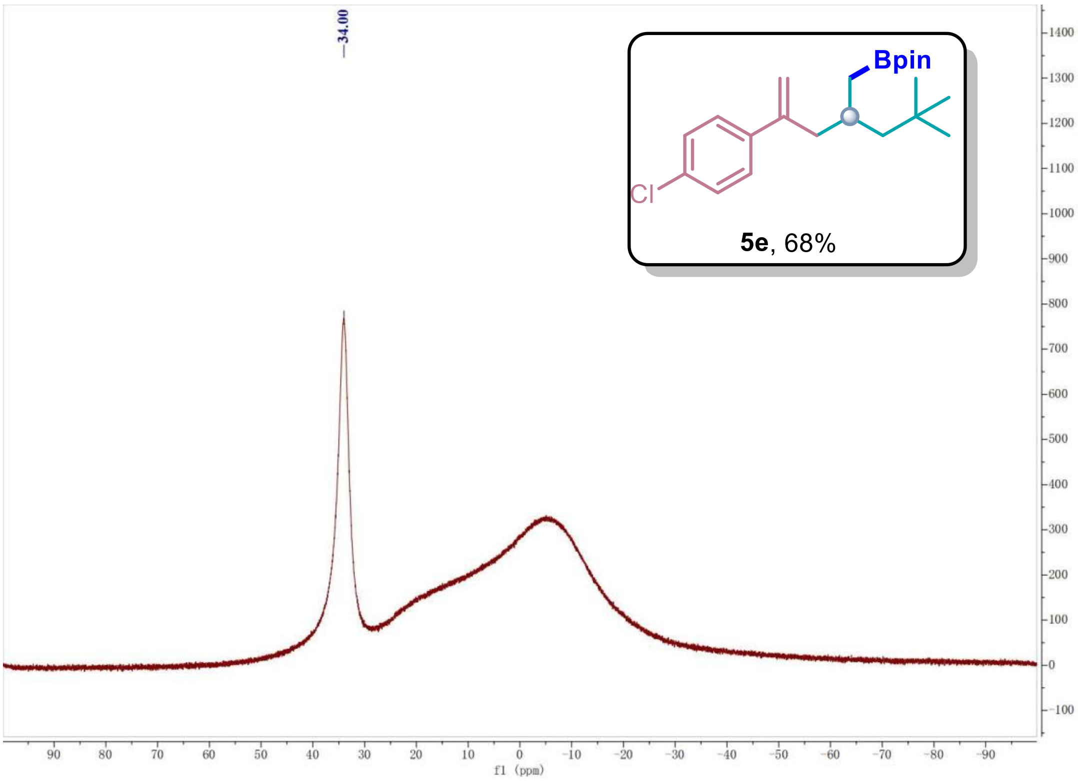

2-(4-(4-bromophenyl)-2-neopentylpent-4-en-1-yl)-4,4,5,5-tetramethyl-1,3,2-dioxaborolane (5f)

^{1}H NMR (500 MHz, Chloroform-*d*)

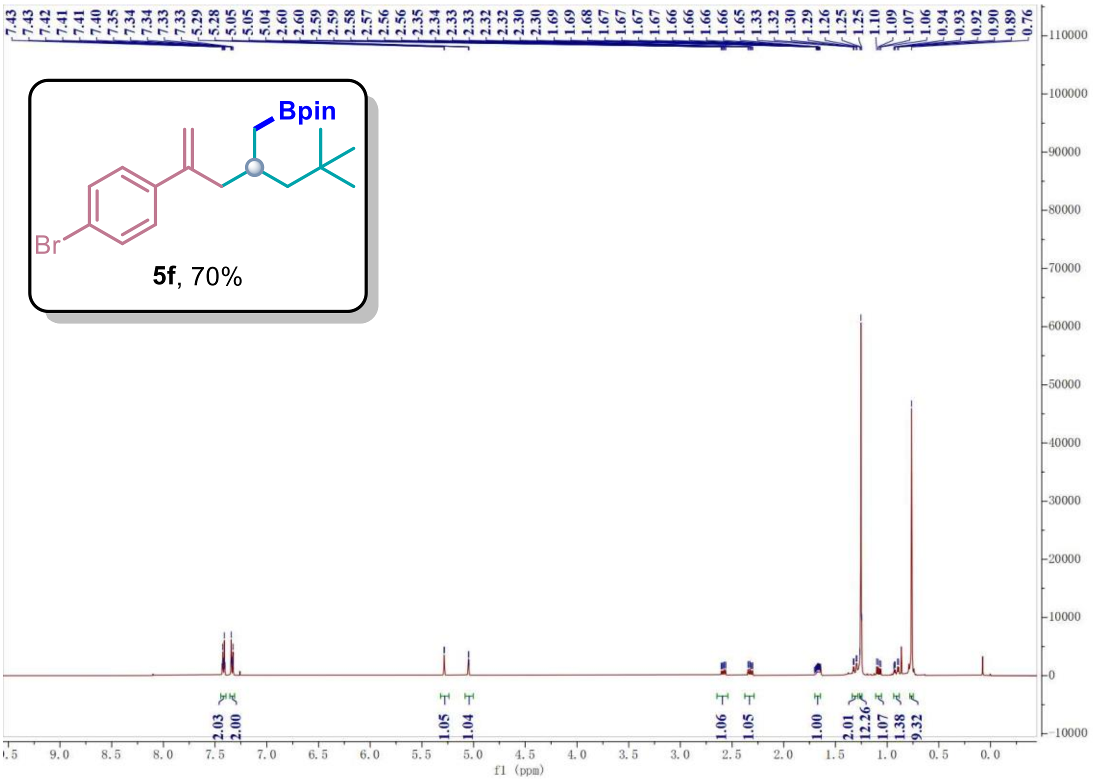

^{13}C NMR (126 MHz, Chloroform-*d*)

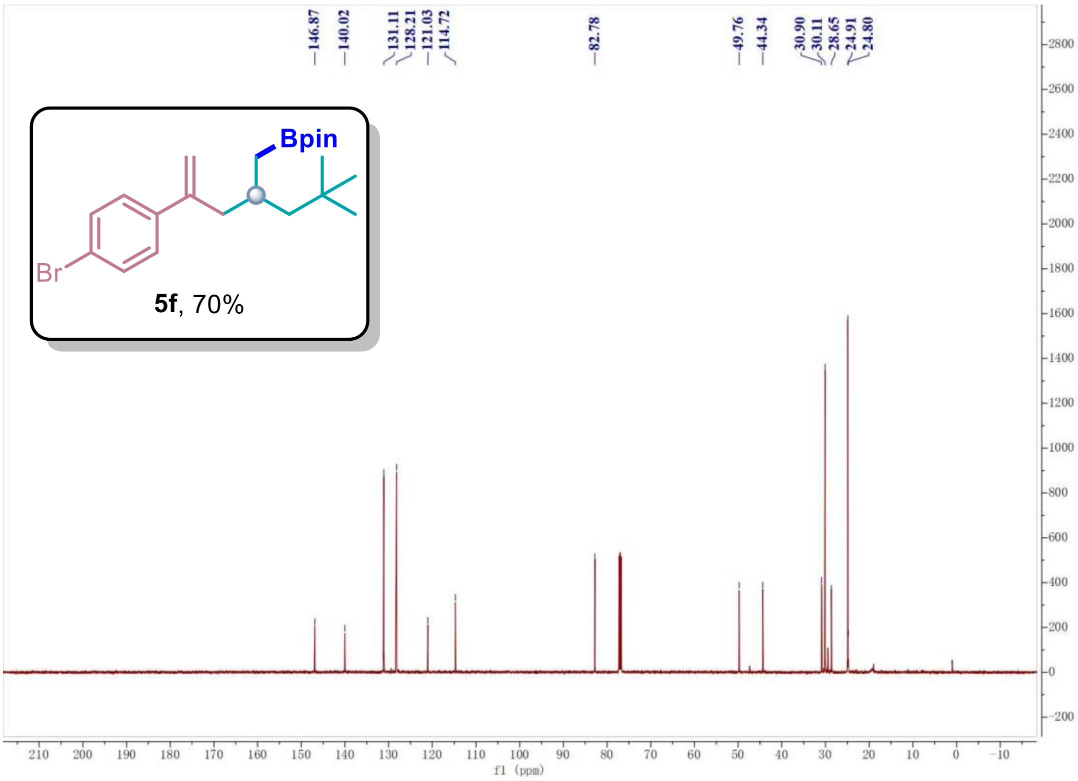

^{11}B NMR (160 MHz, Chloroform-*d*)

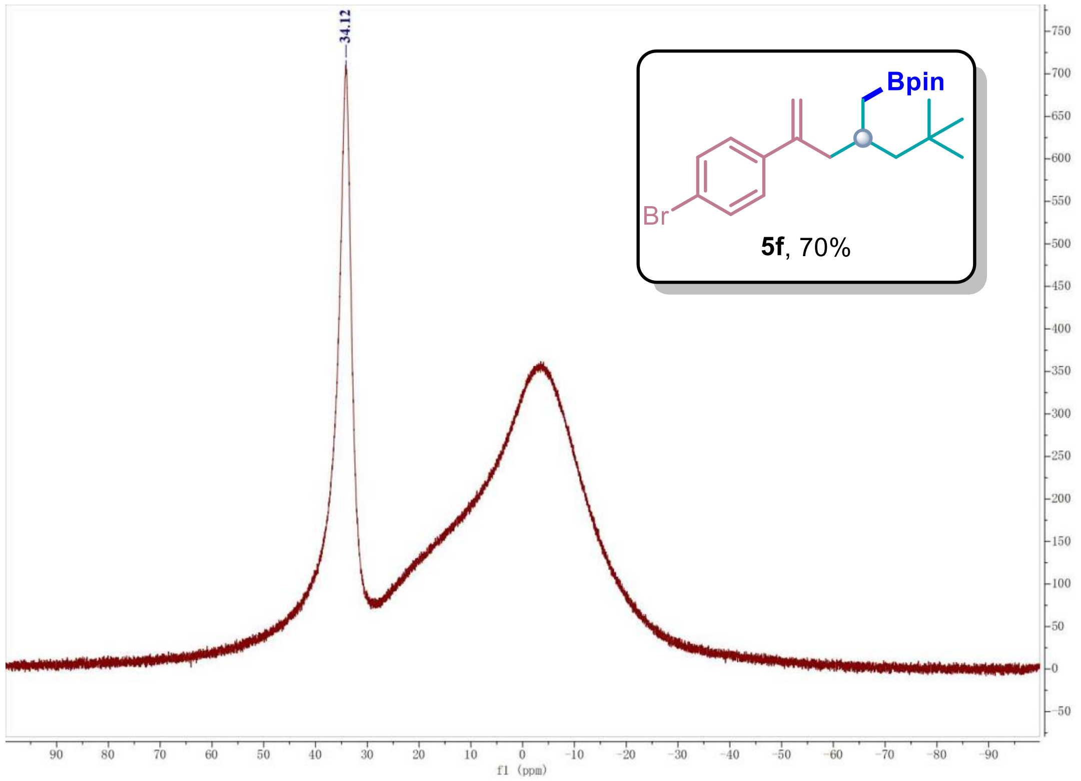

2-(4-(4-methoxyphenyl)-2-neopentylpent-4-en-1-yl)-4,4,5,5-tetramethyl-1,3,2-dioxaborolane (5g)

^{1}H NMR (500 MHz, Chloroform-*d*)

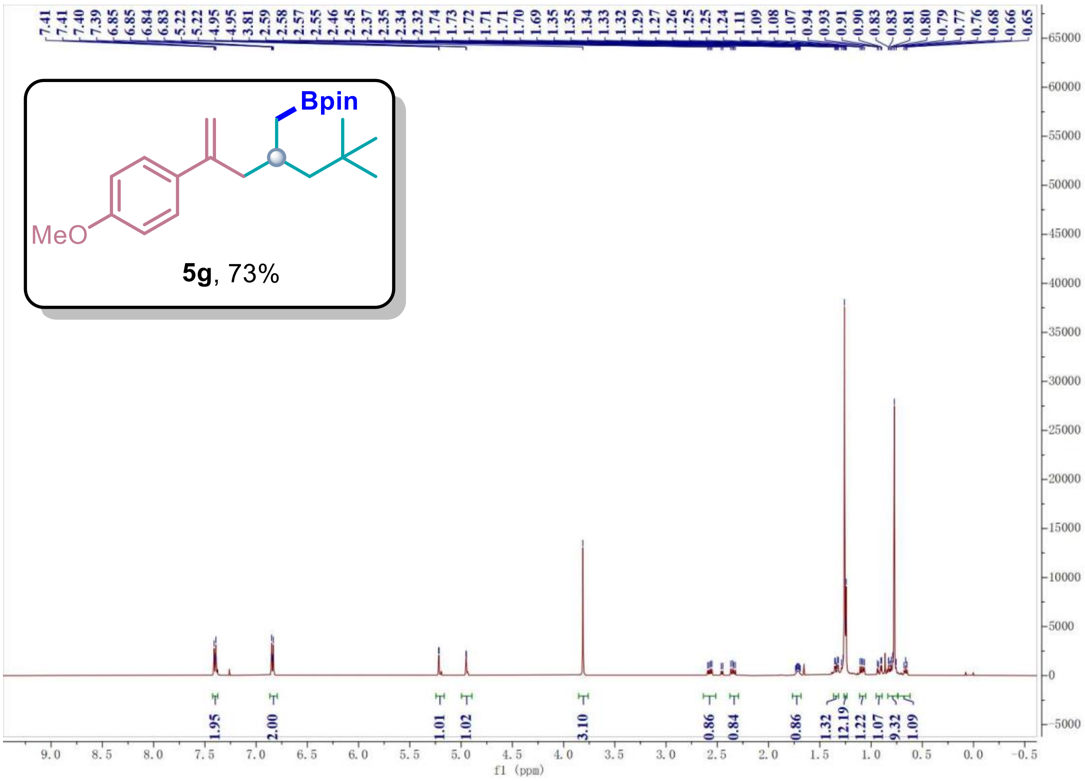

^{13}C NMR (126 MHz, Chloroform-*d*)

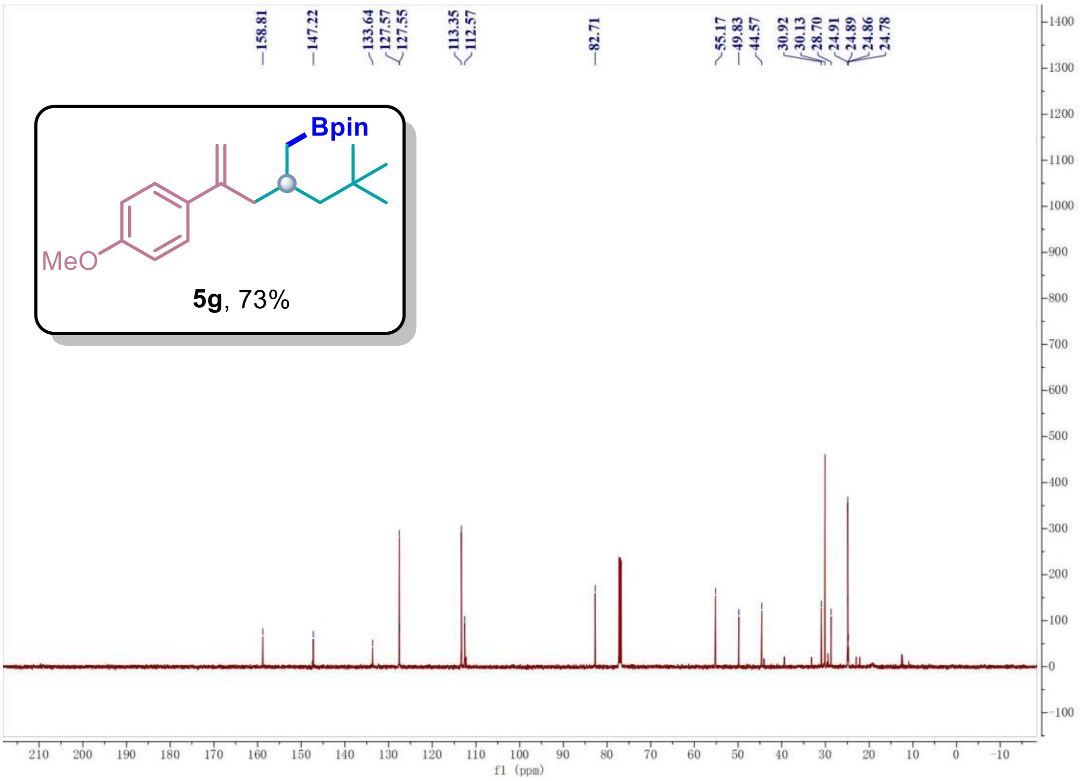

^{11}B NMR (160 MHz, Chloroform-*d*)

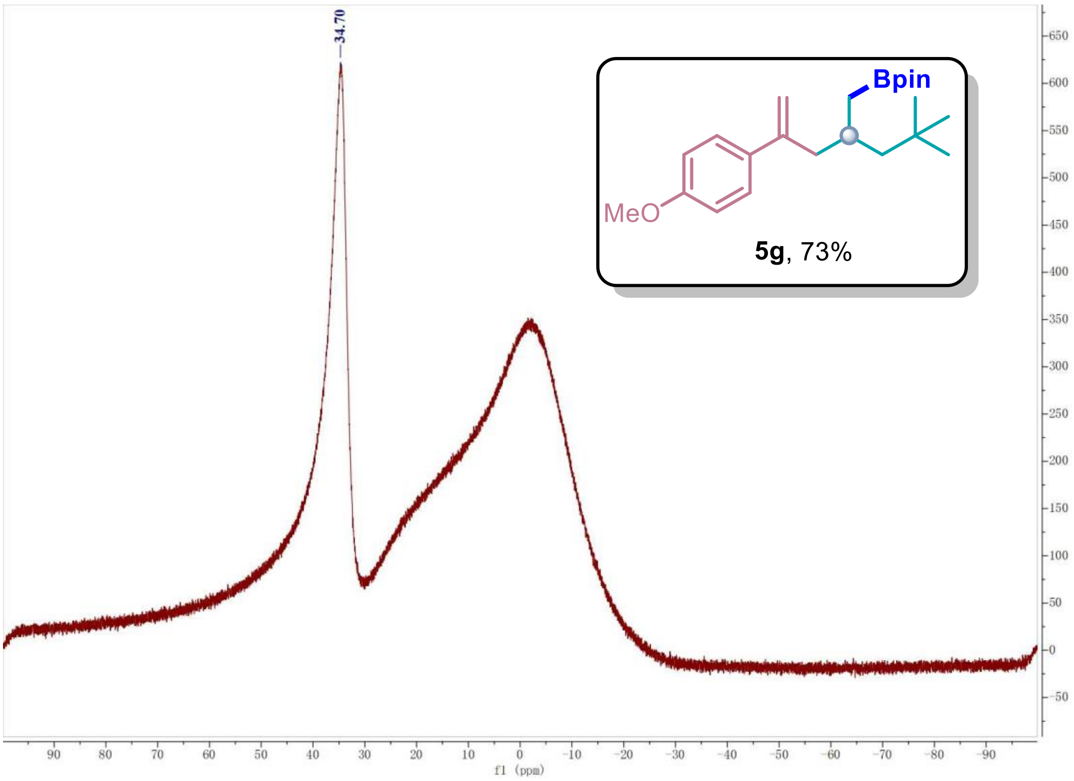

2-(4-(3,4-dimethoxyphenyl)-2-neopentylpent-4-en-1-yl)-4,4,5,5-tetramethyl-1,3,2-dioxaborolane (5h)

^{1}H NMR (500 MHz, Chloroform-*d*)

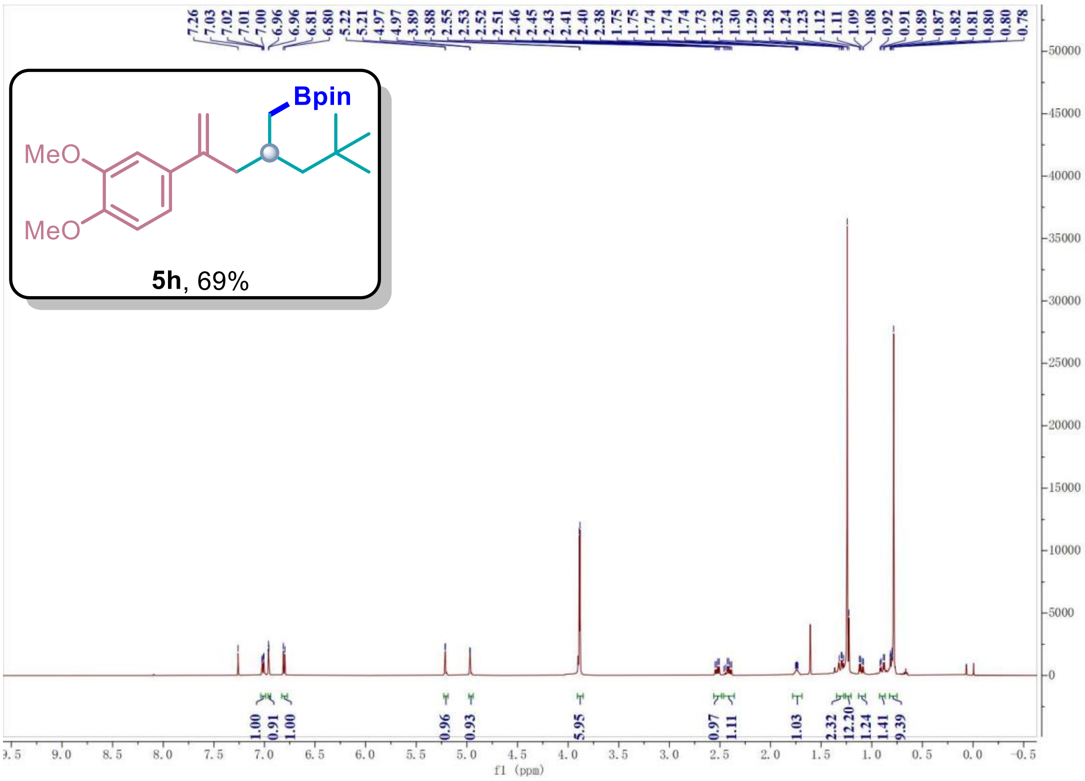

^{13}C NMR (126 MHz, Chloroform-*d*)

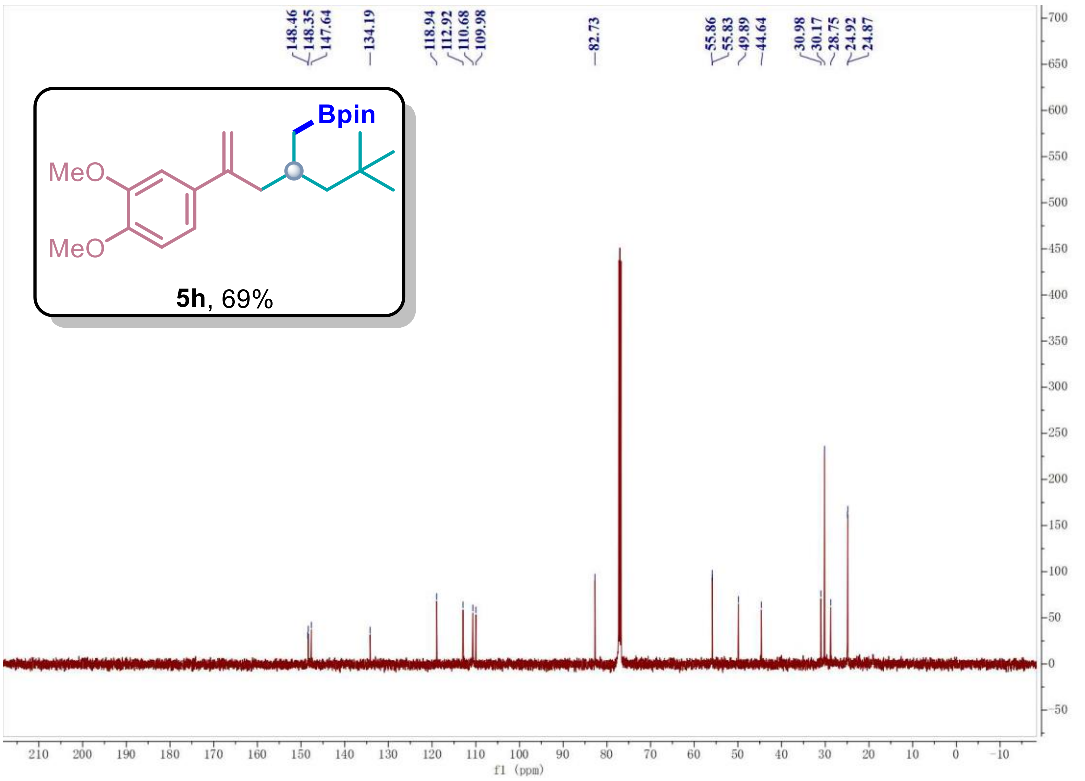

^{11}B NMR (160 MHz, Chloroform-*d*)

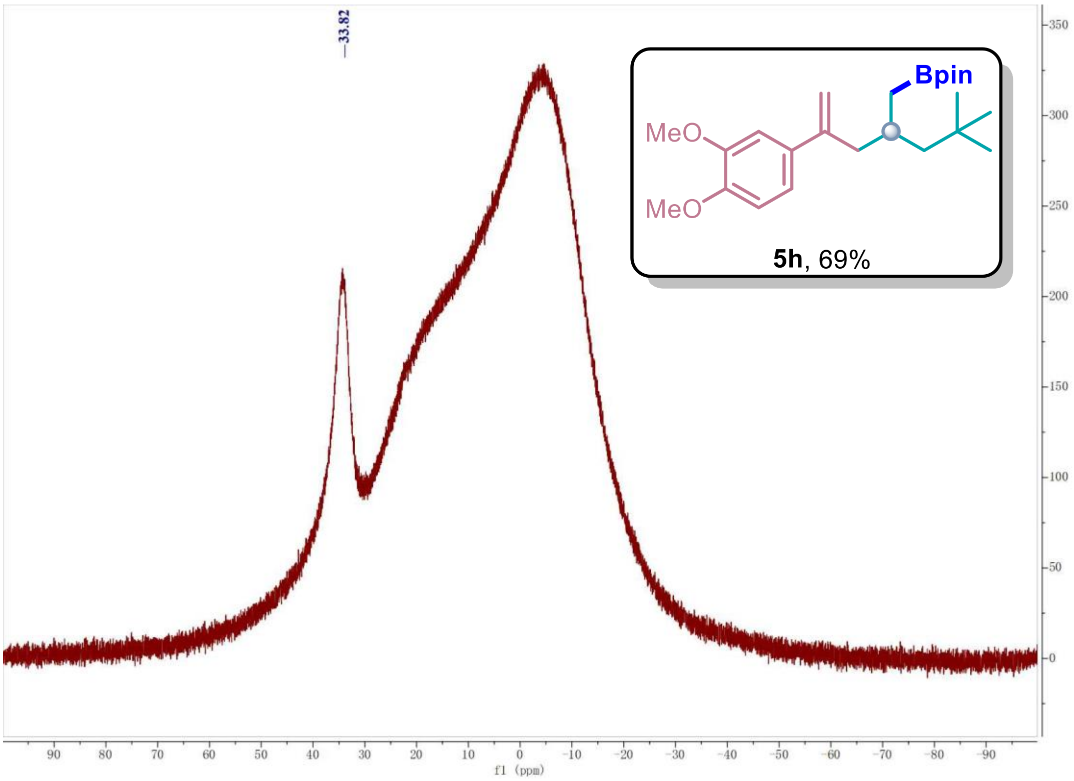

4,4,5,5-tetramethyl-2-(4-(naphthalen-2-yl)-2-neopentylpent-4-en-1-yl)-1,3,2-dioxaborolane (5i)

^{1}H NMR (500 MHz, Chloroform-*d*)

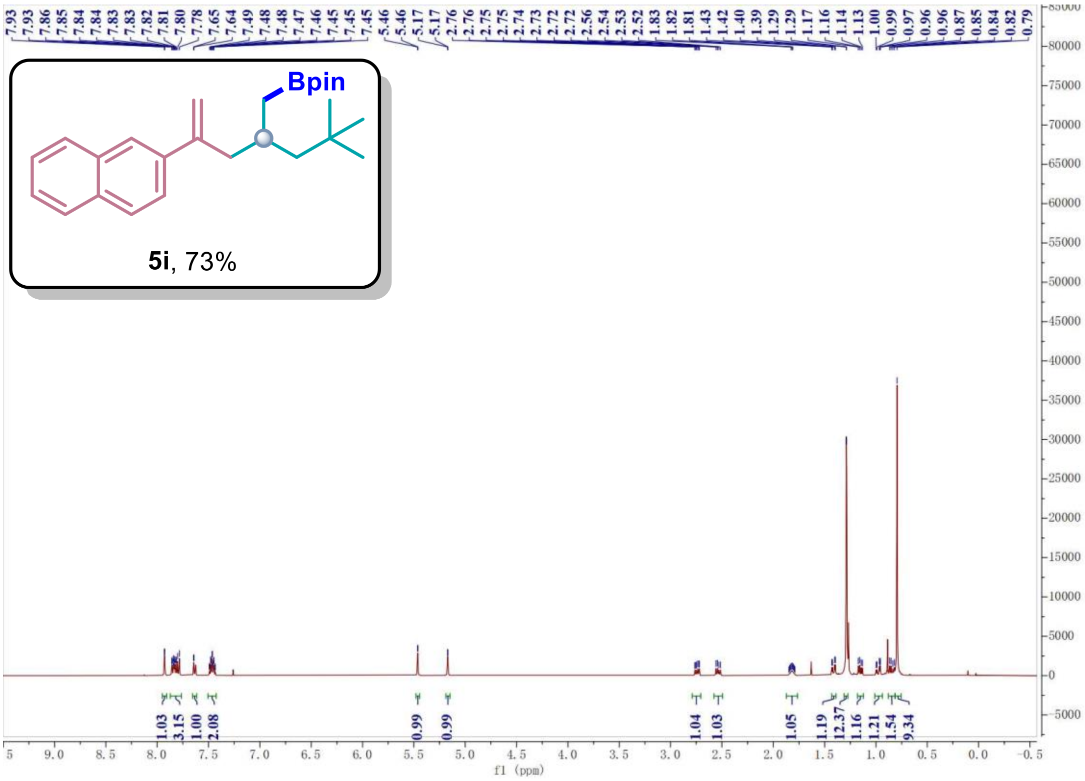

^{13}C NMR (126 MHz, Chloroform-*d*)

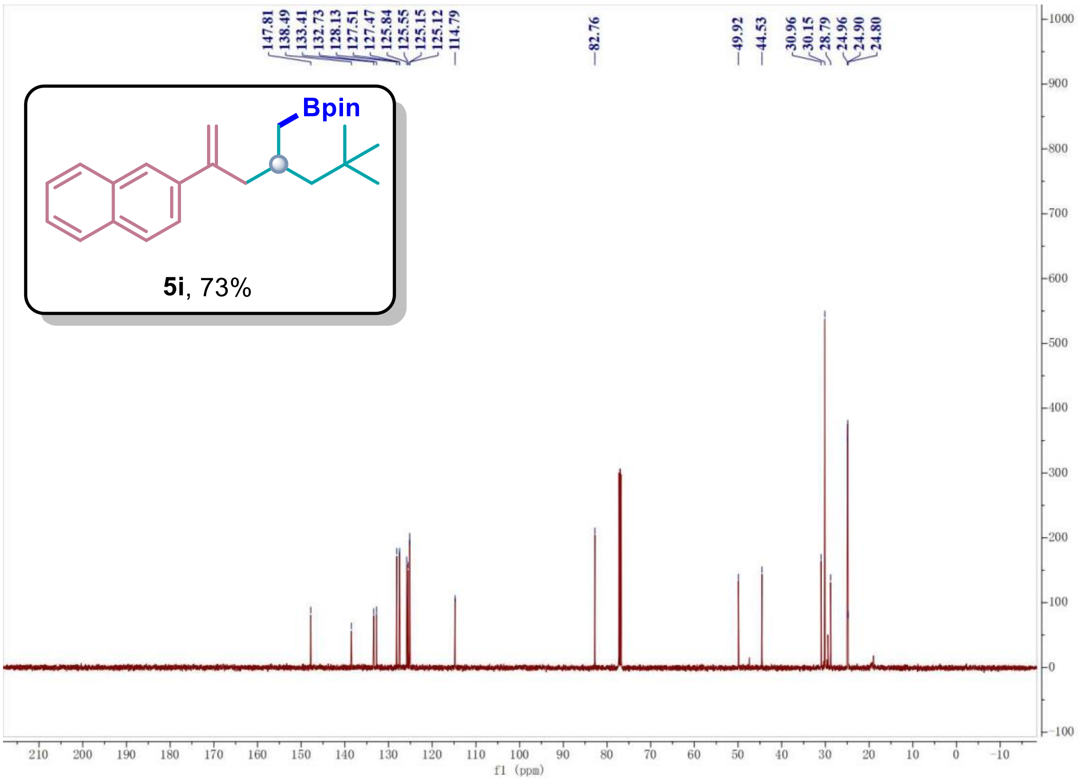

^{11}B NMR (160 MHz, Chloroform-d)

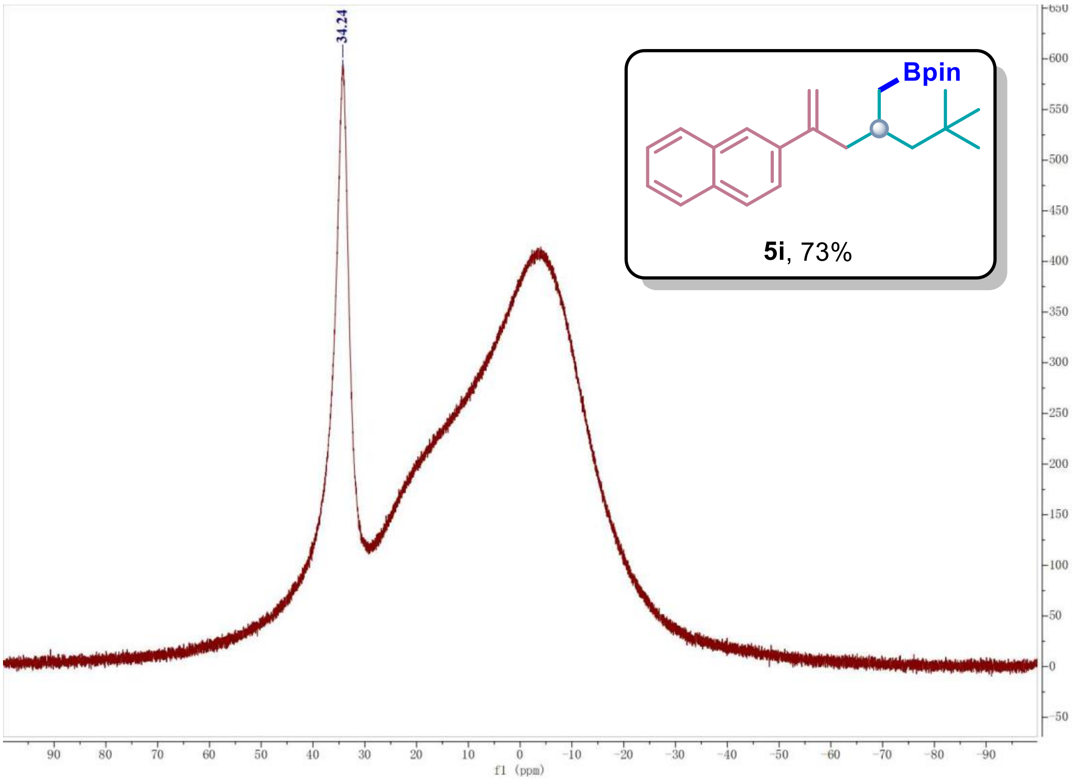

4,4,5,5-tetramethyl-2-(2-neopentyl-4-(thiophen-2-yl)pent-4-en-1-yl)-1,3,2-dioxaborolane (5j)

^{1}H NMR (500 MHz, Chloroform-*d*)

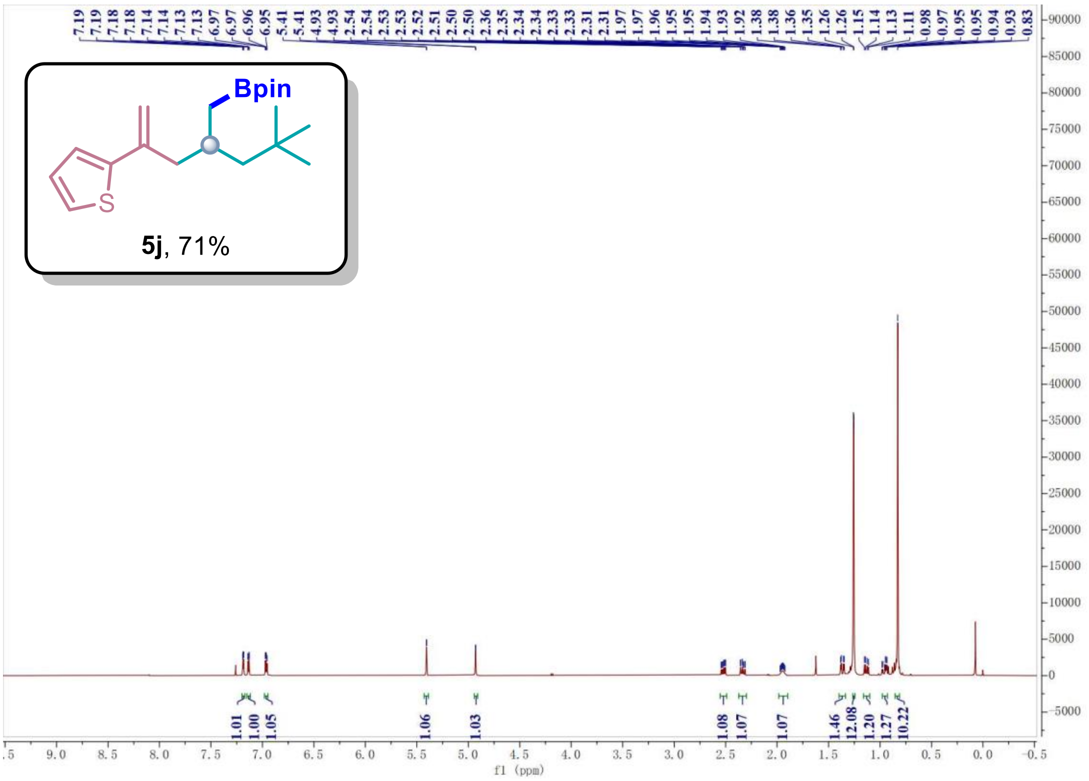

^{13}C NMR (126 MHz, Chloroform-*d*)

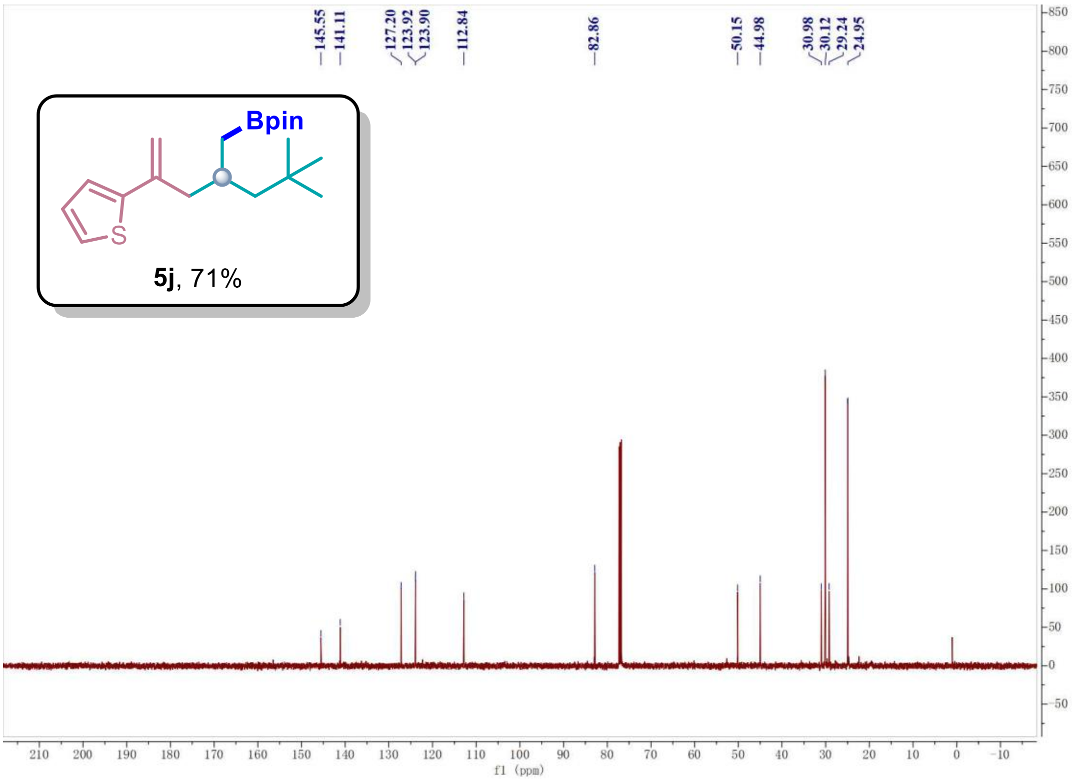

^{11}B NMR (160 MHz, Chloroform-*d*)

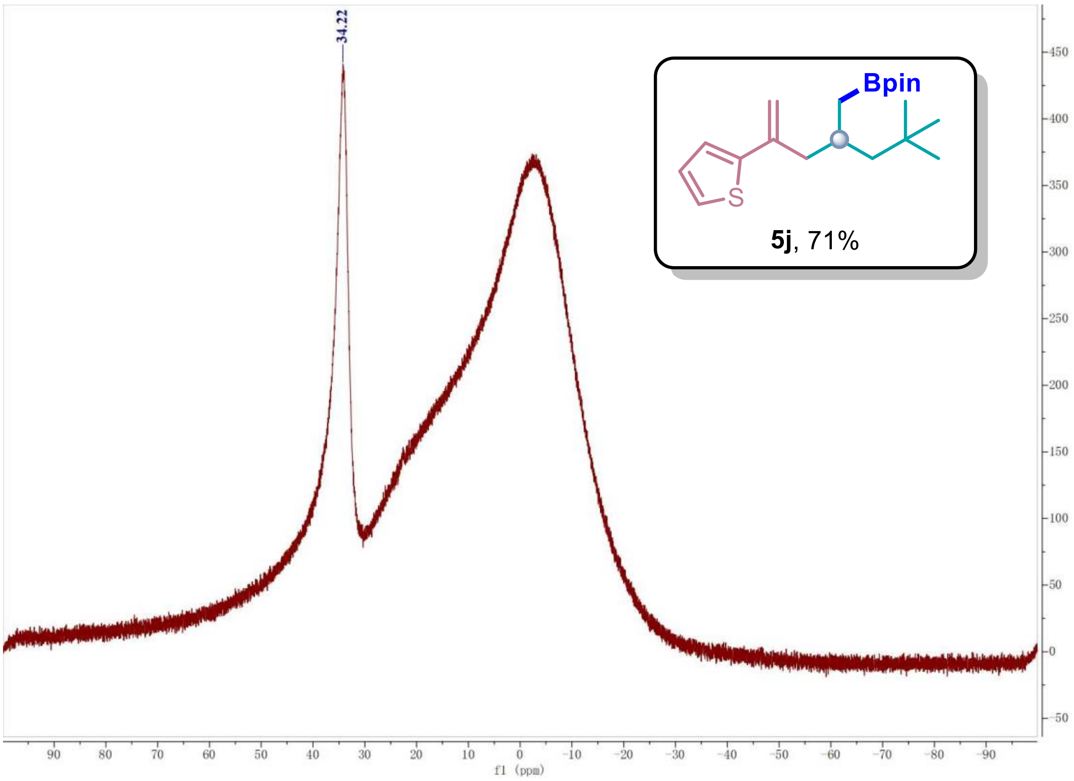

2-(4-chloro-2-neopentylpent-4-en-1-yl)-4,4,5,5-tetramethyl-1,3,2-dioxaborolane (5k)

^{1}H NMR (500 MHz, Chloroform-*d*)

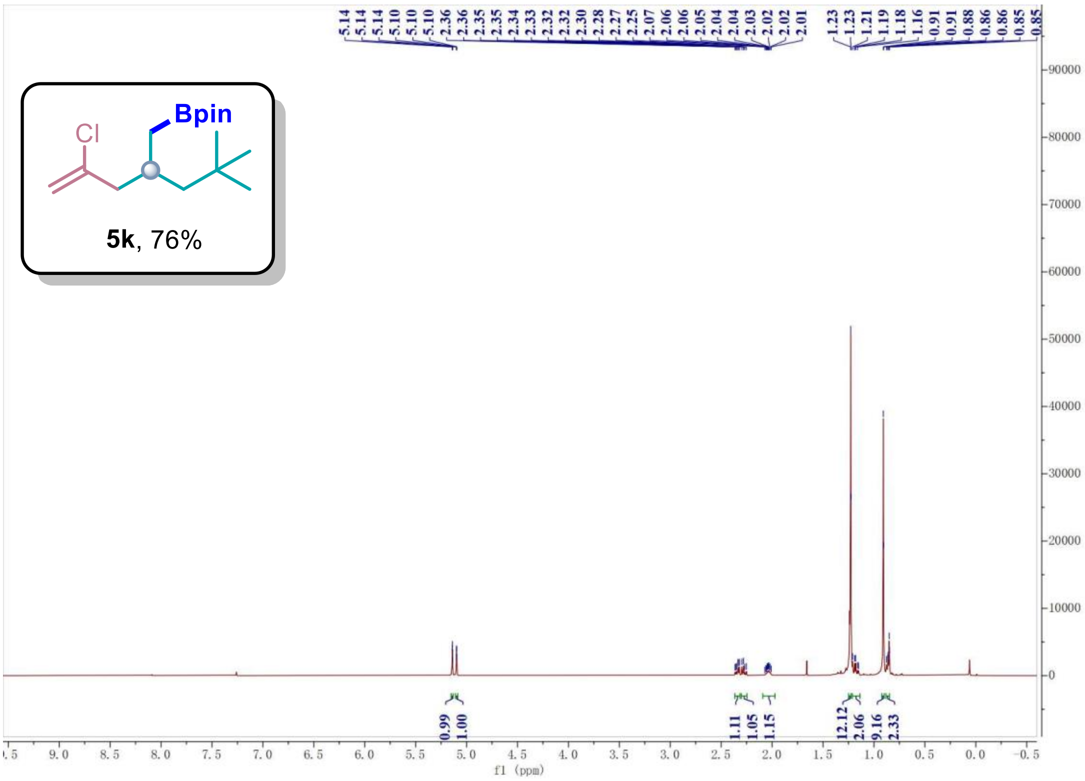

^{13}C NMR (126 MHz, Chloroform-*d*)

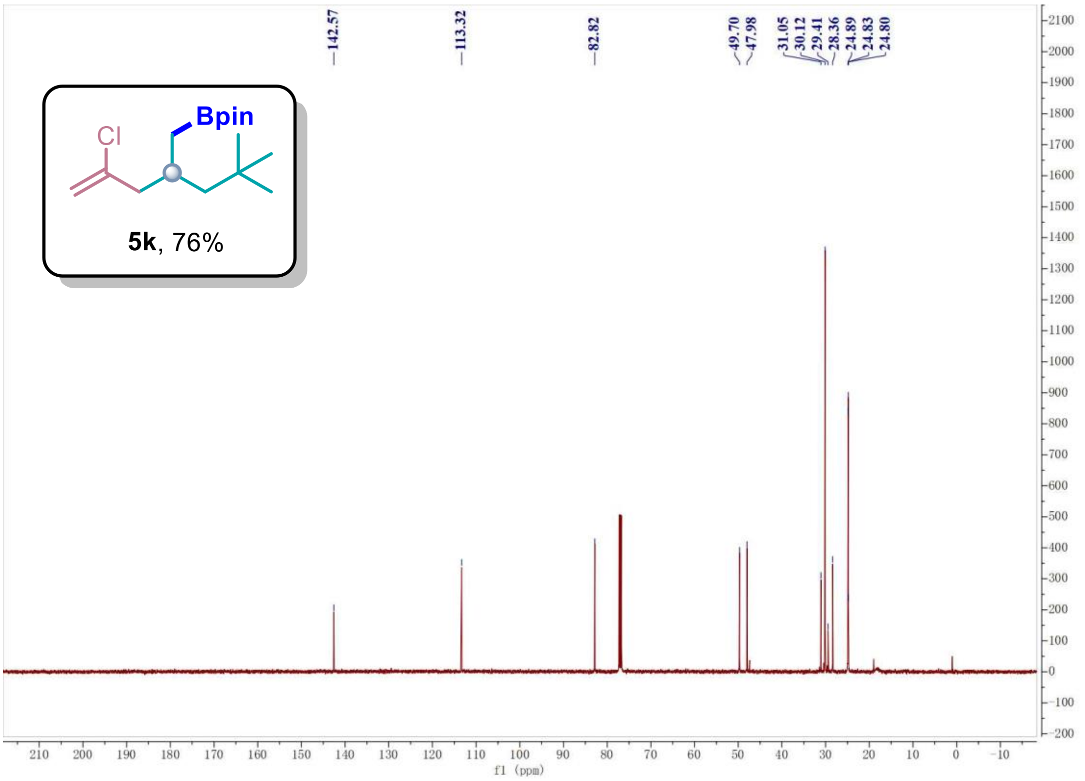

^{11}**B NMR (160 MHz, Chloroform-*d*)**

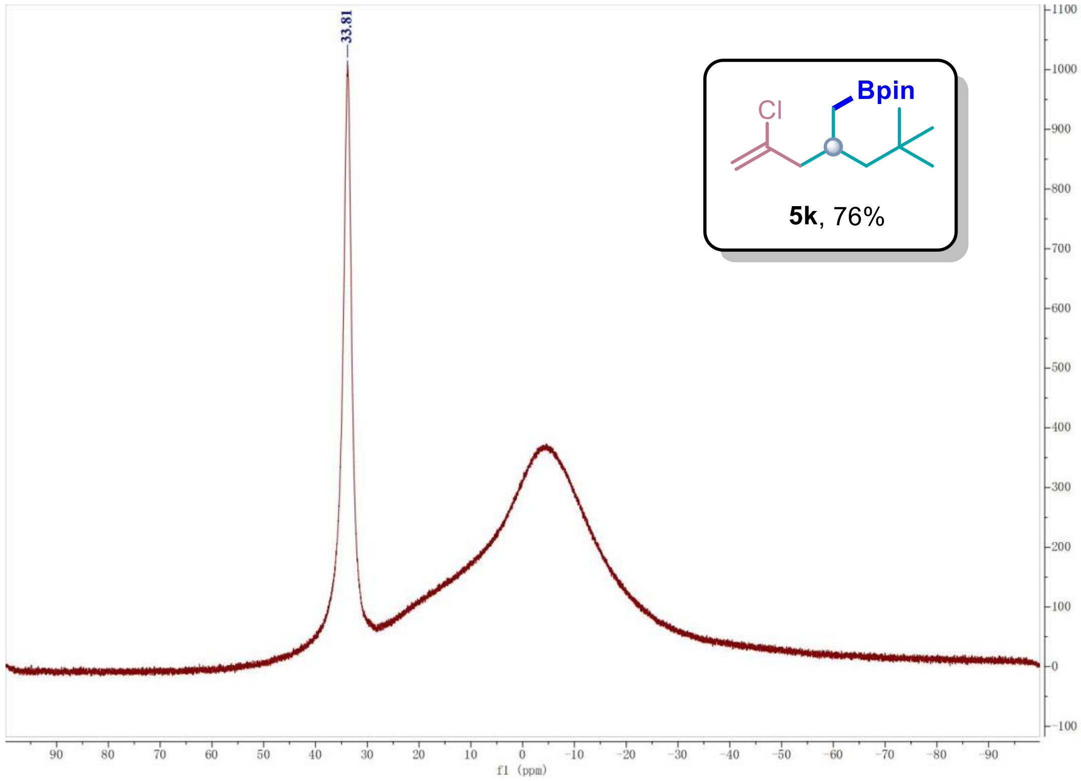

adamantan-1-yl 6,6-dimethyl-2-methylene-4-((4,4,5,5-tetramethyl-1,3,2-dioxaborolan-2-yl)methyl)heptanoate (5l)

^{1}H NMR (500 MHz, Chloroform-*d*)

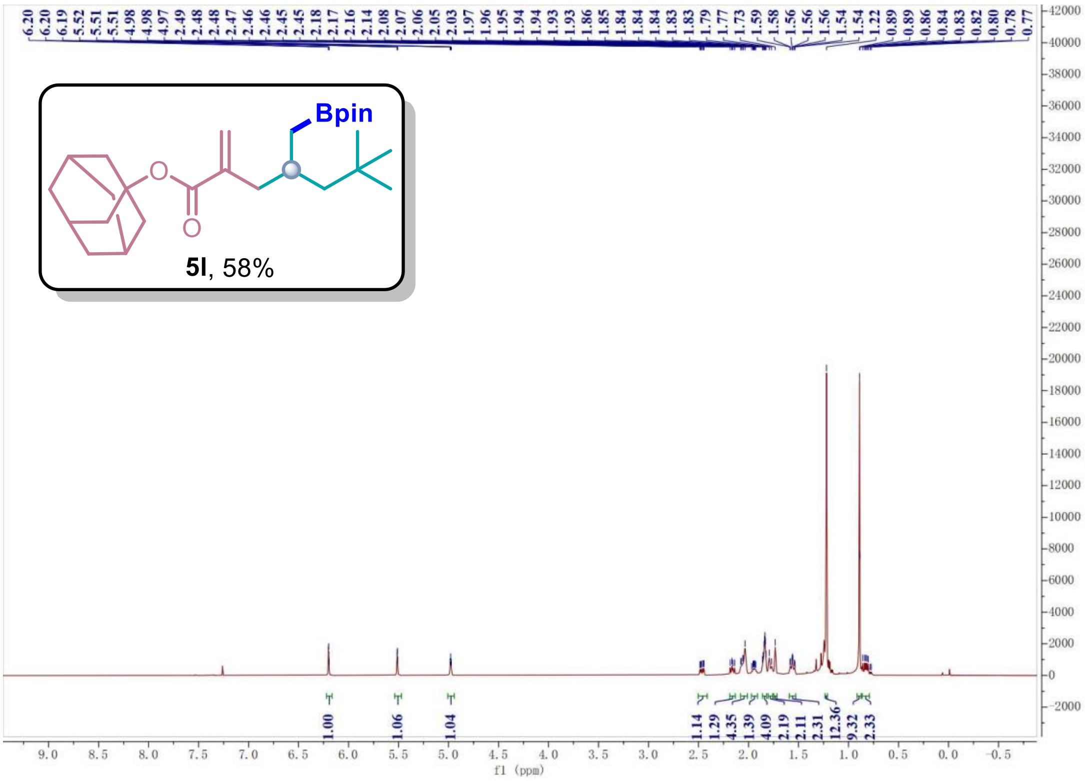

^{13}C NMR (126 MHz, Chloroform-*d*)

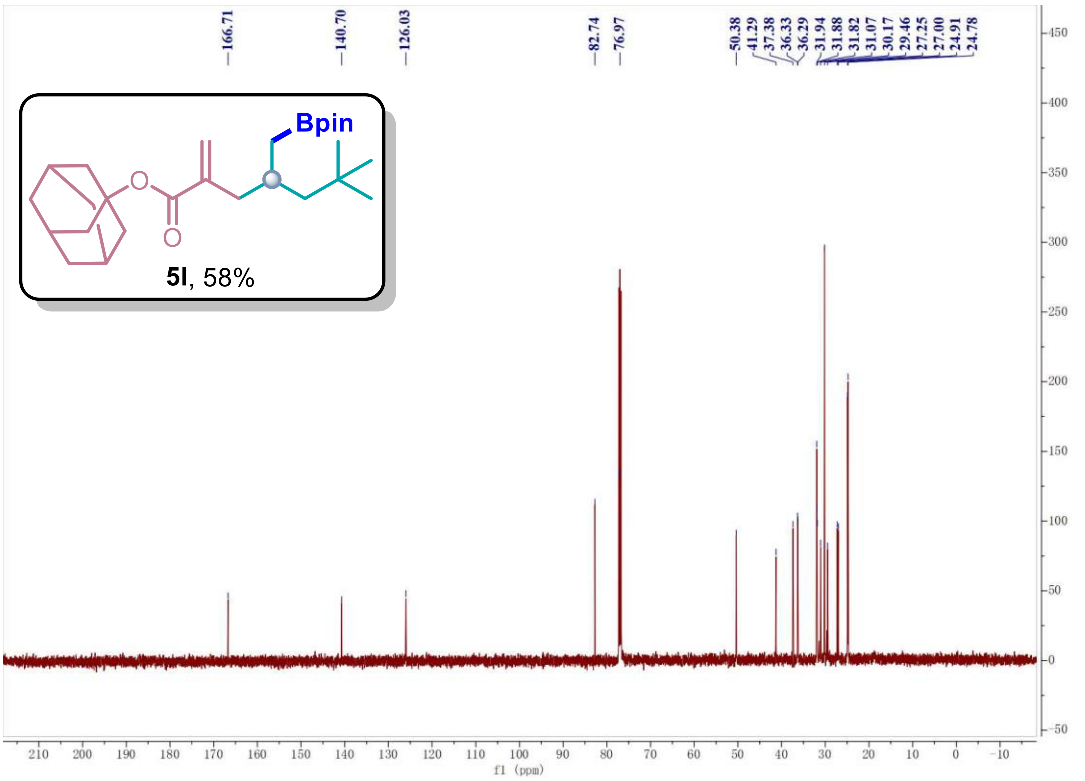

^{11}B NMR (160 MHz, Chloroform-*d*)

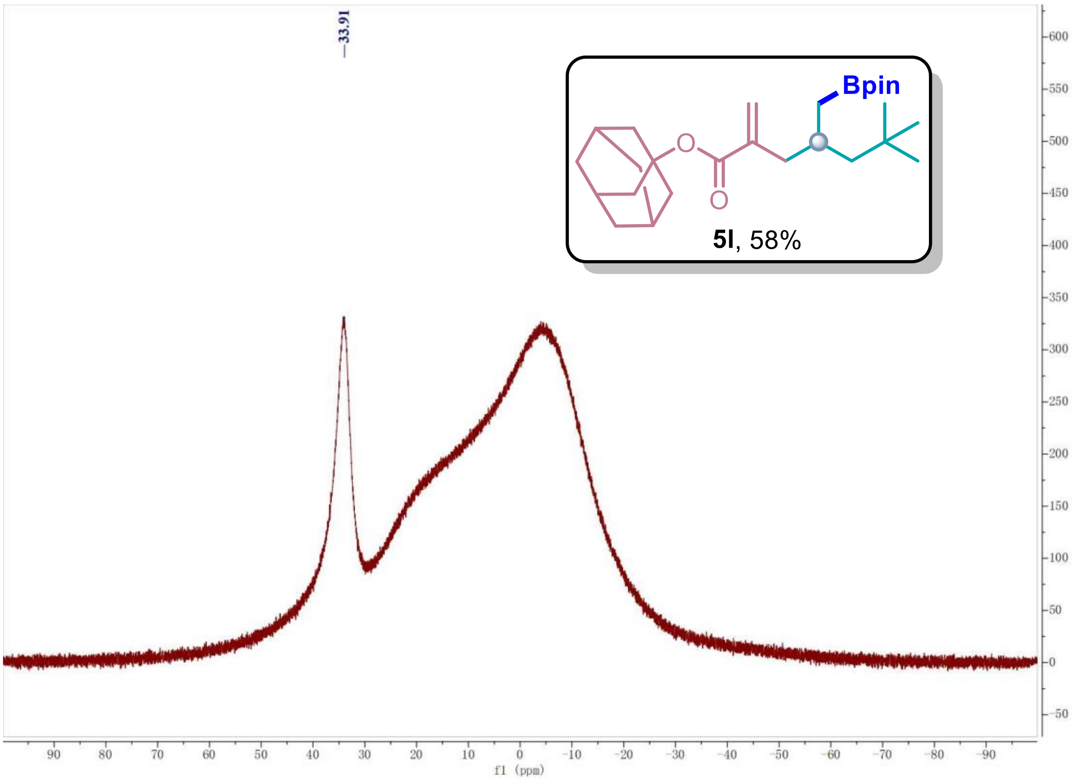

cycloheptyl 6,6-dimethyl-2-methylene-4-((4,4,5,5-tetramethyl-1,3,2-dioxaborolan-2-yl)methyl)heptanoate (5m)

^{1}H NMR (500 MHz, Chloroform-*d*)

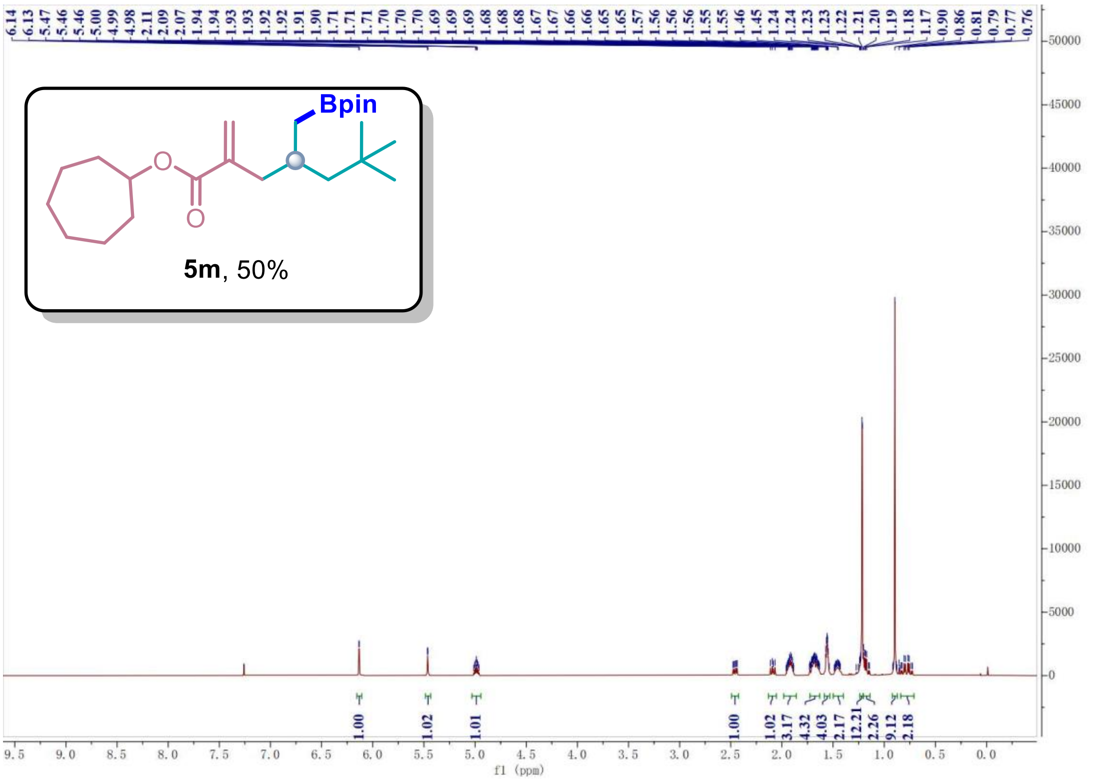

^{13}C NMR (126 MHz, Chloroform-*d*)

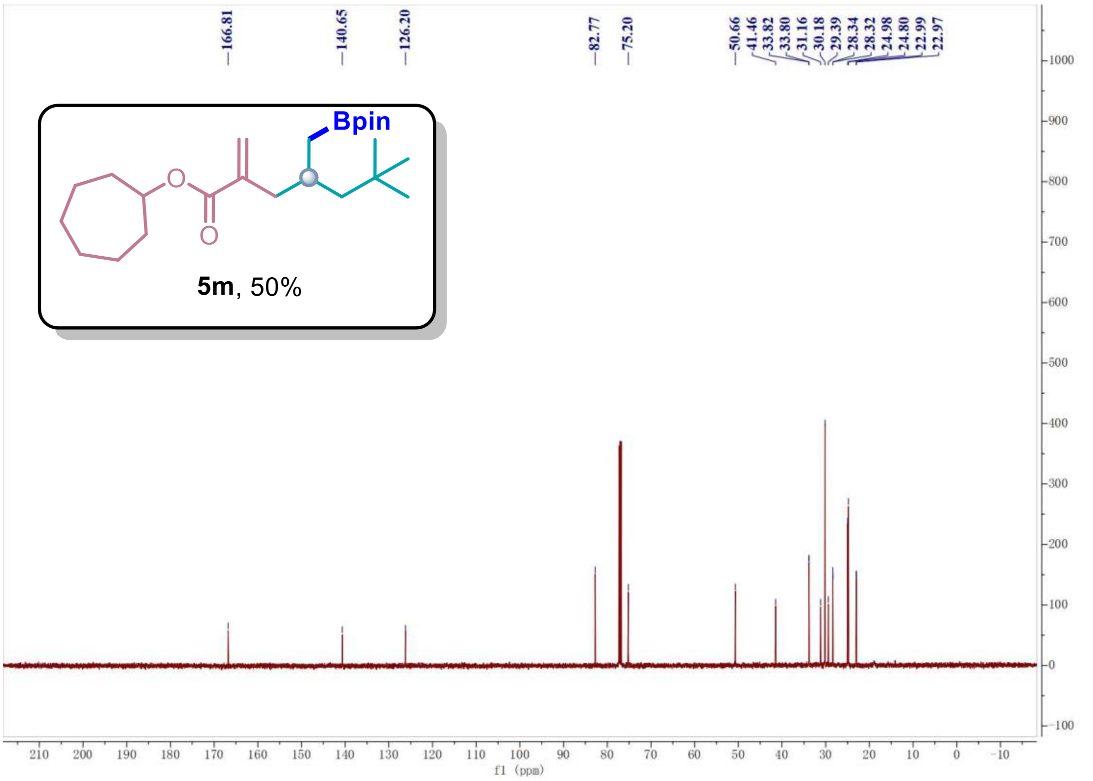

^{11}B NMR (160 MHz, Chloroform-*d*)

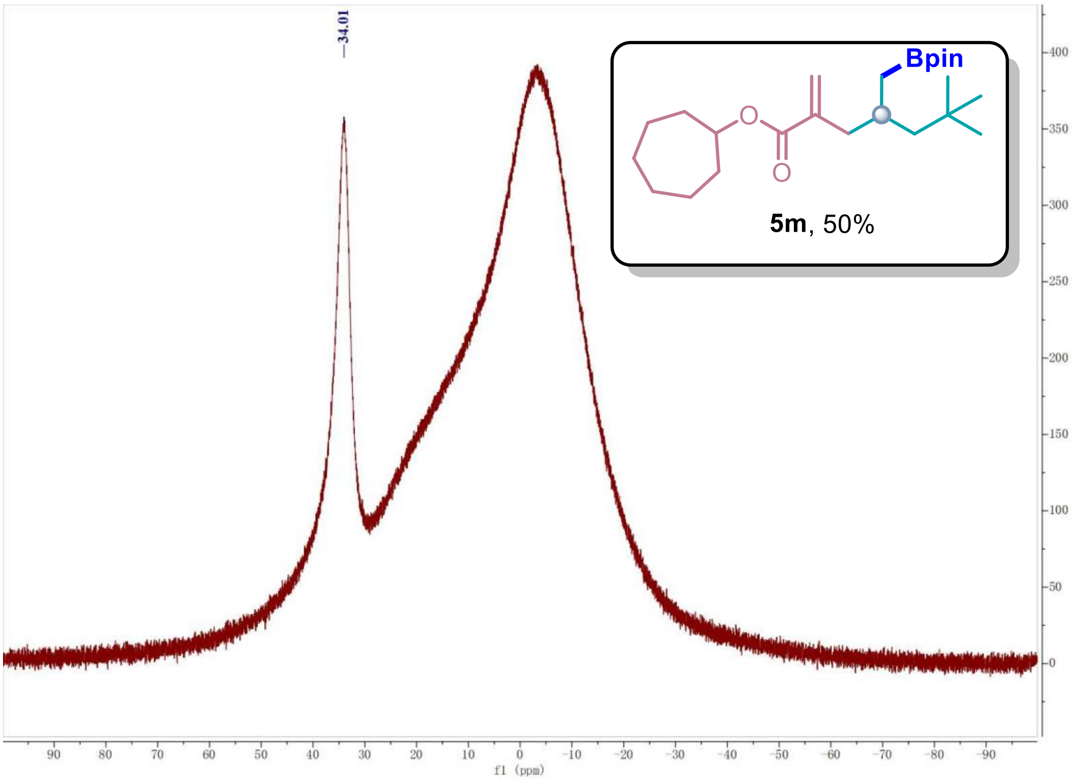

(E)-3,7-dimethylocta-2,6-dien-1-yl 6,6-dimethyl-2-methylene-4-((4,4,5,5-tetramethyl-1,3,2-dioxaborolan-2-yl)methyl)heptanoate (5n)

^{1}H NMR (500 MHz, Chloroform-*d*)

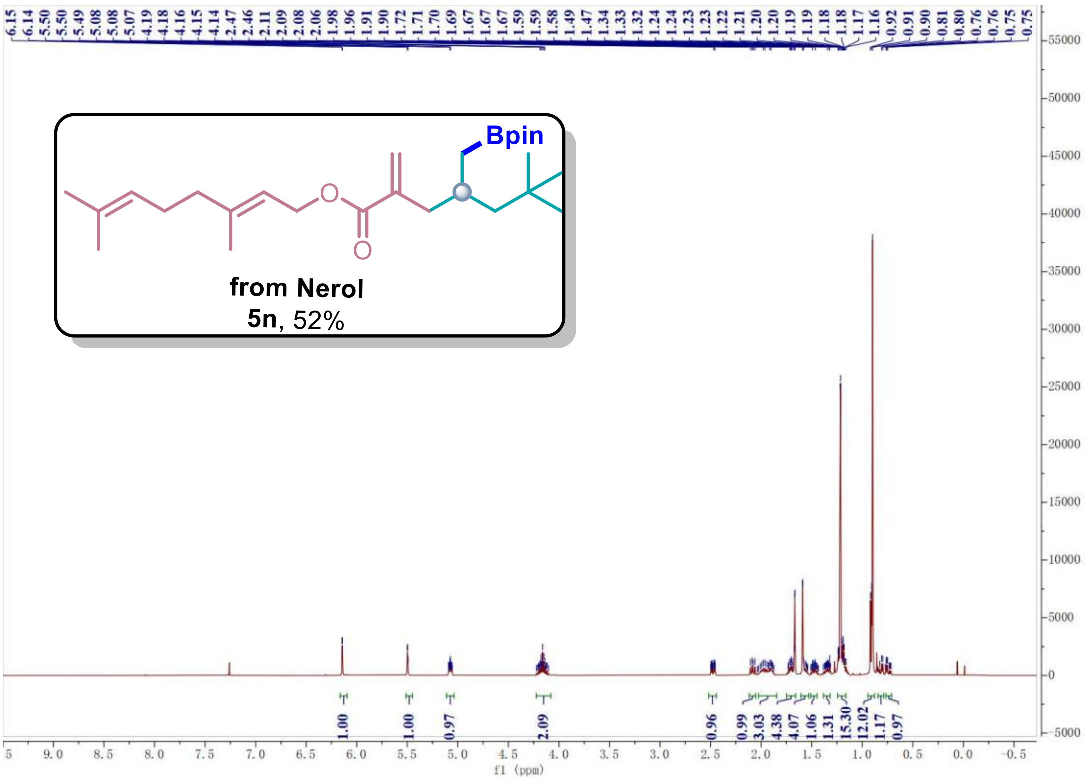

^{13}C NMR (126 MHz, Chloroform-*d*)

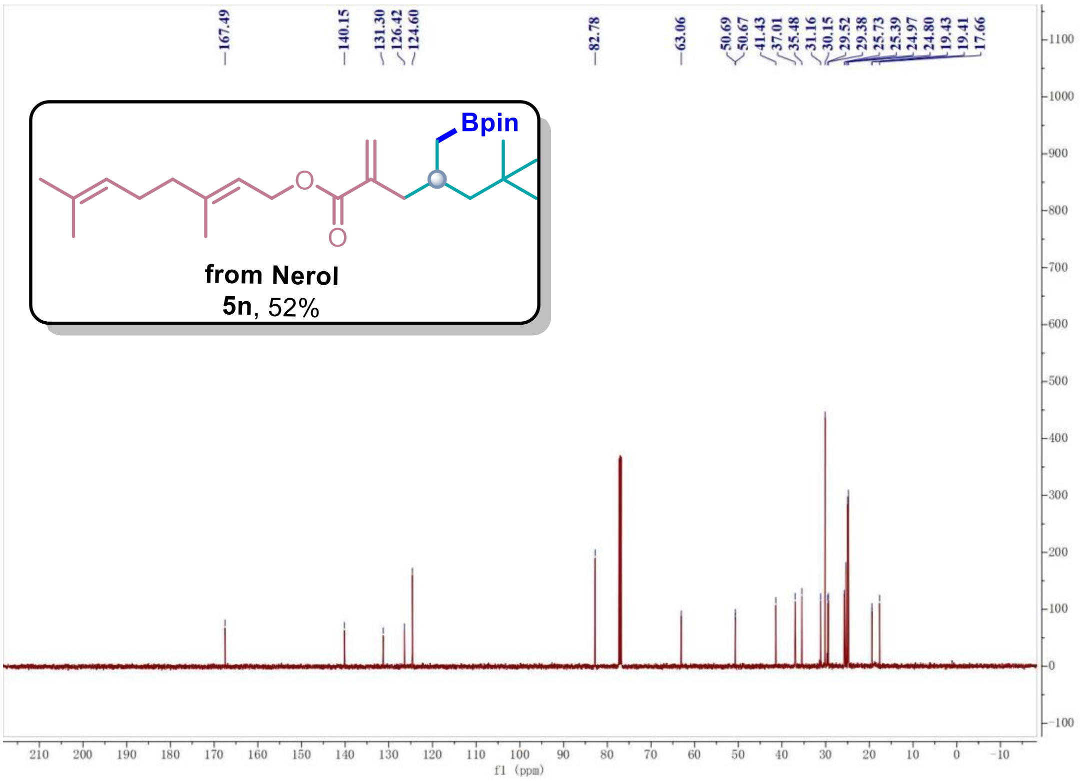

^{11}B NMR (160 MHz, Chloroform-*d*)

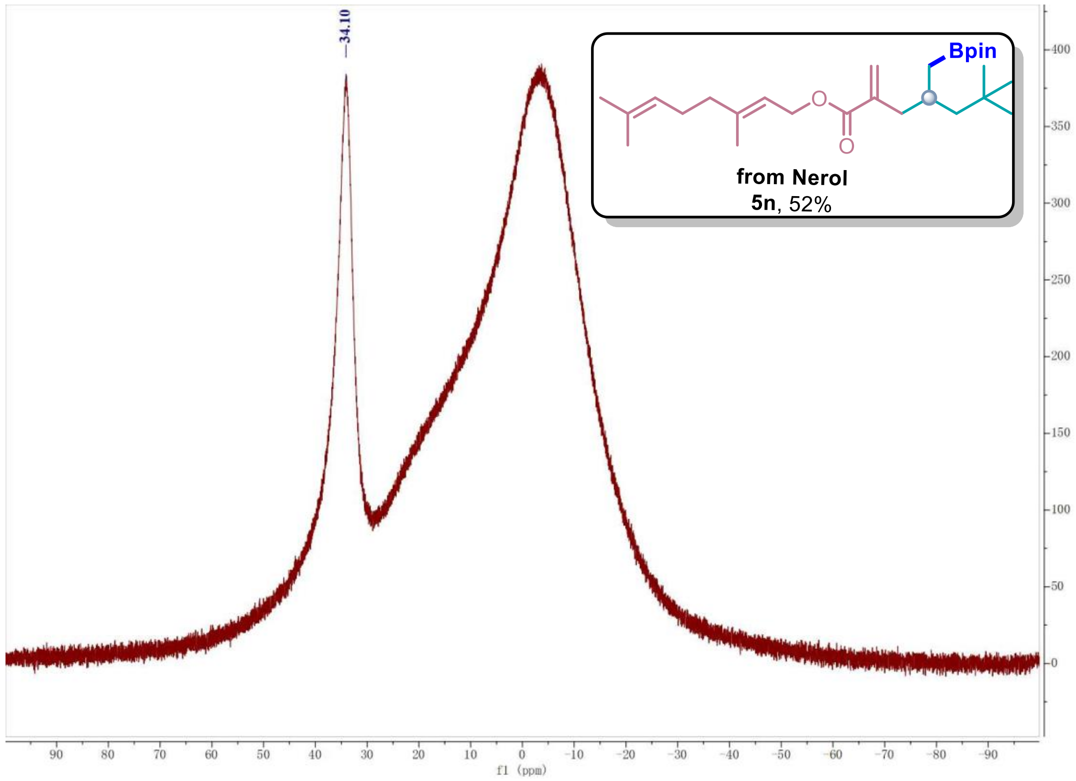

(1R,2S,5R)-2-isopropyl-5-methylcyclohexyl 6,6-dimethyl-2-methylene-4-((4,4,5,5-tetramethyl-1,3,2-dioxaborolan-2-yl)methyl)heptanoate (5o)

^{1}H NMR (500 MHz, Chloroform-*d*)

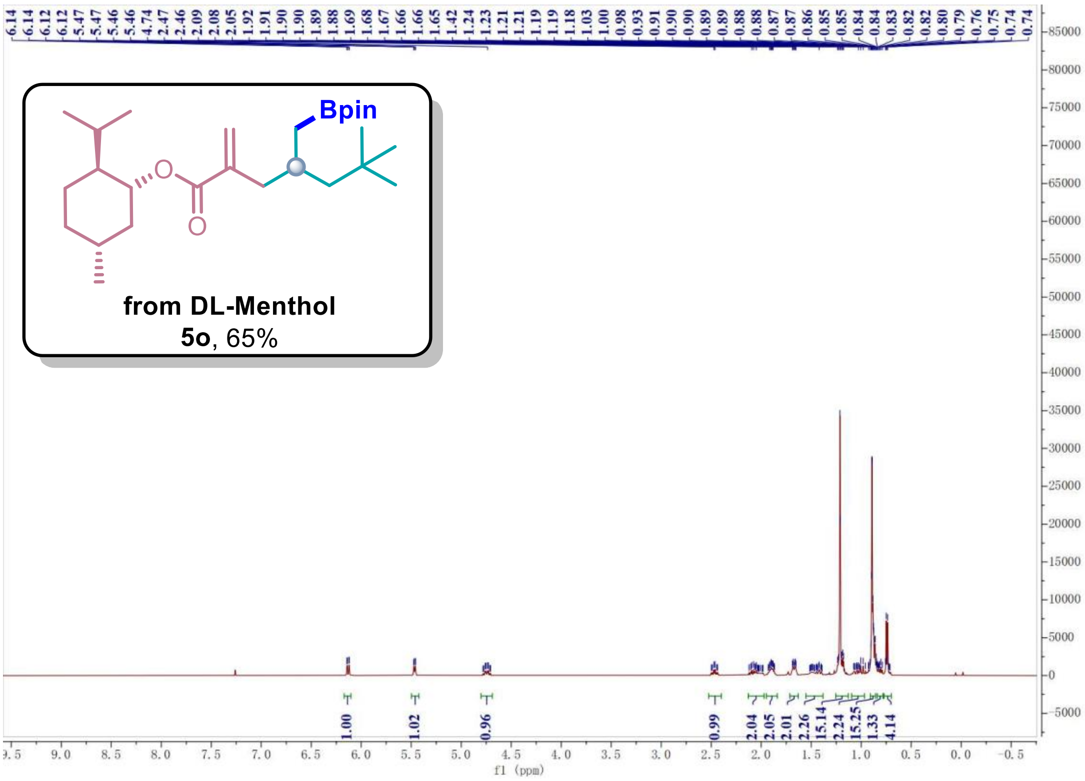

^{13}C NMR (126 MHz, Chloroform-*d*)

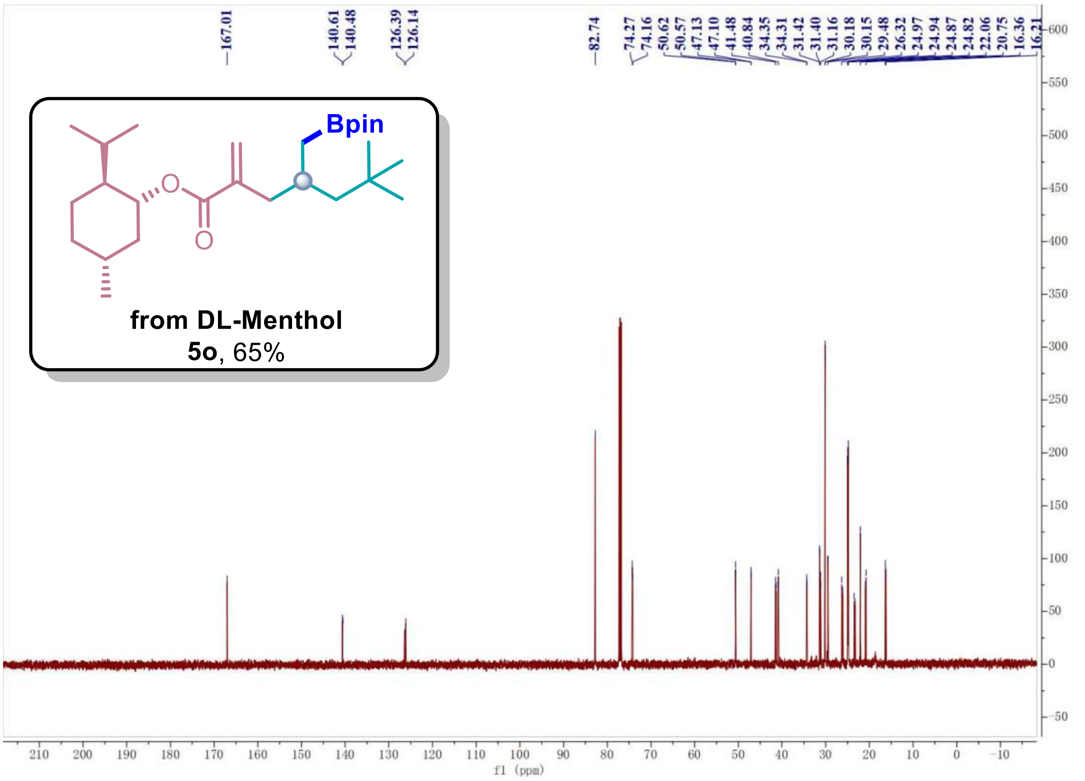

^{11}B NMR (160 MHz, Chloroform-*d*)

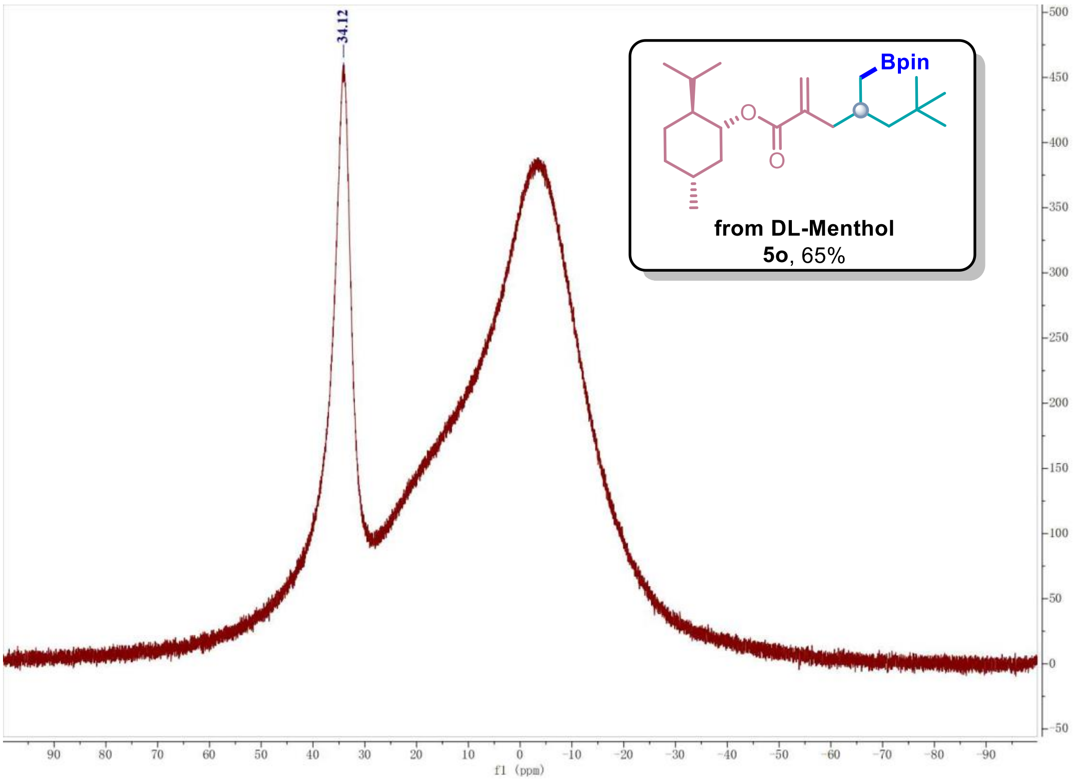

3,7-dimethyloct-6-en-1-yl 6,6-dimethyl-2-methylene-4-((4,4,5,5-tetramethyl-1,3,2-dioxaborolan-2-yl)methyl)heptanoate (5p)

^{1}H NMR (500 MHz, Chloroform-*d*)

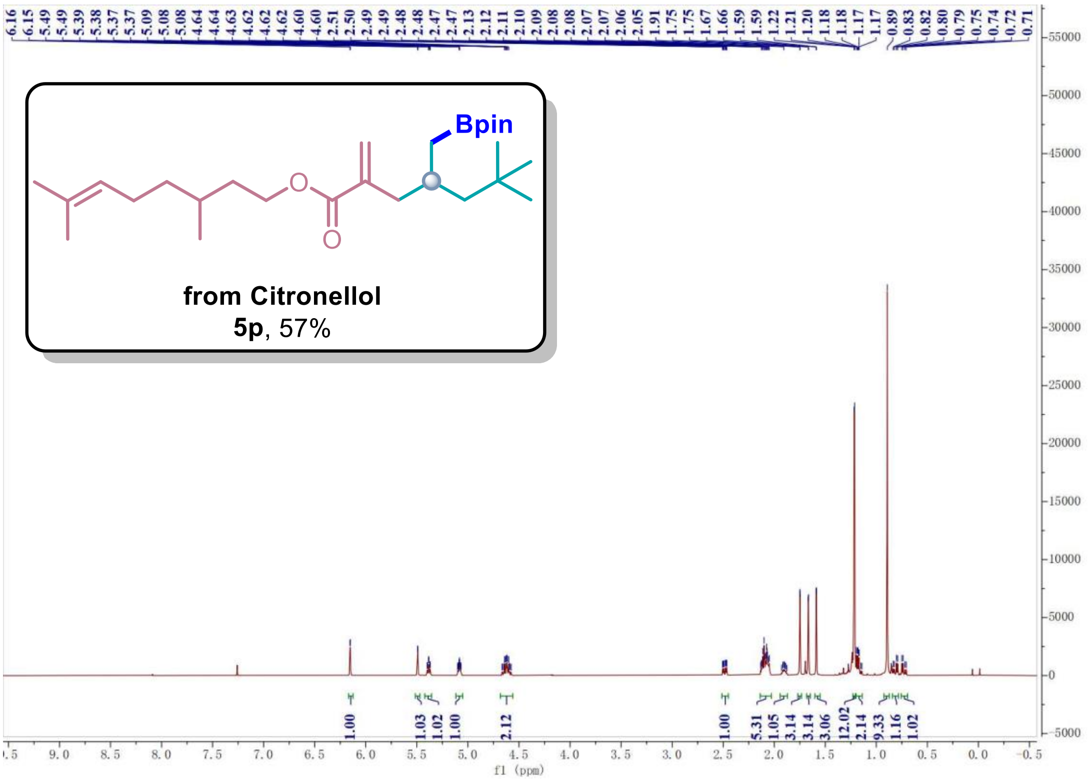

^{13}C NMR (126 MHz, Chloroform-*d*)

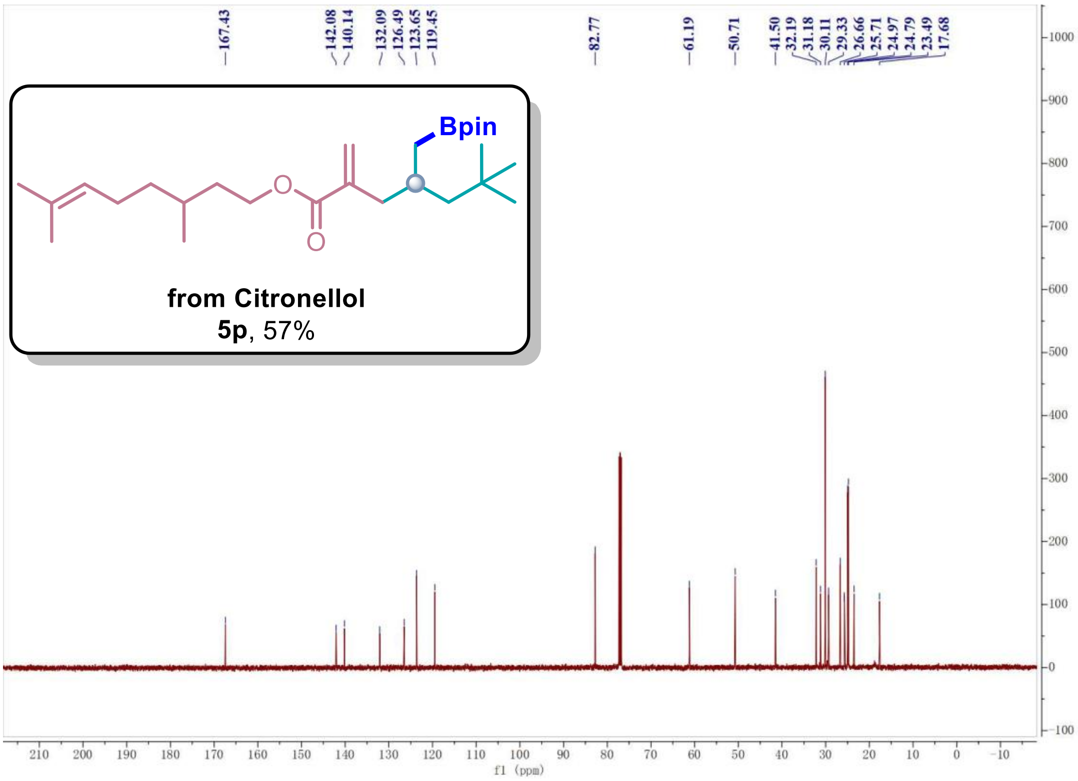

^{11}B NMR (160 MHz, Chloroform-*d*)

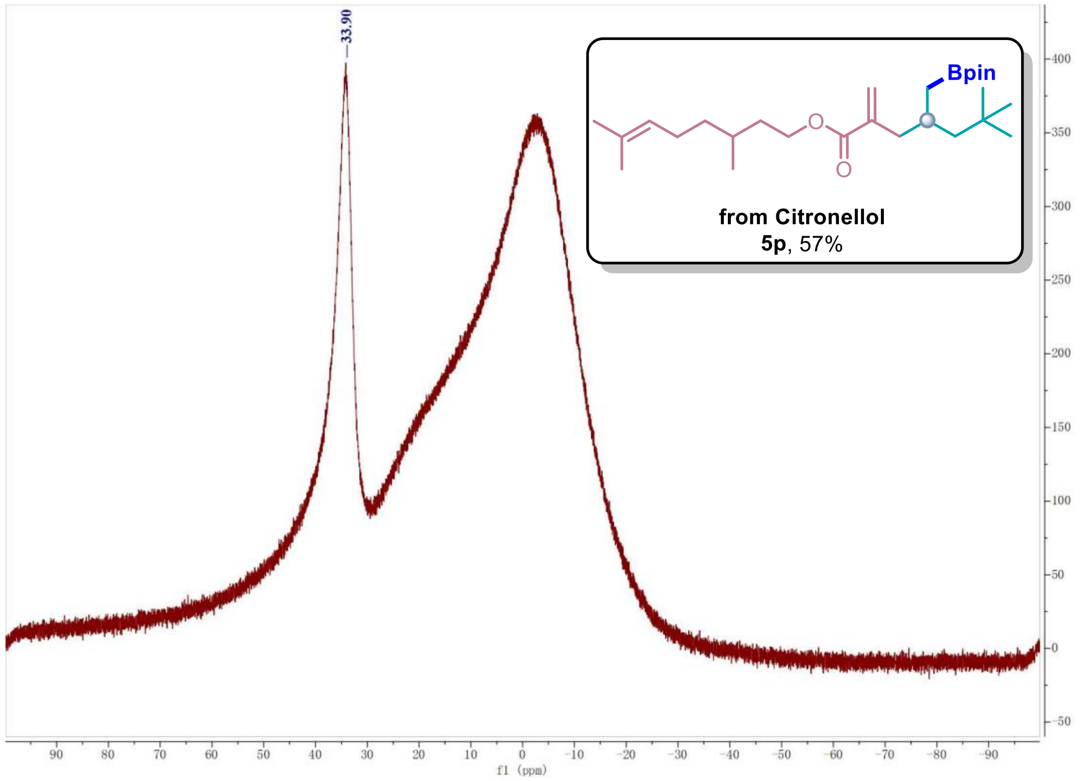

ethyl 4-cyclopentyl-2-methylene-5-(4,4,5,5-tetramethyl-1,3,2-dioxaborolan-2-yl)pentanoate (5q)

^{1}H NMR (500 MHz, Chloroform-*d*)

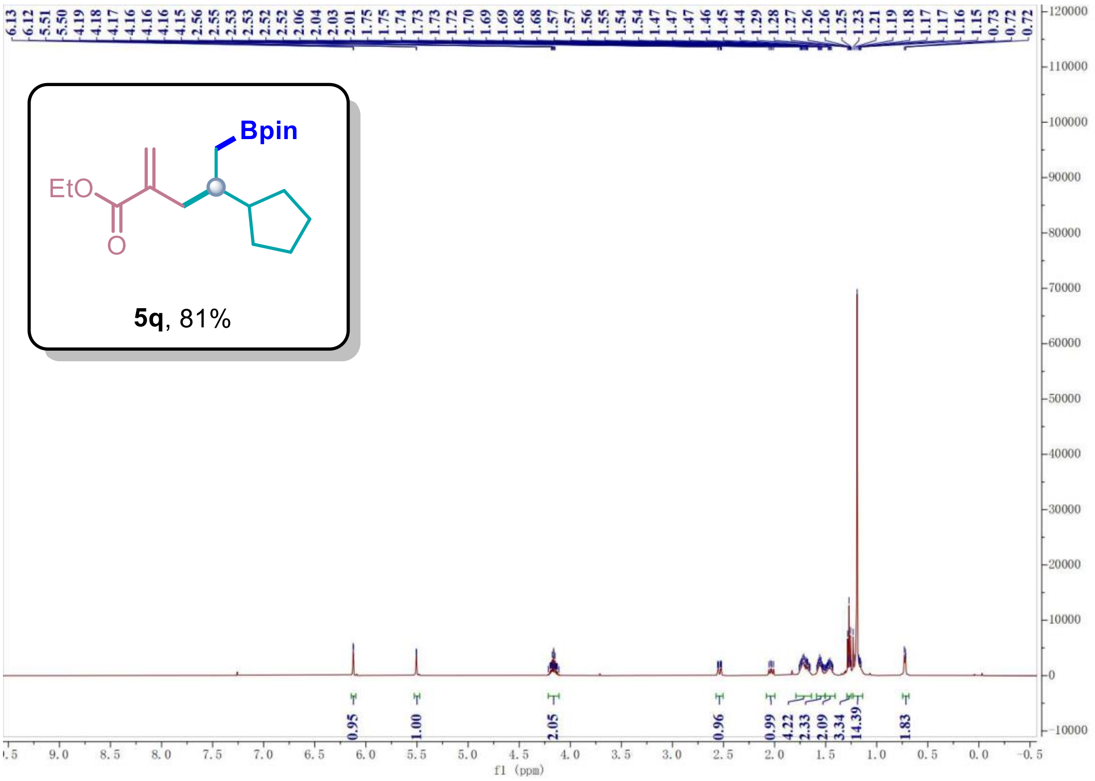

^{13}C NMR (126 MHz, Chloroform-*d*)

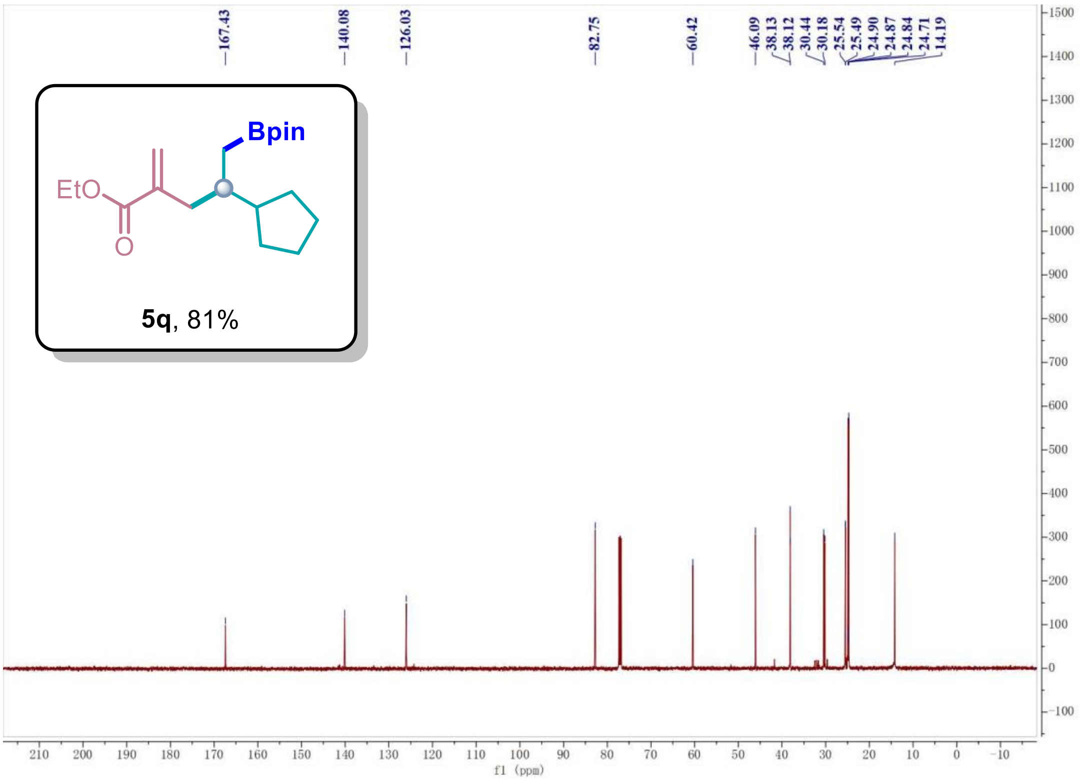

^{11}B NMR (160 MHz, Chloroform-*d*)

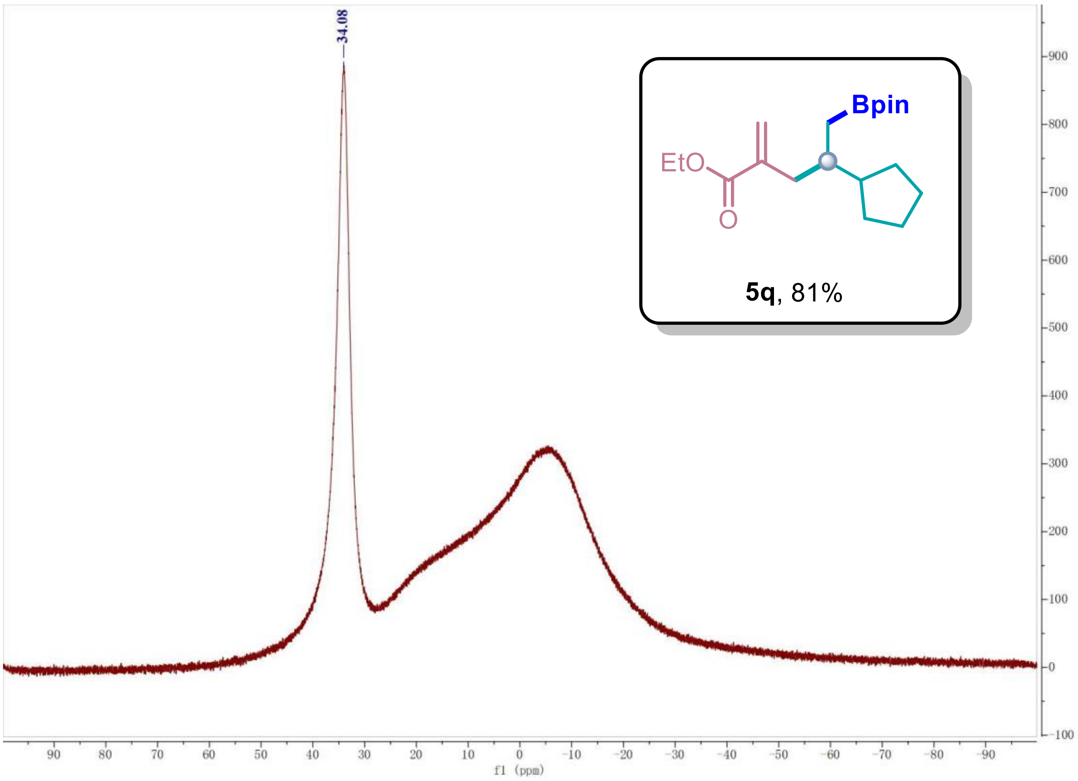

ethyl 4-cyclohexyl-2-methylene-5-(4,4,5,5-tetramethyl-1,3,2-dioxaborolan-2-yl)pentanoate (5r)

^{1}H NMR (500 MHz, Chloroform-*d*)

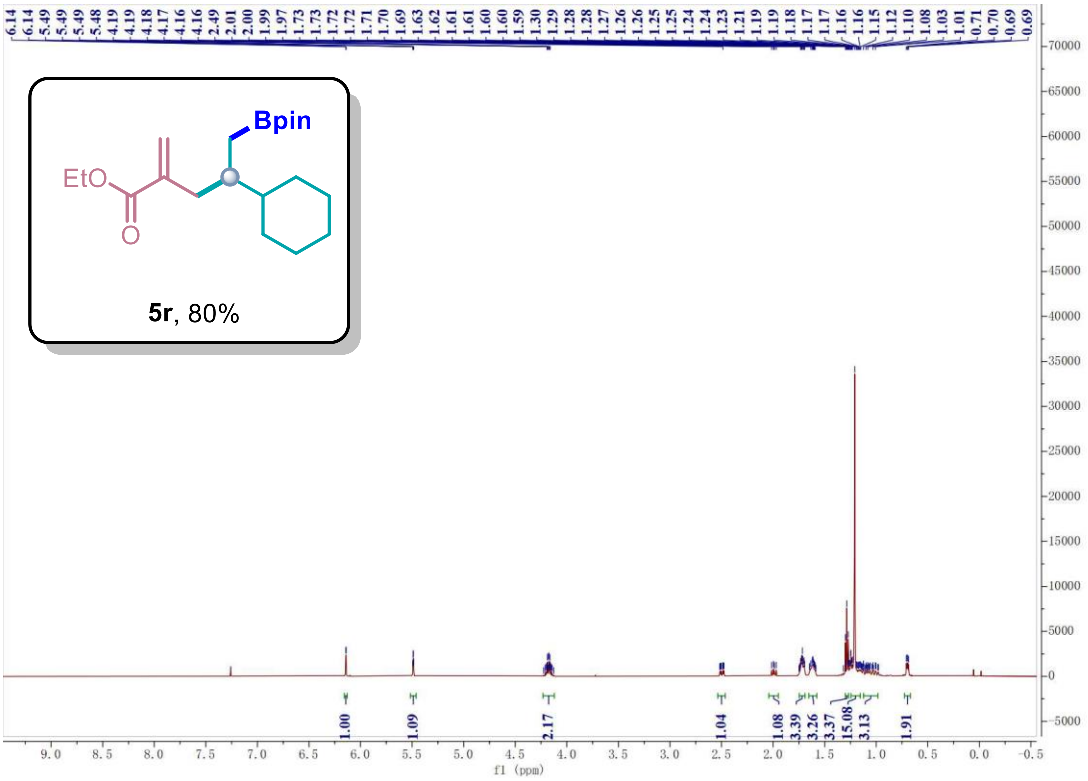

^{13}C NMR (126 MHz, Chloroform-*d*)

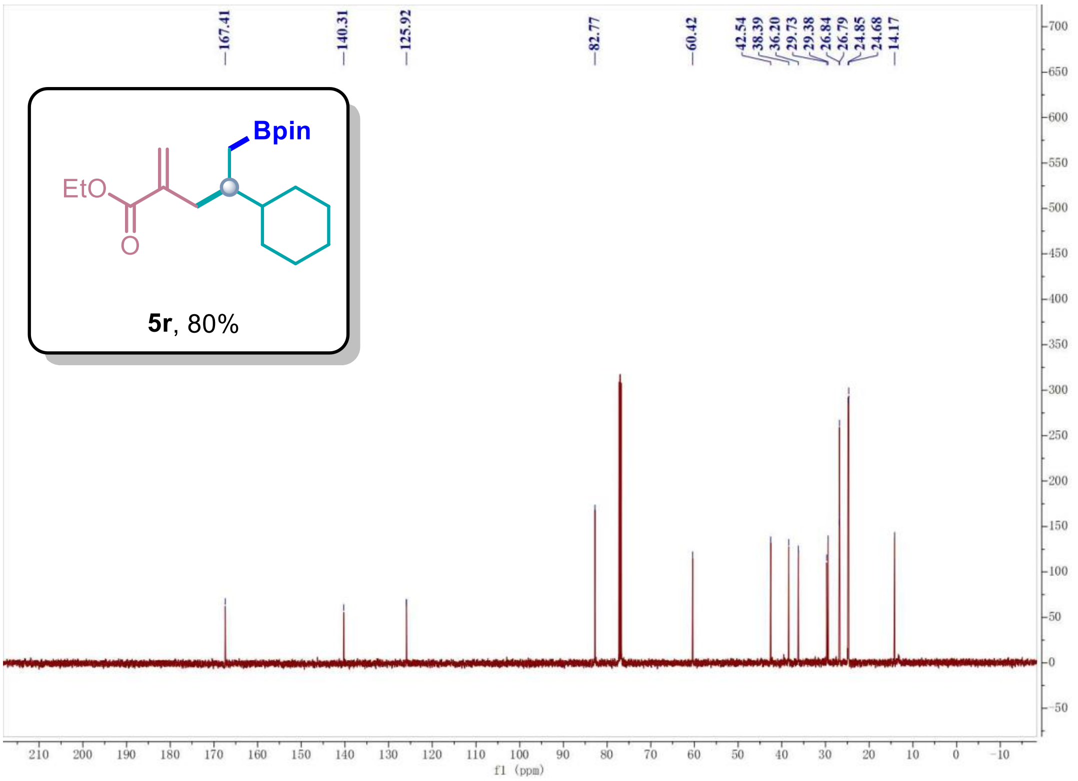

^{11}B NMR (160 MHz, Chloroform-*d*)

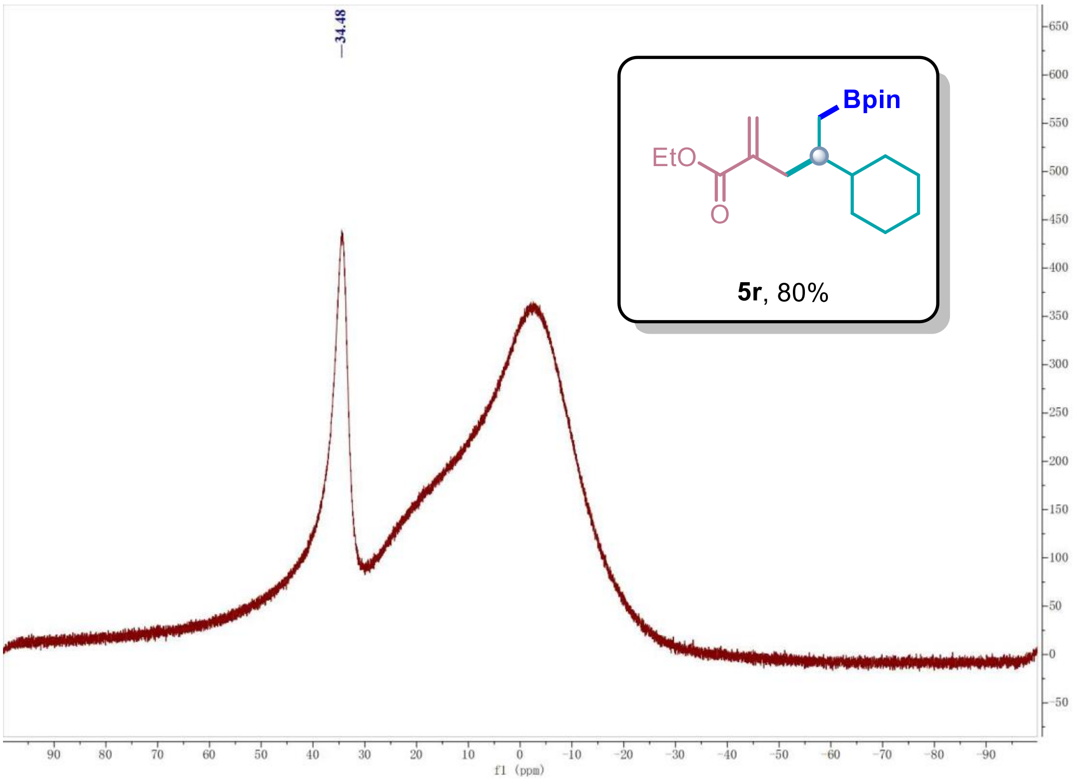

ethyl 5-ethyl-2-methylene-4-((4,4,5,5-tetramethyl-1,3,2-dioxaborolan-2-yl)methyl)heptanoate (5s)

^{1}H NMR (500 MHz, Chloroform-*d*)

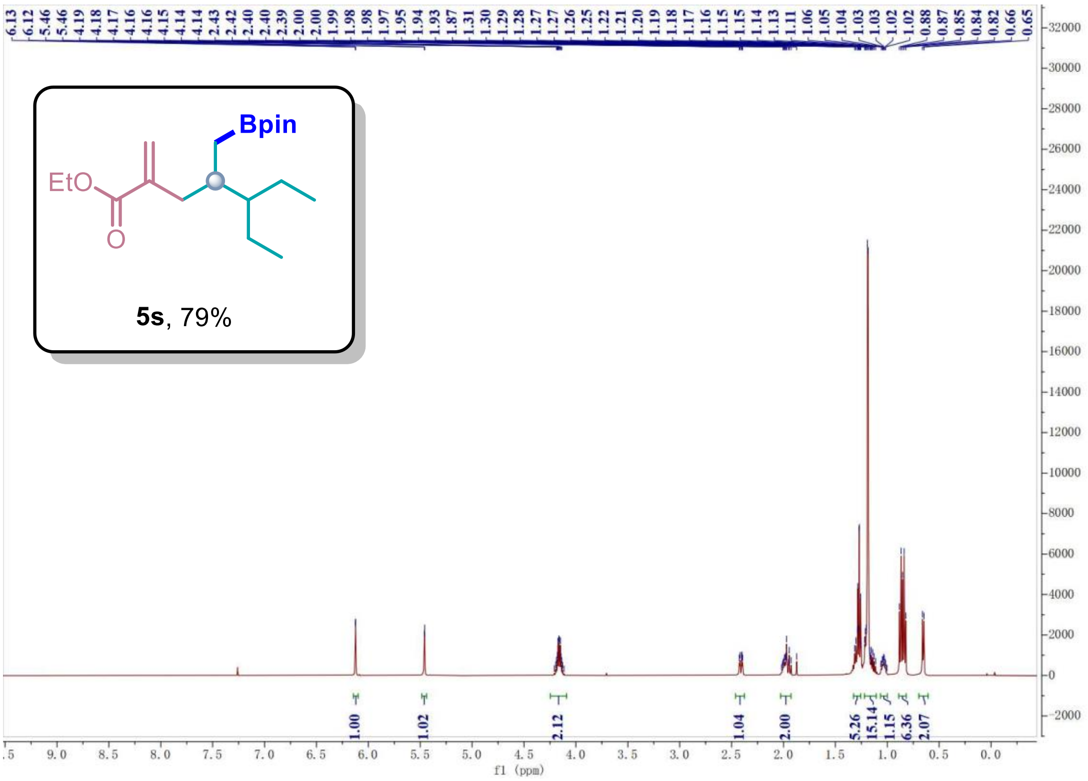

^{13}C NMR (126 MHz, Chloroform-*d*)

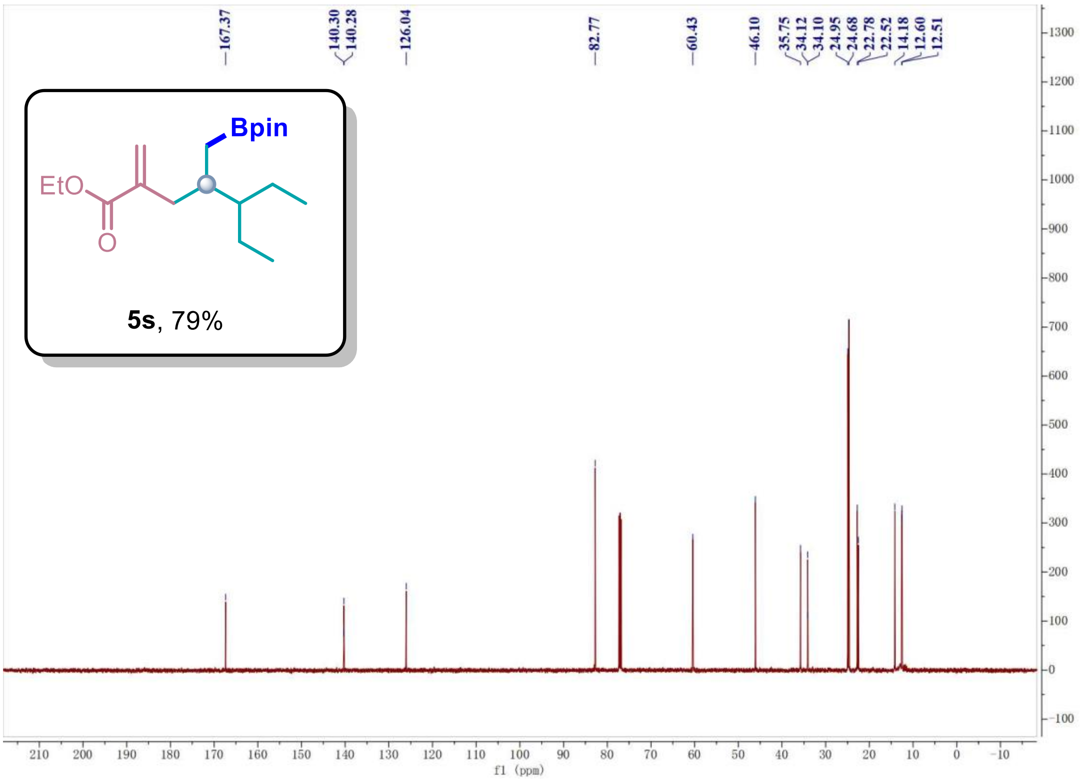

^{11}B NMR (160 MHz, Chloroform-*d*)

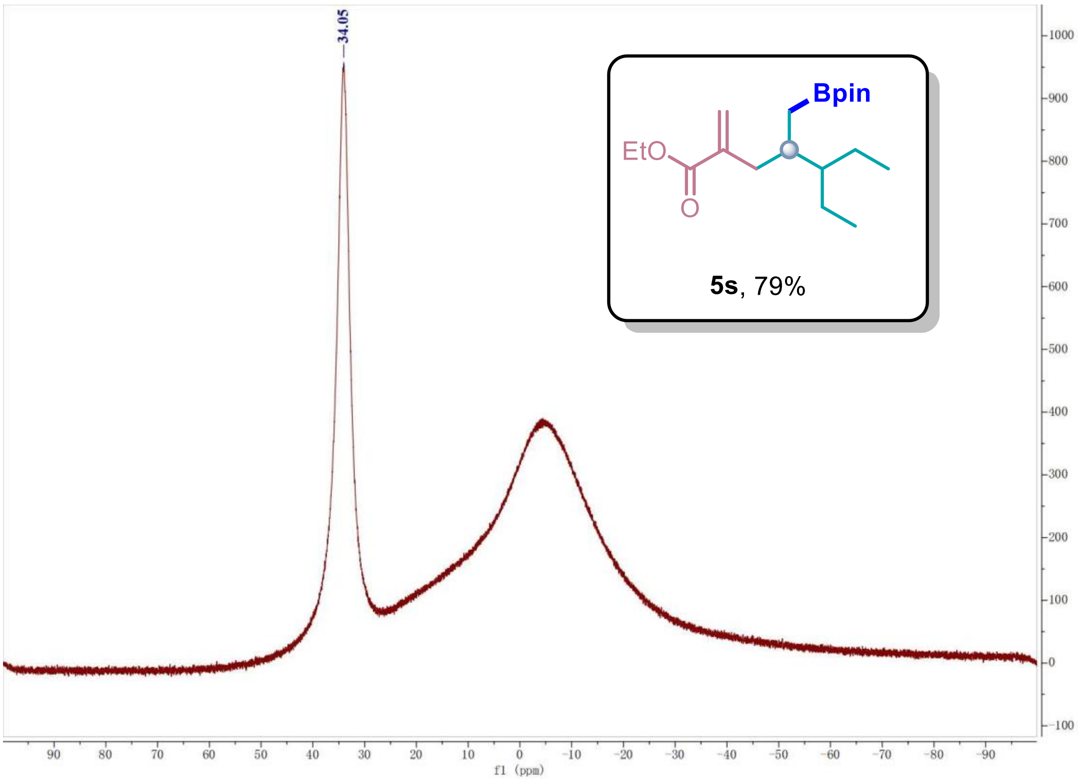

ethyl 5-methyl-2-methylene-4-((4,4,5,5-tetramethyl-1,3,2-dioxaborolan-2-yl)methyl)octanoate (5t)

^{1}H NMR (500 MHz, Chloroform-*d*)

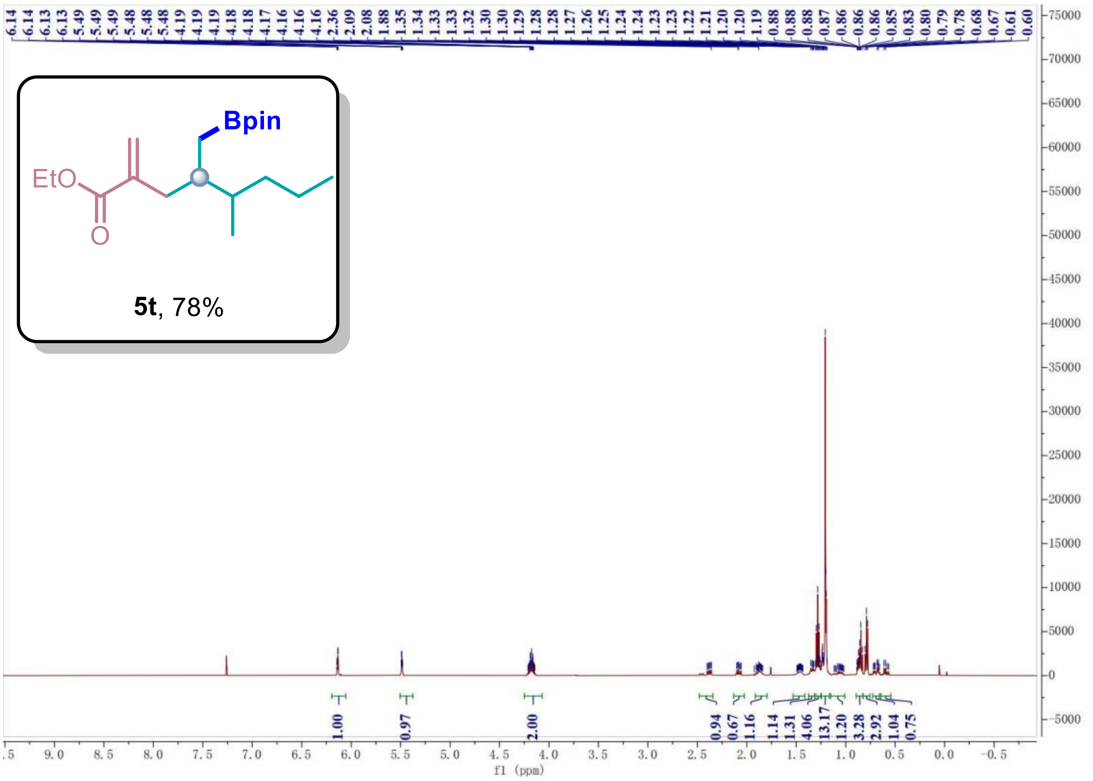

^{13}C NMR (126 MHz, Chloroform-*d*)

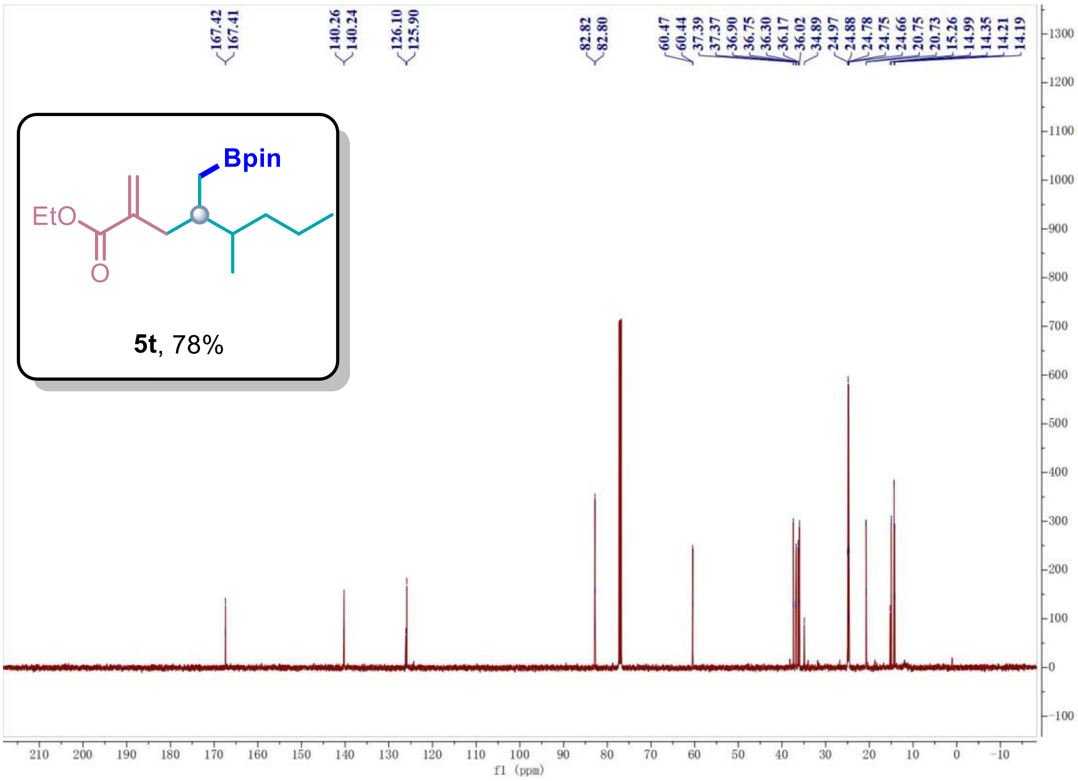

^{11}B NMR (160 MHz, Chloroform-*d*)

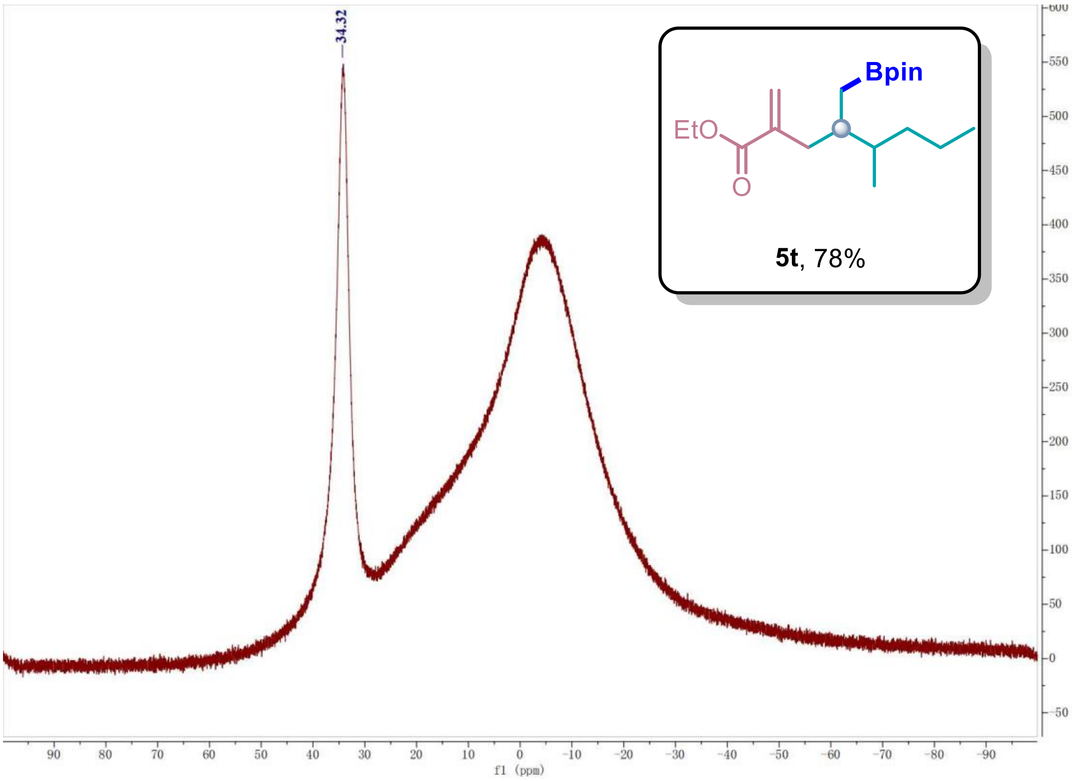

ethyl 4-(cyclohex-2-en-1-yl)-2-methylene-5-(4,4,5,5-tetramethyl-1,3,2-dioxaborolan-2-yl)pentanoate (5u)

^{1}H NMR (500 MHz, Chloroform-*d*)

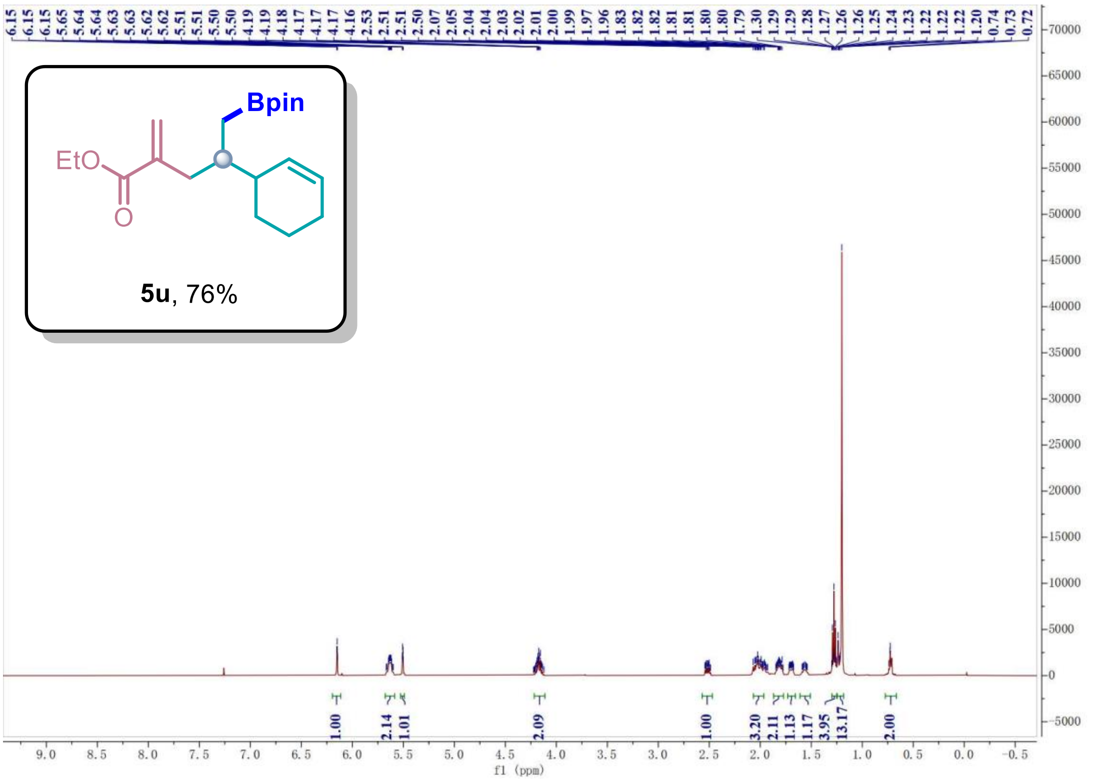

^{13}C NMR (126 MHz, Chloroform-*d*)

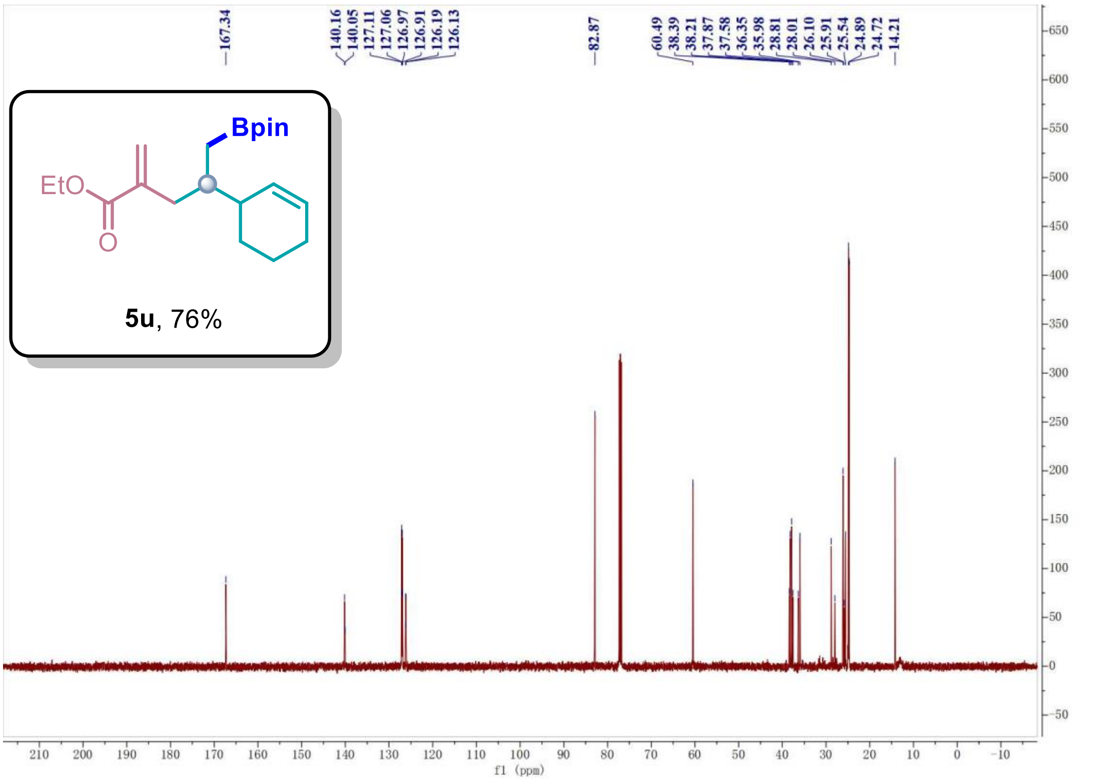

^{11}B NMR (160 MHz, Chloroform-*d*)

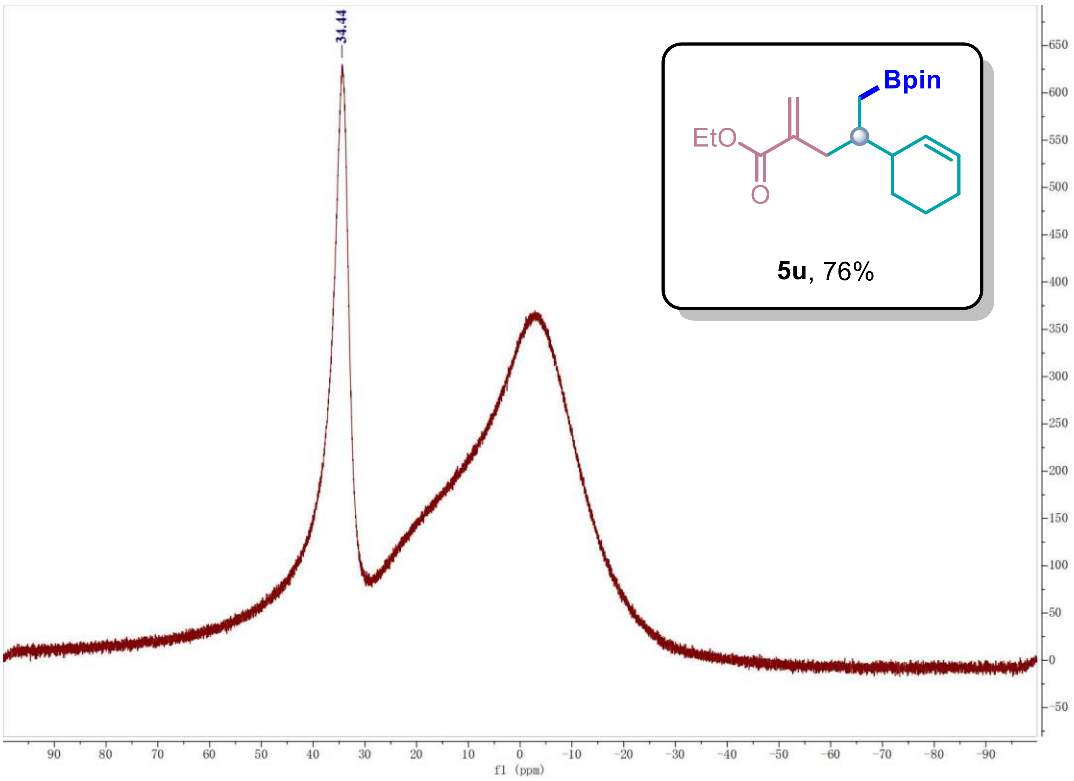

ethyl 4-(3,6-dimethylcyclohex-3-en-1-yl)-2-methylene-5-(4,4,5,5-tetramethyl-1,3,2-dioxaborolan-2-yl)pentanoate (5v)

^{1}H NMR (500 MHz, Chloroform-*d*)

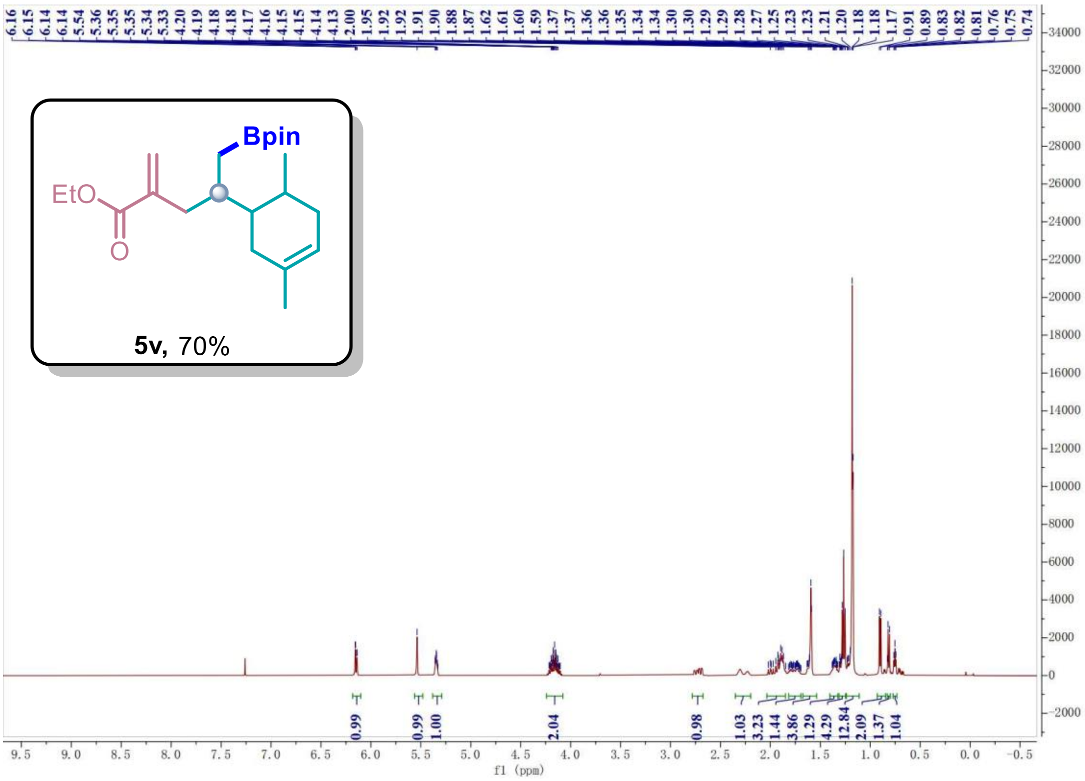

^{13}C NMR (126 MHz, Chloroform-*d*)

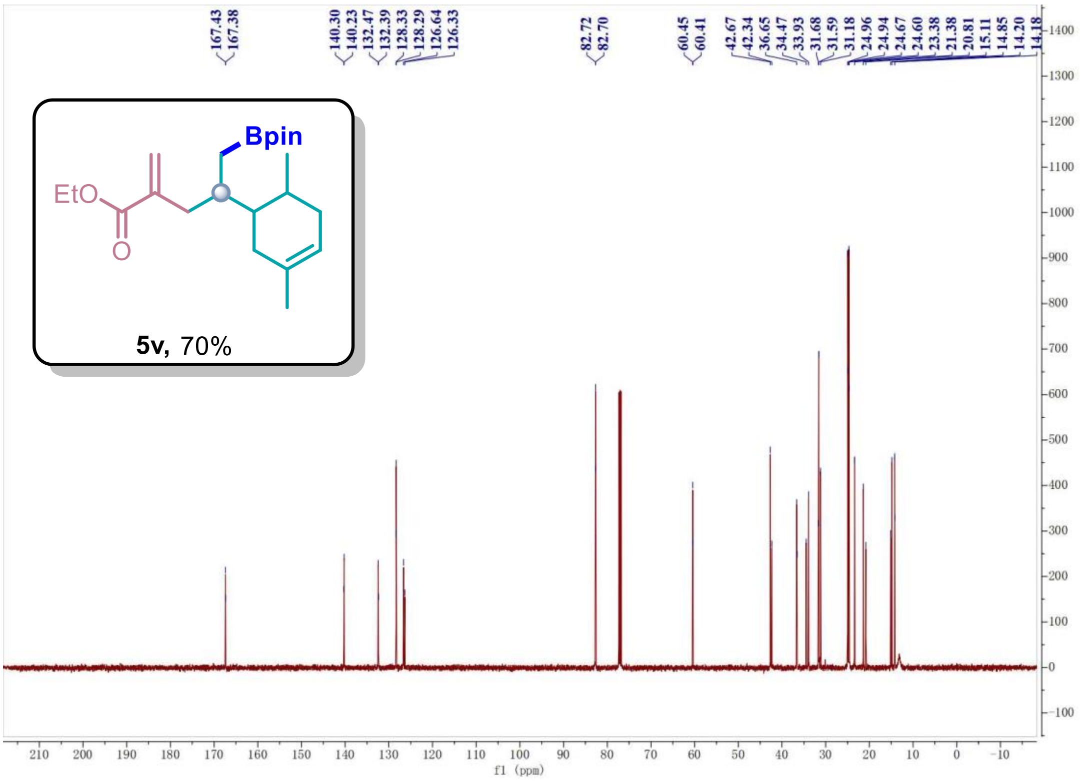

^{11}B NMR (160 MHz, Chloroform-*d*)

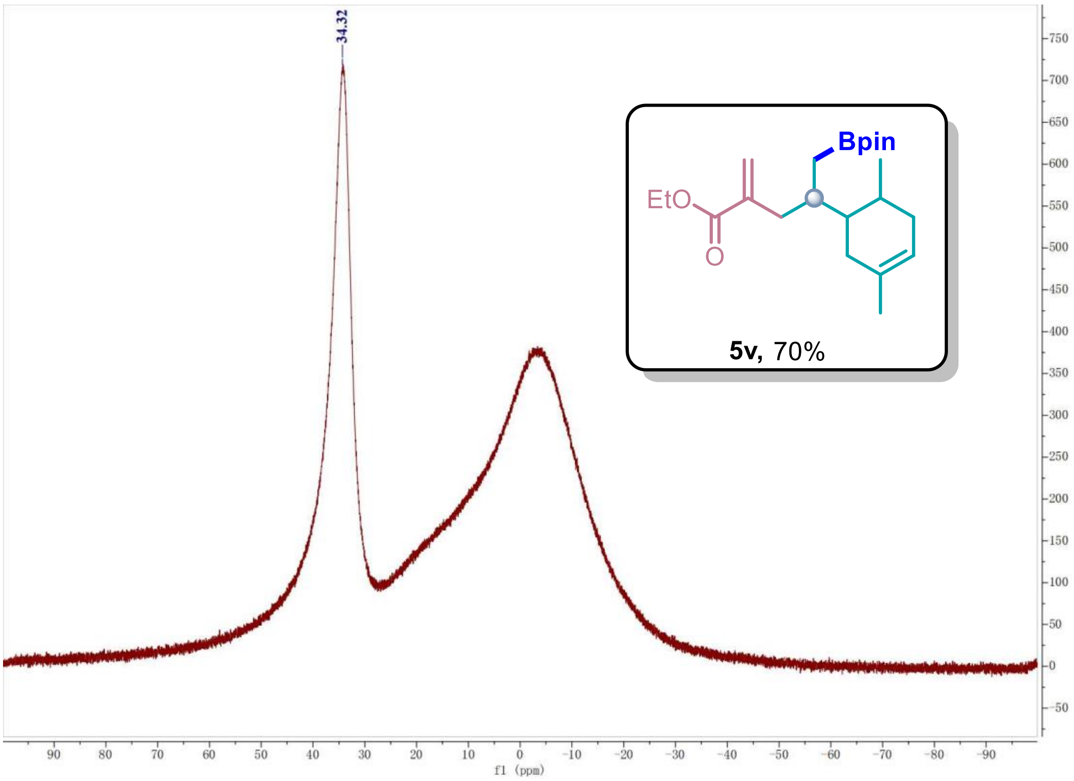

ethyl 2-methylene-6-phenyl-4-((4,4,5,5-tetramethyl-1,3,2-dioxaborolan-2-yl)methyl)hexanoate (5w)

^{1}H NMR (500 MHz, Chloroform-*d*)

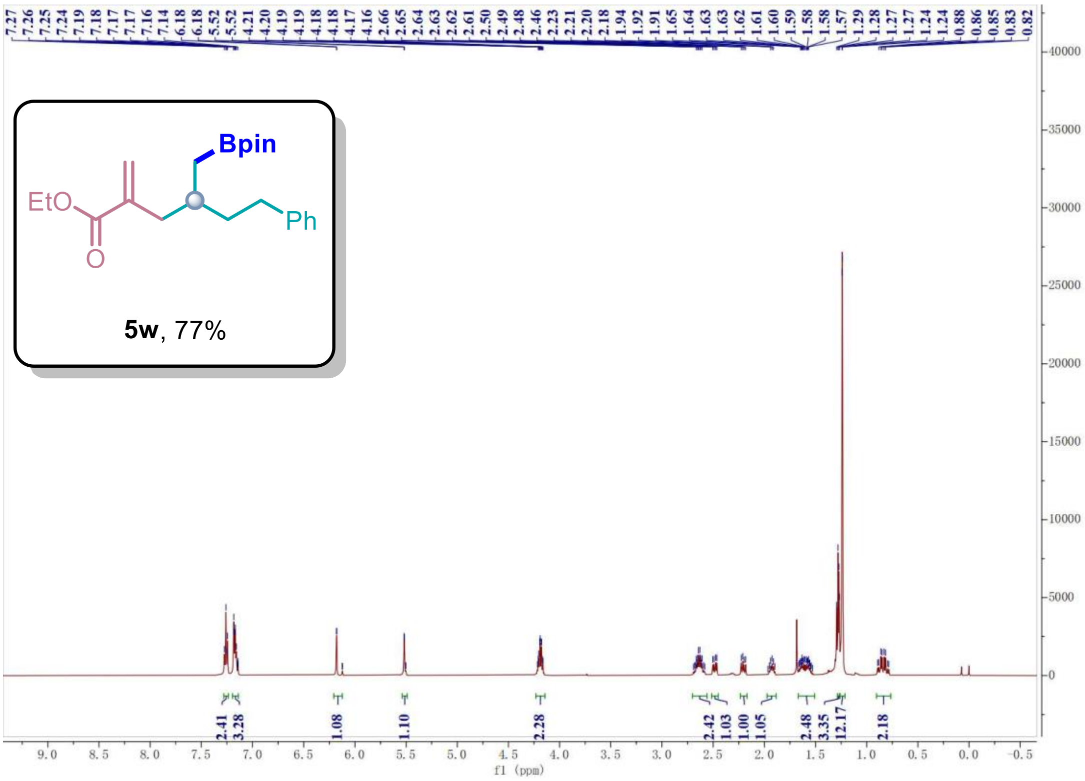

^{13}C NMR (126 MHz, Chloroform-*d*)

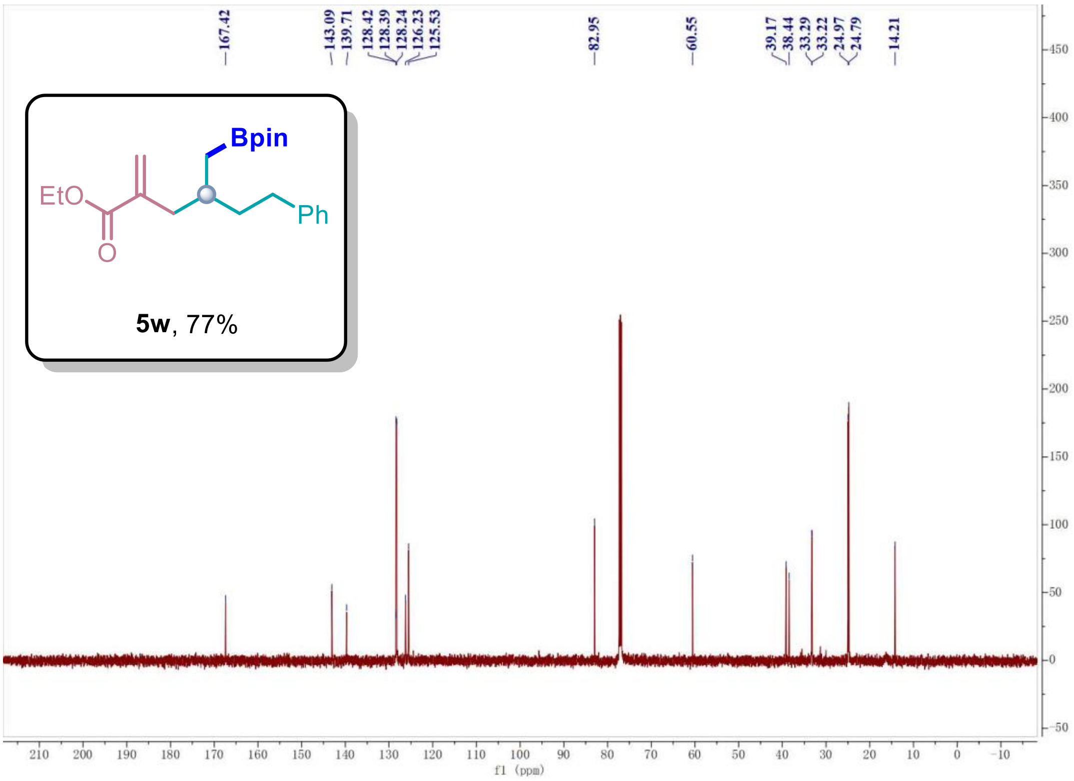

^{11}B NMR (160 MHz, Chloroform-*d*)

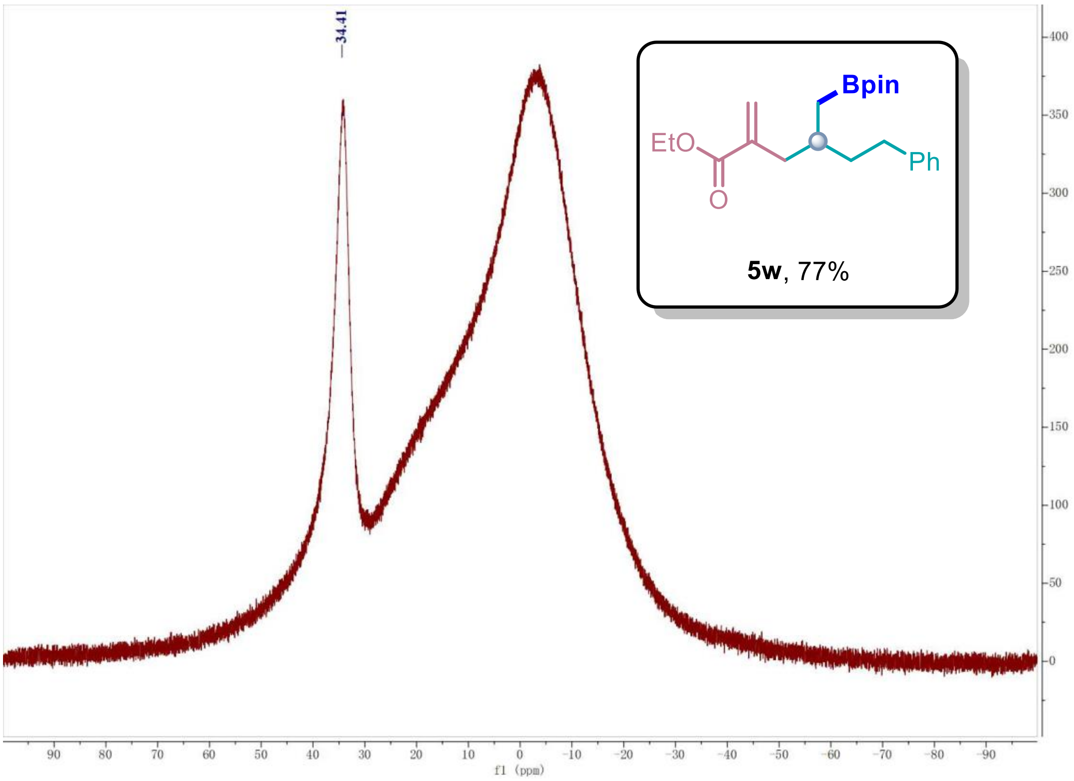

ethyl 6-methyl-2-methylene-4-((4,4,5,5-tetramethyl-1,3,2-dioxaborolan-2-yl)methyl)heptanoate (5x)

^{1}H NMR (500 MHz, Chloroform-*d*)

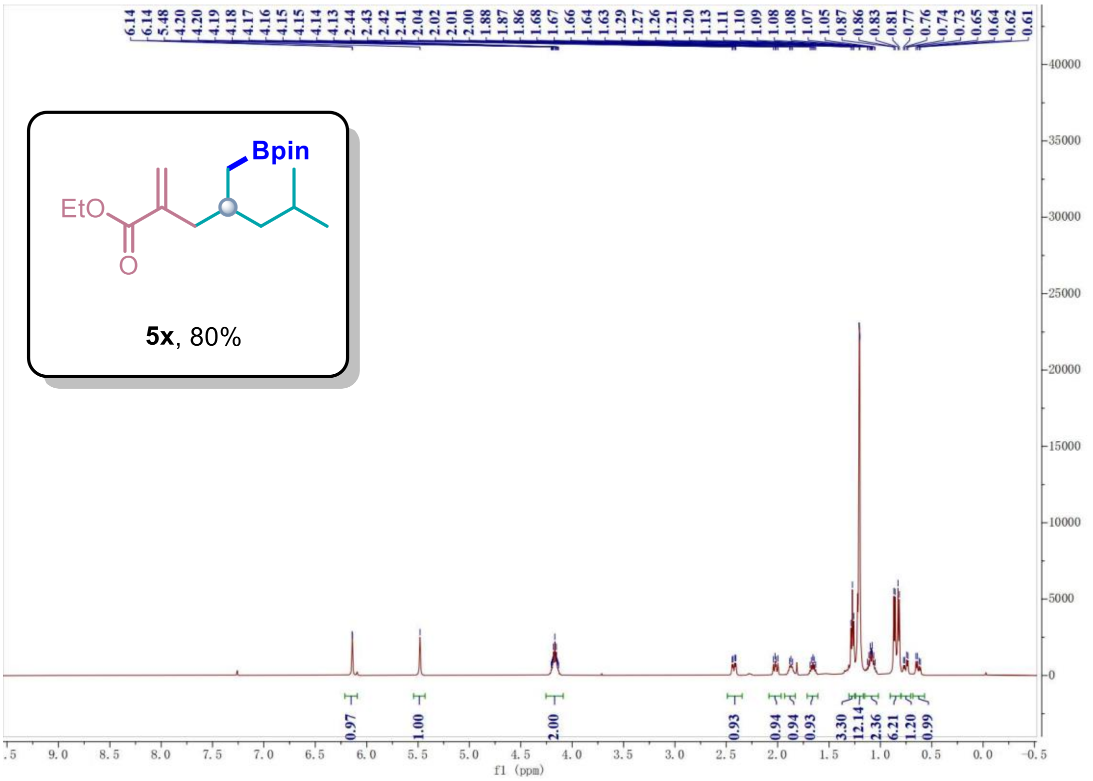

^{13}C NMR (126 MHz, Chloroform-*d*)

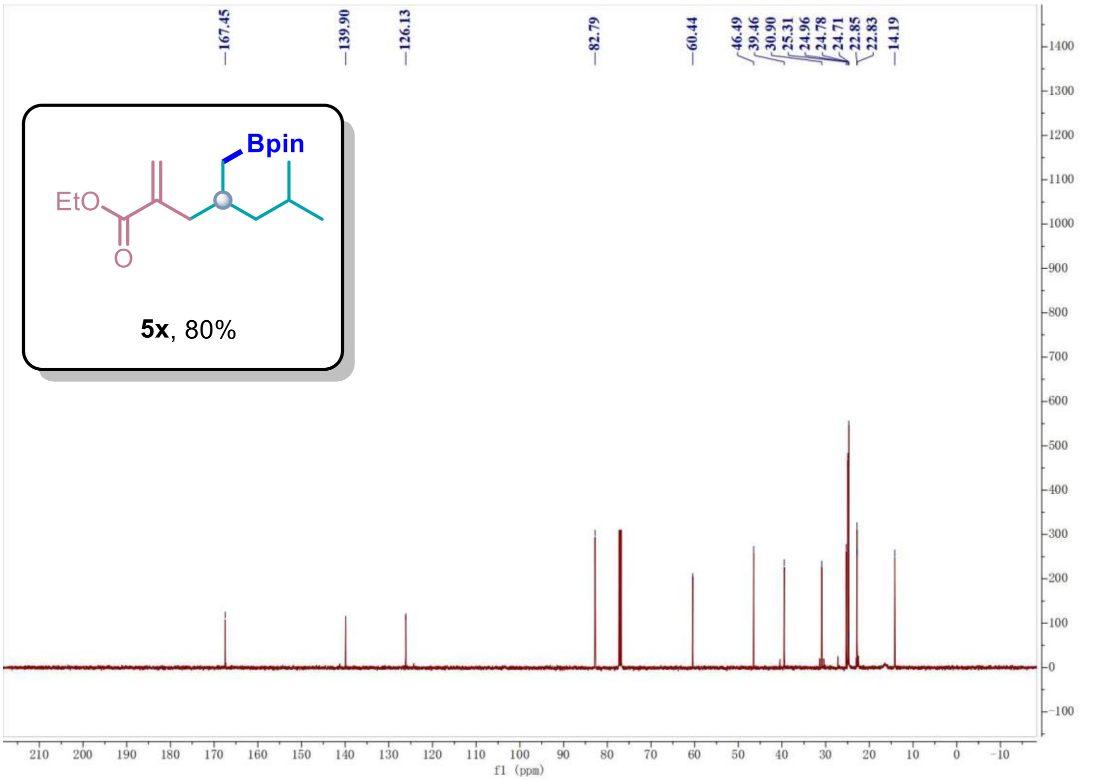

^{11}B NMR (160 MHz, Chloroform-*d*)

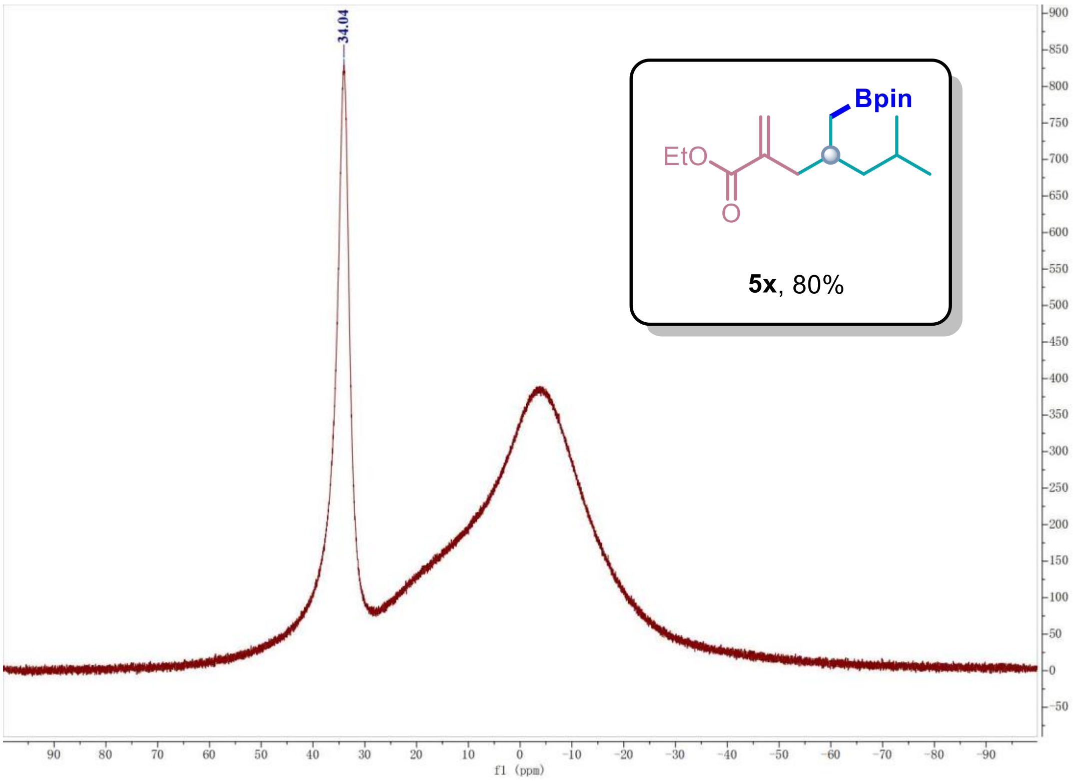

ethyl 2-methylene-4-((4,4,5,5-tetramethyl-1,3,2-dioxaborolan-2-yl)methyl)decanoate (5y)

^{1}H NMR (500 MHz, Chloroform-*d*)

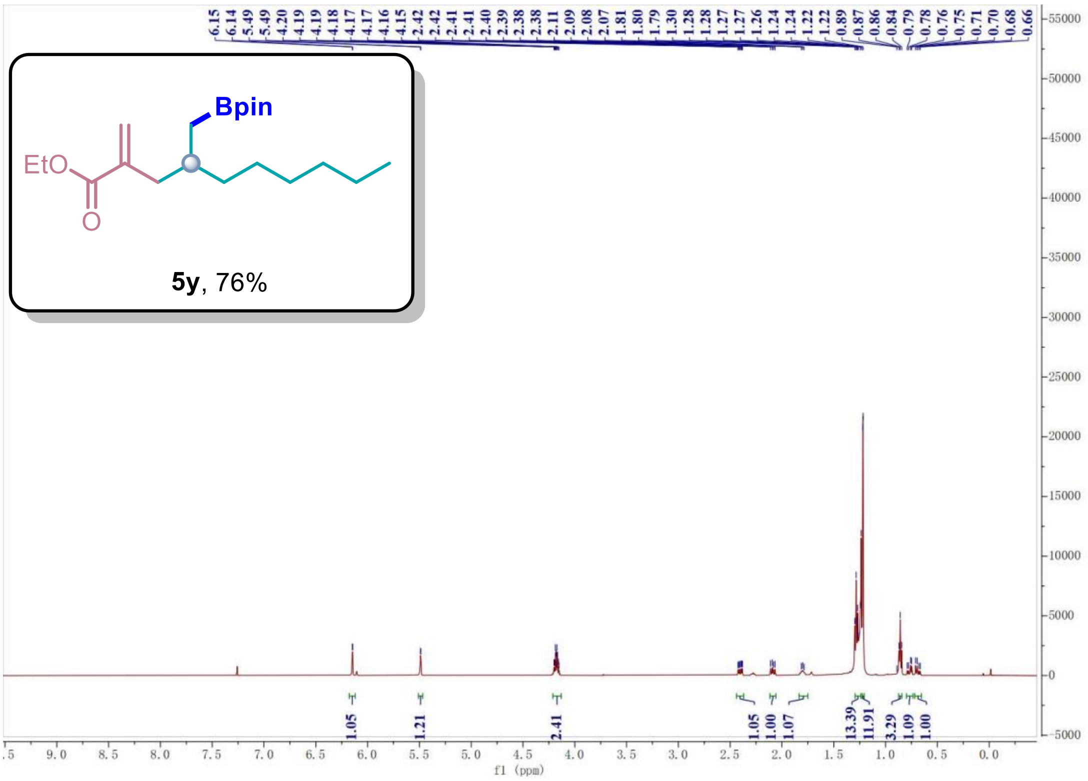

^{13}C NMR (126 MHz, Chloroform-*d*)

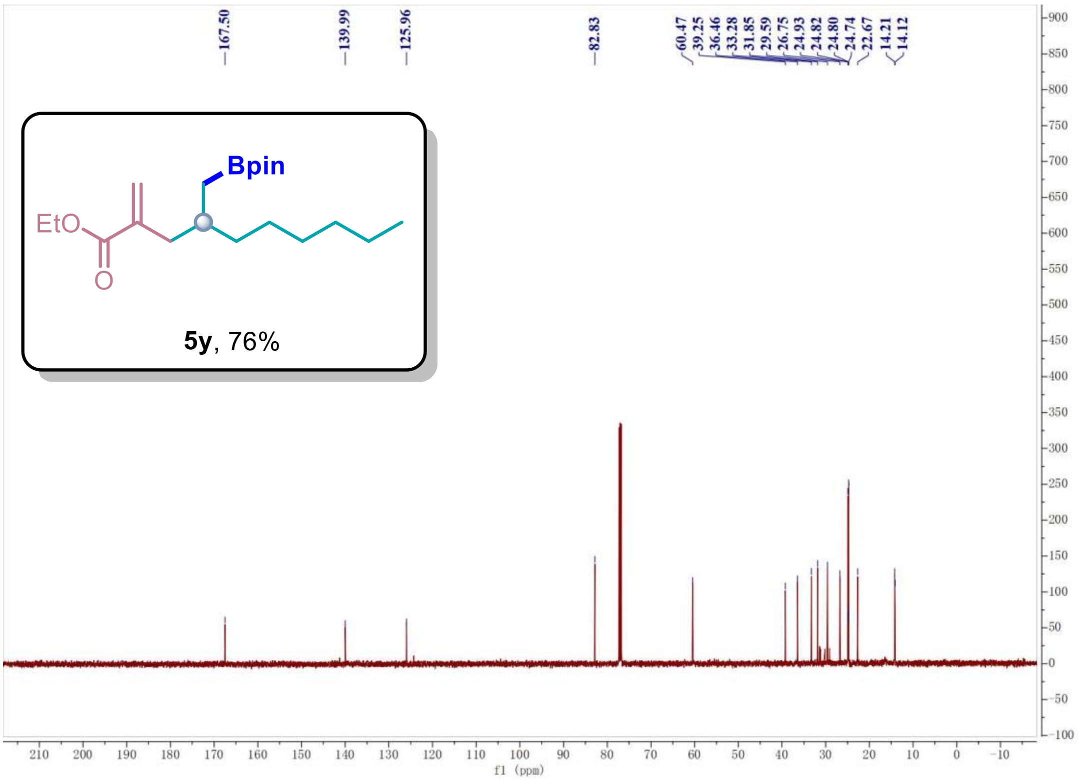

^{11}B NMR (160 MHz, Chloroform-*d*)

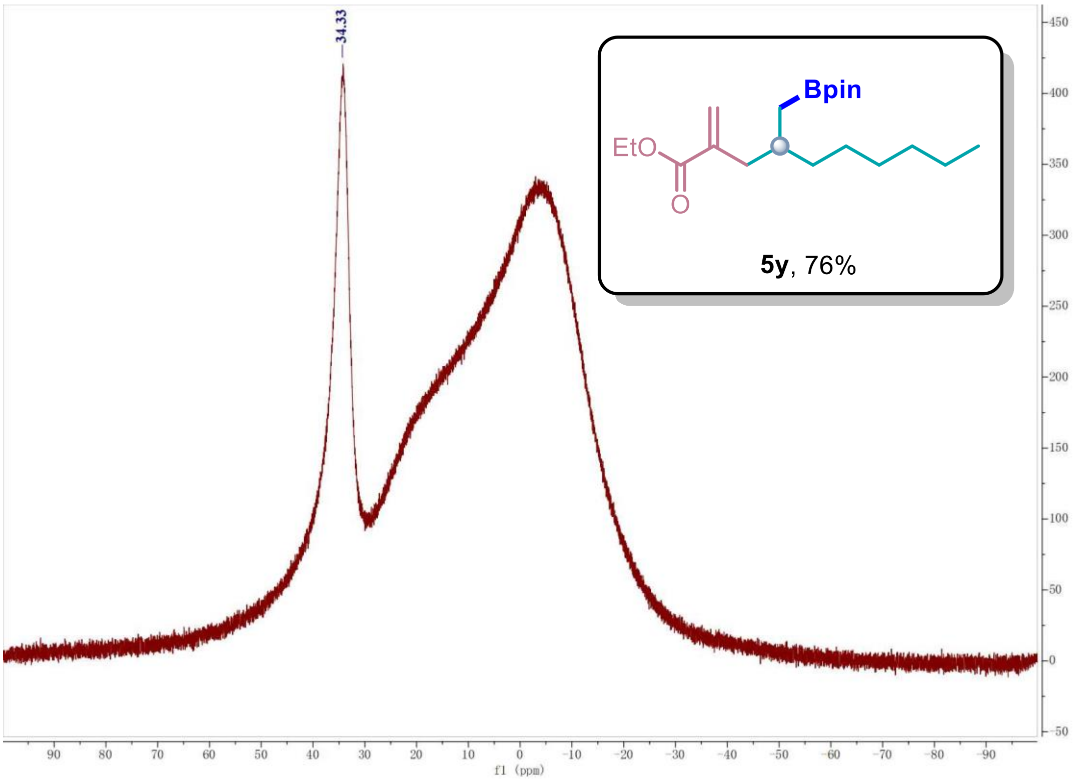

ethyl 2-methylene-4-((4,4,5,5-tetramethyl-1,3,2-dioxaborolan-2-yl)methyl)heptanoate (5z)

^{1}H NMR (500 MHz, Chloroform-*d*)

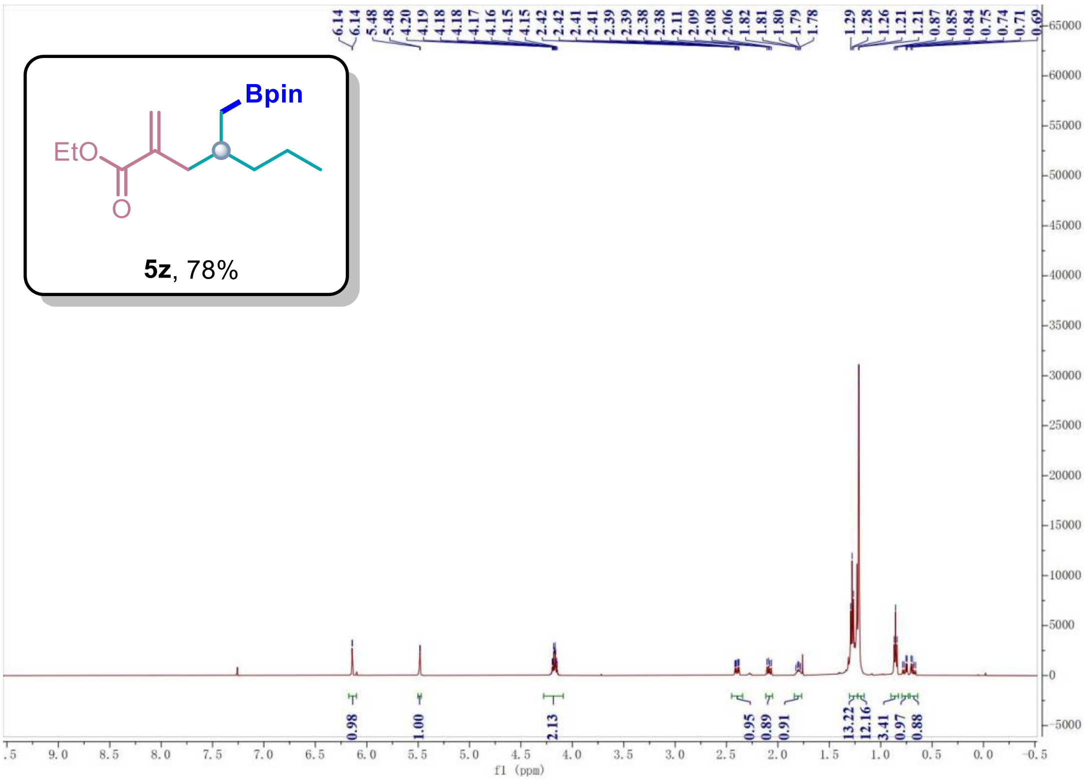

^{13}C NMR (126 MHz, Chloroform-*d*)

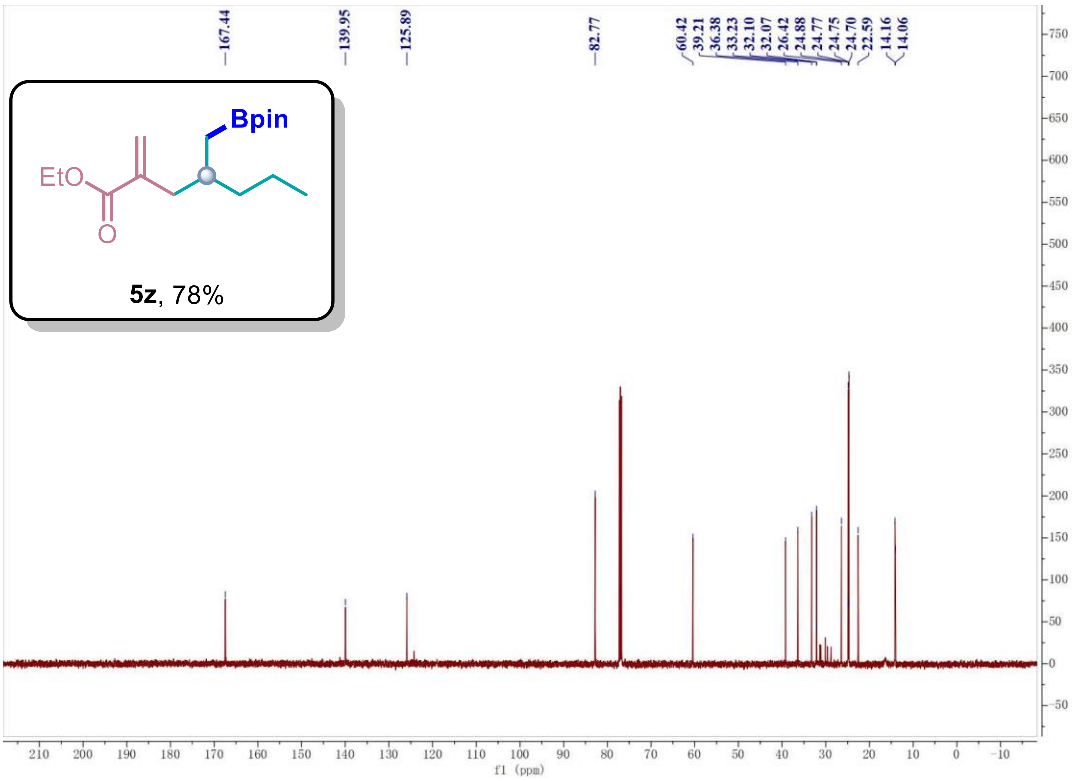

^{11}B NMR (160 MHz, Chloroform-*d*)

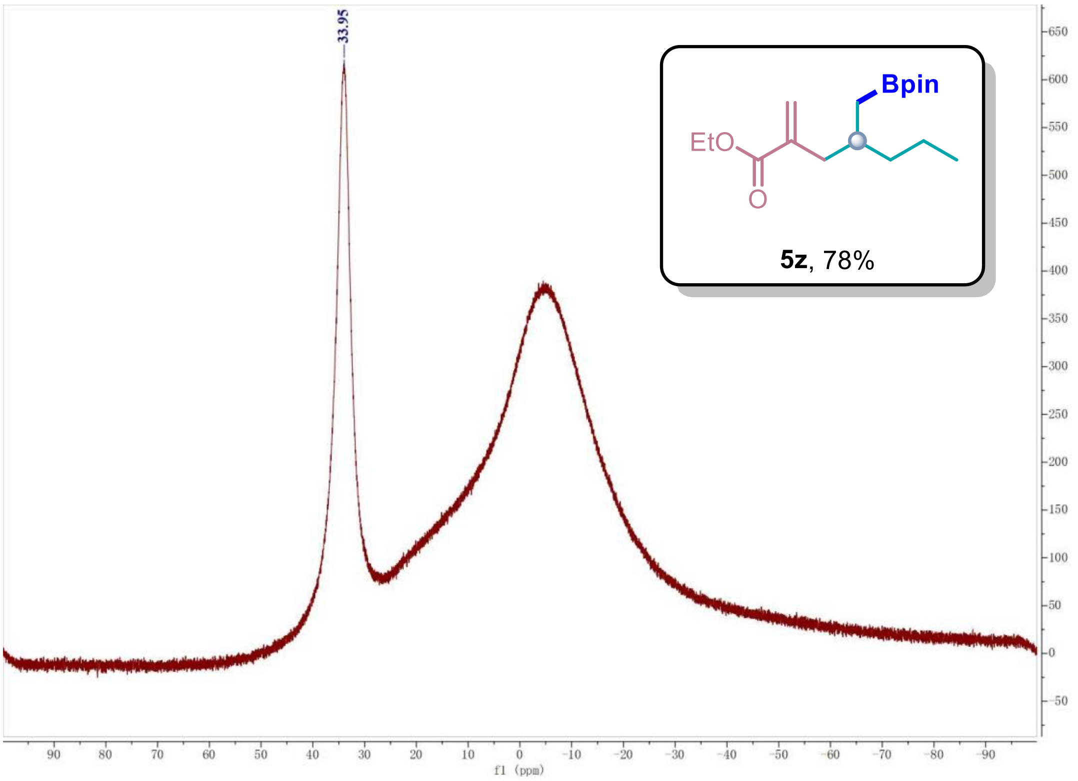

ethyl 6,10-dimethyl-2-methylene-4-((4,4,5,5-tetramethyl-1,3,2-dioxaborolan-2-yl)methyl)undec-9-enoate (5aa)

^{1}H NMR (500 MHz, Chloroform-*d*)

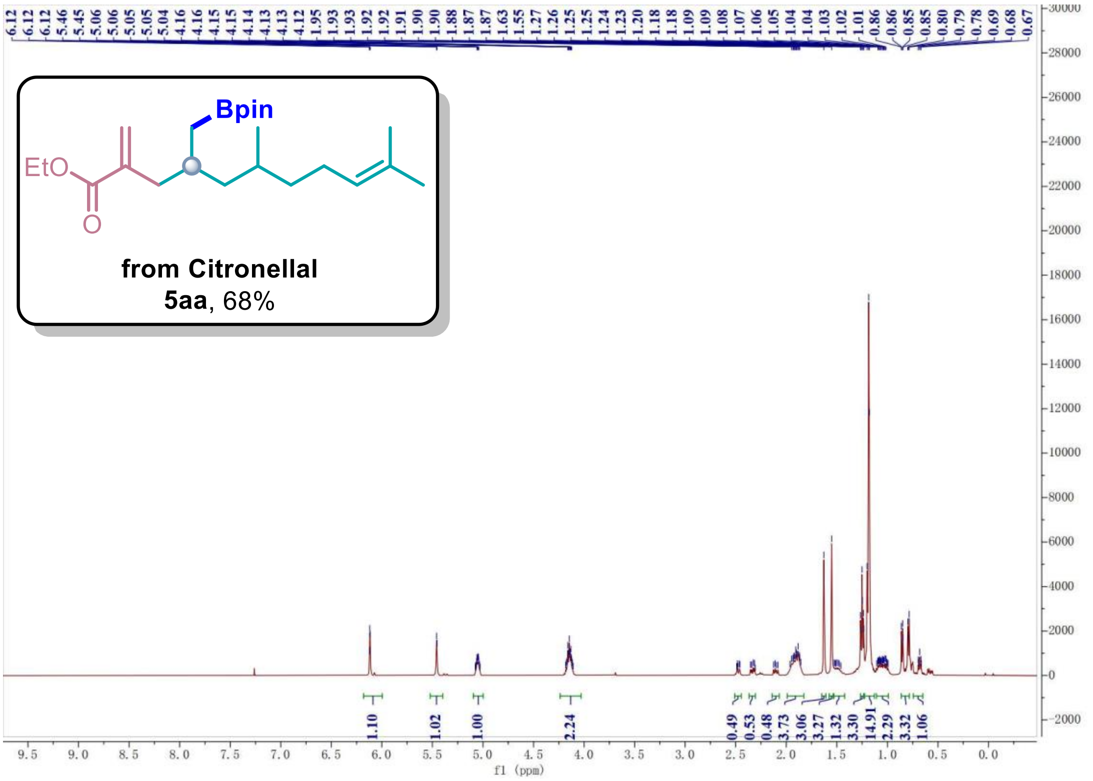

^{13}C NMR (126 MHz, Chloroform-*d*)

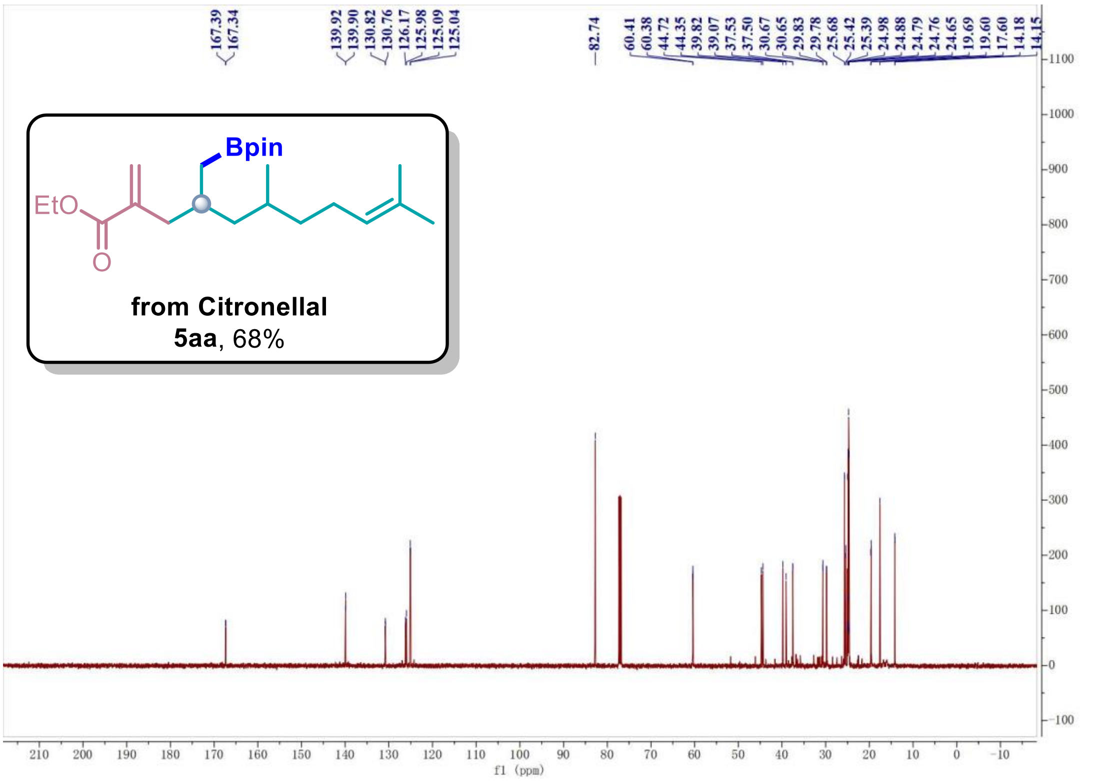

^{11}B NMR (160 MHz, Chloroform-*d*)

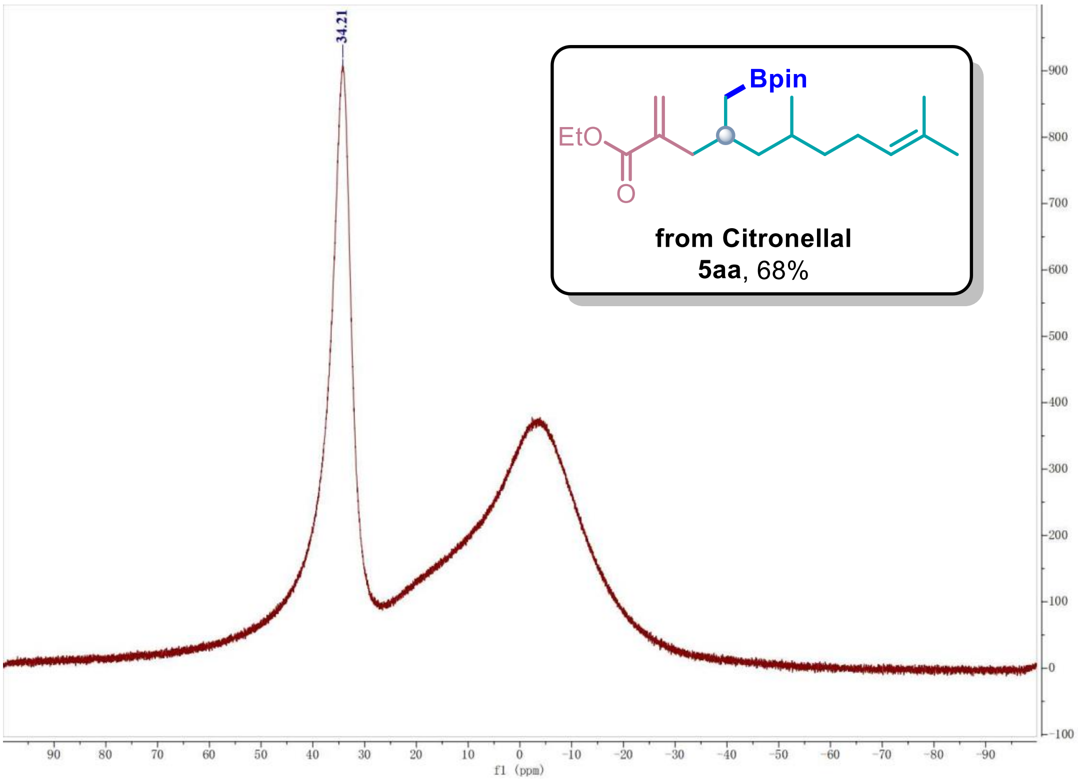

ethyl 2-methylene-4-((4,4,5,5-tetramethyl-1,3,2-dioxaborolan-2-yl)methyl)pentadecanoate (5ab)

^{1}H NMR (500 MHz, Chloroform-*d*)

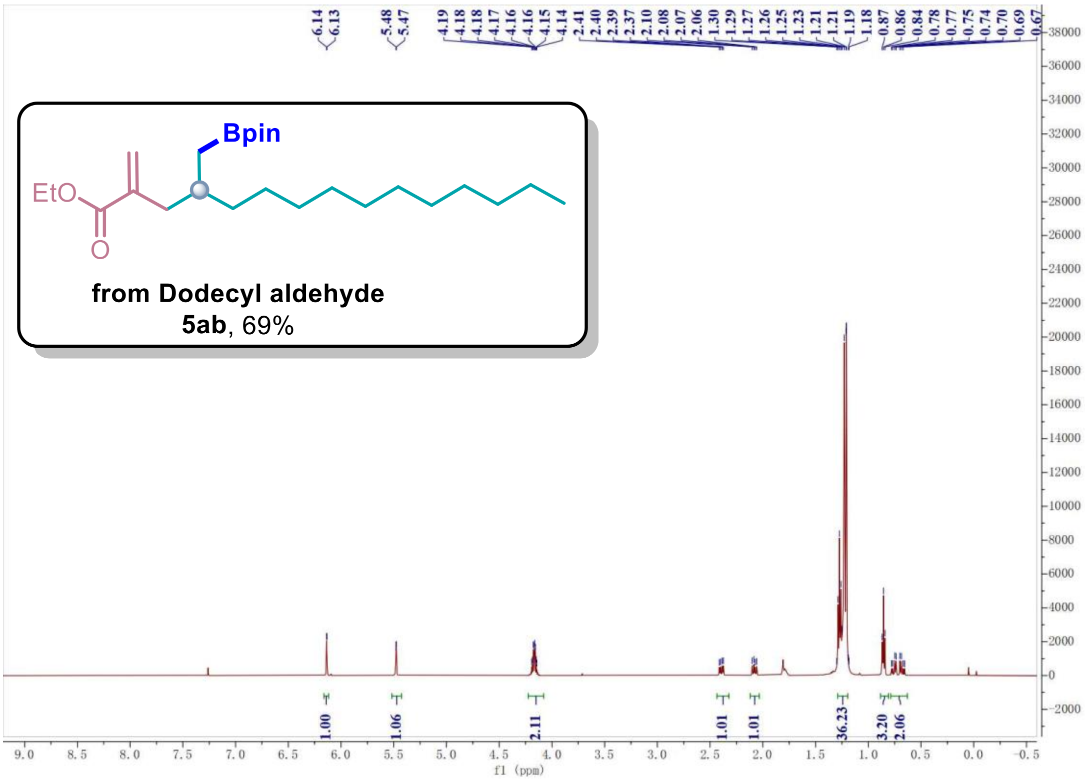

^{13}C NMR (126 MHz, Chloroform-*d*)

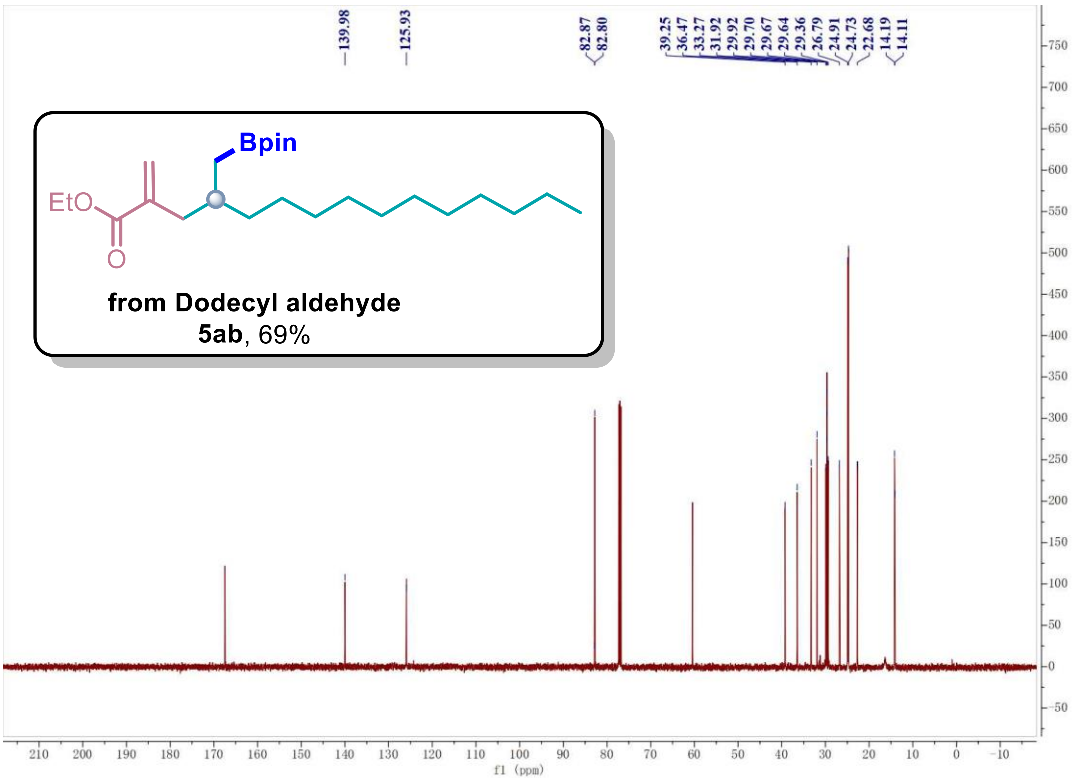

^{11}B NMR (160 MHz, Chloroform-*d*)

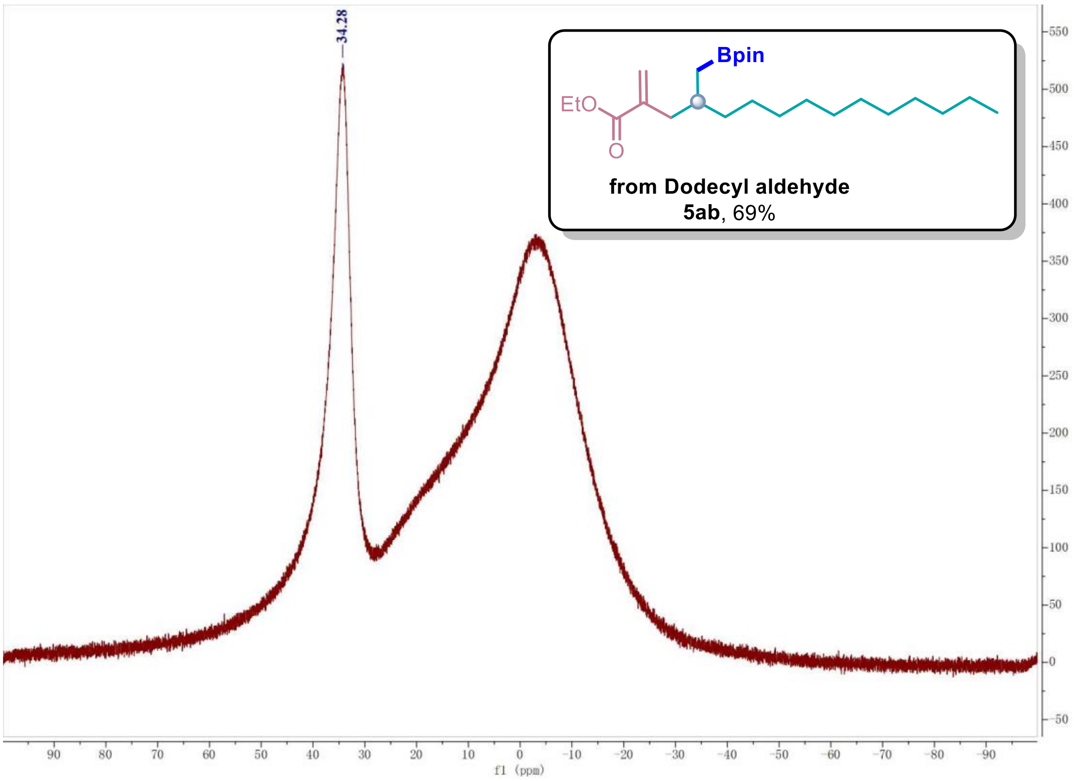

5,5-dimethyl-2-(2-neopentyl-4-phenylpent-4-en-1-yl)-1,3,2-dioxaborinane (5ac)

^{1}H NMR (500 MHz, Chloroform-*d*)

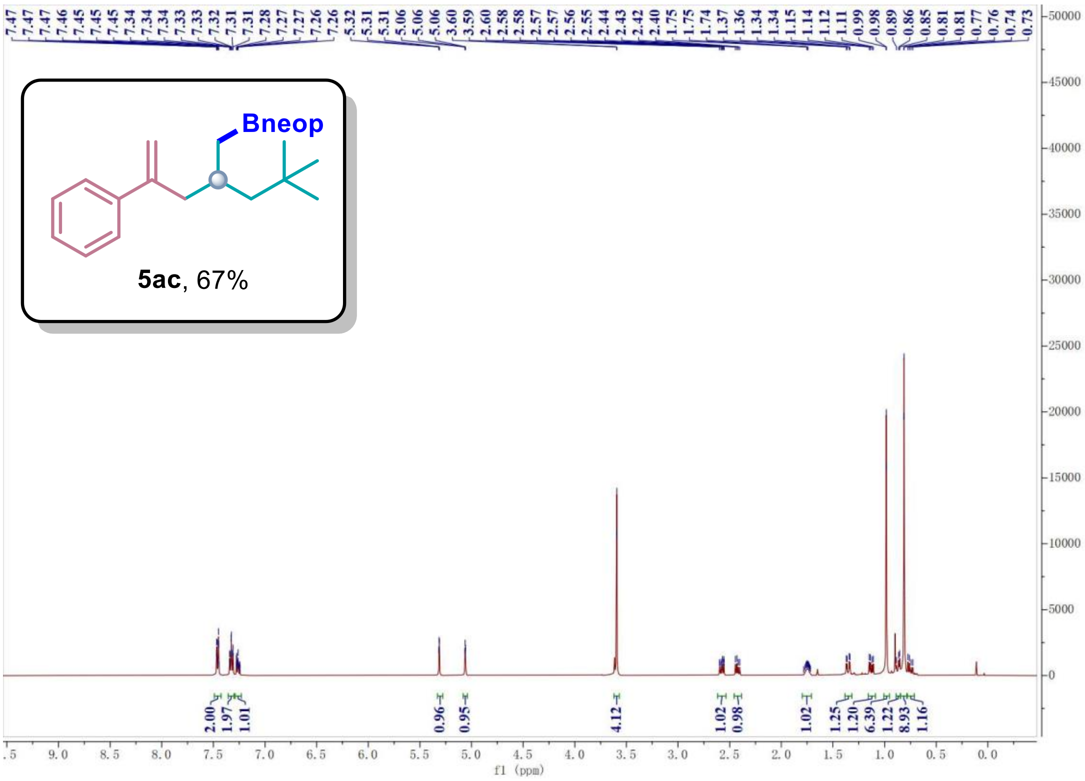

^{13}C NMR (126 MHz, Chloroform-*d*)

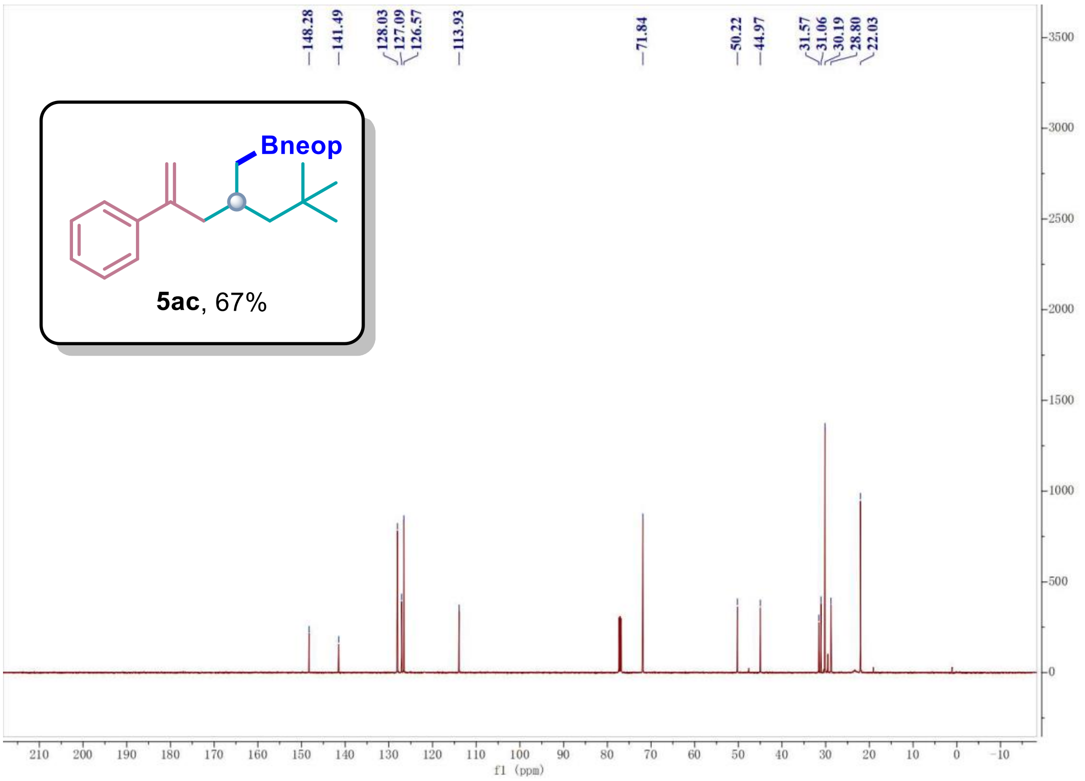

^{11}B NMR (160 MHz, Chloroform-*d*)

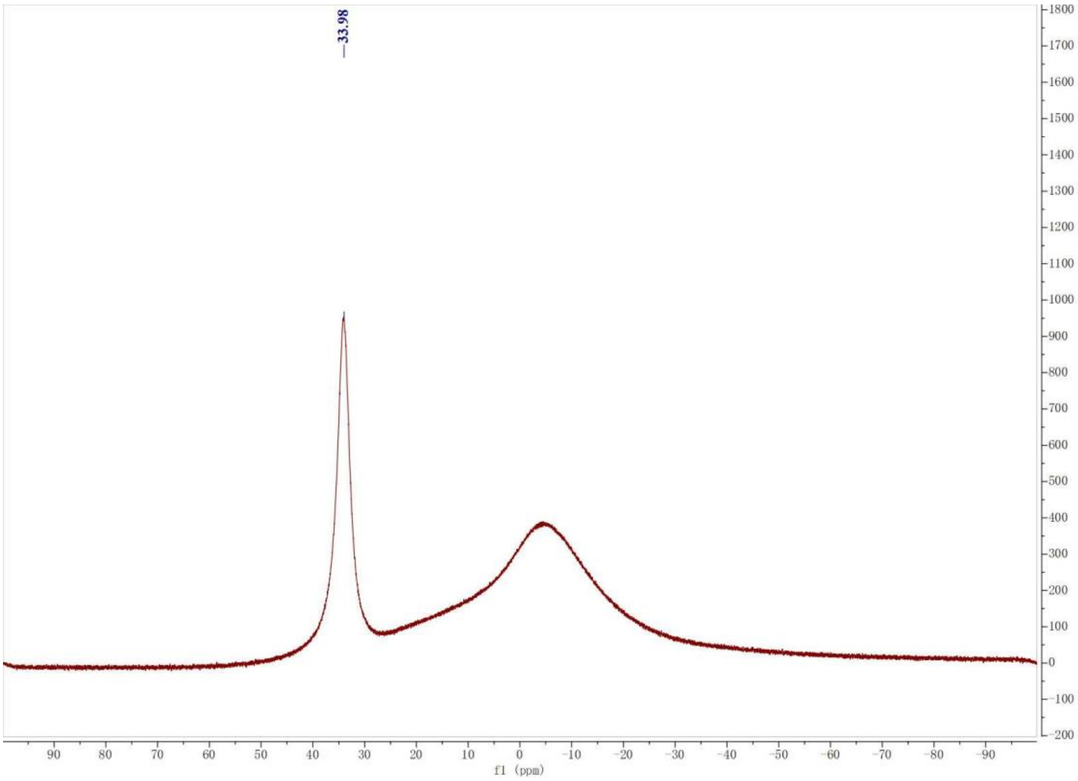

ethyl 6,6-dimethyl-4-((4,4,5,5-tetramethyl-1,3,2-dioxaborolan-2-yl)methyl)heptanoate (7a)

^{1}H NMR (500 MHz, Chloroform-*d*)

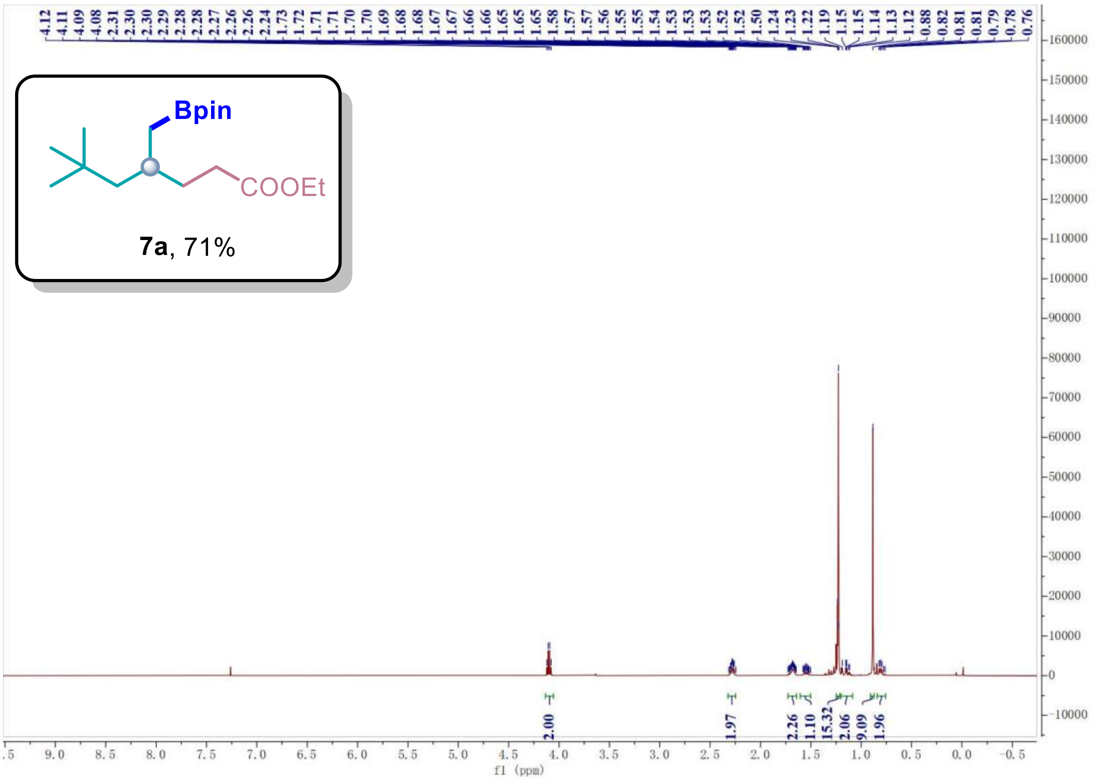

^{13}C NMR (126 MHz, Chloroform-*d*)

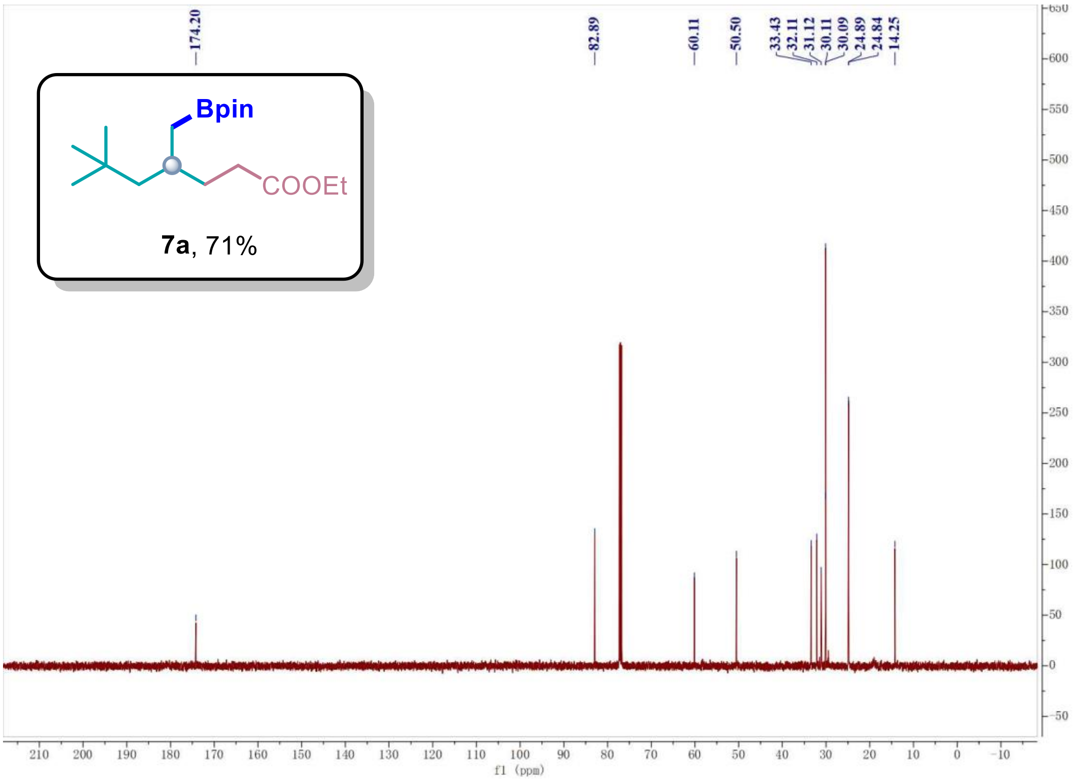

^{11}B NMR (160 MHz, Chloroform-*d*)

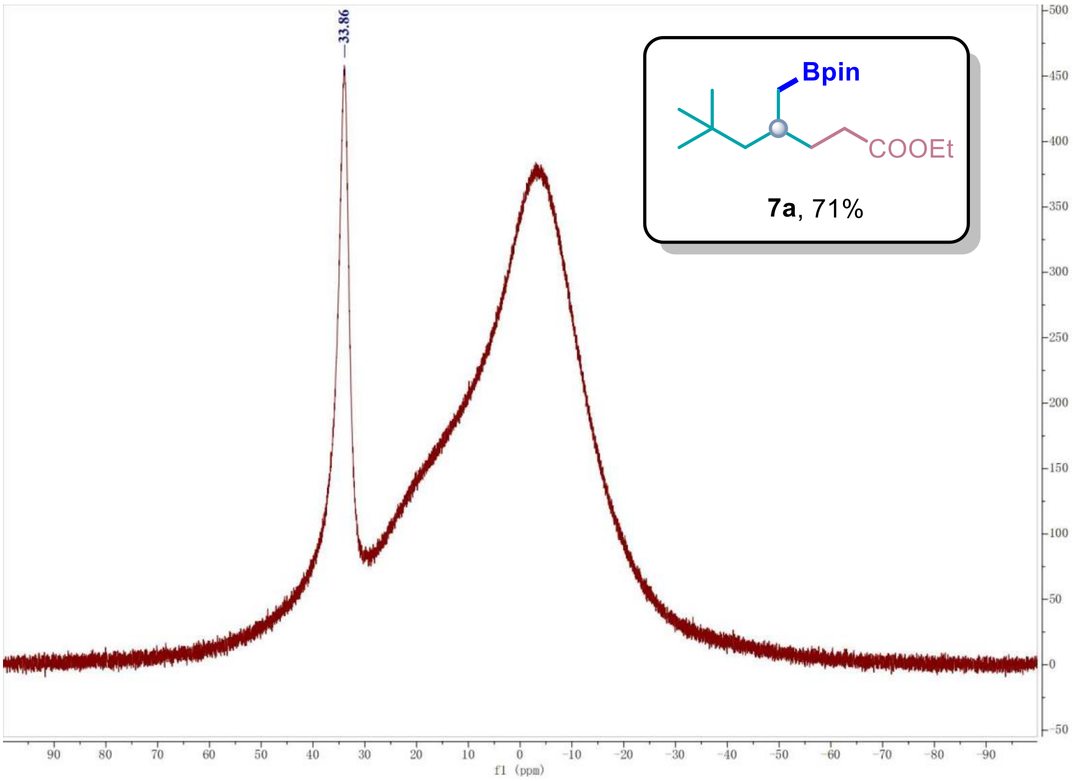

tert-butyl 6,6-dimethyl-4-((4,4,5,5-tetramethyl-1,3,2-dioxaborolan-2-yl)methyl)heptanoate (7b)

^{1}H NMR (500 MHz, Chloroform-*d*)

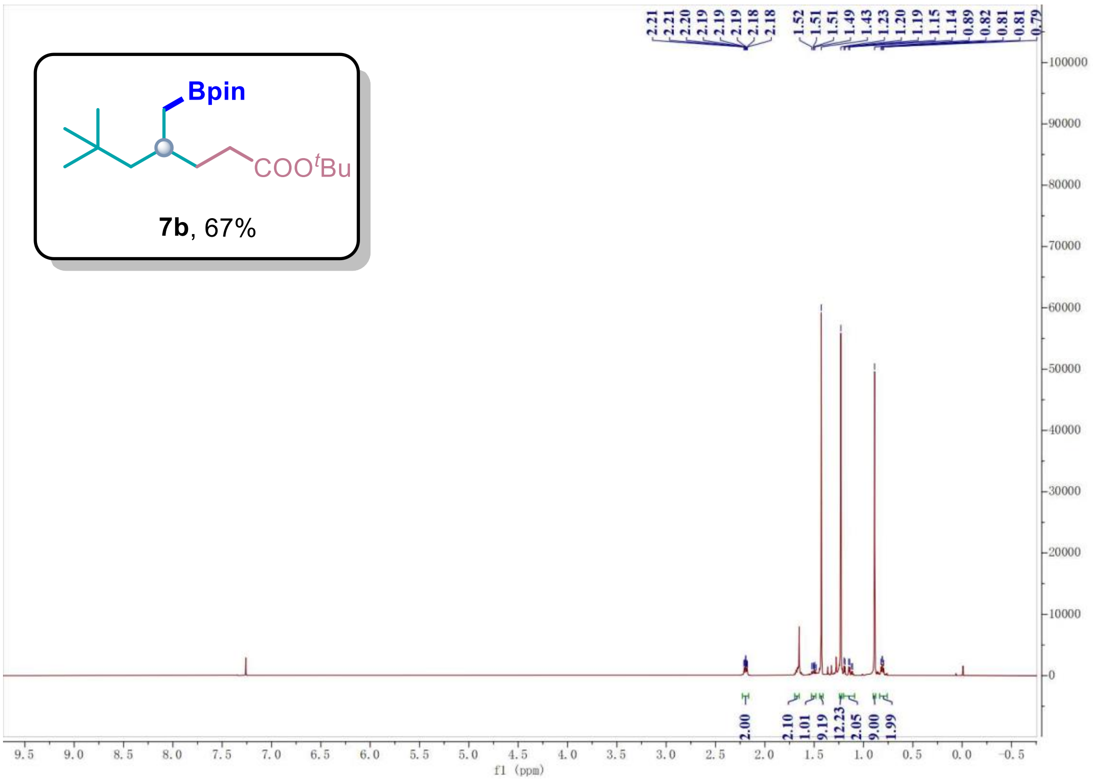

^{13}C NMR (126 MHz, Chloroform-*d*)

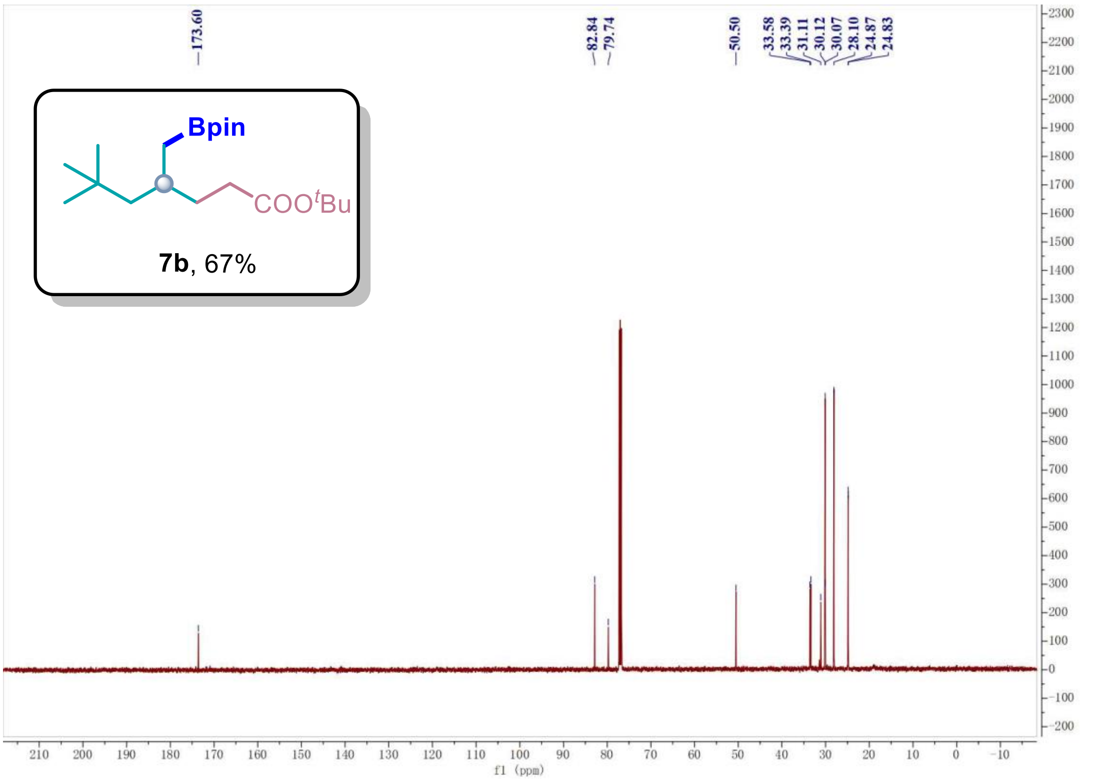

^{11}B NMR (160 MHz, Chloroform-*d*)

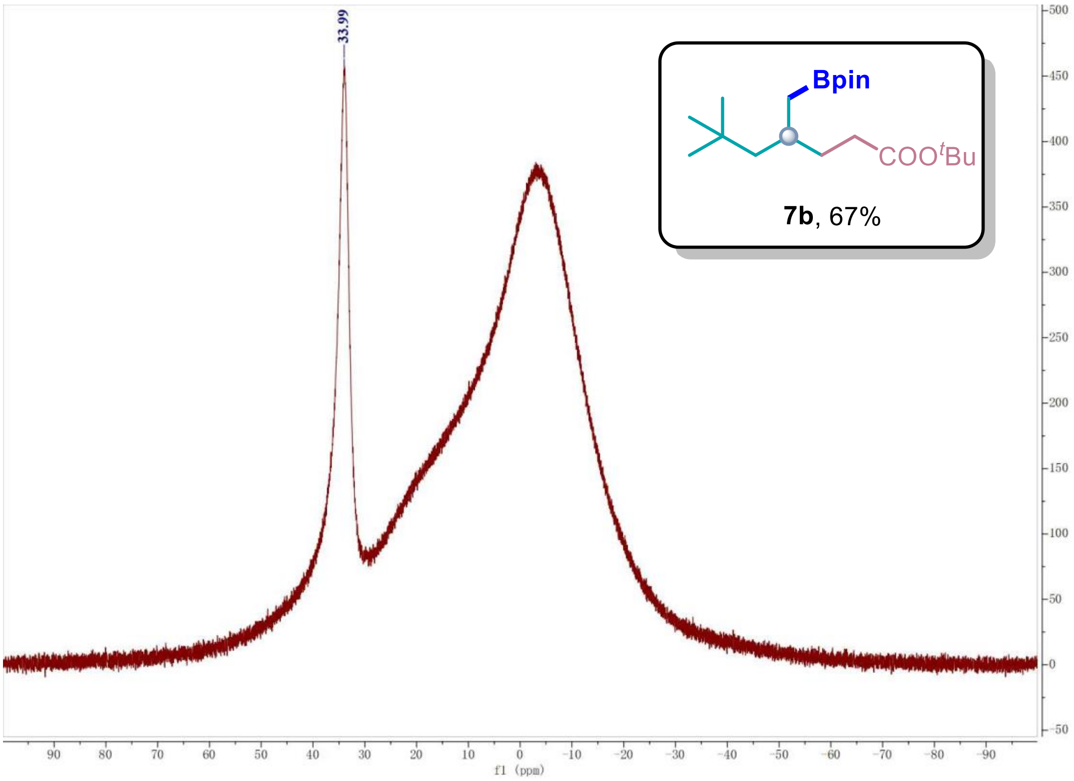

benzyl 6,6-dimethyl-4-((4,4,5,5-tetramethyl-1,3,2-dioxaborolan-2-yl)methyl)heptanoate (4c)

^{1}H NMR (500 MHz, Chloroform-*d*)

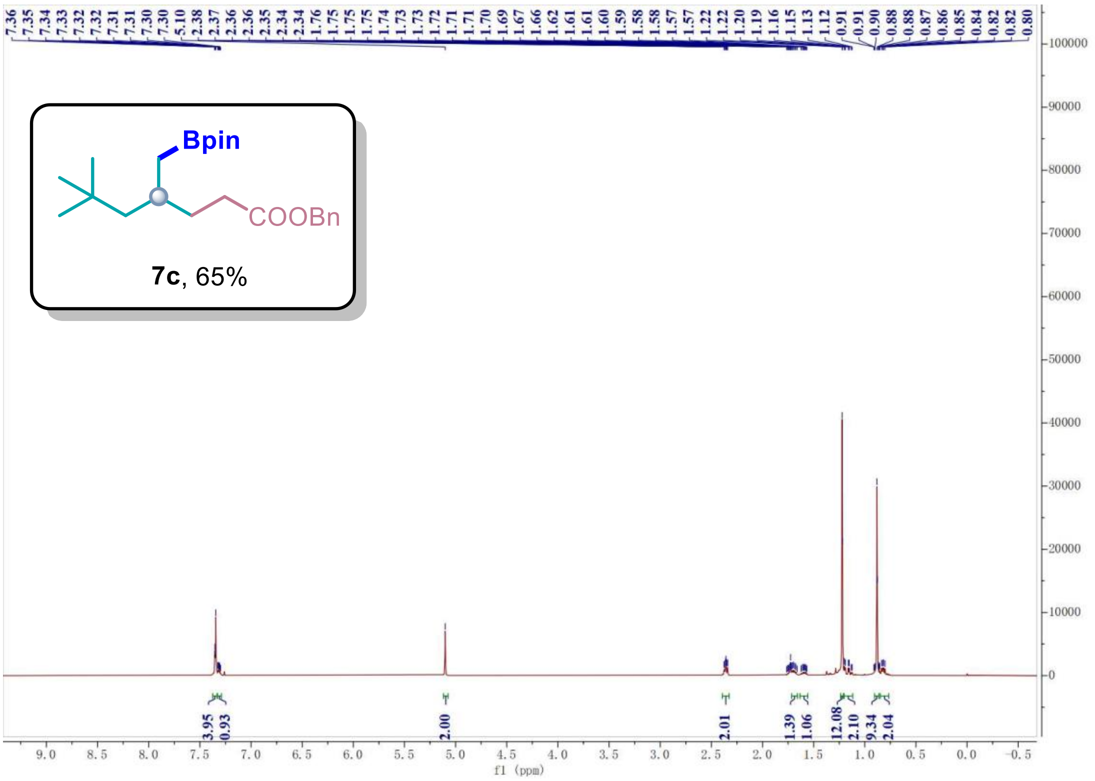

^{13}C NMR (126 MHz, Chloroform-*d*)

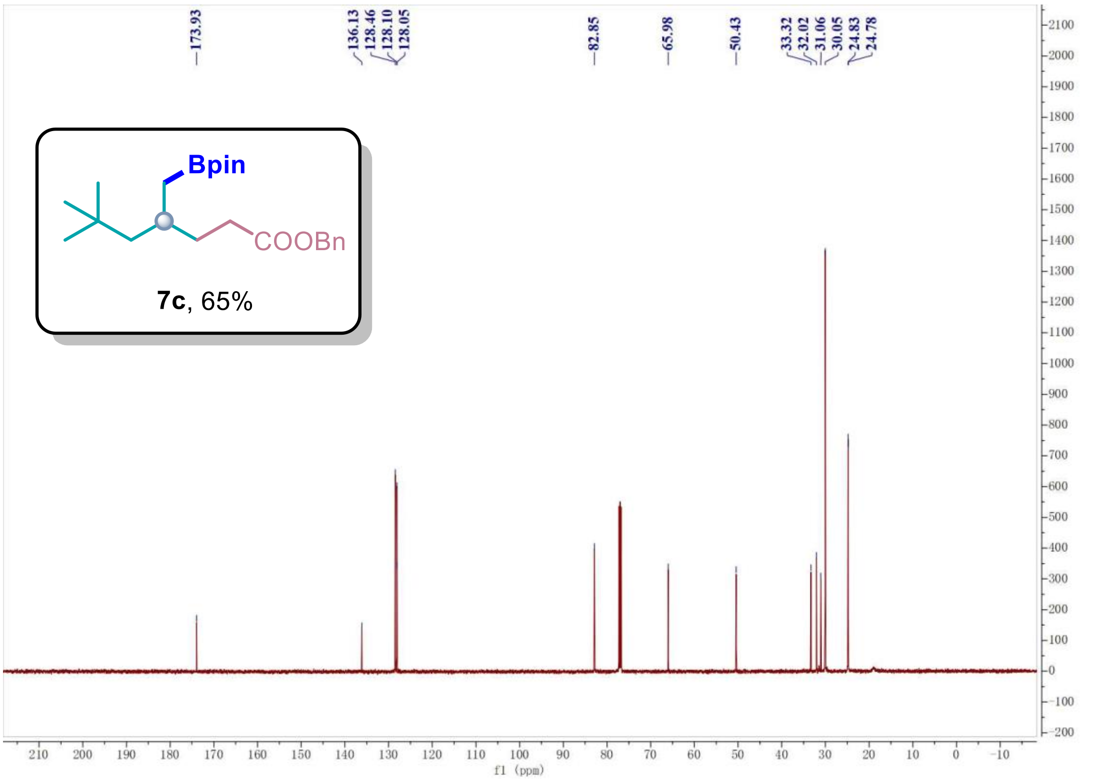

^{11}B NMR (160 MHz, Chloroform-*d*)

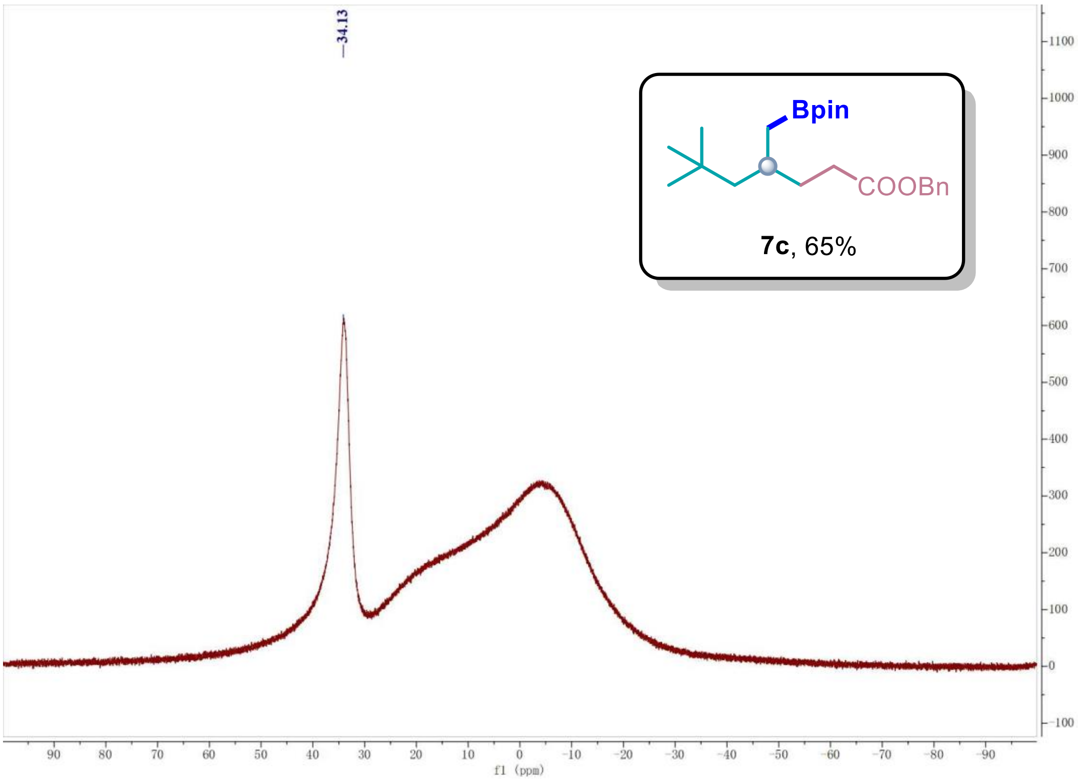

phenyl 6,6-dimethyl-4-((4,4,5,5-tetramethyl-1,3,2-dioxaborolan-2-yl)methyl)heptanoate (7d)

^{1}H NMR (500 MHz, Chloroform-*d*)

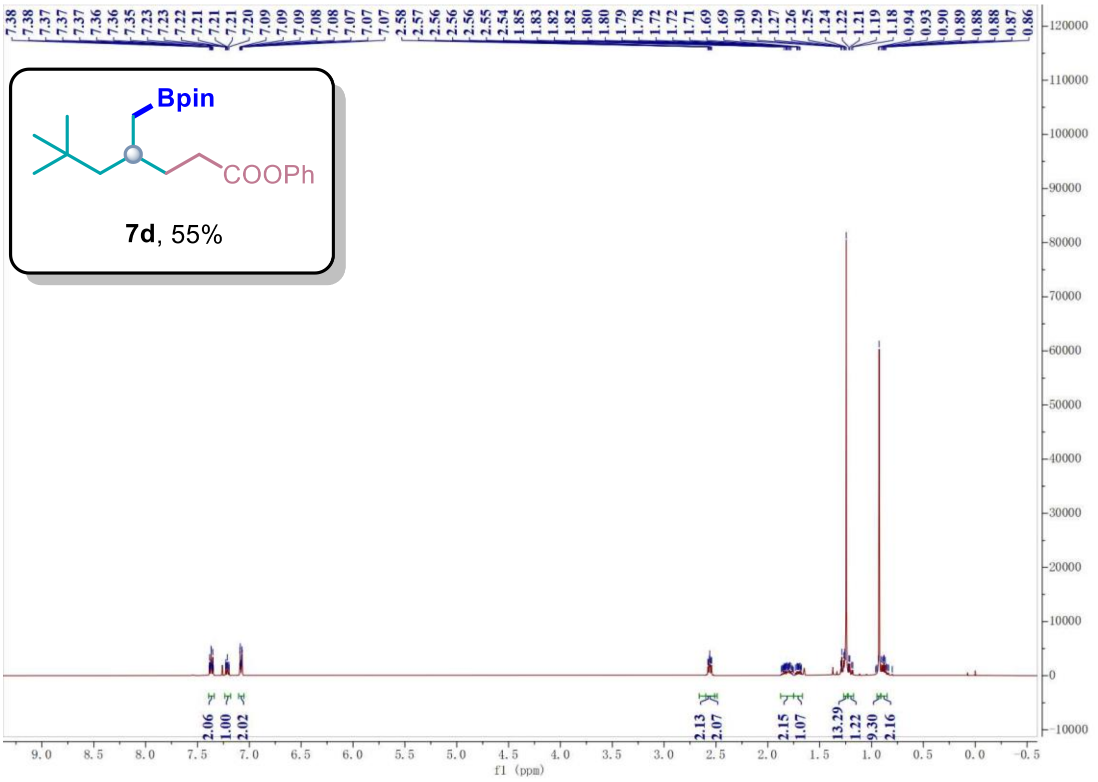

^{13}C NMR (126 MHz, Chloroform-*d*)

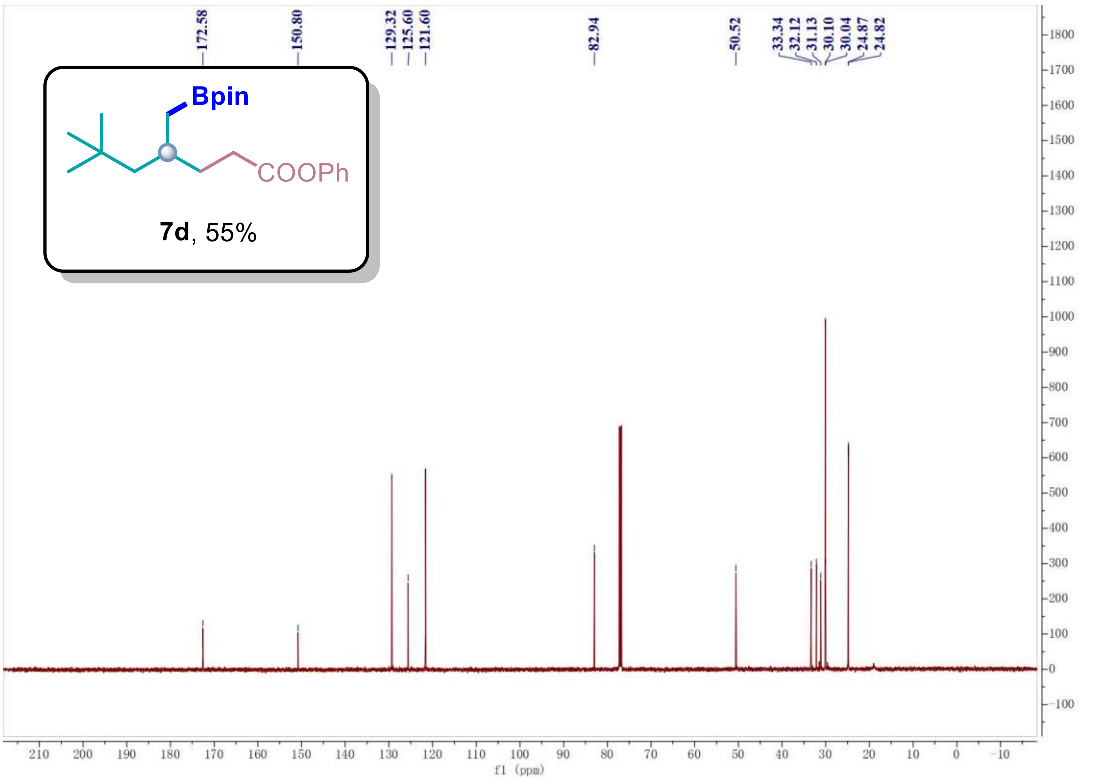

^{11}B NMR (160 MHz, Chloroform-*d*)

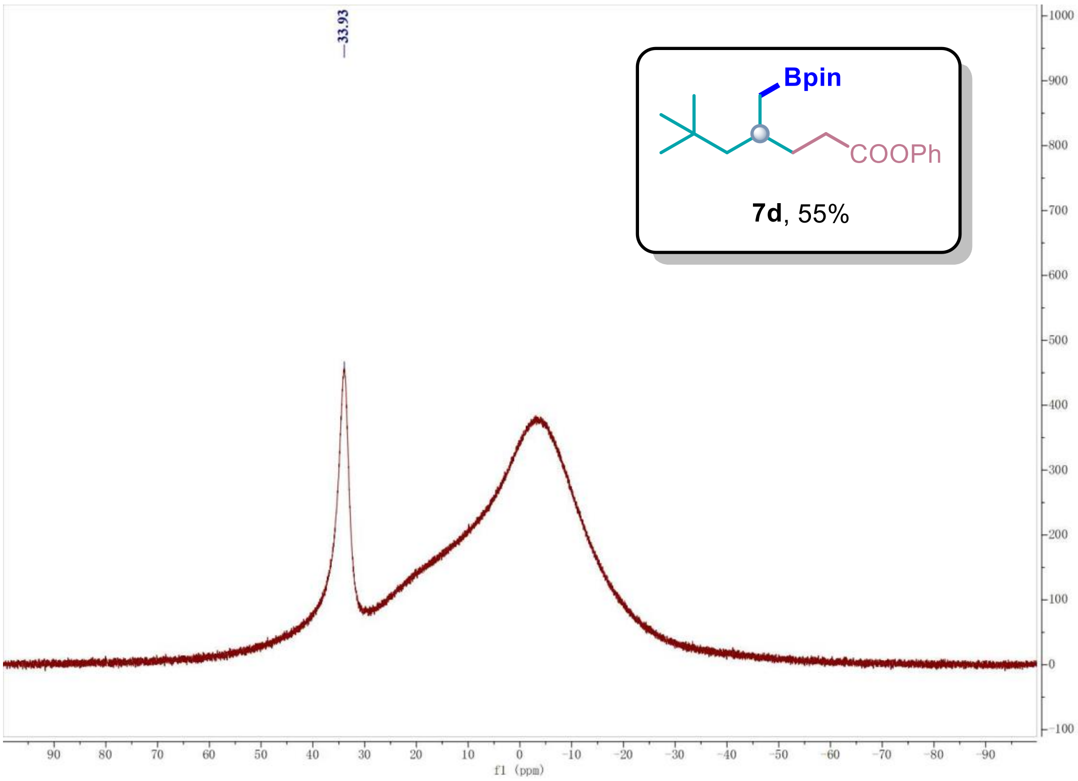

6,6-dimethyl-4-((4,4,5,5-tetramethyl-1,3,2-dioxaborolan-2-yl)methyl)heptanenitrile (7e)

^{1}H NMR (500 MHz, Chloroform-*d*)

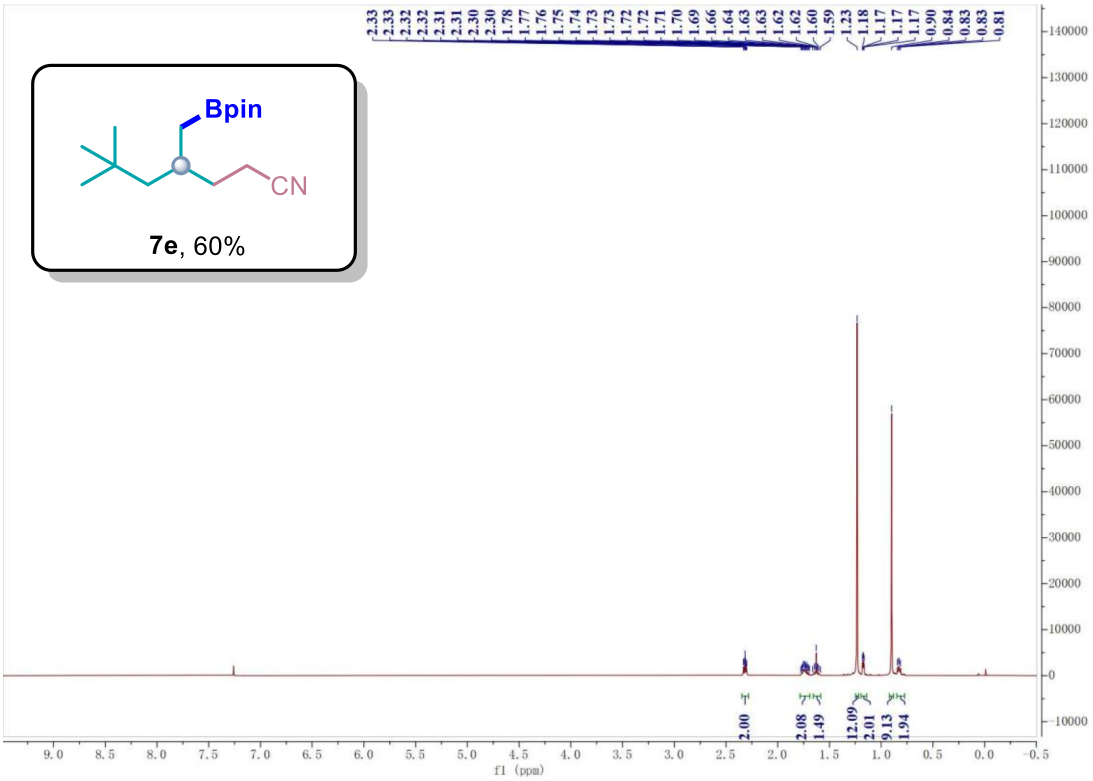

^{13}C NMR (126 MHz, Chloroform-*d*)

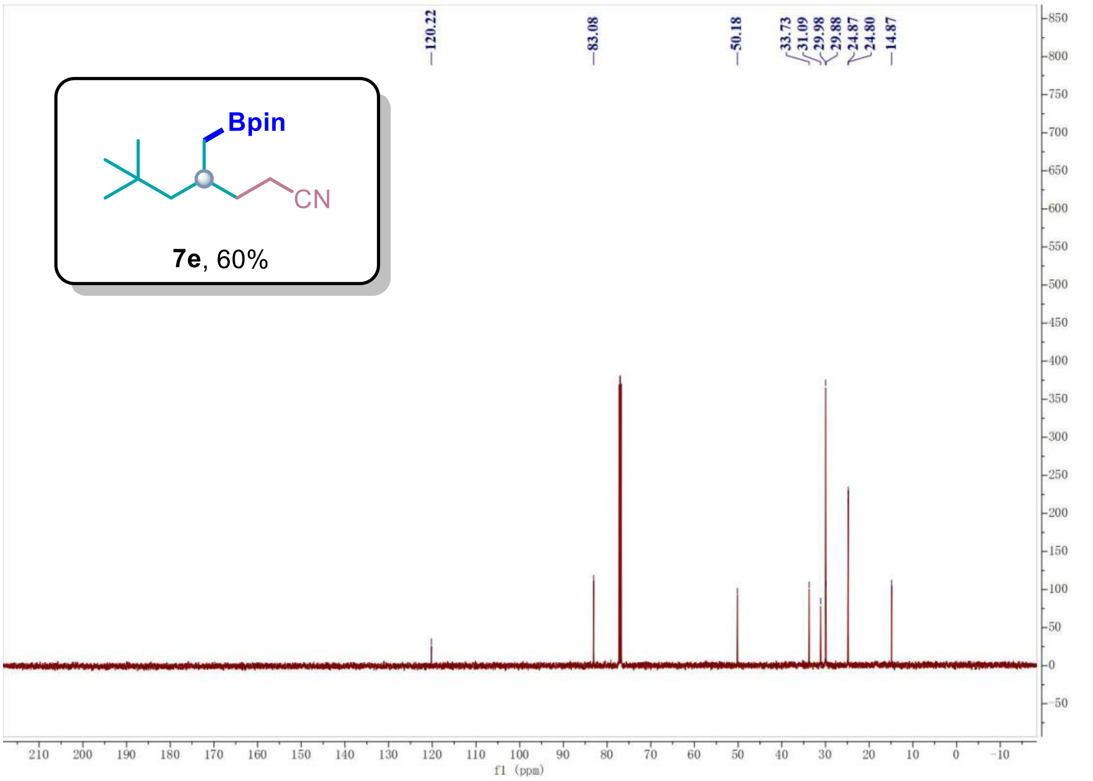

^{11}B NMR (160 MHz, Chloroform-*d*)

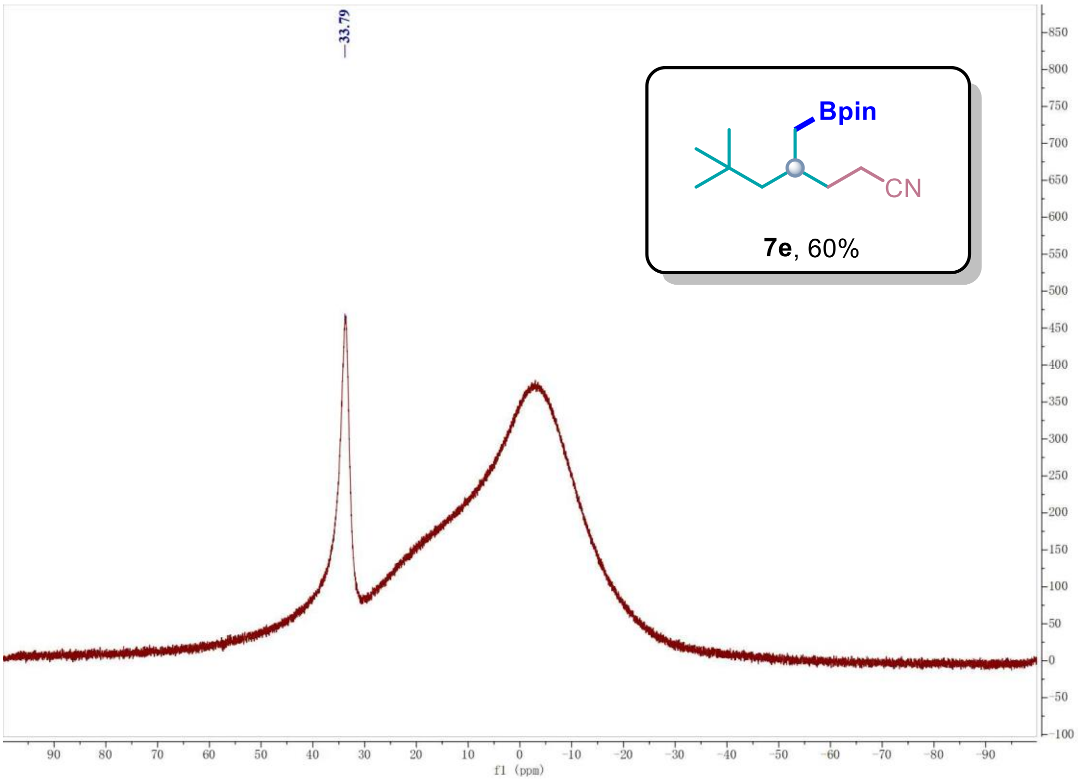

ethyl (5,5-dimethyl-3-((4,4,5,5-tetramethyl-1,3,2-dioxaborolan-2-yl)methyl)hexyl)(oxo)-l4-phosphanecarboxylate (7f)

^{1}H NMR (500 MHz, Chloroform-*d*)

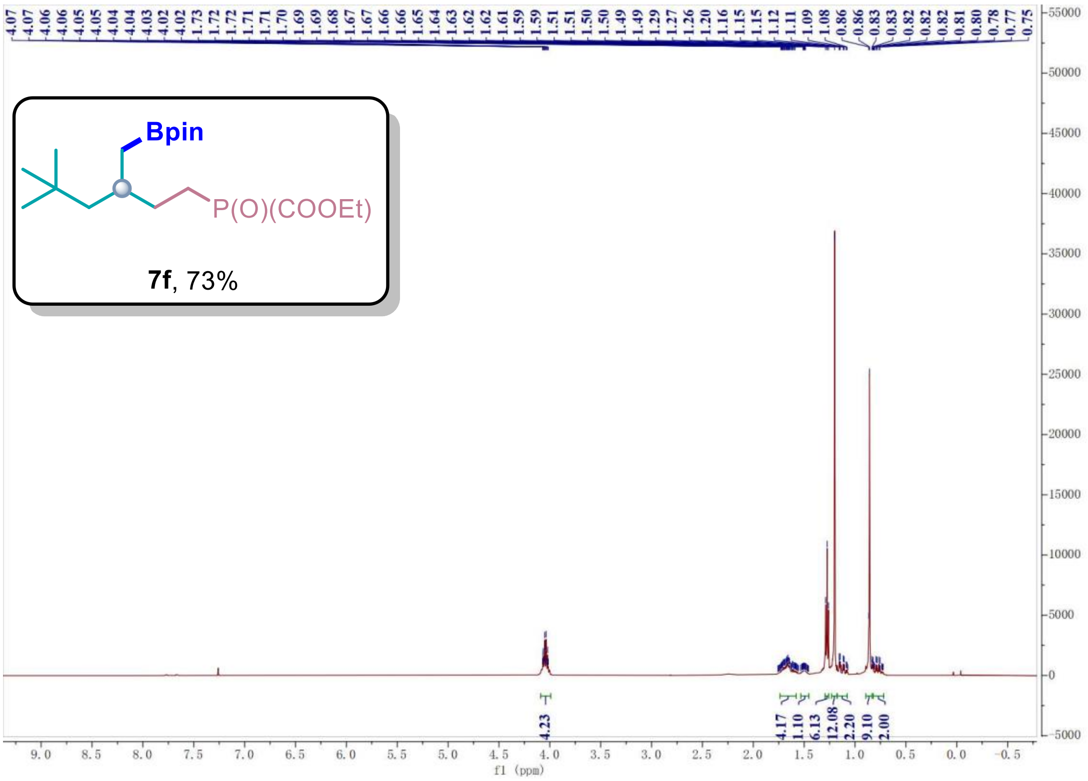

^{13}C NMR (126 MHz, Chloroform-*d*)

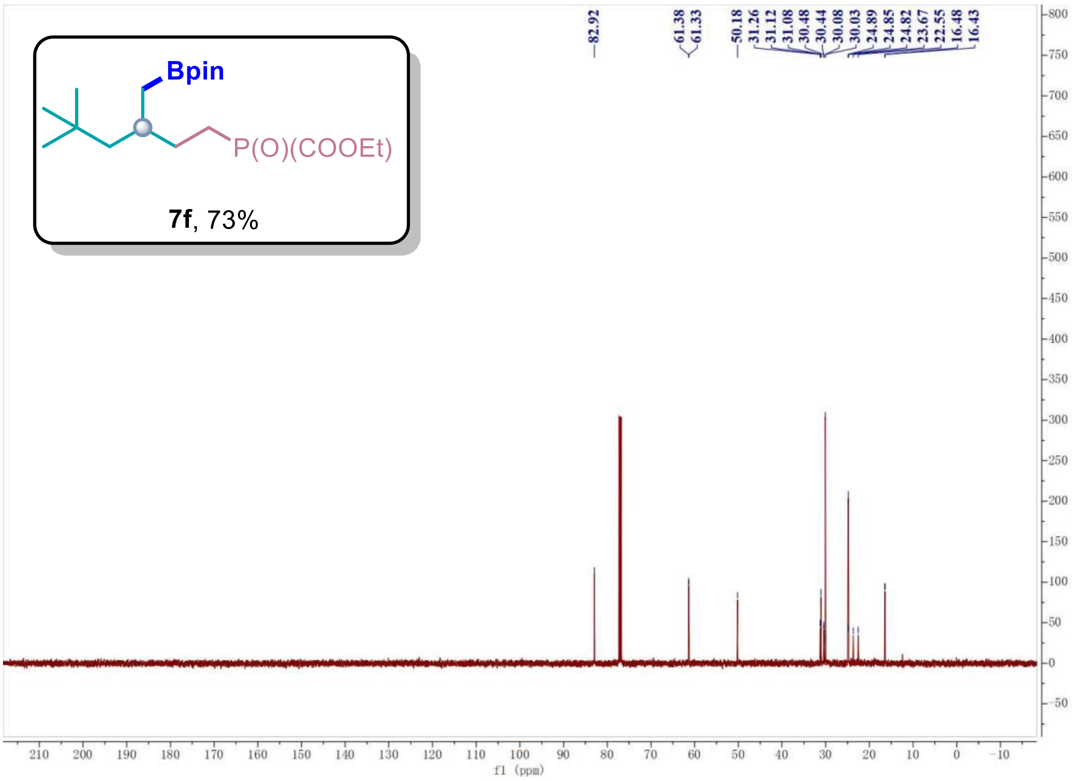

^{11}B NMR (160 MHz, Chloroform-*d*)

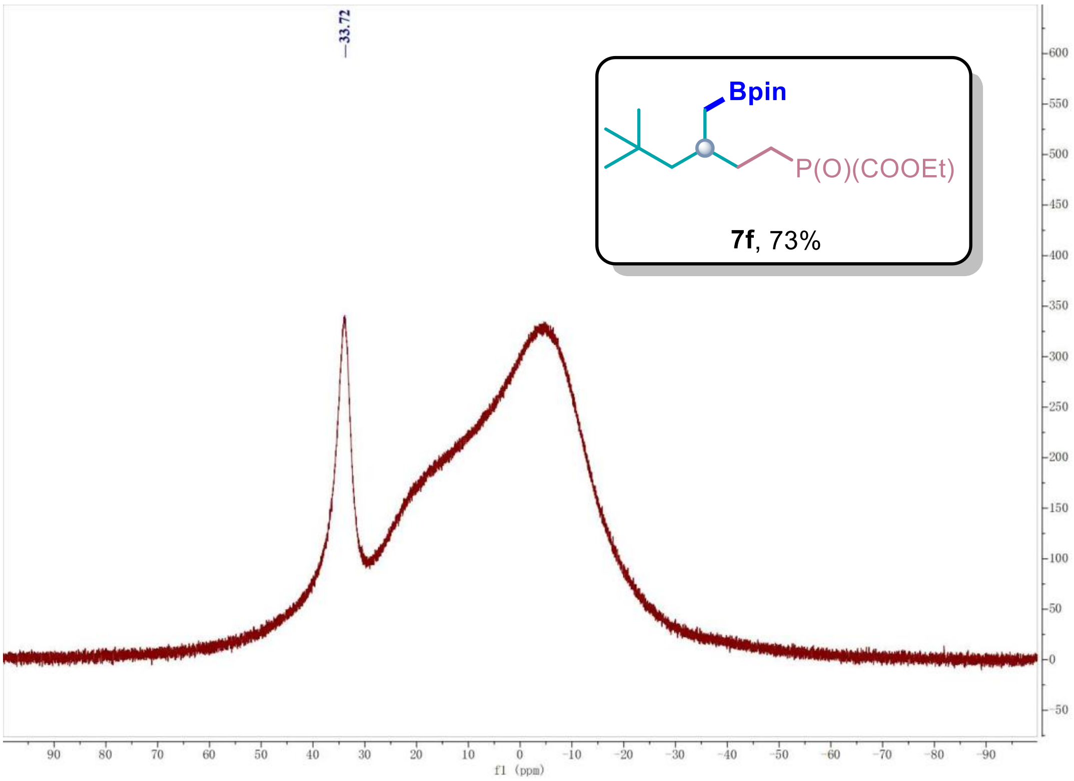

N-(tert-butyl)-6,6-dimethyl-4-((4,4,5,5-tetramethyl-1,3,2-dioxaborolan-2-yl)methyl)heptanamide (7g)

^{1}H NMR (500 MHz, Chloroform-*d*)

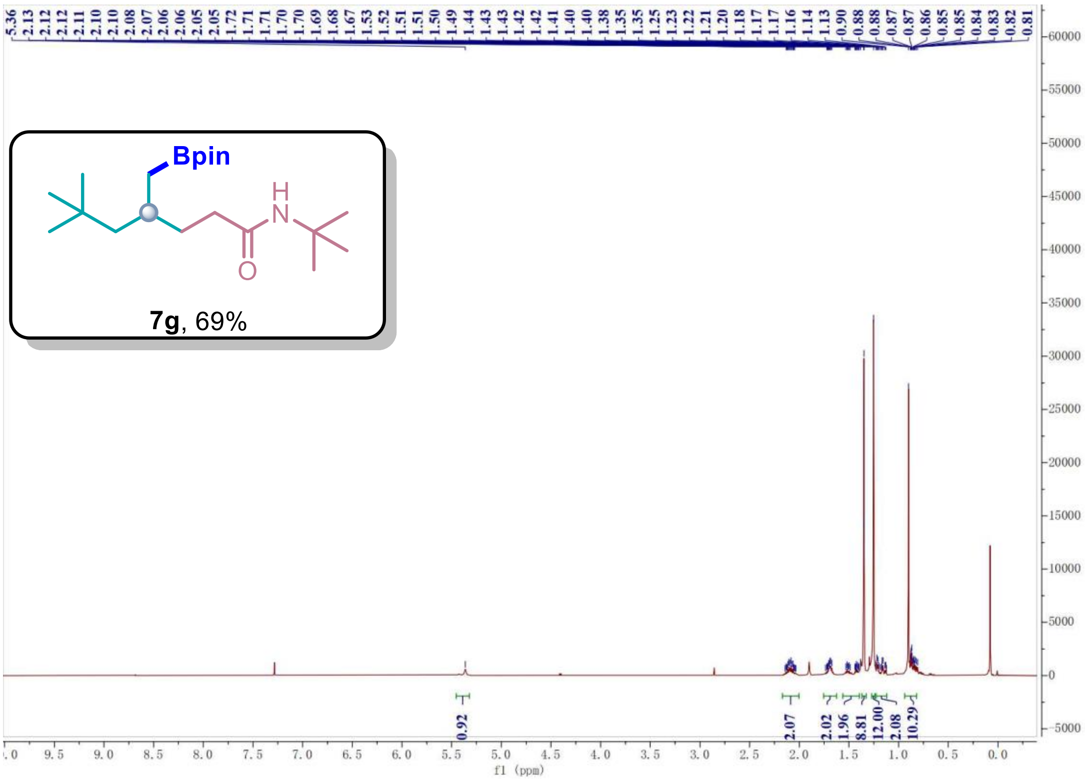

^{13}C NMR (126 MHz, Chloroform-*d*)

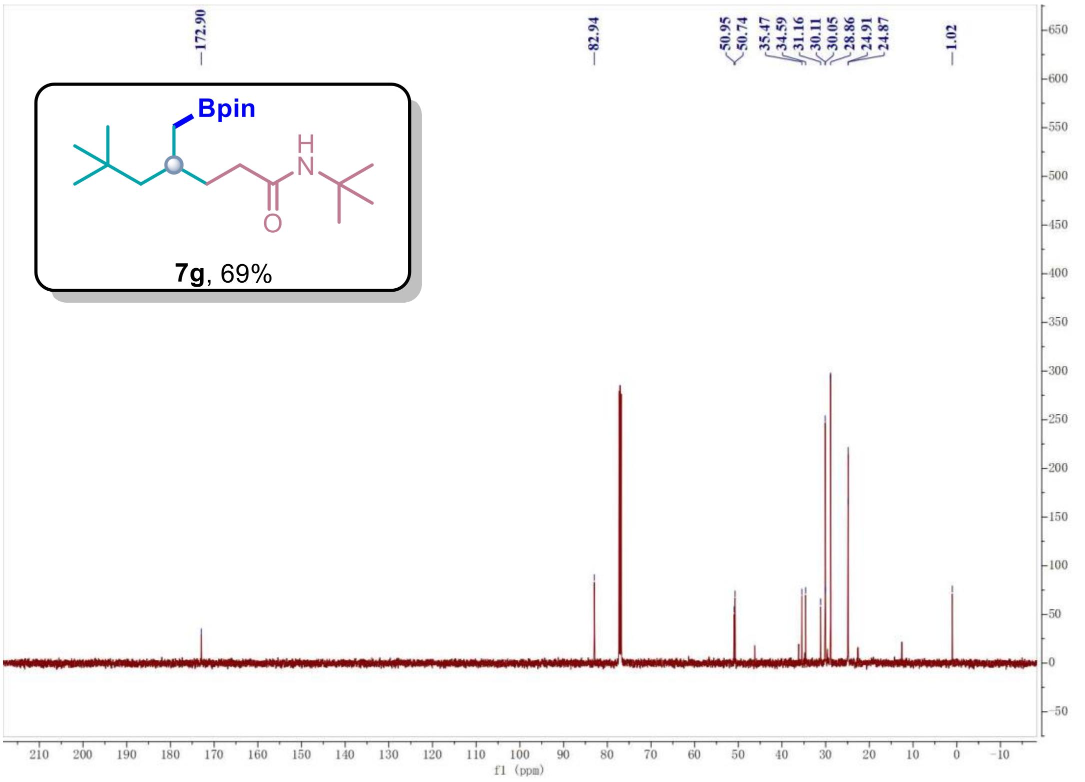

^{11}B NMR (160 MHz, Chloroform-*d*)

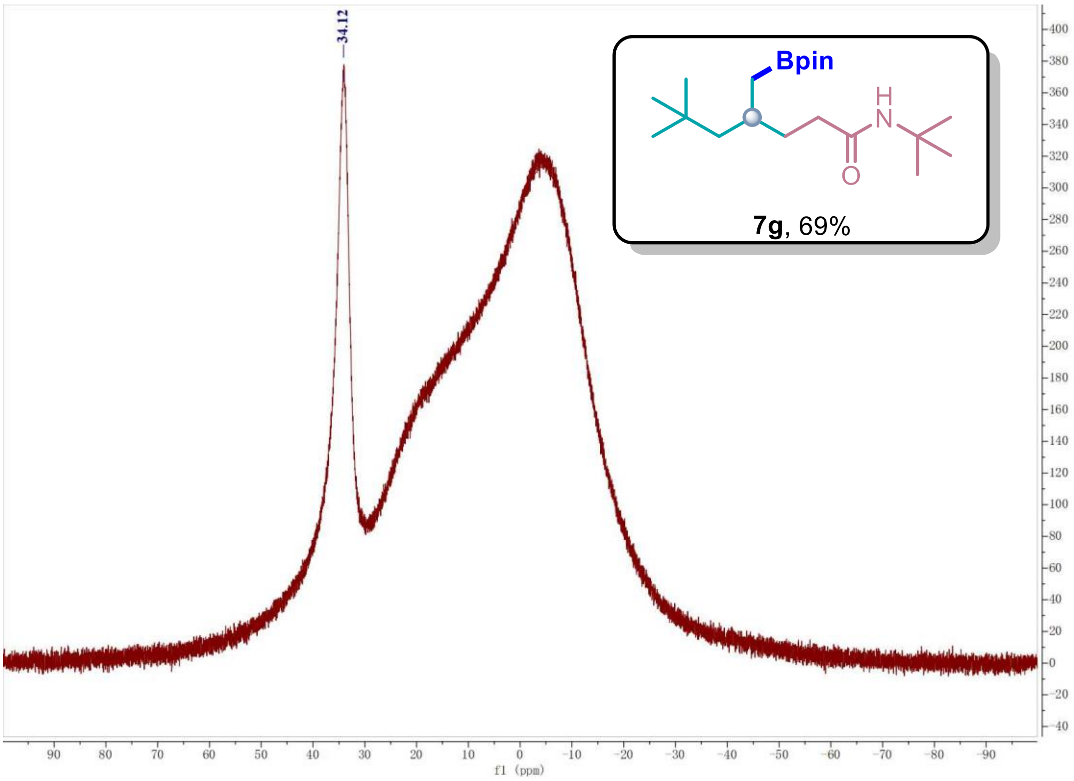

6,6-dimethyl-4-((4,4,5,5-tetramethyl-1,3,2-dioxaborolan-2-yl)methyl)heptanamide (7h)

^{1}H NMR (500 MHz, Chloroform-*d*)

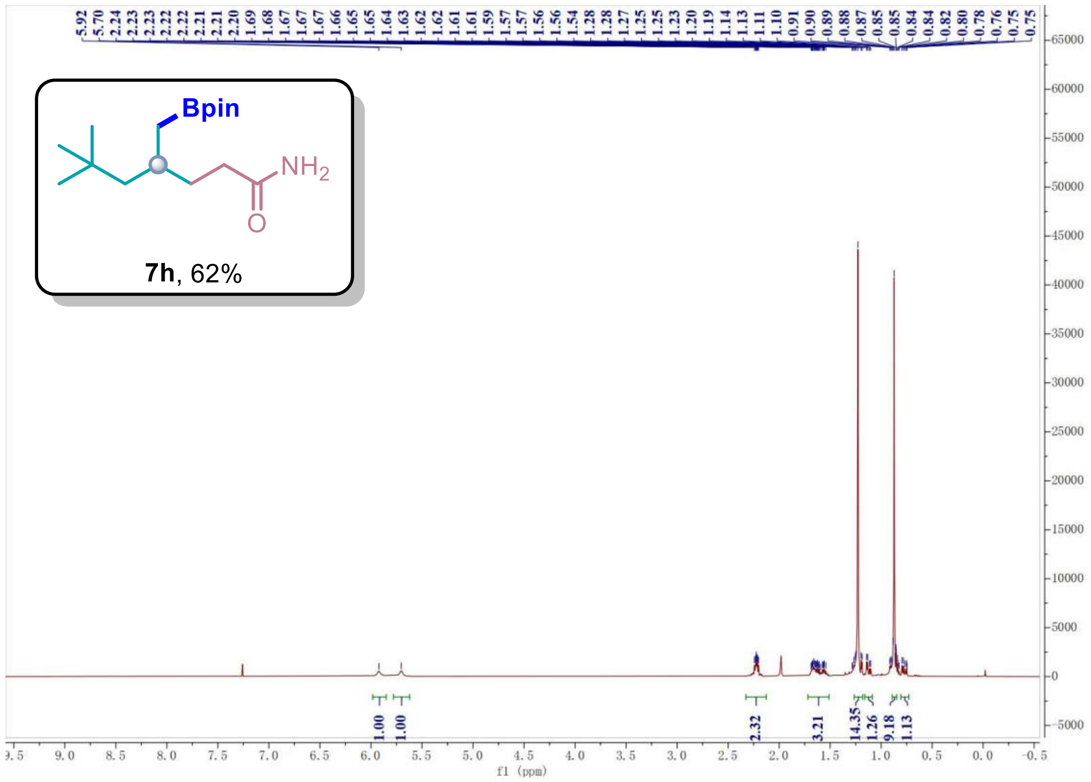

^{13}C NMR (126 MHz, Chloroform-*d*)

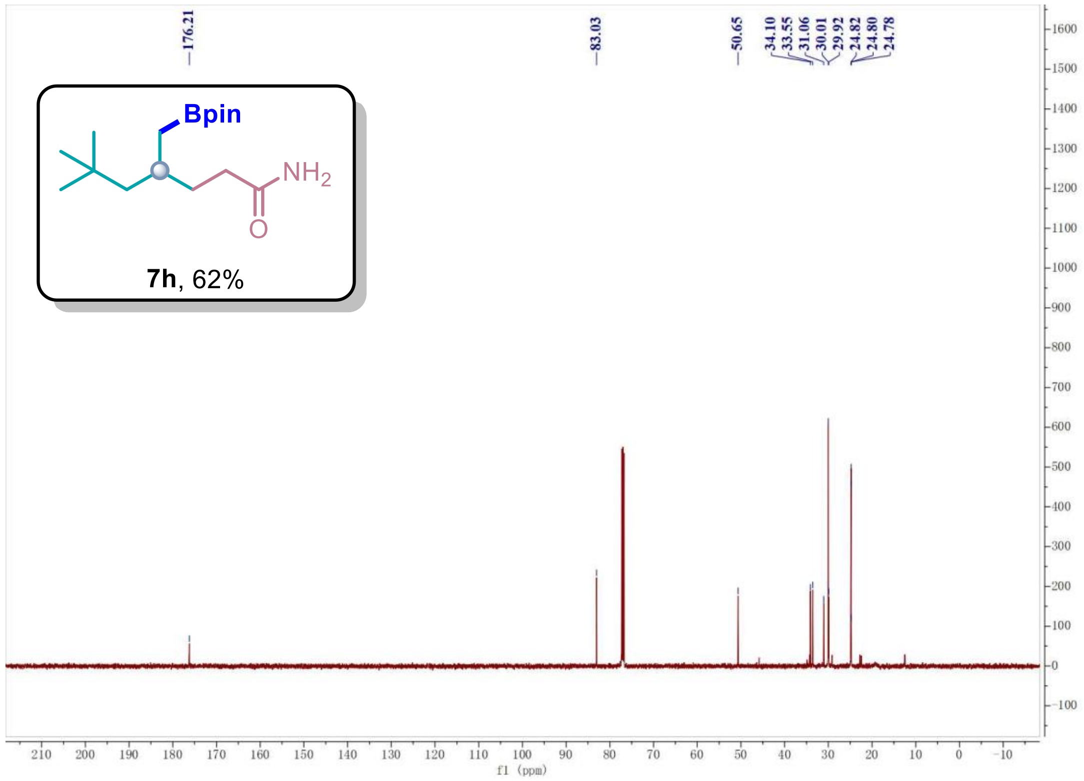

^{11}B NMR (160 MHz, Chloroform-*d*)

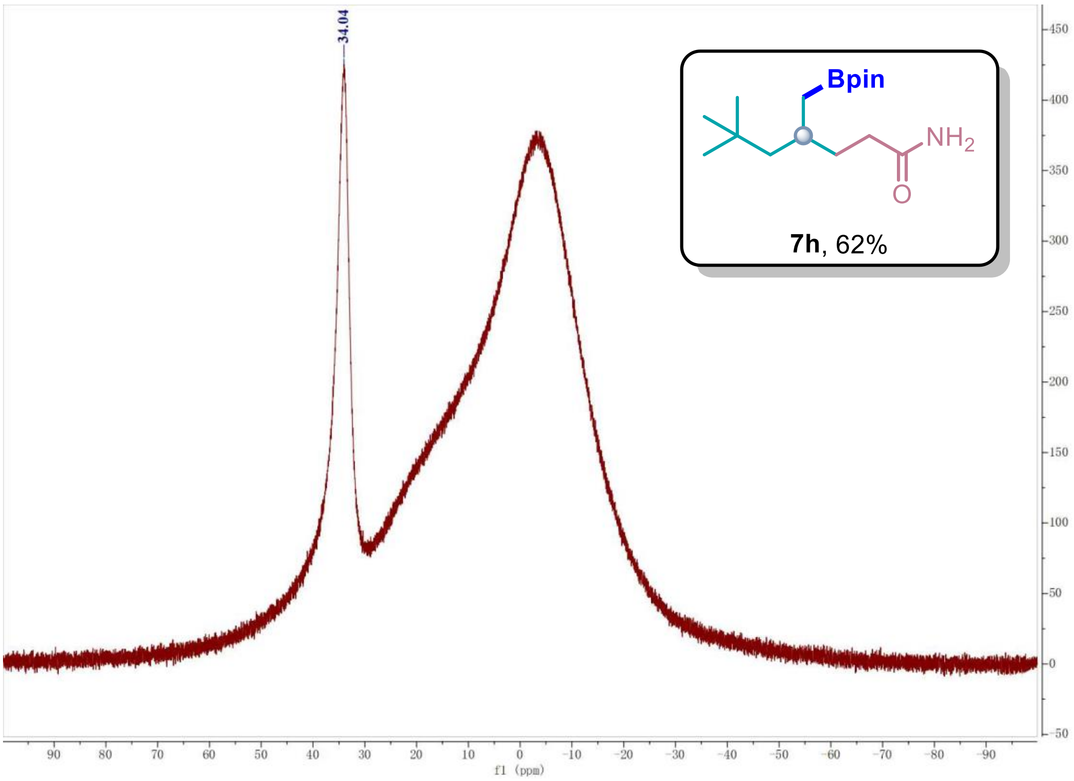

2-(4,4-dimethyl-2-(2-(methylsulfonyl)ethyl)pentyl)-4,4,5,5-tetramethyl-1,3,2-dioxaborolane (7i)

^{1}H NMR (500 MHz, Chloroform-*d*)

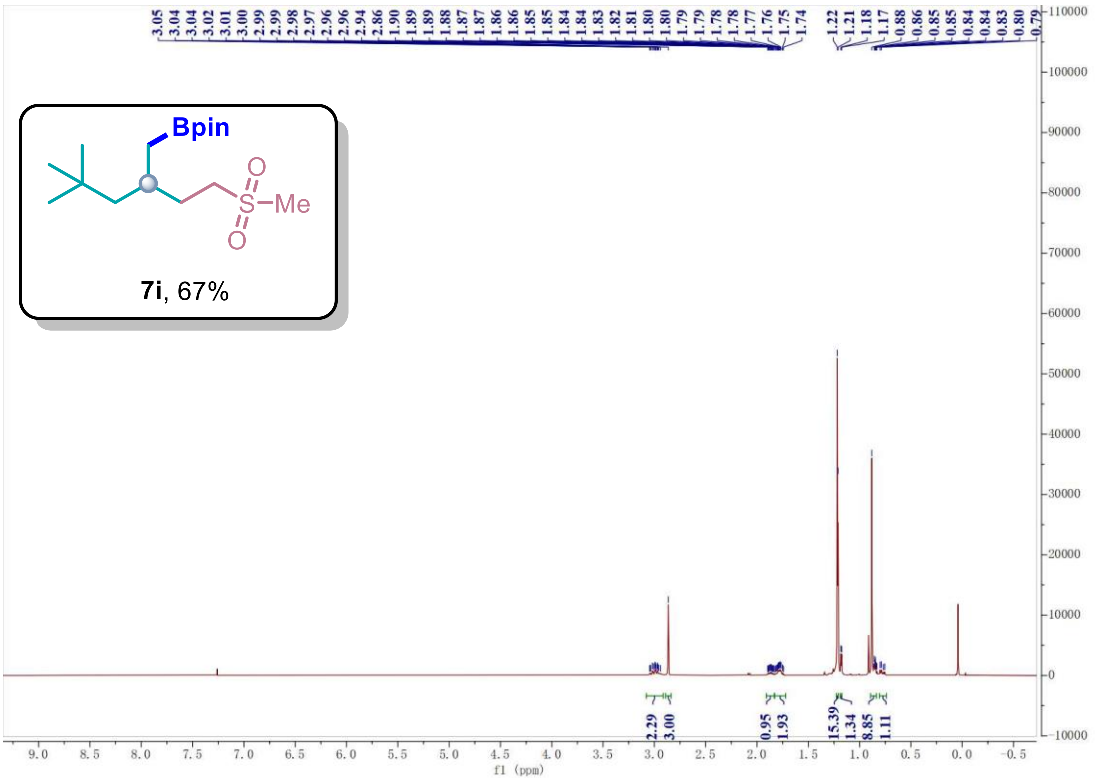

^{13}C NMR (126 MHz, Chloroform-*d*)

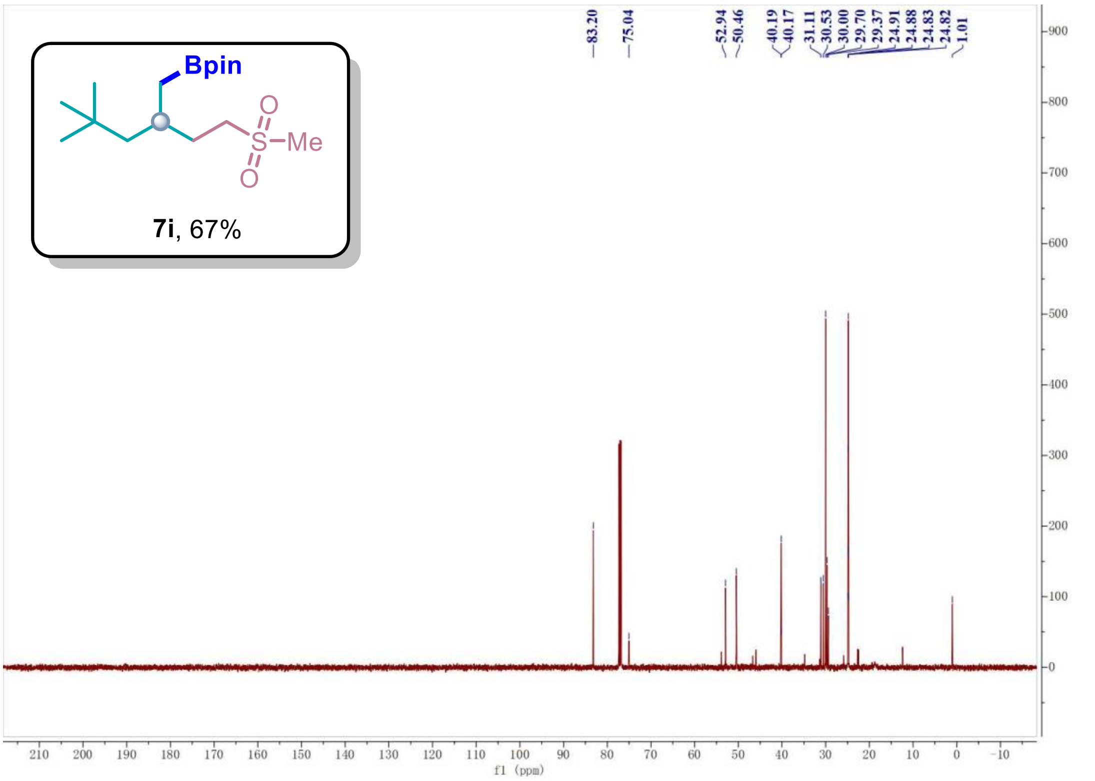

^{11}B NMR (160 MHz, Chloroform-*d*)

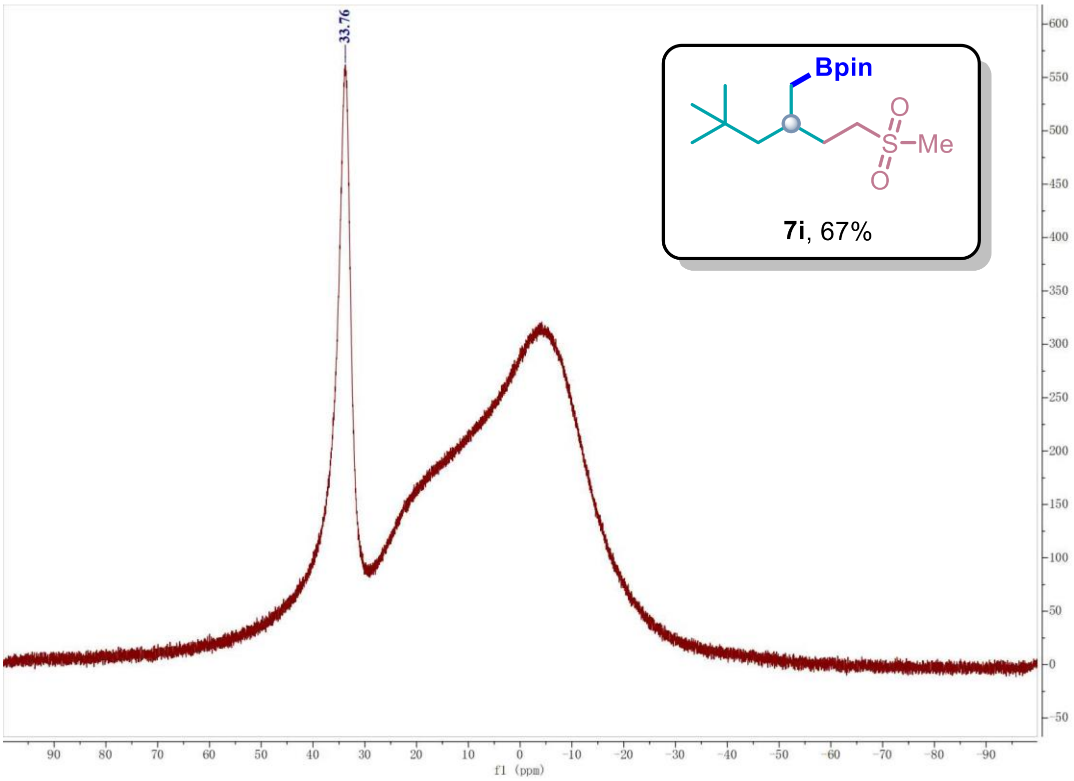

2-(4,4-dimethyl-2-(2-(phenylsulfonyl)ethyl)pentyl)-4,4,5,5-tetramethyl-1,3,2-dioxaborolane (7j)

^{1}H NMR (500 MHz, Chloroform-*d*)

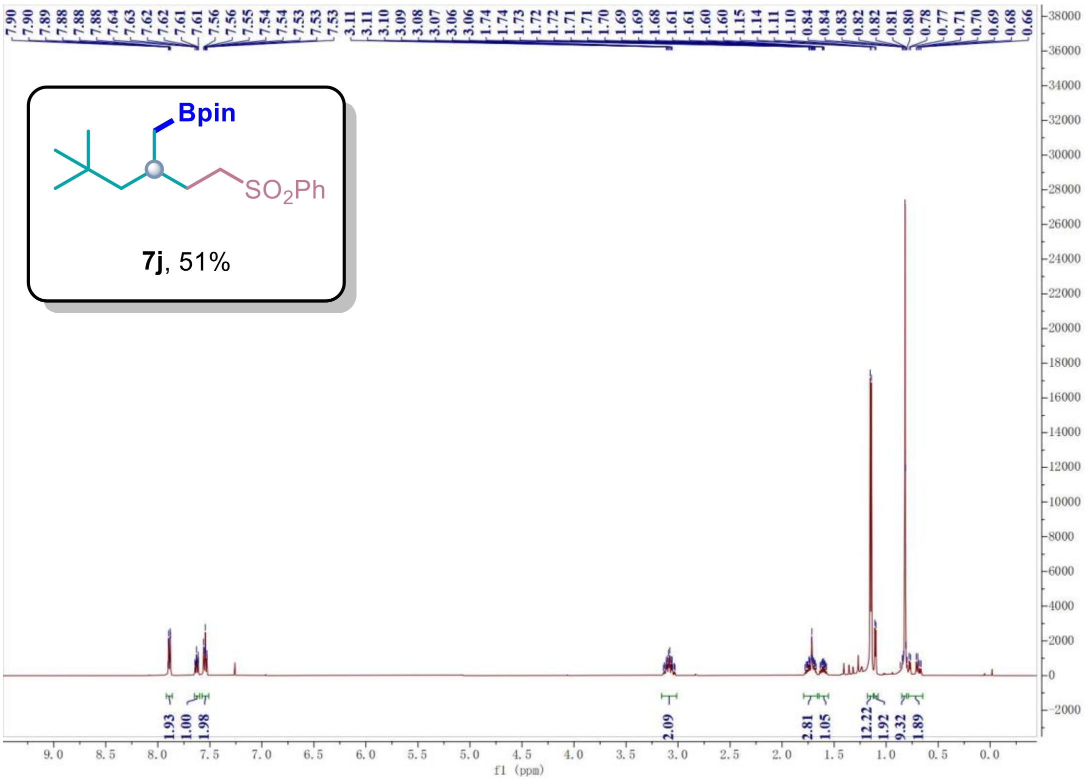

^{13}C NMR (126 MHz, Chloroform-*d*)

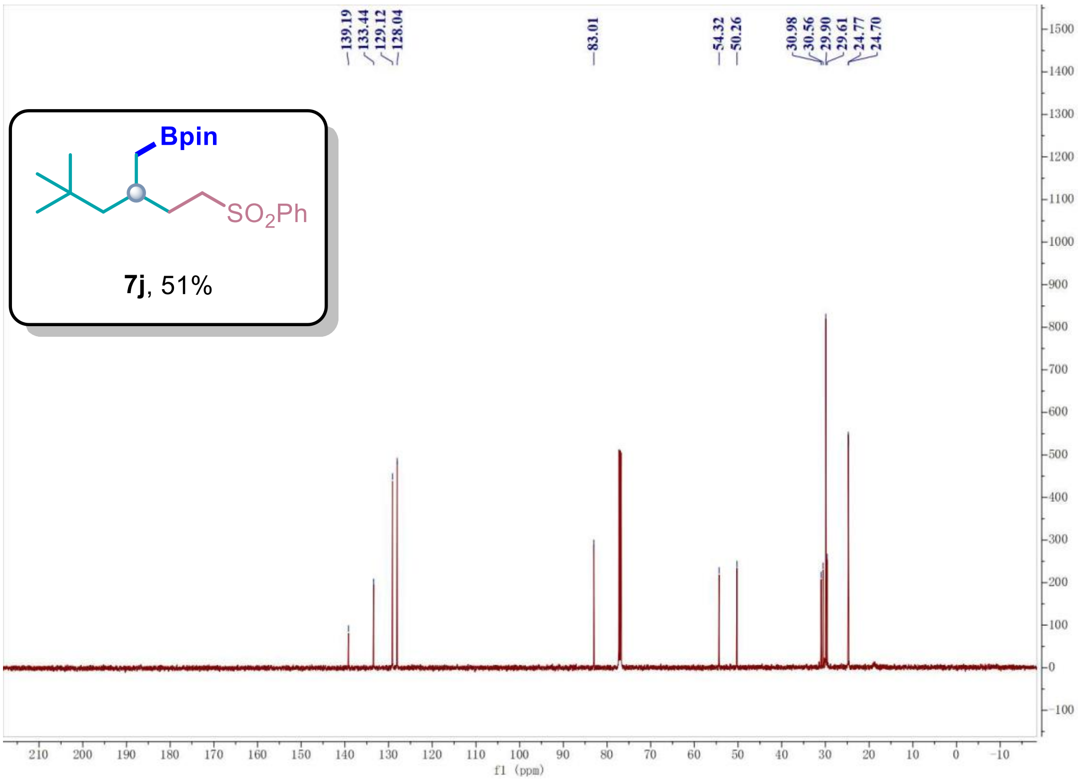

^{11}B NMR (160 MHz, Chloroform-*d*)

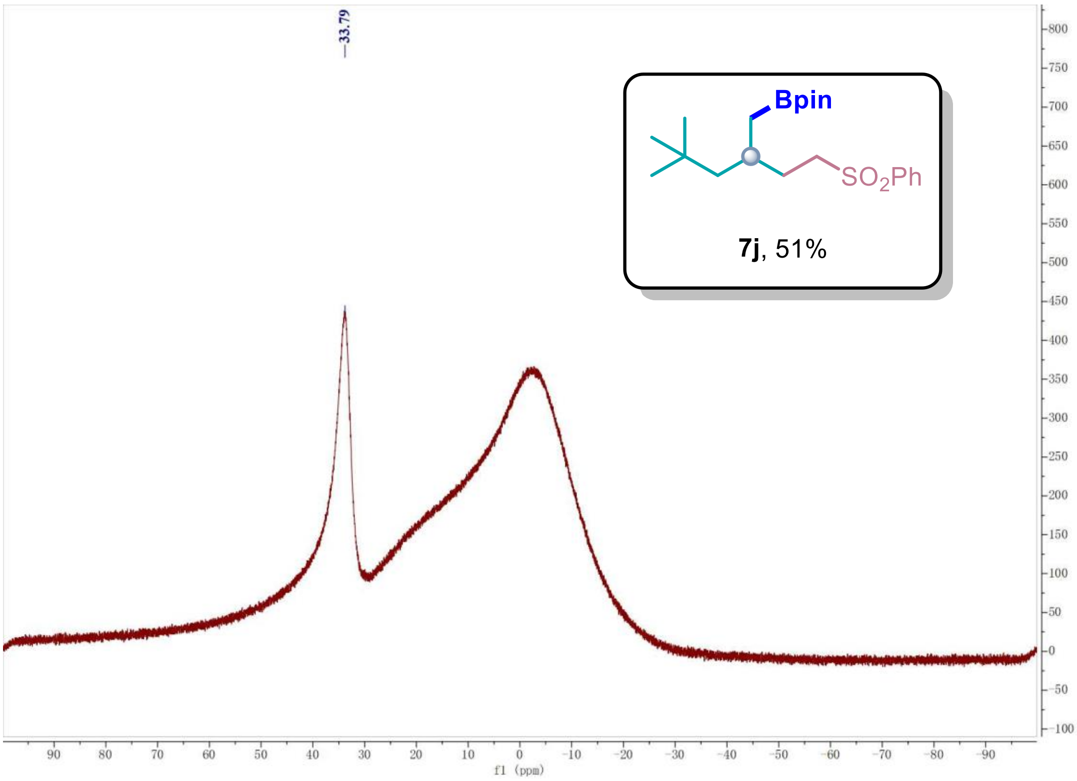

2-methoxyethyl 6,6-dimethyl-4-((4,4,5,5-tetramethyl-1,3,2-dioxaborolan-2-yl)methyl)heptanoate (7k)

^{1}H NMR (500 MHz, Chloroform-*d*)

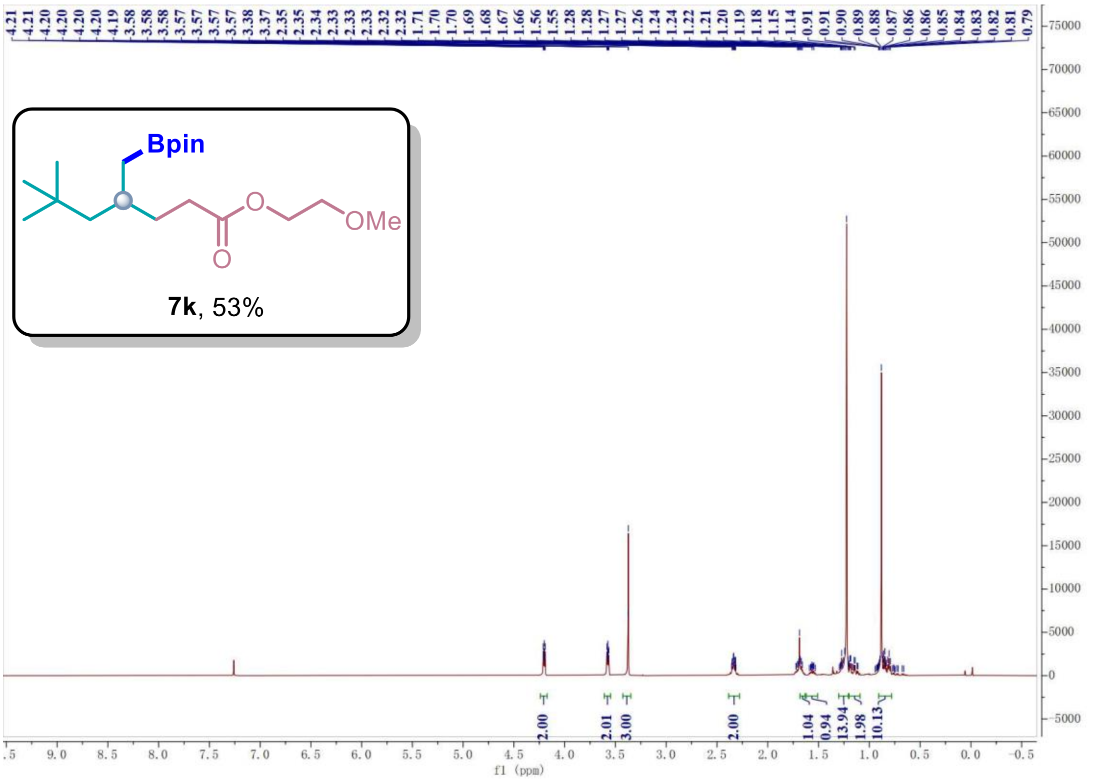

^{13}C NMR (126 MHz, Chloroform-*d*)

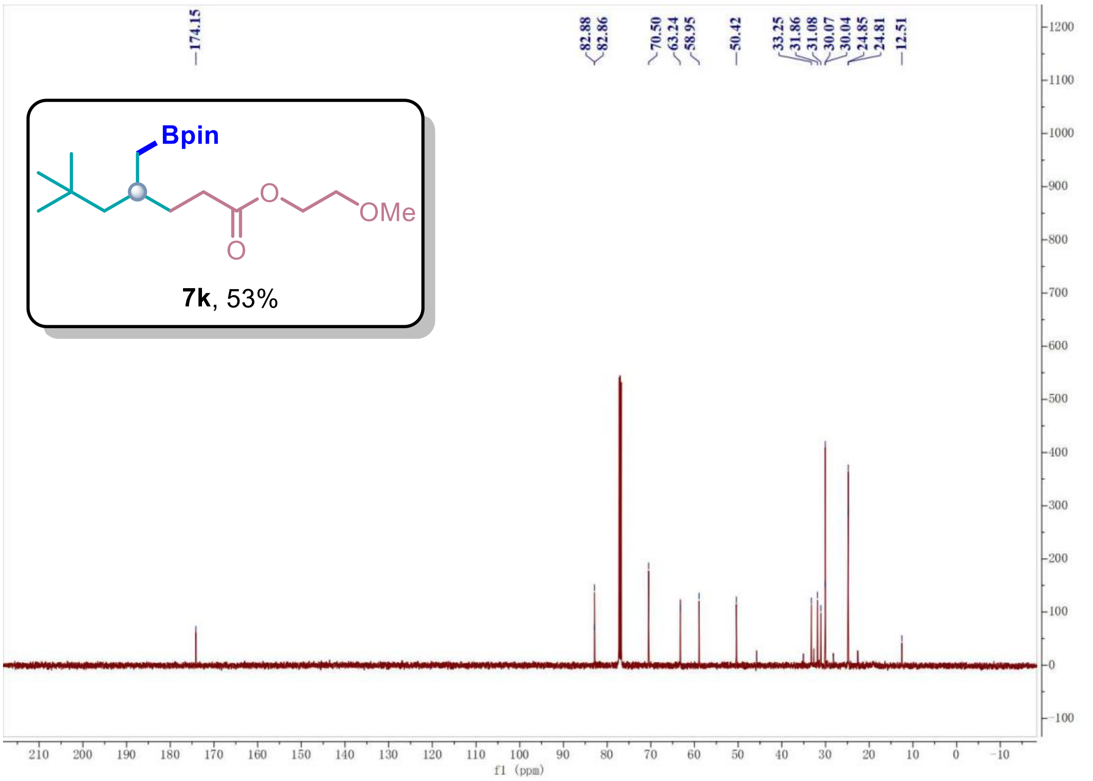

^{11}B NMR (160 MHz, Chloroform-*d*)

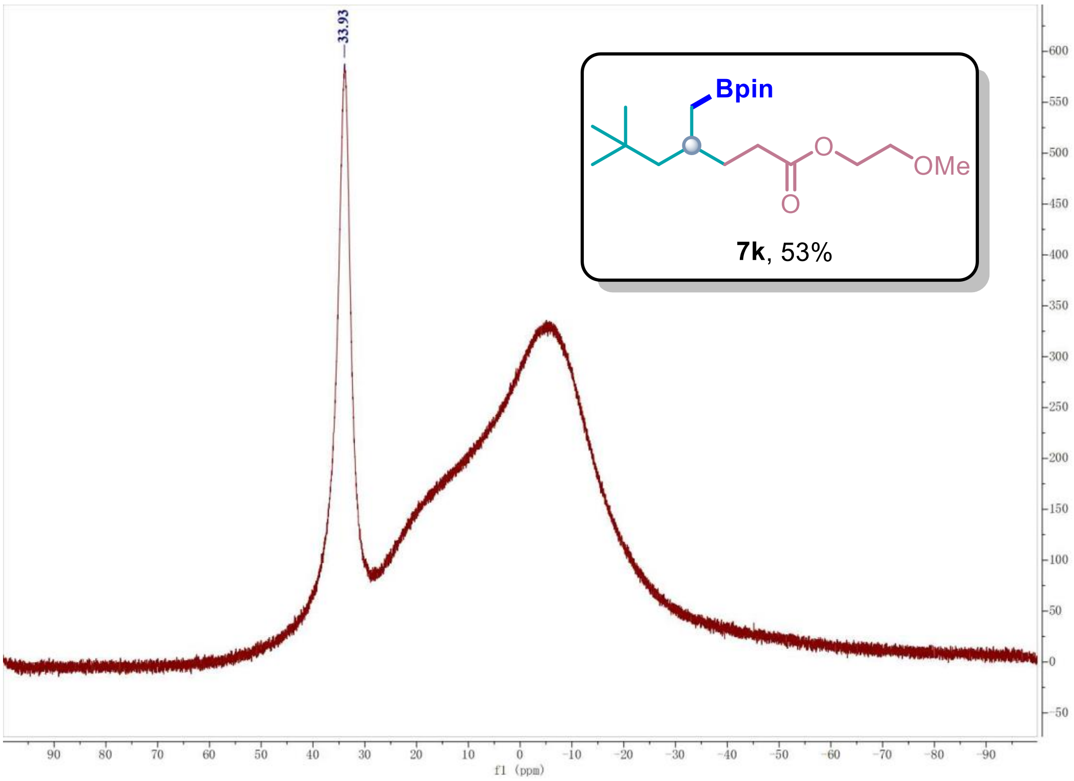

(tetrahydrofuran-2-yl)methyl 6,6-dimethyl-4-((4,4,5,5-tetramethyl-1,3,2-dioxaborolan-2-yl)methyl)heptanoate (7l)

^{1}H NMR (500 MHz, Chloroform-*d*)

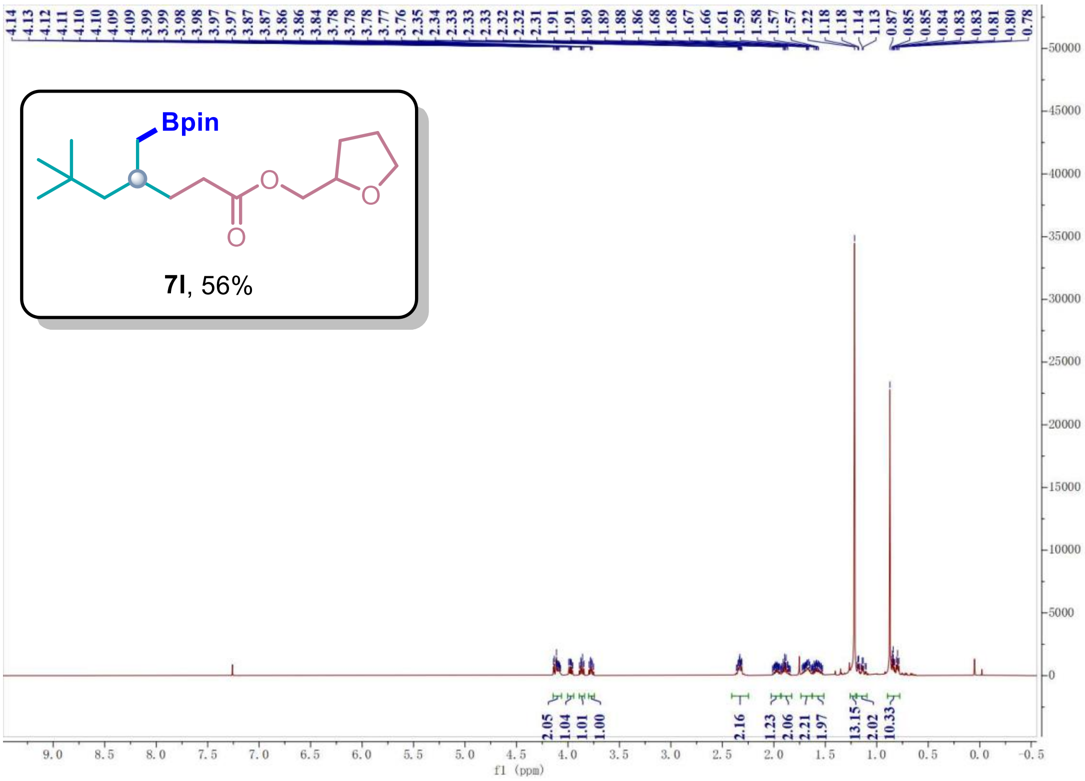

^{13}C NMR (126 MHz, Chloroform-*d*)

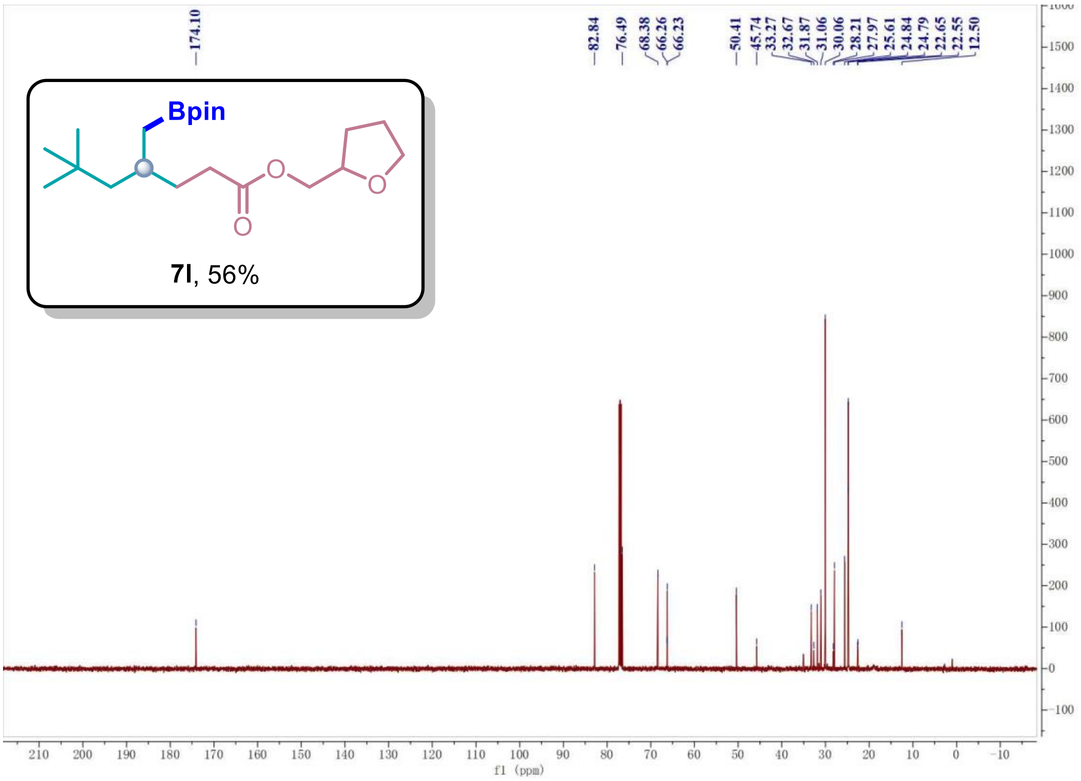

^{11}B NMR (160 MHz, Chloroform-*d*)

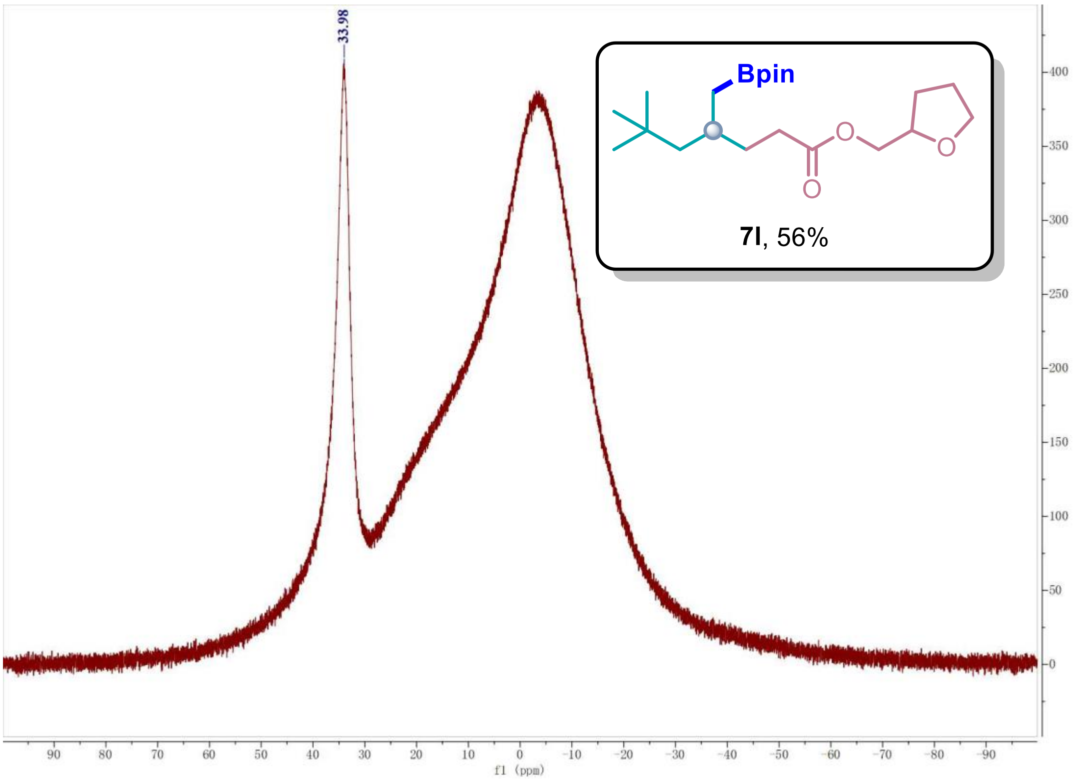

cyclopropylmethyl 6,6-dimethyl-4-((4,4,5,5-tetramethyl-1,3,2-dioxaborolan-2-yl)methyl)heptanoate (7m)

^{1}H NMR (500 MHz, Chloroform-*d*)

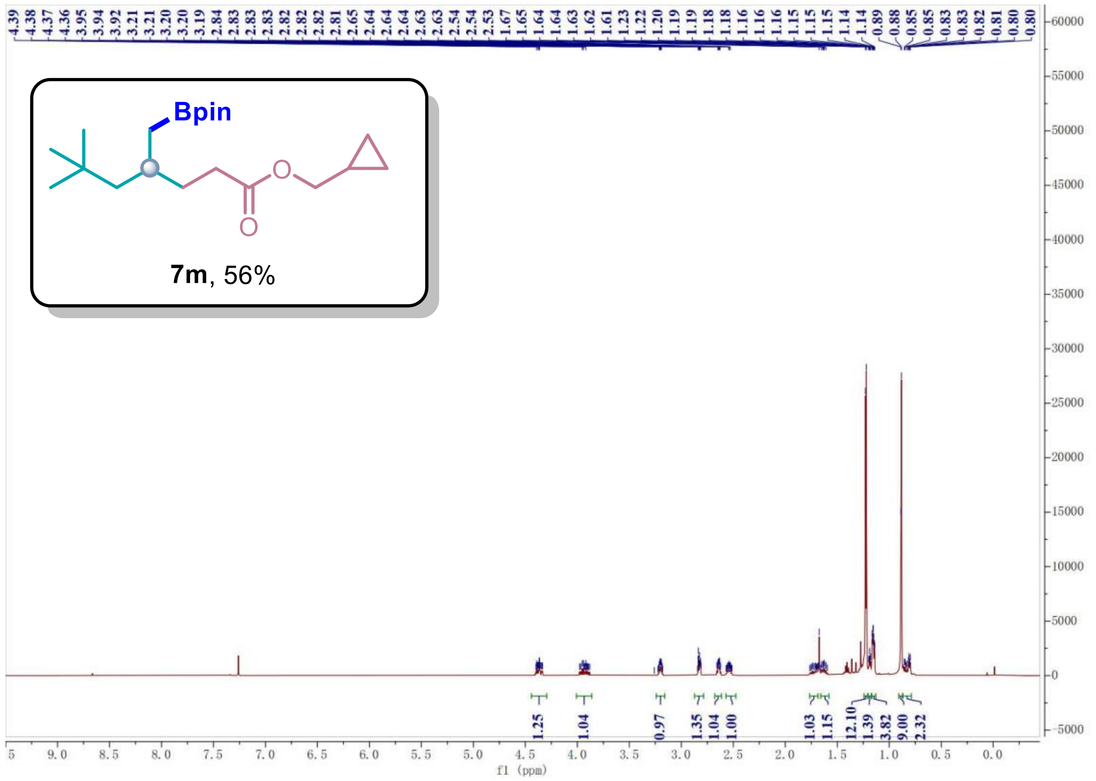

^{13}C NMR (126 MHz, Chloroform-*d*)

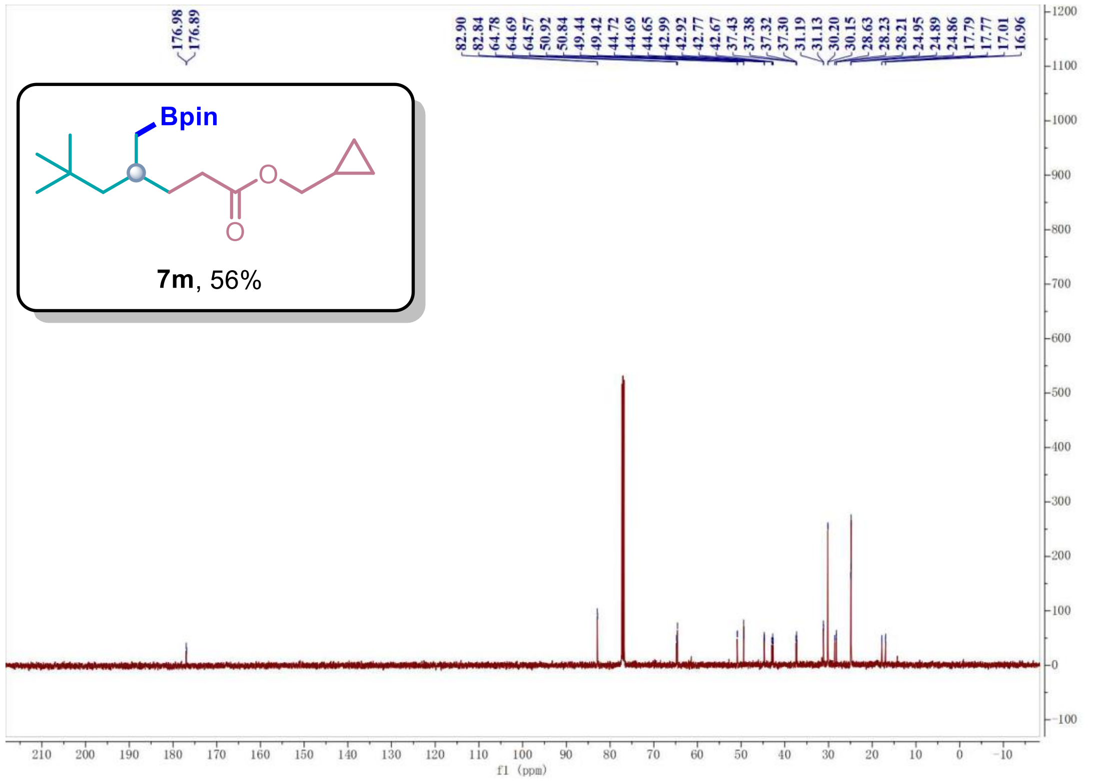

^{11}B NMR (160 MHz, Chloroform-*d*)

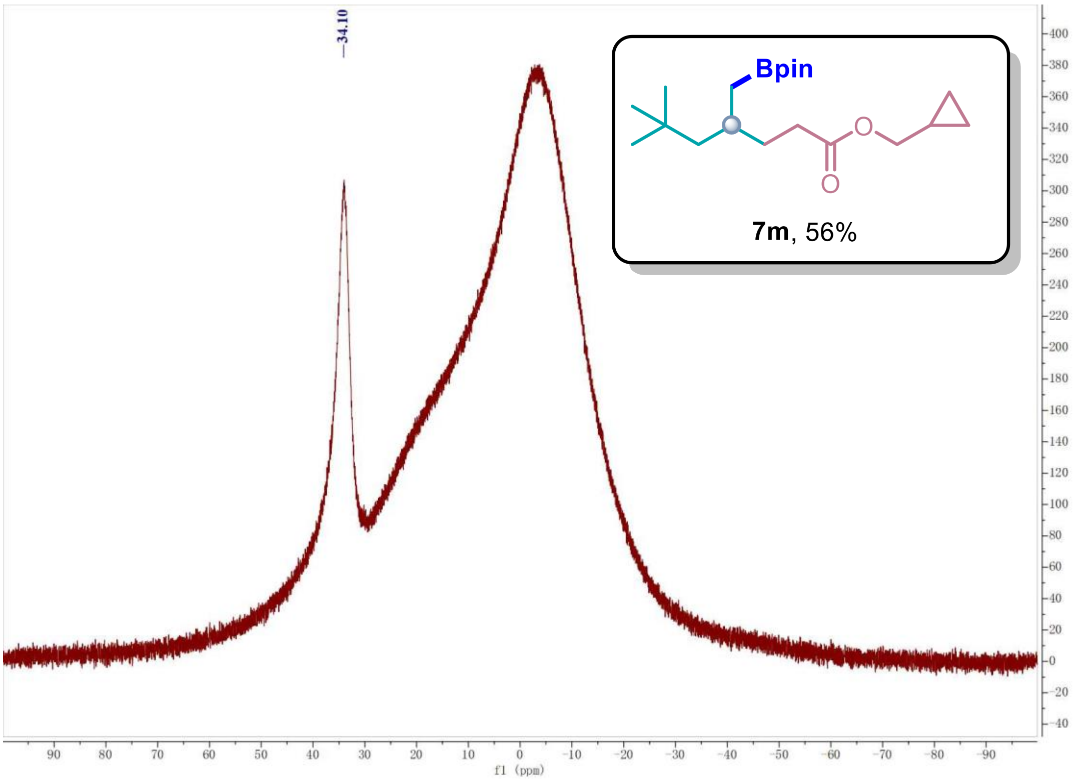

ethyl 4-methyl-5-(4,4,5,5-tetramethyl-1,3,2-dioxaborolan-2-yl)pentanoate (7n)

^{1}H NMR (500 MHz, Chloroform-*d*)

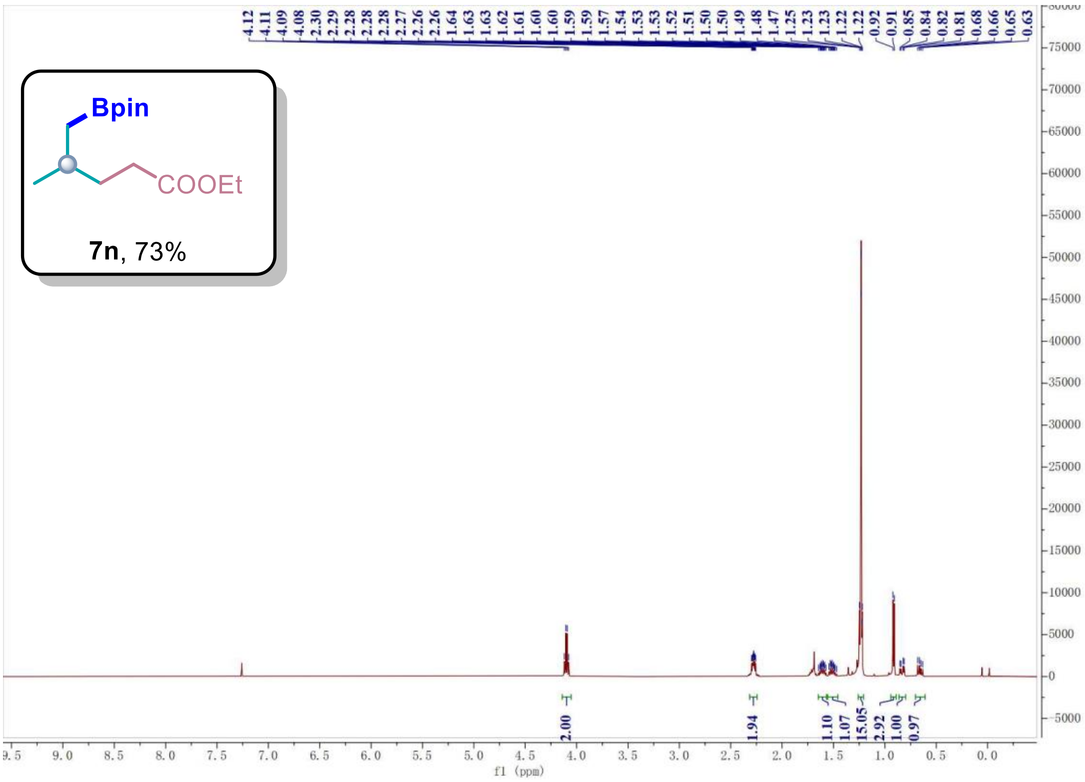

^{13}C NMR (126 MHz, Chloroform-*d*)

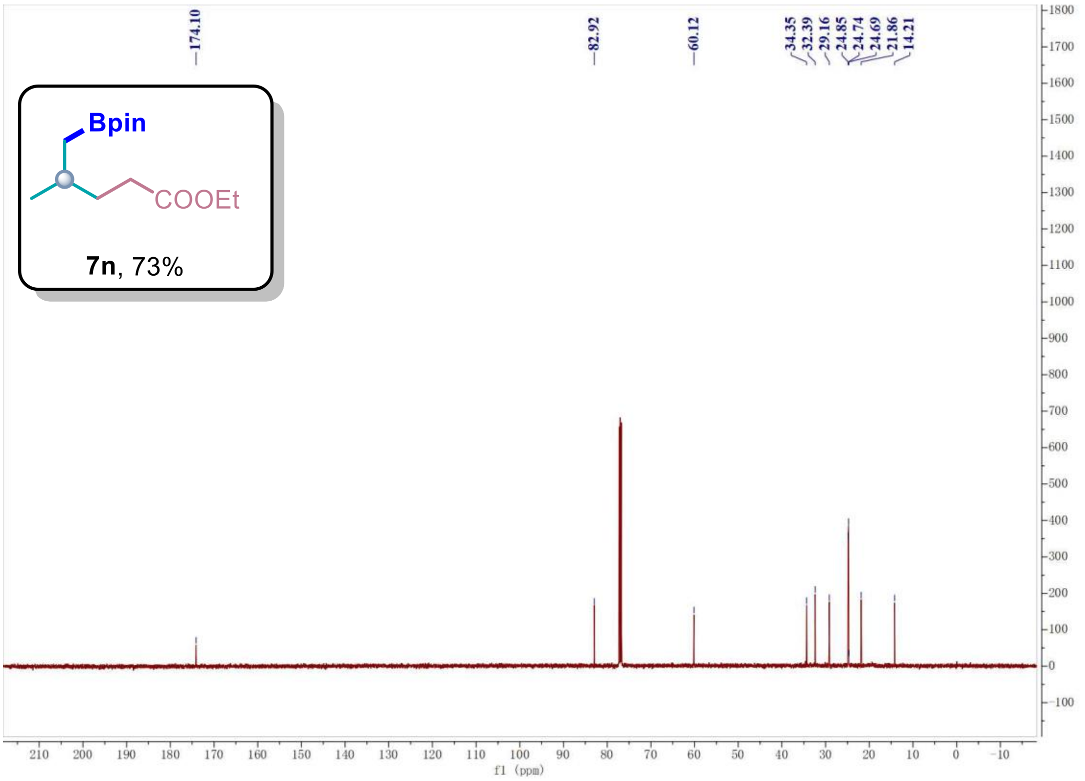

^{11}B NMR (160 MHz, Chloroform-*d*)

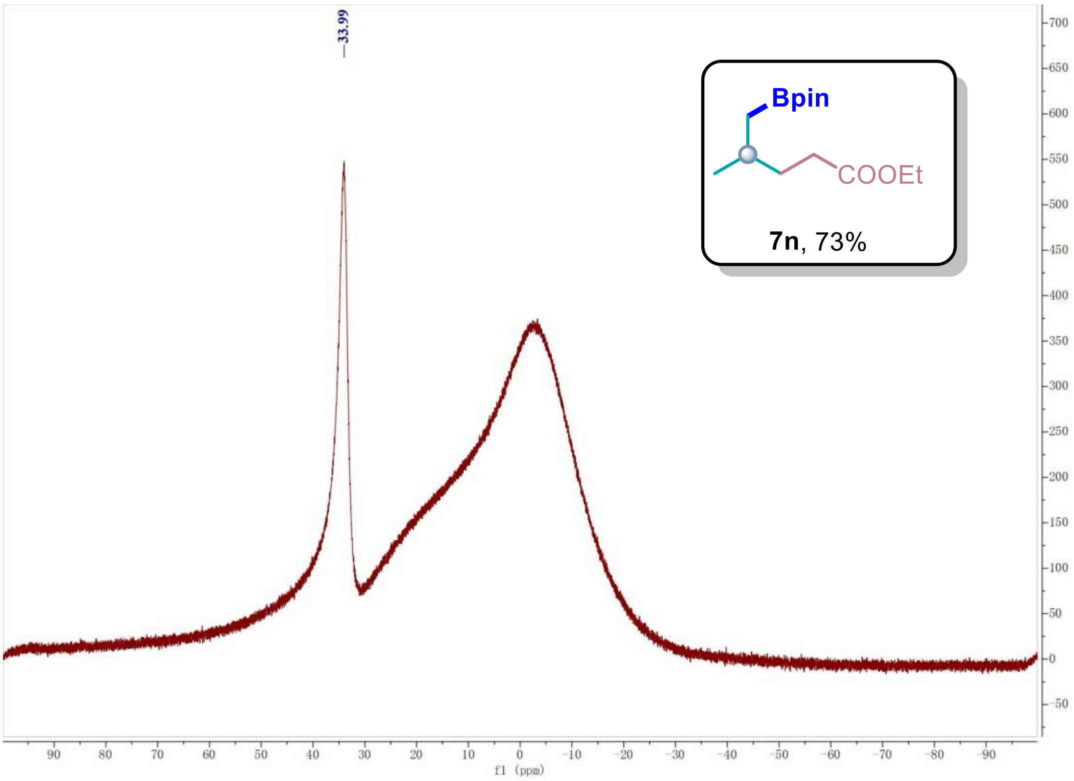

ethyl 4-((4,4,5,5-tetramethyl-1,3,2-dioxaborolan-2-yl)methyl)heptanoate (7o)

^{1}H NMR (500 MHz, Chloroform-*d*)

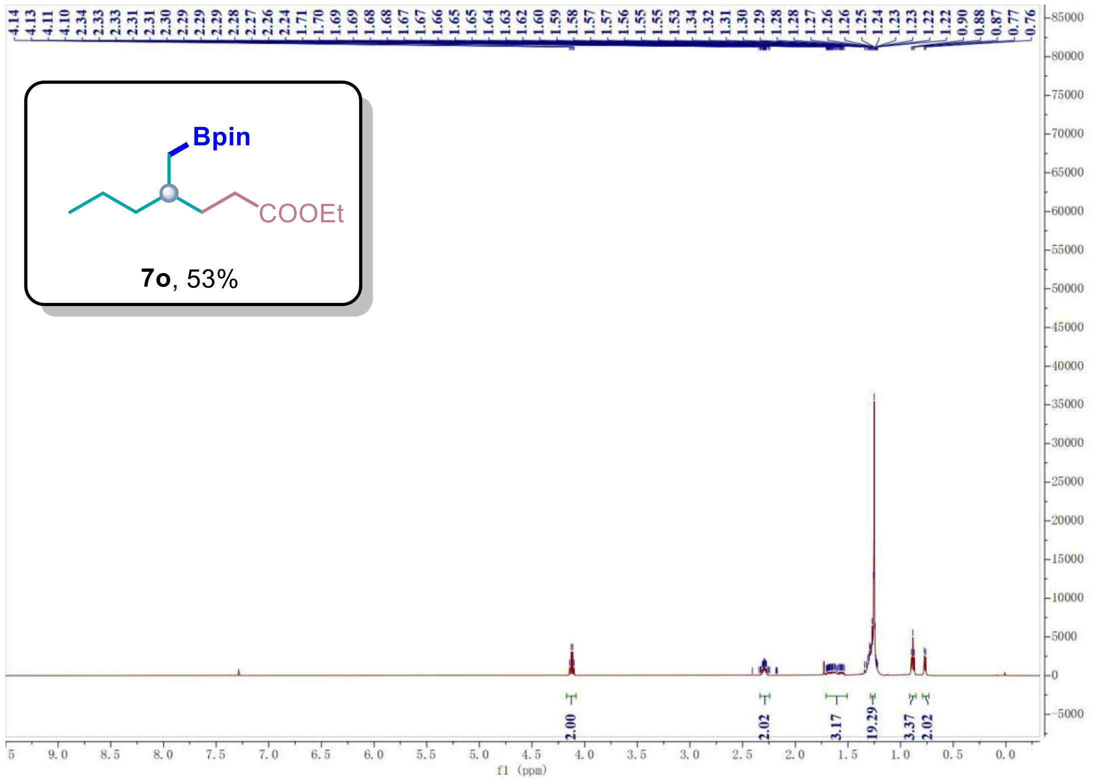

^{13}C NMR (126 MHz, Chloroform-*d*)

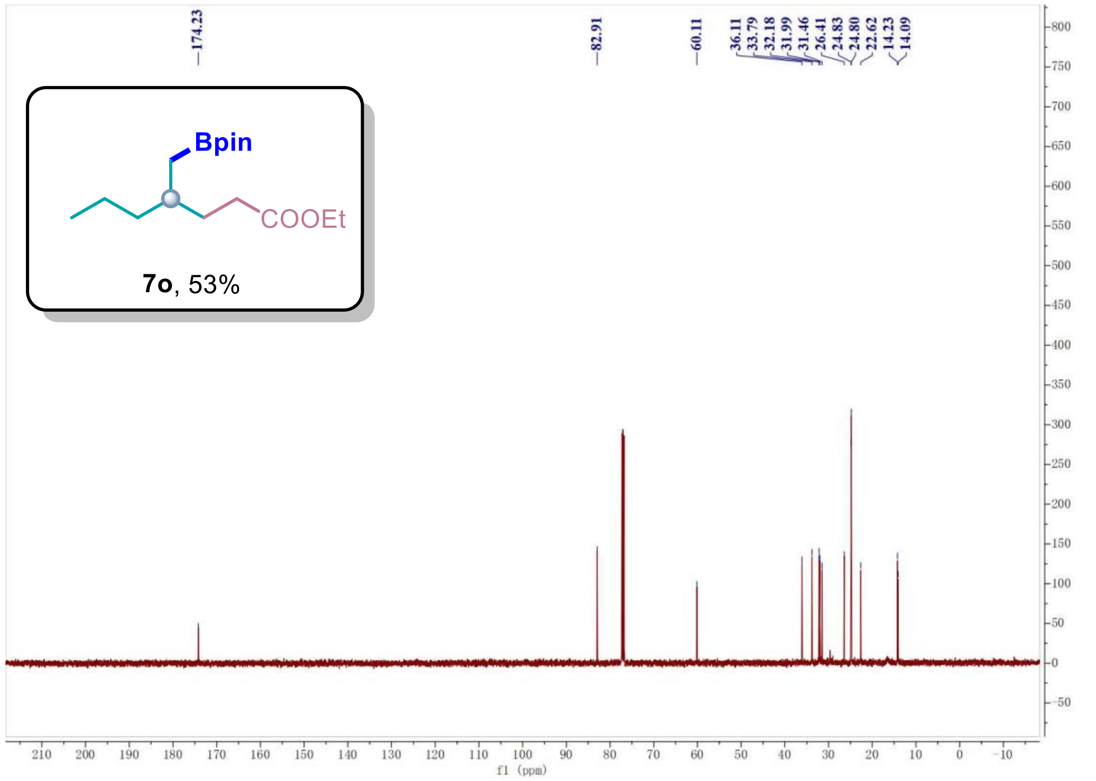

^{11}B NMR (160 MHz, Chloroform-*d*)

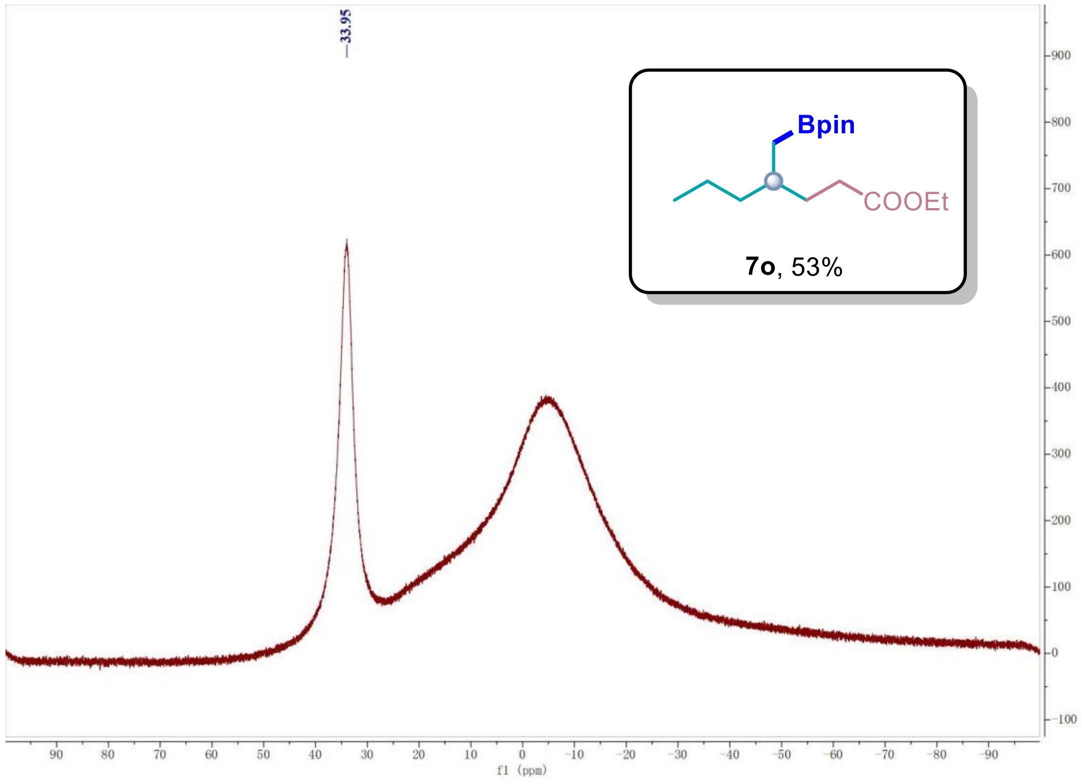

ethyl 6-methyl-4-((4,4,5,5-tetramethyl-1,3,2-dioxaborolan-2-yl)methyl)heptanoate (7p)

^{1}H NMR (500 MHz, Chloroform-*d*)

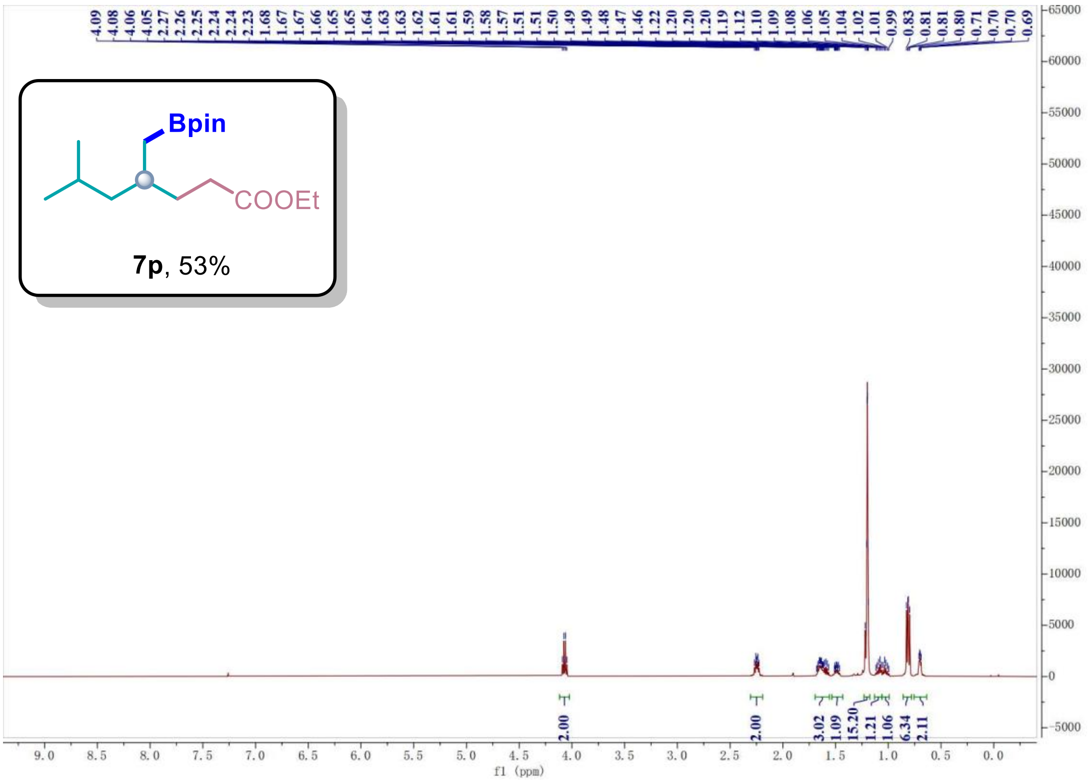

^{13}C NMR (126 MHz, Chloroform-*d*)

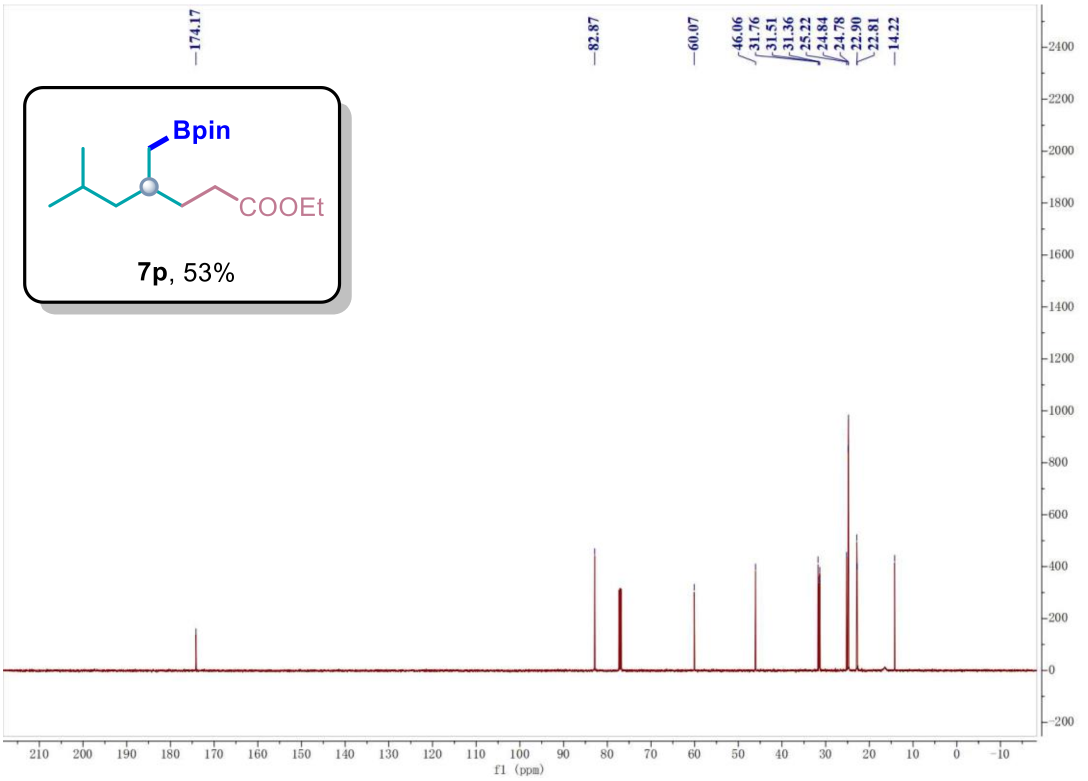

^{11}B NMR (160 MHz, Chloroform-*d*)

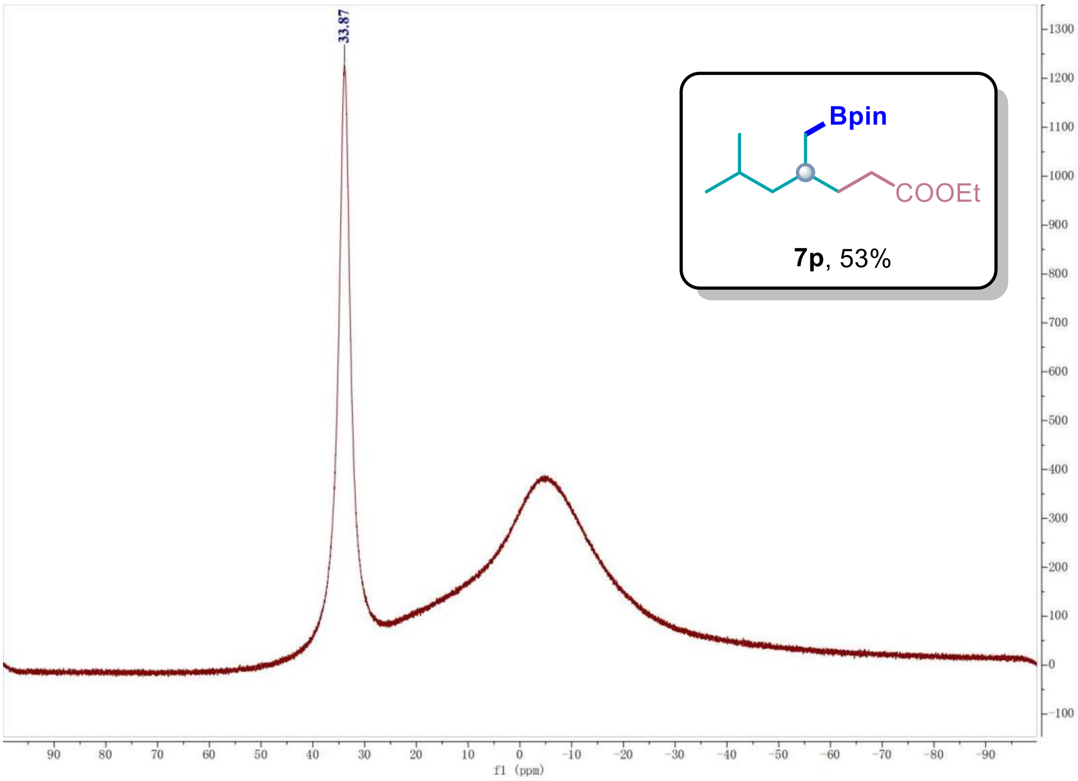

ethyl 6-phenyl-4-((4,4,5,5-tetramethyl-1,3,2-dioxaborolan-2-yl)methyl)hexanoate (7q)

^{1}H NMR (500 MHz, Chloroform-*d*)

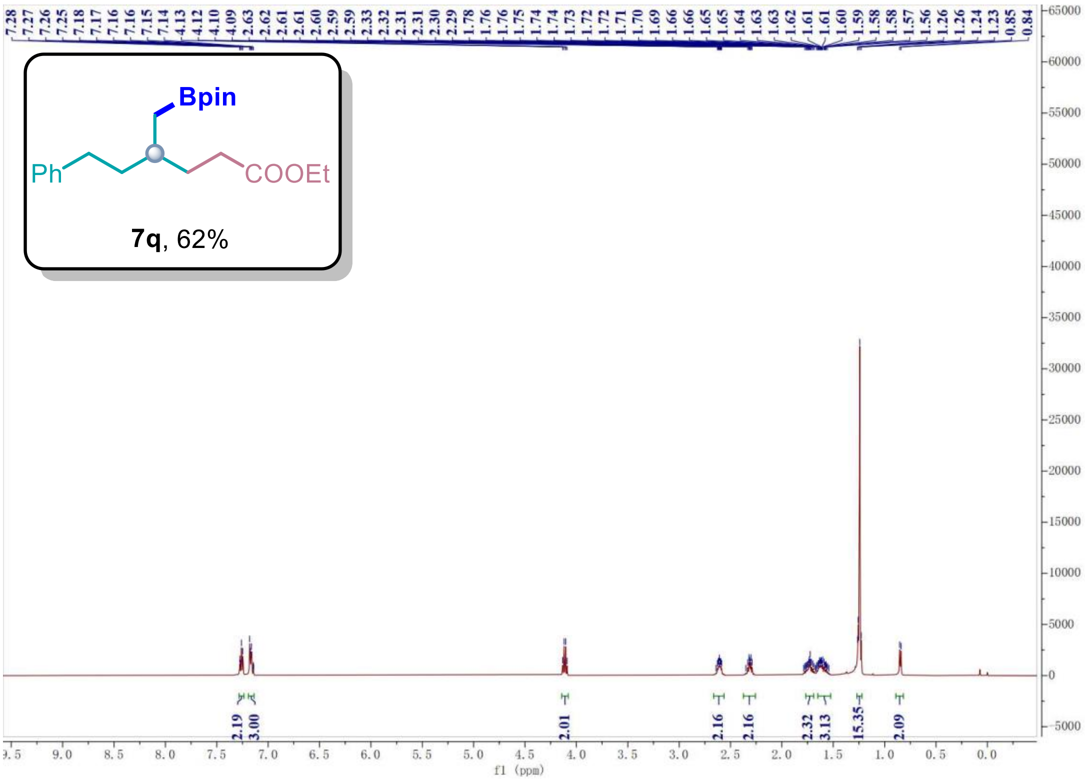

^{13}C NMR (126 MHz, Chloroform-*d*)

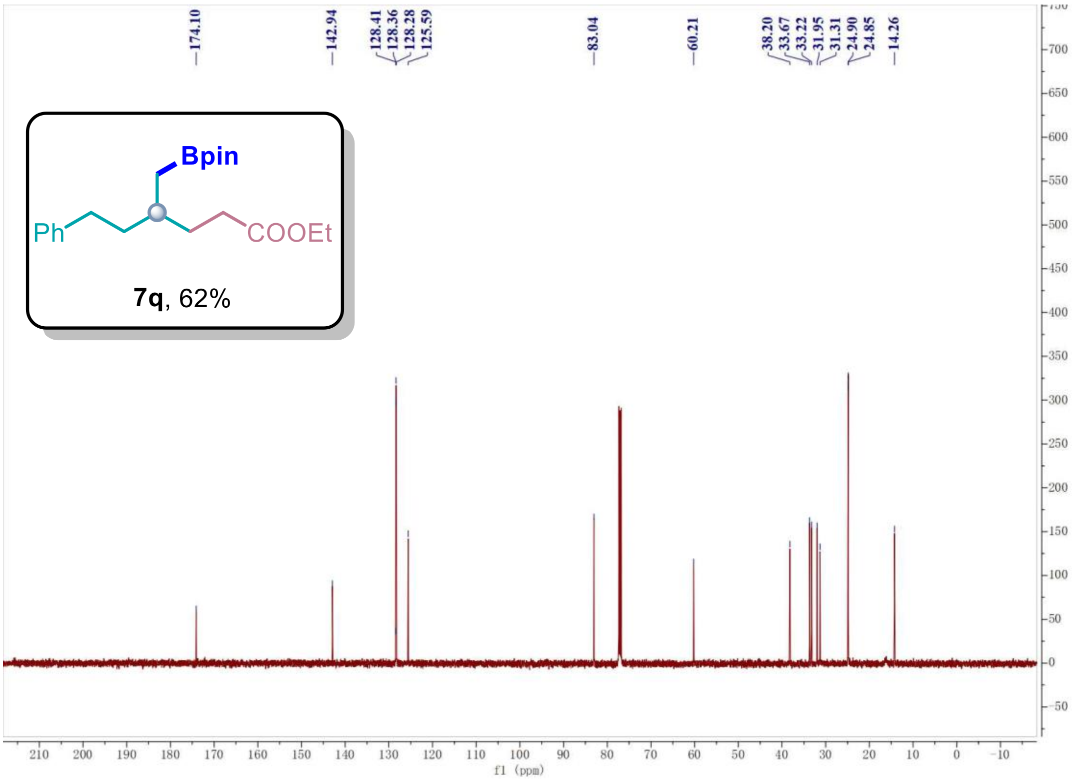

^{11}B NMR (160 MHz, Chloroform-*d*)

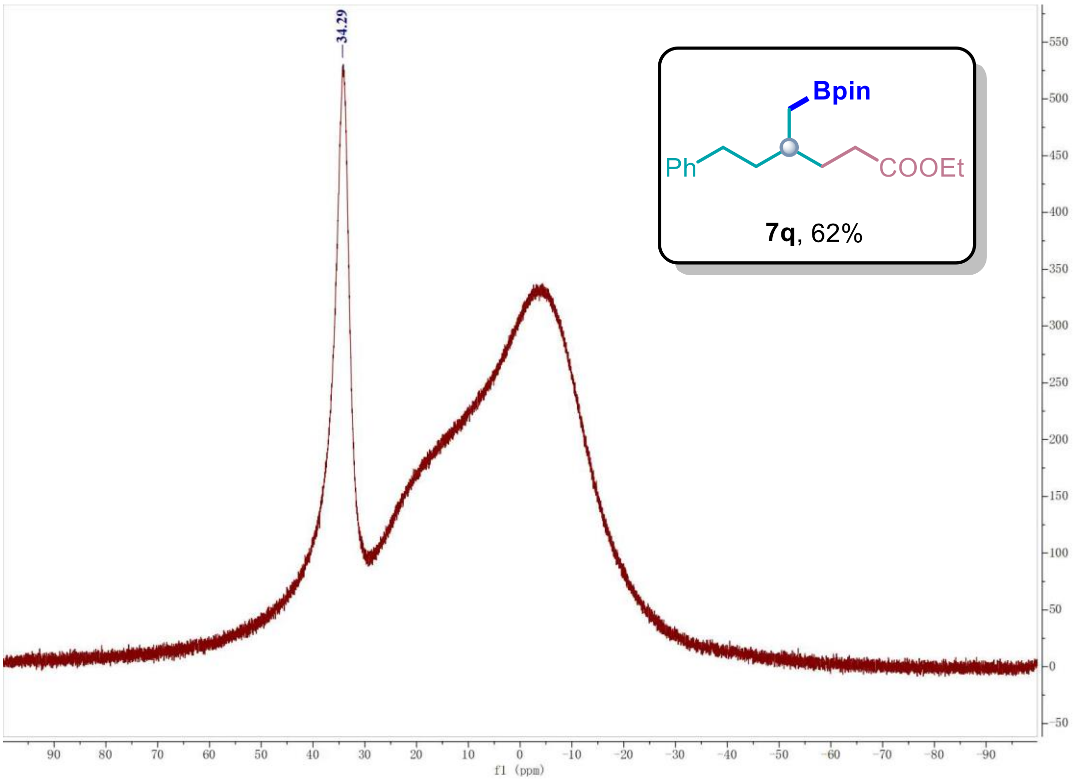

ethyl 5-ethyl-4-((4,4,5,5-tetramethyl-1,3,2-dioxaborolan-2-yl)methyl)heptanoate (7r)

^{1}H NMR (500 MHz, Chloroform-*d*)

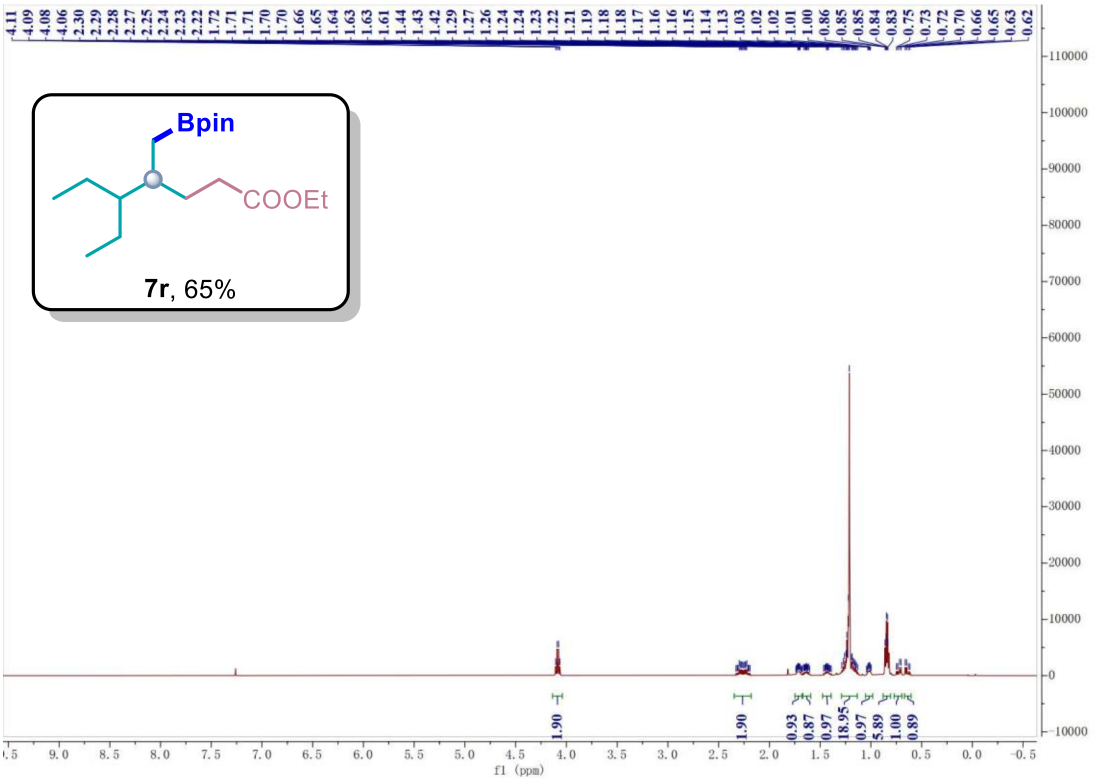

^{13}C NMR (126 MHz, Chloroform-*d*)

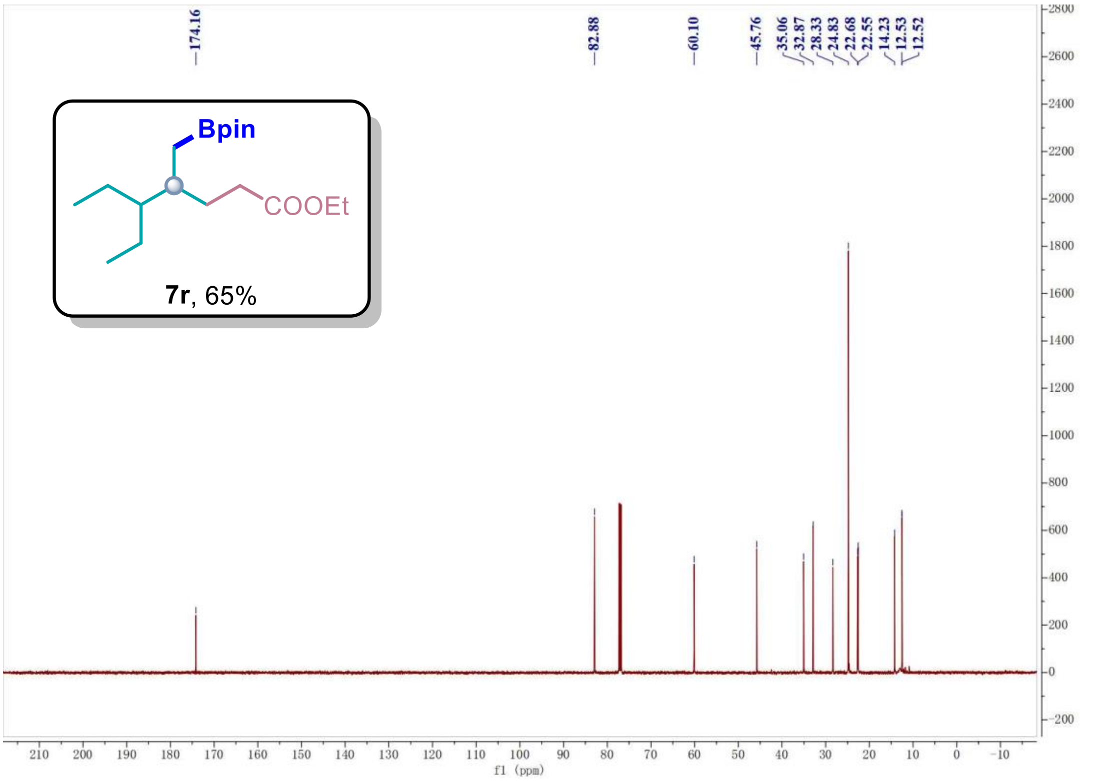

^{11}B NMR (160 MHz, Chloroform-*d*)

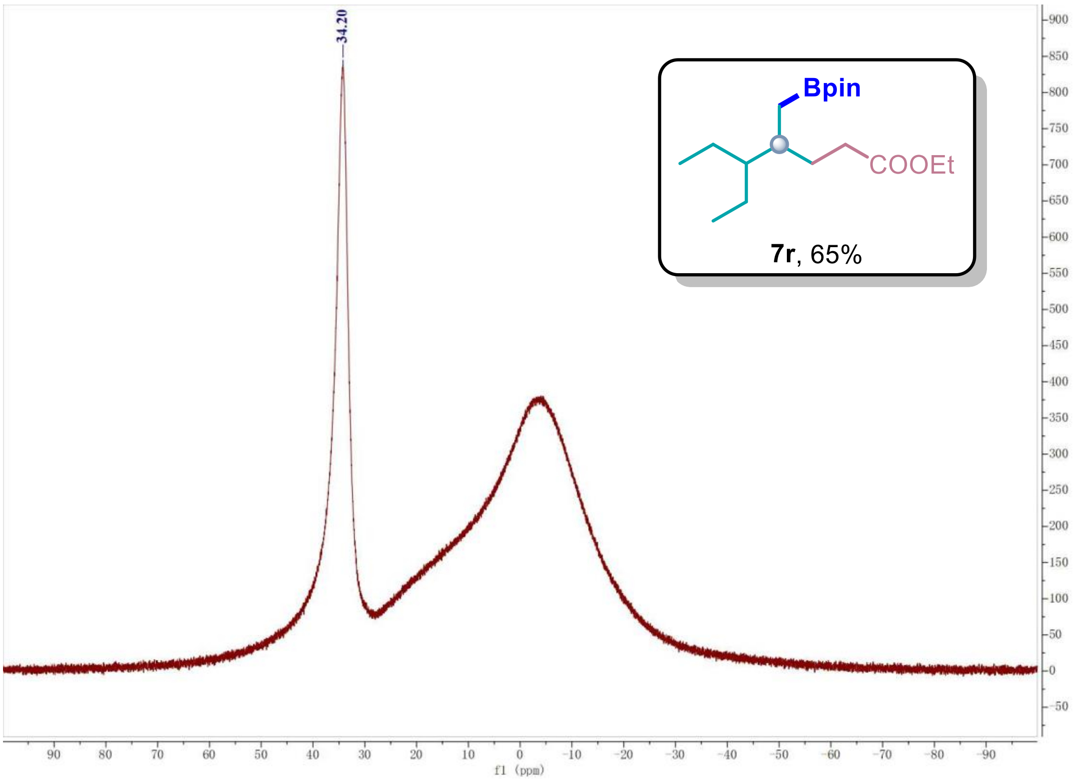

ethyl 4-cyclopentyl-5-(4,4,5,5-tetramethyl-1,3,2-dioxaborolan-2-yl)pentanoate (7s)

^{1}H NMR (500 MHz, Chloroform-*d*)

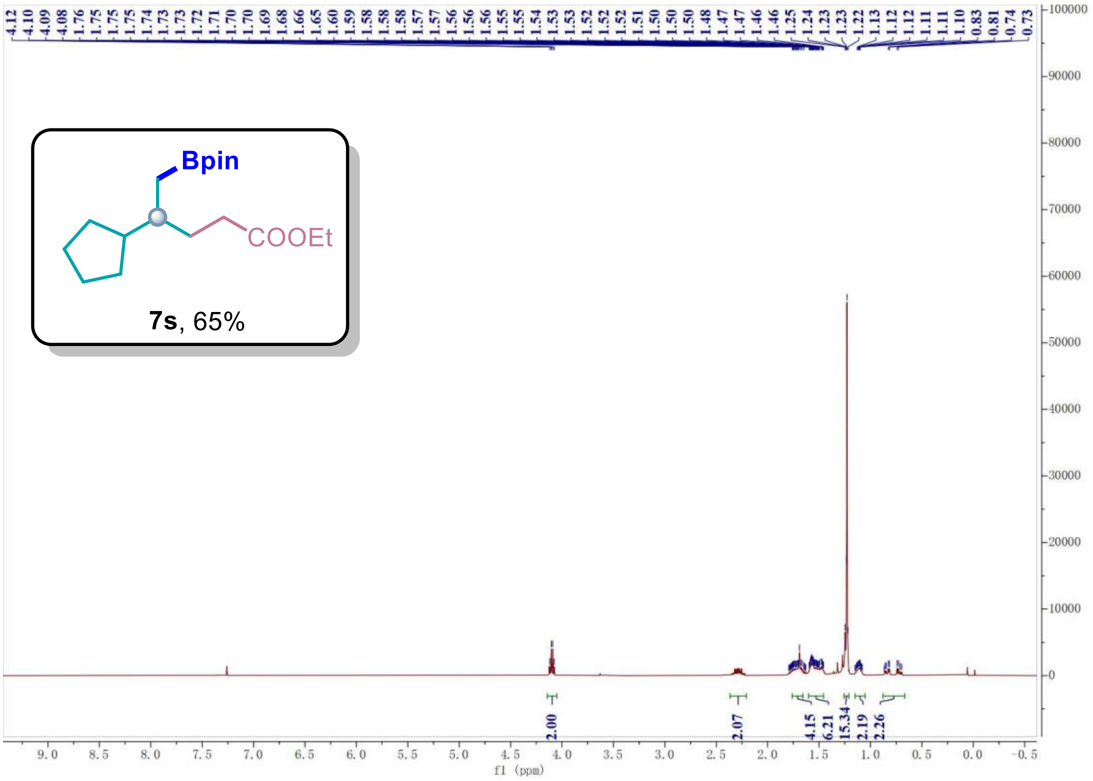

^{13}C NMR (126 MHz, Chloroform-*d*)

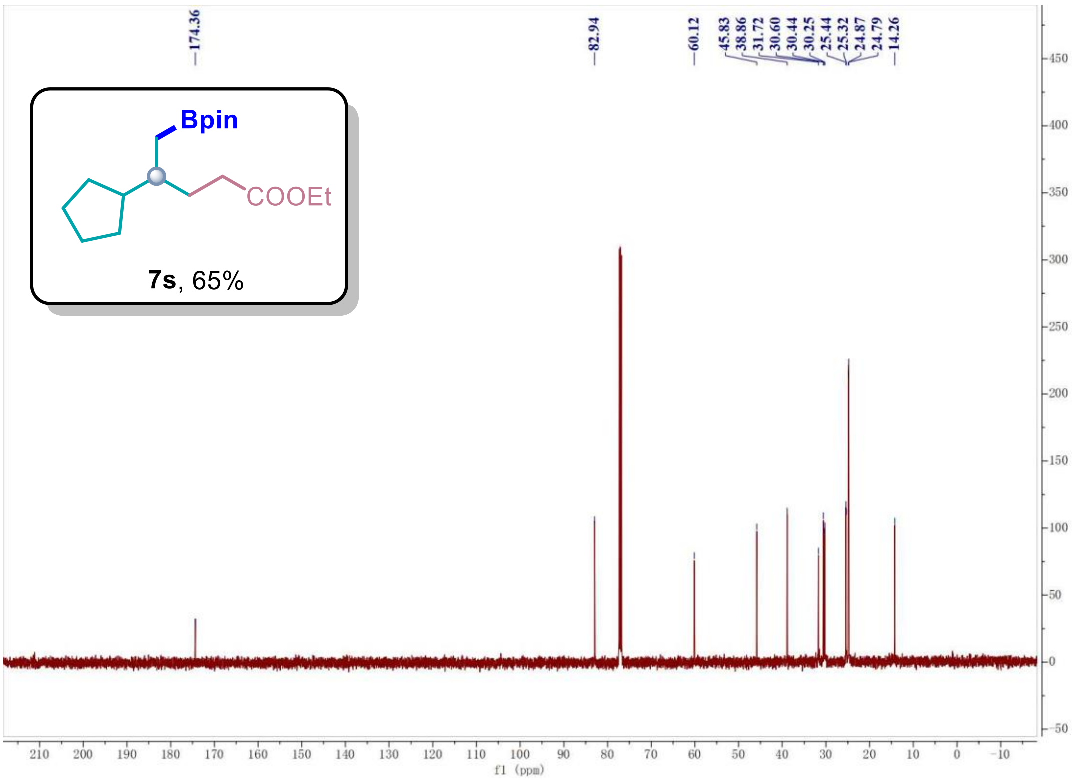

^{11}B NMR (160 MHz, Chloroform-*d*)

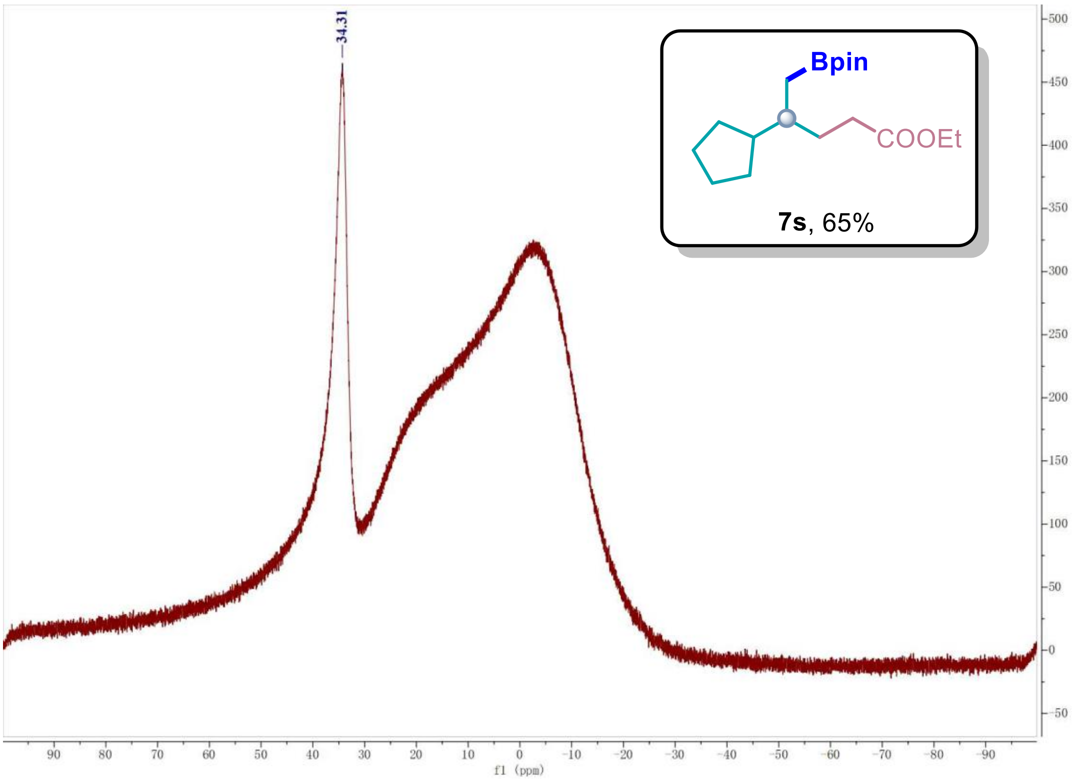

ethyl 4-cyclohexyl-5-(4,4,5,5-tetramethyl-1,3,2-dioxaborolan-2-yl)pentanoate (7t)

^{1}H NMR (500 MHz, Chloroform-*d*)

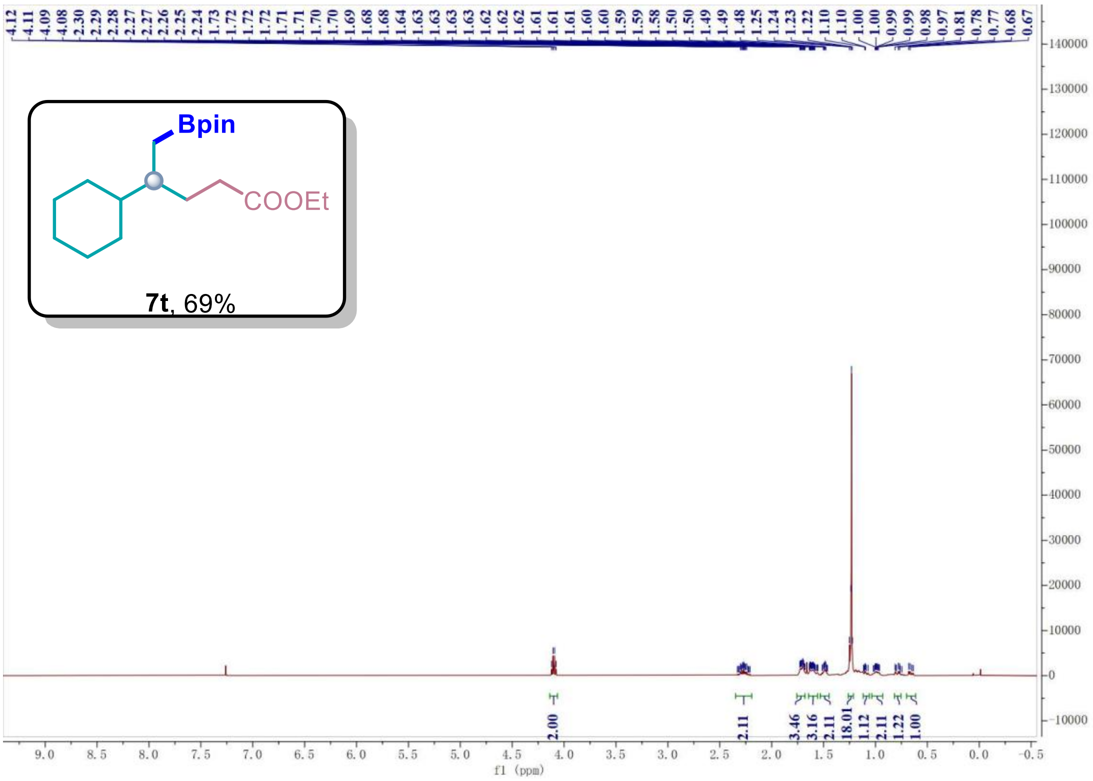

^{13}C NMR (126 MHz, Chloroform-*d*)

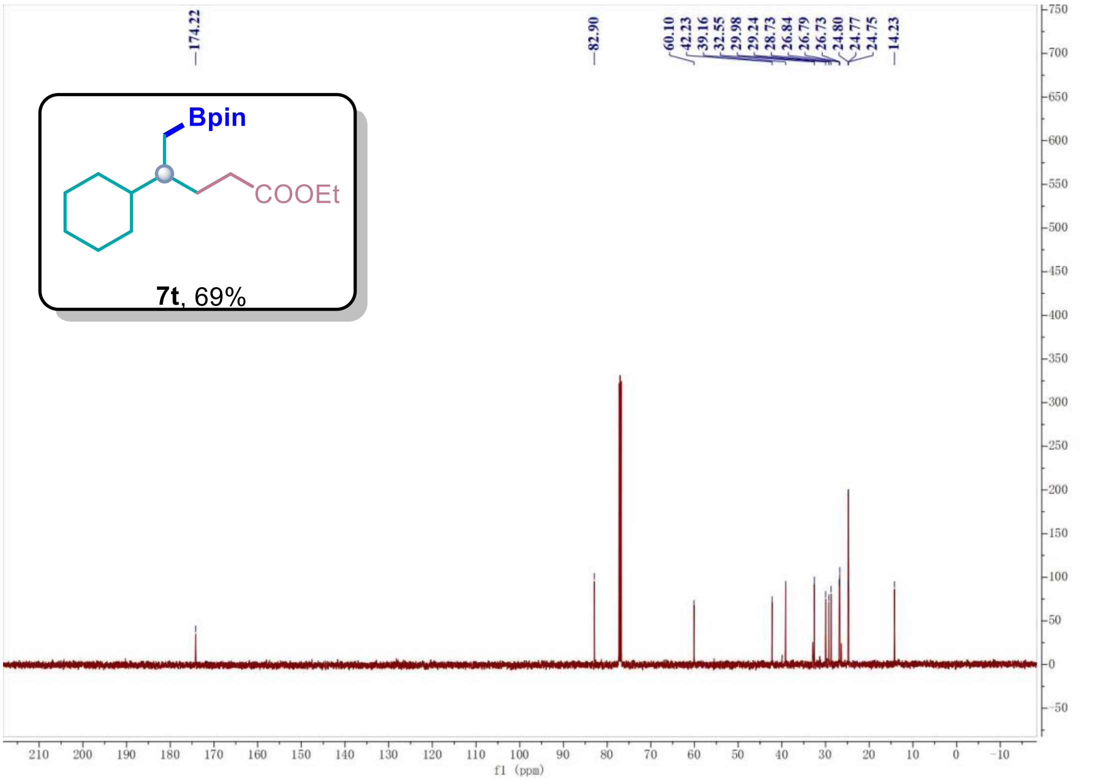

^{11}B NMR (160 MHz, Chloroform-*d*)

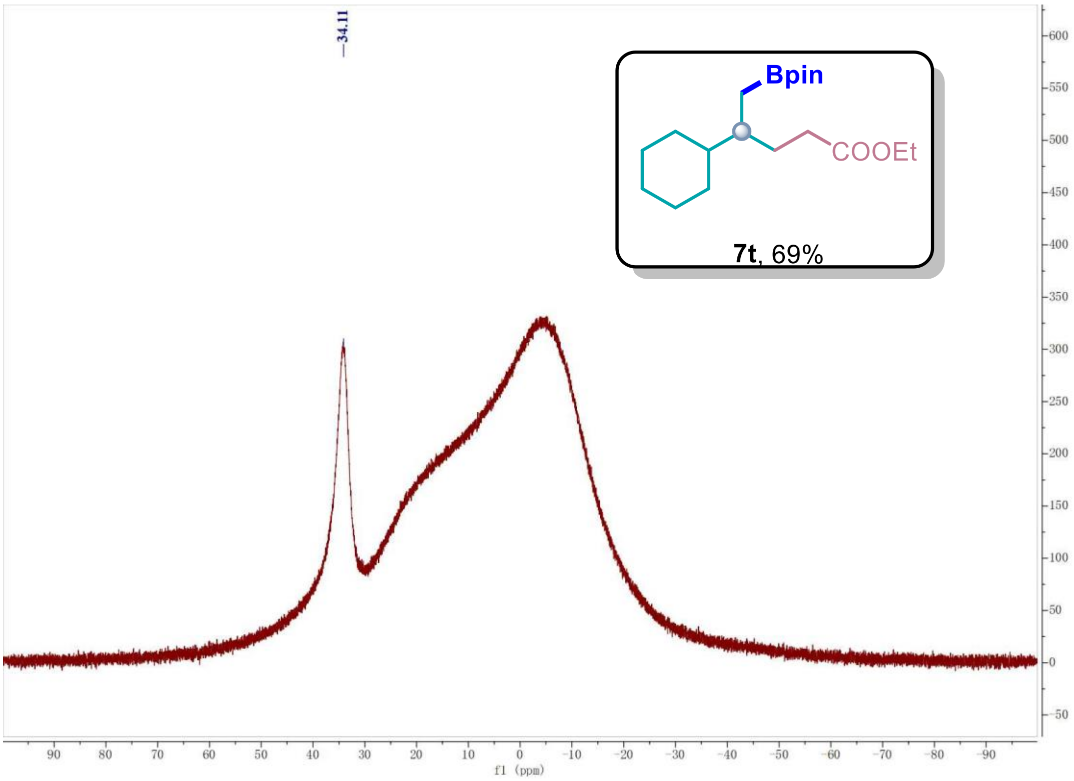

ethyl 5-methyl-4-((4,4,5,5-tetramethyl-1,3,2-dioxaborolan-2-yl)methyl)octanoate (7u)

^{1}H NMR (500 MHz, Chloroform-*d*)

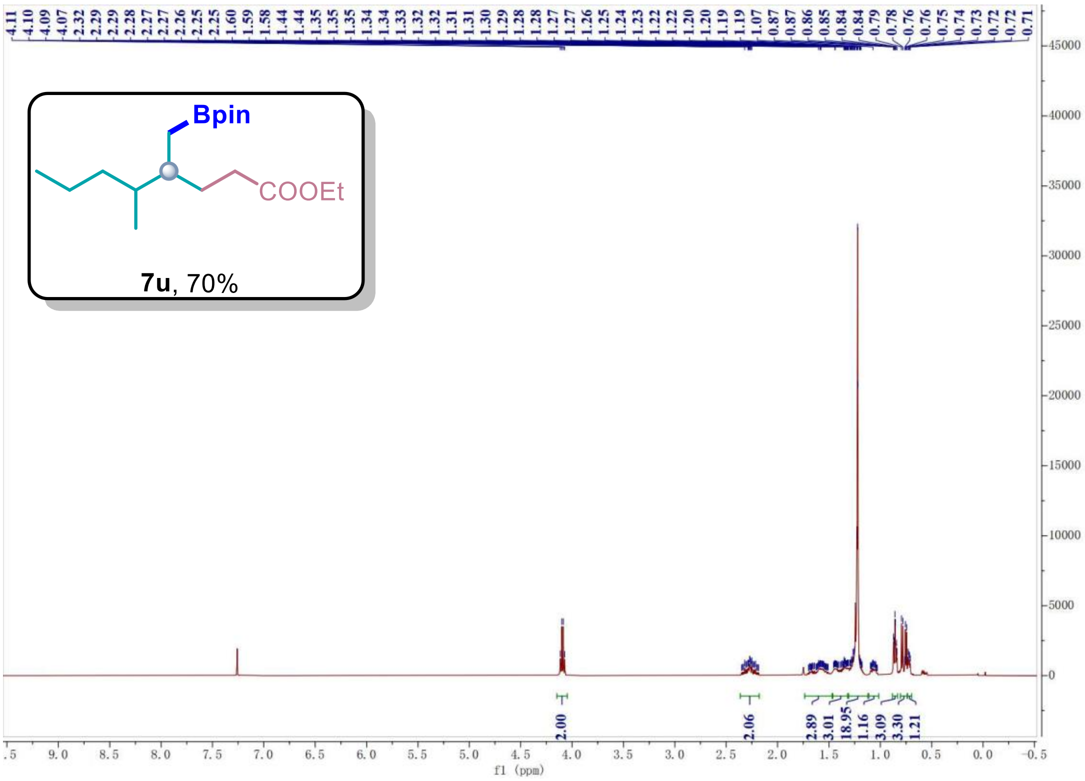

^{13}C NMR (126 MHz, Chloroform-*d*)

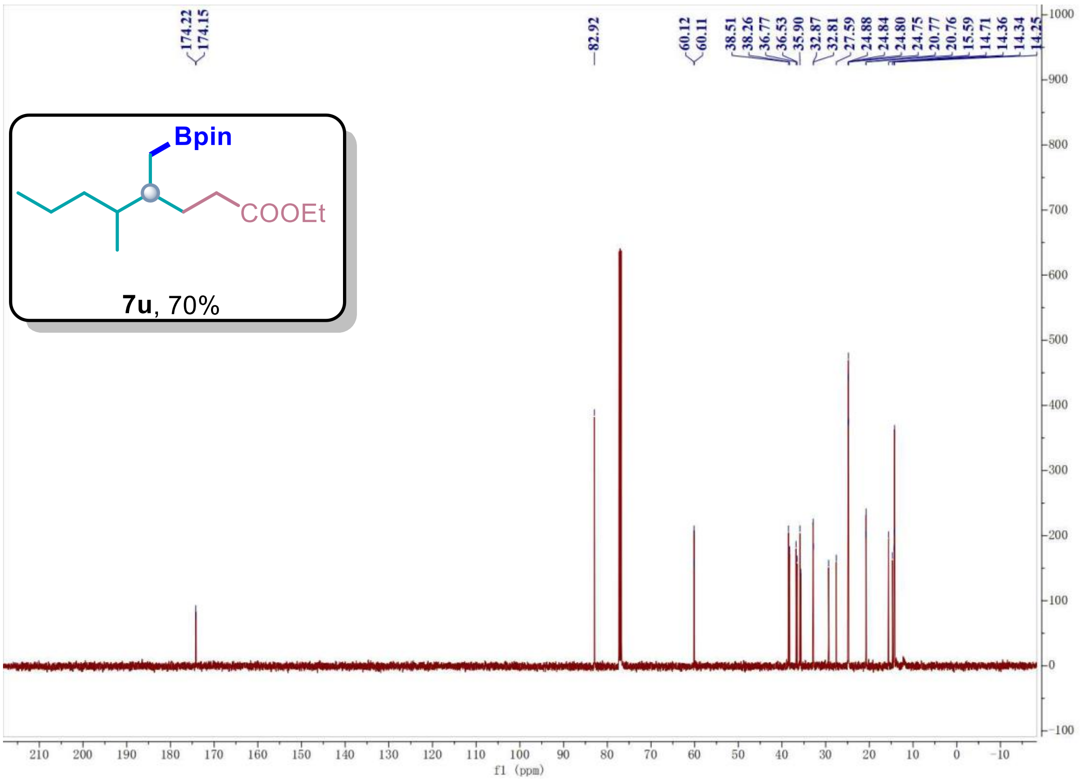

^{11}B NMR (160 MHz, Chloroform-*d*)

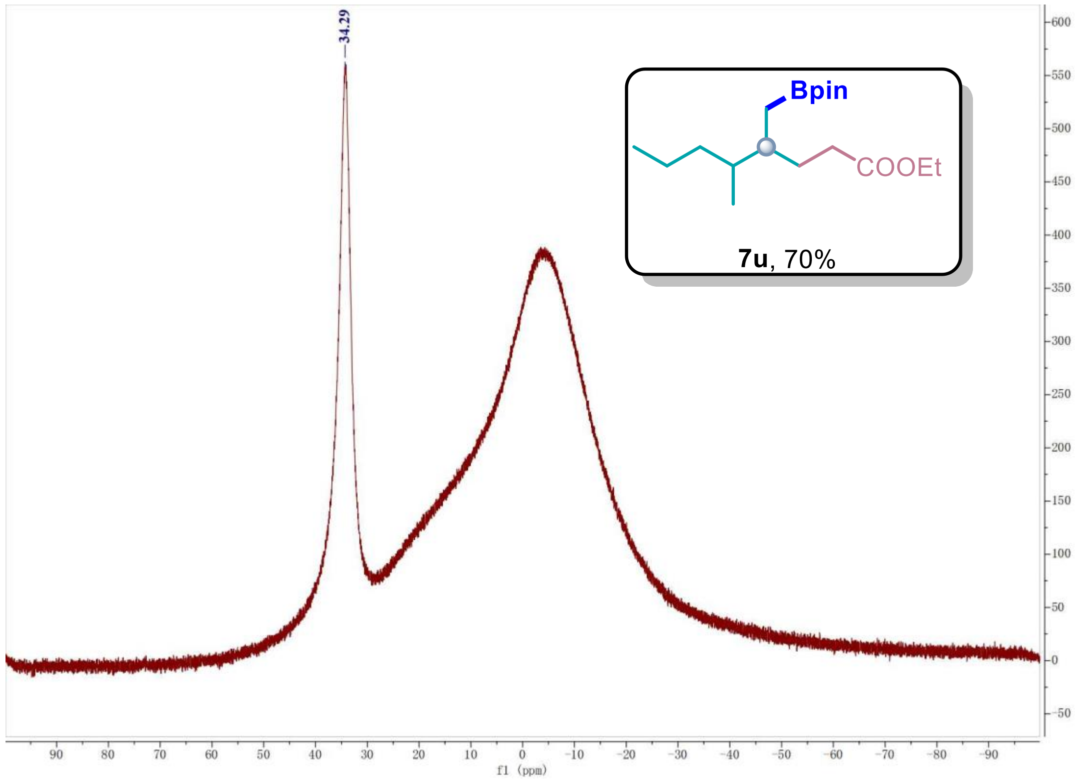

ethyl 3-(1-((4,4,5,5-tetramethyl-1,3,2-dioxaborolan-2-yl)methyl)cyclobutyl)propanoate (7v)

^{1}H NMR (500 MHz, Chloroform-*d*)

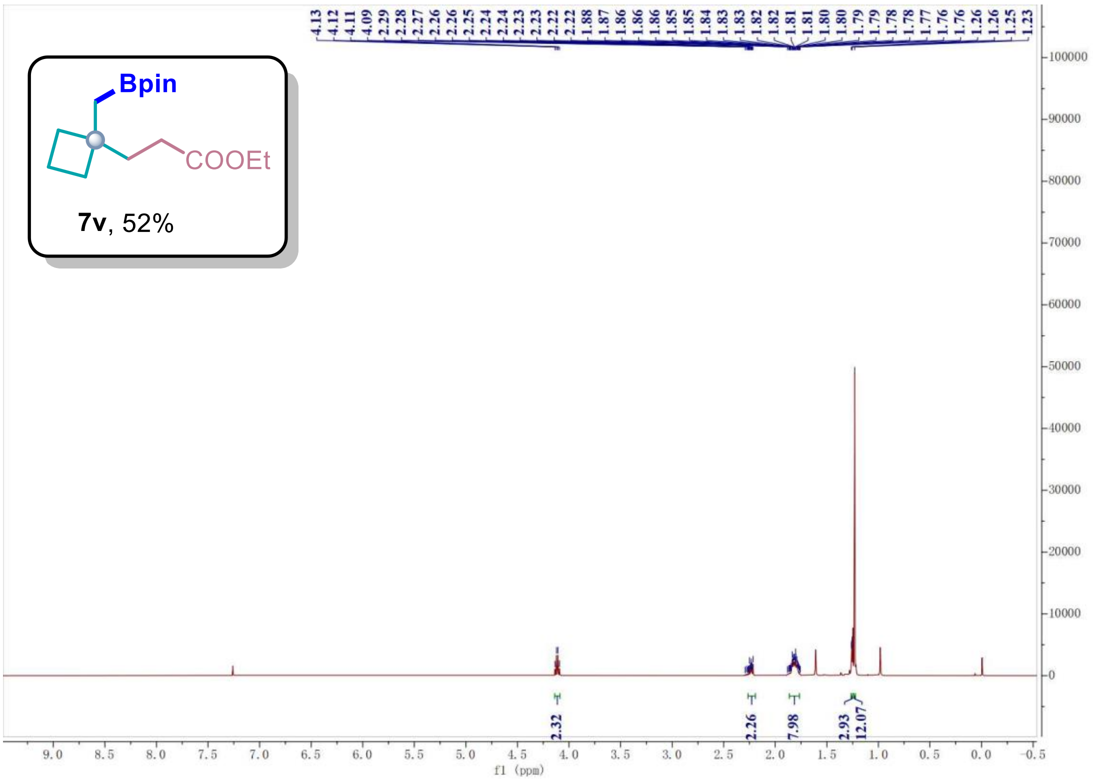

^{13}C NMR (126 MHz, Chloroform-*d*)

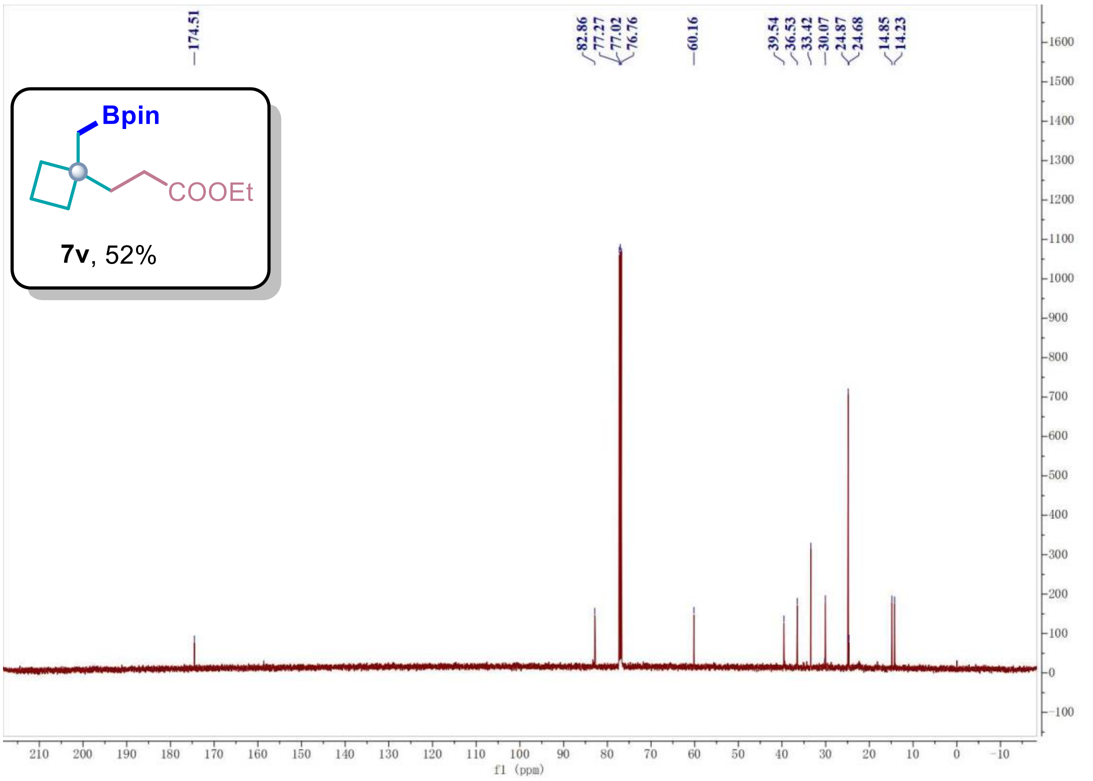

^{11}B NMR (160 MHz, Chloroform-*d*)

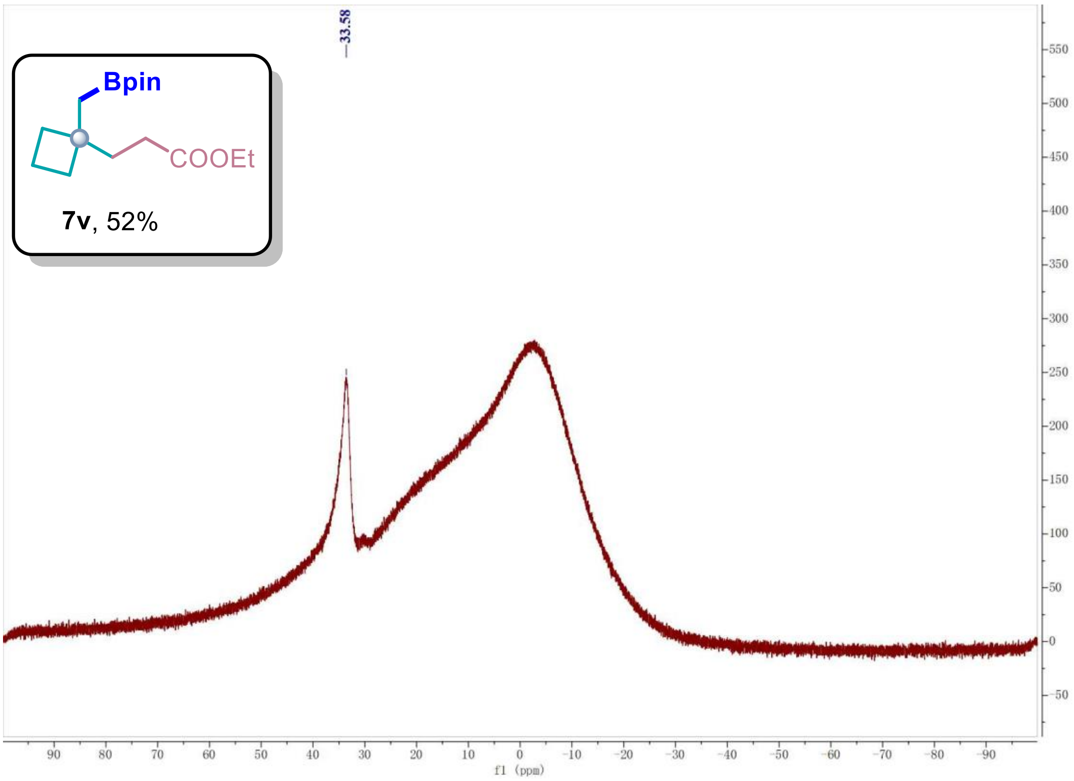

ethyl 6-(4-(tert-butyl)phenyl)-5-methyl-4-((4,4,5,5-tetramethyl-1,3,2-dioxaborolan-2-yl)methyl)hexanoate (7w)

^{1}H NMR (500 MHz, Chloroform-*d*)

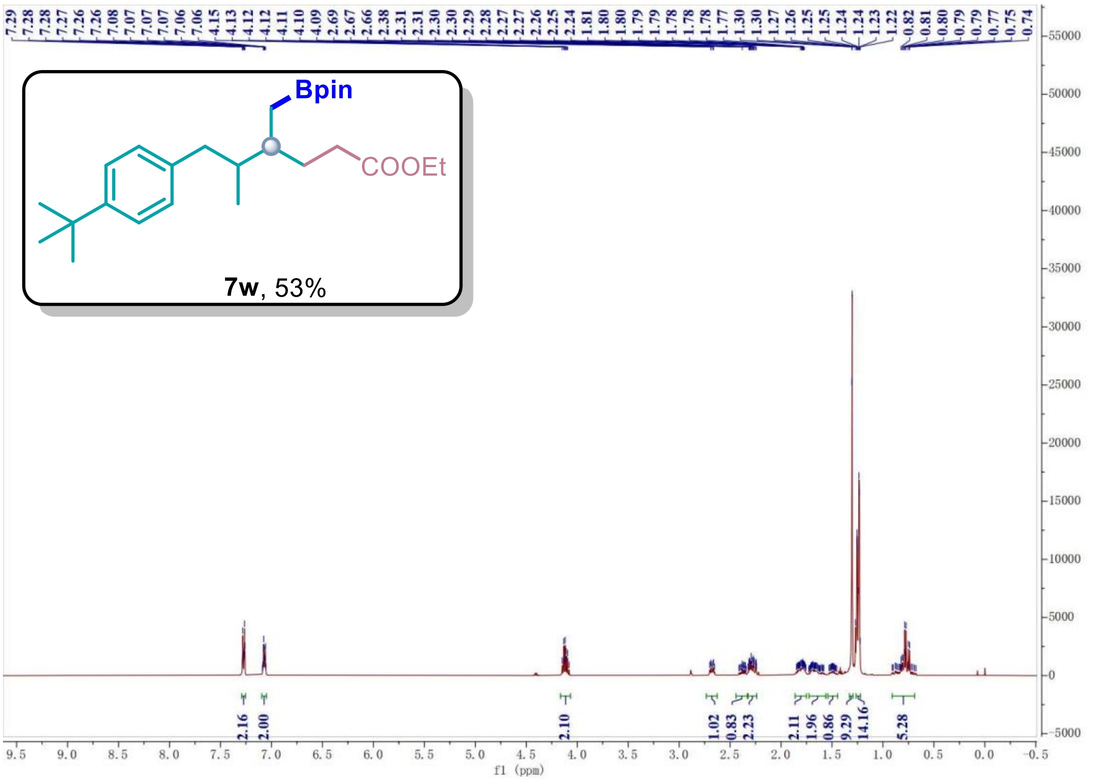

^{13}C NMR (126 MHz, Chloroform-*d*)

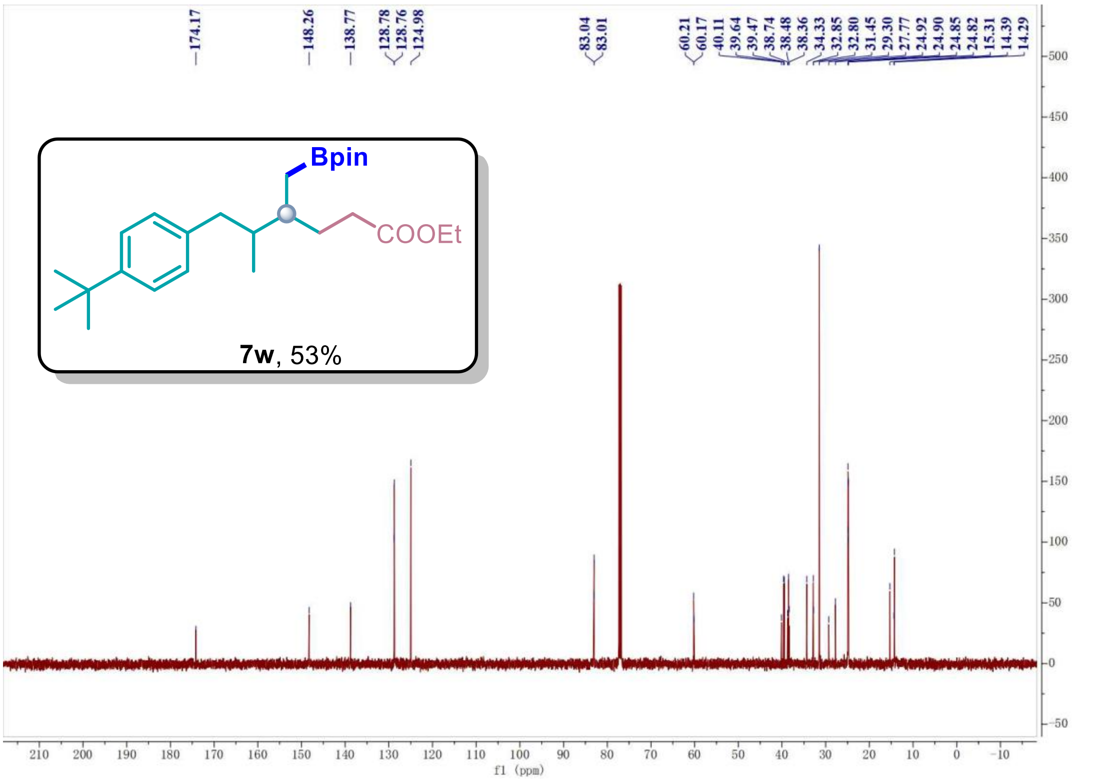

^{11}B NMR (160 MHz, Chloroform-*d*)

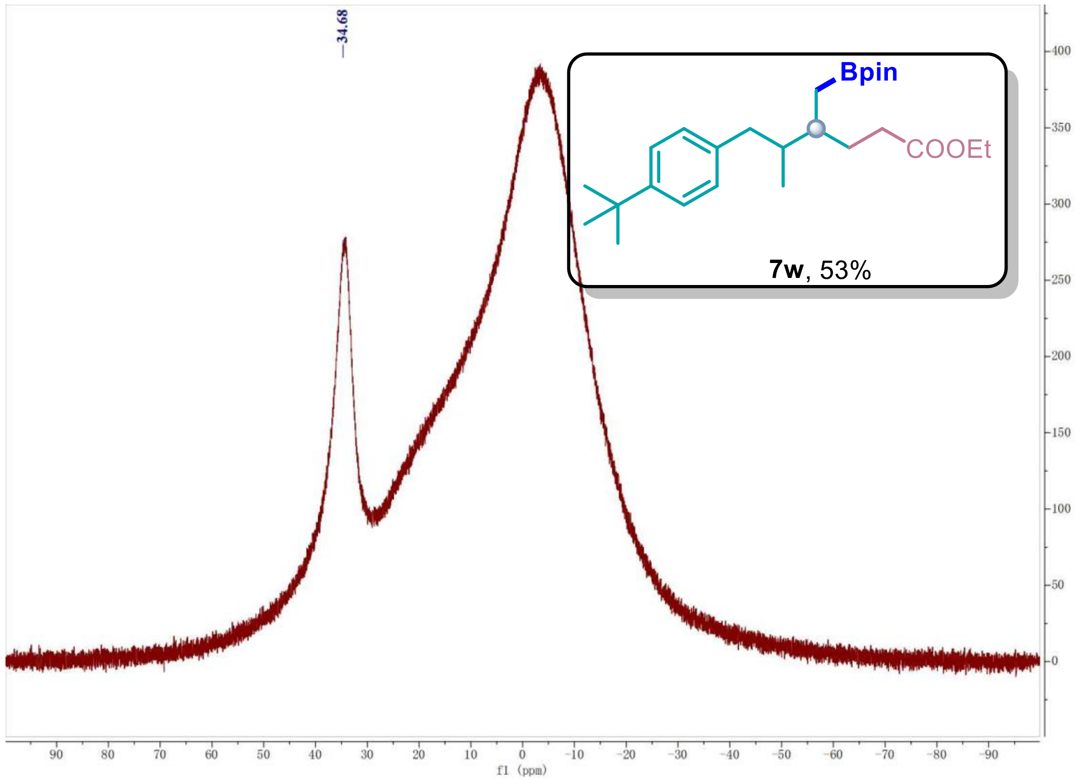

ethyl 4-((4,4,5,5-tetramethyl-1,3,2-dioxaborolan-2-yl)methyl)pentadecanoate (7x)

^{1}H NMR (500 MHz, Chloroform-*d*)

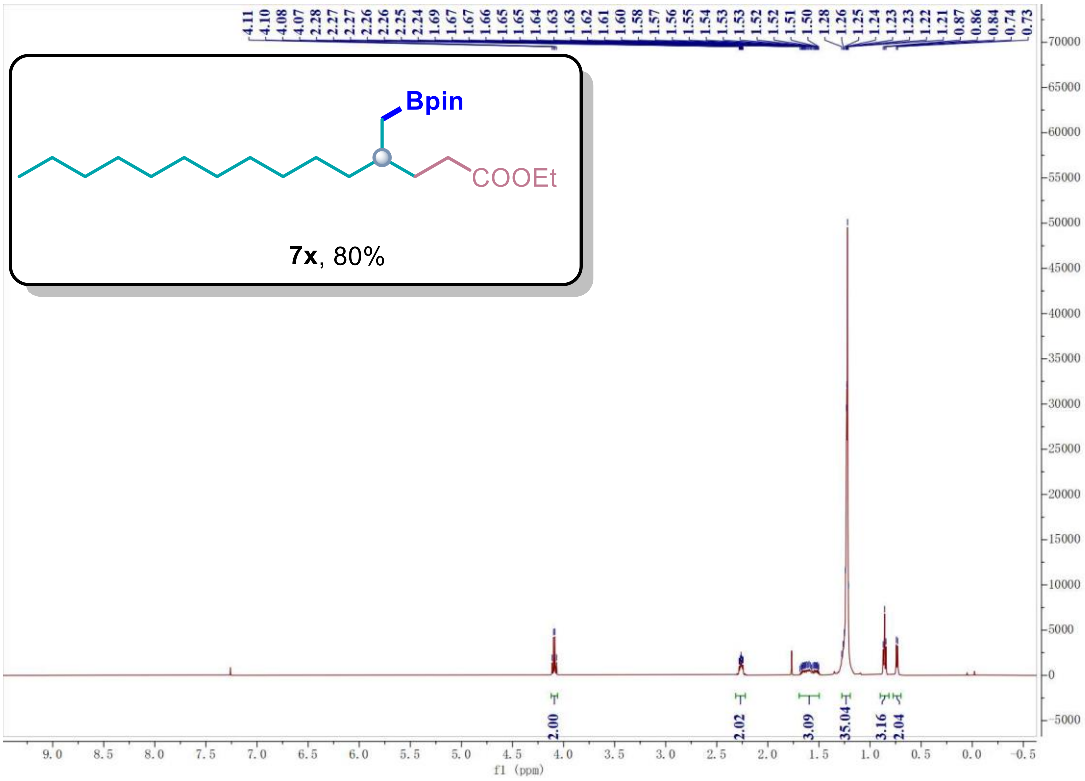

^{13}C NMR (126 MHz, Chloroform-*d*)

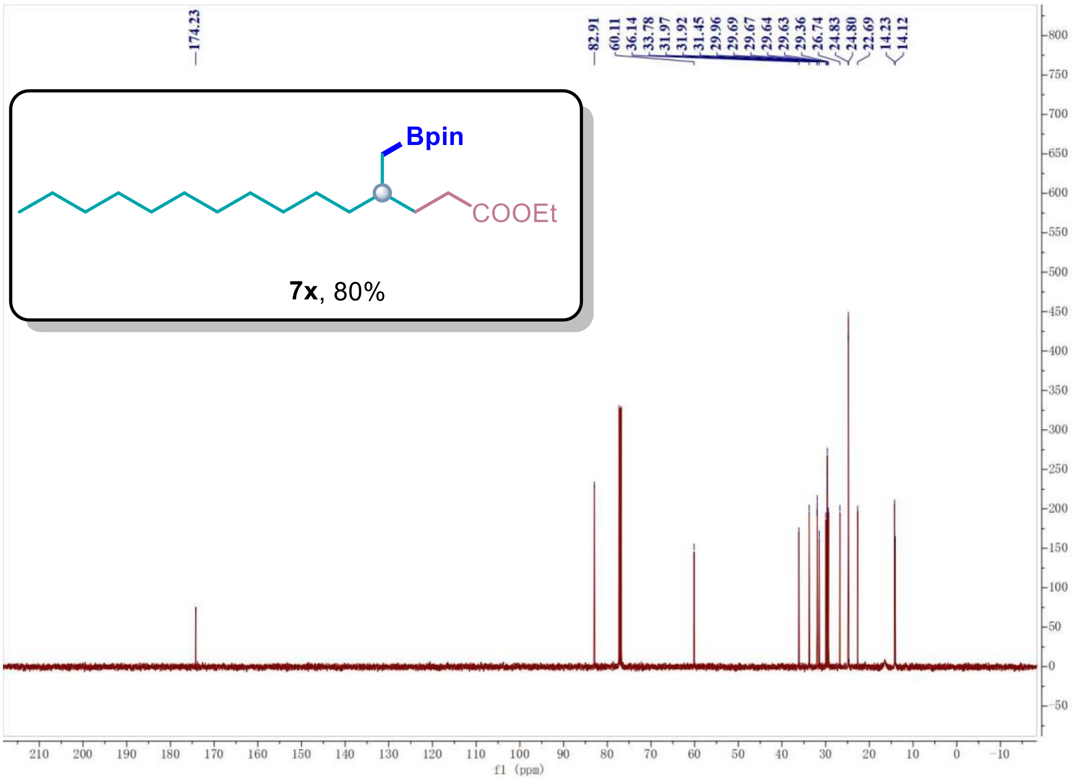

^{11}B NMR (160 MHz, Chloroform-*d*)

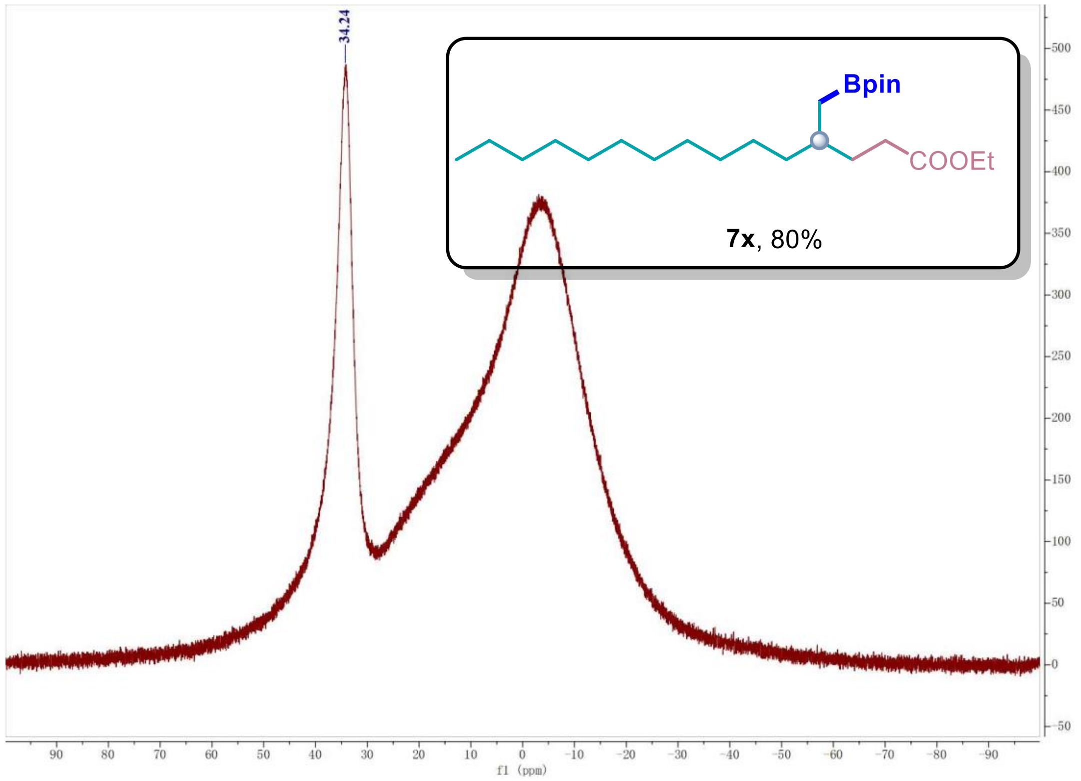

2,2'-(2-methyl-4-phenylpent-4-ene-1,2-diyl)bis(4,4,5,5-tetramethyl-1,3,2-dioxaborolane) (8a)

^{1}H NMR (500 MHz, Chloroform-*d*)

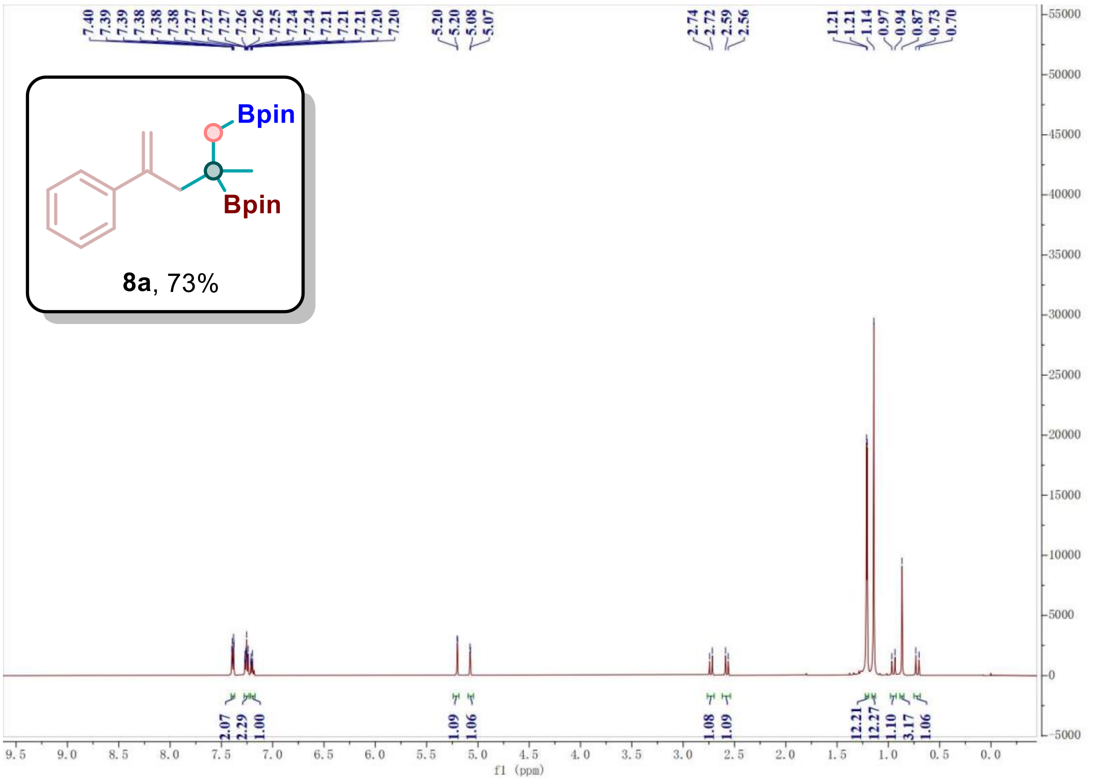

^{13}C NMR (126 MHz, Chloroform-*d*)

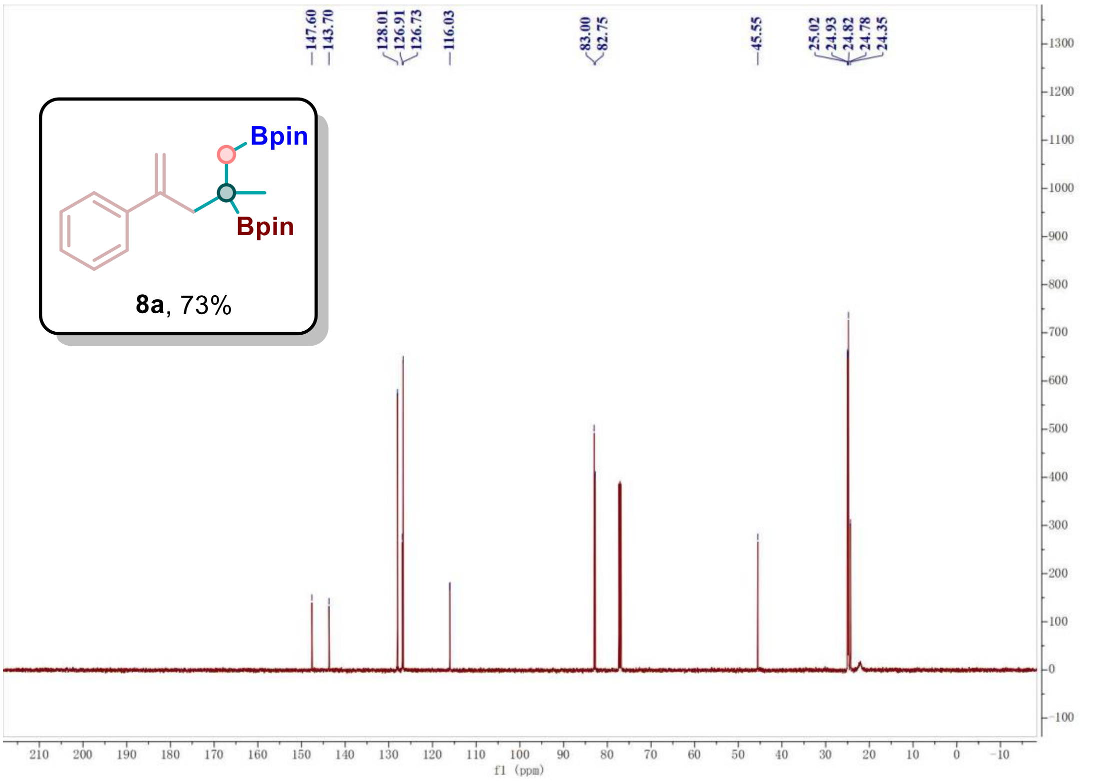

^{11}B NMR (160 MHz, Chloroform-*d*)

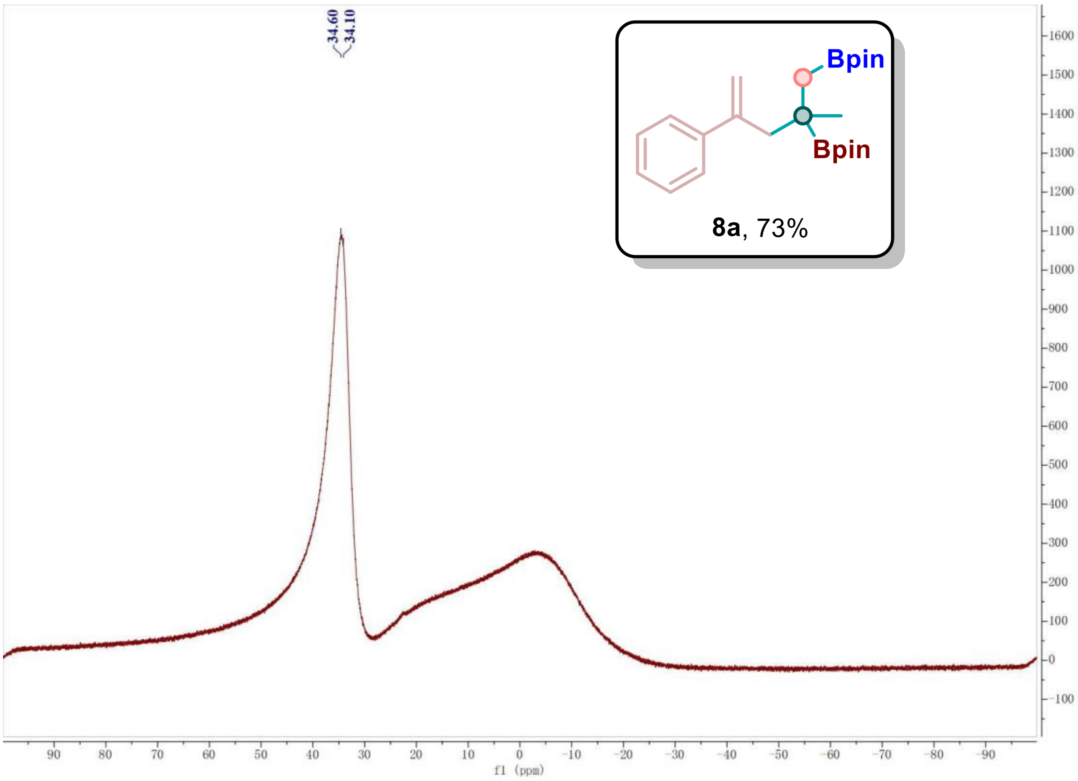

2,2'-(2-methyl-4-(p-tolyl)pent-4-ene-1,2-diyl)bis(4,4,5,5-tetramethyl-1,3,2-dioxaborolane) (8b)

^{1}H NMR (500 MHz, Chloroform-*d*)

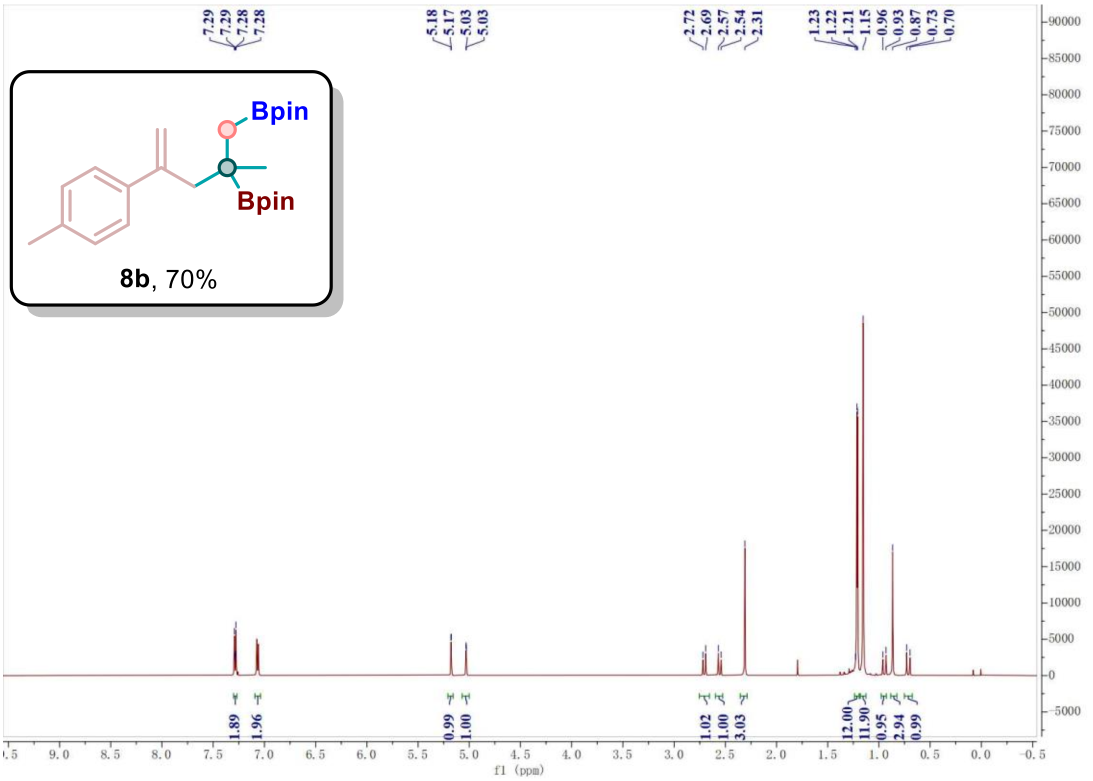

^{13}C NMR (126 MHz, Chloroform-*d*)

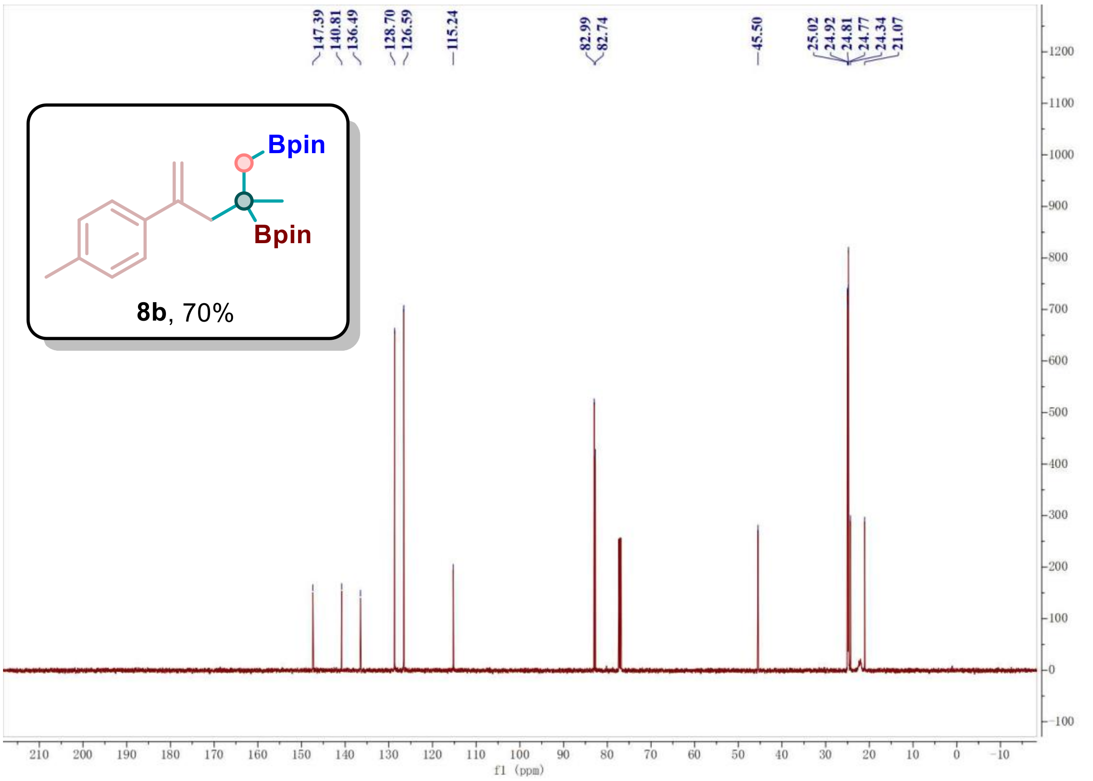

^{11}B NMR (160 MHz, Chloroform-*d*)

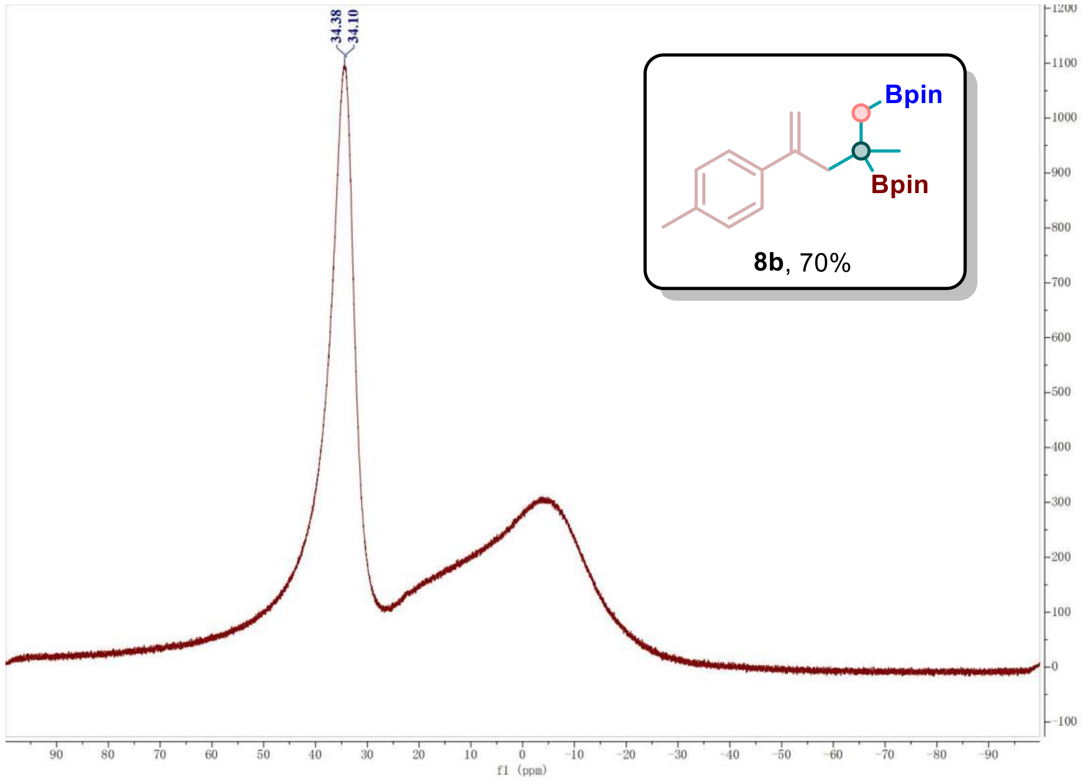

2,2'-(4-(4-bromophenyl)-2-methylpent-4-ene-1,2-diyl)bis(4,4,5,5-tetramethyl-1,3,2-dioxaborolane) (8c)

^{1}H NMR (500 MHz, Chloroform-*d*)

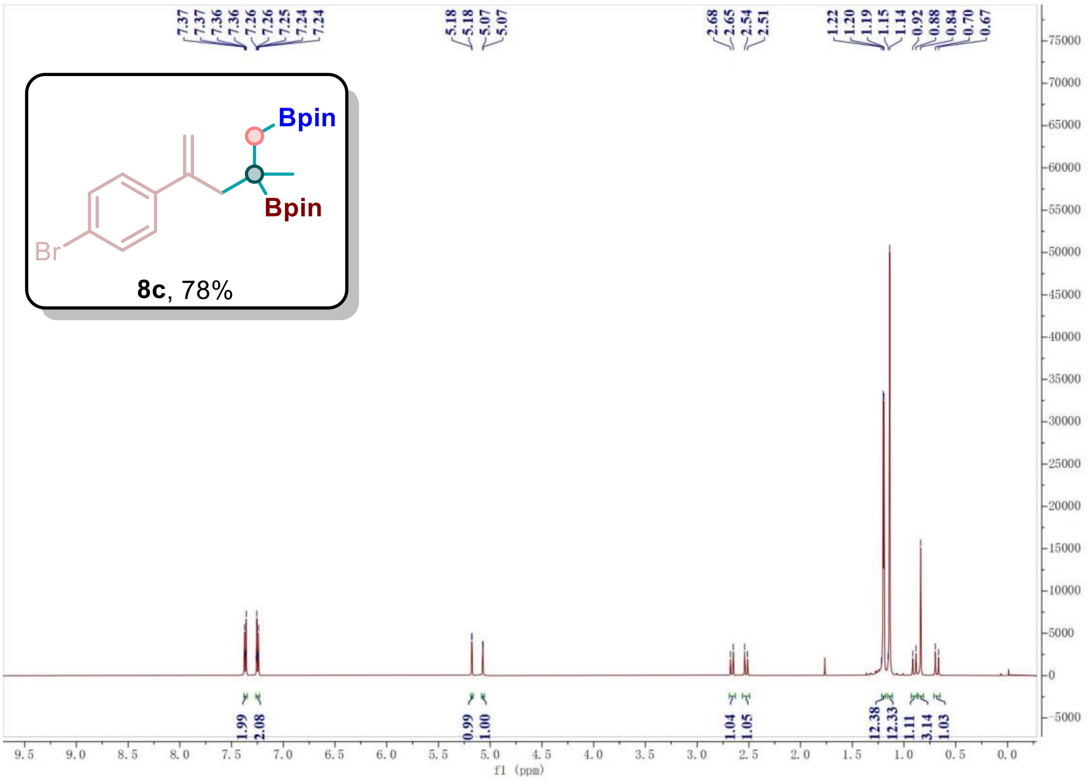

^{13}C NMR (126 MHz, Chloroform-*d*)

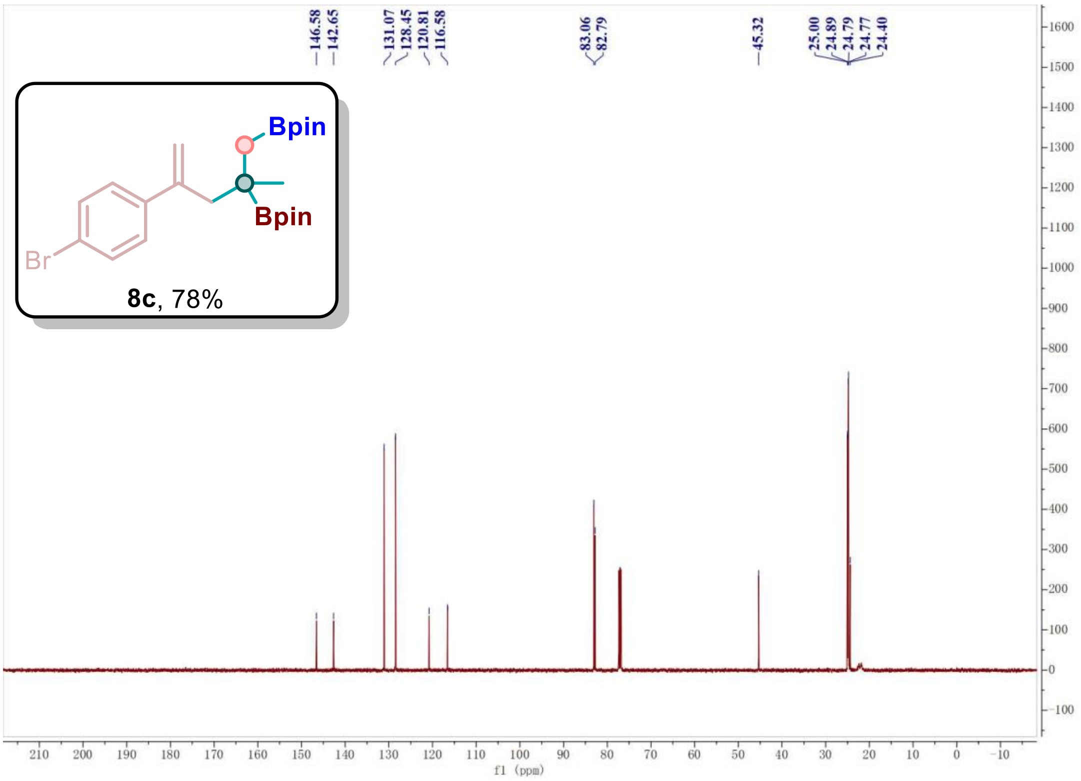

^{11}B NMR (160 MHz, Chloroform-*d*)

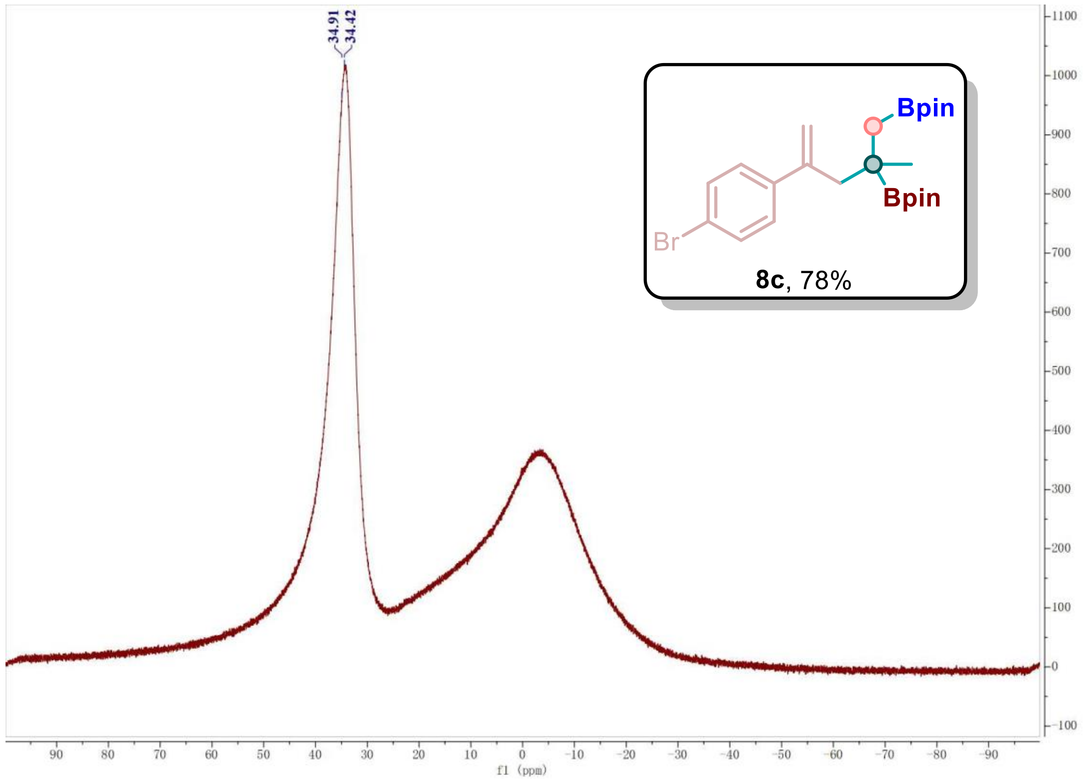

2,2'-(4-chloro-2-methylpent-4-ene-1,2-diyl)bis(4,4,5,5-tetramethyl-1,3,2-dioxaborolane) (8d)

^{1}H NMR (500 MHz, Chloroform-*d*)

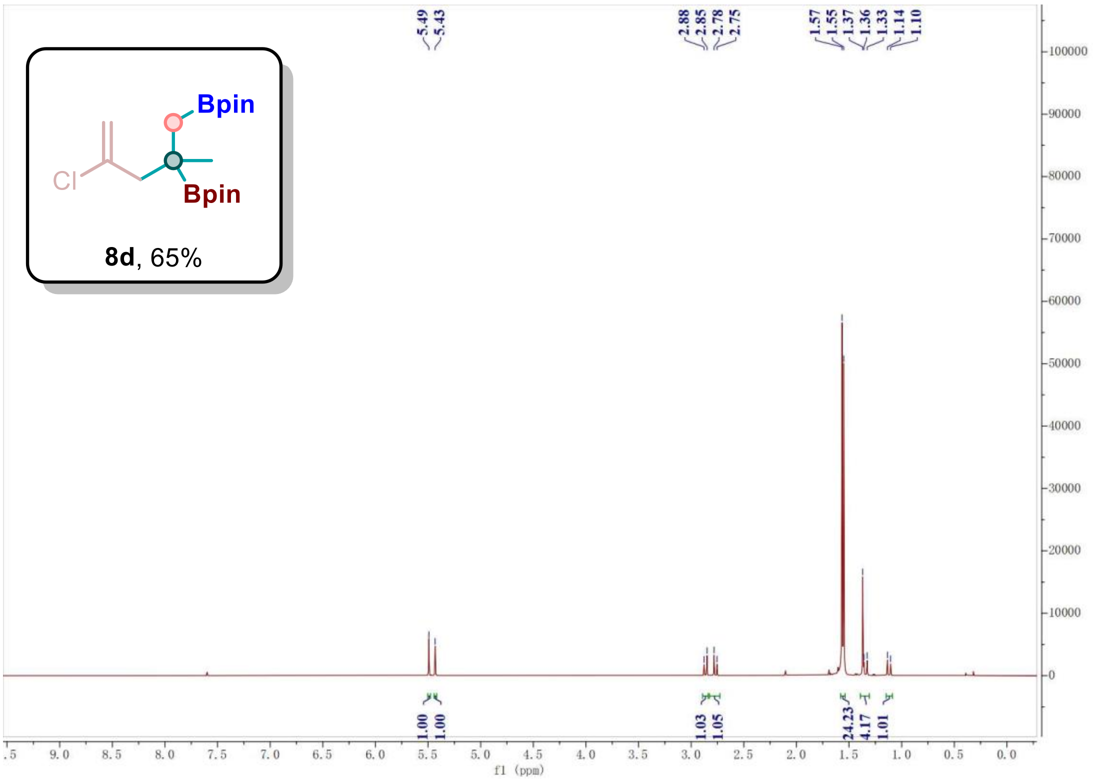

^{13}C NMR (126 MHz, Chloroform-*d*)

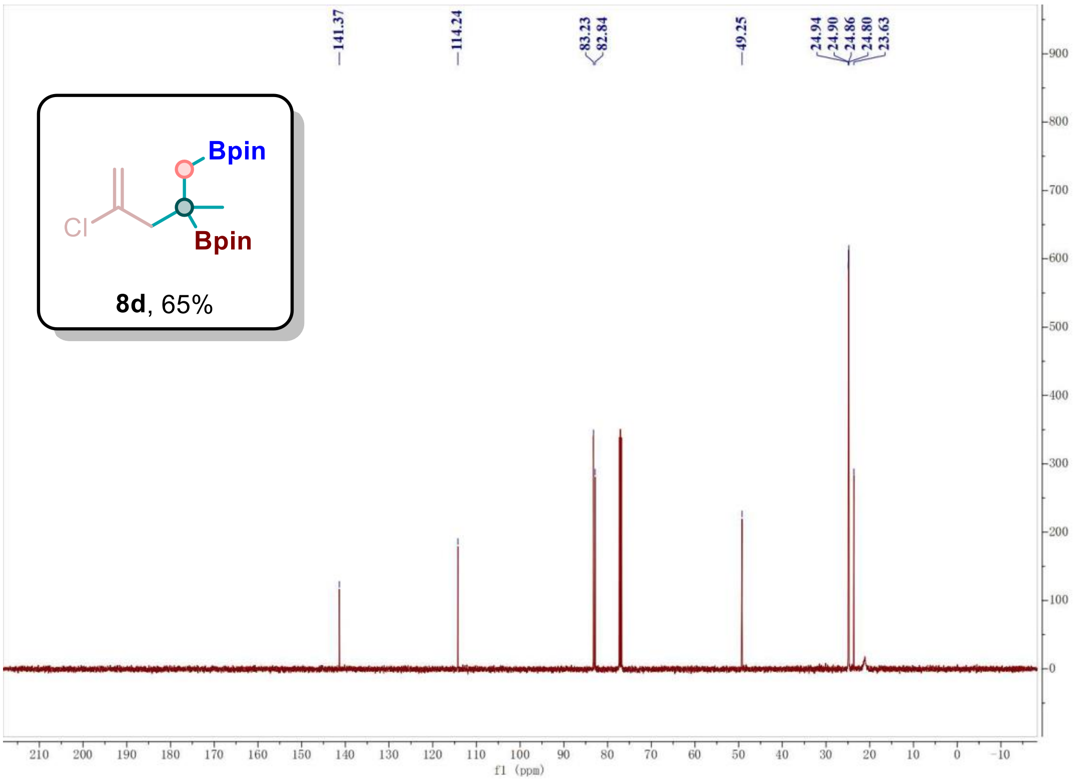

^{11}B NMR (160 MHz, Chloroform-*d*)

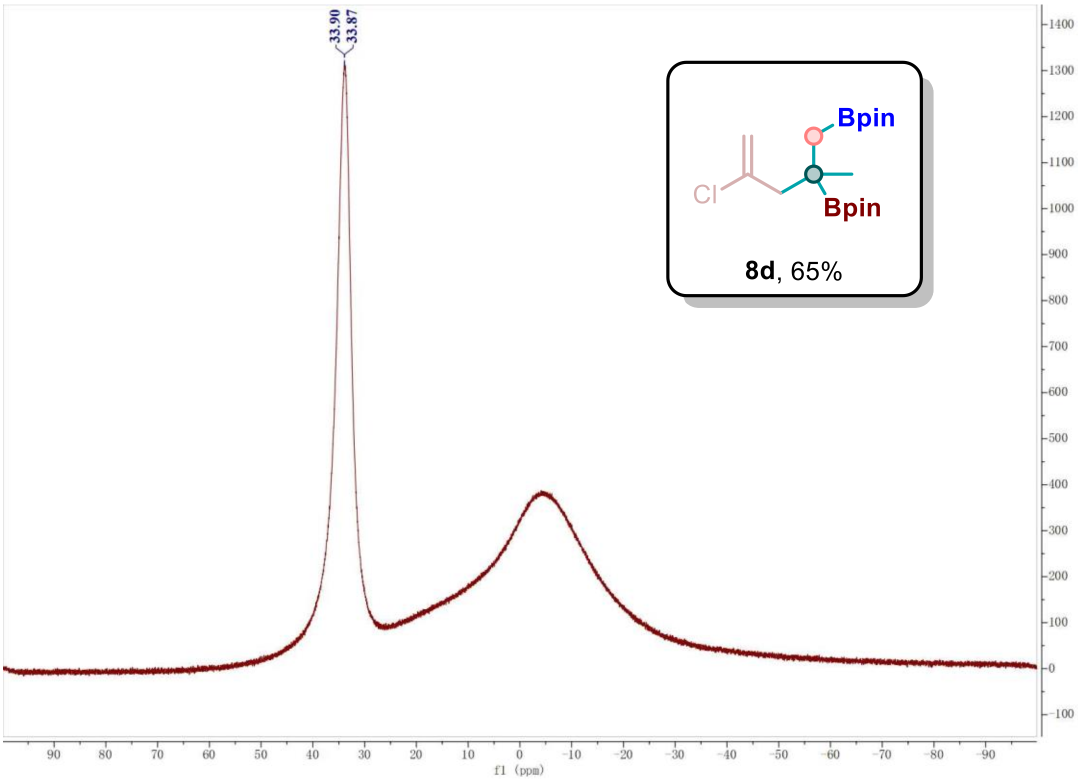

2,2'-(2-ethyl-4-phenylpent-4-ene-1,2-diyl)bis(4,4,5,5-tetramethyl-1,3,2-dioxaborolane) (8e)

^{1}H NMR (500 MHz, Chloroform-*d*)

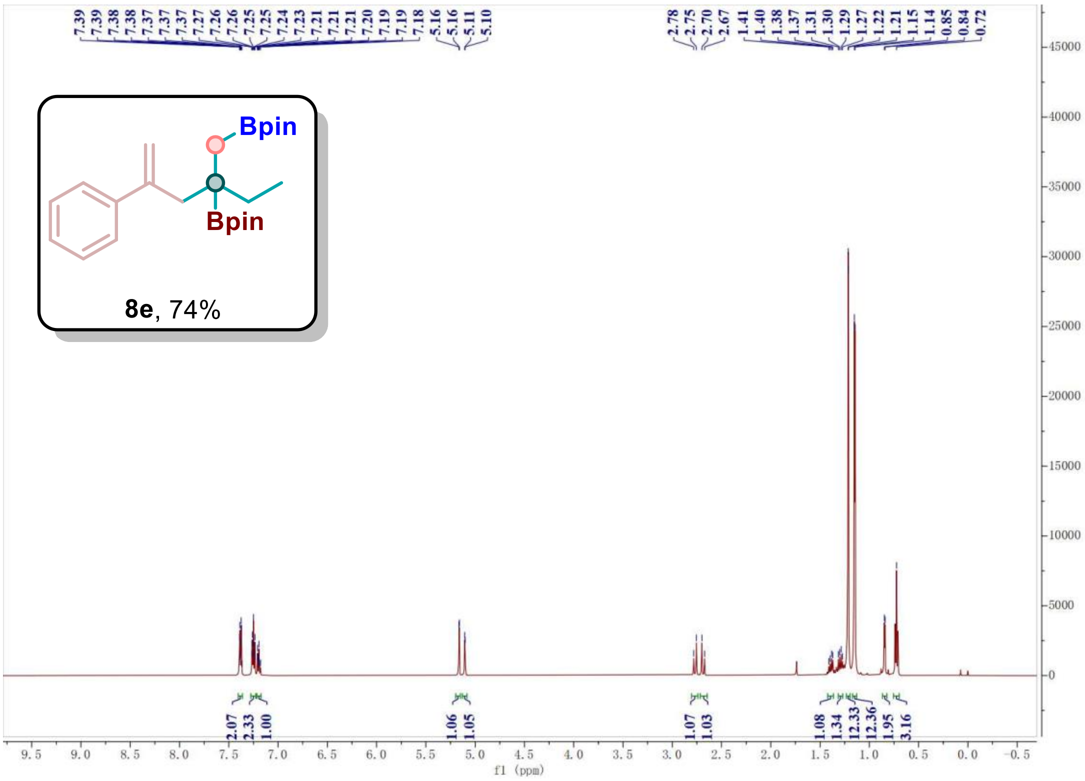

^{13}C NMR (126 MHz, Chloroform-*d*)

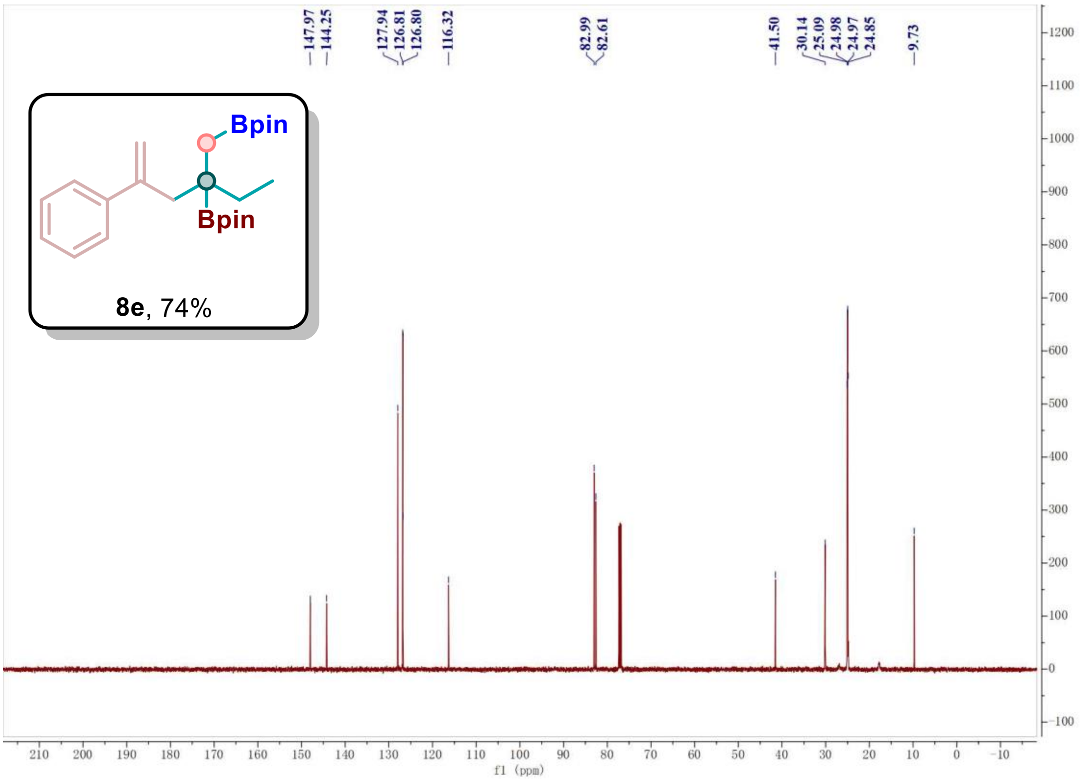

^{11}B NMR (160 MHz, Chloroform-*d*)

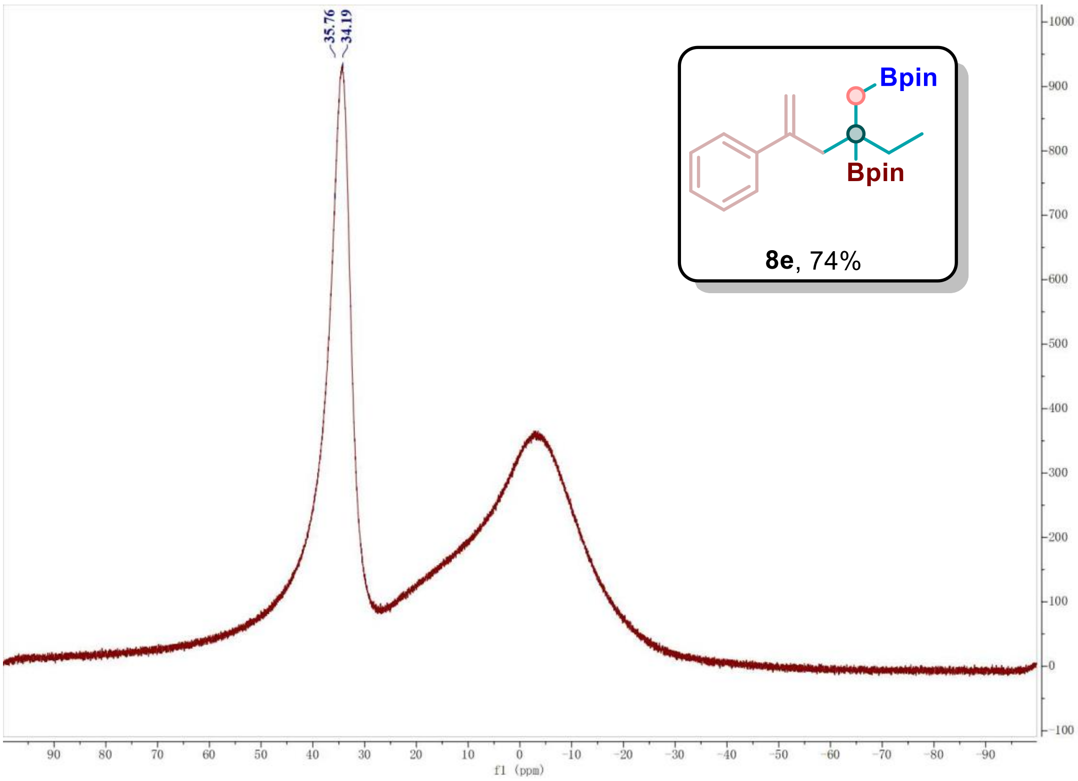

2,2'-(2-ethyl-4-(4-fluorophenyl)pent-4-ene-1,2-diyl)bis(4,4,5,5-tetramethyl-1,3,2-dioxaborolane) (8f)

^{1}H NMR (500 MHz, Chloroform-*d*)

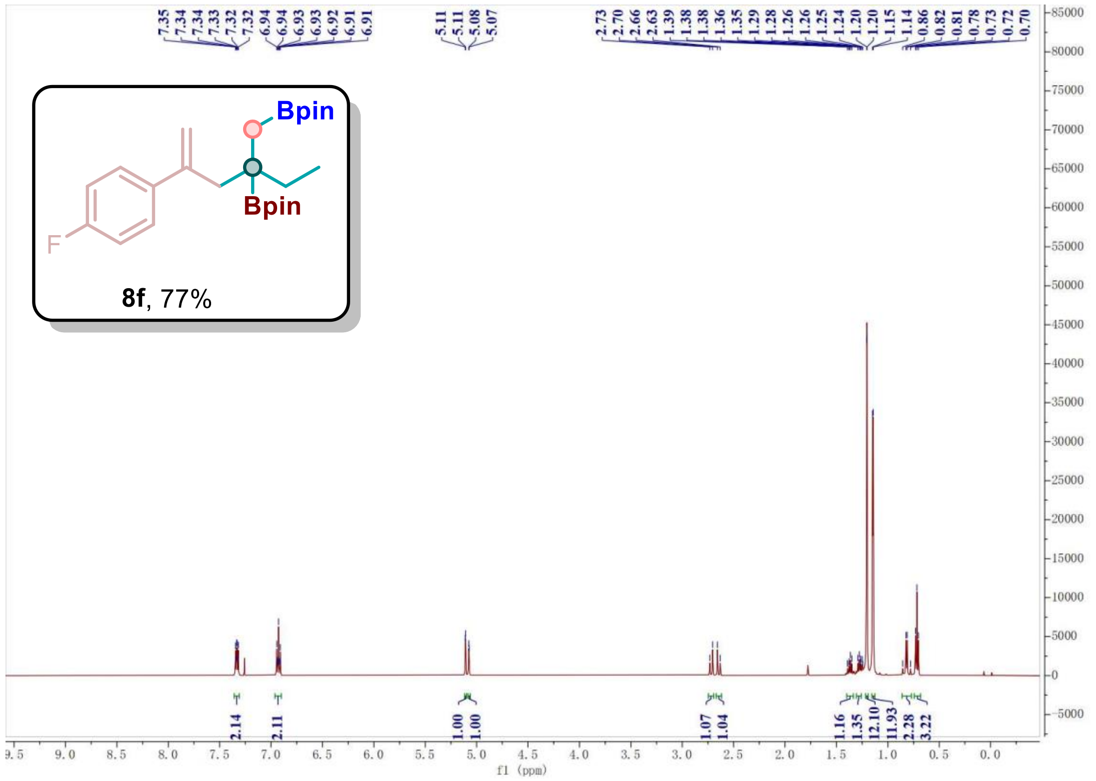

^{13}C NMR (126 MHz, Chloroform-*d*)

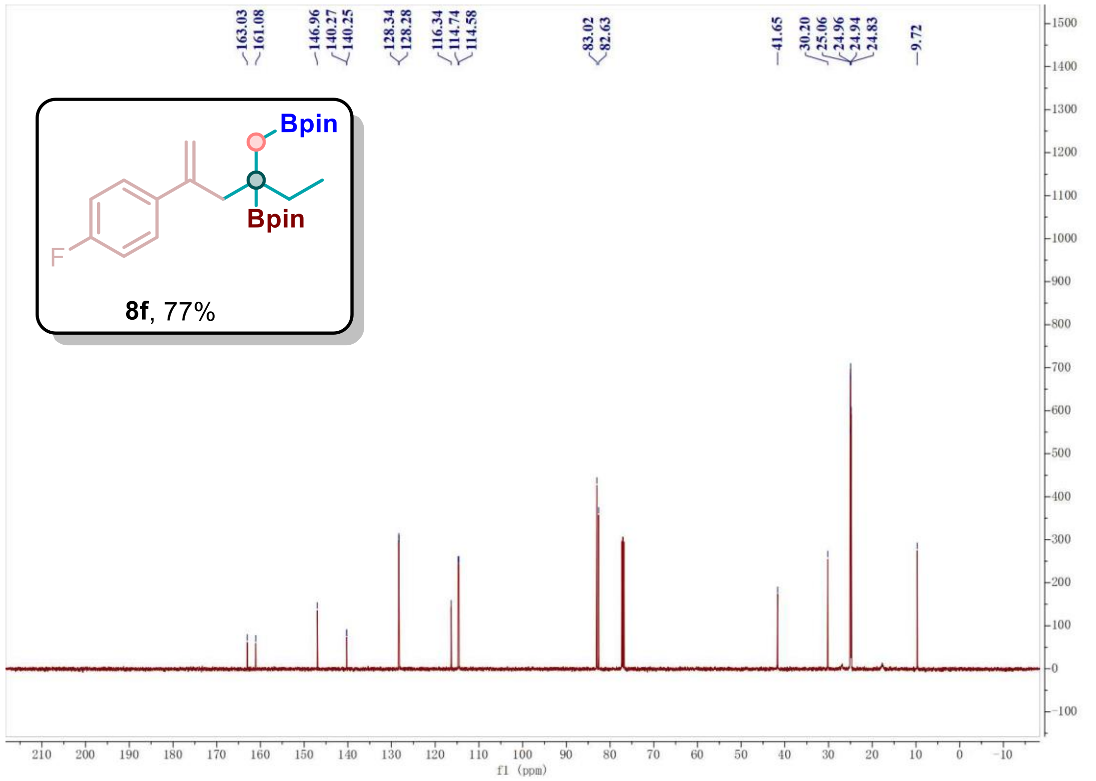

^{11}B NMR (160 MHz, Chloroform-*d*)

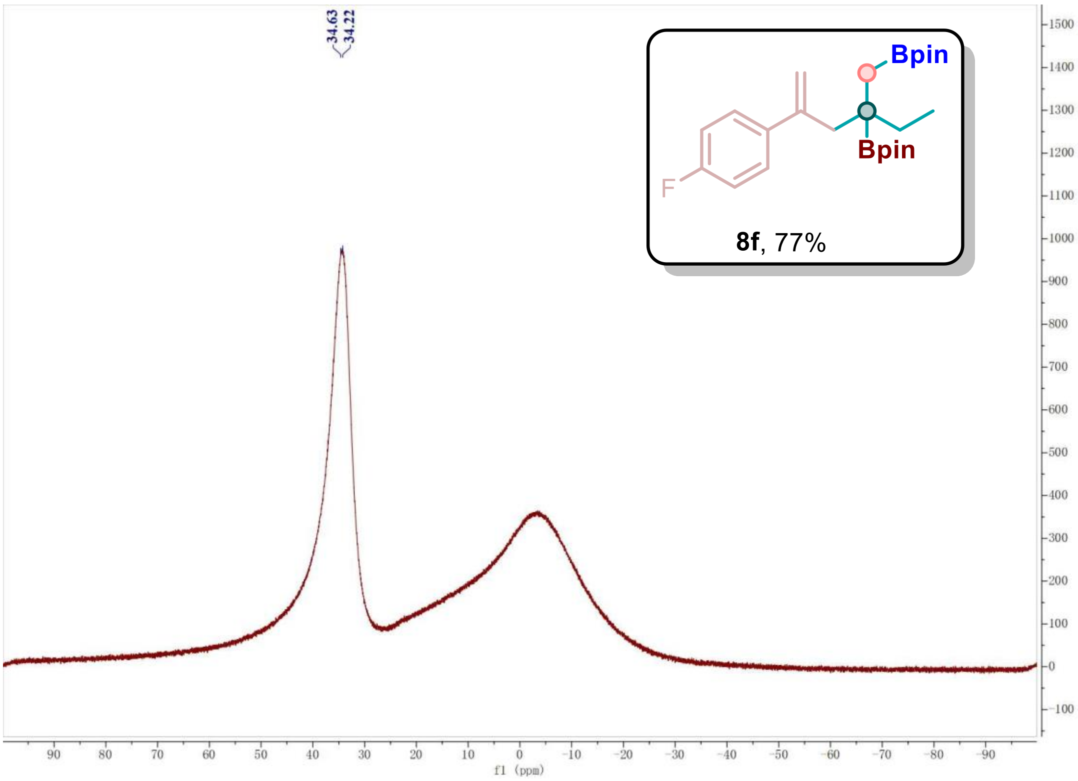

^{19}F NMR (471 MHz, Chloroform-*d*)

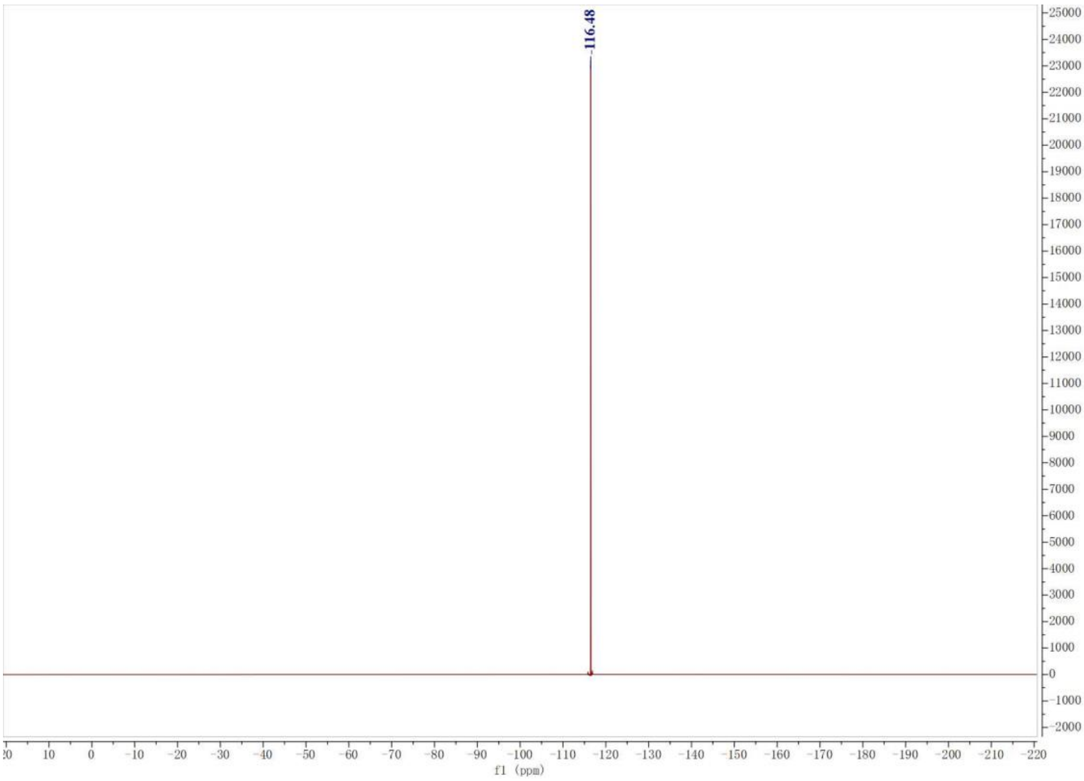

2,2'-(4-(4-chlorophenyl)-2-ethylpent-4-ene-1,2-diyl)bis(4,4,5,5-tetramethyl-1,3,2-dioxaborolane) (8g)

^{1}H NMR (500 MHz, Chloroform-*d*)

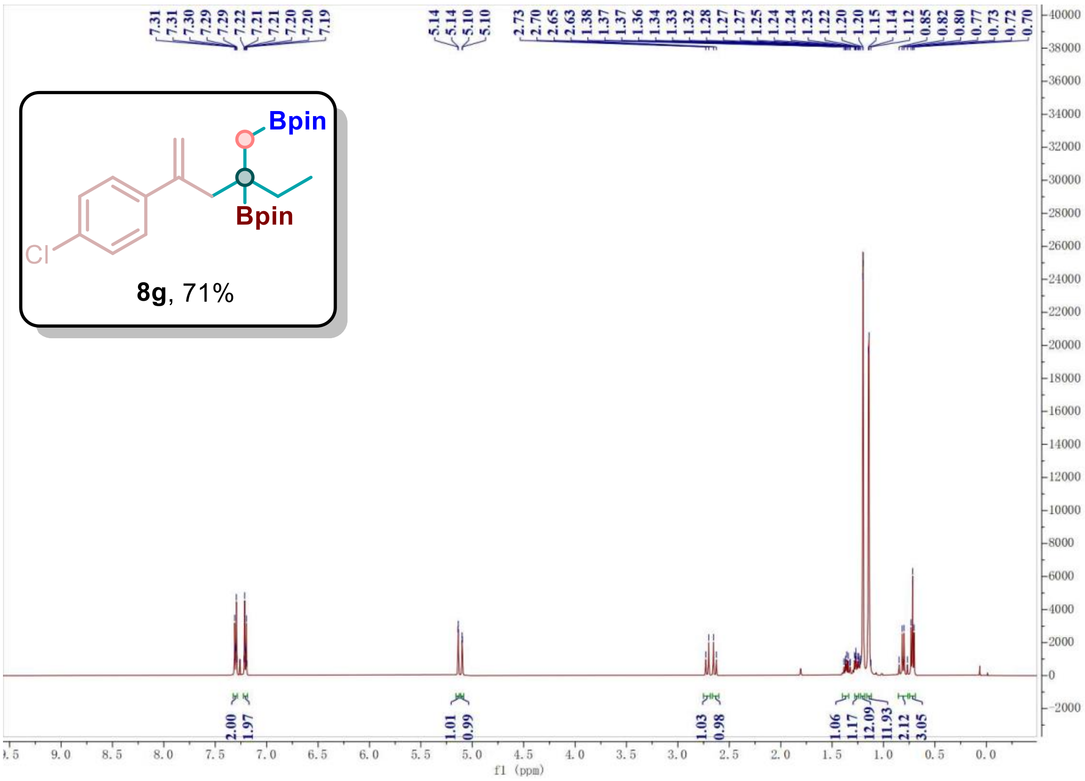

^{13}C NMR (126 MHz, Chloroform-*d*)

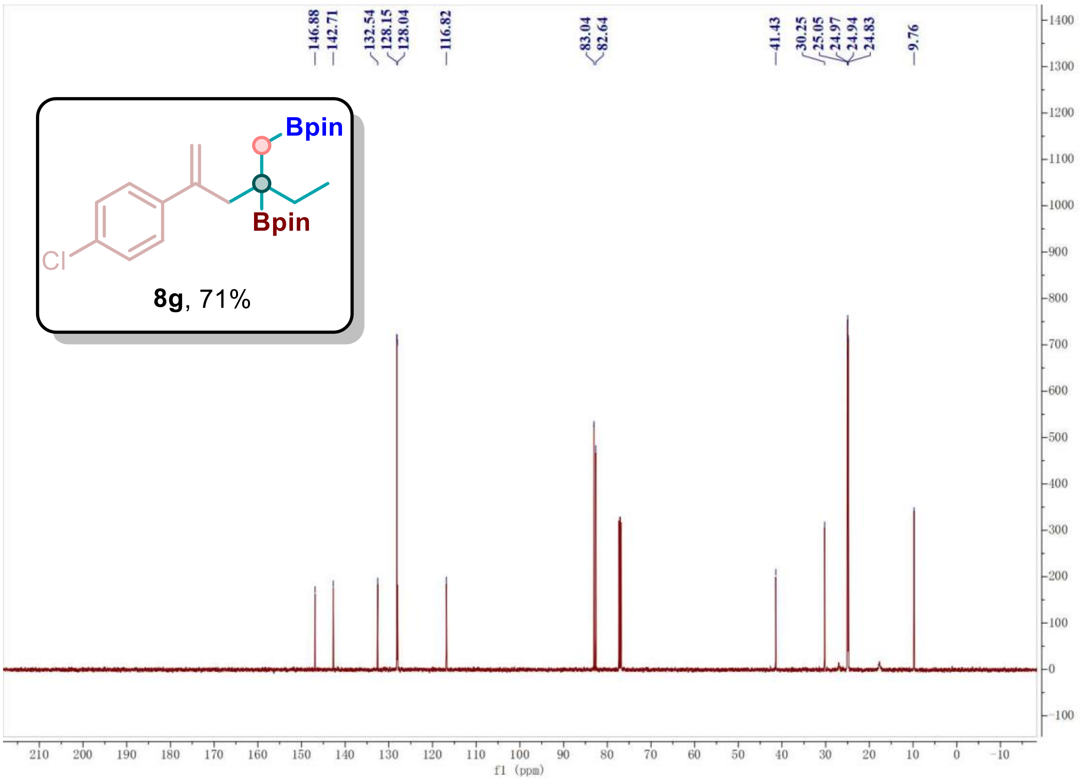

^{11}B NMR (160 MHz, Chloroform-*d*)

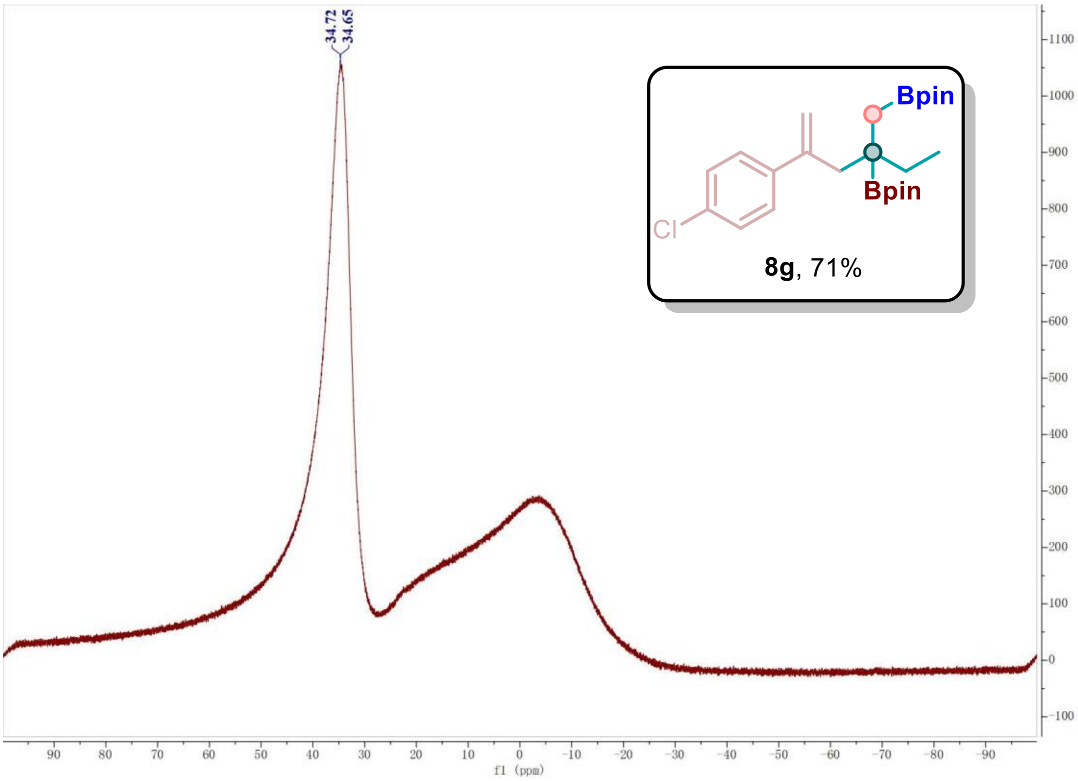

2,2'-(2-ethyl-4-(4-methoxyphenyl)pent-4-ene-1,2-diyl)bis(4,4,5,5-tetramethyl-1,3,2-dioxaborolane) (8h)

^{1}H NMR (500 MHz, Chloroform-*d*)

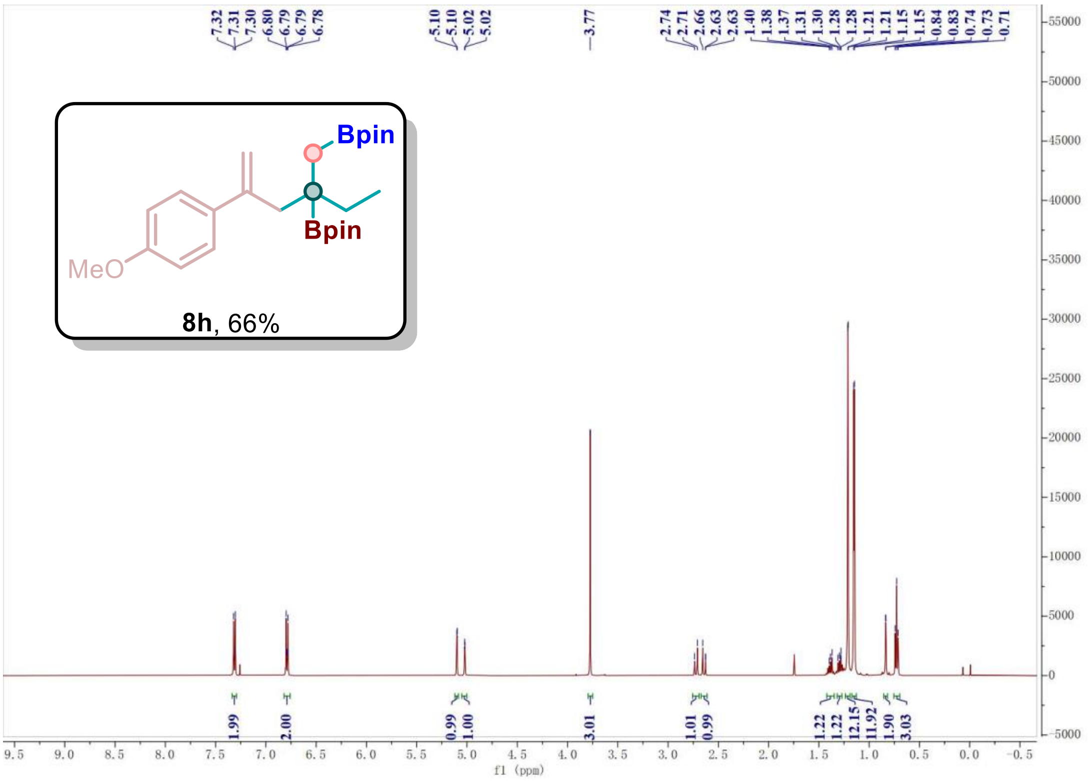

^{13}C NMR (126 MHz, Chloroform-*d*)

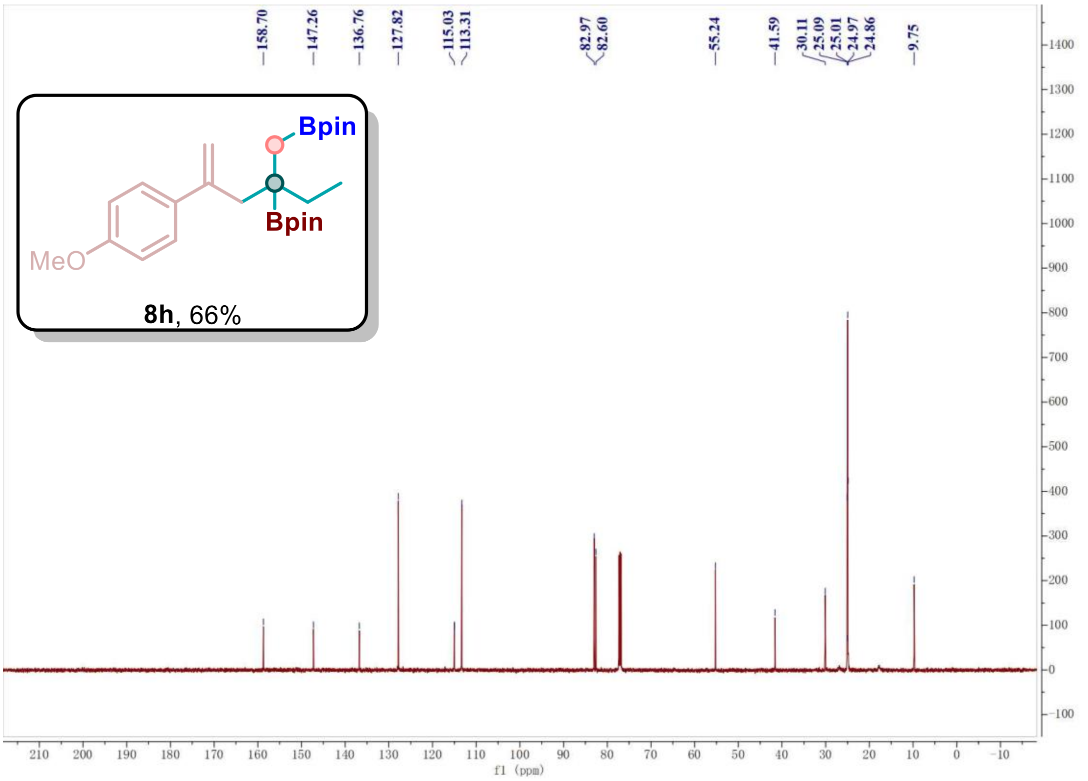

^{11}B NMR (160 MHz, Chloroform-*d*)

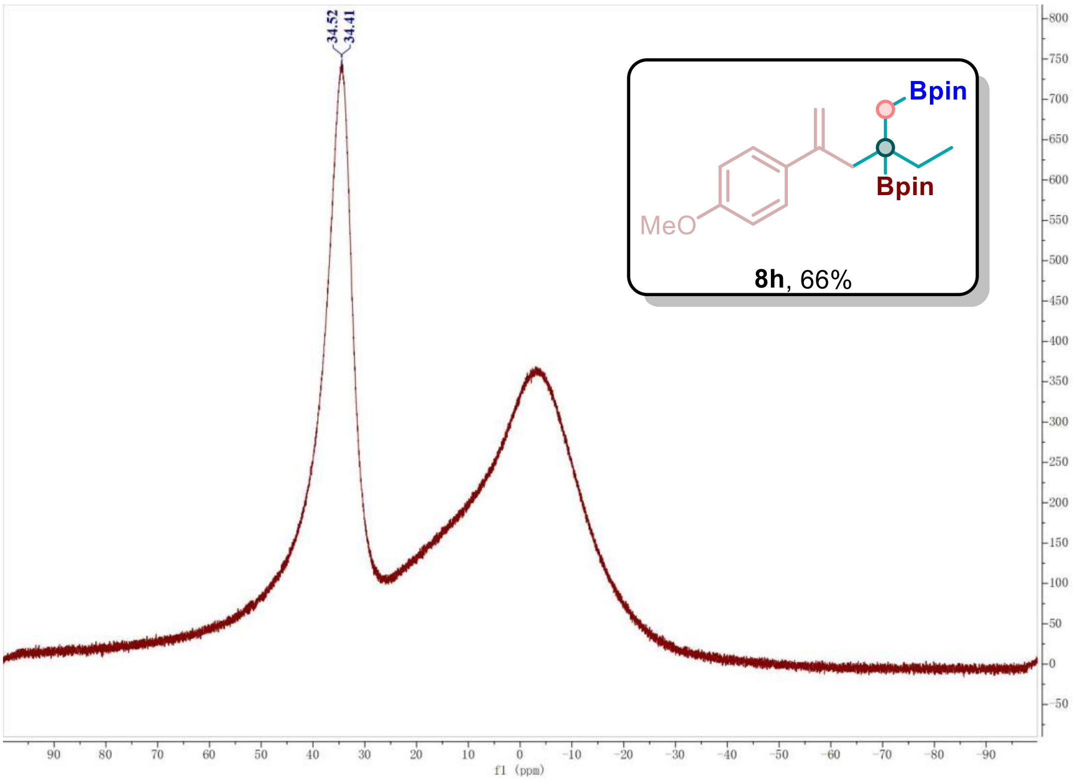

2,2'-(2-ethyl-4-(naphthalen-2-yl)pent-4-ene-1,2-diyl)bis(4,4,5,5-tetramethyl-1,3,2-dioxaborolane) (8i)

^{1}H NMR (500 MHz, Chloroform-*d*)

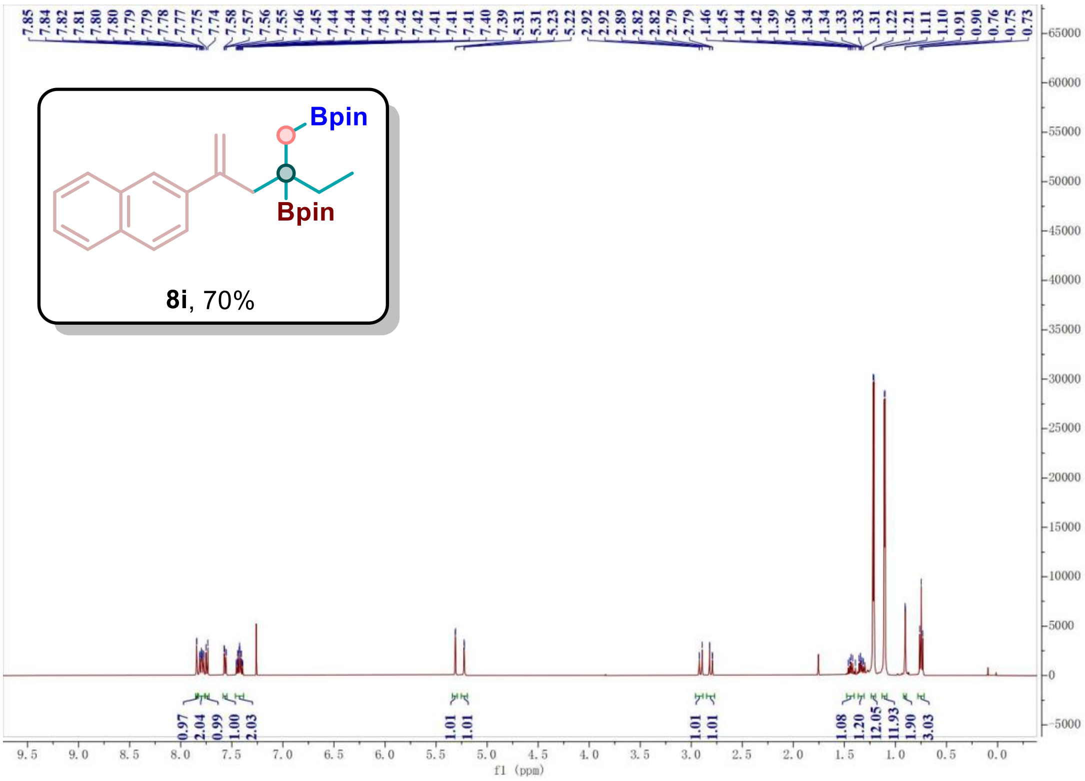

^{13}C NMR (126 MHz, Chloroform-*d*)

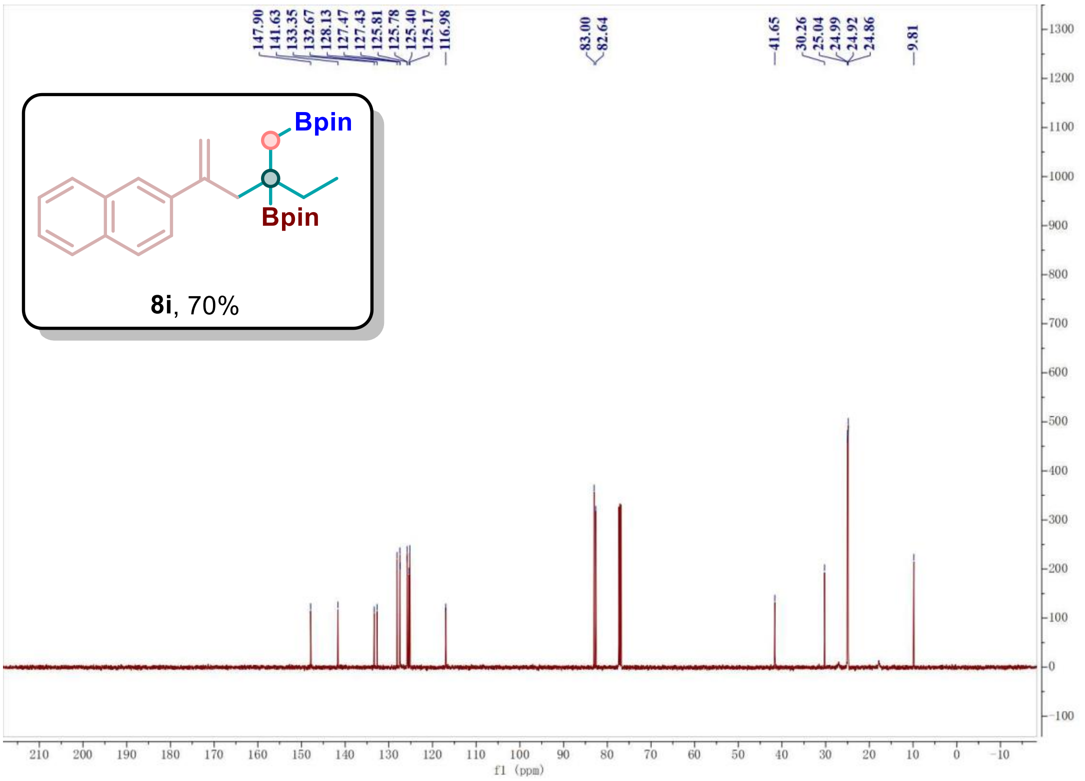

^{11}B NMR (160 MHz, Chloroform-*d*)

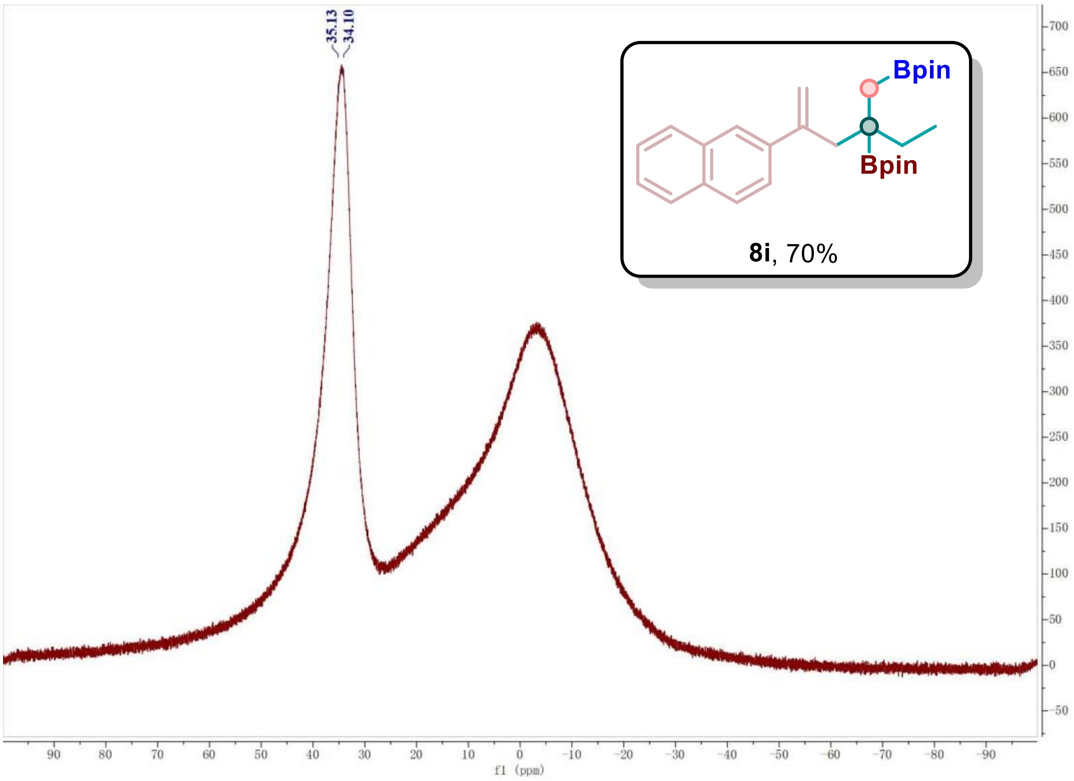

2,2'-(4-chloro-2-ethylpent-4-ene-1,2-diyl)bis(4,4,5,5-tetramethyl-1,3,2-dioxaborolane) (8j)

^{1}H NMR (500 MHz, Chloroform-*d*)

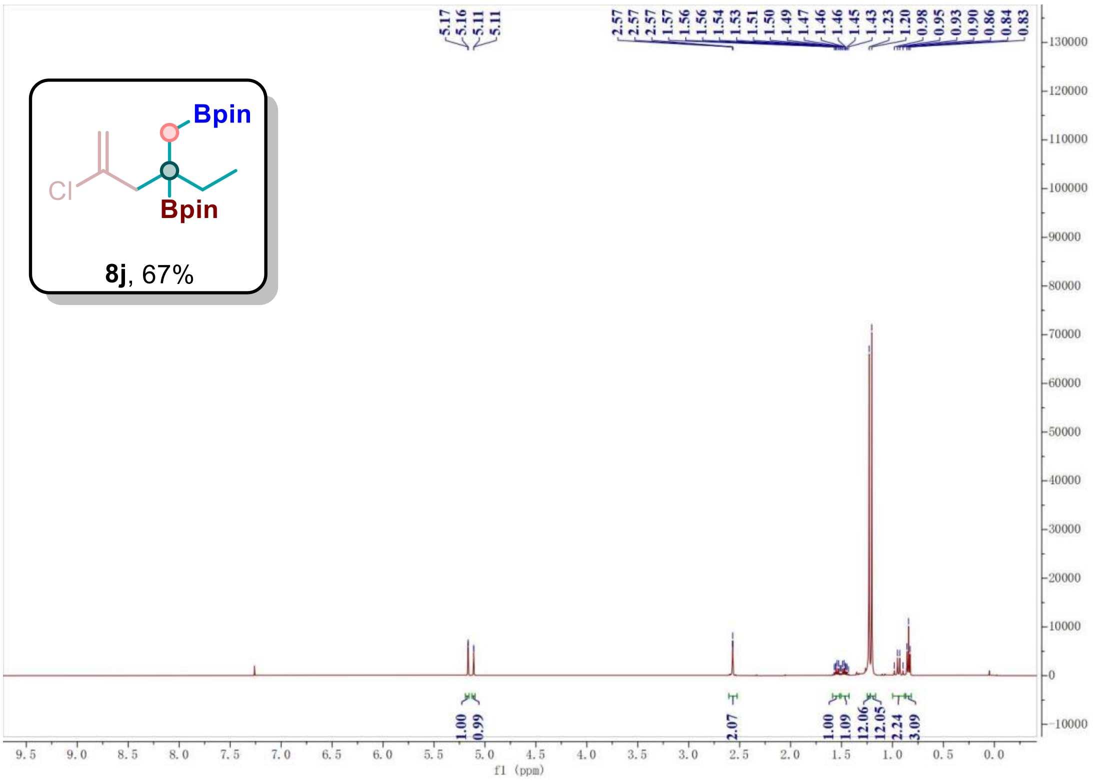

^{13}C NMR (126 MHz, Chloroform-*d*)

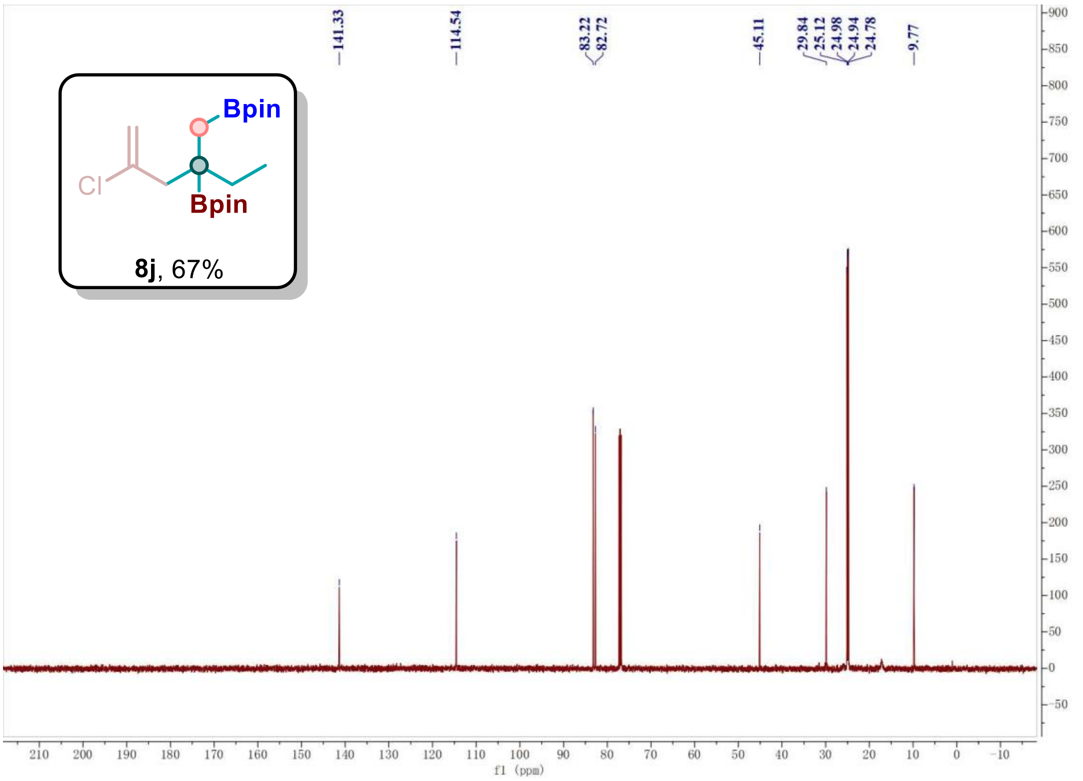

^{11}B NMR (160 MHz, Chloroform-*d*)

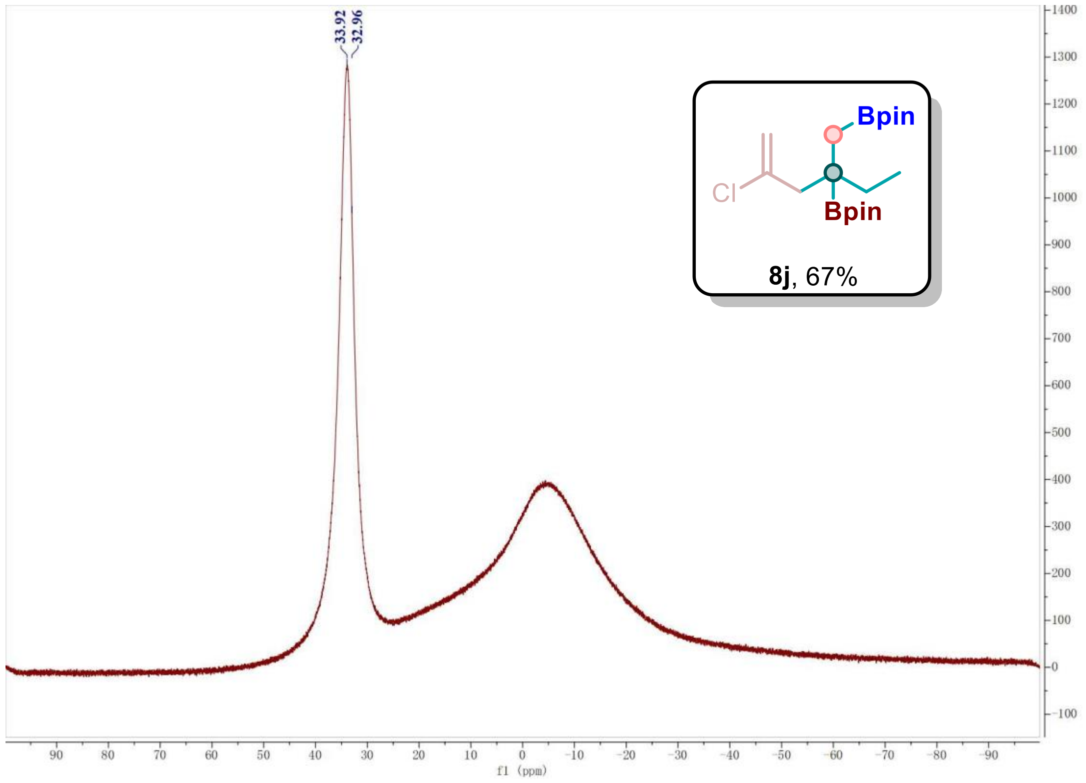
33.92
32.96
Bpin
Cl
Bpin
8j, 67%
f1 (ppm)

2,2'-(2-(2-phenylallyl)hexane-1,2-diyl)bis(4,4,5,5-tetramethyl-1,3,2-dioxaborolane) (8k)

^{1}H NMR (500 MHz, Chloroform-*d*)

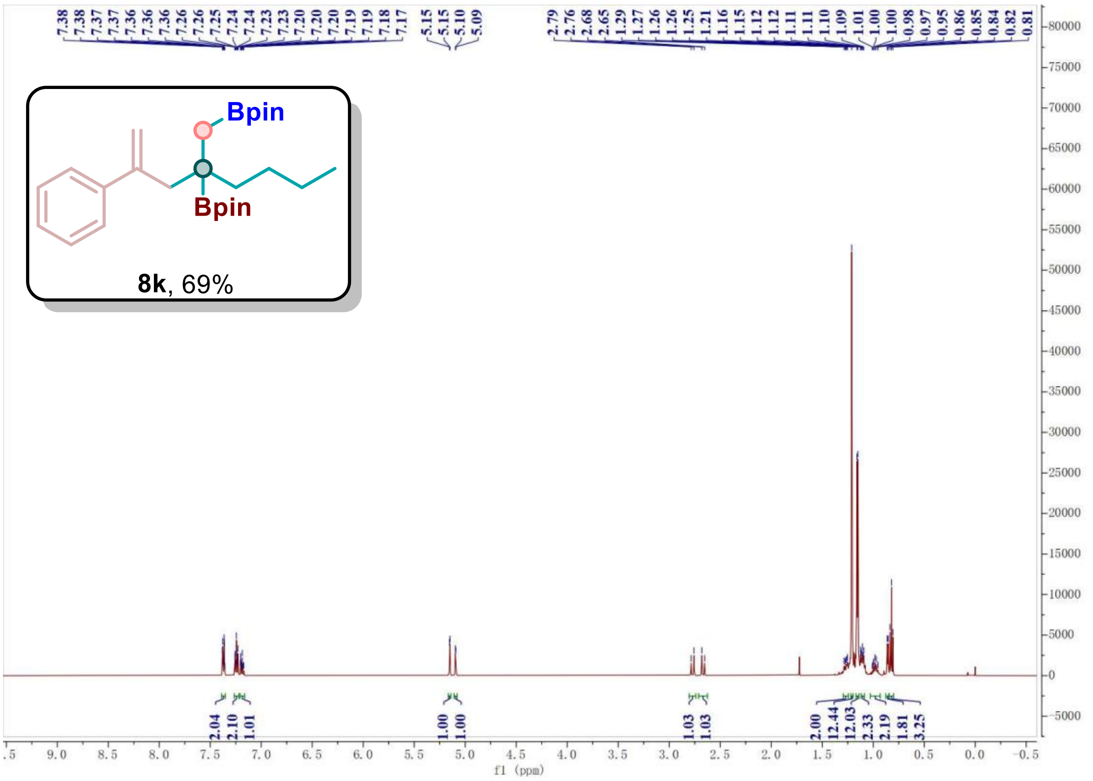

^{13}C NMR (126 MHz, Chloroform-*d*)

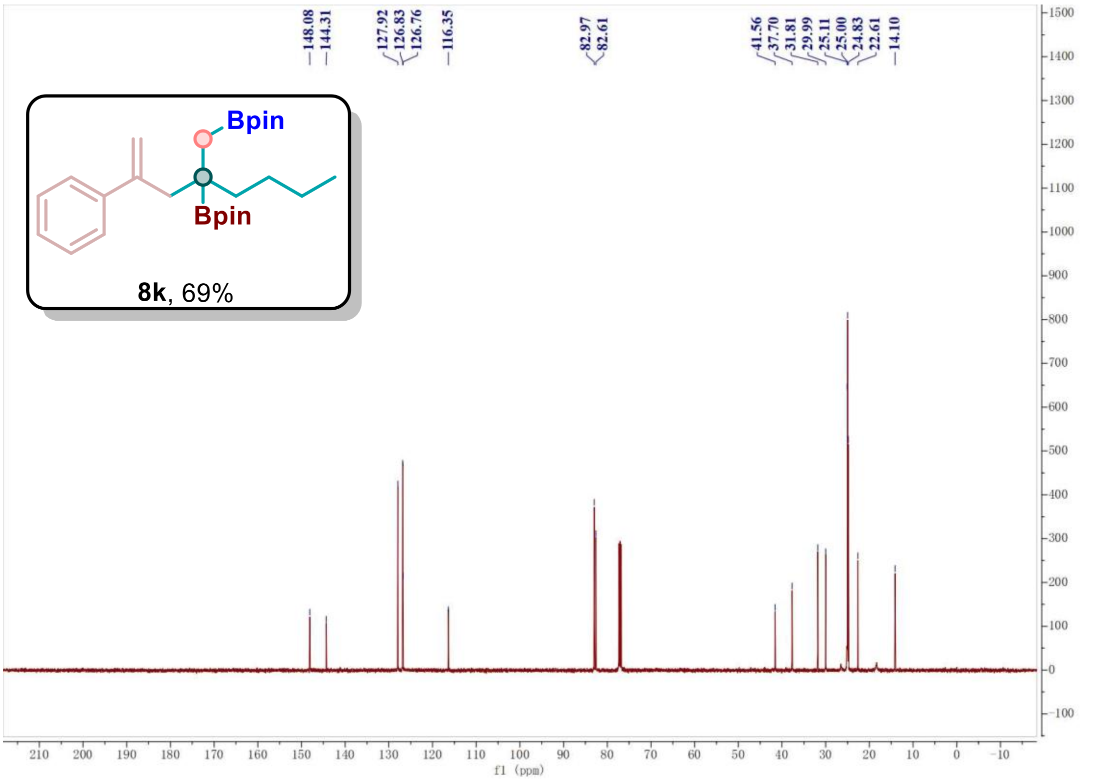

^{11}B NMR (160 MHz, Chloroform-*d*)

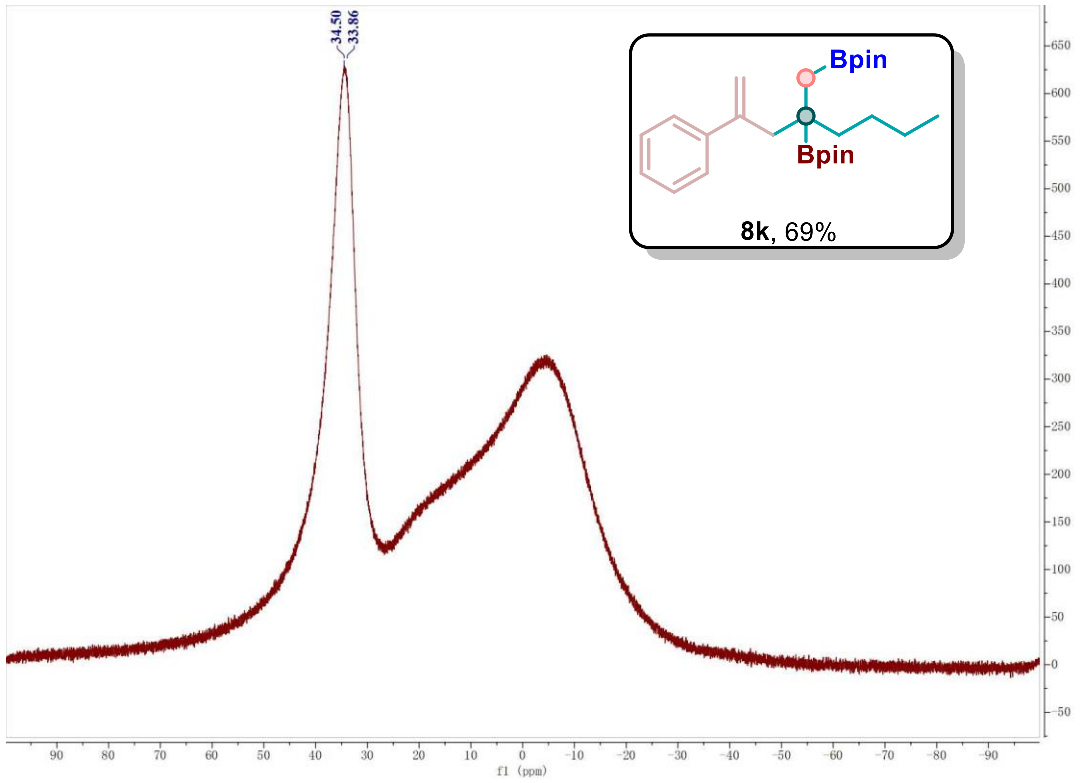

2,2'-(2-(2-chloroallyl)hexane-1,2-diyl)bis(4,4,5,5-tetramethyl-1,3,2-dioxaborolane) (8l)

^{1}H NMR (500 MHz, Chloroform-*d*)

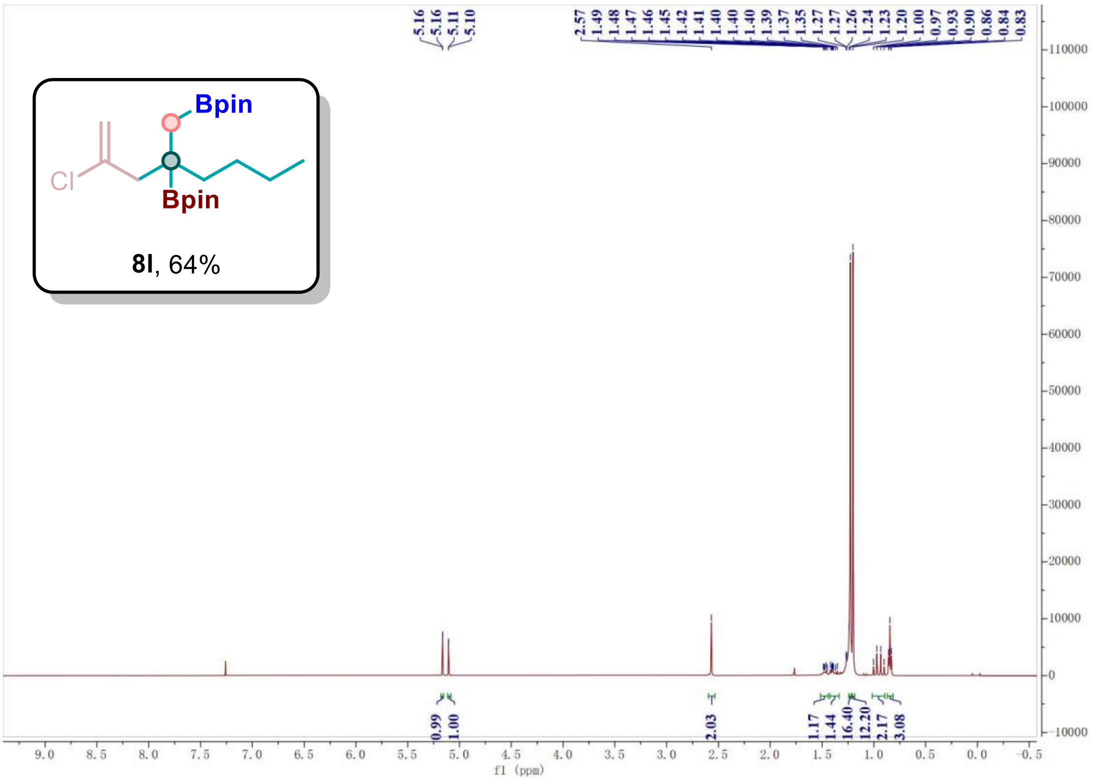

^{13}C NMR (126 MHz, Chloroform-*d*)

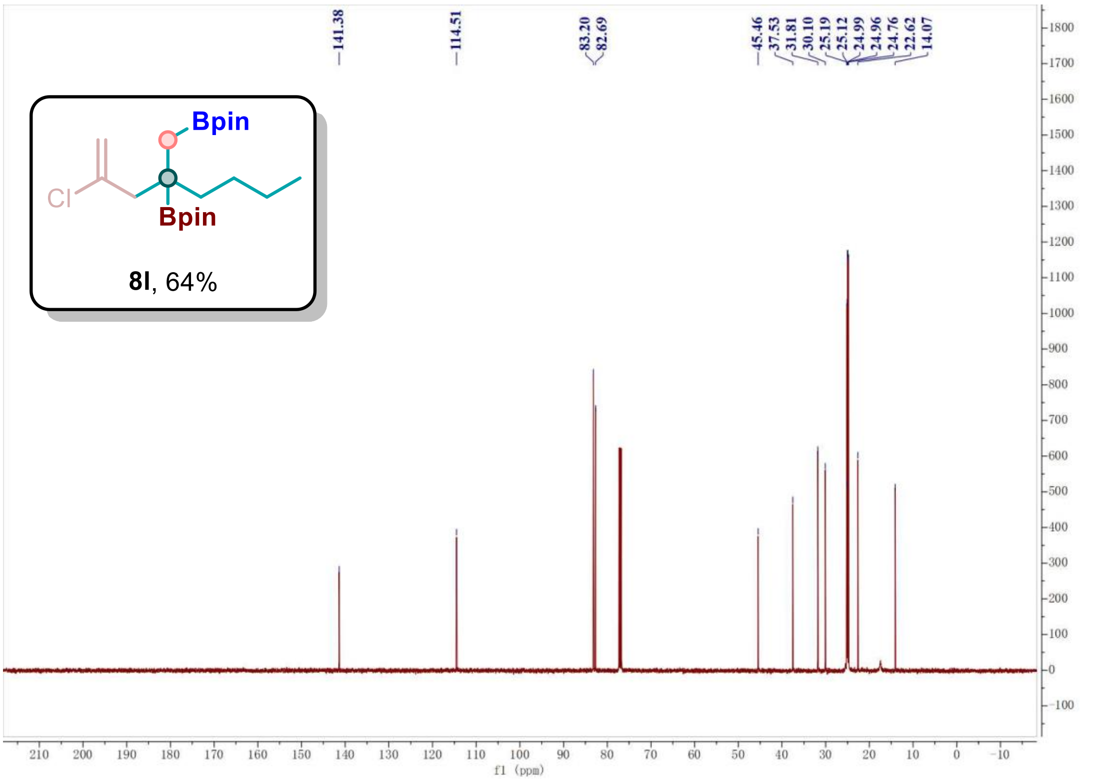

^{11}**B NMR (160 MHz, Chloroform-*d*)**

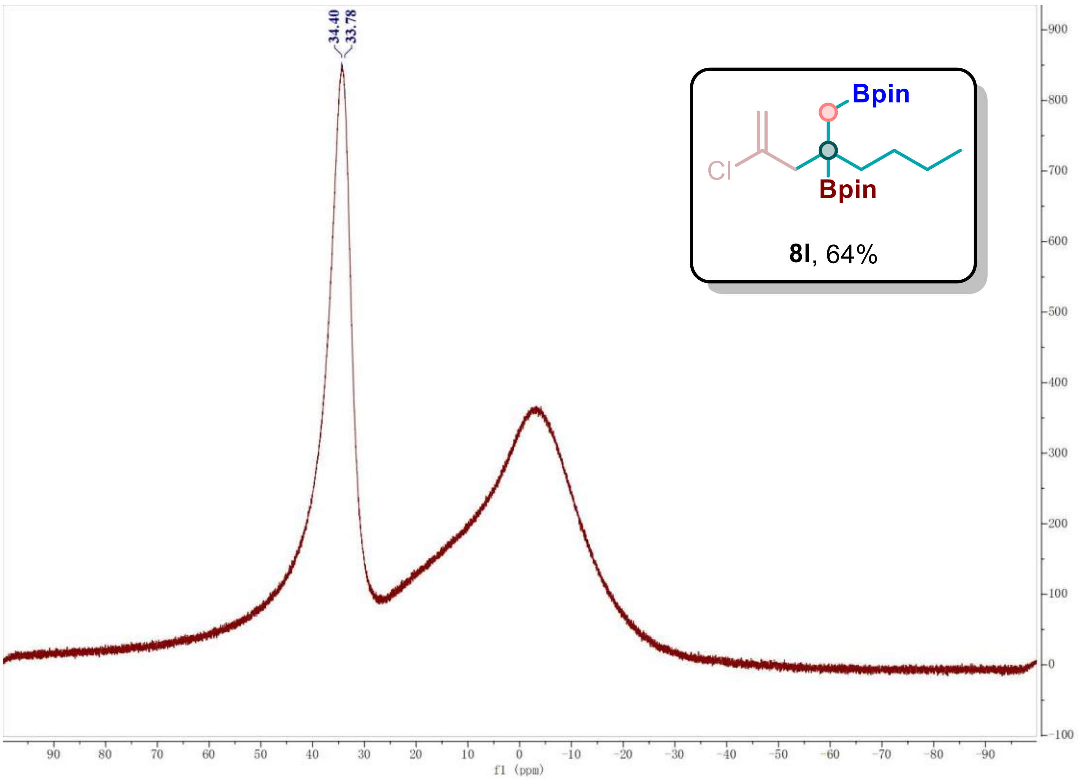

2-methyl-4-phenylbut-3-yne-1,2-diol (8m)

^{1}H NMR (500 MHz, Chloroform-*d*)

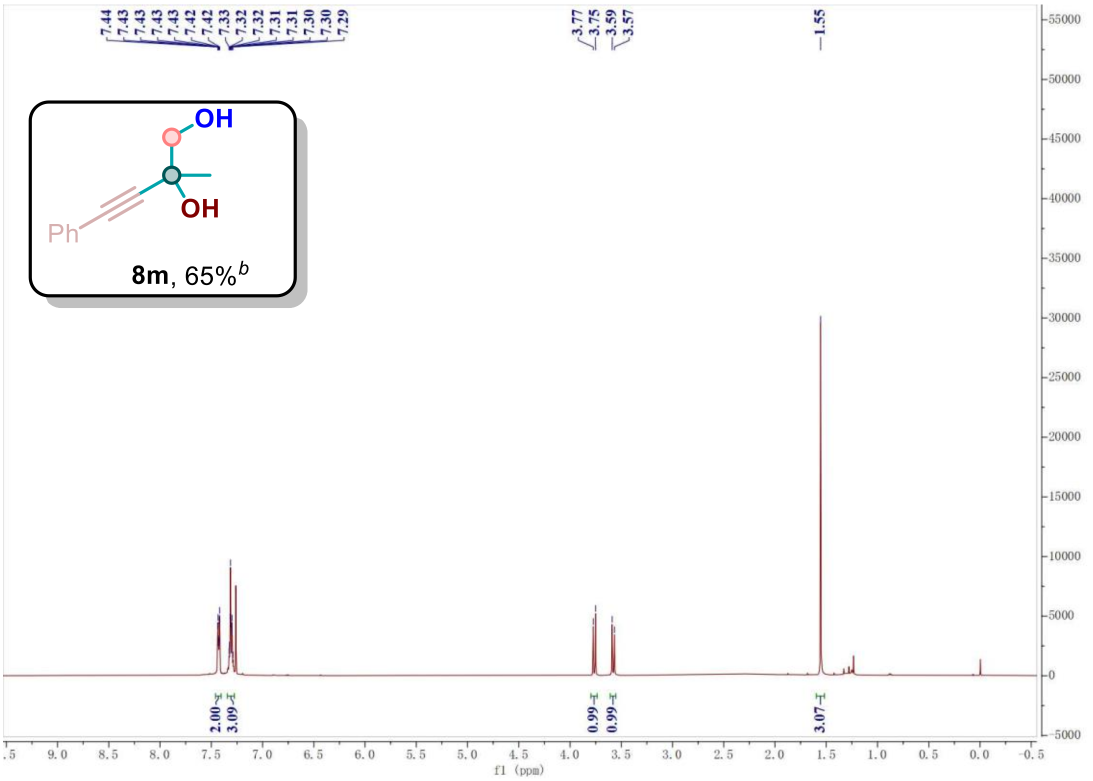

^{13}C NMR (126 MHz, Chloroform-*d*)

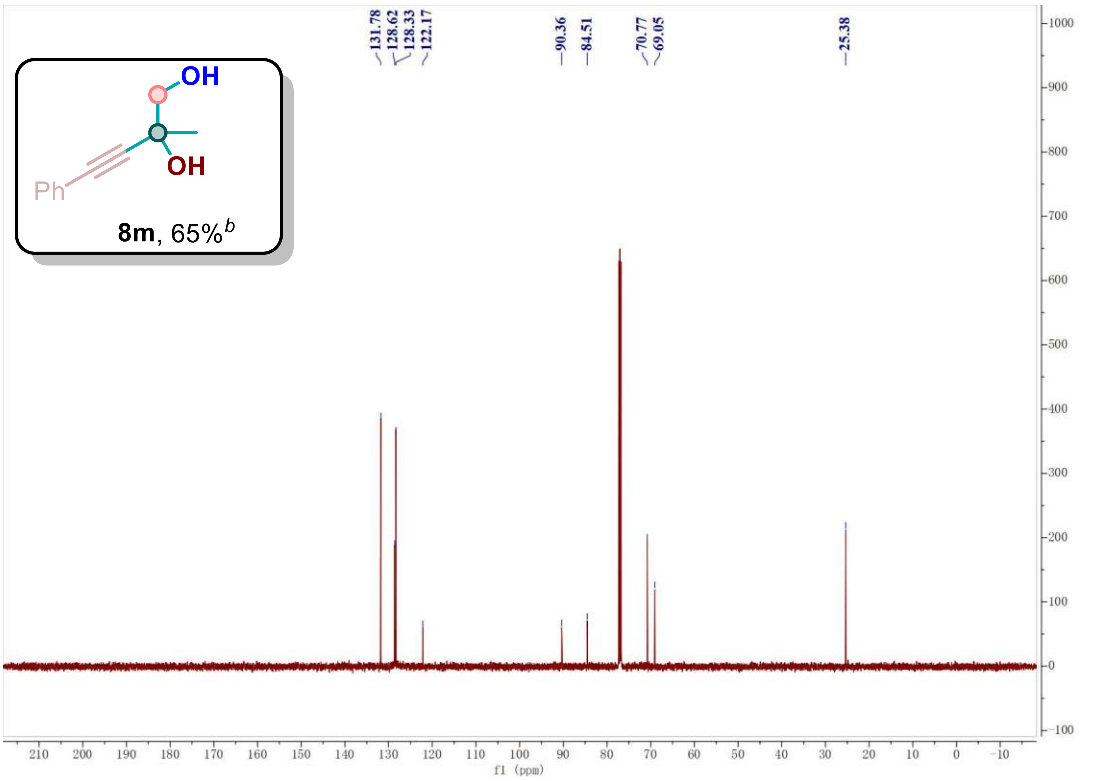

2-ethyl-4-phenylbut-3-yne-1,2-diol (8n)

^{1}H NMR (500 MHz, Chloroform-*d*)

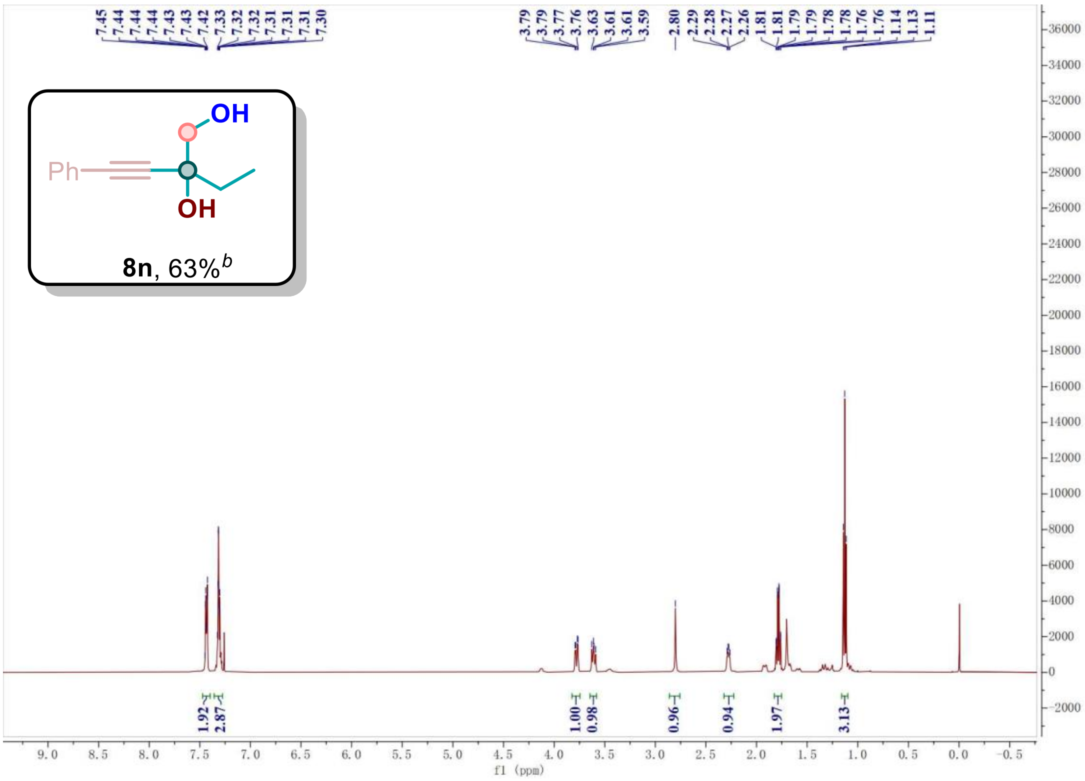

^{13}C NMR (126 MHz, Chloroform-*d*)

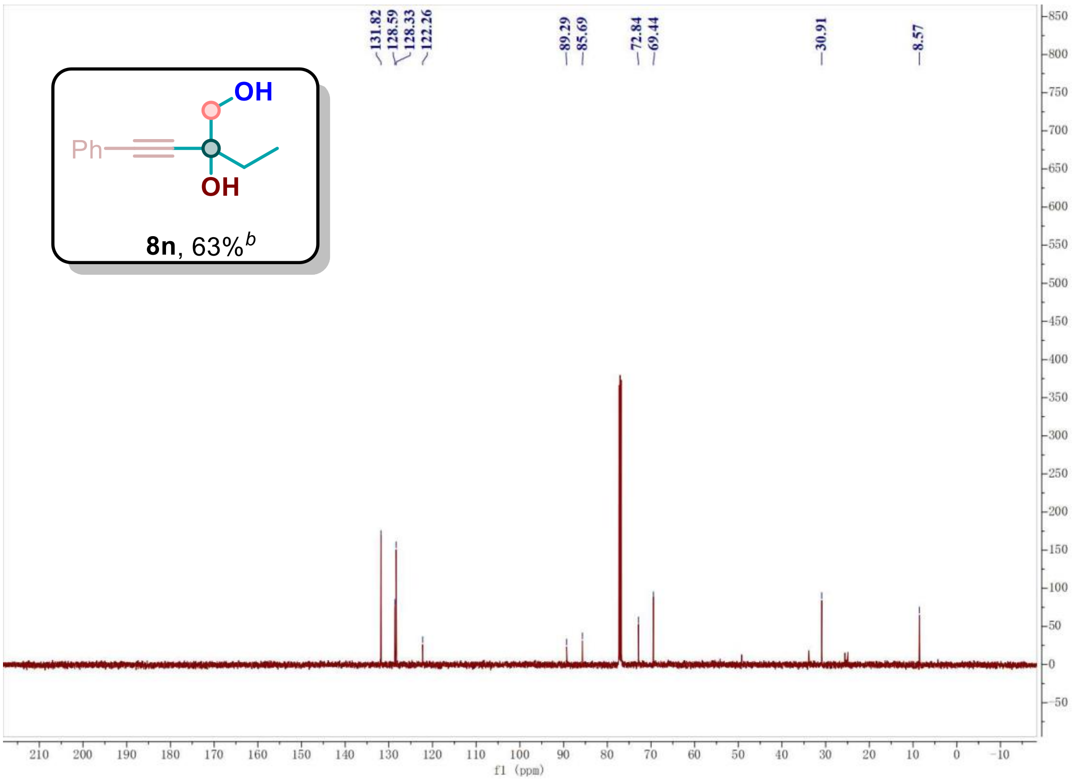

2-(phenylethynyl)hexane-1,2-diol (8o)

^{1}H NMR (500 MHz, Chloroform-*d*)

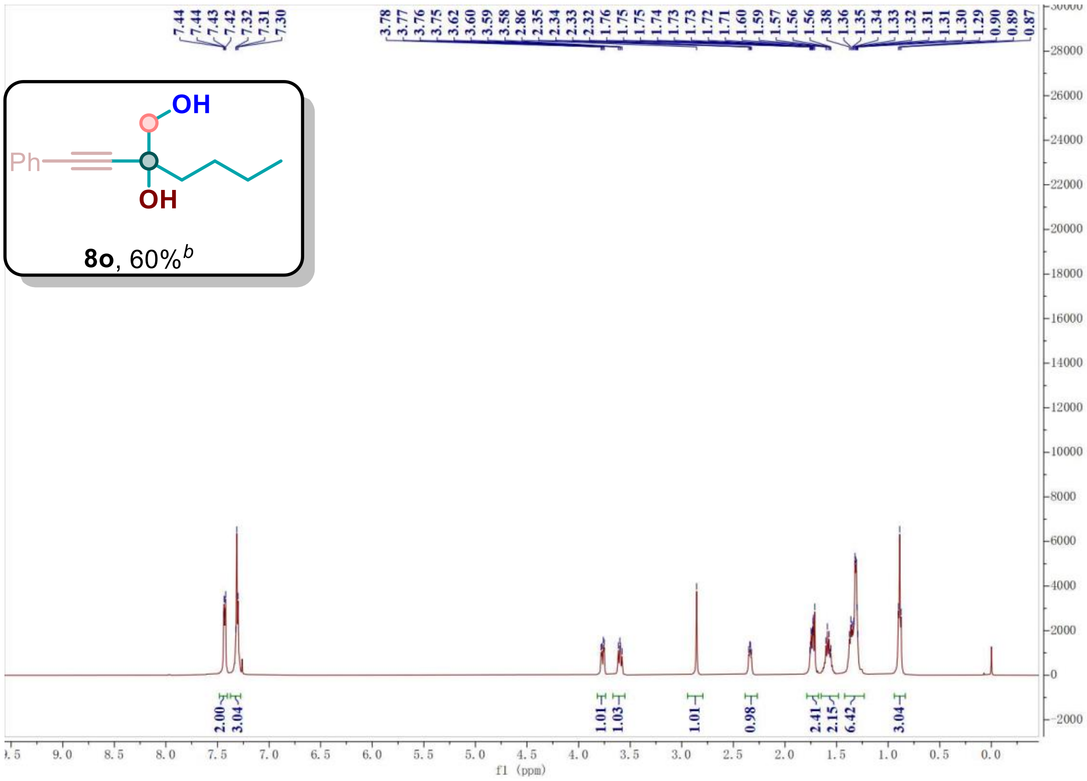

^{13}C NMR (126 MHz, Chloroform-*d*)

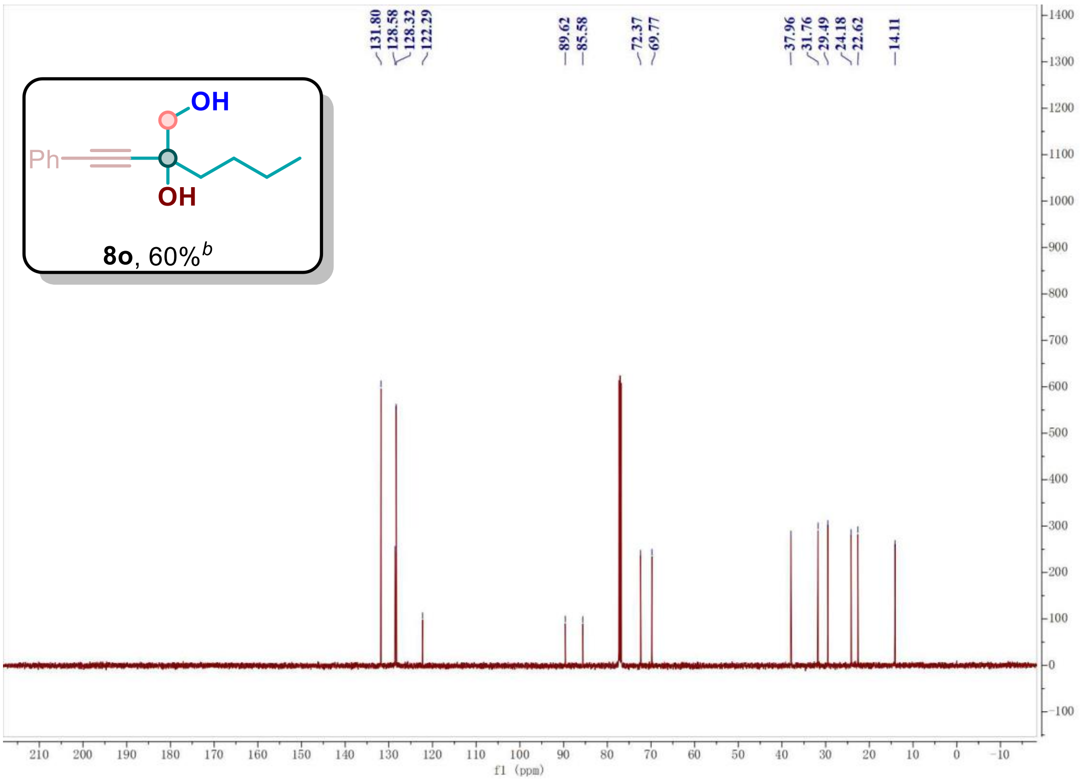

4,4-dimethyl-2-(phenylethynyl)pentan-1-ol (9)

^{1}H NMR (500 MHz, Chloroform-*d*)

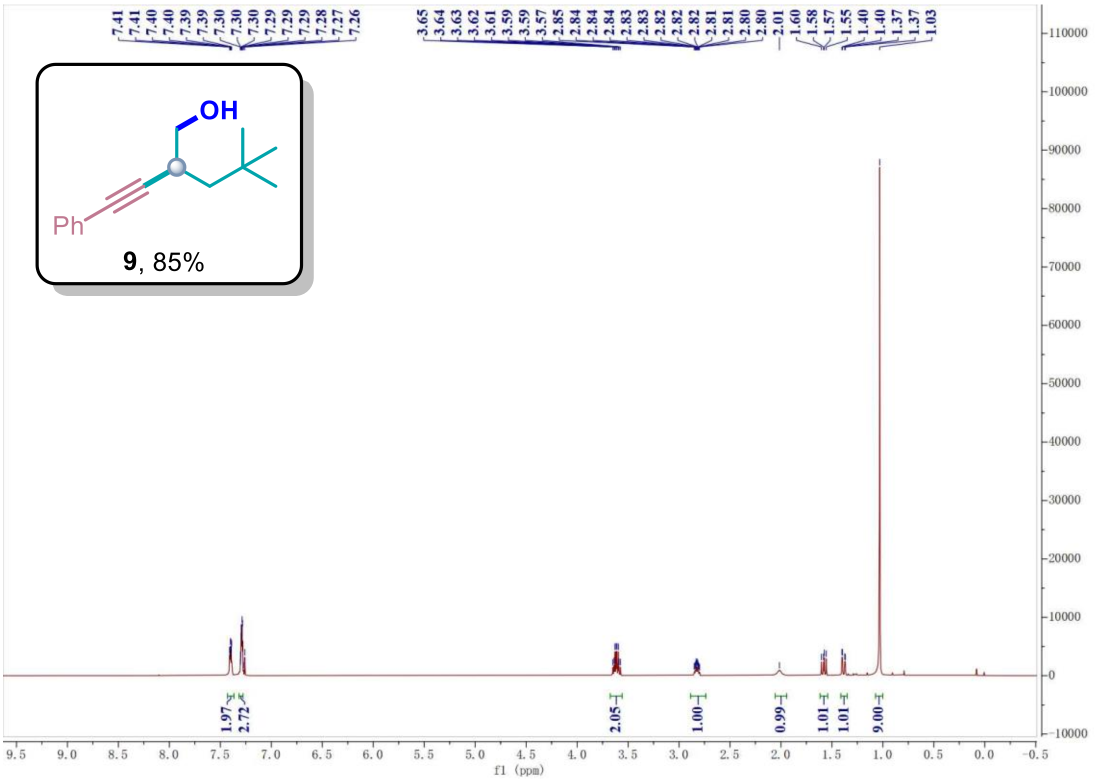

^{13}C NMR (126 MHz, Chloroform-*d*)

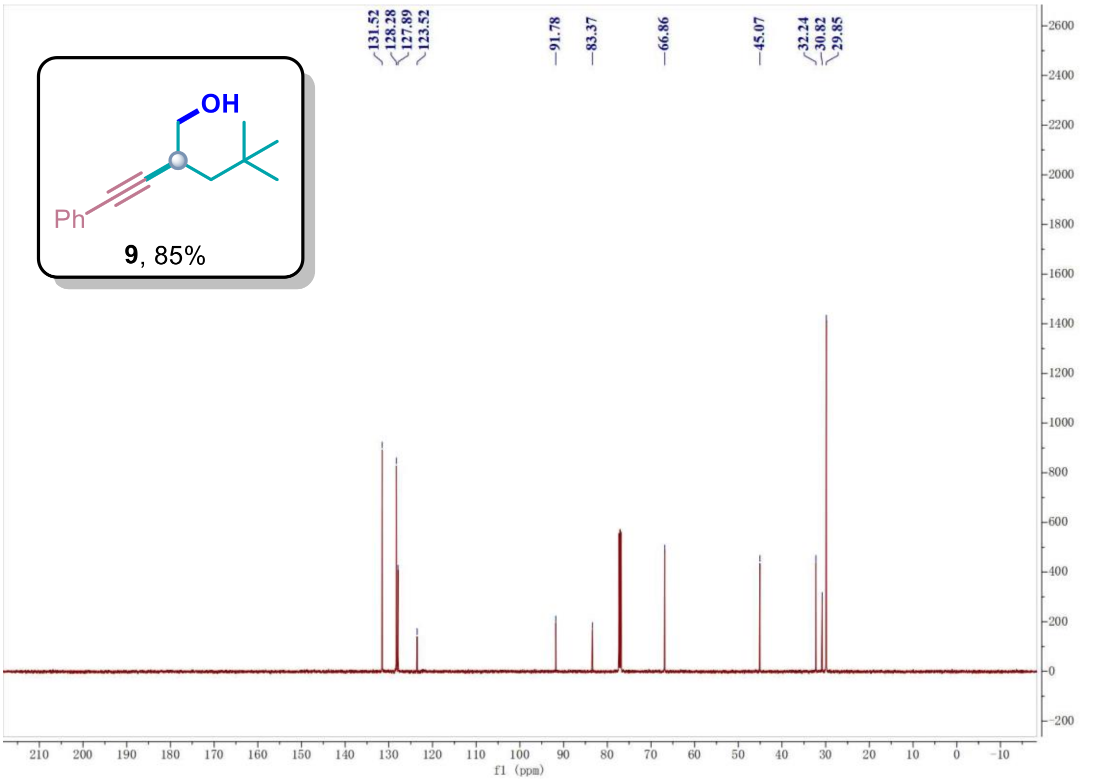

(4,4-dimethyl-2-(phenylethynyl)pentyl)trifluoro-l4-borane, potassium salt (10)

^{1}H NMR (500 MHz, Acetone-d_6)

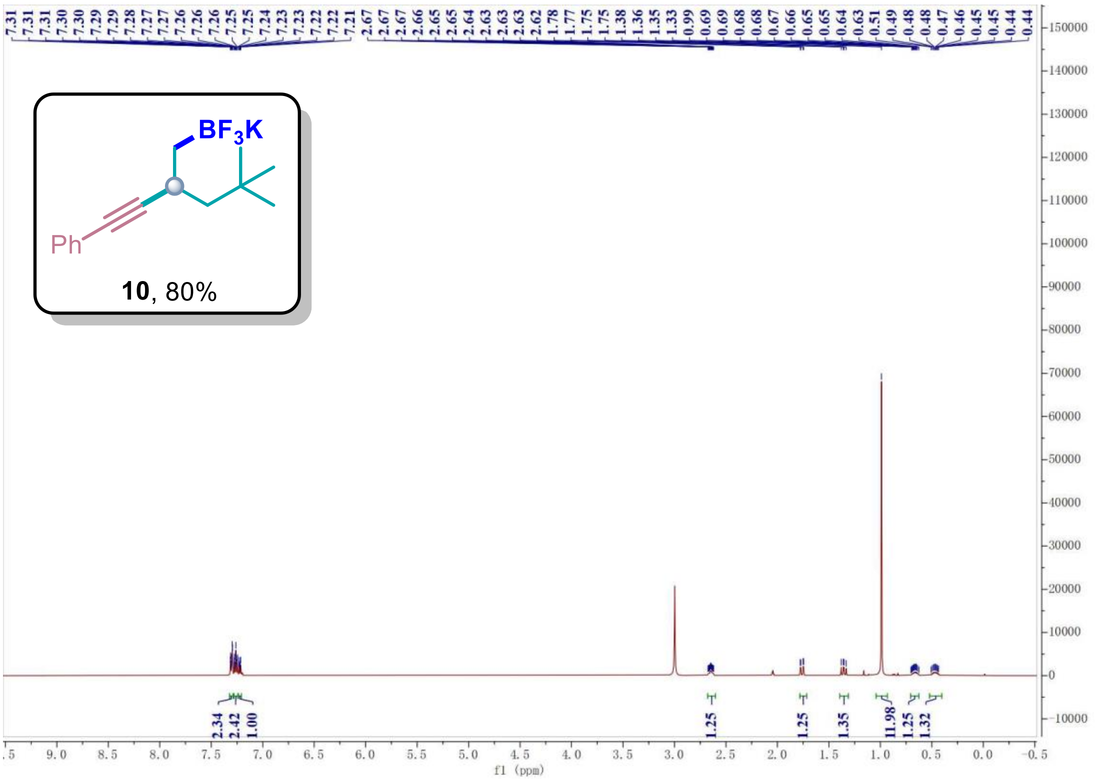

^{13}C NMR (126 MHz, Acetone-d_6)

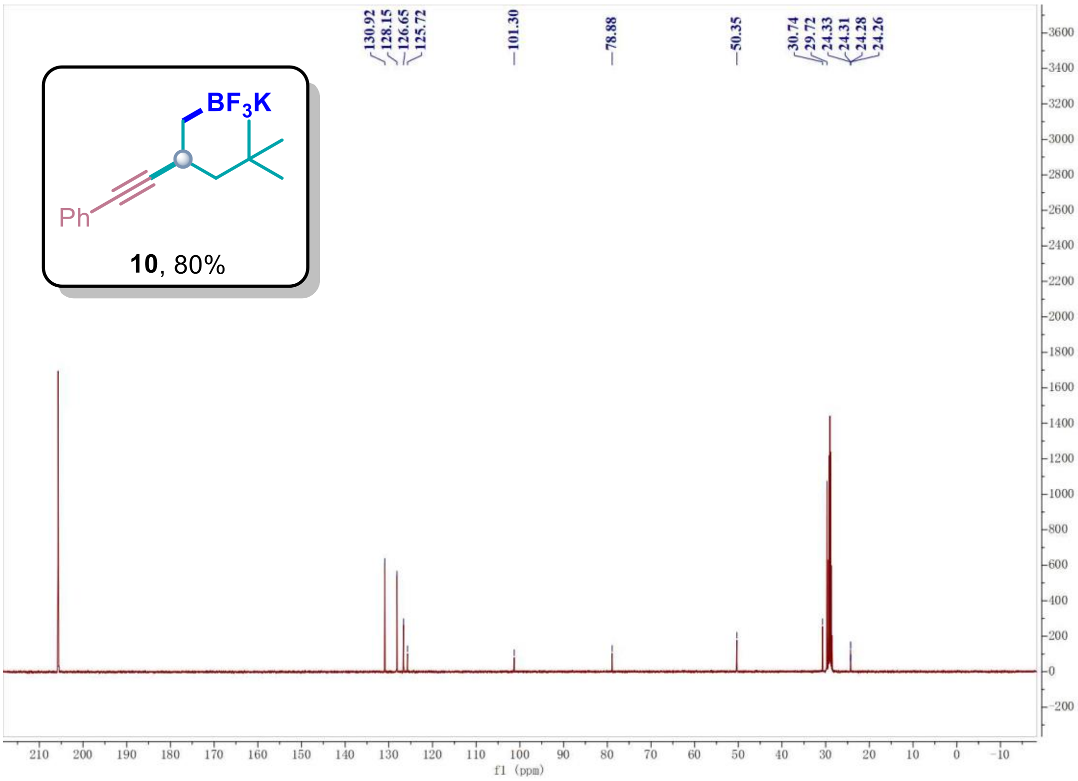

^{11}B NMR (160 MHz, Acetone-d_6)

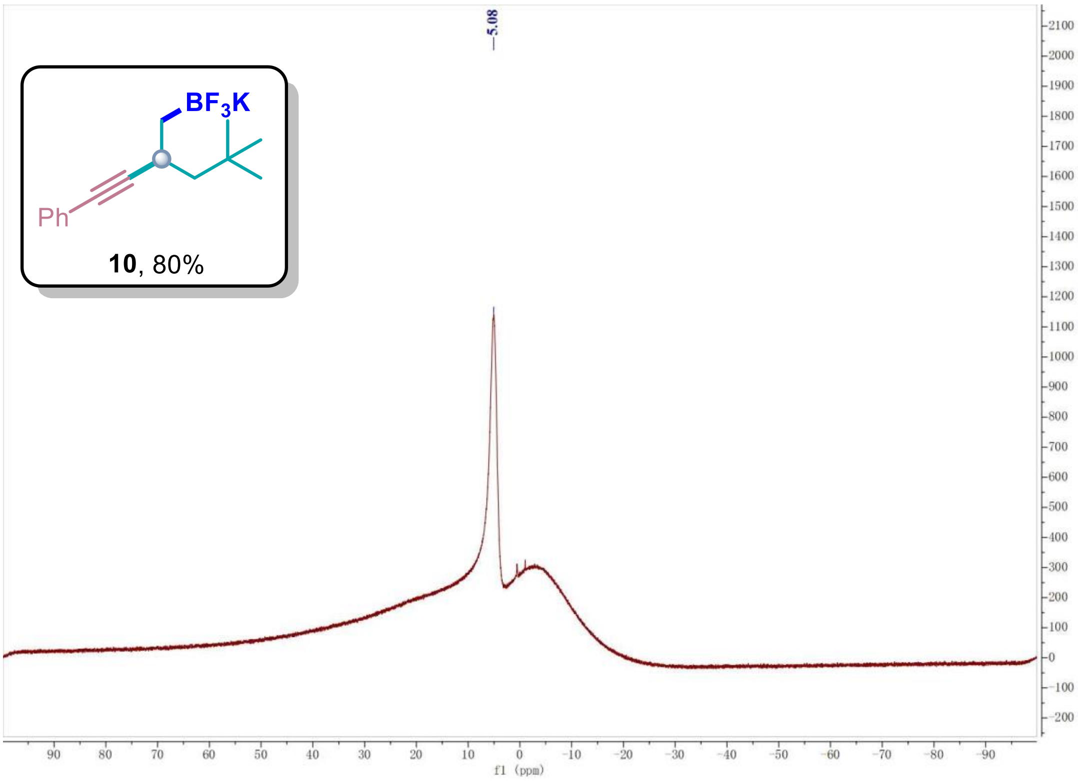

^{19}F NMR (471 MHz, Acetone-d_6)

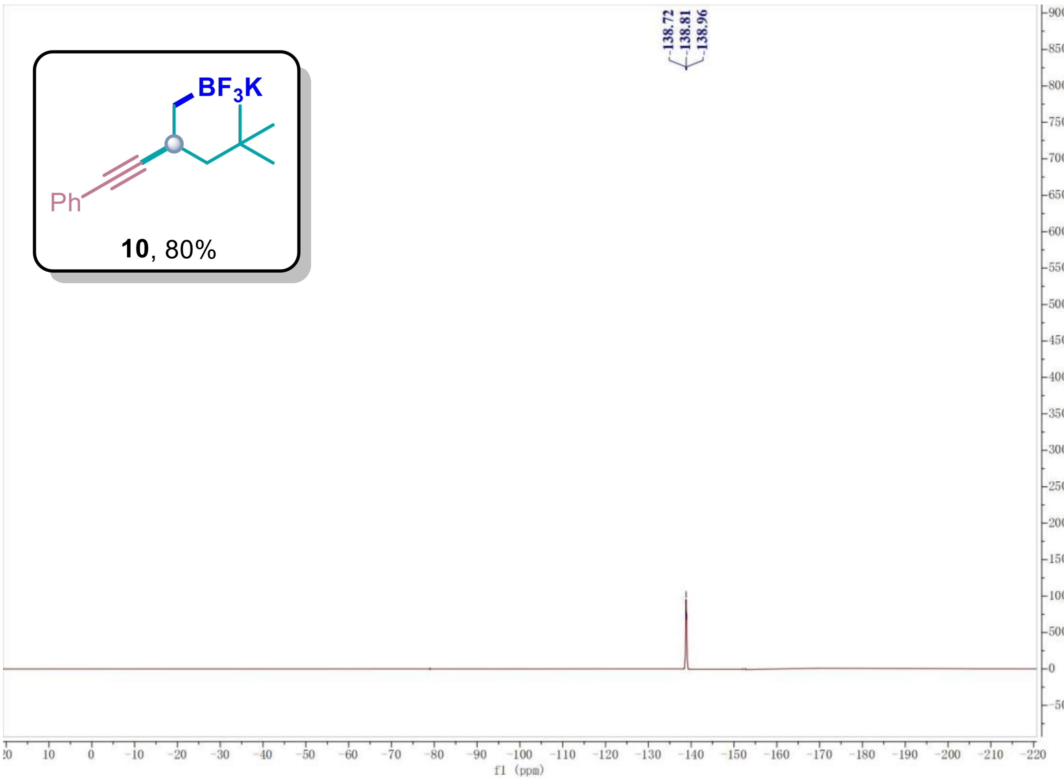

(3-neopentylhex-5-en-1-yn-1-yl)benzene (11)

^{1}H NMR (500 MHz, Chloroform-*d*)

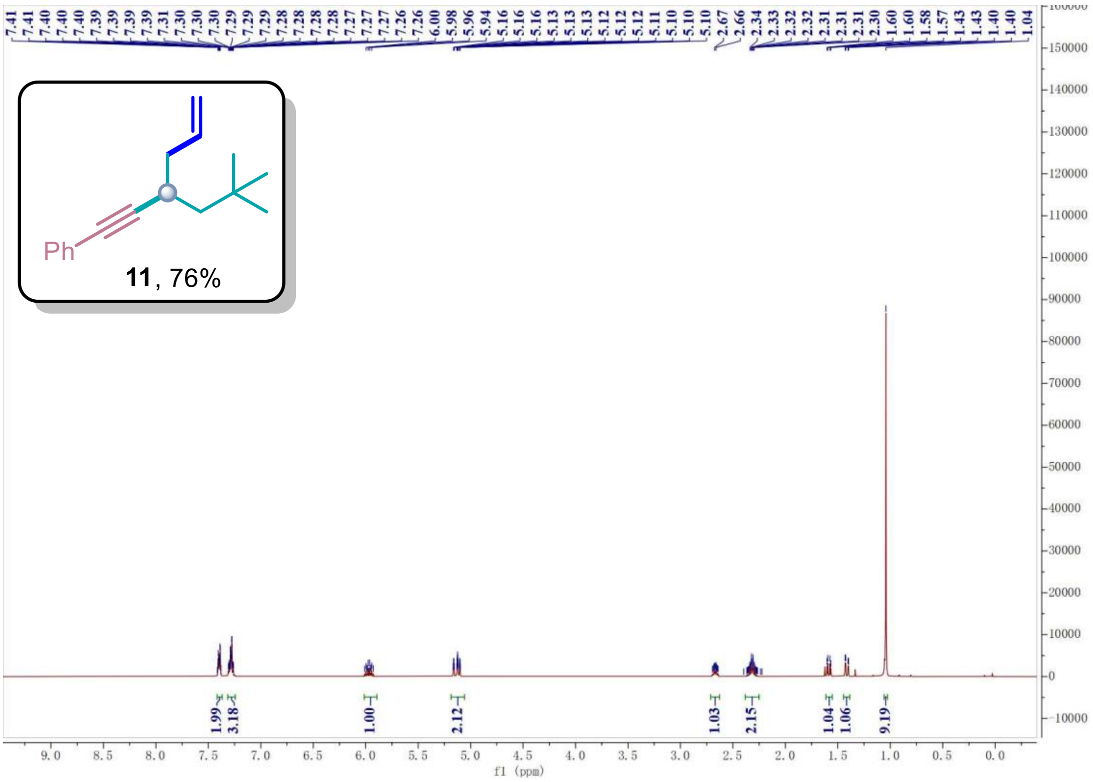

^{13}C NMR (126 MHz, Chloroform-*d*)

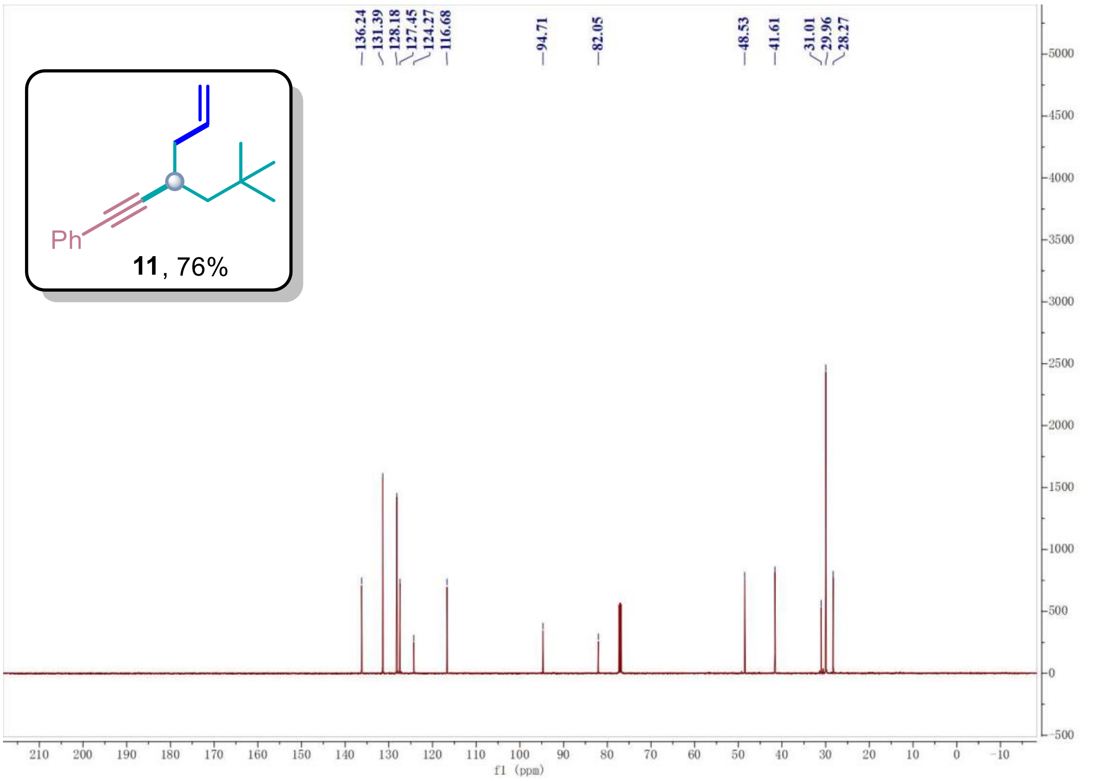

1-(4,4-dimethyl-2-(phenylethynyl)pentyl)-4-methoxybenzene (12)

^{1}H NMR (500 MHz, Chloroform-*d*)

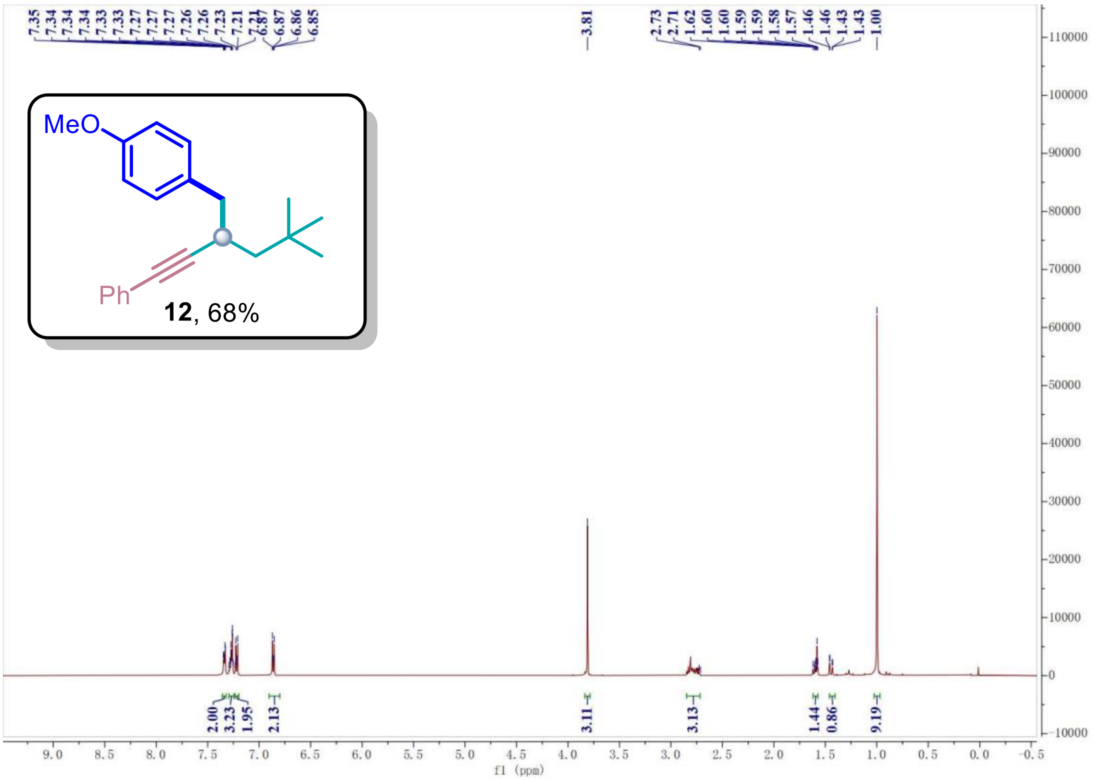

^{13}C NMR (126 MHz, Chloroform-*d*)

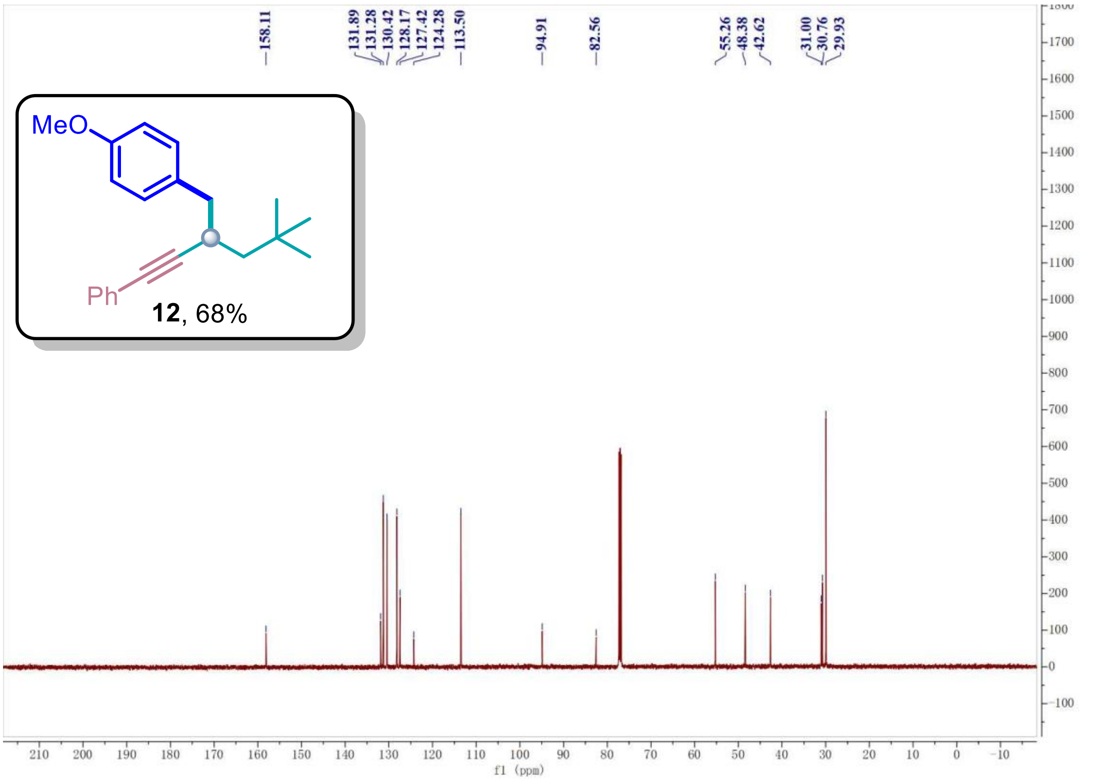

2-(4,4-dimethyl-2-(phenylethynyl)pentyl)naphthalene (13)

^{1}H NMR (500 MHz, Chloroform-*d*)

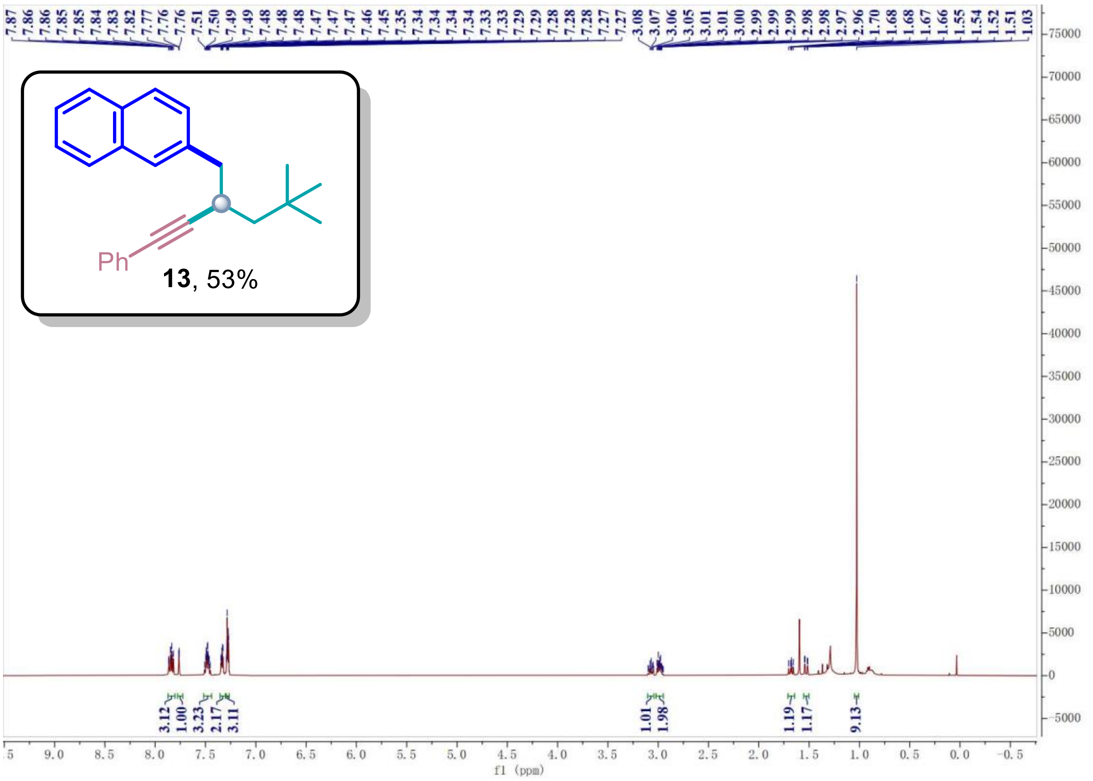

^{13}C NMR (126 MHz, Chloroform-*d*)

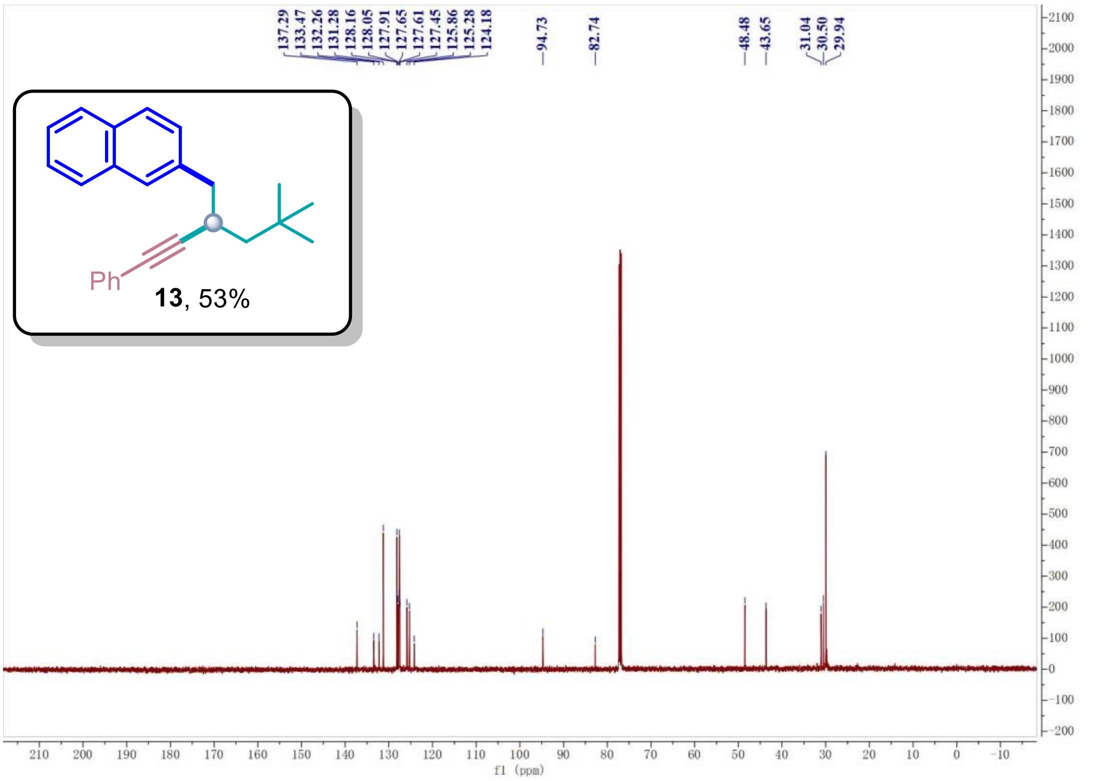

2-(4,4-dimethyl-2-(phenylethynyl)pentyl)furan (14)

^{1}H NMR (500 MHz, Chloroform-*d*)

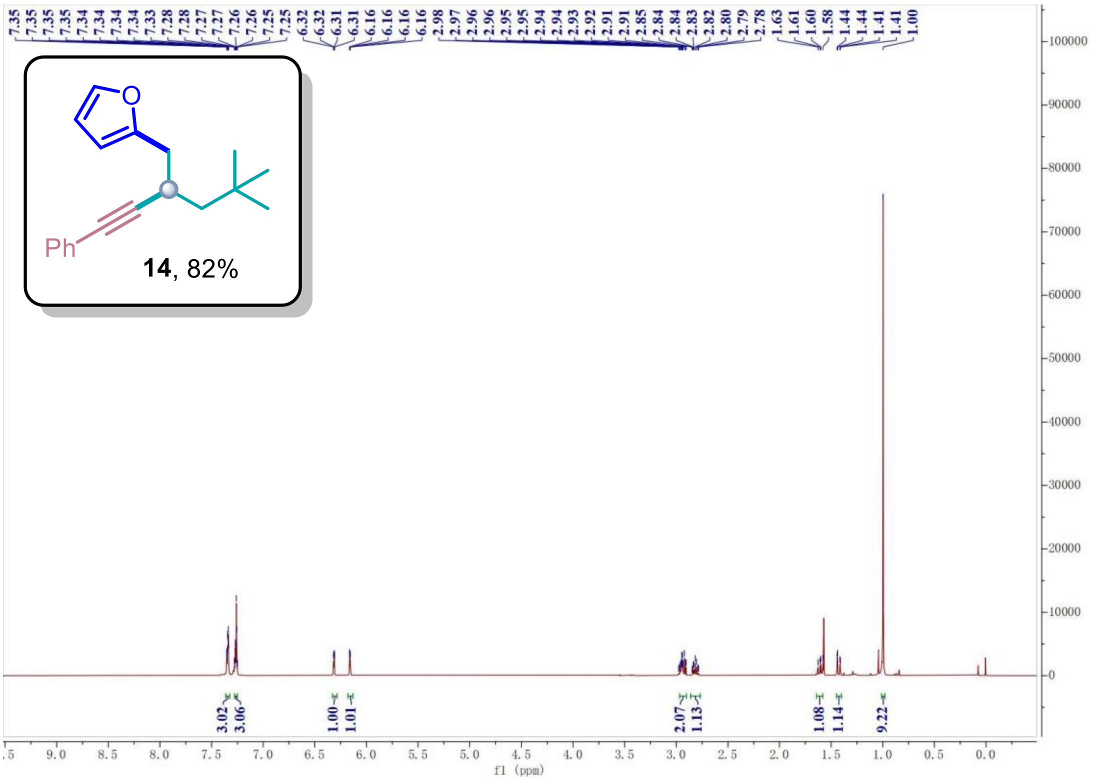

^{13}C NMR (126 MHz, Chloroform-*d*)

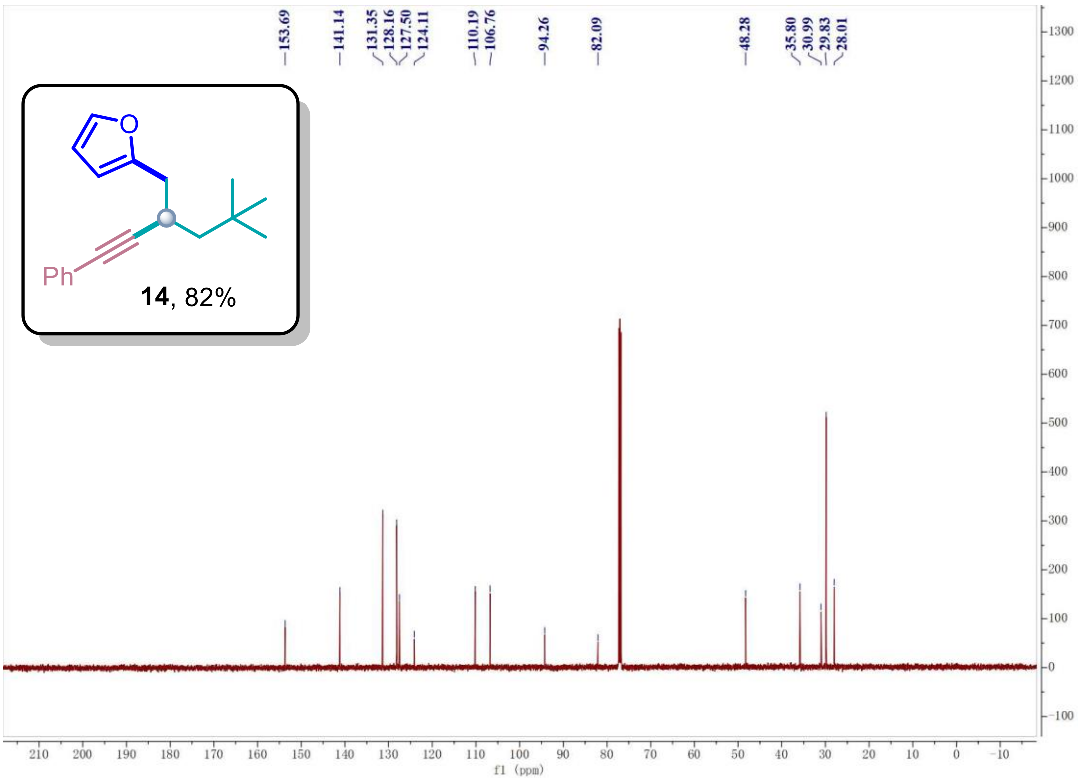

2-(4,4-dimethyl-2-(phenylethynyl)pentyl)thiophene (15)

^{1}H NMR (500 MHz, Chloroform-*d*)

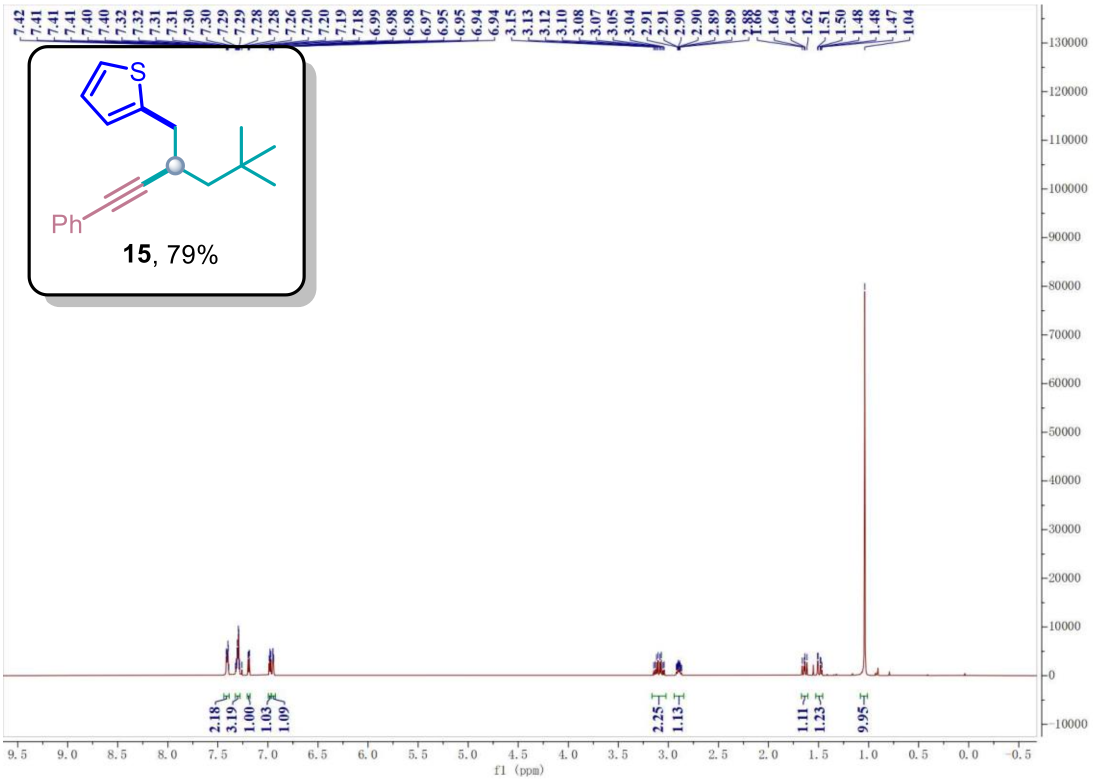

^{13}C NMR (126 MHz, Chloroform-*d*)

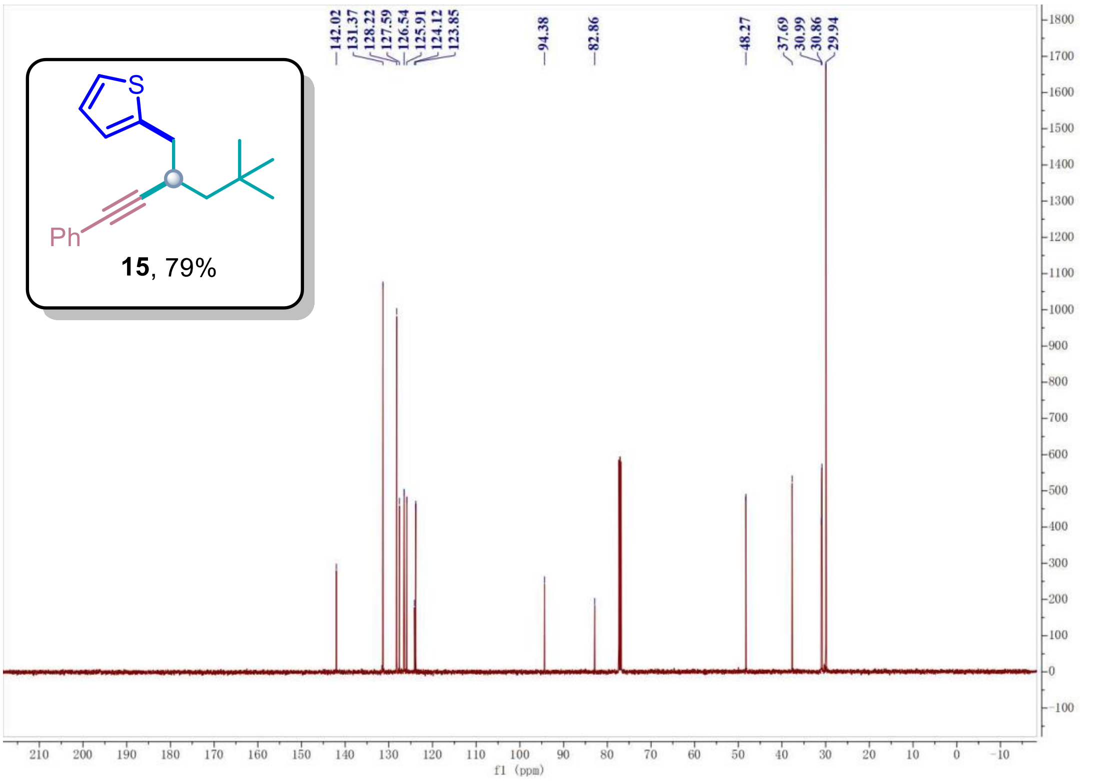

2-(4,4-dimethyl-2-phenethylpentyl)-4,4,5,5-tetramethyl-1,3,2-dioxaborolane (16)

^{1}H NMR (500 MHz, Chloroform-*d*)

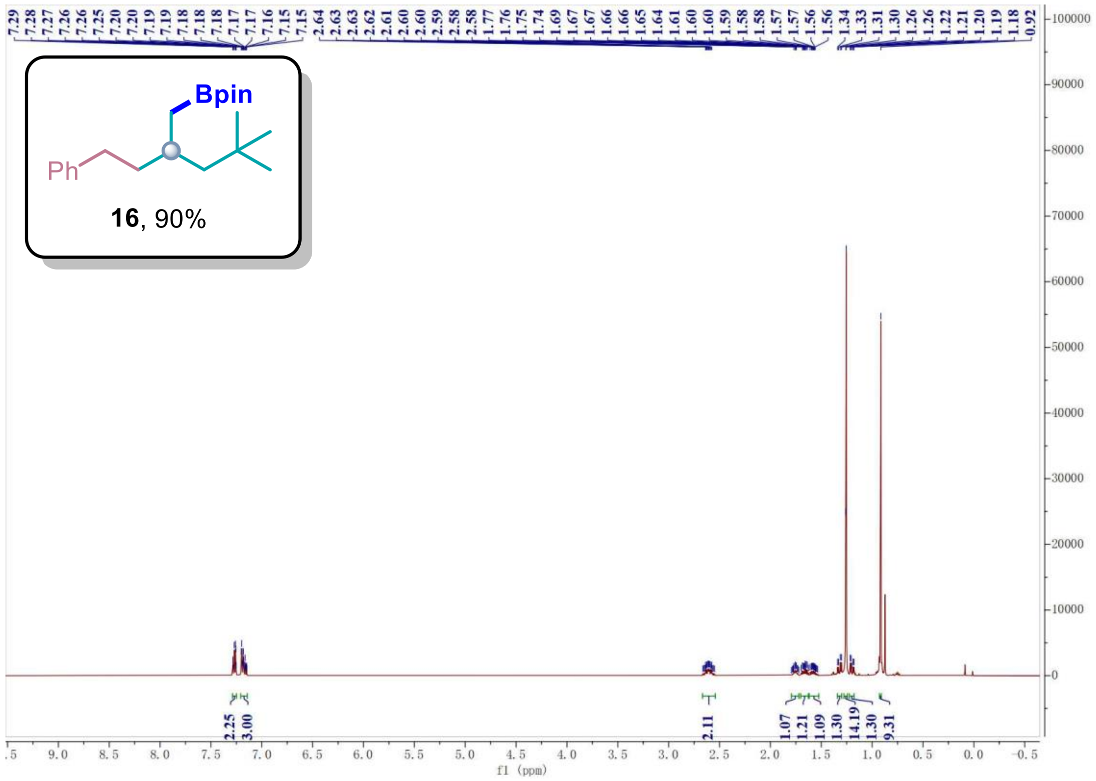

^{13}C NMR (126 MHz, Chloroform-*d*)

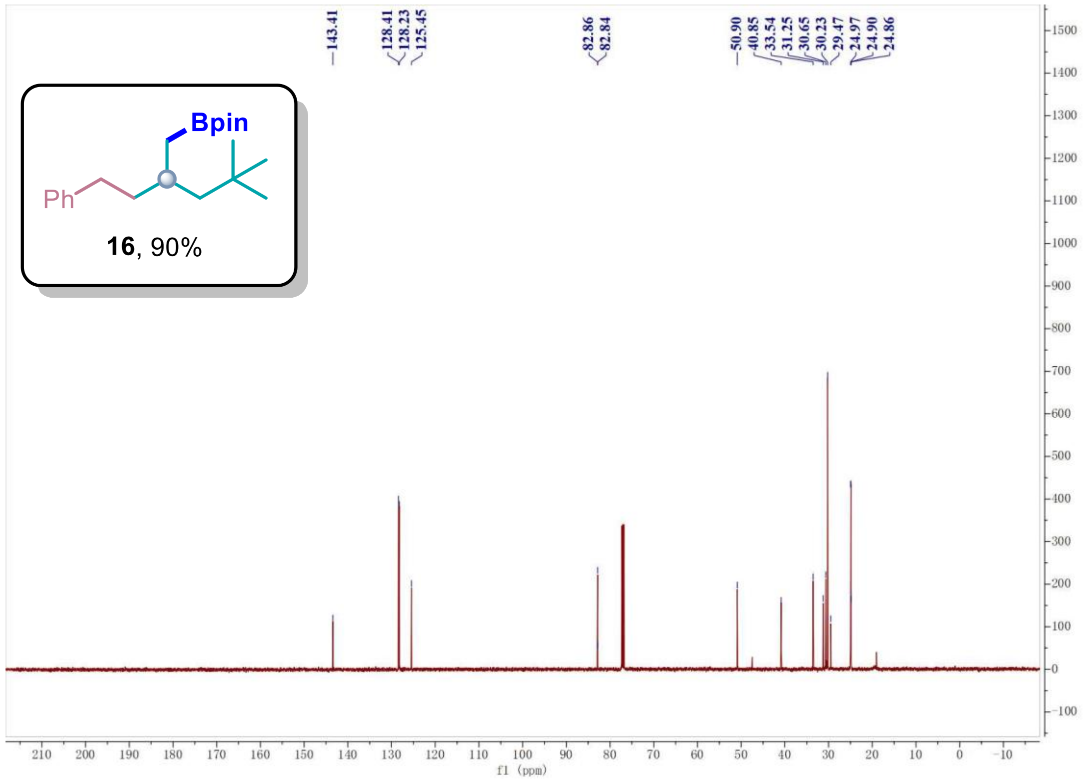

^{11}B NMR (160 MHz, Chloroform-*d*)

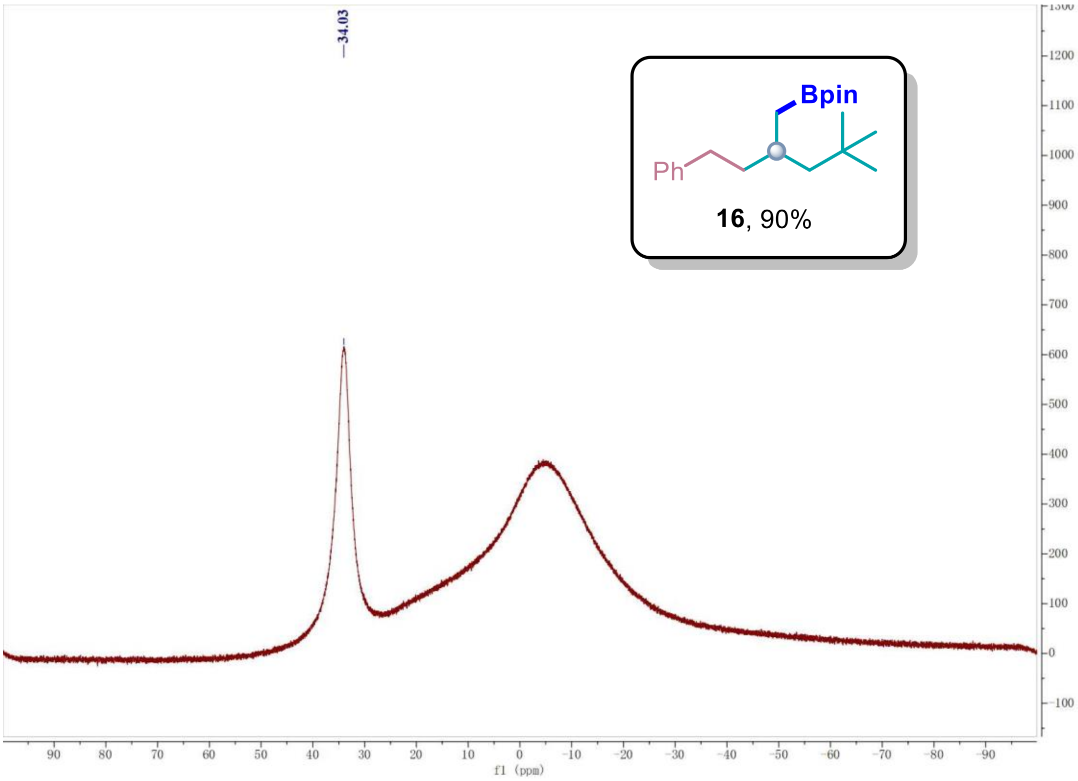

2.7 References

(1) Yoshida, M.; Otaka, H.; Doi, T., *Eur. J. Org. Chem.* **2014**, *2014*, 6010-6016.

(2) a. Mun, S., Lee, J.-E. & Yun, J. *Org. Lett.* **2006**, *8*, 4887-4889. b. Netsu, Y.; Tsukada, N., *Lett. Org. Chem.,* **2017**, 14, 243-247.

(3) Knott, K.; Fishovitz, J.; Thorpe, S. B.; Lee, I.; Santos, W. L., *Org. Biomol. Chem.* **2010**, *8*, 3451-3456.

(4) Chen, X.; Ye, F.; Luo, X.; Liu, X.; Zhao, J.; Wang, S.; Zhou, Q.; Chen, G.; Wang, P., *J. Am. Chem. Soc.* **2019**, *141*, 18230-18237.

(5) Messin, J; Katrun, P.; Pareseecharoen, C.; Pohmakotr, M.; Reutrakul, V.; Soorukram, D.; Kuhakarn, C. *J. Org. Chem.* **2016**, *81,* 2744

(6) Sawangphon, T.; Katrun, P.; Chaisiwamongkhol, K.; Pohmakotr, M.; Reutrakul, V.; Jaipetch, T.; Soorukram, D.; Kuhakarn, C., *Synth. Commun.* **2013**, *43*, 1692-1707.

(7) Lou, Y.; Qiu, J.; Yang, K.; Zhang, F.; Wang, C.; Song, Q., *Org. Lett.* **2021**, *23*, 4564-4569.

(8) Kuang, Z.; Chen, H.; Yan, J.; Yang, K.; Lan, Y.; Song, Q. *Org. Lett.* **2018**, *20*, 5153-5157.

(9) Cao, Z.-C.; Luo, F.-X.; Shi, W.-J.; Shi, Z.-J. *Org. Chem. Front.* **2015**, *2*, 1505−1510.

(10) Chen, Y.; Ma, G.; Gong, H. *Org. Lett.* **2018**, *20*, 4677-4680.

(11) Ma, W.; Zhang, X.; Fan, J.; Liu, Y.; Tang, W.; Xue, D.; Li, C.; Xiao, J.; Wang, C. *J. Am. Chem. Soc.* **2019**, *141*, 13506-13515.

(12) Bai, X.-Y.; Zhao, W.; Sun, X.; Li, B.-J. *J. Am. Chem. Soc.* **2019**, *141*, 19870-19878.

(13) Zhang, Z.-H.; Dong, X.-Y.; Du, X.-Y.; Gu, Q.-S.; Li, Z.-L.; Liu, X.-Y., *Nat. Commun.* **2019**, *10*, 5689.
